# Supplementary material for: Water mediated redox-neutral cleavage of arylalkenes via photoredox catalysis
Source: Nat Commun. 2024 Jul 23;15:6227. doi: 10.1038/s41467-024-50624-2 (PMC11266562; doi:10.1038/s41467-024-50624-2)
Supplement: Supplementary file 1 — Supplementary Information [file 41467_2024_50624_MOESM1_ESM.pdf]

Supplementary Information

Water Mediated Redox-Neutral Cleavage of Arylalkenes via Photoredox Catalysis

Table of Contents

Supplementary Information 1
1. General Information 2
2. Preparation of Starting Materials 2
2.1 Synthesis of the phosphonium salt 2
2.2 Synthesis of Ester 2
2.3 Synthesis of Olefins 3
3. Condition screening 23
4. General procedure 26
4.1 General method for isolating the reducing fragment 26
4.2 General method for isolating the oxidating fragment 26
4.3 Reaction conditions of gram scale 27
5. Experiment for KIE 48
6. ^{18}O labeling experiment 49
7. Plausible mechanisms 49
8. ^{1}H NMR and ^{13}C NMR spectra 50
9. References 181

1. **General Information**

All solvents were distilled according to general practice before use. Solvents for flash column chromatography were technical grade and distilled before use. ^{1}H NMR and ^{13}C NMR data were recorded on Bruker, 400 MHz (101 MHz for ^{13}C) nuclear resonance spectrometers unless otherwise specified. ^{1}H and ^{13}C NMR chemical shifts are given in ppm relative to $SiMe_4$, with the solvent resonance used as the internal reference. Chemical shifts (δ) are given in parts per million and referenced to the residual solvent signal; all coupling constants are reported in Hz. The following abbreviations were used to explain the multiplicits: s (singlet), d (doublet), t (triplet), q (quartet), m (multiplet). Thin-layer chromatography (TLC) was conducted with 0.25 mm Yantai silica gel plates (60F-254) and visualized by exposure to UV light (254 nm) or stained with phosphomolybdic acid in EtOH. Flash column chromatography was performed using Tsingdao silica gel (60, particle size 0.040–0.063 mm). HRMS (ESI) analysis was performed by The Analytical Instrumentation Center at Peking University, Shenzhen Graduate School and (HRMS) data were reported with ion mass/charge (m/z) ratios as values in atomic mass units.

2. Preparation of Starting Materials

2.1 Synthesis of the phosphonium salt

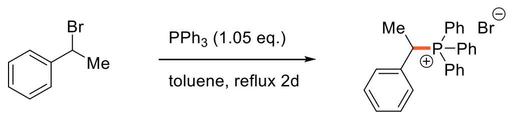

In a round-bottom flask charged with a magnetic stir bar, PPh_3 (1.05 equiv.) was added to a solution of 1-Bromoethylbenzene (1.0 equiv.) in toluene (1.0 M). The well-mixed reaction mixture was placed in an oil bath at 110 °C (pre-heated) for 2 days. The mixture was filtered, and the obtained white precipitate was washed with diethyl ether and dried in the vacuum to give the corresponding phosphonium salt. The NMR spectroscopic data are in accord with the literature[1]. Unless otherwise noted, the phosphonium salts used below were purchased from commercial sources and used without further purification.

2.2 Synthesis of Ester

General Procedure A

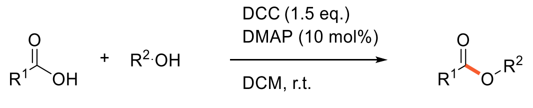

Following a typical esterification reaction procedure, in a round-bottom flask charged with a magnetic stir bar, alcohol (1.0 equiv.), acid (1.0-1.2 equiv.), DCC (1.5 equiv.) and DMAP (10 mol%)

in dichloromethane (0.2M) was stirred at room temperature for 24 h. The mixture was filtered, and the filtrate was washed with water dried over anhydrous Na_2SO_4 and concentrated in vacuo. The crude residue was purified by chromatography on silica gel eluting with a petroleum ether/ethyl acetate gradient to afford the corresponding ester compound.

2.3 Synthesis of Olefins

General Procedure B for substrates **8a-15a, 27-28a, 35a-46a, 49a, 55a, 60a, 62a-64a, 79a**:

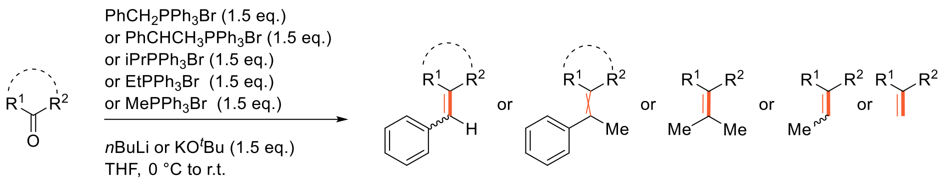

Following a typical wittig reaction procedure, in a round-bottom flask charged with a magnetic stir bar, phosphonium salt (1.5 equiv.) was suspended in dry THF (0.2M) under Ar and then cooled to 0 °C. Base (1.5 equiv.) was added dropwise under stirring, and the red mixture was stirred for 1 h. The corresponding aldehyde or ketone derivative (1.0 equiv.) in THF was added dropwise while keeping the internal temperature at 0 °C. After the addition, the mixture was allowed to warm to room temperature and stirred overnight. The reaction was quenched by the addition of water. The layers were separated, and the aqueous layer was extracted with ethyl acetate (3×20 mL). The combined organic layers were washed with brine, dried over anhydrous Na_2SO_4, filtered, and concentrated in vacuo. The crude residue was purified by chromatography on silica gel eluting with a petroleum ether/ethyl acetate gradient or petroleum ether to afford the corresponding olefin.

General Procedure C for substrates **1a-7a, 23a-26a, 47a-48a, 50a-54a**:

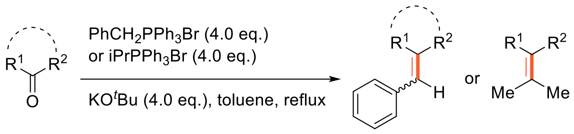

According to reported procedures[2], in a round-bottom flask charged with potassium tert-butoxide (4.0 equiv.) and stir bar, dry DMSO (0.25M) was added, followed by the corresponding phosphonium salt (4.0 equiv.). This mixture was added dropwise with a syringe to a solution of the corresponding ketone (1.0 equiv.) in toluene (0.3 M). The reaction was heated to reflux under Ar overnight. It was allowed to cool to room temperature and petroleum ether and water were added. The organic phase was separated and washed with water (2x25 mL) and brine (25 mL), dried over anhydrous Na_2SO_4, filtered, and concentrated in vacuo. The crude residue was purified by chromatography on silica gel eluting with a petroleum ether/ethyl acetate gradient or petroleum ether to afford the corresponding olefin.

Procedure D for substrates **14a-OH** and **16a** :

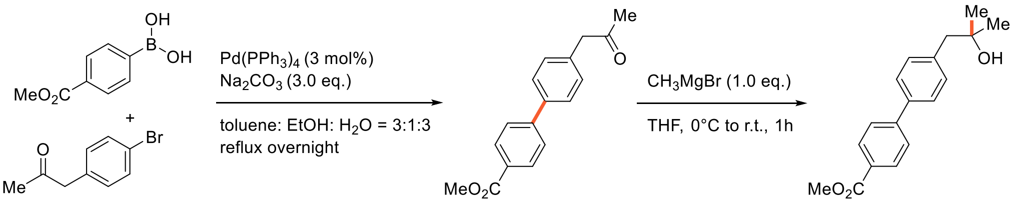

The first step was performed according to the literature [3]; the physical and spectroscopic properties of the target product were in accordance with the literature.

The second step: in a round-bottom flask charged with a magnetic stir bar, the corresponding ketone (1.0 equiv.) was dissolved in THF (0.5 M). The solution was then cooled to 0 °C, and CH_3MgBr (1.0 equiv.) was added dropwise. The mixture was warmed to room temperature, stirred for 1h, quenched using saturated aqueous NH_4Cl, extracted with ethyl acetate, washed with brine and concentrated under reduced pressure. The crude reaction mixture was purified by chromatography on silica gel eluting with a petroleum ether/ethyl acetate gradient to afford the desired product.

Subtrtate **16a** was synthesized while CH_3MgBr (2.0 equiv.) was added (using **14a** as starting material).

Procedure E for substrates **18a, 21a-22a**, **29a-30a**, **32a-33a, 77a**:

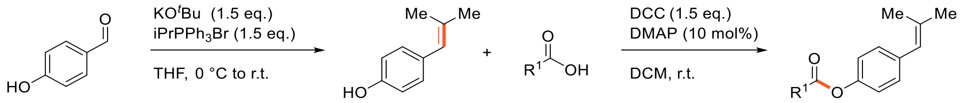

The first step was performed according to general procedure B.

The second step was performed according to general procedure A.

Procedure F for substrates **17a**, **31a, 34a**:

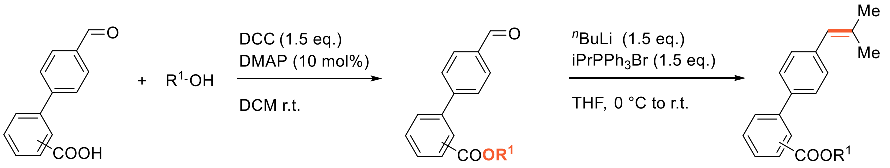

The first step was performed according to general procedure A.

The second step was performed according to general procedure B.

Procedure G for substrates **56a**:

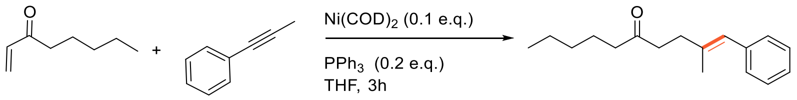

Substrates **56a** was synthetized according to the literature [16]; the physical and spectroscopic properties of the target product were in accordance with the literature.

Procedure H for substrates **57a**:

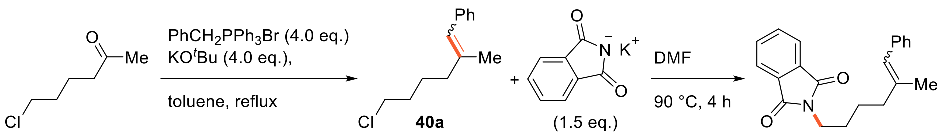

The first step was performed according to general procedure C.
In the second step: a solution of **52a** (1.0 equiv.) in DMF (0.25M) was added phthalimide potassium salt (1.5 equiv.). The reaction mixture was stirred at 90°C and monitored by TLC. Upon completion, the reaction was diluted with diethyl ether, filtered and concentrated. The crude mixture was purified by chromatography on silica gel eluting with a petroleum ether/ethyl acetate gradient to afford the desired product **57a**.

Procedure I for substrates **58a**:

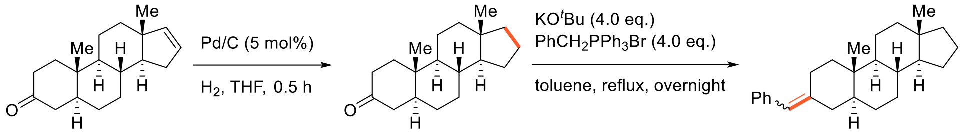

The first step: in a round-bottom flask with a magnetic stir bar, a solution of starting material and 5% Pd/C (5 mol%) were dissolved in THF (0.25 M). The reaction was stirred overnight with a hydrogen balloon. The catalyst was removed by filtration through Celite, and the filtrate was concentrated. The crude residue was used directly without further purification.
The second step was performed according to general procedure C.

Procedure J for substrates **59a, 19a-20a**:

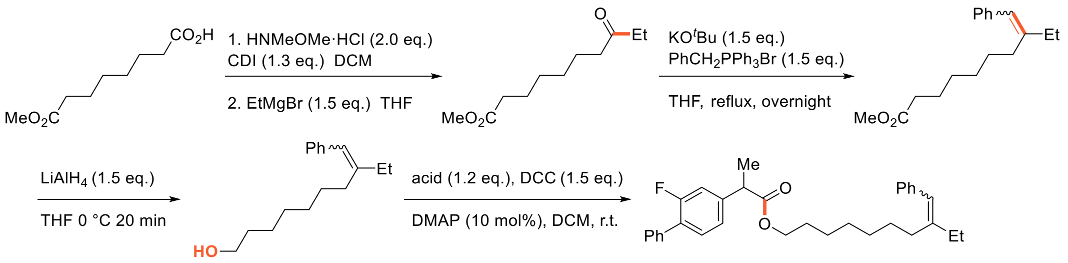

The first step: in a round-bottom flask charged with a magnetic stir bar, a carboxylic acid (1.0 equiv.) was dissolved in DCM (0.4M), CDI (1.3 equiv.) was added portionwise, and the reaction was stirred for 1 hour at room temperature. N, O-Dimethylhydroxylamine hydrochloride(2.0 equiv.) was added, and the reaction was stirred under Ar overnight. The crude mixture was quenched using aqueous HCl (2 M, 20 mL), washed with saturated aqueous $NaHCO_3$, dried over anhydrous Na_2SO4 and concentrated without further purification. Then the crude product was dissolved in THF (0.2 M) and cooled to 0°C. Grignard reagent (1.5 equiv.) was added dropwise at the same temperature. After stirring overnight, the reaction was quenched with aqueous NH_4Cl, extracted with ethyl acetate, concentrated and purified by chromatography on silica gel eluting with a petroleum ether/ethyl acetate gradient to afford the desired product.
The second step was performed according to general procedure B.
The third step: the product from step 2 was dissolved in THF (0.25M) and cooled to 0°C. The suspension of $LiAlH_4$ (1.5 equiv.) in THF was added. The reaction was stirred for 20 minutes and then mixed with water, followed by 2M NaOH. The reaction mixture was filtered through Celite.

The filtrate was extracted with brine, dried over anhydrous Na_2SO_4, filtered, and concentrated in vacuo to obtain alcohol.
The fourth step was performed according to general procedure A.

Subtrtate **19a-20a** were synthesized according to step 1-2 using MeMgBr and PhMgBr.

Procedure K for substrates **61a**:

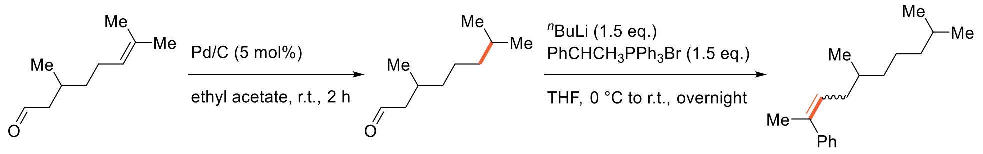

The first step: in a round-bottom flask charged with a magnetic stir bar, a solution of starting material and 5% Pd/C (5 mol%) was dissolved in ethyl acetate (0.25 M). The reaction was stirred overnight with a hydrogen balloon. The catalyst was removed by filtration through Celite, and the filtrate was concentrated. The crude residue was used directly without further purification.
The second step was performed according to general procedure B.

Procedure L for substrates **65a**:

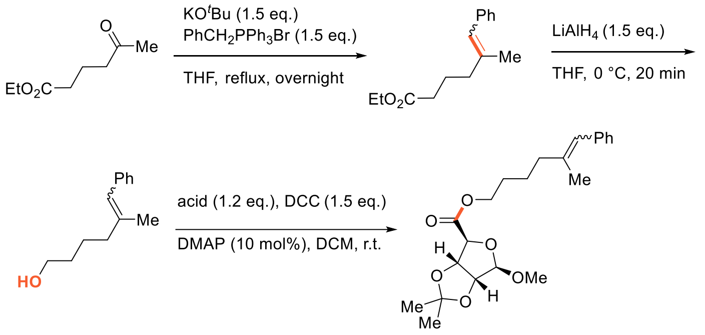

The first step: the starting material was performed according to procedure B, then in a round-bottom flask charged with a magnetic stir bar; it was dissolved in THF (0.25 M) and cooled to 0°C. The suspension of $LiAlH_4$ (1.5 equiv.) in THF was added. The reaction was stirred for 20 minutes and then worked up with water, followed by 2 M NaOH. The reaction mixture was filtered through Celite. The filtrate was extracted with brine, dried over anhydrous Na_2SO_4, filtered, and concentrated in vacuo to obtain alcohol.
The last step was performed according to general procedure A.

Procedure M for substrates **66a**:

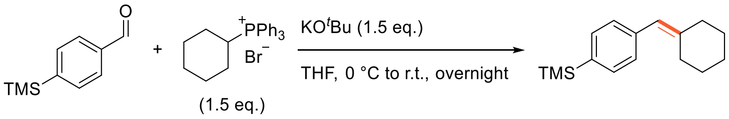

Substrates **66a** was synthesized according to general procedure B.

Procedure N for substrates **68a**:

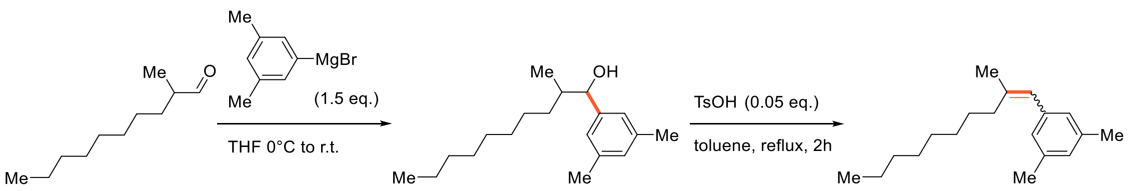

The first step: in a round-bottom flask charged with a magnetic stir bar, the corresponding aldehyde (1.0 equiv.) was dissolved in THF (0.5 M). The solution was then cooled to 0 °C, and Grignard reagent (1.5 equiv.) was added dropwise. The mixture was warmed to room temperature and stirred overnight. Then it was quenched using saturated aqueous NH_4Cl, extracted with ethyl acetate, washed with brine and concentrated under reduced pressure. The crude reaction mixture was purified by chromatography on silica gel eluting with a petroleum ether/ethyl acetate gradient to afford the desired product.
The second step: the target product from step 1 was dissolved in toluene, and the acid (0.05 equiv.) was added. Then the reaction was heated to 130°C for 2h. The reaction was quenched with $NaHCO_3$ and extracted with ethyl acetate. The combined organic layers were washed with brine, dried with anhydrous Na_2SO_4, concentrated and was purified by chromatography on silica gel eluting with a petroleum ether to afford the desired product.

Procedure O for substrates **70a**:

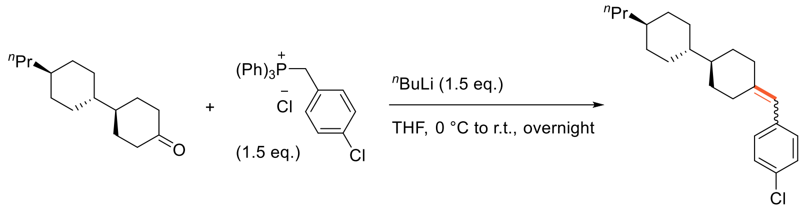

Substrates **70a** was synthesized according to general procedure B.

The physical and spectroscopic properties of substrates **1a**[4], **2a**[2], **6a-7a**[5], **9a**[6], **10a**[7], **24a**[8], **27a**[9], **28a**[10], **35a**[11], **38a**[12], **39a**[4], **42a**[13], **44a**[14], **50a**[15], **55a**[16], **56a**[17], **60a**[18], **63a**[19] were in accordance with the literature. New compounds were characterized as shown below:

1-(tert-butyl)-3-(3-methylbut-2-en-2-yl)benzene **3a**

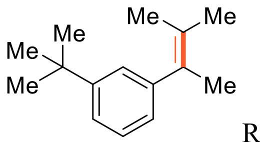

R_f = 0.9, PE/EA = 100:0 (v/v). **^{1}H NMR** (400 MHz, $CDCl_3$) δ 7.30 – 7.24 (m, 2H), 7.22 – 7.18 (m, 1H), 7.02 – 6.96 (m, 1H), 2.01 (s, 3H), 1.85 (s, 3H), 1.64 (s, 3H), 1.36 (s, 9H). **^{13}C NMR** (101 MHz, $CDCl_3$) δ 150.57, 144.86, 130.52, 127.54, 127.02, 125.70, 125.43, 122.57, 34.66, 31.43, 22.15, 20.89, 20.64. **HRMS** (ESI-TOF) calculated for: $C_{15}H_{22}[M+H]^+$: 203.1794, found: 203.1795.

1-(3-methylbut-2-en-2-yl)-4-phenoxybenzene **4a**

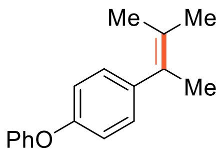

R_f= 0.8, PE/EA = 100:0 (v/v). **^{1}H NMR** (400 MHz, $CDCl_3$) δ 7.37 – 7.31 (m, 2H), 7.13 – 7.07 (m, 3H), 7.06 – 7.00 (m, 2H), 6.98 – 6.92 (m, 2H), 1.96 (s, 3H), 1.81 (s, 3H), 1.62 (s, 3H). **^{13}C NMR** (101 MHz, $CDCl_3$) δ 157.49, 155.04, 140.40, 129.73, 129.71, 129.30, 127.44, 123.04, 118.76, 118.41, 22.17, 20.89, 20.63. **HRMS** (ESI-TOF) calculated for: $C_{17}H_{18}O[M+H]^+$: 239.1430, found: 239.1431.

1-(3-methylbut-2-en-2-yl)-4-pentylbenzene **5a**

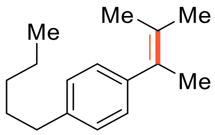

R_f= 0.9, PE/EA = 100:0 (v/v). **^{1}H NMR** (400 MHz, $CDCl_3$) δ 7.14 (d, J = 8.09 Hz, 2H), 7.06 (d, J = 8.11 Hz, 2H), 2.64 – 2.56 (m, 2H), 1.97 (s, 3H), 1.82 (s, 3H), 1.69 – 1.60 (m, 5H), 1.41 – 1.33 (m, 4H), 0.99 – 0.91 (t, J = 7.5 Hz, 3H). **^{13}C NMR** (101 MHz, $CDCl_3$) δ 142.50, 140.27, 129.87, 128.27, 127.89, 126.95, 35.66, 31.67, 31.22, 22.60, 22.16, 20.85, 20.62, 14.09. **HRMS** (ESI-TOF) calculated for: $C_{16}H_{24}[M+H]^+$: 217.1951, found: 217.1952.

methyl 2-(4-(2-methylprop-1-en-1-yl)phenyl)acetate **8a**

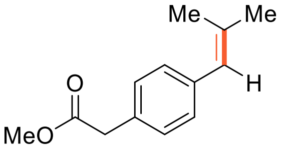

R_f= 0.3, PE/EA =50:1 (v/v). **^{1}H NMR** (400 MHz, $CDCl_3$) δ 7.23 (d, J = 8.26 Hz, 2H), 7.19 (d, J = 8.25 Hz, 2H), 6.24 (s, 1H), 3.70 (s, 3H), 3.61 (s, 2H), 1.90 (s, 3H), 1.86 (s, 3H). **^{13}C NMR** (101 MHz, $CDCl_3$) δ 172.18, 137.58, 135.63, 131.31, 128.93, 124.69, 52.07, 40.90, 26.92, 19.42. **HRMS** (ESI-TOF) calculated for: $C_{13}H_{16}O_2[M+H]^+$: 205.1223, found: 205.1224.

4,4,5,5-tetramethyl-2-(4-(2-methylprop-1-en-1-yl)phenyl)-1,3,2-dioxaborolane **11a**

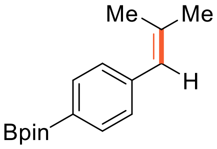

R_f= 0.3, PE/EA = 100:1 (v/v). **^{1}H NMR** (400 MHz, $CDCl_3$) δ 7.76 (d, J = 8.12 Hz, 2H), 7.24 (d, J = 7.86 Hz, 2H), 6.28 (s, 1H), 1.92 (s, 3H), 1.88 (s, 3H), 1.35 (s, 12H). **^{13}C NMR** (101 MHz, $CDCl_3$) δ 141.64, 136.50, 134.56, 128.08, 125.25, 83.68, 27.02, 24.89, 19.52. **HRMS** (ESI-TOF) calculated for: $C_{16}H_{23}BO_2[M+H]^+$: 259.1864, found: 259.1865.

2-fluoro-4-methyl-1-(2-methylprop-1-en-1-yl)benzene **12a**

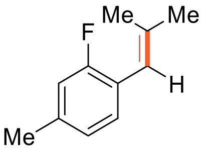

R_f= 0.9, PE/EA = 100:0 (v/v). **^{1}H NMR** (400 MHz, $CDCl_3$) δ 7.15 (t, J = 7.91 Hz, 1H), 6.93 (d, J = 7.84 Hz, 1H), 6.89 (d, J = 11.08 Hz, 1H), 6.25 (s, 1H), 2.37 (s, 3H), 1.97 (s,

3H), 1.83 (s, 3H). **^{13}C NMR** (101 MHz, $CDCl_3$) δ 160.02 (d, J = 245.6 Hz), 138.08 (d, J = 8.0 Hz), 137.26,130.45 (d, J = 4.4 Hz), 124.20 (d, J = 3.1 Hz), 123.21 (d, J = 15.2 Hz), 117.72, 115.83 (d, J = 22.3 Hz), 26.48, 21.00 (d, J = 1.7 Hz), 19.58 (d, J = 1.4 Hz). **^{19}F NMR** (376 MHz, $CDCl_3$) δ -116.44. **HRMS** (ESI-TOF) calculated for $C_{11}H_{13}F$ $[M+H]^+$: 165.1074, found: 165.1075.

tert-butyl (4-(2-methylprop-1-en-1-yl)phenyl) carbonate **13a**

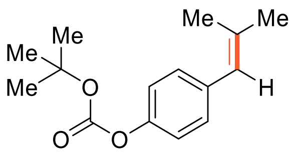

R_f = 0.3, PE/EA = 50:1 (v/v). **^{1}H NMR** (400 MHz, $CDCl_3$) δ 7.21 (d, J = 8.55 Hz, 2H), 7.11 (d, J = 8.67 Hz, 2H), 6.23 (s, 1H), 1.89 (s, 3H), 1.84 (s, 3H), 1.56 (s, 9H). **^{13}C NMR** (101 MHz, $CDCl_3$) δ 152.06, 148.96, 136.35, 135.77, 129.61, 124.22, 120.79, 83.44, 27.72, 26.83, 19.32. **HRMS** (ESI-TOF) calculated for: $C_{15}H_{20}O_3$ $[M+Na]^+$: 271.1305, found: 271.1304.

4'-(2-methylprop-1-en-1-yl)-[1,1'-biphenyl]-4-yl acetate **14a**

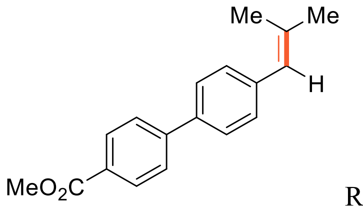

R_f = 0.3, PE/EA = 50:1 (v/v). **^{1}H NMR** (400 MHz, $CDCl_3$) δ 8.11 (d, J = 8.40 Hz, 2H), 7.67 (d, J = 8.43 Hz, 2H), 7.59 (d, J = 8.39 Hz, 2H), 7.33 (d, J = 8.01 Hz, 2H), 6.31 (s, 1H), 3.94 (s, 3H), 1.95 (s, 3H), 1.92 (s, 3H). **^{13}C NMR** (101 MHz, $CDCl_3$) δ 167.06, 145.38, 138.72, 137.17, 136.44, 130.13, 129.30, 128.66, 126.89, 126.77, 124.62, 52.12, 27.08, 19.59. **HRMS** (ESI-TOF) calculated for: $C_{18}H_{18}O_2$$[M+H]^+$: 267.1380, found: 267.1380.

4'-(2-hydroxy-2-methylpropyl)-[1,1'-biphenyl]-4-yl acetate **14a-OH**

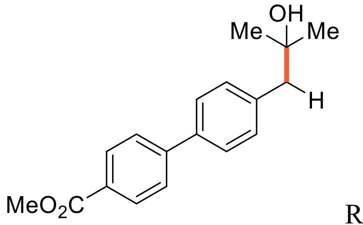

R_f = 0.3, PE/EA = 10:1 (v/v). **^{1}H NMR** (400 MHz, $CDCl_3$) δ 8.09 (d, J = 8.00 Hz, 2H), 7.65 (d, J = 8.03 Hz, 2H), 7.57 (d, J = 7.74 Hz, 2H), 7.31 (d, J = 7.82 Hz, 2H), 3.93 (s, 3H), 2.82 (s, 2H), 1.26 (s, 6H). **^{13}C NMR** (101 MHz, $CDCl_3$) δ 167.04, 145.35, 138.10, 138.05, 131.08, 130.13, 128.77, 127.00, 126.86, 70.86, 52.13, 49.40, 29.28. **HRMS** (ESI-TOF) calculated for: $C_{18}H_{20}O_3$ $[M+H]^+$: 285.1485, found: 285.1486.

4'-(2-methylprop-1-en-1-yl)-[1,1'-biphenyl]-2-carbonitrile **15a**

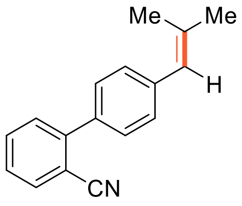

R_f = 0.3, PE/EA = 100:1 (v/v). **^{1}H NMR** (400 MHz, $CDCl_3$) δ 7.76 (dd, J =

1.38, 7.81 Hz, 1H), 7.63 (td, J = 1.43, 7.68 Hz, 1H), 7.55 (d, J = 1.91 Hz, 1H), 7.54 – 7.51 (m, 2H), 7.42 (td, J = 1.33, 7.62 Hz, 1H), 7.36 (d, J = 8.23 Hz, 2H), 6.33 (s, 1H), 1.96 (s, 3H), 1.94 (s, 3H). **^{13}C NMR** (101 MHz, $CDCl_3$) δ 145.35, 139.23, 136.74, 135.42, 133.85, 132.86, 130.03, 129.07, 128.48, 127.38, 124.56, 118.95, 111.07, 27.11, 19.63. **HRMS** (ESI-TOF) calculated for: $C_{17}H_{15}N$ $[M+H]^+$: 234.1277, found: 234.1277.

2-(4'-(2-methylprop-1-en-1-yl)-[1,1'-biphenyl]-4-yl)propan-2-ol **16a**

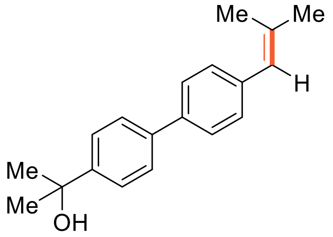

R_f = 0.5, PE/EA = 5:1 (v/v). **^{1}H NMR** (400 MHz, Chloroform-*d*) δ 7.63 – 7.52 (m, 6H), 7.31 (d, J = 8.2 Hz, 2H), 6.31 (t, J = 1.7 Hz, 1H), 1.93 (dd, J = 5.8, 1.4 Hz, 6H), 1.75 (s, 1H), 1.63 (s, 6H). **^{13}C NMR** (101 MHz, Chloroform-*d*) δ 147.97, 139.41, 138.12, 137.73, 135.84, 129.14, 126.77, 126.65, 124.88, 124.74, 72.48, 31.78, 27.05, 19.57. **HRMS** (ESI-TOF) calculated for: $C_{19}H_{22}O$ $[M+Na]^+$: 289.1563, found: 289.1563.

hex-3-yn-1-yl 4'-(2-methylprop-1-en-1-yl)-[1,1'-biphenyl]-4-carboxylate **17a**

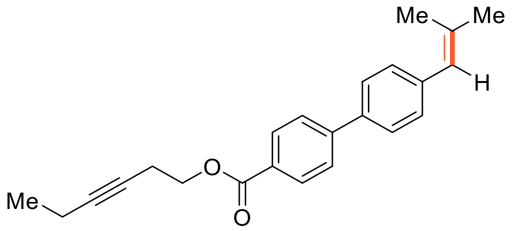

R_f = 0.5, PE/EA = 40:1 (v/v). **^{1}H NMR** (400 MHz, Chloroform-*d*) δ 8.12 (d, J = 8.4 Hz, 2H), 7.71 – 7.64 (m, 2H), 7.59 (d, J = 8.3 Hz, 2H), 7.33 (d, J = 8.3 Hz, 2H), 6.31 (t, J = 1.7 Hz, 1H), 4.41 (t, J = 7.0 Hz, 2H), 2.64 (ddt, J = 7.0, 4.8, 2.4 Hz, 2H), 2.18 (qt, J = 7.5, 2.4 Hz, 2H), 1.93 (dd, J = 8.7, 1.5 Hz, 6H), 1.13 (t, J = 7.5 Hz, 3H). **^{13}C NMR** (101 MHz, Chloroform-*d*) δ 166.30, 145.46, 138.74, 137.21, 136.43, 130.19, 129.28, 128.68, 126.88, 126.76, 124.59, 83.57, 74.87, 63.33, 27.04, 19.57, 19.43, 14.14, 12.41. **HRMS** (ESI-TOF) calculated for: $C_{23}H_{24}O_2$ $[M+H]^+$: 333.1849, found: 333.1848.

4-(2-methylprop-1-en-1-yl)phenyl 4,4-dimethylpent-2-ynoate **18a**

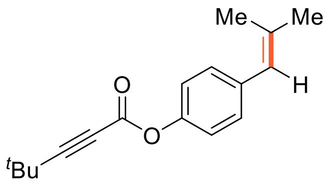

R_f = 0.5, PE/EA = 50:1 (v/v). **^{1}H NMR** (400 MHz, Chloroform-*d*) δ 7.25 – 7.18 (m, 2H), 7.10 – 7.03 (m, 2H), 6.23 (s, 1H), 1.90 (d, J = 1.5 Hz, 3H), 1.85 (d, J = 1.4 Hz, 3H), 1.32 (s, 9H). **^{13}C NMR** (101 MHz, Chloroform-*d*) δ 152.46, 148.07, 136.86, 136.02, 129.71, 124.14, 120.99, 99.15, 71.48, 29.89, 27.72, 26.85, 19.36. **HRMS** (ESI-TOF) calculated for: $C_{17}H_{20}O_2$ $[M+H]^+$: 257.1536, found: 257.1535.

1-(4-(2-methylprop-1-en-1-yl)phenyl)propan-2-one **19a**

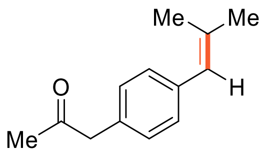

R_f = 0.4, PE/EA = 20:1 (v/v). **^{1}H NMR** (400 MHz, Chloroform-*d*) δ 7.19 (d, *J* = 8.2 Hz, 2H), 7.15 (d, *J* = 8.2 Hz, 2H), 6.27 – 6.20 (m, 1H), 3.67 (s, 2H), 2.15 (s, 3H), 1.88 (dd, *J* = 16.0, 1.5 Hz, 6H). **^{13}C NMR** (101 MHz, Chloroform-*d*) δ 206.60, 137.55, 135.68, 131.61, 129.10, 129.07, 124.64, 50.78, 29.25, 26.92, 19.43. **HRMS** (ESI-TOF) calculated for: $C_{13}H_{16}O$ $[M+H]^+$: 189.1274, found: 189.1273.

2-(4-(2-methylprop-1-en-1-yl)phenyl)-1-phenylethan-1-one **20a**

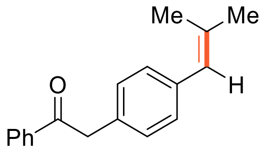

R_f = 0.4, PE/EA = 20:1 (v/v). **^{1}H NMR** (400 MHz, Chloroform-*d*) δ 8.06 – 7.98 (m, 2H), 7.59 – 7.52 (m, 1H), 7.49 – 7.42 (m, 2H), 7.24 – 7.16 (m, 4H), 6.23 (t, *J* = 1.7 Hz, 1H), 4.27 (s, 2H), 1.89 (d, *J* = 1.5 Hz, 3H), 1.85 (d, *J* = 1.4 Hz, 3H). **^{13}C NMR** (101 MHz, Chloroform-*d*) δ 197.74, 137.36, 136.69, 135.52, 133.12, 131.89, 129.13, 129.03, 128.66, 128.64, 124.74, 45.23, 26.91, 19.43. **HRMS** (ESI-TOF) calculated for: $C_{18}H_{18}O$ $[M+H]^+$: 251.1430, found: 251.1430.

4-(2-methylprop-1-en-1-yl)phenyl cinnamate **21a**

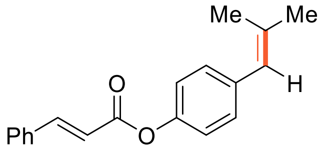

R_f = 0.5, PE/EA = 40:1 (v/v). **^{1}H NMR** (400 MHz, Chloroform-*d*) δ 7.87 (d, *J* = 16.0 Hz, 1H), 7.64 – 7.53 (m, 2H), 7.48 – 7.39 (m, 3H), 7.25 (d, *J* = 8.5 Hz, 2H), 7.15 – 7.07 (m, 2H), 6.64 (d, *J* = 16.0 Hz, 1H), 6.26 (t, *J* = 1.7 Hz, 1H), 1.89 (dd, *J* = 15.7, 1.5 Hz, 6H). **^{13}C NMR** (101 MHz, Chloroform-*d*) δ 165.52, 148.69, 146.49, 136.39, 135.77, 134.24, 130.68, 129.69, 129.01, 128.31, 124.30, 121.13, 117.42, 26.86, 19.38. **HRMS** (ESI-TOF) calculated for: $C_{19}H_{18}O_2$ $[M+H]^+$: 279.1380, found: 279.1378.

4-(2-methylprop-1-en-1-yl)phenyl (E)-octadec-9-enoate **22a**

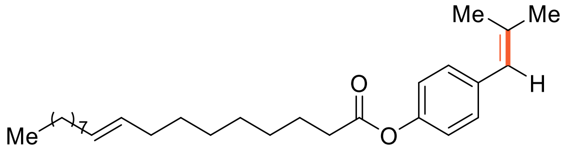

R_f = 0.5, PE/EA = 50:1 (v/v). **^{1}H NMR** (400 MHz, Chloroform-*d*) δ 7.24 – 7.16 (m, 2H), 7.07 – 6.97 (m, 2H), 6.24 (t, *J* = 1.7 Hz, 1H), 5.45 – 5.33 (m, 2H), 2.54 (t, *J* = 7.5 Hz, 2H), 1.97 (ddt, *J* = 8.1, 5.9, 2.9 Hz, 4H), 1.87 (dd, *J* = 19.2, 1.4 Hz, 6H), 1.75 (p, *J* = 7.5 Hz, 2H), 1.46 – 1.20 (m, 20H), 0.92 – 0.83 (m, 3H). **^{13}C NMR** (101 MHz, Chloroform-*d*) δ 172.44, 148.65, 136.30, 135.69, 130.53, 130.21, 129.63, 124.27, 121.07, 34.44, 32.63, 32.57, 31.92, 29.67, 29.57, 29.51, 29.34, 29.21, 29.13, 29.09, 28.96, 26.83, 24.98, 22.70, 19.34, 14.13. **HRMS** (ESI-TOF) calculated for: $C_{28}H_{44}O_2$ $[M+H]^+$: 413.3414, found: 413.3415.

(2-methylhex-2-en-3-yl)benzene **23a**

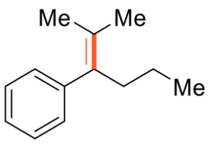

R_f= 0.9, PE/EA = 100:0 (v/v). **^{1}H NMR** (400 MHz, $CDCl_3$) δ 7.34 – 7.27 (m, 2H), 7.24 – 7.17 (m, 1H), 7.15 – 7.04 (m, 2H), 2.41 – 2.25 (m, 2H), 1.82 (s, 3H), 1.55 (s, 3H), 1.34 – 1.23 (m, 2H), 0.86 (t, J = 7.34 Hz, 3H). **^{13}C NMR** (101 MHz, $CDCl_3$) δ 144.28, 135.34, 129.00, 127.82, 127.31, 125.70, 36.43, 22.20, 21.40, 20.19, 13.94. **HRMS** (ESI-TOF) calculated for: $C_{13}H_{18}$ $[M+H]^+$: 175.1481, found: 175.1482.

(5-methylhex-4-ene-1,4-diyl)dibenzene **25a**

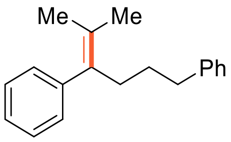

R_f= 0.9, PE/EA = 100:0 (v/v). **^{1}H NMR** (400 MHz, $CDCl_3$) δ 7.41 – 7.35 (m, 2H), 7.35 – 7.27 (m, 3H), 7.26 – 7.22 (m, 1H), 7.22 – 7.15 (m, 4H), 2.65(t, *J* = 7.5 Hz, 2H), 2.50 (t, *J* = 7.77 Hz, 2H), 1.87 (s, 3H), 1.72 – 1.65 (m, 2H), 1.63 (s, 3H). **^{13}C NMR** (101 MHz, $CDCl_3$) δ 144.10, 142.71, 135.14, 129.11, 128.46, 128.31, 128.02, 127.73, 125.93, 125.69, 35.84, 34.17, 30.11, 22.34, 20.30. **HRMS** (ESI-TOF) calculated for: $C_{19}H_{22}$ $[M+H]^+$: 251.1794, found: 251.1796.

4-(3-methyl-2-phenylbut-2-en-1-yl)tetrahydro-2H-pyran **26a**

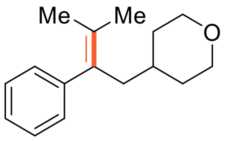

R_f= 0.5, PE/EA = 50:1 (v/v). **^{1}H NMR** (400 MHz, $CDCl_3$) δ 7.34 – 7.28 (m, 2H), 7.24 – 7.19 (m, 1H), 7.12 – 7.03 (m, 2H), 3.89 (dd, *J* = 2.61, 10.98 Hz, 2H), 3.24 (td, *J* = 2.00, 11.59 Hz, 2H), 2.33 (d, *J* = 6.90 Hz, 2H), 1.82 (s, 3H), 1.55 (s, 3H), 1.55 – 1.50 (m, 2H), 1.43 – 1.33 (m, 1H), 1.33 – 1.21 (m, 2H). **^{13}C NMR** (101 MHz, $CDCl_3$) δ 143.99, 133.03, 128.96, 127.97, 125.89, 68.10, 41.38, 33.80, 33.07, 22.33, 20.73. **HRMS** (ESI-TOF) calculated for: $C_{16}H_{22}O$ $[M+H]^+$: 231.1743, found: 231.1744.

4-(2-methylprop-1-en-1-yl)phenyl 4-sulfamoylbenzoate **29a**

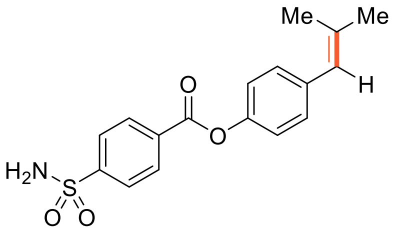

R_f= 0.3, PE/EA = 1:1 (v/v). **^{1}H NMR** (400 MHz, Chloroform-*d*) δ 8.38 – 8.27 (m, 2H), 8.12 – 8.00 (m, 2H), 7.32 – 7.26 (m, 2H), 7.20 – 7.12 (m, 2H), 6.27 (s, 1H), 4.91 (s, 2H), 1.90 (dd, *J* = 16.9, 1.4 Hz, 6H). **^{13}C NMR** (101 MHz, Chloroform-*d*) δ 148.44, 146.25, 136.94, 136.17, 133.56, 129.85, 126.71, 124.12, 120.96, 26.86, 19.39. **HRMS** (ESI-TOF) calculated for: $C_{17}H_{17}NO_4S$ $[M+H]^+$: 332.0951, found: 332.0951.

4-(2-methylprop-1-en-1-yl)phenyl 4-(N,N-dipropylsulfamoyl)benzoate **30a**

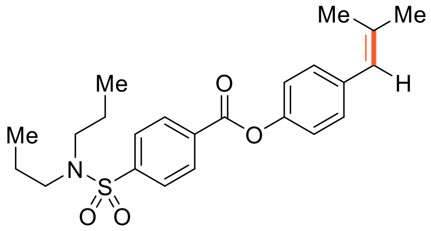

R_f = 0.3, PE/EA = 10:1 (v/v). **^{1}H NMR** (400 MHz, $CDCl_3$) δ 8.32 (d, *J* = 8.47 Hz, 2H), 7.94 (d, *J* = 8.47 Hz, 2H), 7.28 (d, *J* = 8.58 Hz, 2H), 7.16 (d, *J* = 8.57 Hz, 2H), 6.27 (s, 1H), 3.17 – 3.09 (m, 4H), 1.91 (s, 3H), 1.87 (s, 3H), 1.61 – 1.53 (m, 4H), 0.89 (t, *J* = 7.39 Hz, 6H). **^{13}C NMR** (101 MHz, $CDCl_3$) δ 163.99, 148.49, 144.85, 136.87, 136.13, 132.95, 130.80, 129.84, 127.17, 124.14, 120.99, 49.96, 26.88, 21.96, 19.39, 11.19. **HRMS** (ESI-TOF) calculated for: $C_{23}H_{29}NO_4S$ $[M+H]^+$: 416.1890, found: 416.1891.

(1R,2S,4S)-2-isopropyl-4-methylcyclohexyl 4'-(2-methylprop-1-en-1-yl)-[1,1'-biphenyl]-4-carboxylate **31a**

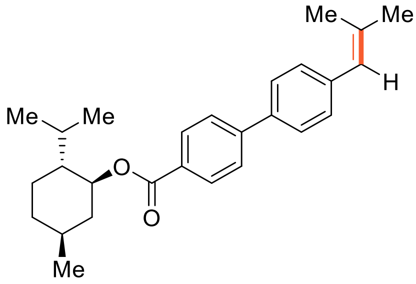

R_f = 0.5, PE/EA = 50:1 (v/v). **^{1}H NMR** (400 MHz, $CDCl_3$) δ 8.12 (d, *J* = 8.41 Hz, 2H), 7.68 (d, *J* = 8.39 Hz, 2H), 7.59 (d, *J* = 8.35 Hz, 2H), 7.34 (d, *J* = 8.31 Hz, 2H), 6.31 (d, *J* = 1.74 Hz, 1H), 4.97 (td, *J* = 4.39, 10.86 Hz, 1H), 2.16 (d, *J* = 13.11 Hz, 1H), 2.06 – 1.97 (m, 1H), 1.95 (s, 3H), 1.93 (s, 3H), 1.75 (d, *J* = 11.27 Hz, 2H), 1.63 – 1.54 (m, 2H), 1.22 – 1.08 (m, 2H), 0.95 (dd, *J* = 2.40, 6.82 Hz, 7H), 0.82 (d, *J* = 6.96 Hz, 3H). **^{13}C NMR** (101 MHz, $CDCl_3$) δ 166.06, 145.24, 138.67, 137.33, 136.40, 130.11, 129.39, 129.29, 126.90, 126.76, 124.62, 47.33, 41.04, 34.38, 31.49, 27.08, 26.55, 23.68, 22.11, 20.83, 19.60, 16.58. **HRMS** (ESI-TOF) calculated for: $C_{27}H_{34}O_2$ $[M+Na]^+$: 413.2451, found: 413.2452.

4-(2-methylprop-1-en-1-yl)phenyl 2-(11-oxo-6,11-dihydrodibenzo[b,e]oxepin-2-yl)acetate **32a**

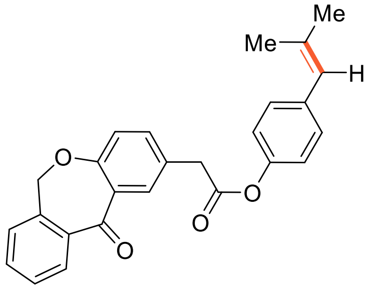

R_f = 0.3, PE/EA = 10:1 (v/v). **^{1}H NMR** (400 MHz, $CDCl_3$) δ 8.23 (d, *J* = 2.44 Hz, 1H), 7.91 (dd, *J* = 1.43, 7.66 Hz, 1H), 7.59 – 7.51 (m, 2H), 7.47 (td, *J* = 1.35, 7.59 Hz, 1H), 7.36 (dd, *J* = 1.32, 7.44 Hz, 1H), 7.20 (d, *J* = 8.59 Hz, 2H), 7.07 (d, *J* = 8.43 Hz, 1H), 7.05 – 7.00 (m, 2H), 6.23 (s, 1H), 5.20 (s, 2H), 3.89 (s, 2H), 1.89 (s, 3H), 1.83 (s, 3H). **^{13}C NMR** (101 MHz, $CDCl_3$) δ 190.83, 169.99, 160.64, 148.58, 140.46, 136.51, 136.34, 135.84, 135.55, 132.84, 132.63, 129.65, 129.53, 129.32, 127.87, 127.32, 125.26, 124.23, 121.26, 120.97, 73.66, 40.33, 26.85, 19.36. **HRMS** (ESI-TOF) calculated for: $C_{26}H_{22}O_4$ $[M+H]^+$: 399.1591, found: 399.1591.

4-(2-methylprop-1-en-1-yl)phenyl (2S,5R)-3,3-dimethyl-7-oxo-4-thia-1-azabicyclo[3.2.0]hep-tane -2-carboxylate 4,4-dioxide **33a**

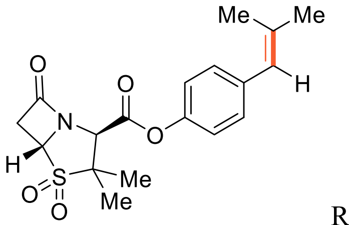

R_f = 0.3, PE/EA = 3:1 (v/v). **^{1}H NMR** (400 MHz, $CDCl_3$) δ 7.24 (d, *J* = 8.61 Hz, 2H), 7.05 (d, *J* = 8.57 Hz, 2H), 6.23 (s, 1H), 4.69 – 4.66 (m, 1H), 4.63 (s, 1H), 3.58 – 3.43 (m, 2H), 1.90 (s, 3H), 1.84 (s, 3H), 1.72 (s, 3H), 1.58 (s, 3H). **^{13}C NMR** (101 MHz, $CDCl_3$) δ 170.79, 165.64, 147.64, 137.42, 136.56, 129.94, 123.88, 120.49, 63.25, 62.89, 61.23, 38.47, 26.85, 20.50, 19.37, 18.79. **HRMS** (ESI-TOF) calculated for: $C_{18}H_{21}NO_5S$ $[M+Na]^+$: 386.1033, found: 386.1032.

((3aS,5aR,8aR,8bS)-2,2,7,7-tetramethyltetrahydro-3aH-bis([1,3]dioxolo)[4,5-b:4',5'-d]pyran-3a-y l)methyl 4'-(2-methylprop-1-en-1-yl)-[1,1'-biphenyl]-2-carboxylate **34a**

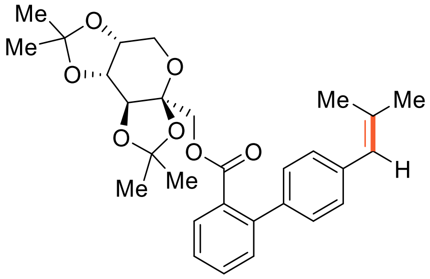

R_f = 0.4, PE/EA = 10:1 (v/v). **^{1}H NMR** (400 MHz, $CDCl_3$) δ 7.80 (d, *J* = 7.67 Hz, 1H), 7.52 (t, *J* = 7.48 Hz, 1H), 7.39 (dd, *J* = 7.32, 14.10 Hz, 2H), 7.29 (s, 4H), 6.29 (s, 1H), 4.36 (t, *J* = 9.18 Hz, 2H), 4.13 (d, *J* = 7.84 Hz, 1H), 3.92 (d, *J* = 11.62 Hz, 1H), 3.82 (d, *J* = 12.96 Hz, 1H), 3.67 (d, *J* = 12.98 Hz, 1H), 3.33 (s, 1H), 1.94 (s, 3H), 1.92 (s, 3H), 1.43 (s, 3H), 1.40 (s, 3H), 1.31 (s, 3H), 1.11 (s, 3H). **^{13}C NMR** (101 MHz, $CDCl_3$) δ 168.79, 141.69, 138.25, 137.68, 135.61, 131.29, 131.01, 130.45, 130.03, 129.06, 128.02, 127.02, 124.92, 108.91, 108.67, 101.36, 70.83, 69.88, 69.80, 65.66, 61.41, 27.14, 26.48, 25.92, 25.29, 24.11, 19.68. **HRMS** (ESI-TOF) calculated for: $C_{29}H_{34}O_7[M+H]^+$: 495.2377, found: 495.2379.

1-methyl-4-(2-methylenebutyl)benzene **36a**

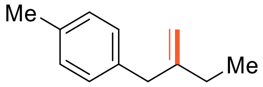

R_f = 0.9, PE/EA = 100:0 (v/v). **^{1}H NMR** (400 MHz, Chloroform-*d*) δ 7.10 (d, *J* = 2.0 Hz, 4H), 4.88 – 4.67 (m, 2H), 3.32 (s, 2H), 2.33 (s, 3H), 1.99 (qd, *J* = 7.5, 1.5 Hz, 2H), 1.03 (td, *J* = 7.4, 1.1 Hz, 3H). **^{13}C NMR** (101 MHz, Chloroform-*d*) δ 150.95, 136.89, 135.45, 128.95, 128.85, 109.58, 42.82, 28.14, 21.04, 12.20. **HRMS** (ESI-TOF) calculated for: $C_{12}H_{16}[M+H]^+$: 161.1325, found: 161.1325.

1-(2,3-dimethylbut-3-en-2-yl)-4-methylbenzene **37a**

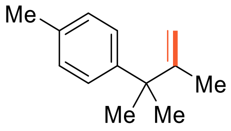

R_f= 0.9, PE/EA = 100:0 (v/v). **^{1}H NMR** (400 MHz, Chloroform-*d*) δ 7.25 – 7.19 (m, 2H), 7.15 – 7.09 (m, 2H), 4.98 (dd, *J* = 1.7, 0.8 Hz, 1H), 4.88 (t, *J* = 1.5 Hz, 1H), 2.63 (q, *J* = 7.6 Hz, 2H), 1.54 (dd, *J* = 1.4, 0.6 Hz, 3H), 1.42 (s, 6H), 1.24 (t, *J* = 7.6 Hz, 3H). **^{13}C NMR** (101 MHz, Chloroform-*d*) δ 152.90, 145.57, 141.39, 127.50, 125.99, 109.33, 43.55, 28.45, 28.30, 20.21, 15.46. **HRMS** (ESI-TOF) calculated for $C_{13}H_{18}$ [M+H]$^{+}$: 175.1481, found: 175.1482.

benzylidenecyclopentadecane **40a**

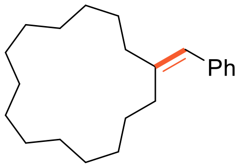

R_f= 0.9, PE/EA = 100:0 (v/v). **^{1}H NMR** (400 MHz, $CDCl_3$) δ 7.34 – 7.28 (m, 2H), 7.24 – 7.15 (m, 3H), 6.28 (s, 1H), 2.27 – 2.22 (m, 2H), 2.20 – 2.14 (m, 2H), 1.54 – 1.31 (m, 24H). **^{13}C NMR** (101 MHz, $CDCl_3$) δ 144.42, 138.73, 128.59, 128.07, 125.77, 125.18, 37.58, 30.42, 27.89, 27.65, 27.38, 26.75, 26.67, 26.64, 26.60, 26.58, 26.52, 26.46, 26.41. **HRMS** (ESI-TOF) calculated for $C_{22}H_{34}$ [M+H]$^{+}$: 299.2733, found: 299.2736.

3-benzylidenespiro[5.5]undecane **41a**

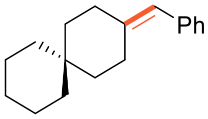

R_f= 0.9, PE/EA = 100:0 (v/v). **^{1}H NMR** (400 MHz, $CDCl_3$) δ 7.34 – 7.28 (m, 2H), 7.24 – 7.16 (m, 3H), 6.24 (s, 1H), 2.43 – 2.36 (m, 2H), 2.30 – 2.25 (m, 2H), 1.54 – 1.38 (m, 14H). **^{13}C NMR** (101 MHz, $CDCl_3$) δ 143.82, 138.42, 128.97, 128.02, 125.77, 121.73, 36.54, 32.66, 32.56, 27.02, 24.50, 21.82. **HRMS** (ESI-TOF) calculated for $C_{18}H_{24}$ [M+H]$^{+}$: 241.1951, found: 241.1951.

4-benzylidene-1-phenylcyclohexane-1-carbonitrile **43a**

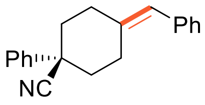

R_f= 0.5, PE/EA = 50:1 (v/v). **^{1}H NMR** (400 MHz, $CDCl_3$) δ 7.55 – 7.50 (m, 2H), 7.45 – 7.39 (m, 2H), 7.39 – 7.31 (m, 3H), 7.29 – 7.23 (m, 3H), 6.42 (d, *J* = 2.02 Hz, 1H), 3.08 (dt, *J* = 4.37, 14.51 Hz, 1H), 2.92 – 2.80 (m, 1H), 2.61 – 2.48 (m, 2H), 2.38 – 2.28 (m, 1H), 2.27 – 2.18 (m, 1H), 2.02 (td, *J* = 4.03, 13.38 Hz, 1H), 1.87 (td, *J* = 3.98, 13.38 Hz, 1H). **^{13}C NMR** (101 MHz, $CDCl_3$) δ 140.41, 138.29, 137.42, 129.02, 128.94, 128.27, 128.07, 126.48, 125.63, 124.53, 122.28, 44.56, 38.42, 37.86, 34.00, 26.23. **HRMS** (ESI-TOF) calculated for $C_{20}H_{19}N$ [M+H]$^{+}$: 274.1590, found: 274.1592.

4-benzylidenetetrahydro-2H-pyran **45a**

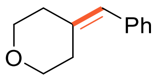

R_f= 0.4, PE/EA = 100:1 (v/v). **^{1}H NMR** (400 MHz, $CDCl_3$) δ 7.37 – 7.29 (m, 2H),

7.26 – 7.16 (m, 3H), 6.34 (s, 1H), 3.83 – 3.77 (t, *J* = 5.55 Hz, 2H), 3.67 (t, *J* = 5.55 Hz, 2H), 2.58 – 2.52 (td, *J* = 5.60, 1.3 Hz, 2H), 2.44 – 2.38 (td, *J* = 5.60, 1.3 Hz, 2H). **^{13}C NMR** (101 MHz, $CDCl_3$) δ 137.71, 137.45, 128.89, 128.20, 126.29, 123.89, 69.51, 68.61, 37.25, 30.67. **HRMS** (ESI-TOF) calculated for $C_{12}H_{14}O$ $[M+H]^+$: 175.1117, found: 175.1119.

((4,4-difluorocyclohexylidene)methyl)benzene **46a**

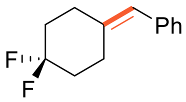

R_f = 0.9, PE/EA = 100:0 (v/v). **^{1}H NMR** (400 MHz, $CDCl_3$) δ 7.41 – 7.30 (m, 2H), 7.26 – 7.12 (m, 3H), 6.40 (s, 1H), 2.56 (td, *J* = 1.24, 6.50 Hz, 2H), 2.47 (td, *J* = 1.27, 6.47 Hz, 2H), 2.12 – 2.00 (m, 2H), 2.01 – 1.88 (m, 2H). **^{13}C NMR** (101 MHz, $CDCl_3$) δ137.62, 137.46, 128.83, 128.25, 126.53, 125.17, 123.32 (t, J = 240.84 Hz), 34.97 (t, J = 23.49 Hz), 34.30 (t, J = 23.77 Hz), 32.70 (t, J = 5.28 Hz), 24.55 (t, J = 5.32 Hz). **^{19}F NMR** (376 MHz, $CDCl_3$) δ -97.40 (p, *J* = 13.7 Hz). **HRMS** (ESI-TOF) calculated for $C_{13}H_{14}F_2$ $[M+H]^+$: 209.1136, found: 209.1137.

3-(2-benzylidenecyclohexyl)propanenitrile **47a**

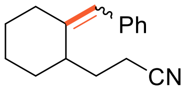

R_f = 0.3, PE/EA = 100:1 (v/v). (*Note*: As determined by NMR spectroscopy, olefin 36a was isolated as a mixture of E/Z isomers (*E:Z*, 1:0.1). The protons of alkyl group and benzene ring could not be assigned to the respective olefinic isomer due to overlap in the spectrum. Therefore, the multiple analysis of the two isomers were integrated together.) **^{1}H NMR** (400 MHz, $CDCl_3$) δ 7.36 – 7.29 (m, 2H), 7.24 – 7.12 (m, 3H), 6.29 (s, 1H), 2.54 – 2.46 (m, 1H), 2.44 – 2.29 (m, 3H), 2.23 – 2.04 (m, 2H), 1.87 – 1.70 (m, 2H), 1.70 – 1.62 (m, 2H), 1.62 – 1.55 (m, 2H), 1.53 – 1.39 (m, 1H). **^{13}C NMR** (101 MHz, $CDCl_3$) δ 142.97, 137.67, 128.98, 128.65, 128.34, 128.14, 126.39, 126.28, 123.53, 120.01, 43.86, 33.16, 32.78, 28.60, 27.91, 27.70, 27.57, 26.52, 22.70, 15.51. **HRMS** (ESI-TOF) calculated for $C_{16}H_{19}N$ $[M+H]^+$: 226.1590, found: 226.1588.

(1R,3R,5S,7R)-4-((E)-benzylidene)adamantan-1-ol **48a**

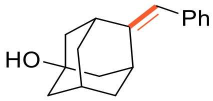

R_f = 0.4, PE/EA = 10:1 (v/v). **^{1}H NMR** (400 MHz, Chloroform-*d*) δ 7.35 – 7.28 (m, 2H), 7.19 (td, *J* = 5.8, 5.3, 2.6 Hz, 3H), 6.22 (s, 1H), 3.31 (dt, *J* = 3.2, 1.7 Hz, 1H), 2.69 (td, *J* = 3.2, 1.6 Hz, 1H), 2.35 – 2.23 (m, 1H), 1.92 – 1.69 (m, 10H).**^{13}C NMR** (101 MHz, Chloroform-*d*) δ 148.27, 138.04, 128.69, 128.14, 126.01, 118.66, 68.33, 46.63, 45.84, 44.86, 42.30, 38.50, 37.63, 33.58, 30.87. **HRMS** (ESI-TOF) calculated for $C_{17}H_{20}O$ $[M+Na]^+$: 263.1406, found: 263.1407.

5-benzylideneoctahydro-1H-4,7-methanoindene **49a**

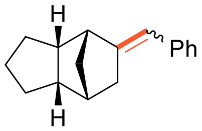

R_f = 0.9, PE/EA = 100:0 (v/v). (*Note*: As determined by NMR spectroscopy, olefin 37a was isolated as a mixture of E/Z isomers (*E:Z*, 1:0.8). The protons of alkyl group and benzene ring could not be assigned to the respective olefinic isomer due to overlap in the spectrum. Therefore,

the multiple analysis of the two isomers were integrated together.) **^{1}H NMR** (400 MHz, $CDCl_3$) ^{1}H NMR (400 MHz, $CDCl_3$) δ 7.38 – 7.33 (m, 2H), 7.33 – 7.29 (m, 2H), 7.24 – 7.12 (m, 1H), 6.35, 6.14 (s, 1H), 3.05,2.60 (s, 1H), 2.43-2.40 (m, 1H), 2.29 – 2.13 (m, 2H), 2.10 – 1.88 (m, 4H), 1.80 – 1.67 (m, 1H), 1.54 (m, 1H), 1.36 – 1.15 (m, 2H), 1.15 – 0.96 (m, 2H). **^{13}C NMR** (101 MHz, $CDCl_3$) δ 149.44, 148.84, 139.00, 138.97, 128.32, 128.25, 127.91, 127.65, 125.57, 118.83, 118.18, 51.94, 47.63, 47.52, 47.46, 46.69, 45.58, 41.62, 40.30, 39.44, 38.03, 33.43, 32.73, 32.45, 31.82, 31.73, 27.81, 27.68. **HRMS** (ESI-TOF) calculated for $C_{17}H_{20}$ $[M+H]^+$: 225.1638, found: 225.1639.

1-(3-methyl-4-phenylbut-3-en-1-yl)-4-(trifluoromethyl)benzene **51a**

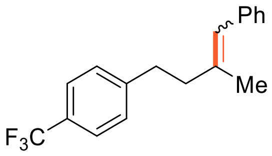

R_f= 0.9, PE/EA = 100:0 (v/v). (*Note*: As determined by NMR spectroscopy, olefin 39a was isolated as a mixture of E/Z isomers (*E:Z*, 1:0.4). *(E)*-Isomer Characterization Data: **^{1}H NMR** (400 MHz, $CDCl_3$) δ 7.57 (d, J = 8.07 Hz, 2H), 7.35 (d, J = 6.11 Hz, 2H), 7.34 – 7.31 (m, 2H), 7.25-7.23 (m, 2H), 7.22-7.20 (m, 1H), 6.27 (s, 1H), 2.95 – 2.88 (t, J = 7.91 Hz, 2H), 2.53 – 2.47 (t, J = 6.79 Hz, 2H), 1.93 (s, 3H). *(Z)*-Isomer Characterization Data: **^{1}H NMR** (400 MHz, $CDCl_3$) δ 7.51 (d, J = 8.00 Hz, 2H), 7.29 (d, J = 7.47 Hz, 2H), 7.25 (m, 2H), 7.21 (m, 1H), 7.13 – 7.08 (m, 2H), 6.36 (s, 1H), 2.88 – 2.83 (m, 2H), 2.59 – 2.54 (m, 2H), 1.96 (s, 3H). **^{13}C NMR** (101 MHz, $CDCl_3$) δ 146.17 (d, J = 1.5 Hz), 145.96, 138.25, 138.21, 137.68, 137.55, 128.80, 128.65, 128.62 (d, J = 16.1 Hz), 128.46, 128.15, 128.10, 127.12 (q, J = 271.7 Hz), 126.83, 126.14, 126.09, 125.92, 125.27 (q, J = 3.6 Hz), 42.18, 34.48, 34.06, 34.00, 23.98, 17.92. **^{19}F NMR** (376 MHz, $CDCl_3$) δ -62.22, -66.26. **HRMS** (ESI-TOF) calculated for $C_{18}H_{17}F_3$ $[M+Na]^+$: 313.1175, found: 313.1174.

(6-chloro-2-methylhex-1-en-1-yl)benzene **52a**

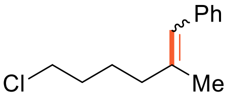

R_f= 0.9, PE/EA = 100:0 (v/v). (*Note*: As determined by NMR spectroscopy, olefin 40a was isolated as a mixture of E/Z isomers (*E:Z*, 1:0.6)). *(E)*-Isomer Characterization Data: **^{1}H NMR** (400 MHz, $CDCl_3$) δ 7.36 – 7.30 (m, 2H), 7.26 – 7.21 (m, 2H), 7.20 – 7.17 (m, 1H), 6.32 (s, 1H), 3.62 (t, J = 6.6 Hz, 2H), 2.26 – 2.22 (m, 2H), 1.90 (d, J = 1.4 Hz, 3H), 1.89 – 1.82 (m, 2H), 1.74 – 1.69 (m, 2H). *(Z)*-Isomer Characterization Data: **^{1}H NMR** (400 MHz, $CDCl_3$) δ 7.35 – 7.33 (m, 2H), 7.26 – 7.23 (m, 2H), 7.23 – 7.21 (m, 1H), 6.35 (s, 1H), 3.53 (t, J = 6.5 Hz, 2H), 2.26 – 2.22 (m, 2H), 1.92 (d, J = 1.5 Hz, 3H), 1.85 – 1.80 (m, 2H), 1.68 – 1.64 (m, 2H). **^{13}C NMR** (101 MHz, $CDCl_3$) δ 138.43, 138.37, 128.84, 128.71, 128.58, 128.16, 128.07, 126.10, 126.02, 125.96, 125.38, 45.08, 44.85, 39.87, 32.33, 32.14, 31.50, 25.15, 25.12, 23.83, 17.65. **HRMS** (ESI-TOF) calculated for $C_{13}H_{17}Cl$ $[M+H]^+$: 209.1092, found: 209.1094.

(2-heptylnon-1-en-1-yl)benzene **54a**

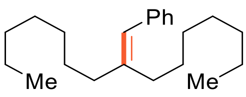

R_f= 0.9, PE/EA = 100:0 (v/v). **^{1}H NMR** (400 MHz, $CDCl_3$) δ 7.36 – 7.28 (m, 2H), 7.24 – 7.15 (m, 3H), 6.27 (s, 1H), 2.26 – 2.11 (m, 4H), 1.50 (td, J = 5.86, 12.99, 15.66 Hz, 4H), 1.38 – 1.23 (m, 16H), 0.90 (dt, J = 6.69, 9.26 Hz, 6H). **^{13}C NMR** (101 MHz, $CDCl_3$) δ 144.04,

138.79, 128.66, 128.03, 125.75, 124.67, 37.30, 31.94, 31.87, 30.65, 29.79, 29.50, 29.31, 29.17, 28.35, 28.26, 22.74, 22.71, 14.17, 14.14. **HRMS** (ESI-TOF) calculated for $C_{22}H_{36}$ $[M+H]^+$: 301.2890, found: 301.2892.

2-(5-methyl-6-phenylhex-5-en-1-yl)isoindoline-1,3-dione **57a**

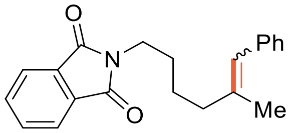

R_f= 0.4, PE/EA = 15:1 (v/v). (*Note*: As determined by NMR spectroscopy, olefin 45a was isolated as a mixture of E/Z isomers (*E:Z*, 1:0.6). The protons of alkyl group and benzene ring could not be assigned to the respective olefinic isomer due to overlap in the spectrum. Therefore, the multiple analysis of the two isomers were integrated together.). **^{1}H NMR** (400 MHz, $CDCl_3$) δ 7.89 – 7.79 (m, 2H), 7.74 – 7.66 (m, 2H), 7.32 – 7.26 (m, 2H), 7.24 – 7.21 (m, 1H), 7.20 – 7.12 (m, 2H), 6.28 (s, 1H), 3.79 – 3.62 (m, 2H), 2.31 – 2.17 (m, 2H), 1.85 (s, 3H), 1.78 – 1.65 (m, 2H), 1.62 – 1.49 (m, 2H). **^{13}C NMR** (101 MHz, $CDCl_3$) δ 168.48, 168.44, 138.81, 138.47, 138.40, 133.90, 132.18, 132.15, 128.84, 128.55, 128.12, 128.01, 125.99, 125.95, 125.86, 125.32, 123.21, 40.16, 37.92, 31.96, 28.57, 28.23, 25.35, 25.19, 23.95, 17.66. **HRMS** (ESI-TOF) calculated for $C_{21}H_{21}NO_2$ $[M+H]^+$:320.1645, found: 320.1646.

(5S,8S,9S,10S,13S,14S)-3-benzylidene-10,13-dimethylhexadecahydro-1H-cyclopenta[a]phena-nthrene **58a**

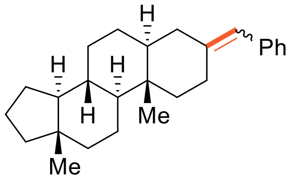

R_f = 0.9, PE/EA = 100:0 (v/v). (*Note*: As determined by NMR spectroscopy, olefin 46a was isolated as a mixture of E/Z isomers (*E:Z*, 1:1). The protons of alkyl group and benzene ring could not be assigned to the respective olefinic isomer due to overlap in the spectrum. Therefore, the multiple analysis of the two isomers were integrated together.).**^{1}H NMR** (400 MHz, $CDCl_3$) δ 7.35 – 7.28 (m, 2H), 7.24 – 7.15 (m, 3H), 6.22 (s, 1H), 2.83 – 2.50 (m, 1H), 2.49 – 2.04 (m, 2H), 2.02 – 1.78 (m, 2H), 1.77 – 1.50 (m, 7H), 1.47 – 1.40 (m, 1H), 1.39 – 1.21 (m, 5H), 1.20 – 1.07 (m, 4H), 1.04 – 0.85 (m, 6H), 0.72 (s, 3H). **^{13}C NMR** (101 MHz, $CDCl_3$) δ 143.29, 143.14, 138.43, 138.34, 129.06, 129.00, 128.04, 128.02, 125.76, 125.74, 121.73, 121.59, 54.73, 54.66, 54.57, 54.54, 50.89, 50.86, 48.24, 48.07, 47.48, 47.34, 46.24, 46.19, 41.83, 40.89, 40.88, 40.55, 40.47, 40.07, 40.03, 39.56, 38.95, 36.47, 36.41, 35.87, 35.85, 33.21, 32.42, 32.39, 32.22, 29.06, 28.92, 25.57, 25.55, 25.26, 21.20, 21.11, 20.55, 17.60, 11.97, 11.93. **HRMS** (ESI-TOF) calculated for: $C_{26}H_{36}$$[M+H]^+$: 349.2890, found: 349.2891.

8-benzylidenedecyl 2-(2-fluoro-[1,1'-biphenyl]-4-yl)propanoate **59a**

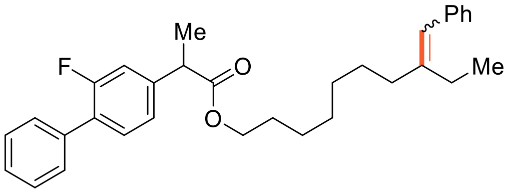

R_f= 0.3, PE/EA = 50:1 (v/v). (*Note*: As determined by

NMR spectroscopy, olefin 47a was isolated as a mixture of E/Z isomers (*E:Z*, 1:0.8). The protons of alkyl group and benzene ring could not be assigned to the respective olefinic isomer due to overlap in the spectrum. Therefore, the multiple analysis of the two isomers were integrated together.). **^{1}H NMR** (400 MHz, $CDCl_3$) δ 7.57 – 7.52 (m, 2H), 7.47 – 7.42 (m, 2H), 7.42 – 7.35 (m, 2H), 7.35 – 7.28 (m, 2H), 7.24 – 7.20 (m, 2H), 7.20 – 7.16 (m, 2H), 7.16 – 7.11 (m, 1H), 6.25 (s, 1H), 4.17 – 4.03 (m, 2H), 3.81 – 3.70 (m, 1H), 2.28 – 2.12 (m, 4H), 1.67 – 1.57 (m, 2H), 1.55 (dd, *J* = 2.33, 7.18 Hz, 3H), 1.52 – 1.39 (m, 2H), 1.34 – 1.24 (m, 6H), 1.14 – 1.03 (m, 3H). **^{13}C NMR** (101 MHz, $CDCl_3$) δ 174.08, 159.69 (d, J = 248.25 Hz), 145.18, 145.03, 142.01 (d, J = 7.69 Hz), 138.77, 138.63, 135.54, 130.76 (d, J = 4.03 Hz), 128.97 (d, J = 2.93 Hz), 128.64, 128.59, 128.47, 128.08, 128.06, 127.82, 127.67, 125.85, 125.81, 124.36, 123.73, 123.57 (d, J = 3.28 Hz), 115.27 (d, J = 23.56 Hz), 65.13, 45.13, 36.69, 30.68, 29.91, 29.59, 29.36, 29.15, 29.00, 28.57, 28.53, 28.20, 28.05, 25.85, 25.80, 23.62, 18.38, 13.10, 12.87. **^{19}F NMR** (376 MHz, $CDCl_3$) δ -117.72. **HRMS** (ESI-TOF) calculated for $C_{32}H_{37}FO_2$ $[M+H]^+$: 473.2850, found: 473.2851.

(5,9-dimethyldec-2-en-2-yl)benzene **61a**

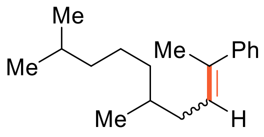

R_f = 0.9, PE/EA = 100:0 (v/v). (*Note*: As determined by NMR spectroscopy, olefin 61a was isolated as a mixture of E/Z isomers (*E:Z*, 1:0.3).*(E)*-Isomer Characterization Data: **^{1}H NMR** (400 MHz, $CDCl_3$) δ 7.43 – 7.38 (m, 2H), 7.36 – 7.28 (m, 3H), 5.81 (td, *J* = 1.42, 7.37 Hz, 1H), 2.28 – 2.15 (m, 1H), 2.04 (s, 3H), 1.58 – 1.44 (m, 2H), 1.42 – 1.23 (m, 4H), 1.21 – 1.09 (m, 4H), 0.93 (d, *J* = 6.68 Hz, 3H), 0.89 (d, *J* = 0.94 Hz, 3H), 0.88 (d, *J* = 0.90 Hz, 3H). *(Z)*-Isomer Characterization Data: **^{1}H NMR** (400 MHz, $CDCl_3$) δ 7.24 – 7.22 (m, 2H), 7.22 – 7.20 (m, 1H), 7.20 – 7.16 (m, 2H), 5.48 (td, *J* = 1.55, 7.37 Hz, 1H), 2.05 (s, 3H), 2.02 – 1.93 (m, 1H), 1.85 – 1.76 (m, 1H), 1.64 – 1.59 (m, 2H), 1.53 – 1.45 (m, 2H), 1.27 – 1.22 (m, 2H), 1.09 – 0.97 (m, 2H), 0.86 (s, 3H), 0.84 (s, 3H), 0.83 (d, *J* = 6.70 Hz, 3H). **^{13}C NMR** (101 MHz, $CDCl_3$) δ 144.25, 142.48, 136.65, 135.09, 128.15, 128.08, 127.98, 127.68, 126.64, 126.42, 126.29, 125.65, 39.33, 39.27, 37.09, 36.89, 36.19, 33.78, 28.02, 25.83, 24.98, 24.84, 22.75, 22.73, 22.65, 22.63, 19.81, 19.70, 15.98. **HRMS** (ESI-TOF) calculated for: $C_{18}H_{28}$ $[M+H]^+$: 245.2264, found: 245.2264.

methyl (1r,4r)-4-(2-phenylprop-1-en-1-yl)cyclohexane-1-carboxylate **62a**

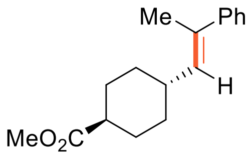

R_f = 0.3, PE/EA =100:1 (v/v). (*Note*: As determined by NMR spectroscopy, olefin 50a was isolated as a mixture of E/Z isomers (*E:Z*, 1:0.3). *(E)*-Isomer Characterization Data: **^{1}H NMR** (400 MHz, $CDCl_3$) δ 7.31 – 7.27 (m, 2H), 7.24 – 7.21 (m, 2H), 7.20 – 7.17 (m, 1H), 5.48 (dd, *J* = 1.41, 8.99 Hz, 1H), 3.60 (s, 3H), 2.31 – 2.16 (m, 2H), 1.96 (d, *J* = 1.39 Hz, 3H), 1.85 – 1.78 (m, 2H), 1.75 – 1.65 (m, 1H), 1.50 – 1.39 (m, 2H), 1.16 – 0.98 (m, 3H). *(Z)*-Isomer Characterization Data: **^{1}H NMR** (400 MHz, $CDCl_3$) δ 7.16 – 7.14 (m, 1H), 7.14 – 7.10 (m, 2H), 7.09 – 7.05 (m, 2H), 5.13 (dd, J = 1.52, 9.90 Hz, 1H) 3.54 (s, 3H), 2.12 (m, 2H), 1.91 (d, *J* = 1.50 Hz, 3H), 1.81 (m, 2H), 1.61 (m, 1H), 1.60 – 1.55 (m, 2H), 1.23 (m, 3H). **^{13}C NMR** (101 MHz, $CDCl_3$) δ 176.61, 143.79, 142.37, 135.37, 133.74, 133.48, 132.94, 128.17, 128.14, 127.71, 126.61, 126.52, 125.64, 51.58,

51.50, 42.84, 42.80, 37.07, 36.84, 32.49, 32.03, 28.68, 28.37, 25.84, 15.93. **HRMS** (ESI-TOF) calculated for: $C_{17}H_{22}O_2$ [M+H]$^+$: 259.1693, found: 259.1693.

(1-(cyclohex-3-en-1-yl)prop-1-en-2-yl)benzene **64a**

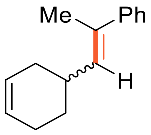

R$_f$= 0.9, PE/EA = 100:0 (v/v). (*Note*: As determined by NMR spectroscopy, olefin 52a was isolated as a mixture of E/Z isomers (*E:Z*,1:0.3). The protons of alkyl group and benzene ring fcould not be assigned to the respective olefinic isomer due to overlap in the spectrum. Therefore, the multiple analysis of the two isomers were integrated together.).**^{1}H NMR** (400 MHz, $CDCl_3$) δ 7.43 – 7.33 (m, 2H), 7.33 – 7.24 (m, 2H), 7.24 – 7.17 (m, 1H), 5.77 – 5.70 (m, 2H), 5.70 – 5.33 (m, 1H), 2.74 – 2.26 (m, 1H), 2.25 – 2.15 (m, 1H), 2.15 – 2.09 (m, 2H), 2.06 (dd, J = 20.7, 1.4 Hz, 3H), 1.94 – 1.83 (m, 1H), 1.82 – 1.62 (m, 1H), 1.53 – 1.35 (m, 1H). **^{13}C NMR** (101 MHz, $CDCl_3$) δ 143.93, 142.50, 135.20, 133.69, 133.60, 132.98, 128.19, 128.14, 127.85, 127.04, 126.82, 126.59, 126.46, 126.31, 126.22, 125.70, 33.55, 33.37, 32.06, 31.55, 30.98, 28.82, 25.92, 24.82, 24.55, 15.89. **HRMS** (ESI-TOF) calculated for: $C_{15}H_{18}$ [M+H]$^+$: 199.1481, found: 199.1483.

6-phenylhept-5-en-1-yl(3aS,4S,6R,6aR)-6-methoxy-2,2-dimethyltetrahydrofuro[3,4-d][1,3]dioxole -4-carboxylate **65a**

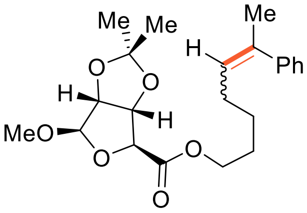

R$_f$ = 0.4, PE/EA = 20:1 (v/v). (*Note*: As determined by NMR spectroscopy, olefin 53a was isolated as a mixture of E/Z isomers (*E:Z*, 1:0.3). *(E)*-Isomer Characterization Data:**^{1}H NMR** (400 MHz, $CDCl_3$) δ 7.39 – 7.35 (m, 2H), 7.34 – 7.30 (m, 2H), 7.29 (d, J = 1.7 Hz, 1H), 5.80 – 5.71 (m, 1H), 5.22 (dd, J = 5.8, 1.1 Hz, 1H), 5.04 (s, 1H), 4.61 (s, 1H), 4.55 (d, J = 5.6 Hz, 1H), 4.24 – 4.15 (m, 2H), 3.41 (s, 3H), 2.30 – 2.20 (m, 2H), 2.0 (s, 3H), 1.80 – 1.70 (m, 2H), 1.57 – 1.52 (m, 2H), 1.50 (s, 3H), 1.34 (s, 3H). *(Z)*-Isomer Characterization Data: **^{1}H NMR** (400 MHz, $CDCl_3$) δ 7.24 – 7.21 (m, 3H), 7.16 (dd, J = 8.1, 1.4 Hz, 2H), 5.43 (td, J = 7.3, 1.6 Hz, 1H), 5.20 (dd, J = 6.0, 1.1 Hz, 1H), 5.03 (s, 1H), 4.60 (s, 1H), 4.54 (s, 1H), 4.12 – 4.02 (m, 2H), 3.37 (s, 3H), 2.05 (s, 3H), 2.02 – 1.98 (m, 2H), 1.64 – 1.59 (m, 1H), 1.45 – 1.38 (m, 2H). **^{13}C NMR** (101 MHz, $CDCl_3$) δ 170.21, 170.16, 143.80, 142.04, 136.88, 135.33, 128.19, 128.10, 127.90, 127.68, 126.91, 126.62, 126.53, 125.61, 112.72, 112.70, 109.36, 109.34, 84.39, 83.75, 83.72, 82.19, 65.40, 55.44, 55.41, 28.55, 28.28, 28.25, 28.09, 26.40, 26.27, 25.84, 25.64, 25.07, 22.65, 15.86. **HRMS** (ESI-TOF) calculated for: $C_{22}H_{30}O_6$ [M+H]$^+$: 391.2115, found: 391.2115.

(4-(cyclohexylidenemethyl)phenyl)trimethylsilane **66a**

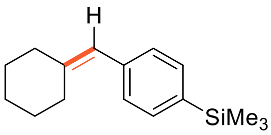

R$_f$= 0.9, PE/EA = 100:0 (v/v). **^{1}H NMR** (400 MHz, $CDCl_3$) δ 7.50 (d, J =

8.1 Hz, 2H), 7.23 (d, *J* = 7.8 Hz, 2H), 6.25 (s, 1H), 2.43 (t, *J* = 6.0 Hz, 2H), 2.34 – 2.25 (t, *J* = 7.3 Hz, 2H), 1.73 – 1.57 (m, 6H), 0.30 (s, 9H). **^{13}C NMR** (101 MHz, $CDCl_3$) δ 143.76, 138.92, 137.52, 133.10, 128.29, 121.95, 37.74, 29.56, 28.65, 27.91, 26.70, -1.04. **HRMS** (ESI-TOF) calculated for: $C_{16}H_{24}Si$ $[M+H]^+$: 245.1720, found: 245.1723.

1,3-dimethyl-5-(2-methyldec-1-en-1-yl)benzene **68a**

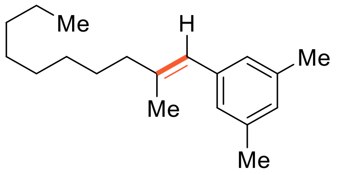

R_f = 0.9, PE/EA = 100:0 (v/v). (*Note*: As determined by NMR spectroscopy, olefin 56a was isolated as a mixture of E/Z isomers (*E:Z*, 1:0.5). The protons of alkyl group and benzene ring could not be assigned to the respective olefinic isomer due to overlap in the spectrum. Therefore, the multiple analysis of the two isomers were integrated together.).**^{1}H NMR** (400 MHz, $CDCl_3$) δ 6.90 (s, 1H), 6.86 (s, 2H), 6.24 (s, 1H), 2.34 (s, 6H), 2.26 – 2.14 (m, 2H), 1.88 (s, 3H), 1.57 – 1.51 (m, 2H), 1.38 – 1.29 (m, 10H), 0.95 – 0.90 (m, 3H). **^{13}C NMR** (101 MHz, $CDCl_3$) δ 139.60, 139.08, 138.69, 138.57, 137.41, 127.51, 127.47, 126.68, 126.44, 125.36, 124.78, 40.82, 32.62, 31.98, 31.94, 29.73, 29.61, 29.49, 29.38, 29.36, 28.22, 28.09, 24.15, 22.75, 22.73, 21.38, 17.84, 14.18. **HRMS** (ESI-TOF) calculated for: $C_{19}H_{30}$ $[M+H]^+$: 259.2420, found: 259.2421.

(1S,1's,4'S)-4-((E)-4-chlorobenzylidene)-4'-propyl-1,1'-bi(cyclohexane) **70a**

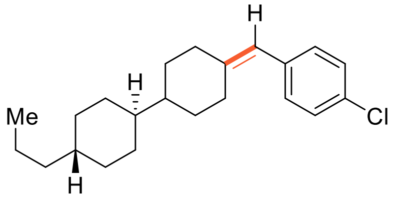

R_f= 0.9, PE/EA = 100:0 (v/v). **^{1}H NMR** (400 MHz, $CDCl_3$) δ 7.27 (d, J = 8.00 Hz, 2H), 7.13 (d, J = 8.08 Hz, 2H), 6.17 (s, 1H), 2.85 (d, J = 13.50 Hz, 1H), 2.37 (d, J = 13.38 Hz, 1H), 2.21 (d, J = 13.36 Hz, 1H), 1.94 – 1.67 (m, 8H), 1.32 (p, J = 6.83 Hz, 4H), 1.21 – 1.12 (m, 4H), 1.09 (d, J = 12.68 Hz, 2H), 1.00 (d, J = 12.12 Hz, 2H), 0.91 – 0.87 (m, 3H). **^{13}C NMR** (101 MHz, $CDCl_3$) δ 144.34, 136.87, 131.49, 130.25, 128.17, 120.67, 43.31, 42.97, 39.87, 37.65, 37.36, 33.62, 31.72, 31.05, 30.23, 30.18, 29.08, 20.12, 14.51. **HRMS** (ESI-TOF) calculated for: $C_{22}H_{31}Cl$ $[M+H]^+$: 331.2187, found: 331.2180.

1-methyl 4-(4-(2-methylprop-1-en-1-yl)phenyl) (2r,3R,4s,5S)-cubane-1,4-dicarboxylate **77a**

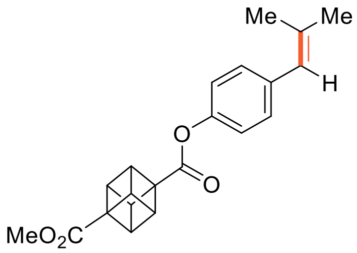

R_f= 0.3, PE/EA = 50:1 (v/v). **^{1}H NMR** (400 MHz, $CDCl_3$) δ 7.21 (d, J = 8.51 Hz, 2H), 7.03 (d, J = 8.58 Hz, 2H), 6.23 (s, 1H), 4.42 – 4.35 (m, 3H), 4.35 – 4.28 (m, 3H), 3.73 (s, 3H), 1.89 (s, 3H), 1.85 (s, 3H). **^{13}C NMR** (101 MHz, $CDCl_3$) δ 171.89, 169.95, 148.41, 136.42, 135.80, 129.67, 124.23, 120.99, 55.92, 55.84, 51.68, 47.28, 47.21, 26.84, 19.36. **HRMS**

(ESI-TOF) calculated for: $C_{21}H_{20}O_4$ [M+Na]$^+$: 359.1254, found: 359.1254.

trideca-2,12-dien-2-ylbenzene **79a**

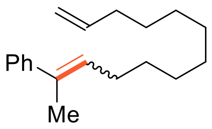

R$_f$ = 0.9, PE/EA = 100:0 (v/v). (*Note*: As determined by NMR spectroscopy, olefin 67a was isolated as a mixture of E/Z isomers (*E:Z*, 1:0.9). The protons of alkyl group and benzene ring could not be assigned to the respective olefinic isomer due to overlap in the spectrum. Therefore, the multiple analysis of the two isomers were integrated together.). **^{1}H NMR** (400 MHz, $CDCl_3$) δ 7.45 – 7.36 (m, 2H), 7.35 – 7.26 (m, 2H), 7.25 – 7.18 (m, 1H), 5.91 – 5.83 (m, 1H), 5.83 – 5.77 (m, 1H), 5.07 – 4.99 (m, 1H), 4.99 – 4.94 (m, 1H), 2.32 – 2.14 (m, 2H), 2.11 – 1.96 (m, 6H), 1.50 – 1.25 (m, 12H). **^{13}C NMR** (101 MHz, $CDCl_3$) δ 144.18, 142.44, 139.25, 136.09, 134.66, 128.85, 128.26, 128.14, 128.10, 126.56, 126.50, 125.74, 114.34, 114.30, 34.03, 30.35, 29.85, 29.77, 29.72, 29.65, 29.62, 29.43, 29.37, 29.34, 29.25, 29.16, 29.14, 29.00, 25.76, 15.91. **HRMS** (ESI-TOF) calculated for: $C_{19}H_{28}$ [M+H]$^+$: 257.2264, found: 257.2264.

3. **Condition screening**

Supplementary Table 1. Condition screening of PCs

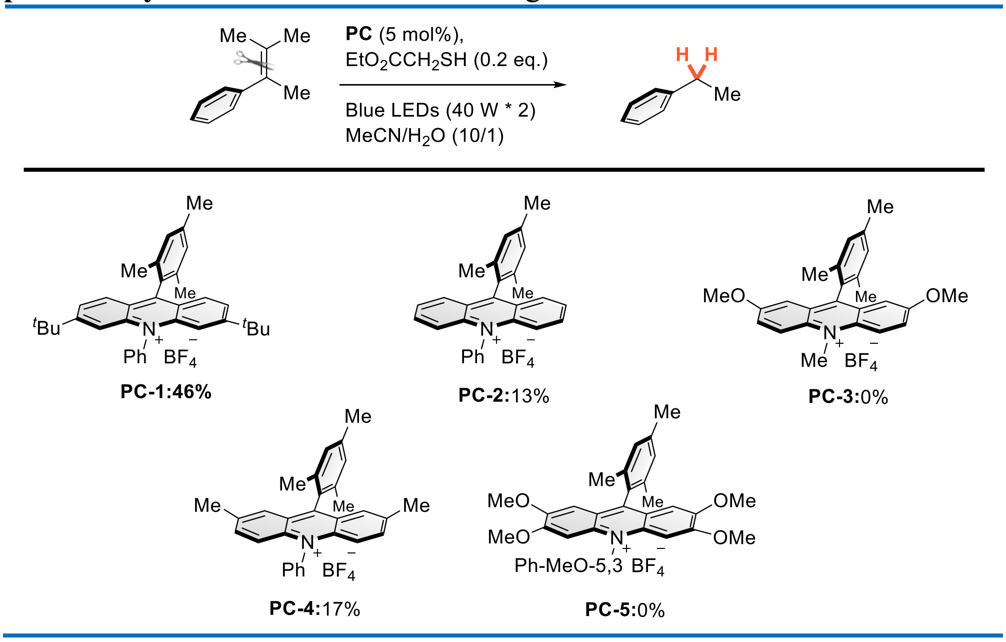

Supplementary Table 2.. Condition screening of HATs

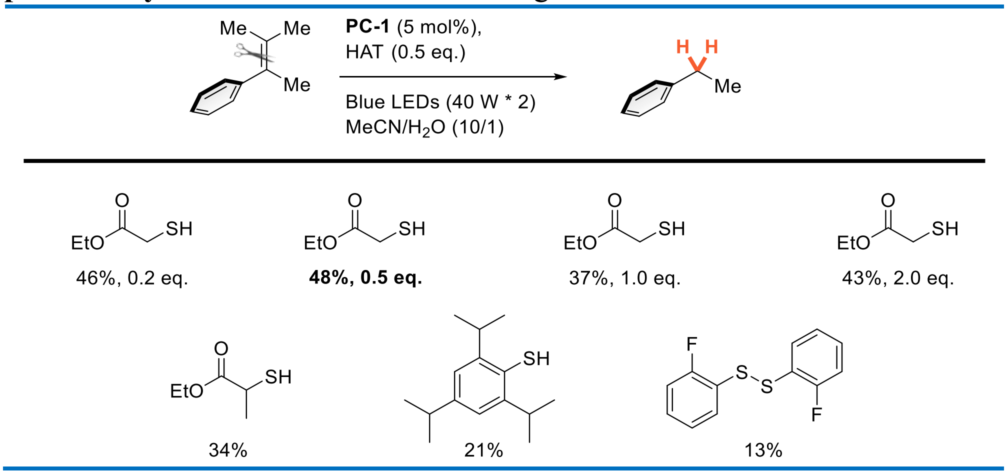

The low boiling point affects the stability of conditional screening, therefore substrate replacement is necessary

Supplementary Table 3. Condition screening of HATs using 1a as substrate

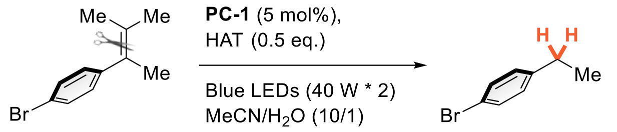

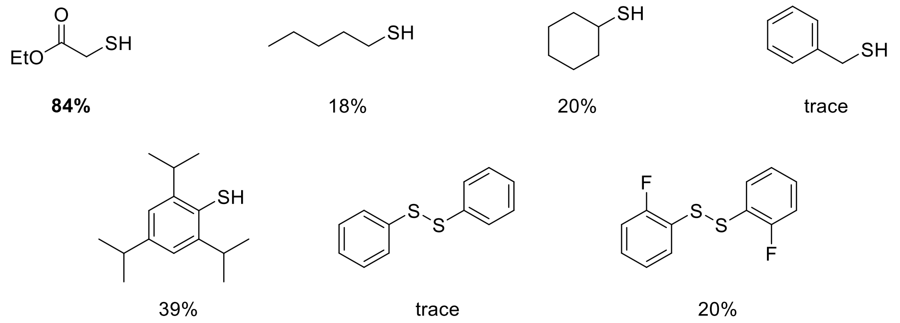

Supplementary Table 4. Condition screening using *p*-Br substituted styrenes [a]

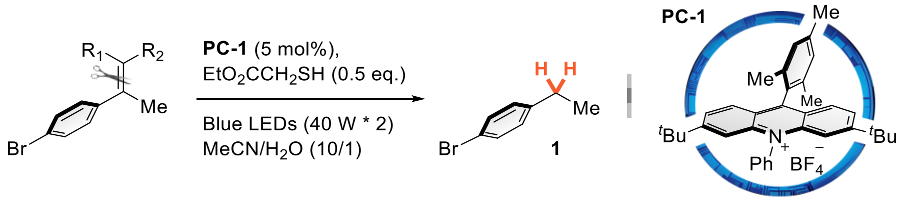

entry	R group	*hv* (nm)	Solvent	yield (%)
1	$R_{1,2}$ = Me	456	$MeCN/H_2O$	60
2[b]	$R_{1,2}$ = Me	456	$MeCN/H_2O$	73
**3[c]**	**$R_{1,2}$ = Me**	**440**	**$MeCN/H_2O$**	**84**
4	$R_{1,2}$ = H	440	$MeCN/H_2O$	0
5	R_1 = H, R_2 = Me	440	$MeCN/H_2O$	74
6	$R_{1,2}$ = Me	440	MeCN	0
7[d]	$R_{1,2}$ = Me	440	$MeCN/H_2O$	0
8[e]	$R_{1,2}$ = Me	440	$MeCN/H_2O$	0
9[f]	$R_{1,2}$ = Me	440	$MeCN/H_2O$	0

[a] A two-dram glass vial containing a mixture of ^{t}Bu-Acr-BF_4 (0.01 mmol, 5 mol%), **1a** (0.2 mmol), EtO_2CCH_2SH (0.1 mmol) in MeCN/water (1.0mL/0.1 mL) was placed between two Kessil LED lights (440 nm, 40 W) and vigorously stirred for 24 h. [b] The distance of the fan was reduced from 10 cm to 5 cm. [c] water bath at 35 °C. [d] EtO_2CCH_2SH was removed. [e] The reaction was run in the dark. [f] Photocatalyst was removed.

The function of benzalmalononitrile:

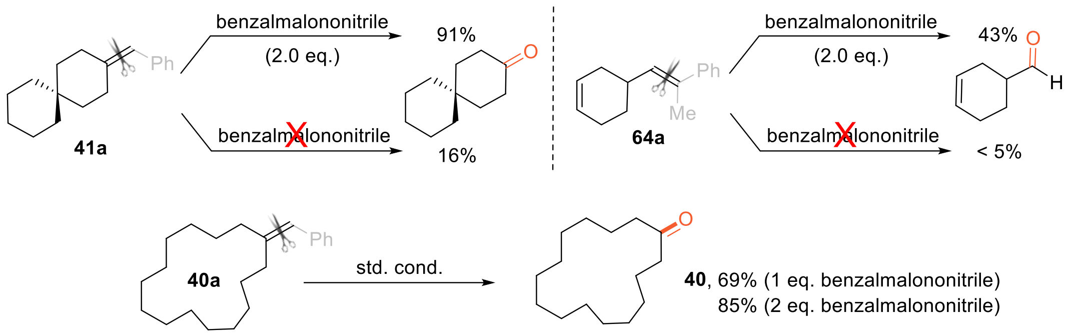

Unsuccessfully substrates:

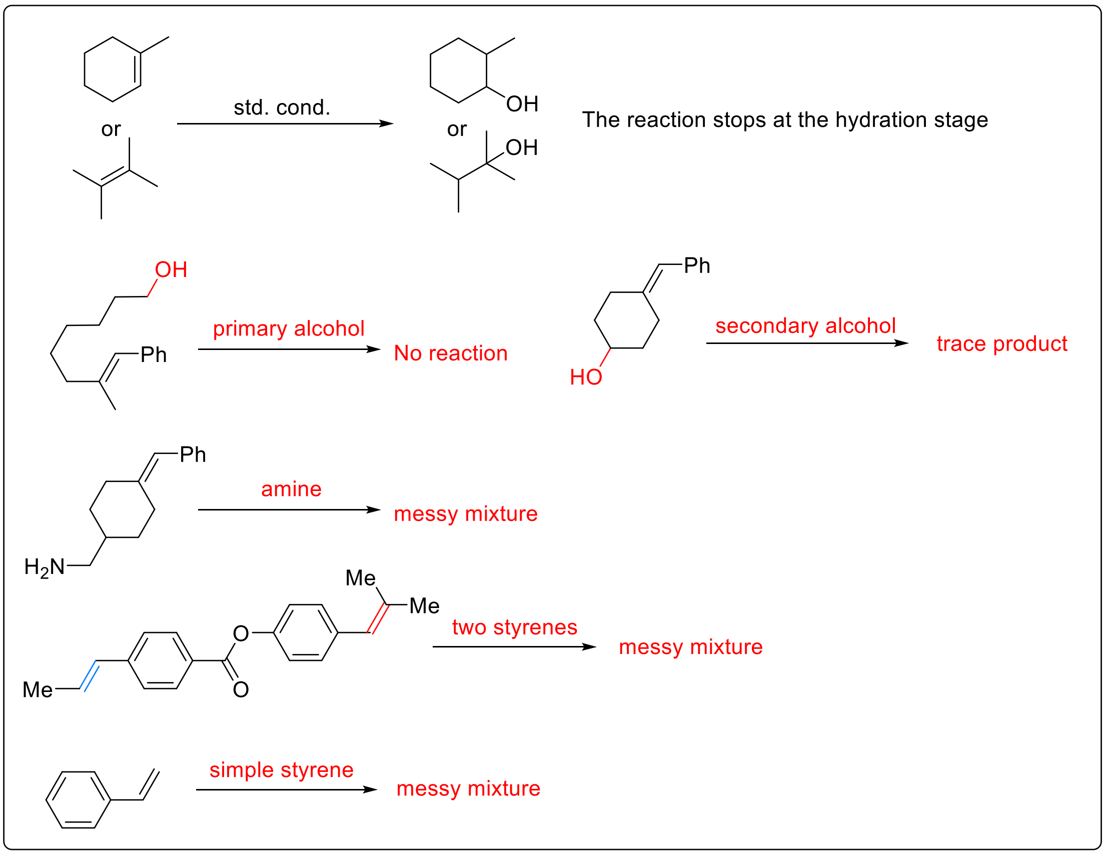

4. **General procedure**

4.1 General method for isolating the reducing fragment

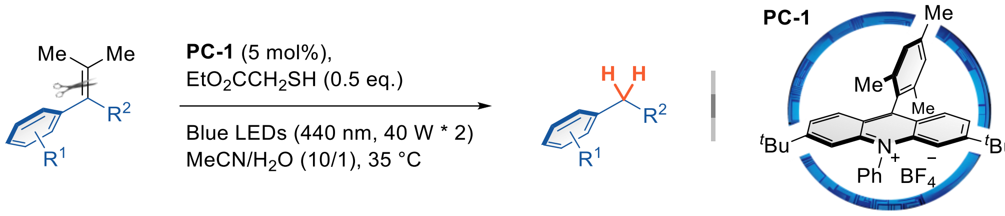

PC-1 (0.01 mmol, 5.7 mg) was weighed in an oven-dried 8 mL vial equipped with a magnetic stirring bar. Water (0.1 mL) and MeCN (1.0 mL) were added, followed by EtO_2CCH_2SH (0.1 mmol) and an arylalkene substrate (0.2 mmol). The reaction vessel was degassed, back-filled with argon, and placed in a water bath between two kessil lights (40 W*2). The vial was placed against the wall of the water bath to minimize light reflection (the distance of the Kessil lamps from the vials is about 5 cm, refer to **Supplementary Figure 1.** in the Supplementary Information for pictures of the reaction setup). The water bath was maintained at 35 °C to ensure stable temperature control. The progress of the reaction was monitored by TLC. Upon completion, the reaction mixture was concentrated and purified by silica gel flash column chromatography.

4.2 General method for isolating the oxidating fragment

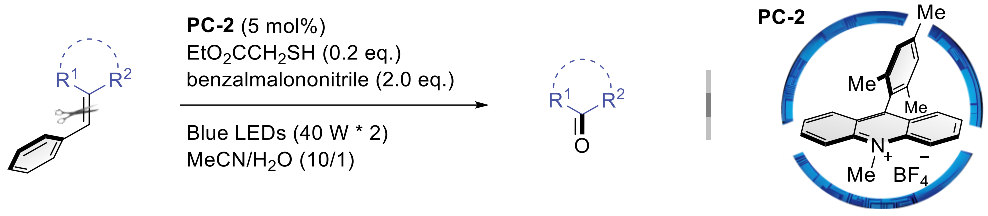

PC-2 (0.01 mmol, 4.0 mg) and benzalmalononitrile (0.4 mmol) were weighed in an oven-dried 8 mL vial equipped with a magnetic stirring bar. Water (0.1 mL) and MeCN (1.0 mL) were added, followed by EtO_2CCH_2SH (0.04 mmol) and an arylalkene substrate (0.2 mmol). The reaction vessel was degassed, back-filled with argon, and placed in a water bath between two kessil lights (40 W*2). The vial was placed against the wall of the water bath to minimize light reflection (the distance of the Kessil lamps from the vials is about 5 cm, refer to **Supplementary Figure 1.** for pictures of the reaction setup). The water bath was maintained at 35 °C to ensure stable temperature control. The progress of the reaction was monitored by TLC. Upon completion, the reaction mixture was concentrated and purified by silica gel flash column chromatography.

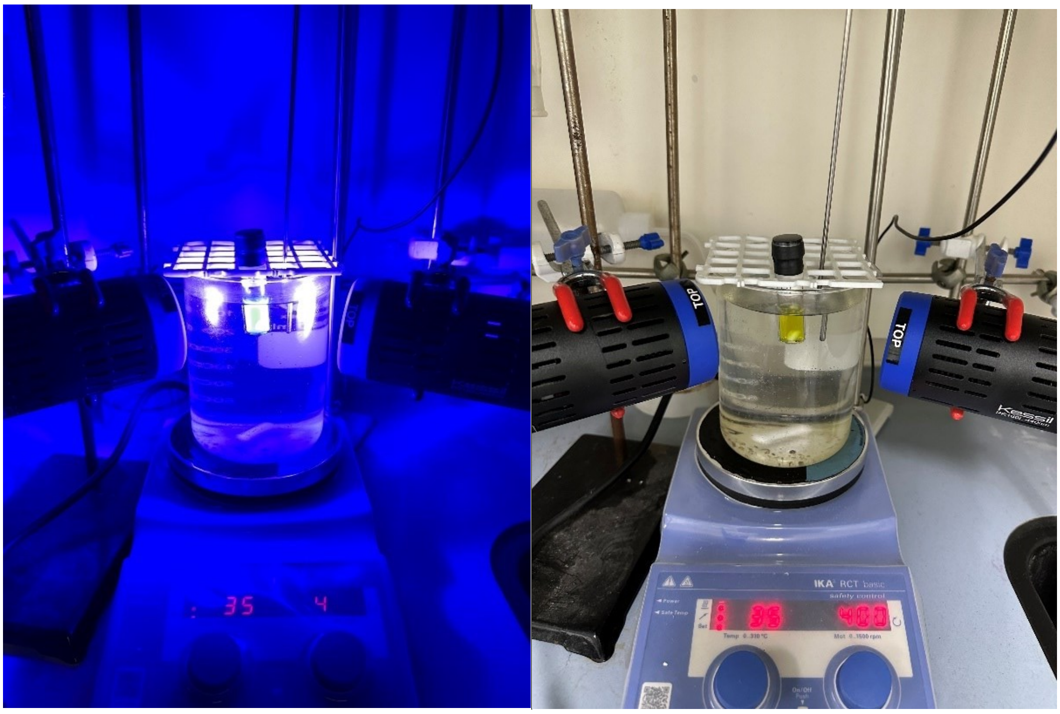

Supplementary Figure 1. Reaction setup (The distance of the Kessil lamps from the vials is about 5 cm)

4.3 Reaction conditions of gram scale

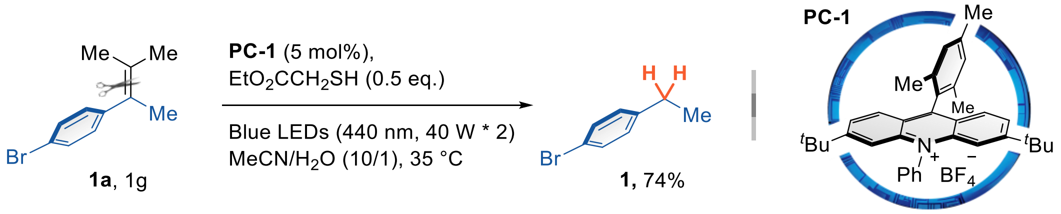

PC-1 (0.22 mmol, 127 mg) was weighed in an oven-dried 100 mL RB flask equipped with a magnetic stirring bar. H_2O (2.2 mL) and MeCN (22.0 mL) were added, followed by EtO_2CCH_2SH (2.23 mmol, 276.6 mg) and **1a** (4.46 mmol, 1g). The reaction was degassed, back-filled with argon, and placed between two kessil lights (40 W*2). (the distance of the Kessil lamps from the RB flask is about 5 cm, refer to **Supplementary Figure 2** below for pictures of the actually reaction setup). A fan is needed to ensure stable temperature control. The progress of the reaction was monitored by TLC. Upon completion, the reaction mixture was concentrated and purified by silica gel flash column chromatography.

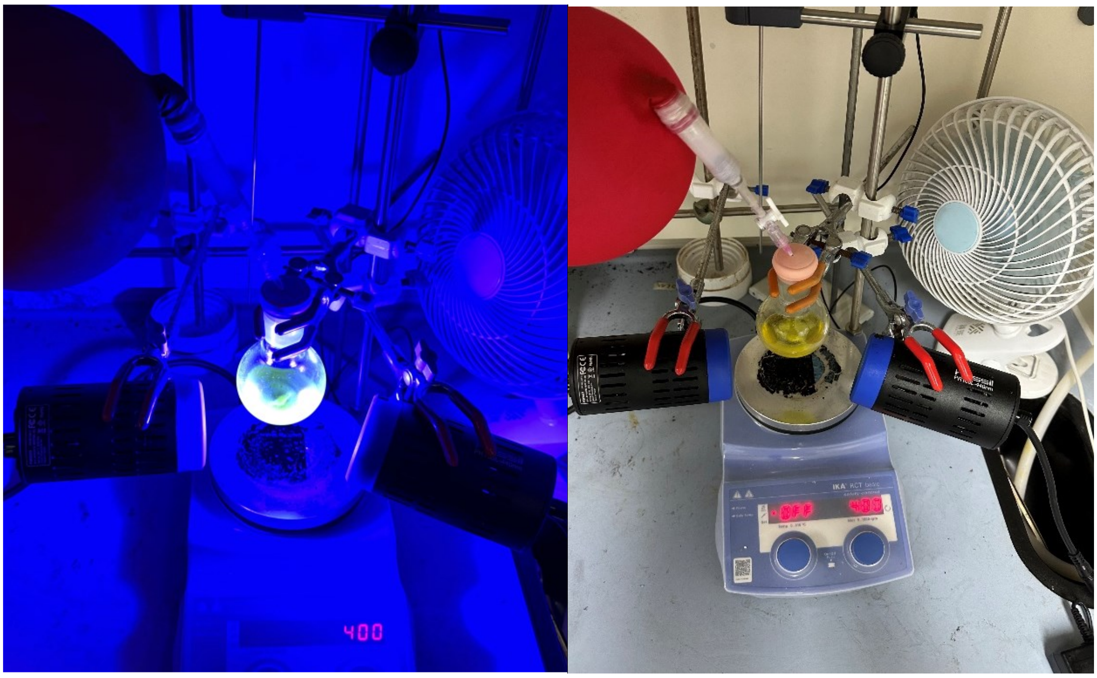

Supplementary Figure 2. Reaction setup (The distance of the Kessil lamps from the vials is about 5 cm)

All products were characterized as shown below:

1-bromo-4-ethylbenzene **1**

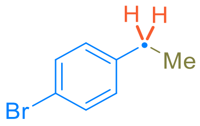

R_f = 0.9, PE/EA = 100:0 (v/v). Colorless oil, yield: 84%. **^{1}H NMR** (400 MHz, $CDCl_3$) δ 7.40 (d, J = 8.43 Hz, 2H), 7.08 (d, J = 8.34 Hz, 2H), 2.61 (q, J = 7.62 Hz, 2H), 1.23 (t, J = 7.63 Hz, 3H). **^{13}C NMR** (101 MHz, $CDCl_3$) δ 143.17, 131.34, 129.67, 119.28, 28.35, 15.51. **HRMS** (ESI-TOF) calculated for C_8H_9Br $[M+H]^+$: 184.9960, found: 184.9967.

Ethylbenzene **2**

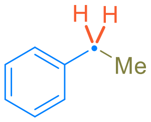

GC yield: 84%. **HRMS** (ESI-TOF) calculated for $C_8H_{10}[M+H]^+$: 107.0855, found: 107.0859.

1-(tert-butyl)-3-ethylbenzene **3**

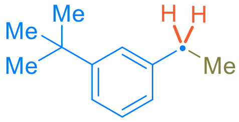

R_f= 0.9, PE/EA = 100:0 (v/v). Colorless oil, yield: 89%. **^{1}H NMR** (400 MHz, $CDCl_3$) δ 7.30 – 7.24 (m, 3H), 7.08 – 7.05 (m, 1H), 2.69 (q, J = 7.58 Hz, 2H), 1.37 (s, 9H), 1.29 (t, J = 7.60 Hz, 3H). **^{13}C NMR** (101 MHz, $CDCl_3$) δ 151.12, 143.87, 128.03, 124.96, 124.92, 122.61, 34.64, 31.45, 29.17, 15.75. **HRMS** (ESI-TOF) calculated for $C_{12}H_{18}$ $[M+H]^+$: 163.1481, found: 163.1482.

1-ethyl-4-phenoxybenzene **4**

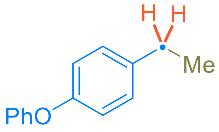

R_f= 0.8, PE/EA = 100:0 (v/v). Colorless oil, yield: 72%. **^{1}H NMR** (400 MHz, $CDCl_3$) δ 7.37 – 7.28 (m, 2H), 7.21 – 7.14 (m, 2H), 7.12 – 7.06 (m, 1H), 7.05 – 6.98 (m, 2H), 6.98 – 6.93 (m, 2H), 2.65 (q, J = 7.62 Hz, 2H), 1.26 (t, J = 7.62 Hz, 3H). **^{13}C NMR** (101 MHz, $CDCl_3$) δ 157.79, 154.91, 139.33, 129.67, 129.06, 122.85, 119.10, 118.46, 28.19, 15.77. **HRMS** (ESI-TOF) calculated for $C_{14}H_{14}O$ $[M+H]^+$: 199.1117, found: 199.1118.

1-ethyl-4-pentylbenzene **5**

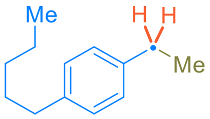

R_f= 0.9, PE/EA = 100:0 (v/v). Colorless oil, yield: 70%. **^{1}H NMR** (400 MHz, $CDCl_3$) δ 7.19 – 7.03 (m, 4H), 2.70 – 2.50 (m, 4H), 1.61 (p, *J* = 7.59 Hz, 2H), 1.40 – 1.29 (m, 4H), 1.24 (t, *J* = 7.59 Hz, 3H), 0.90 (t, *J* =7.59 Hz,3H). **^{13}C NMR** (101 MHz, $CDCl_3$) δ 141.40, 140.16,

128.33, 127.71, 35.55, 31.60, 31.33, 28.45, 22.59, 15.68, 14.07. **HRMS** (ESI-TOF) calculated for $C_{13}H_{20}$ [M+H]$^+$: 177.1638, found: 177.1639.

2-ethylbenzo[d]thiazole **6**

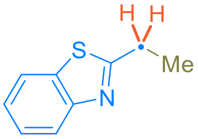

R_f = 0.5, PE/EA = 50:1 (v/v). Colorless oil, yield: 67%. **^{1}H NMR** (400 MHz, $CDCl_3$) δ 7.97 (d, J = 8.11 Hz, 1H), 7.84 (d, J = 8.69 Hz, 1H), 7.48 – 7.42 (m, 1H), 7.37 – 7.31 (m, 1H), 3.15 (q, J = 7.56 Hz, 2H), 1.47 (t, J = 7.60 Hz, 3H). **^{13}C NMR** (101 MHz, $CDCl_3$) δ 173.61, 153.26, 135.07, 125.90, 124.64, 122.50, 121.52, 27.79, 13.84. **HRMS** (ESI-TOF) calculated for C_9H_9NS [M+H]$^+$: 164.0528, found: 164.0528.

2-ethylbenzo[d]oxazole **7**

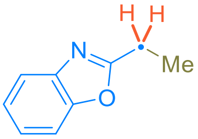

R_f = 0.5, PE/EA = 50:1 (v/v). Yellow oil, yield: 56%. **^{1}H NMR** (400 MHz, $CDCl_3$) δ 7.72 – 7.63 (m, 1H), 7.50 – 7.45 (m, 1H), 7.33 – 7.26 (m, 2H), 2.97 (q, J = 7.60 Hz, 2H), 1.45 (t, J = 7.59 Hz, 3H). **^{13}C NMR** (101 MHz, $CDCl_3$) δ 168.18, 150.82, 141.30, 124.46, 124.08, 119.52, 110.27, 22.18, 10.92. **HRMS** (ESI-TOF) calculated for C_9H_9NO [M+H]$^+$: 148.0757, found: 148.0757.

methyl 2-(p-tolyl)acetate **8**

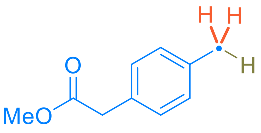

R_f = 0.3, PE/EA =50:1 (v/v). Colorless oil, yield: 94%. **^{1}H NMR** (400 MHz, $CDCl_3$) δ 7.21 – 7.12 (m, 4H), 3.69 (s, 3H), 3.60 (s, 2H), 2.34 (s, 3H). **^{13}C NMR** (101 MHz, $CDCl_3$) δ 172.28, 136.76, 130.94, 129.31, 129.13, 52.04, 40.81, 21.10. **HRMS** (ESI-TOF) calculated for $C_{10}H_{12}O_2$ [M+H]$^+$: 165.0910, found: 165.0911.

Ethylbenzene **9**

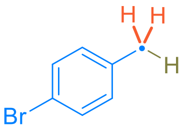

R_f = 0.9, PE/EA = 100:0 (v/v). Colorless oil, yield: 84%. **^{1}H NMR** (400 MHz, $CDCl_3$) δ 7.38 (d, J = 8.32 Hz, 2H), 7.06 (d, J = 7.99 Hz, 2H), 2.31 (s, 3H). **^{13}C NMR** (101 MHz, $CDCl_3$) δ 136.79, 131.26, 130.85, 119.08, 20.96. **HRMS** (ESI-TOF) calculated for C_7H_7Br [M+H]$^+$: 170.9804, found: 170.9805.

p-tolyl acetate **10**

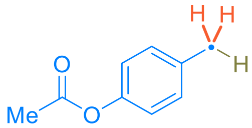

R_f = 0.7, PE/EA =100:0 (v/v). Colorless oil, yield: 82%. **^{1}H NMR** (400 MHz, $CDCl_3$) δ 7.18 (d, J = 7.99 Hz, 2H), 6.97 (d, J = 8.44 Hz, 2H), 2.35 (s, 3H), 2.29 (s, 3H). **^{13}C NMR** (101 MHz, $CDCl_3$) δ 169.78, 148.45, 135.50, 129.97, 121.26, 21.14, 20.88. **HRMS** (ESI-TOF) calculated for $C_9H_{10}O_2$ $[M+H]^+$: 151.0754, found: 151.0754.

4,4,5,5-tetramethyl-2-(p-tolyl)-1,3,2-dioxaborolane **11**

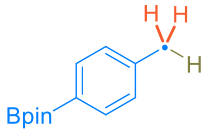

R_f = 0.4, PE/EA = 100:1 (v/v). White solid, yield: 50%. **^{1}H NMR** (400 MHz, $CDCl_3$) δ 7.72 (d, J = 7.95 Hz, 2H), 7.20 (d, J = 7.60 Hz, 2H), 2.38 (s, 3H), 1.35 (s, 12H). **^{13}C NMR** (101 MHz, $CDCl_3$) δ 141.42, 134.83, 128.55, 83.63, 24.88, 21.76. **HRMS** (ESI-TOF) calculated for $C_{13}H_{19}BO_2$ $[M+H]^+$: 219.1551, found: 219.1552.

2-fluoro-1,4-dimethylbenzene **12**

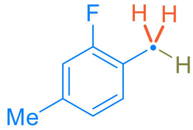

GC yield: 92%. **HRMS** (ESI-TOF) calculated for C_8H_9F $[M+H]^+$: 125.0761, found: 125.0763.

tert-butyl p-tolyl carbonate **13**

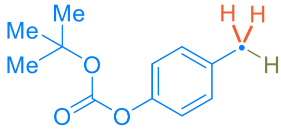

R_f = 0.4, PE/EA = 50:1 (v/v). Colorless oil, yield: 81%. **^{1}H NMR** (400 MHz, $CDCl_3$) δ 7.16 (d, J = 8.94 Hz, 2H), 7.05 (d, J = 8.49 Hz, 2H), 2.34 (s, 3H), 1.56 (s, 9H). **^{13}C NMR** (101 MHz, $CDCl_3$) δ 152.15, 148.89, 135.39, 129.87, 120.98, 83.36, 27.72, 20.85. **HRMS** (ESI-TOF) calculated for $C_{12}H_{16}O_3$ $[M+H]^+$: 209.1172, found: 209.1174.

methyl 4'-methyl-[1,1'-biphenyl]-4-carboxylate **14**

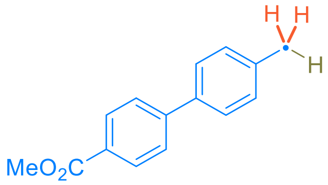

R_f = 0.3, PE/EA = 50:1 (v/v). White solid, yield: 98%. **^{1}H NMR** (400 MHz, $CDCl_3$) δ 8.10 (d, J = 8.42 Hz, 2H), 7.65 (d, J = 8.49 Hz, 2H), 7.53 (d, J = 8.21 Hz, 2H), 7.28 (d, J = 7.92 Hz, 2H), 3.94 (s, 3H), 2.41 (s, 3H).**^{13}C NMR** (101 MHz, $CDCl_3$) δ 167.08, 145.59, 138.14, 137.10, 130.11, 129.68, 128.60, 127.13, 126.81, 52.13, 21.19. **HRMS** (ESI-TOF) calculated for $C_{15}H_{14}O_2$ $[M+H]^+$: 227.1067, found: 227.1067.

4'-methyl-[1,1'-biphenyl]-2-carbonitrile **15**

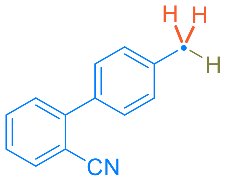

R_f = 0.3, PE/EA = 100:1 (v/v). White solid, yield: 74%. **^{1}H NMR** (400 MHz, $CDCl_3$) δ 7.75 (d, J = 7.78 Hz, 1H), 7.63 (td, J = 1.39, 7.66 Hz, 1H), 7.51 (d, J = 7.91 Hz, 1H), 7.47 (d, J = 8.17 Hz, 2H), 7.42 (td, J = 1.28, 7.62 Hz, 1H), 7.31 (d, J = 7.94 Hz, 2H), 2.43 (s, 3H). **^{13}C NMR** (101 MHz, $CDCl_3$) δ 145.55, 138.73, 135.29, 133.75, 132.80, 130.01, 129.48, 128.64, 127.30, 118.92, 111.21, 21.29. **HRMS** (ESI-TOF) calculated for $C_{14}H_{11}N$ $[M+H]^+$: 194.0964, found: 194.0965.

2-(4'-methyl-[1,1'-biphenyl]-4-yl)propan-2-ol **16**

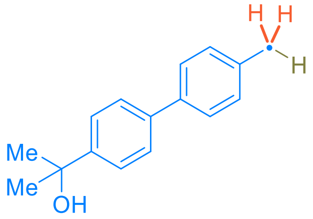

R_f = 0.5, PE/EA = 5:1 (v/v). White solid, yield: 64%. **^{1}H NMR** (400 MHz, Chloroform-*d*) δ 7.57 (s, 4H), 7.53 – 7.48 (m, 2H), 7.29 – 7.23 (m, 2H), 2.41 (s, 3H), 1.82 (s, 1H), 1.64 (s, 6H). **^{13}C NMR** (101 MHz, Chloroform-*d*) δ 147.88, 139.59, 137.98, 137.00, 129.50, 126.92, 126.79, 124.86, 72.47, 31.78, 21.12. **HRMS** (ESI-TOF) calculated for $C_{16}H_{18}O$ $[M+Na]^+$: 249.1250, found:249.1250.

hex-3-yn-1-yl 4'-methyl-[1,1'-biphenyl]-4-carboxylate **17**

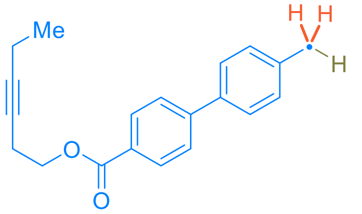

R_f = 0.5, PE/EA = 40:1 (v/v). White solid, yield: 43%. **^{1}H NMR** (400 MHz, Chloroform-*d*) δ 8.14 – 8.07 (m, 2H), 7.69 – 7.62 (m, 2H), 7.56 – 7.50 (m, 2H), 7.28 (d, *J* = 7.9 Hz, 2H), 4.45 – 4.36 (m, 2H), 2.64 (tt, *J* = 7.1, 2.4 Hz, 2H), 2.41 (s, 3H), 2.24 – 2.12 (m, 2H), 1.13 (t, *J* = 7.5 Hz, 3H). **^{13}C NMR** (101 MHz, Chloroform-*d*) δ 166.32, 145.66, 138.13, 137.13, 130.17, 129.66, 128.62, 127.12, 126.78, 83.56, 63.32, 21.16, 19.43, 14.14, 12.40. **HRMS** (ESI-TOF) calculated for $C_{20}H_{20}O_2$ $[M+H]^+$: 293.1536, found: 293.1535.

p-tolyl 4,4-dimethylpent-2-ynoate **18**

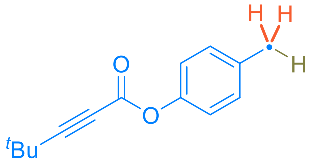

R_f = 0.5, PE/EA = 50:1 (v/v). White solid, yield: 69%. **^{1}H NMR** (400 MHz, Chloroform-*d*) δ 7.21 – 7.15 (m, 2H), 7.04 – 6.98 (m, 2H), 2.34 (s, 3H), 1.32 (s, 9H). **^{13}C**

NMR (101 MHz, Chloroform-*d*) δ 152.58, 147.98, 135.96, 130.01, 121.16, 98.99, 71.50, 29.89, 27.71, 20.88. **HRMS** (ESI-TOF) calculated for $C_{14}H_{16}O_2$ $[M+H]^+$: 217.1223, found: 217.1225.

1-(p-tolyl)propan-2-one **19**

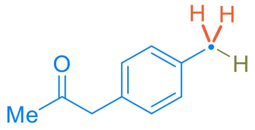

R_f = 0.4, PE/EA = 20:1 (v/v). Colorless oil, yield: 65%. **^{1}H NMR** (400 MHz, Chloroform-*d*) δ 7.15 (d, *J* = 7.8 Hz, 2H), 7.09 (d, *J* = 8.1 Hz, 2H), 3.65 (s, 2H), 2.34 (s, 3H), 2.14 (s, 3H). **^{13}C NMR** (101 MHz, Chloroform-*d*) δ 206.76, 136.72, 131.20, 129.48, 129.26, 50.68, 29.17, 21.08. **HRMS** (ESI-TOF) calculated for $C_{10}H_{12}O$ $[M+H]^+$: 149.0961, found: 149.0962.

1-phenyl-2-(p-tolyl)ethan-1-one **20**

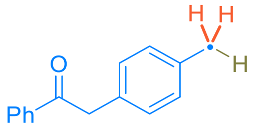

R_f = 0.4, PE/EA = 20:1 (v/v). White solid, yield: 64%. **^{1}H NMR** (400 MHz, Chloroform-*d*) δ 8.05 – 7.97 (m, 2H), 7.59 – 7.52 (m, 1H), 7.49 – 7.42 (m, 2H), 7.20 – 7.09 (m, 4H), 4.25 (s, 2H), 2.33 (s, 3H). **^{13}C NMR** (101 MHz, Chloroform-*d*) δ 197.84, 136.66, 136.50, 133.10, 131.45, 129.42, 129.32, 128.64, 128.63, 45.16, 21.09. **HRMS** (ESI-TOF) calculated for $C_{15}H_{14}O$ $[M+H]^+$: 211.1117, found: 211.1117.

p-tolyl cinnamate **21**

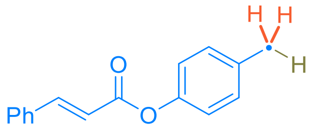

R_f = 0.5, PE/EA = 40:1 (v/v). White solid, yield: 51%. **^{1}H NMR** (400 MHz, Chloroform-*d*) δ 7.87 (d, *J* = 16.0 Hz, 1H), 7.64 – 7.54 (m, 2H), 7.48 – 7.39 (m, 3H), 7.21 (d, *J* = 8.2 Hz, 2H), 7.10 – 7.03 (m, 2H), 6.64 (d, *J* = 16.0 Hz, 1H), 2.37 (s, 3H). **^{13}C NMR** (101 MHz, Chloroform-*d*) δ 165.63, 148.59, 146.41, 135.44, 134.26, 130.66, 129.98, 129.01, 128.30, 121.32, 117.46, 20.92. **HRMS** (ESI-TOF) calculated for $C_{16}H_{14}O_2$ $[M+H]^+$: 239.1067, found: 239.1067.

p-tolyl (E)-octadec-9-enoate **22**

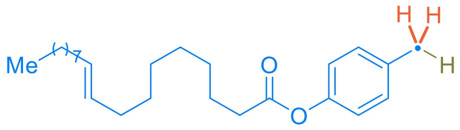

R_f = 0.5, PE/EA = 40:1 (v/v). Colorless oil, yield: 40%. **^{1}H NMR** (400 MHz, Chloroform-*d*) δ 7.20 – 7.13 (m, 2H), 6.98 – 6.91 (m, 2H), 5.43 – 5.31 (m, 2H), 2.53 (t, *J* = 7.5 Hz, 2H), 2.34 (s, 3H), 2.06 – 1.93 (m, 4H), 1.74 (p, *J* = 7.4 Hz, 2H), 1.46 – 1.30 (m, 10H), 1.29 – 1.22 (m, 10H), 0.92 – 0.84 (m, 3H). **^{13}C NMR** (101 MHz, Chloroform-*d*) δ 172.54, 148.53, 135.31, 130.52, 130.21, 129.90, 121.25, 34.42, 32.62, 32.56, 31.92, 29.67, 29.57, 29.50, 29.33, 29.20, 29.12, 29.09, 28.95, 24.99, 22.70, 20.86, 14.13. **HRMS** (ESI-TOF) calculated for $C_{25}H_{40}O_2$ $[M+H]^+$: 373.3101, found: 373.3101.

butylbenzene **23**

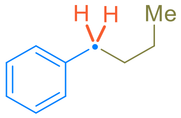

R_f = 0.9, PE/EA = 100:0 (v/v). Colorless oil, yield: 80%. **^{1}H NMR** (400 MHz, $CDCl_3$) δ 7.32 – 7.26 (m, 2H), 7.25 – 7.15 (m, 3H), 2.68 – 2.60 (m, 2H), 1.67 – 1.58 (m, 2H), 1.44 – 1.33 (m, 2H), 0.95 (t, J = 7.35 Hz, 3H). **^{13}C NMR** (101 MHz, $CDCl_3$) δ 142.95, 128.45, 128.25, 125.57, 35.71, 33.72, 22.42, 14.00. **HRMS** (ESI-TOF) calculated for $C_{10}H_{14}$ $[M+H]^+$: 135.1168, found: 135.1171.

methyl 5-phenylpentanoate **24**

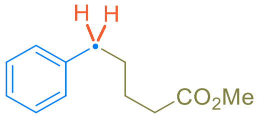

R_f = 0.3, PE/EA = 100:1 (v/v). Colorless oil, yield: 61%. **^{1}H NMR** (400 MHz, $CDCl_3$) δ 7.31 – 7.26 (m, 2H), 7.24 – 7.13 (m, 3H), 3.66 (s, 3H), 2.63 (t, J = 7.06 Hz, 2H), 2.34 (t, J = 7.09 Hz, 2H), 1.71 – 1.63 (m, 4H). **^{13}C NMR** (101 MHz, $CDCl_3$) δ 174.12, 142.14, 128.40, 128.33, 125.79, 51.51, 35.58, 33.96, 30.92, 24.59. **HRMS** (ESI-TOF) calculated for $C_{12}H_{16}O_2$ $[M+H]^+$: 193.1223, found: 193.1224.

1,4-diphenylbutane **25**

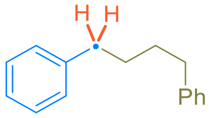

R_f = 0.9, PE/EA = 100:0 (v/v). Colorless oil, yield: 62%. **^{1}H NMR** (400 MHz, $CDCl_3$) δ 7.33 – 7.26 (m, 4H), 7.26 – 7.13 (m, 6H), 2.71 – 2.63 (m, 4H), 1.75 – 1.66 (m, 4H). **^{13}C NMR** (101 MHz, $CDCl_3$) δ 142.61, 128.46, 128.30, 125.69, 35.86, 31.14. **HRMS** (ESI-TOF) calculated for $C_{16}H_{18}$ $[M+Na]^+$: 233.1301, found: 233.130.

4-phenethyltetrahydro-2H-pyran **26**

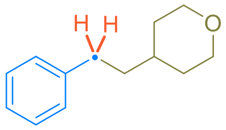

R_f = 0.5, PE/EA = 50:1 (v/v). White solid, yield: 75%. **^{1}H NMR** (400 MHz, $CDCl_3$) δ 7.32 – 7.26 (m, 2H), 7.23 – 7.14 (m, 3H), 3.96 (dd, J = 3.58, 11.55 Hz, 2H), 3.37 (td, J = 2.09, 11.74 Hz, 2H), 2.68 – 2.61 (m, 2H), 1.69 – 1.57 (m, 4H), 1.51 (tdd, J = 3.55, 6.82, 13.36 Hz, 1H), 1.38 – 1.28 (m, 2H). **^{13}C NMR** (101 MHz, $CDCl_3$) δ 142.56, 128.37, 128.34, 125.73, 68.10, 38.75, 34.52, 33.11, 32.69. **HRMS** (ESI-TOF) calculated for $C_{13}H_{18}O$ $[M+H]^+$: 191.1430, found: 191.1431.

(cyclohexylmethyl)benzene **27**

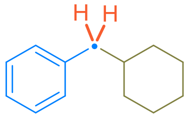

R_f = 0.9, PE/EA = 100:0 (v/v). Colorless oil, yield: 52%. **^{1}H NMR** (400 MHz,

$CDCl_3$) δ 7.30 – 7.25 (m, 2H), 7.22 – 7.08 (m, 3H), 2.49 (d, J = 7.12 Hz, 2H), 1.74 – 1.63 (m, 5H), 1.58 – 1.47 (m, 1H), 1.24 – 1.12 (m, 3H), 1.01 – 0.89 (m, 2H). **^{13}C NMR** (101 MHz, $CDCl_3$) δ 141.39, 129.20, 128.04, 125.57, 44.17, 39.81, 33.18, 26.60, 26.34. **HRMS** (ESI-TOF) calculated for $C_{13}H_{18}$ $[M+H]^+$: 175.1481, found: 175.1483.

Neopentylbenzene **28**

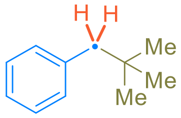

R_f = 0.9, PE/EA = 100:0 (v/v). Colorless oil, yield: 73%. **^{1}H NMR** (400 MHz, $CDCl_3$) δ 7.29 (t, J = 7.17 Hz, 2H), 7.22 (t, J = 7.27 Hz, 1H), 7.15 (d, J = 6.75 Hz, 2H), 2.52 (s, 2H), 0.93 (s, 9H). **^{13}C NMR** (101 MHz, $CDCl_3$) δ 139.77, 130.51, 127.62, 125.73, 50.26, 31.76, 29.41. **HRMS** (ESI-TOF) calculated for $C_{11}H_{16}$ $[M+H]^+$: 149.1325, found: 149.1326.

p-tolyl 4-sulfamoylbenzoate **29**

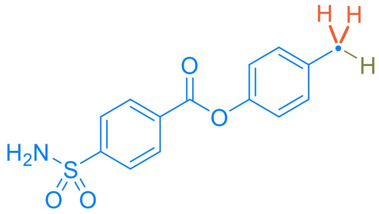

R_f = 0.3, PE/EA = 1:1 (v/v). White solid, yield: 75%. **^{1}H NMR** (400 MHz, Methanol-d_4) δ 8.33 (d, J = 8.6 Hz, 2H), 8.09 (d, J = 8.5 Hz, 2H), 7.27 (dd, J = 7.5, 1.4 Hz, 2H), 7.17 – 7.10 (m, 2H), 2.39 (s, 3H). **^{13}C NMR** (101 MHz, Methanol-d_4) δ 164.13, 148.66, 148.31, 135.71, 132.63, 130.23, 129.61, 126.13, 120.97, 19.49. **HRMS** (ESI-TOF) calculated for $C_{14}H_{13}NO_4S$ $[M+Na]^+$: 314.0457, found: 314.0457.

p-tolyl 4-(N,N-dipropylsulfamoyl)benzoate **30**

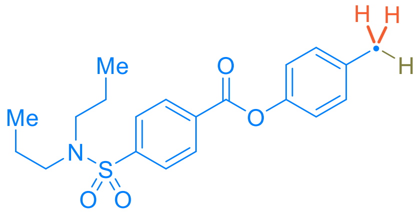

R_f = 0.3, PE/EA = 10:1 (v/v). White solid, yield: 64%. **^{1}H NMR** (400 MHz, $CDCl_3$) δ 8.31 (d, J = 8.47 Hz, 2H), 7.94 (d, J = 8.48 Hz, 2H), 7.24 (d, J = 8.09 Hz, 2H), 7.10 (d, J = 8.46 Hz, 2H), 3.20 – 3.06 (m, 4H), 2.38 (s, 3H), 1.60 – 1.53 (m, 4H), 0.88 (t, J = 7.38 Hz, 6H). **^{13}C NMR** (101 MHz, $CDCl_3$) δ 164.10, 148.41, 144.81, 135.98, 132.98, 130.79, 130.14, 127.15, 121.16, 49.94, 21.95, 20.94, 11.19. **HRMS** (ESI-TOF) calculated for $C_{20}H_{25}NO_4S$ $[M+H]^+$: 376.1577, found: 376.1577.

(1S,2R,5S)-2-isopropyl-5-methylcyclohexyl 4'-methyl-[1,1'-biphenyl]-4-carboxylate **31**

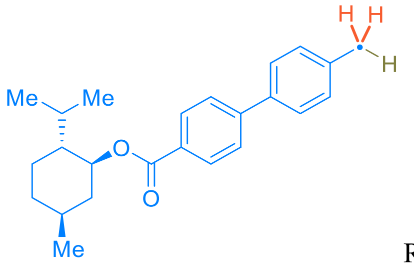

R_f = 0.4, PE/EA = 50:1 (v/v). White solid, yield: 89%. **^{1}H NMR** (400 MHz, $CDCl_3$) δ 8.11 (d, J = 8.39 Hz, 2H), 7.65 (d, J = 8.44 Hz, 2H), 7.54 (d, J = 8.15 Hz, 2H), 7.28 (d, J = 7.93 Hz, 2H), 4.97 (td, J = 4.41, 10.84 Hz, 1H), 2.42 (s, 3H), 2.16 (d, J = 11.99 Hz, 1H), 2.06 – 1.96 (m, 1H), 1.80 – 1.70 (m, 2H), 1.64 – 1.54 (m, 2H), 1.23 – 1.08 (m, 2H), 0.95 (dd, J = 2.90, 6.80 Hz, 7H), 0.83 (d, J = 6.95 Hz, 3H). **^{13}C NMR** (101 MHz, $CDCl_3$) δ 166.06, 145.43, 138.06, 137.25, 130.09, 129.68, 129.33, 127.14, 126.79, 47.34, 41.04, 34.37, 31.49, 26.56, 23.69, 22.10, 21.19, 20.82, 16.58. **HRMS** (ESI-TOF) calculated for $C_{24}H_{30}O_2$ $[M+Na]^+$: 373.2138, found: 373.2138.

p-tolyl 2-(11-oxo-6,11-dihydrodibenzo[b,e]oxepin-2-yl)acetate **32**

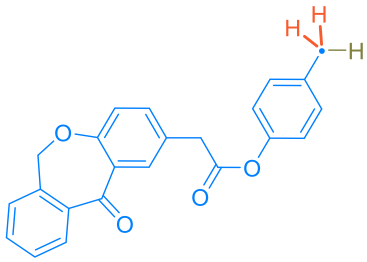

R_f = 0.3, PE/EA = 10:1 (v/v). Colorless oil, yield: 55%. **^{1}H NMR** (400 MHz, $CDCl_3$) δ 8.22 (d, J = 2.44 Hz, 1H), 7.90 (dd, J = 1.40, 7.61 Hz, 1H), 7.60 – 7.45 (m, 3H), 7.37 (dd, J = 1.28, 7.42 Hz, 1H), 7.15 (d, J = 8.24 Hz, 2H), 7.07 (d, J = 8.44 Hz, 1H), 6.96 (d, J = 8.46 Hz, 2H), 5.20 (s, 2H), 3.87 (s, 2H), 2.33 (s, 3H). **^{13}C NMR** (101 MHz, $CDCl_3$) δ 190.86, 170.08, 160.63, 148.45, 140.47, 136.33, 135.57, 135.54, 132.84, 132.62, 129.93, 129.53, 129.32, 127.86, 127.35, 125.24, 121.25, 121.12, 40.30, 20.88. **HRMS** (ESI-TOF) calculated for $C_{23}H_{18}O_4$ $[M+H]^+$: 359.1278, found: 359.1278.

p-tolyl (2S,5R)-3,3-dimethyl-7-oxo-4-thia-1-azabicyclo[3.2.0]heptane-2-carboxylate 4,4-dioxide **33**

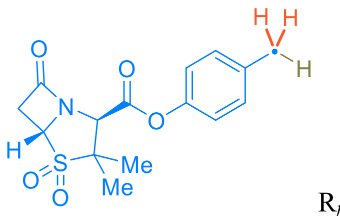

R_f = 0.3, PE/EA = 3:1 (v/v). Yellow oil, yield: 52%. **^{1}H NMR** (400 MHz, $CDCl_3$) δ 7.21 (d, J = 8.37 Hz, 2H), 6.99 (d, J = 8.51 Hz, 2H), 4.69 – 4.65 (m, 1H), 4.62 (s, 1H), 3.55 – 3.44 (m, 2H), 2.36 (s, 3H), 1.72 (s, 3H), 1.58 (s, 3H). **^{13}C NMR** (101 MHz, $CDCl_3$) δ 170.71, 165.70, 147.60, 136.62, 130.28, 120.63, 63.26, 62.88, 61.21, 38.48, 20.90, 20.51, 18.81. **HRMS** (ESI-TOF) calculated for $C_{15}H_{17}NO_5S$ $[M+Na]^+$: 346.0720, found: 346.0722.

((3aS,5aR,8aR,8bS)-2,2,7,7-tetramethyltetrahydro-3aH-bis([1,3]dioxolo)[4,5-b:4',5'-d]pyran-3a-

yl)methyl 4'-methyl-[1,1'-biphenyl]-2-carboxylate **34**

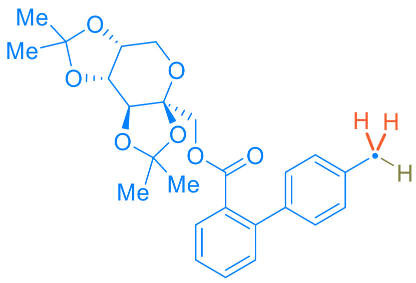

R_f = 0.4, PE/EA = 10:1 (v/v). White solid, yield: 83%. **^{1}H NMR** (400 MHz, $CDCl_3$) δ 7.80 (dd, J = 1.47, 8.03 Hz, 1H), 7.51 (td, J = 1.42, 7.52 Hz, 1H), 7.41 – 7.35 (m, 2H), 7.23 (s, 4H), 4.41 – 4.35 (m, 2H), 4.16 (dd, J = 1.76, 7.84 Hz, 1H), 3.89 (d, J = 11.61 Hz, 1H), 3.83 (dd, J = 1.93, 12.99 Hz, 1H), 3.69 (d, J = 13.00 Hz, 1H), 3.34 (d, J = 2.67 Hz, 1H), 2.39 (s, 3H), 1.43 (d, J = 7.38 Hz, 6H), 1.35 (s, 3H), 1.12 (s, 3H). **^{13}C NMR** (101 MHz, $CDCl_3$) δ 168.65, 141.92, 138.10, 137.01, 131.29, 130.97, 130.48, 129.95, 129.37, 128.19, 126.96, 108.93, 108.66, 101.38, 70.85, 69.96, 69.89, 65.61, 61.31, 26.50, 25.94, 25.29, 24.22, 21.19. **HRMS** (ESI-TOF) calculated for $C_{26}H_{30}O_7$ $[M+H]^+$: 455.2064, found: 455.2063.

1-methyl-4-propylbenzene **35**

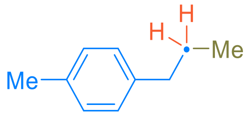

GC yield: 54%. **HRMS** (ESI-TOF) calculated for $C_{10}H_{14}$ $[M+H]^+$: 135.1168, found: 135.1168.

1-butyl-4-methylbenzene **36**

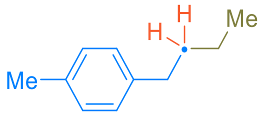

GC yield: 59%. **HRMS** (ESI-TOF) calculated for $C_{11}H_{16}$ $[M+H]^+$: 149.1325, found: 149.1325.

1-ethyl-4-propylbenzene **37**

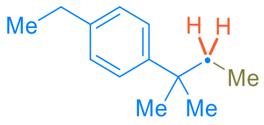

R_f = 0.9, PE/EA = 100:0 (v/v). Colorless oil, yield: 62%. **^{1}H NMR (**400 MHz, Chloroform-*d*) δ 7.26 (d, *J* = 8.3 Hz, 2H), 7.17 – 7.12 (m, 2H), 2.64 (q, *J* = 7.6 Hz, 2H), 1.64 (q, *J* = 7.5 Hz, 2H), 1.30 – 1.22 (m, 9H), 0.70 (t, *J* = 7.4 Hz, 3H). **^{13}C NMR** (101 MHz, Chloroform-*d*) δ 146.69, 140.99, 127.41, 125.85, 37.54, 36.88, 28.49, 28.29, 28.26, 15.46, 9.17. **HRMS** (ESI-TOF) calculated for $C_{13}H_{20}$ $[M+H]^+$: 177.1638, found: 177.1638.

Adamantan-2-one **38**

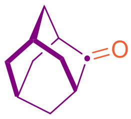

R_f= 0.3, PE/EA = 50:1 (v/v). White solid, yield: 73%. **^{1}H NMR** (400 MHz, $CDCl_3$) δ 2.52 (s, 2H), 2.09 – 1.89 (m, 12H). **^{13}C NMR** (101 MHz, $CDCl_3$) δ 218.50, 46.97, 39.25, 36.28, 27.43. **HRMS** (ESI-TOF) calculated for $C_{10}H_{14}O$ $[M+H]^+$: 151.1117, found: 151.1117.

Cyclododecanone **39**

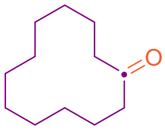

R_f= 0.3, PE/EA = 50:1 (v/v). White solid, yield: 65%. **^{1}H NMR** (400 MHz, $CDCl_3$) δ 2.48 – 2.41 (m, 4H), 1.74 – 1.65 (m, 4H), 1.33 – 1.21 (m, 14H). **^{13}C NMR** (101 MHz, $CDCl_3$) δ 212.96, 40.38, 24.75, 24.59, 24.21, 22.56. **HRMS** (ESI-TOF) calculated for $C_{12}H_{22}O$ $[M+H]^+$: 183.1743, found: 183.1744.

Cyclopentadecanone **40**

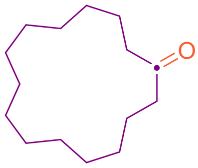

R_f= 0.3, PE/EA = 50:1 (v/v). White solid, yield: 85%. **^{1}H NMR** (400 MHz, CDCl3) δ 2.41 (t, J = 6.70 Hz, 4H), 1.68 – 1.59 (m, 4H), 1.35 – 1.25 (m, 20H). **^{13}C NMR** (101 MHz, CDCl3) δ 212.82, 42.11, 27.58, 26.77, 26.72, 26.43, 26.29, 23.46. **HRMS** (ESI-TOF) calculated for $C_{15}H_{28}O$ $[M+H]^+$: 225.2213, found: 225.2213.

Spiro[5.5]undecan-3-one **41**

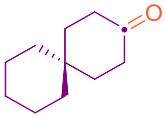

R_f= 0.3, PE/EA = 50:1 (v/v). White solid, yield: 91%. **^{1}H NMR** (400 MHz, CDCl3) δ 2.40 – 2.22 (m, 4H), 1.74 – 1.65 (m, 4H), 1.54 – 1.38 (m, 10H). **^{13}C NMR** (101 MHz, CDCl3) δ 213.14, 37.24, 36.18, 35.81, 32.04, 26.69, 21.97. **HRMS** (ESI-TOF) calculated for $C_{11}H_{18}O$ $[M+H]^+$: 167.1430, found: 167.1431.

1,4-dioxaspiro[4.5]decan-8-one **42**

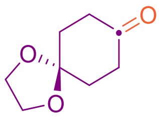

R_f= 0.3, PE/EA = 20:1 (v/v). White solid, yield: 84%. **^{1}H NMR** (400 MHz, $CDCl_3$) δ 4.01 (s, 1H), 2.49 (t, J = 7.11 Hz, 1H), 1.99 (t, J = 7.19 Hz, 1H). **^{13}C NMR** (101 MHz, $CDCl_3$) δ

210.42, 107.13, 64.67, 38.19, 33.87. **HRMS** (ESI-TOF) calculated for $C_8H_{12}O_3$ $[M+H]^+$: 157.0859, found: 157.0860.

4-cyano-4-pjemylcyclohexanone **43**

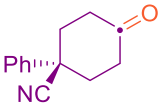

R_f= 0.2, PE/EA = 20:1 (v/v). White solid, yield: 70%. **^{1}H NMR** (400 MHz, CDCl3) δ 7.54 – 7.50 (m, 2H), 7.46 – 7.41 (m, 2H), 7.40 – 7.34 (m, 1H), 2.98 – 2.87 (m, 2H), 2.62 – 2.54 (m, 2H), 2.53 – 2.45 (m, 2H), 2.30 (td, J = 4.21, 13.83 Hz, 2H). **^{13}C NMR** (101 MHz, CDCl3) δ 207.32, 138.56, 129.30, 128.63, 125.48, 121.30, 43.12, 38.62, 36.99. **HRMS** (ESI-TOF) calculated for $C_{13}H_{13}NO$ $[M+Na]^+$: 222.0889, found: 222.0891.

N-(tert-Butoxycarbonyl)-4-piperidone **44**

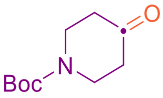

R_f= 0.4, PE/EA = 20:1 (v/v). White solid, yield: 32%. **^{1}H NMR** (400 MHz, CDCl3) δ 3.70 (t, J = 6.23 Hz, 4H), 2.42 (t, J = 6.22 Hz, 4H), 1.48 (s, 9H). **^{13}C NMR** (101 MHz, CDCl3) δ 207.85, 154.48, 80.45, 41.18, 28.37. **HRMS** (ESI-TOF) calculated for $C_{10}H_{17}NO_3$ $[M+Na]^+$: 222.1101, found: 222.1102.

Tetrahydro-4H-pyran-4-one **45**

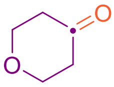

R_f= 0.3, PE/EA = 50:1 (v/v). colorless oil, yield: 85%. **^{1}H** NMR (400 MHz, $CDCl_3$) δ 3.96 (t, J = 5.94 Hz, 4H), 2.49 (t, J = 6.12 Hz, 4H). **^{13}C** NMR (101 MHz, $CDCl_3$) δ 206.59, 67.83, 42.89. **HRMS** (ESI-TOF) calculated for $C_5H_8O_2$ $[M+Na]^+$: 123.0417, found: 123.0419.

4,4-difluorocyclohexan-1-one **46**

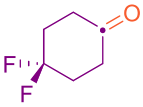

R_f= 0.3, PE/EA = 50:1 (v/v). Yellow oil, yield: 87%. **^{1}H NMR** (400 MHz, $CDCl_3$) δ 2.54 (t, J = 7.22 Hz, 2H), 2.37 – 2.23 (m, 2H). **^{13}C NMR** (101 MHz, $CDCl_3$) δ 207.23, 121.53 (t, J = 241.6 Hz), 36.60 (t, J = 5.6 Hz), 32.68 (t, J = 26.0 Hz). **^{19}F NMR** (376 MHz, $CDCl_3$) δ -100.32 (p, J = 13.02 Hz). **HRMS** (ESI-TOF) calculated for $C_6H_8F_2O$ $[M+H]^+$: 135.0616, found: 135.0617.

3-(2-oxocyclohexyl)propanenitrile **47**

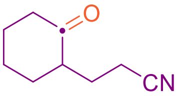

R_f= 0.3, PE/EA = 20:1 (v/v). Light yellow oil, yield: 68%. **^{1}H NMR** (400 MHz, $CDCl_3$) δ 2.52 – 2.41 (m, 3H), 2.41 – 2.19 (m, 2H), 2.18 – 1.98 (m, 3H), 1.93 – 1.84 (m, 1H), 1.79 – 1.55 (m, 2H), 1.55 – 1.44 (m, 1H), 1.44 – 1.32 (m, 1H). **^{13}C NMR** (101 MHz, $CDCl_3$) δ 211.90,

119.80, 48.86, 42.27, 34.32, 28.02, 25.59, 25.18, 15.27. **HRMS** (ESI-TOF) calculated for $C_9H_{13}NO$ $[M+H]^+$: 152.1070, found: 152.1070.

(1R,3S,5s,7s)-5-hydroxyadamantan-2-one **48**

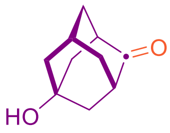

R_f = 0.3, PE/EA = 1:1 (v/v). white solid, yield: 92%. **^{1}H NMR** (400 MHz, Chloroform-*d*) δ 2.60 (t, *J* = 3.3 Hz, 2H), 2.38 – 2.28 (m, 1H), 2.11 – 2.02 (m, 2H), 1.94 (tdd, *J* = 11.1, 4.5, 2.7 Hz, 8H), 1.81 (s, 1H). **^{13}C NMR** (101 MHz, Chloroform-*d*) δ 216.67, 67.21, 46.98, 45.02, 44.16, 38.16, 29.83. **HRMS** (ESI-TOF) calculated for $C_{10}H_{14}O$ $[M+H]^+$: 167.1067, found: 167.1067.

octahydro-5H-4,7-methanoinden-5-one **49**

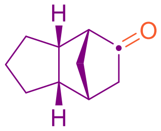

R_f= 0.3, PE/EA = 50:1 (v/v). Colorless oil, yield: 67%. **^{1}H NMR** (400 MHz, $CDCl_3$) δ 2.43 – 2.31 (m, 2H), 2.17 – 1.88 (m, 5H), 1.83 – 1.71 (m, 3H), 1.55 – 1.47 (m, 1H), 1.40 – 1.25 (m, 1H), 1.15 – 0.98 (m, 2H). **^{13}C NMR** (101 MHz, $CDCl_3$) δ 218.25, 54.34, 46.89, 44.54, 41.92, 39.71, 32.34, 31.64, 31.43, 28.12. **HRMS** (ESI-TOF) calculated for $C_{10}H_{14}NO$ $[M+H]^+$: 151.1117, found: 151.1118.

4-phenylbutan-2-one **50**

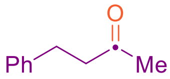

R_f= 0.3, PE/EA = 50:1 (v/v). Colorless oil, yield: 66%. **^{1}H NMR** (400 MHz, $CDCl_3$) δ 7.32 – 7.26 (m, 2H), 7.24 – 7.13 (m, 3H), 2.90 (t, J = 7.84 Hz, 2H), 2.77 (t, J = 7.35 Hz, 2H), 2.15 (s, 3H). **^{13}C NMR** (101 MHz, $CDCl_3$) δ 208.01, 141.01, 128.53, 128.32, 126.14, 45.21, 30.12, 29.74. **HRMS** (ESI-TOF) calculated for $C_{10}H_{12}O$ $[M+H]^+$: 149.0961, found: 149.0962.

4-(4-(trifluoromethyl)phenyl)butan-2-one **51**

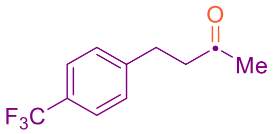

R_f= 0.3, PE/EA = 50:1 (v/v). Colorless oil, yield: 94%. **^{1}H NMR** (400 MHz, $CDCl_3$) δ 7.52 (d, J = 8.03 Hz, 2H), 7.29 (d, J = 8.01 Hz, 2H), 2.94 (t, J = 7.45 Hz, 2H), 2.78 (t, J = 7.46 Hz, 2H), 2.15 (s, 3H). **^{13}C NMR** (101 MHz, $CDCl_3$) δ 207.23 , 145.20 (d, J =1.5 Hz) , 128.71 , 128.49 (q, J = 32.2 Hz), 125.41 (q, J = 3.7 Hz), 124.28 (q, J = 271.7 Hz), 44.59 , 30.09 , 29.36 . **^{19}F NMR** (376 MHz, $CDCl_3$) δ -62.37. **HRMS** (ESI-TOF) calculated for $C_{11}H_{11}F_3O$ $[M+H]^+$: 217.0835, found: 217.0836.

6-chlorohexan-2-one **52**

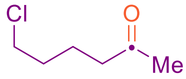

R_f= 0.3, PE/EA = 50:1 (v/v). Yellow oil, yield: 84%. **^{1}H NMR** (400 MHz, $CDCl_3$) δ 3.52 (t, J = 6.26 Hz, 2H), 2.46 (t, J = 6.86 Hz, 2H), 2.13 (s, 3H), 1.81 – 1.66 (m, 4H). **^{13}C NMR** (101 MHz, $CDCl_3$) δ 208.33, 44.65, 42.67, 31.86, 29.92, 21.01. **HRMS** (ESI-TOF) calculated for $C_6H_{11}ClO$ $[M+H]^+$: 135.0571, found: 135.0573.

tridecan-2-one **53**

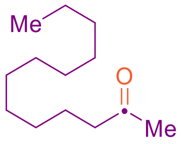

R_f= 0.3, PE/EA = 50:1 (v/v). White solid, yield: 80%.**^{1}H NMR** (400 MHz, $CDCl_3$) δ 2.40 (t, J = 7.48 Hz, 2H), 2.12 (s, 3H), 1.61 – 1.50 (m, 2H), 1.31 – 1.20 (m, 16H), 0.86 (t, J = 6.75 Hz, 3H). **^{13}C NMR** (101 MHz, $CDCl_3$) δ 209.46, 43.84, 31.92, 29.87, 29.62, 29.49, 29.42, 29.35, 29.19, 23.87, 22.70, 14.13. **HRMS** (ESI-TOF) calculated for $C_{13}H_{26}O$ $[M+H]^+$: 199.2056, found: 199.2058.

pentadecan-8-one **54**

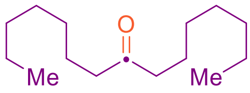

R_f= 0.3, PE/EA = 50:1 (v/v). White solid, yield: 77%. **^{1}H NMR** (400 MHz, $CDCl_3$) δ 2.37 (t, J = 7.49 Hz, 4H), 1.59 – 1.49 (m, 4H), 1.31 – 1.21 (m, 16H), 0.90 – 0.83 (m, 6H). **^{13}C NMR** (101 MHz, $CDCl_3$) δ 211.76, 42.82, 31.69, 29.24, 29.09, 23.90, 22.61, 14.06. **HRMS** (ESI-TOF) calculated for $C_{15}H_{30}O$ $[M+H]^+$: 227.2369, found: 227.2369.

ethyl 5-oxohexanoate **55**

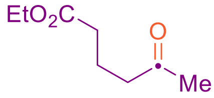

R_f= 0.4, PE/EA = 10:1 (v/v). Colorless oil, yield: 84%. **^{1}H NMR** (400 MHz, $CDCl_3$) δ 4.08 (q, J = 7.15 Hz, 2H), 2.47 (t, J = 7.23 Hz, 2H), 2.29 (t, J = 7.26 Hz, 2H), 2.10 (s, 3H), 1.92 – 1.79 (m, 2H), 1.21 (t, J = 7.16 Hz, 3H). **^{13}C NMR** (101 MHz, $CDCl_3$) δ 208.06, 173.16, 60.33, 42.45, 33.22, 29.93, 18.84, 14.21. **HRMS** (ESI-TOF) calculated for $C_8H_{14}O_3$ $[M+H]^+$: 159.1016, found: 159.1017.

decane-2,5-dione **56**

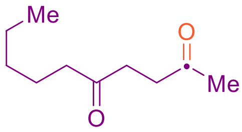

R_f= 0.4, PE/EA = 10:1 (v/v). Yellow oil, yield: 68%. **^{1}H NMR** (400 MHz, $CDCl_3$) δ 2.73 – 2.62 (m, 4H), 2.43 (t, J = 7.49 Hz, 2H), 2.18 (s, 3H), 1.62 – 1.52 (m, 2H), 1.33 – 1.22 (m, 4H), 0.87 (t, J = 7.04 Hz, 3H). **^{13}C NMR** (101 MHz, $CDCl_3$) δ 209.74, 207.38, 42.81, 36.90, 36.04, 31.37, 30.00, 23.52, 22.45, 13.93. **HRMS** (ESI-TOF) calculated for $C_{10}H_{18}O_2$ $[M+H]^+$:

171.1380, found: 171.1380.

2-(5-oxohexyl)isoindoline-1,3-dione **57**

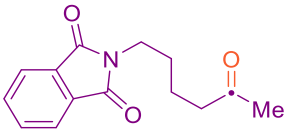

R_f = 0.3, PE/EA = 5:1 (v/v). Colorless oil, yield: 80%. **^{1}H NMR** (400 MHz, $CDCl_3$) δ 7.43 – 7.36 (m, 2H), 7.31 – 7.24 (m, 2H), 3.25 (t, J = 6.93 Hz, 2H), 2.05 (t, J = 7.14 Hz, 2H), 1.69 (s, 3H), 1.30 – 1.13 (m, 4H). **^{13}C NMR** (101 MHz, $CDCl_3$) δ 208.40, 168.41, 133.94, 132.09, 123.22, 42.87, 37.49, 29.98, 27.93, 20.77. **HRMS** (ESI-TOF) calculated for $C_{14}H_{15}NO_3$ $[M+H]^+$: 246.1125, found: 246.1125.

(5S,8S,9S,10S,13S,14S)-10,13-dimethylhexadecahydro-3H-cyclopenta[a]phenanthren-3-one **58**

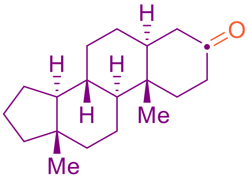

R_f = 0.4, PE/EA = 30:1 (v/v). White solid, yield: 98%. **^{1}H NMR** (400 MHz, $CDCl_3$) δ 2.45 – 2.18 (m, 3H), 2.13 – 1.95 (m, 2H), 1.79 – 1.68 (m, 2H), 1.68 – 1.47 (m, 5H), 1.47 – 1.23 (m, 6H), 1.23 – 1.07 (m, 3H), 1.06 – 0.83 (m, 5H), 0.82 – 0.61 (m, 4H). **^{13}C NMR** (101 MHz, $CDCl_3$) δ 212.29, 54.35, 54.06, 46.74, 44.76, 40.84, 40.38, 38.78, 38.66, 38.24, 35.78, 35.75, 32.09, 29.00, 25.54, 21.47, 20.52, 17.52, 11.52. **HRMS** (ESI-TOF) calculated for $C_{19}H_{30}O$ $[M+H]^+$: 275.2369, found: 275.2368.

8-oxodecyl 2-(2-fluoro-[1,1'-biphenyl]-4-yl)propanoate **59**

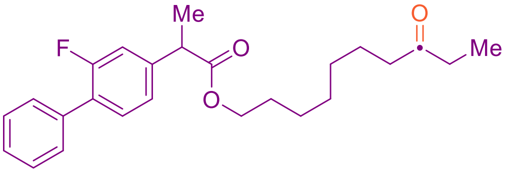

R_f = 0.3, PE/EA = 30:1 (v/v). Light yellow oil, yield: 78%. **^{1}H NMR** (400 MHz, $CDCl_3$) δ 7.57 – 7.49 (m, 2H), 7.47 – 7.32 (m, 4H), 7.19 – 7.08 (m, 2H), 4.08 (t, J = 6.63 Hz, 2H), 3.74 (q, J = 7.15 Hz, 1H), 2.49 – 2.22 (m, 4H), 1.64 – 1.49 (m, 7H), 1.31 – 1.20 (m, 6H), 1.03 (t, J = 7.32 Hz, 3H). **^{13}C NMR** (101 MHz, $CDCl_3$) δ 211.88, 174.06, 159.66 (d, *J* = 248.2 Hz), 142.01 (d, *J* = 7.6 Hz), 135.53, 130.75 (d, *J* = 4.0 Hz), 128.95 (d, *J* = 2.9 Hz), 128.46, 127.79, 127.67, 123.58 (d, *J* = 3.3 Hz), 115.25 (d, *J* = 23.6 Hz), 65.03, 45.10, 42.30, 35.87, 29.12, 28.97, 28.47, 25.65, 23.75, 18.32, 7.86. **^{19}F NMR** (376 MHz, $CDCl_3$) δ -117.78. **HRMS** (ESI-TOF) calculated for $C_{25}H_{31}FO_3$ $[M+H]^+$: 399.2330, found: 399.2332.

Dodecanal **60**

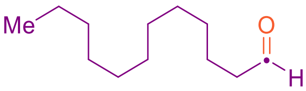

R_f = 0.3, PE/EA = 50:1 (v/v). Colorless oil, yield: 63%. **^{1}H NMR** (400 MHz, $CDCl_3$) δ 9.76 (t, J = 1.88 Hz, 1H), 2.42 (td, J = 1.94, 7.39 Hz, 2H), 1.66 – 1.58 (m, 2H), 1.31 – 1.23 (m, 16H), 0.88 (t, J = 6.75 Hz, 3H). **^{13}C NMR** (101 MHz, $CDCl_3$) δ 203.05, 43.95, 31.92,

29.61, 29.60, 29.44, 29.37, 29.34, 29.18, 22.70, 22.10, 14.14. **HRMS** (ESI-TOF) calculated for $C_{12}H_{24}O$ $[M+Na]^+$: 207.1719, found: 207.1721.

3,7-dimethyloctanal **61**

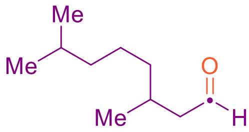

R_f = 0.3, PE/EA = 50:1 (v/v). Colorless oil, yield: 58%. **^{1}H NMR** (400 MHz, $CDCl_3$) δ 9.75 (t, J = 2.36 Hz, 1H), 2.39 (ddd, J = 2.13, 5.75, 16.01 Hz, 1H), 2.21 (ddd, J = 2.63, 7.85, 16.01 Hz, 1H), 2.09 – 1.98 (m, 1H), 1.58 – 1.46 (m, 1H), 1.35 – 1.20 (m, 4H), 1.19 – 1.10 (m, 2H), 0.95 (d, J = 6.73 Hz, 3H), 0.86 (d, J = 6.64 Hz, 6H). **^{13}C NMR** (101 MHz, $CDCl_3$) δ 203.27, 51.12, 39.00, 37.14, 28.19, 27.92, 24.69, 22.66, 22.58, 20.00. **HRMS** (ESI-TOF) calculated for $C_{10}H_{20}O$ $[M+Na]^+$: 179.1406, found: 179.1406.

methyl (1r,4r)-4-formylcyclohexane-1-carboxylate **62**

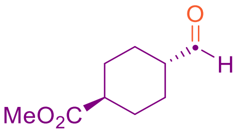

R_f = 0.3, PE/EA = 20:1 (v/v). Colorless oil, yield: 70%. **^{1}H NMR** (400 MHz, $CDCl_3$) δ 9.61 (d, J = 1.23 Hz, 1H), 3.65 (s, 3H), 2.31 – 2.16 (m, 2H), 2.10 – 2.02 (m, 4H), 1.52 – 1.41 (m, 2H), 1.36 – 1.20 (m, 2H). **^{13}C NMR** (101 MHz, $CDCl_3$) δ 203.93, 175.79, 51.68, 49.33, 42.50, 27.67, 25.02. **HRMS** (ESI-TOF) calculated for $C_9H_{14}O_3$ $[M+Na]^+$: 193.0835, found: 193.0835.

1-Adamantanecarbaldehyde **63**

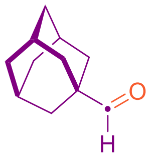

R_f = 0.3, PE/EA = 50:1 (v/v). White solid, yield: 53%. **^{1}H NMR** (400 MHz, $CDCl_3$) δ 9.31 (s, 1H), 2.13 – 2.00 (m, 3H), 1.81 – 1.66 (m, 12H). **^{13}C NMR** (101 MHz, $CDCl_3$) δ 206.06, 44.86, 36.57, 35.85, 27.36. **HRMS** (ESI-TOF) calculated for $C_{11}H_{16}O$ $[M+Na]^+$: 187.1093, found: 187.1095.

cyclohex-3-ene-1-carbaldehyde **64**

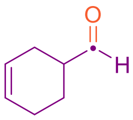

R_f = 0.3, PE/EA = 50:1 (v/v). Colorless oil, yield: 43%. **^{1}H NMR** (400 MHz, $CDCl_3$) δ 9.69 (d, J = 1.21 Hz, 1H), 5.74 – 5.65 (m, 2H), 2.58 – 2.47 (m, 1H), 2.26 – 2.19 (m, 2H), 2.15 – 2.06 (m, 2H), 2.04 – 1.95 (m, 1H), 1.72 – 1.61 (m, 1H). **^{13}C NMR** (101 MHz, $CDCl_3$) δ 204.54, 127.21, 124.76, 46.04, 24.28, 23.71, 22.07. **HRMS** (ESI-TOF) calculated for $C_7H_{11}O$ $[M+Na]^+$: 133.0624, found: 133.0625.

5-oxopentyl (3aS,4S,6R,6aR)-6-methoxy-2,2-dimethyltetrahydrofuro[3,4-d][1,3]dioxole-4-

carboxylate **65**

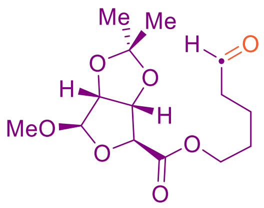

R_f= 0.3, PE/EA = 5:1 (v/v). Yellow oil, yield: 66%. **^{1}H NMR** (400 MHz, $CDCl_3$) δ 9.77 (t, J = 1.45 Hz, 1H), 5.19 (dd, J = 1.10, 5.85 Hz, 1H), 5.02 (s, 1H), 4.60 (s, 1H), 4.54 (d, J = 5.92 Hz, 1H), 4.24 – 4.09 (m, 2H), 3.38 (s, 3H), 2.50 (ddt, J = 2.18, 5.13, 6.74 Hz, 2H), 1.76 – 1.64 (m, 4H), 1.48 (s, 3H), 1.33 (s, 3H). **^{13}C NMR** (101 MHz, $CDCl_3$) δ 201.76, 170.14, 112.76, 109.37, 84.34, 83.69, 82.14, 64.86, 55.45, 43.27, 27.96, 26.39, 25.04, 18.43. **HRMS** (ESI-TOF) calculated for $C_{14}H_{22}O_7$ $[M+Na]^+$: 325.1258, found: 325.1257.

trimethyl(p-tolyl)silane **66**

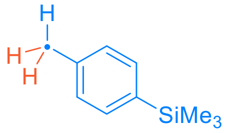

R_f= 0.9, PE/EA = 100:0 (v/v). Colorless oil, yield: 80%. **^{1}H NMR** (400 MHz, $CDCl_3$) δ δ 7.44 (d, J = 8.01 Hz, 2H), 7.20 (d, J = 7.37 Hz, 2H), 2.37 (s, 3H), 0.27 (s, 9H). **^{13}C NMR** (101 MHz, $CDCl_3$) δ 138.65, 136.85, 133.40, 128.62, 21.47, -1.01. **HRMS** (ESI-TOF) calculated for: $C_{10}H_{16}Si$ $[M-H]^-$: 163.0949, found: 163.0939.

cyclohexanone **67**

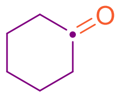

GC yield: 83%. **HRMS** (ESI-TOF) calculated for: $C_6H_{10}O$ $[M+Na]^+$: 121.0624, found: 121.0626.

mesitylene **68**

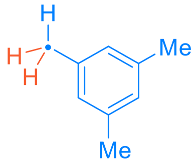

GC yield: 88%. **HRMS** (ESI-TOF) calculated for: $C_9H_{12}[M+H]^+$: 121.1012, found: 121.1014.

decan-2-one **69**

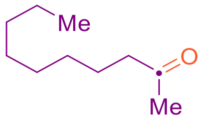

R_f= 0.3, PE/EA = 50:1 (v/v). Colorless oil, yield: 90%.**^{1}H NMR** (400 MHz, $CDCl_3$) δ 2.40 (t, J = 7.49 Hz, 2H), 2.12 (s, 3H), 1.60 – 1.49 (m, 2H), 1.32 – 1.19 (m, 10H), 0.92 – 0.82 (m, 3H). **^{13}C NMR** (101 MHz, $CDCl_3$) δ 209.39, 43.82, 31.81, 29.83, 29.36, 29.18, 29.13, 23.87, 22.64, 14.08. **HRMS** (ESI-TOF) calculated for: $C_{10}H_{20}O[M+H]^+$: 157.1587, found: 157.1588.

1-chloro-4-methylbenzene **70**

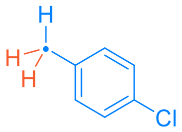

GC yield: 50%. **HRMS** (ESI-TOF) calculated for: C_7H_7Cl $[M+H]^+$: 127.0309, found: 127.0311.

(1's,4'r)-4'-propyl-[1,1'-bi(cyclohexan)]-4-one **71**

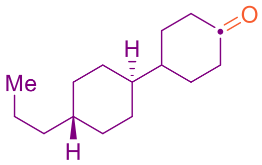

R_f = 0.4, PE/EA = 50:1 (v/v). White solid, yield: 65%. **^{1}H NMR** (400 MHz, $CDCl_3$) δ 2.41 – 2.23 (m, 4H), 2.07 – 1.95 (m, 2H), 1.80 – 1.69 (m, 4H), 1.57 – 1.40 (m, 3H), 1.33 – 1.24 (m, 2H), 1.22 – 1.08 (m, 4H), 1.06 – 0.95 (m, 2H), 0.91 – 0.79 (m, 5H). **^{13}C NMR** (101 MHz, $CDCl_3$) δ 212.72, 42.02, 41.71, 41.17, 39.69, 37.43, 33.39, 30.22, 29.87, 20.02, 14.41. **HRMS** (ESI-TOF) calculated for: $C_{15}H_{26}O[M+H]^+$: 223.2056, found: 223.2056.

methyl 4'-(methyl-d2)-[1,1'-biphenyl]-4-carboxylate **72**

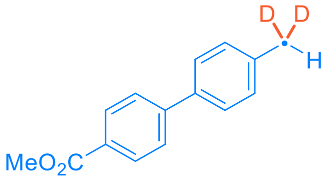

R_f = 0.3, PE/EA = 50:1 (v/v). White solid, yield: 95%. **^{1}H NMR** (400 MHz, $CDCl_3$) δ 8.10 (d, J = 8.48 Hz, 2H), 7.65 (d, J = 8.44 Hz, 2H), 7.53 (d, J = 8.23 Hz, 2H), 7.28 (d, J = 8.10 Hz, 2H), 3.94 (s, 3H), 2.41 – 2.37 (m, 1H). **^{13}C NMR** (101 MHz, $CDCl_3$) δ 167.07, 145.59, 138.06, 137.11, 130.10, 129.68, 128.61, 127.12, 126.81, 52.11, 21.10-20.44. **HRMS** (ESI-TOF) calculated for: $C_{15}H_{12}D_2O_2$ $[M+H]^+$: 229.1192, found: 229.1194.

2-(methyl-d2)benzo[d]thiazole **73**

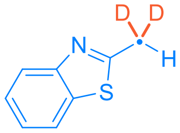

R_f = 0.3, PE/EA = 50:1 (v/v). Colorless oil, yield: 77%. **^{1}H NMR** (400 MHz, $CDCl_3$) δ 7.95 (d, J = 8.15 Hz, 1H), 7.82 (d, J = 8.11 Hz, 1H), 7.48 – 7.40 (m, 1H), 7.37 – 7.31 (m, 1H), 2.85 – 2.79 (m, 1H). **^{13}C NMR** (101 MHz, $CDCl_3$) δ 166.94, 153.39, 135.64, 125.93, 124.71, 122.40, 121.40, 19.84-19.44. **HRMS** (ESI-TOF) calculated for $C_8H_5D_2NS$ $[M+H]^+$: 152.0497, found: 152.0497.

4-(methyl-d2)phenyl 4-(N,N-dipropylsulfamoyl)benzoate **74**

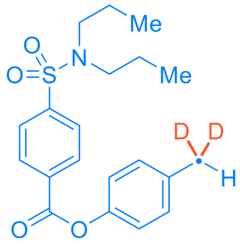

R_f = 0.3, PE/EA = 10:1 (v/v). White solid, yield: 65%. **^{1}H NMR** (400 MHz, $CDCl_3$) δ 8.31 (d, J = 8.48 Hz, 2H), 7.94 (d, J = 8.49 Hz, 2H), 7.23 (d, J = 8.41 Hz, 2H), 7.10 (d, J = 8.48 Hz, 2H), 3.16 – 3.09 (m, 4H), 2.39 – 2.34 (m, 2H), 1.61 – 1.51 (m, 4H), 0.93 – 0.85 (m, 6H). **^{13}C NMR** (101 MHz, $CDCl_3$) δ 164.09, 148.41, 144.80, 135.93, 135.90, 132.98, 130.79, 130.14, 127.15, 121.16, 49.95, 21.95, 20.94-20.39, 11.19. **HRMS** (ESI-TOF) calculated for $C_{20}H_{23}D_2NO_4S$ $[M+H]^+$: 378.1703, found: 378.1700.

(1R,2S,4S)-2-isopropyl-4-methylcyclohexyl 4'-(methyl-d2)-[1,1'-biphenyl]-4-carboxylate **75**

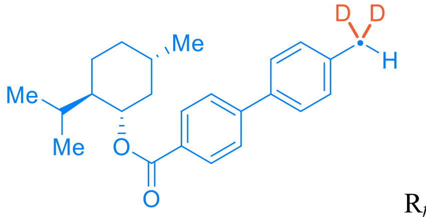

R_f = 0.3, PE/EA = 50:1 (v/v). White solid, yield: 86%. **^{1}H NMR** (400 MHz, $CDCl_3$) δ 8.11 (d, J = 8.41 Hz, 2H), 7.65 (d, J = 8.44 Hz, 2H), 7.53 (d, J = 8.19 Hz, 2H), 7.28 (d, J = 8.10 Hz, 2H), 5.02 – 4.92 (m, 1H), 2.42 – 2.37 (m, 1H), 2.16 (d, J = 12.06 Hz, 1H), 2.06 – 1.96 (m, 1H), 1.80 – 1.71 (m, 2H), 1.64 – 1.56 (m, 2H), 1.20 – 1.07 (m, 2H), 0.95 (dd, J = 3.06, 6.82 Hz, 7H), 0.82 (d, J = 6.98 Hz, 3H). **^{13}C NMR** (101 MHz, $CDCl_3$) δ 166.06, 145.43, 138.03, 137.99, 137.25, 130.08, 129.68, 129.32, 127.14, 126.79, 74.81, 47.33, 41.03, 34.37, 31.49, 26.55, 23.68, 22.10, 21.11, 20.91-20.45, 16.58. **HRMS** (ESI-TOF) calculated for $C_{24}H_{28}D_2O_2$ $[M+Na]^+$: 375.2264, found: 375.2263.

((3aS,5aR,8aR,8bS)-2,2,7,7-tetramethylhexahydro-3aH-[1,3]dioxolo[4,5-b]furo[2,3-d]pyran-3a-yl)methyl 4'-(methyl-d2)-[1,1'-biphenyl]-2-carboxylate **76**

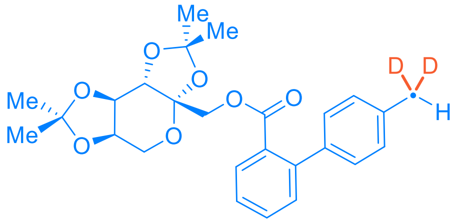

R_f = 0.3, PE/EA = 5:1 (v/v). White solid, yield: 77%. **^{1}H NMR** (400 MHz, $CDCl_3$) δ 7.80 (dd, J = 1.50, 8.01 Hz, 1H), 7.51 (td, J = 1.44, 7.50 Hz, 1H), 7.43 – 7.34 (m, 2H), 7.23 (s, 4H), 4.43 – 4.33 (m, 2H), 4.16 (dd, J = 1.77, 7.83 Hz, 1H), 3.89 (d, J = 11.60 Hz, 1H), 3.83 (dd, J = 1.94, 12.98 Hz, 1H), 3.69 (d, J = 12.98 Hz, 1H), 3.33 (d, J = 2.69 Hz, 1H), 2.42 – 2.34 (m, 2H), 1.43 (d, J = 6.80 Hz, 6H), 1.35 (s, 3H), 1.12 (s, 3H). **^{13}C NMR** (101 MHz, $CDCl_3$) δ 168.65, 141.92, 138.11, 136.98, 136.94, 131.29, 130.97, 130.48, 129.95, 129.37, 128.19, 126.96, 108.93, 108.66, 101.38, 70.85, 69.96, 69.89, 65.61, 61.31, 26.50, 25.94, 25.30, 24.22, 21.20, 21.11, 20.92-20.45. **HRMS** (ESI-TOF) calculated for $C_{26}H_{28}D_2O_7$ $[M+H]^+$: 457.2190, found: 457.2188.

1-methyl 4-(4-(methyl-d2)phenyl) (1s,2R,3s,4r,5S,6r,7R,8S)-cubane-1,4-dicarboxylate **77**

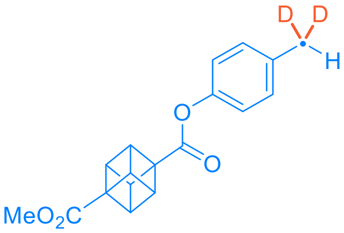

R_f = 0.3, PE/EA = 30:1 (v/v). White solid, yield: 54%. **^{1}H NMR** (400 MHz, $CDCl_3$) δ 7.17 (d, J = 8.40 Hz, 2H), 6.97 (d, J = 8.45 Hz, 2H), 4.41 – 4.35 (m, 3H), 4.34 – 4.29 (m, 3H), 3.73 (s, 3H), 2.36 – 2.30 (m, 2H). **^{13}C NMR** (101 MHz, $CDCl_3$) δ 171.91, 170.08, 148.29, 135.45, 129.97, 121.18, 55.92, 55.83, 51.68, 47.27, 47.21, 20.87-20.33. **HRMS** (ESI-TOF) calculated for $C_{18}H_{14}D_2O_4$ $[M+Na]^+$: 321.1066, found: 321.1066.

undec-10-enal **79**

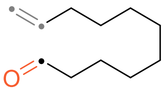

R_f = 0.3, PE/EA = 50:1 (v/v). Colorless oil, yield: 56%. **^{1}H NMR** (400 MHz, $CDCl_3$) δ 9.76 (s, 1H), 5.90 – 5.69 (m, 1H), 5.12 – 4.84 (m, 2H), 2.41 (td, J = 1.89, 7.34 Hz, 2H), 2.07 – 1.98 (m, 2H), 1.67 – 1.57 (m, 2H), 1.39 – 1.25 (m, 10H). **^{13}C NMR** (101 MHz, $CDCl_3$) δ 202.97, 139.16, 114.18, 43.92, 33.78, 29.30, 29.26, 29.14, 29.04, 28.88, 22.07. **HRMS** (ESI-TOF) calculated for $C_{11}H_{20}O$ $[M+Na]^+$: 191.1406, found: 191.1408.

5. **Experiment for KIE**

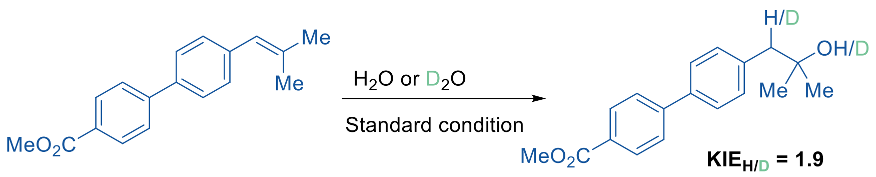

Supplementary Table 5. The results of KIE

T/min	Yield (%) of H_2O	Yield (%) of D_2O
0	0	0
5	8	2
10	22	6
20	29	18
30	39	21

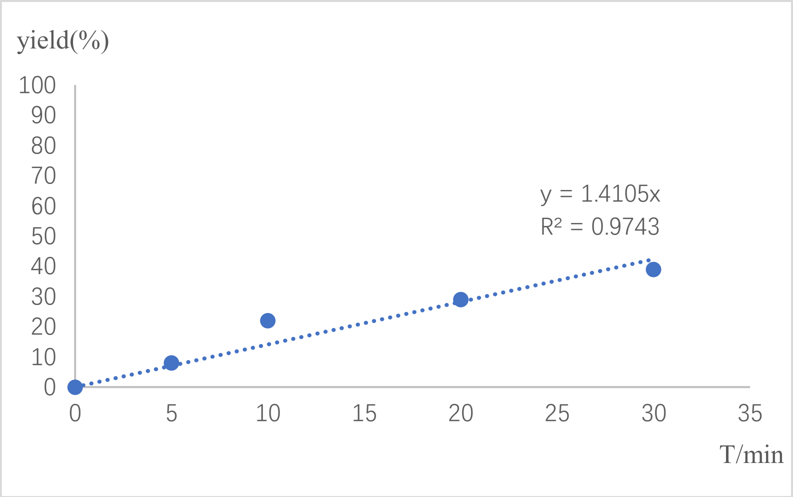

Supplementary Figure 3. experiments of H_2O

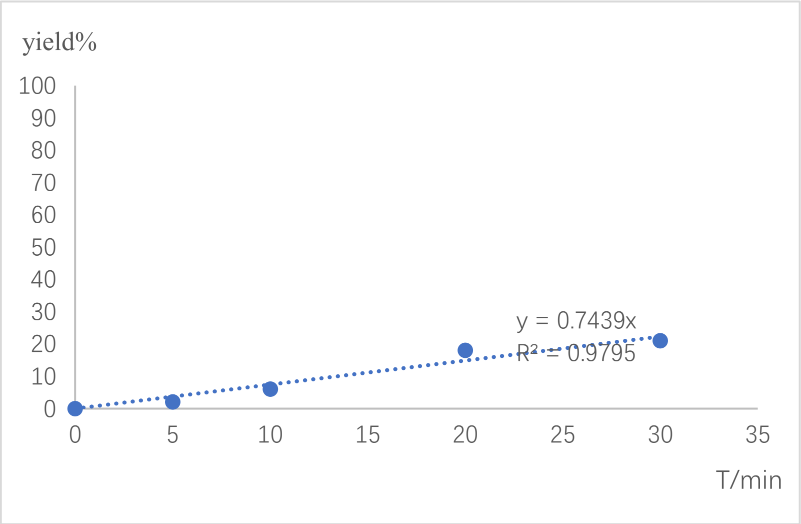

Supplementary Figure 4. experiments of D_2O

6. ^{18}O labeling experiment

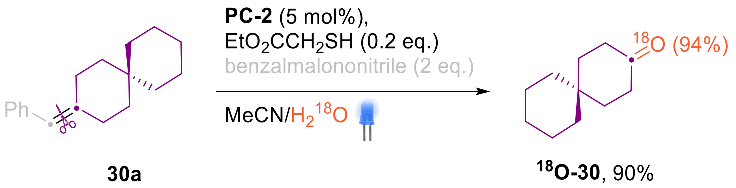

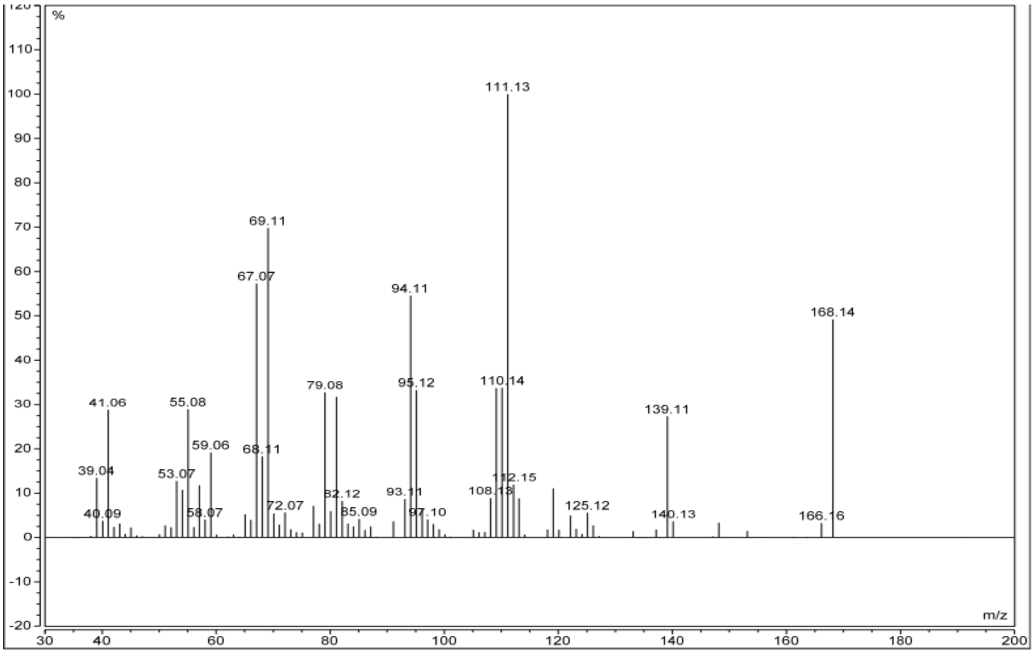

Supplementary Figure 5. Mass spectrometry data

7. Plausible mechanisms

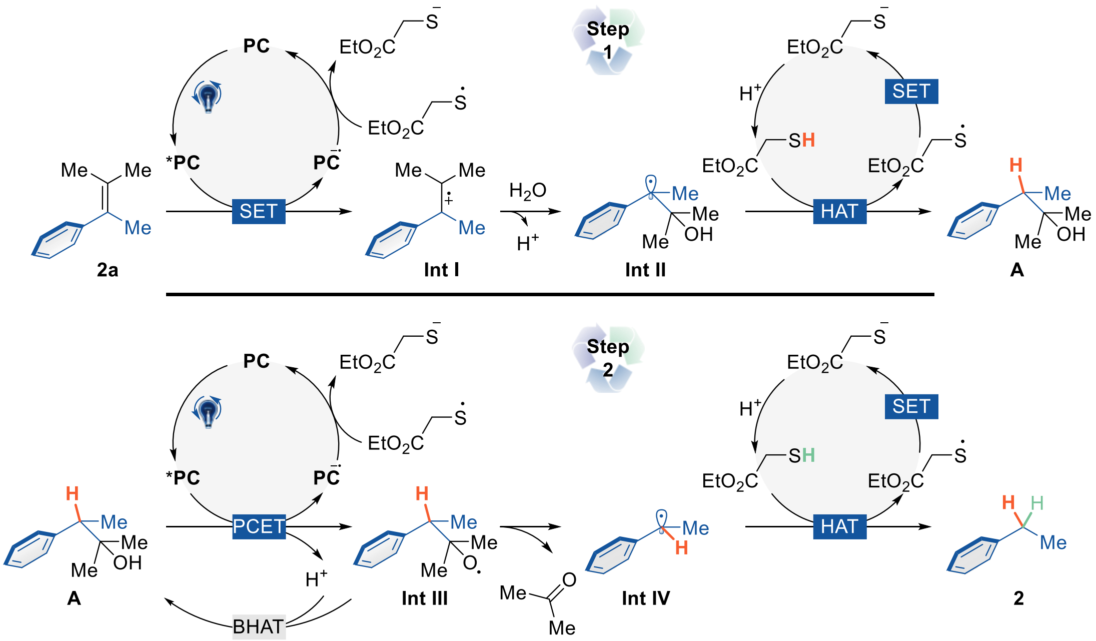

Supplementary Figure 6. Catalytic cycle

8. ^{1}H NMR and ^{13}C NMR spectra

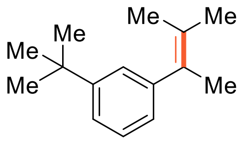

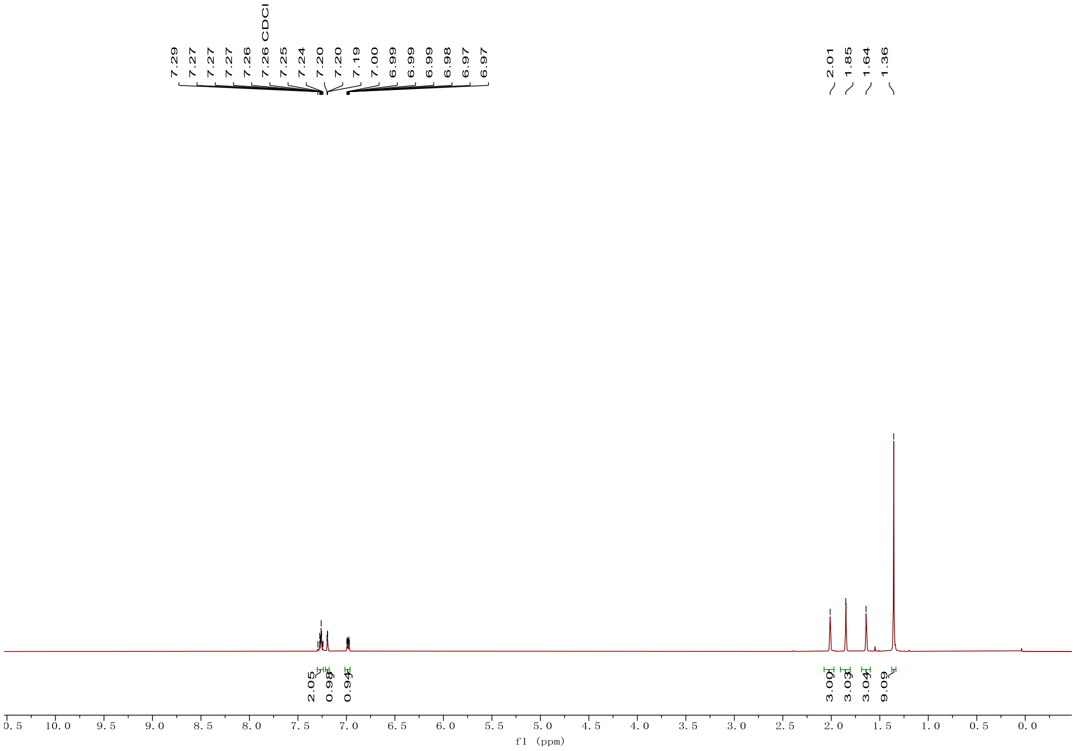

Supplementary Figure 7. **^{1}H NMR** (400 MHz, Chloroform-*d*) of **3a**

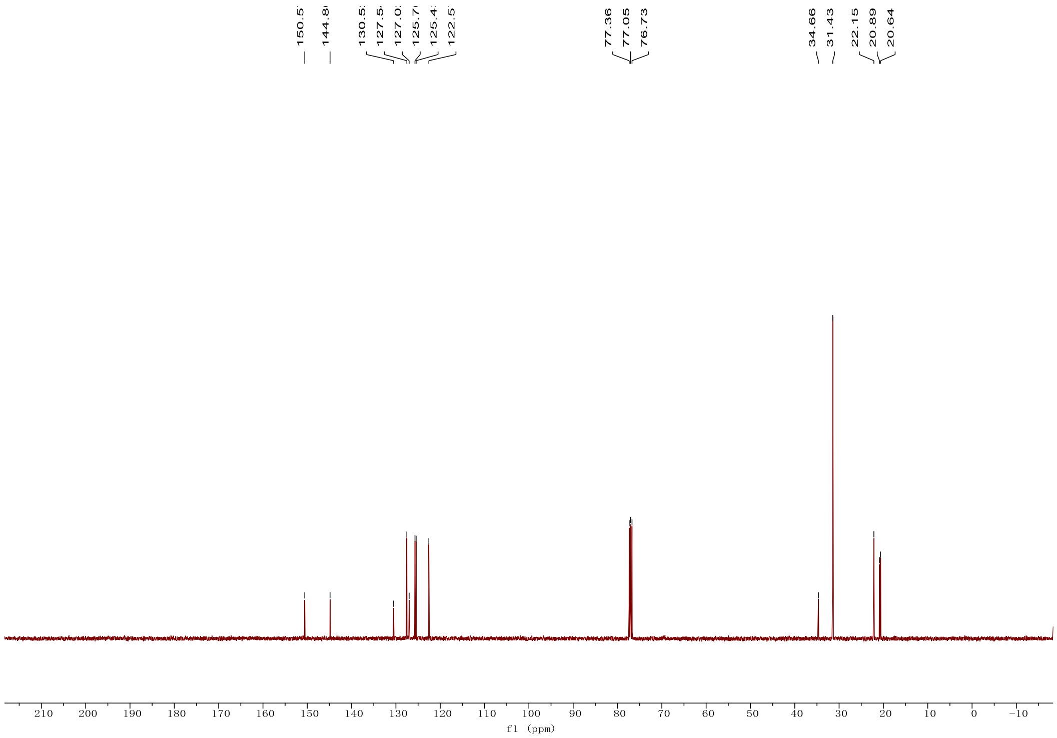

Supplementary Figure 8. ^{13}C NMR (101 MHz, Chloroform-*d*) of **3a**

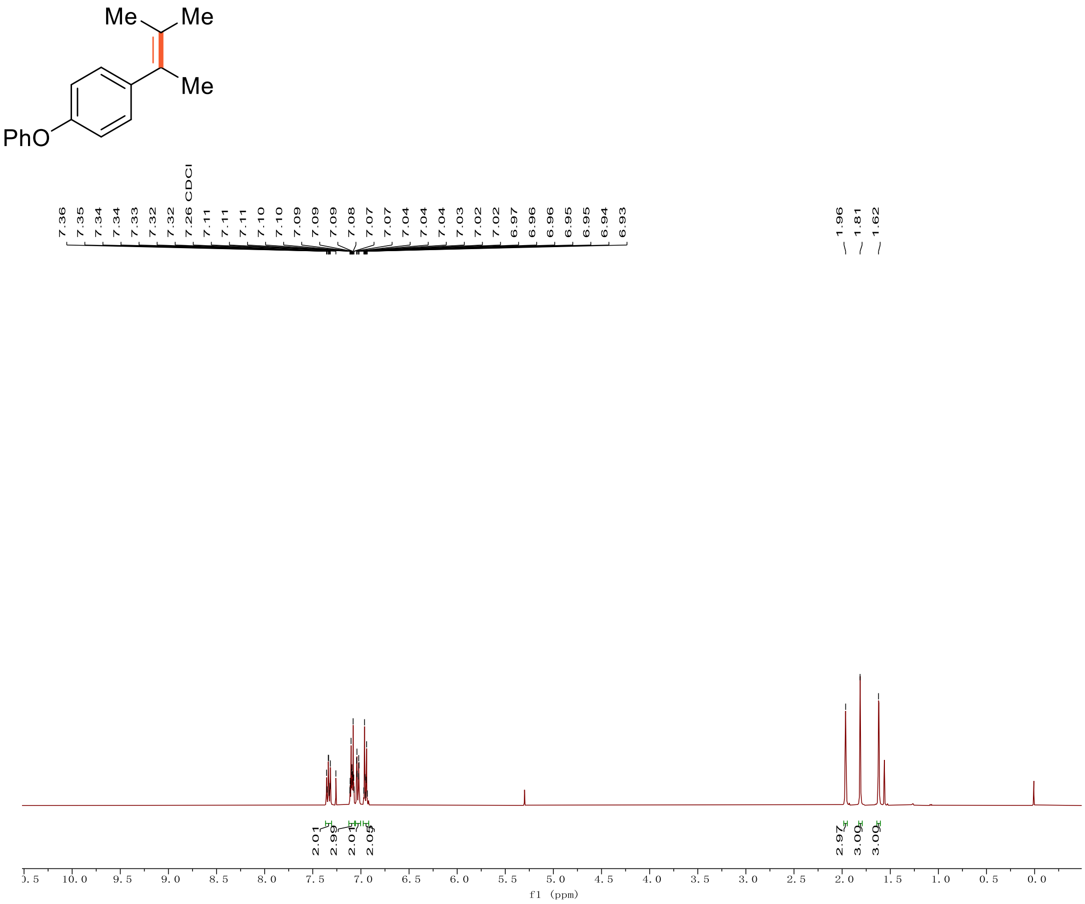

Supplementary Figure 9. ^{1}H NMR (400 MHz, Chloroform-*d*) of **4a**

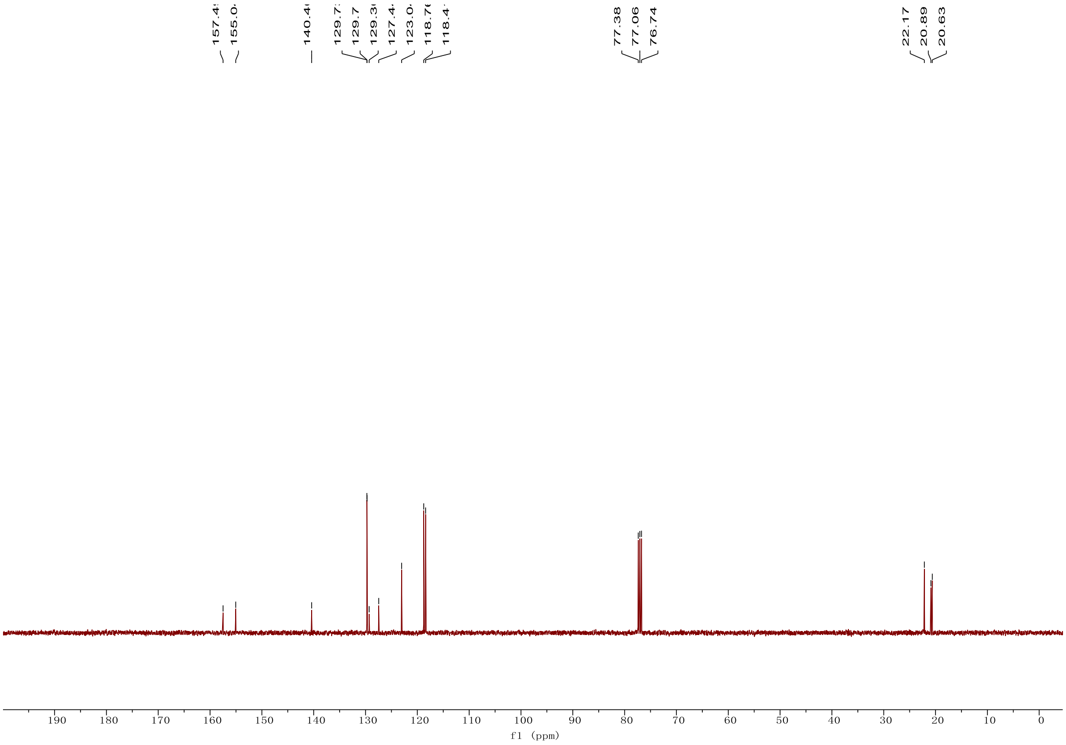

Supplementary Figure 10. ^{13}C NMR (101 MHz, Chloroform-*d*) of **4a**

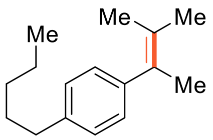

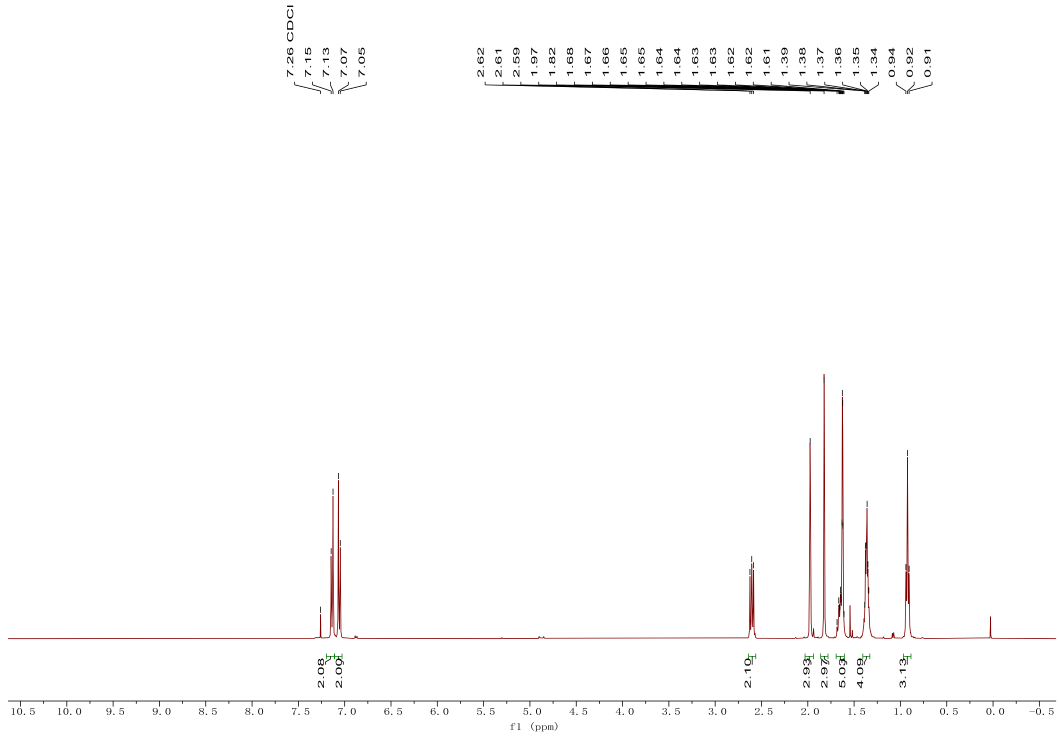

Supplementary Figure 11. ^{1}**H NMR** (400 MHz, Chloroform-*d*) of **5a**

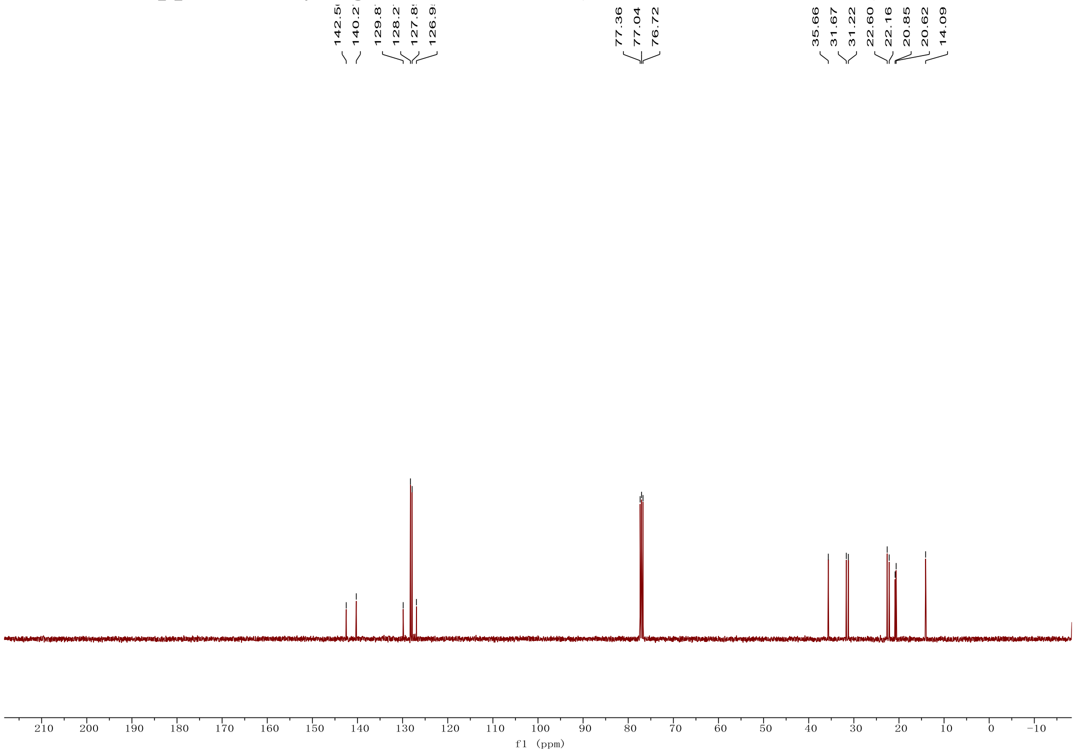

Supplementary Figure 12. ^{13}**C NMR (**101 MHz, Chloroform-*d*) of **5a**

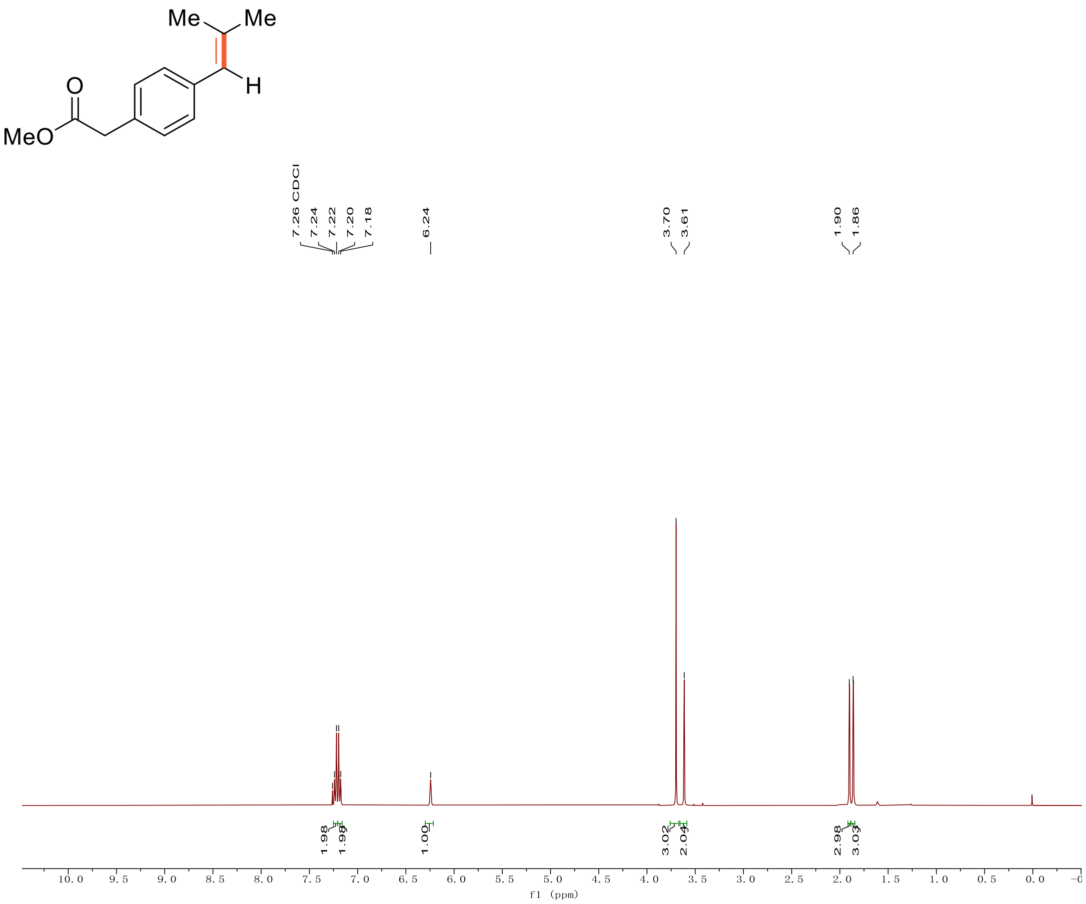

Supplementary Figure 13. ^{1}H NMR (400 MHz, Chloroform-*d*) of **8a**

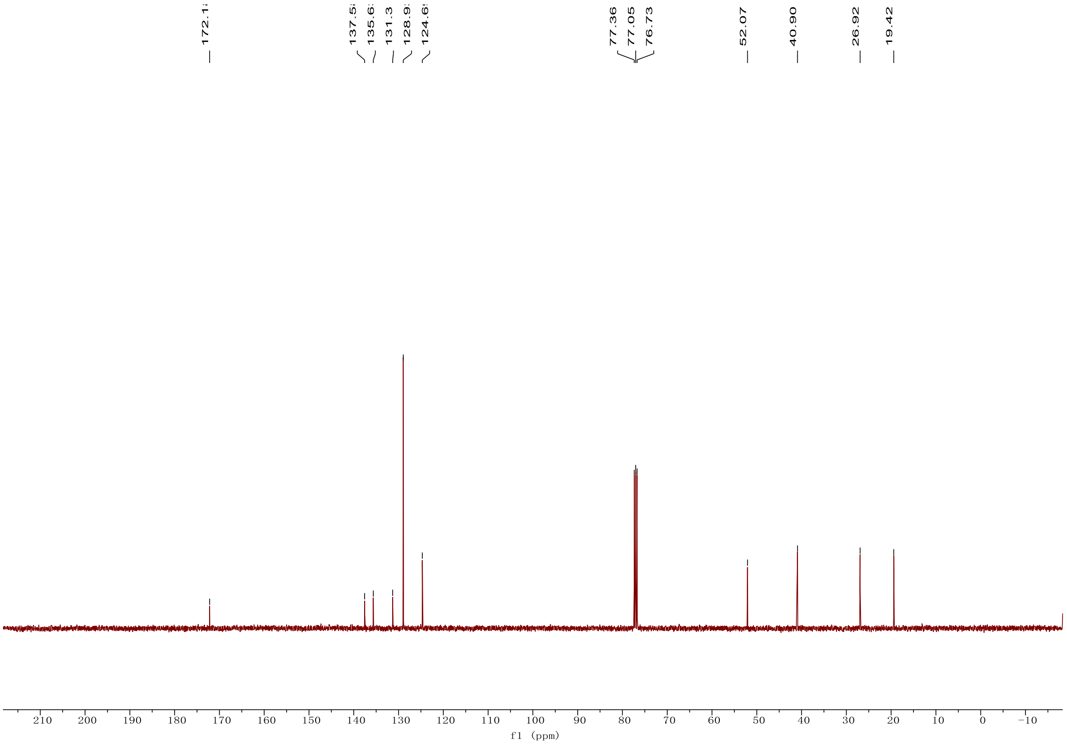

Supplementary Figure 14. ^{13}C NMR (101 MHz, Chloroform-*d*) of **8a**

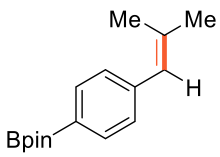

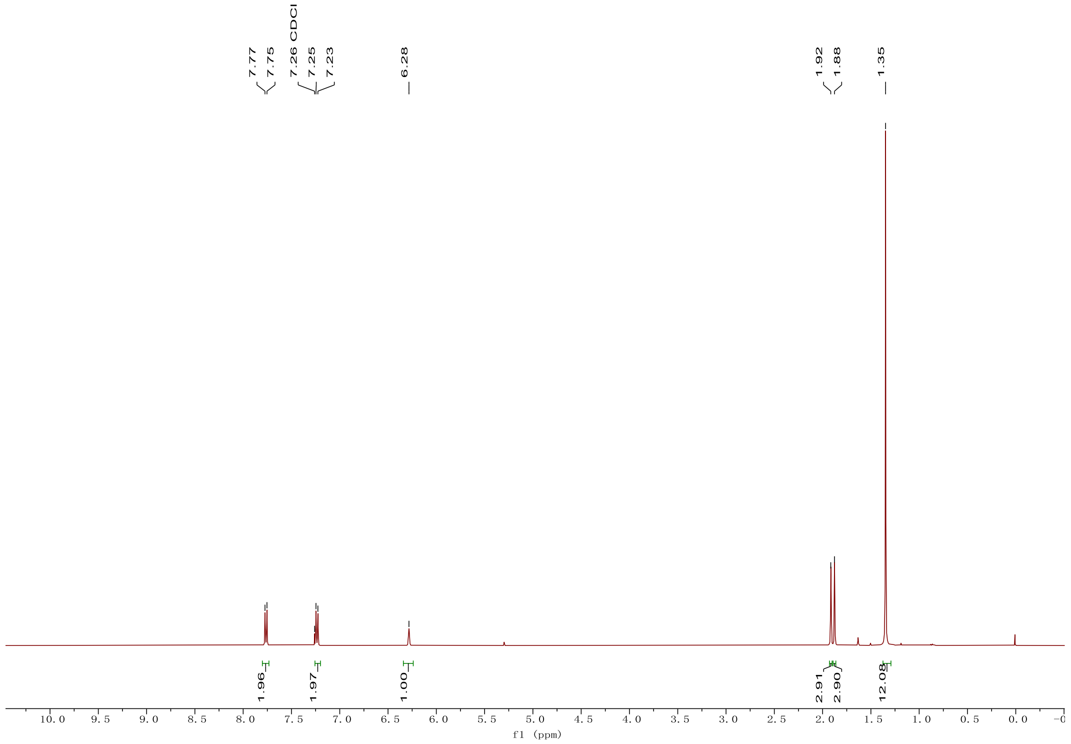

Supplementary Figure 15. ^{1}H NMR (400 MHz, Chloroform-*d*) of **11a**

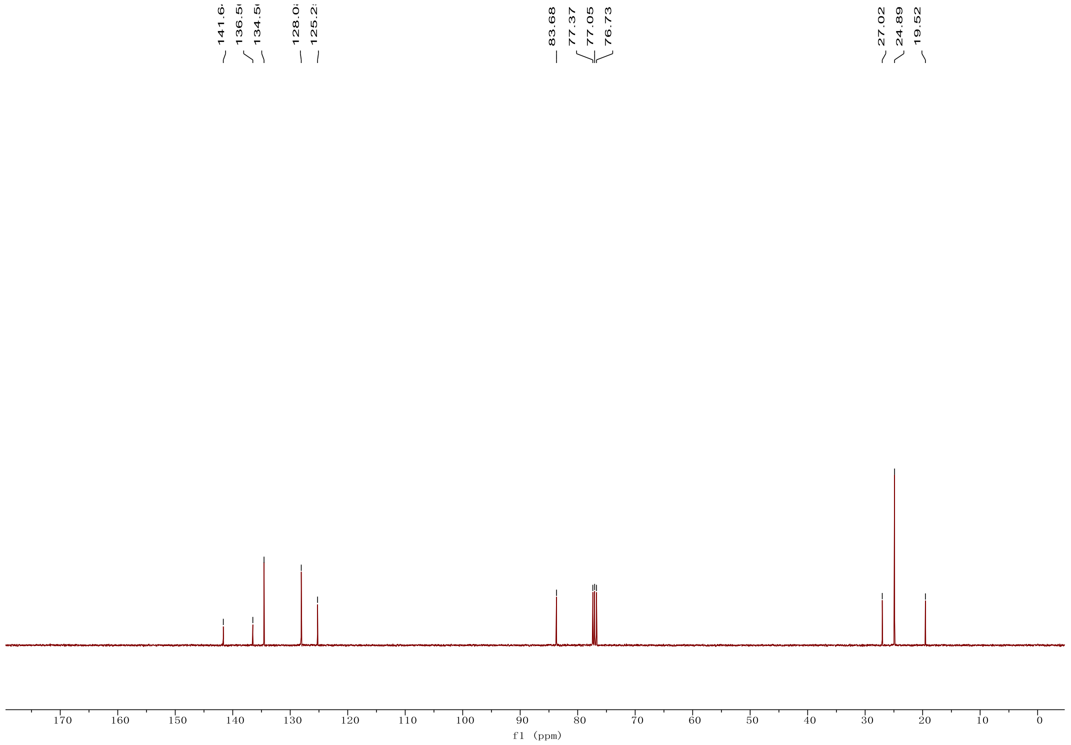

Supplementary Figure 16. ^{13}C NMR (101 MHz, Chloroform-*d*) of **11a**

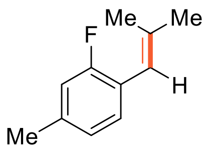

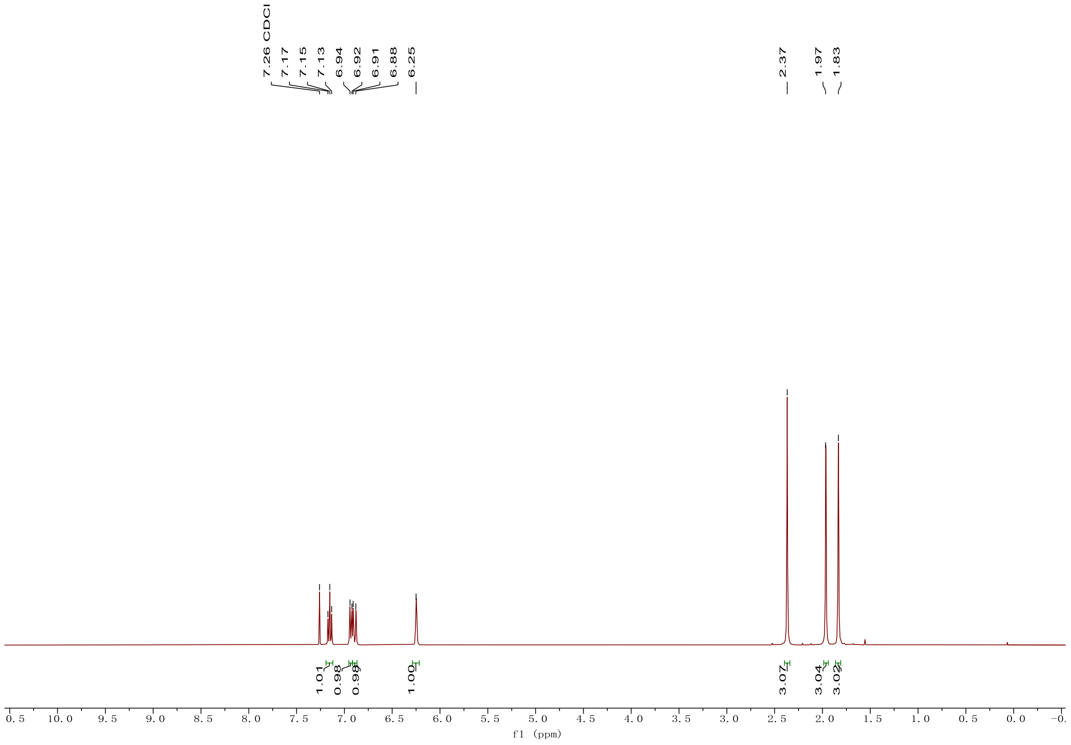

Supplementary Figure 17. ^{1}**H NMR** (400 MHz, Chloroform-*d*) of **12a**

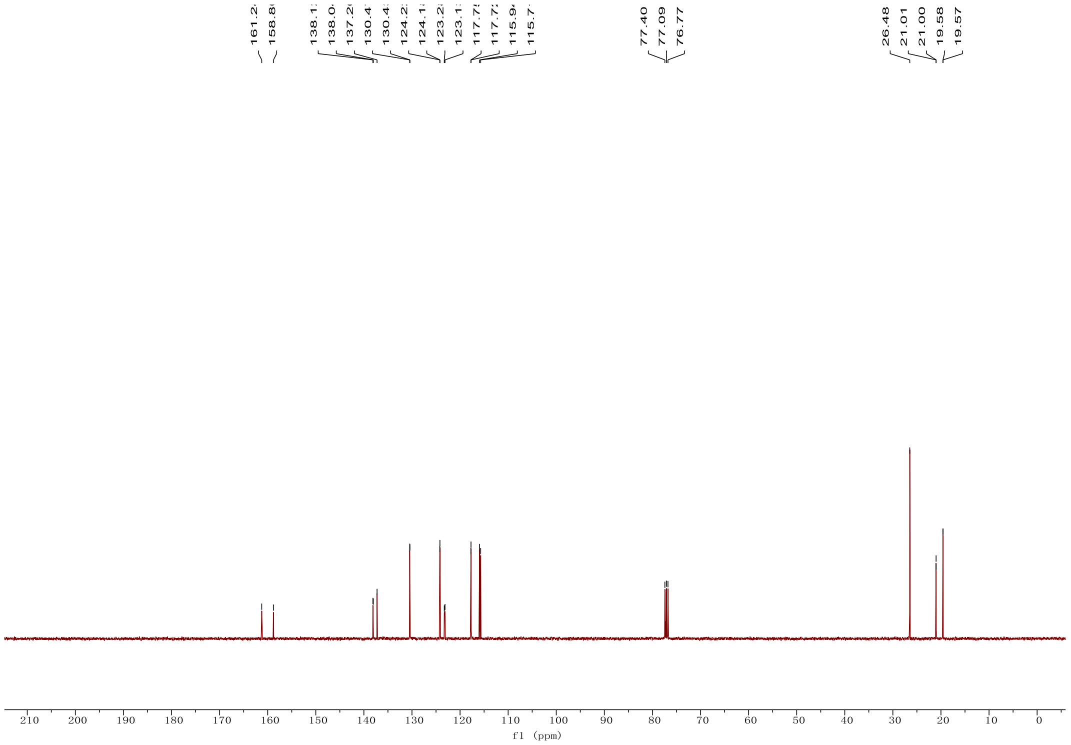

Supplementary Figure 18. ^{13}**C NMR (**101 MHz, Chloroform-*d*) of **12a**

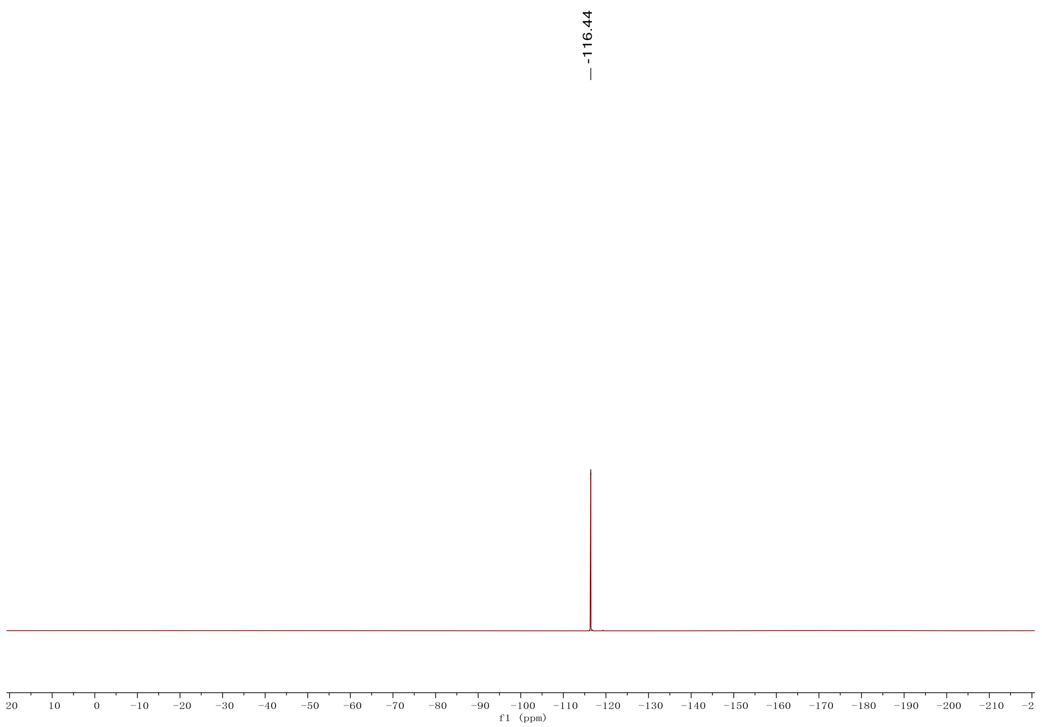

Supplementary Figure 19. ^{19}F NMR (376 MHz, Chloroform-*d*) of **12a**

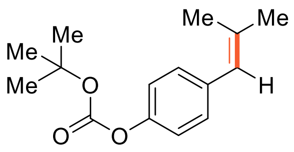

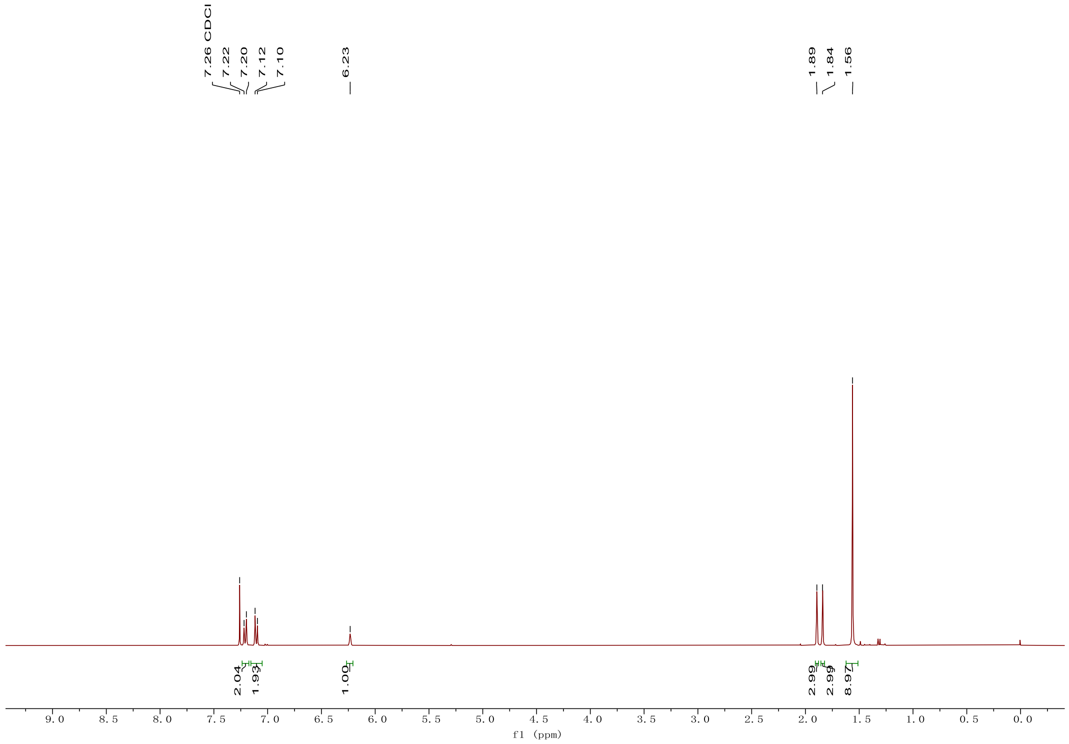

Supplementary Figure 20. **^{1}H NMR** (400 MHz, Chloroform-*d*) of **13a**

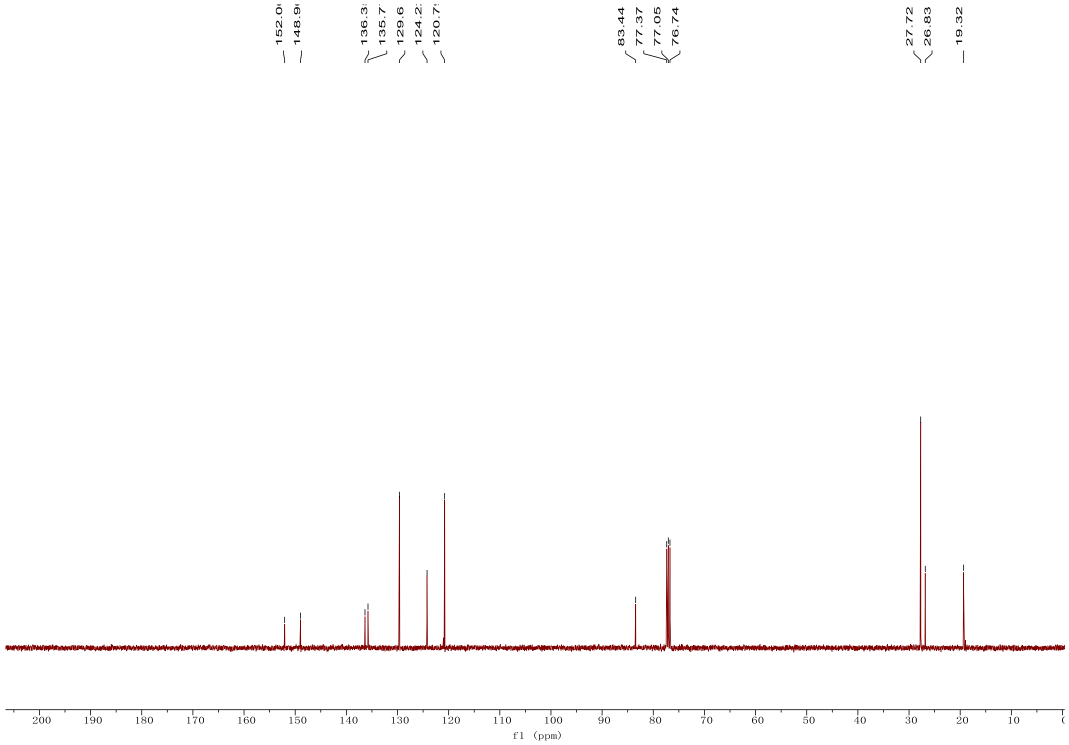

Supplementary Figure 21. ^{13}C NMR (101 MHz, Chloroform-*d*) of **13a**

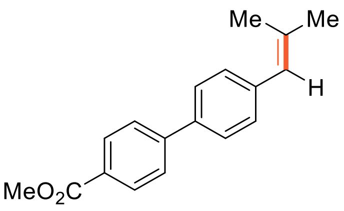

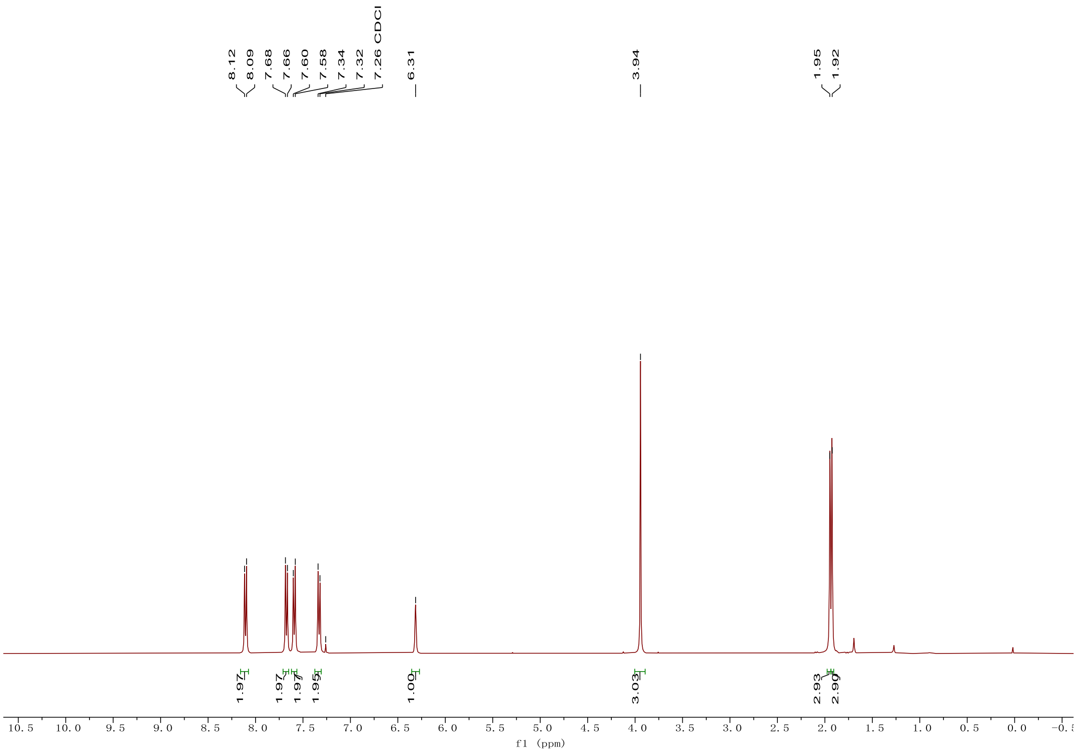

Supplementary Figure 22. **^{1}H NMR** (400 MHz, Chloroform-*d*) of **14a**

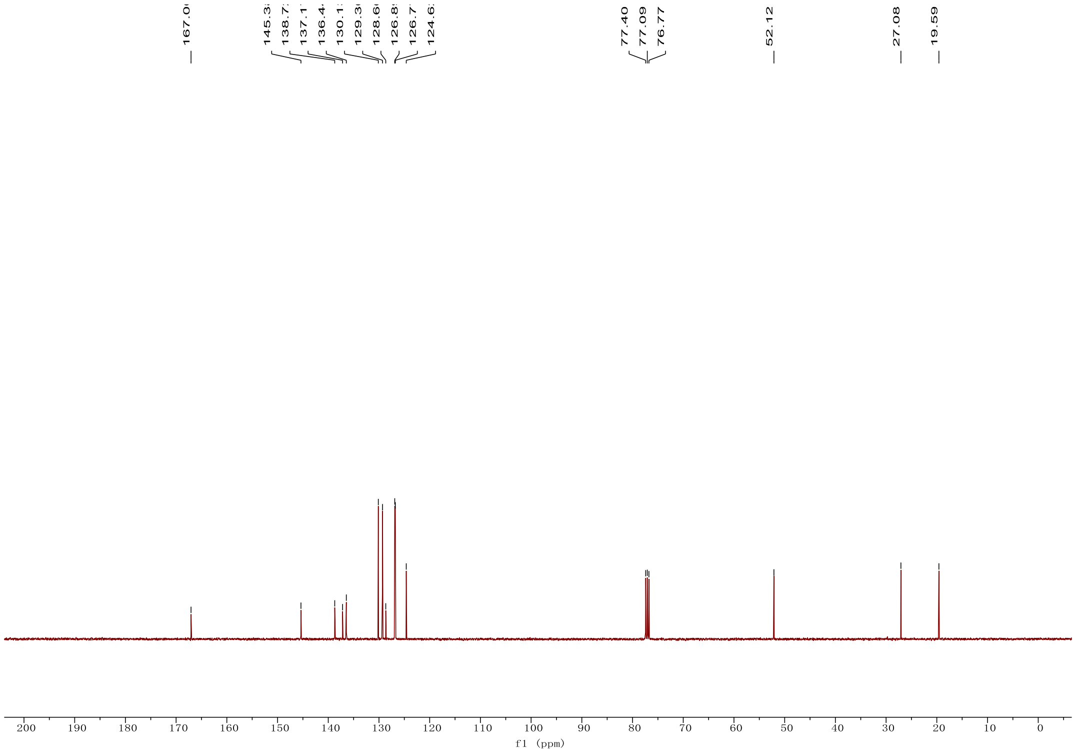

Supplementary Figure 23. **^{13}C NMR (**101 MHz, Chloroform-*d*) of **14a**

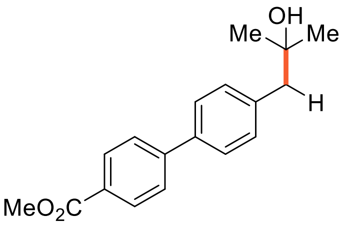

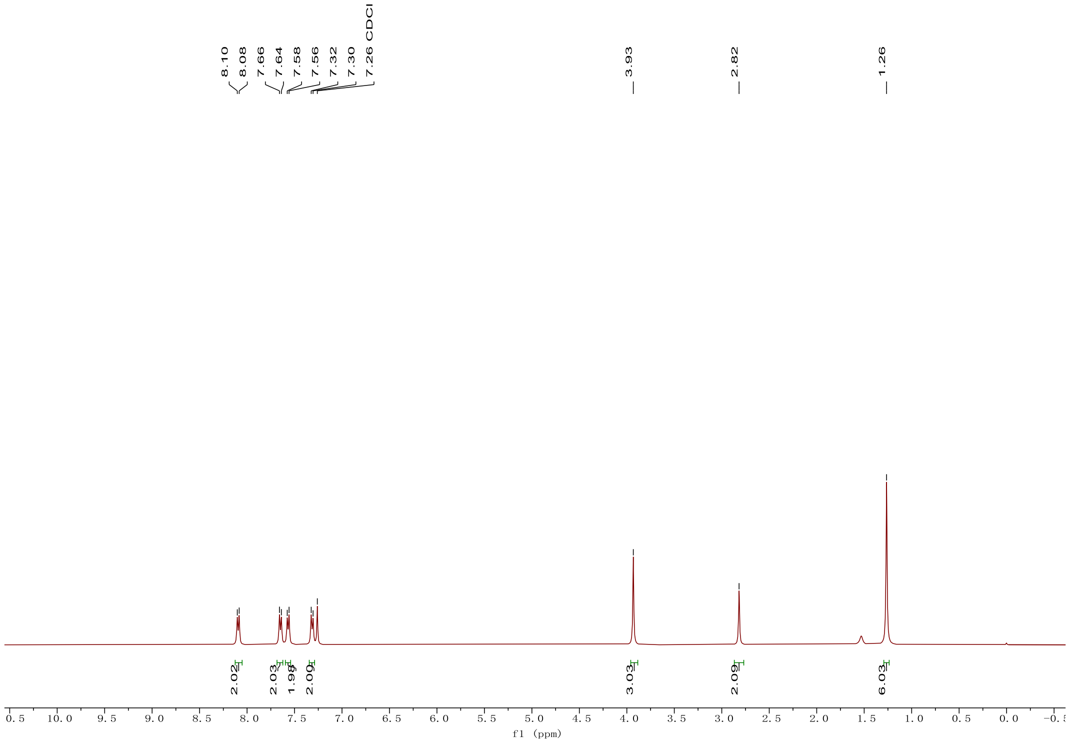

Supplementary Figure 24. **^{1}H NMR** (400 MHz, Chloroform-*d*) of **14a-OH**

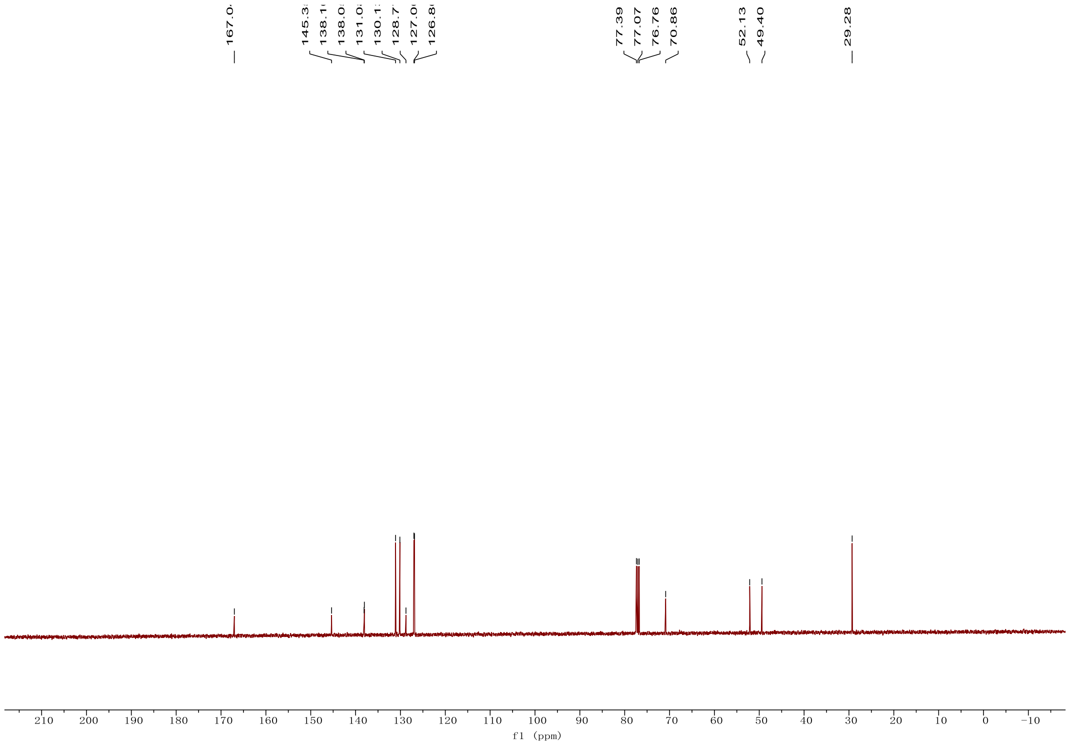

Supplementary Figure 25. **^{13}C NMR (**101 MHz, Chloroform-*d*) of **14a-OH**

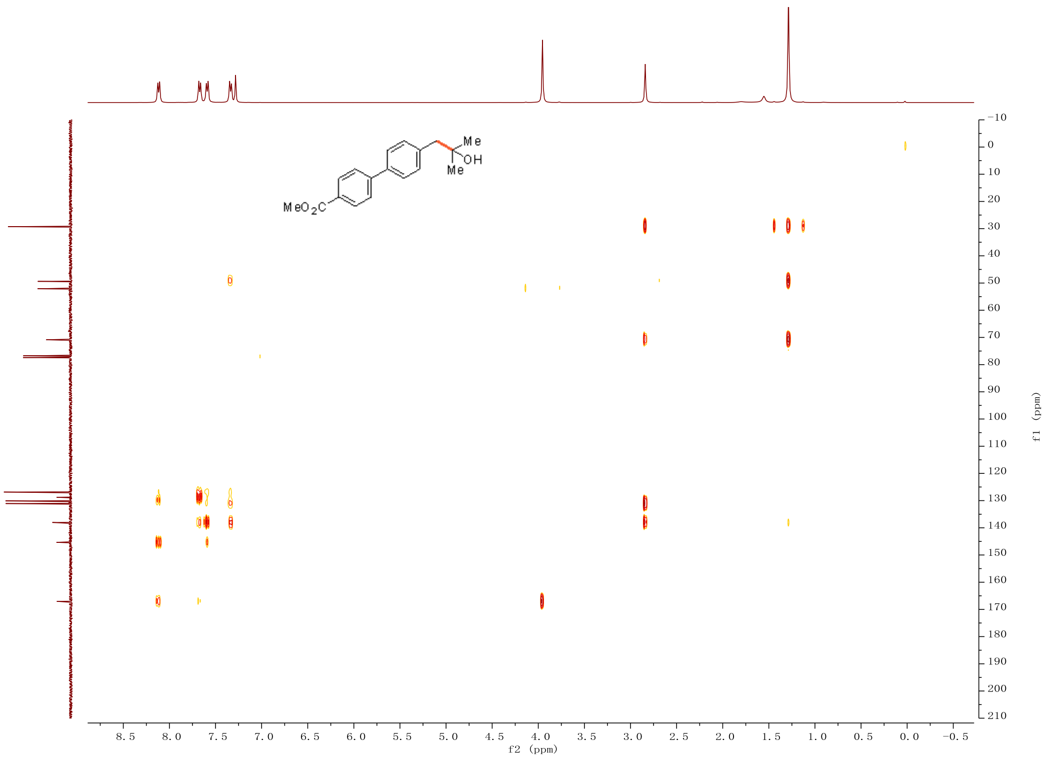

Supplementary Figure 26. HMBC spectrum of **14a-OH** in $CDCl_3$

Comparison of [1]**H NMR** of **14a-OH** at different dilutions to identify the peak at 1.55 ppm.

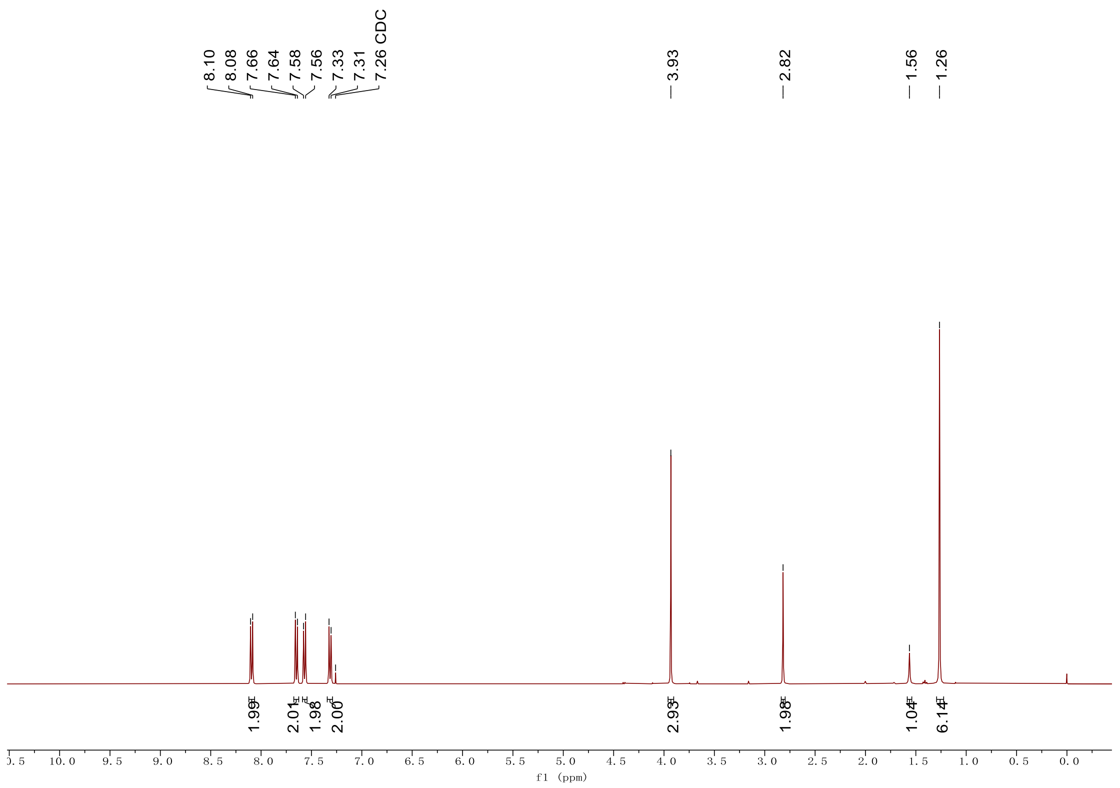

Supplementary Figure 27. ^{1}**H NMR** spectrum of the undiluted **14a-OH**.

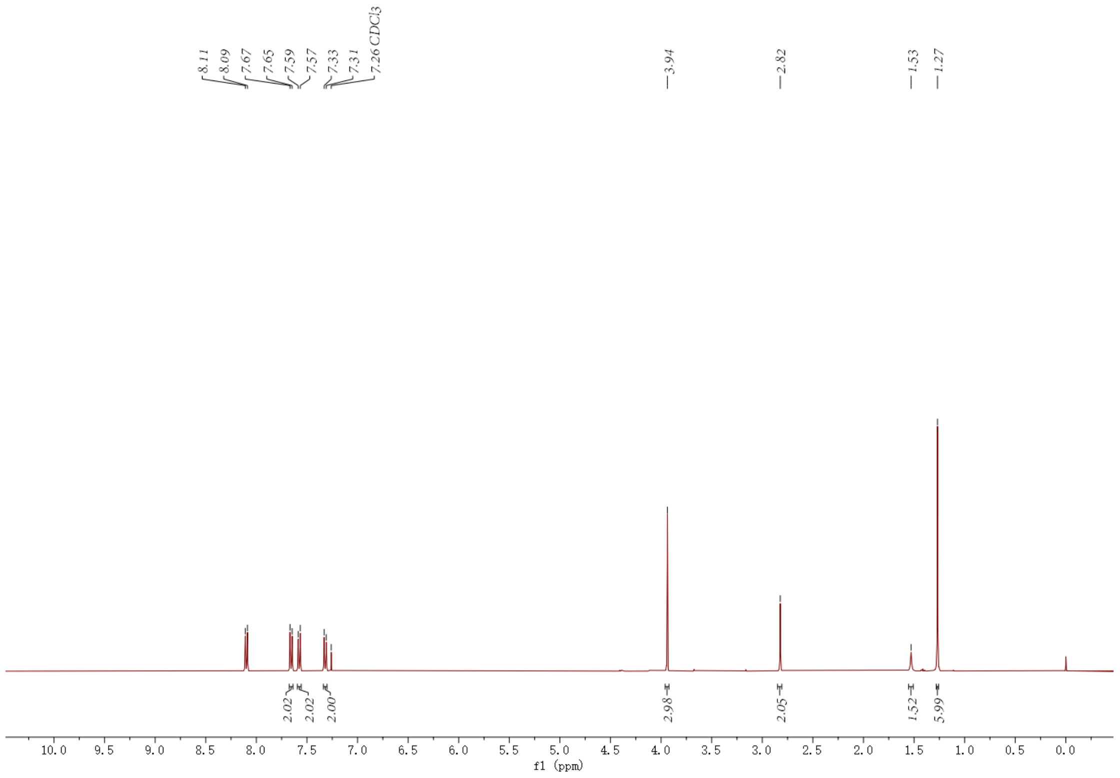

Supplementary Figure 28. ^{1}**H NMR** spectrum of the **14a-OH** after first dilution.

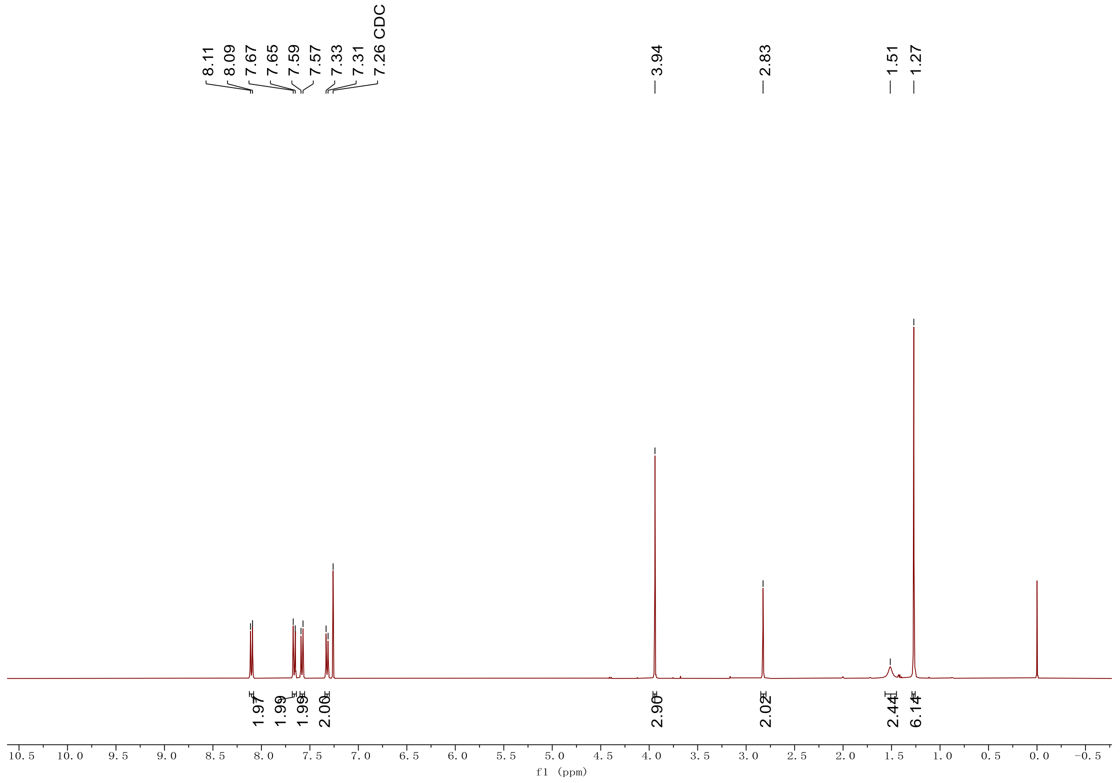

Supplementary Figure 29. ^{1}**H NMR** spectrum of the **14a-OH** after second dilution.

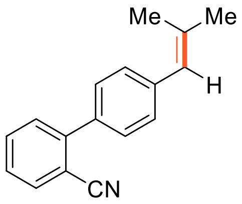

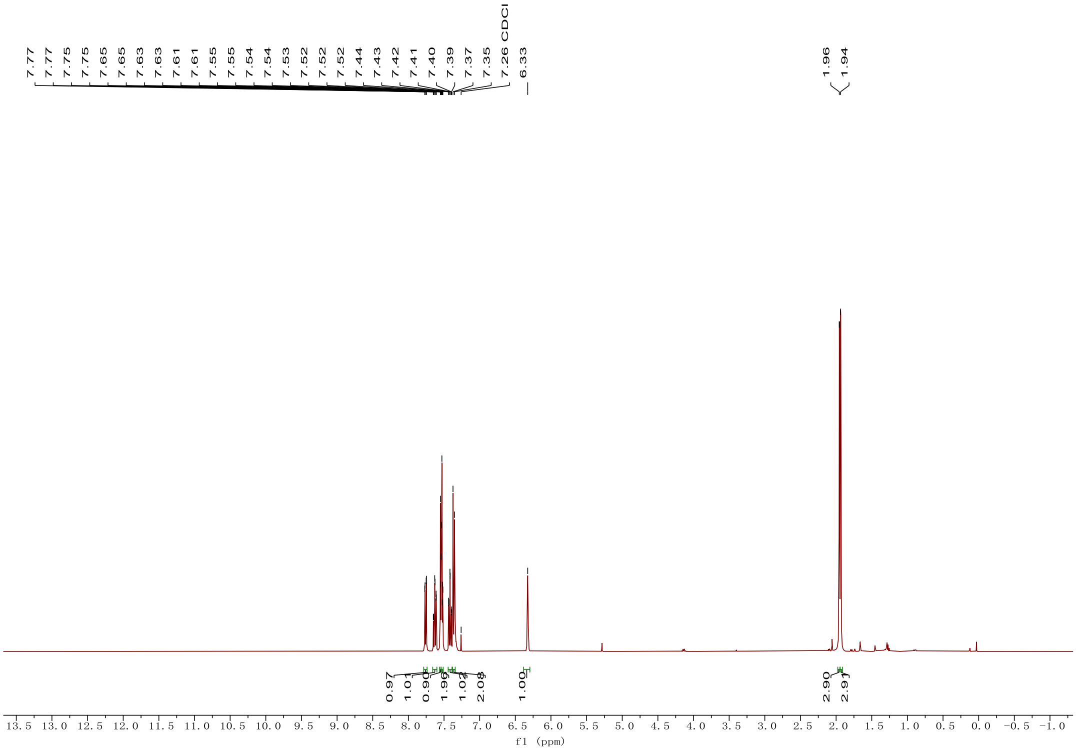

Supplementary Figure 30. ¹H NMR (400 MHz, Chloroform-*d*) of **15a**

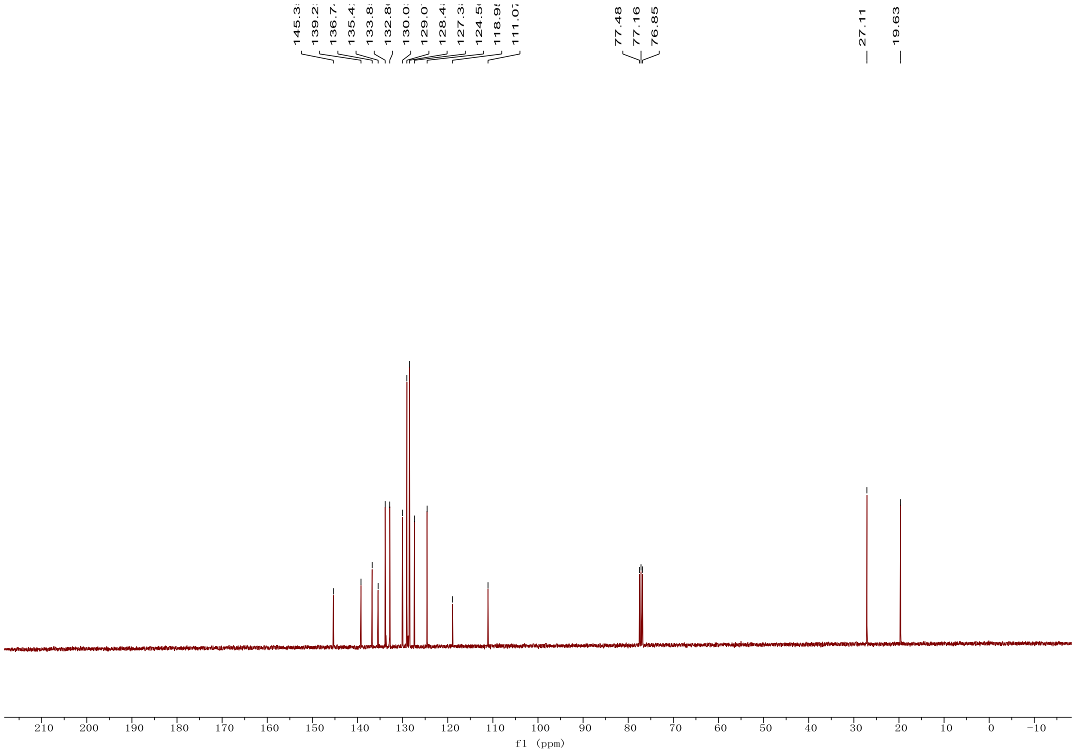

Supplementary Figure 31. ¹³C NMR (101 MHz, Chloroform-*d*) of **15a**

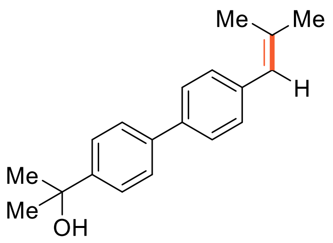

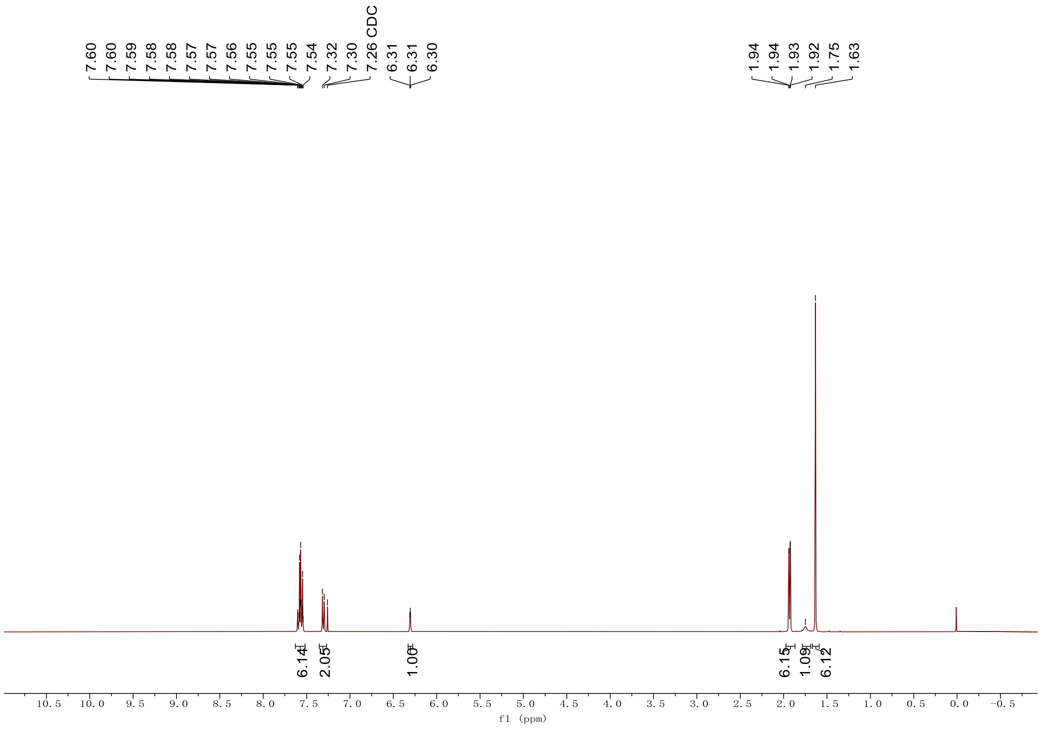

Supplementary Figure 32. ^{1}**H NMR** (400 MHz, Chloroform-*d*) of **16a**

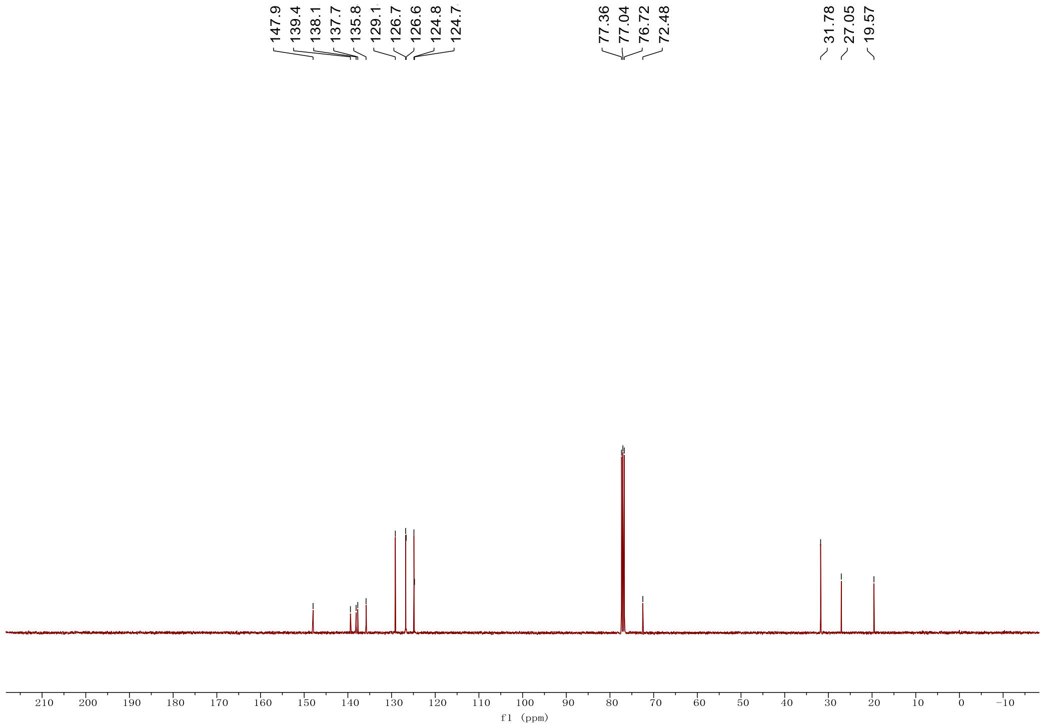

Supplementary Figure 33. ^{13}**C NMR (**101 MHz, Chloroform-*d*) of **16a**

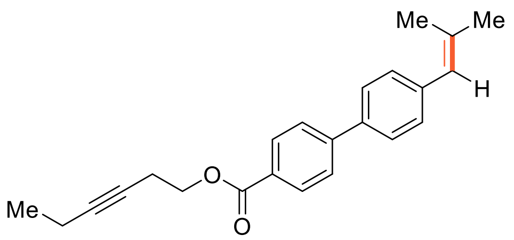

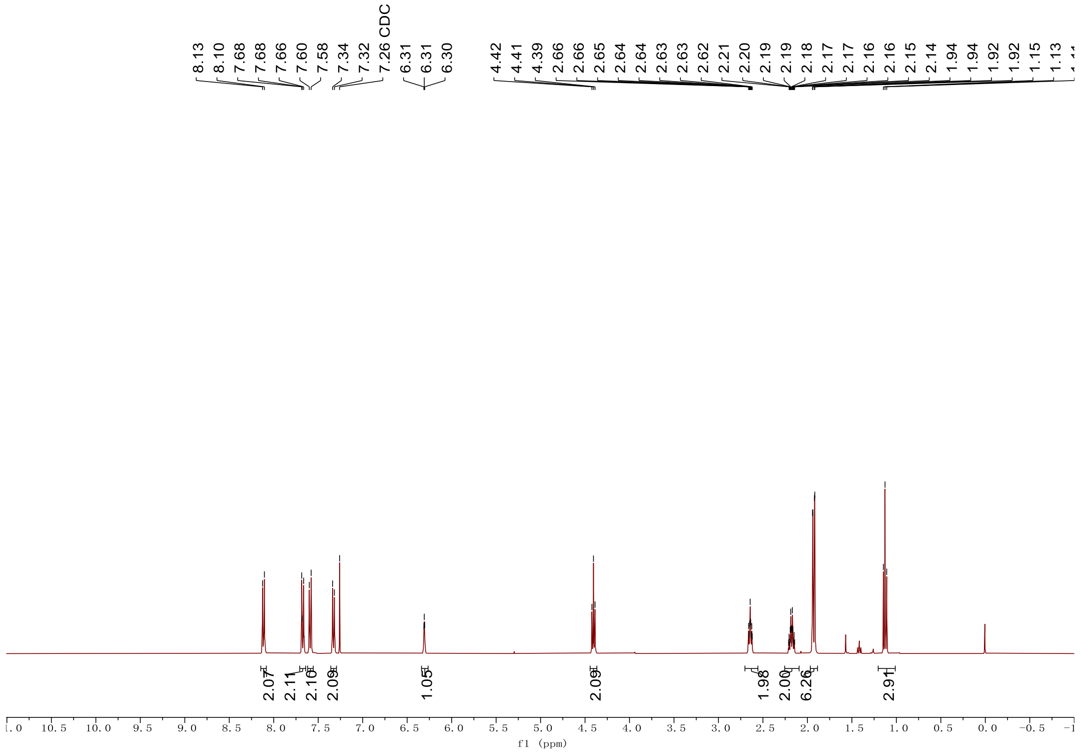

Supplementary Figure 34. ^{1}**H NMR** (400 MHz, Chloroform-*d*) of **17a**

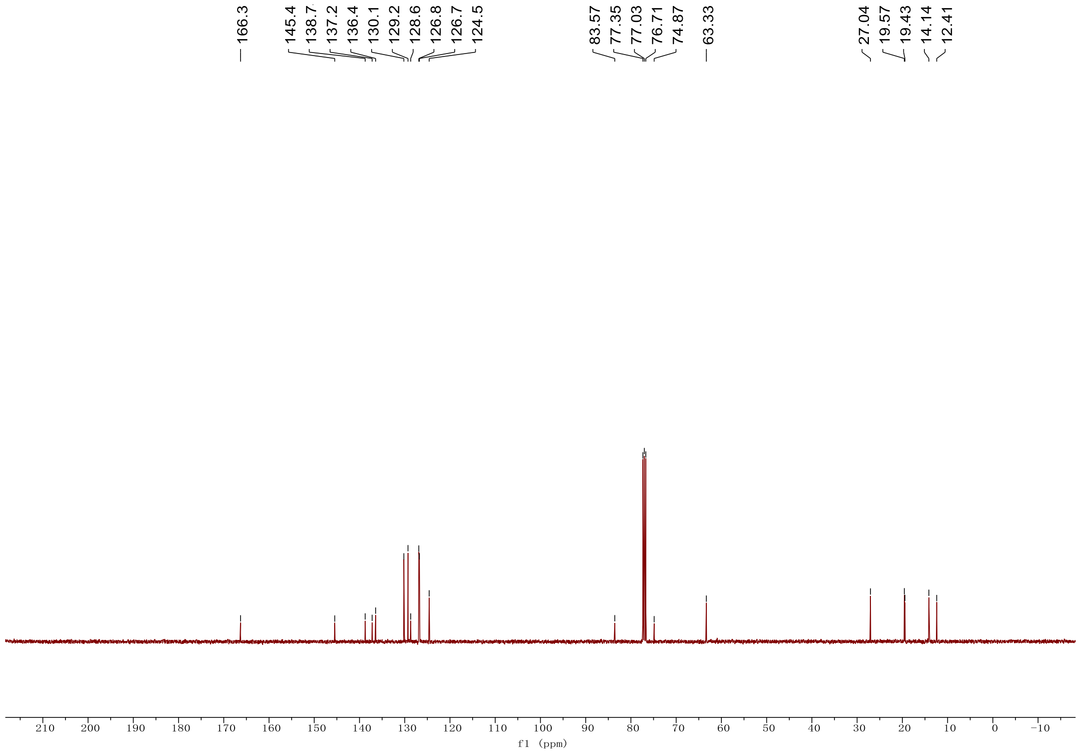

Supplementary Figure 35. ^{13}**C NMR (**101 MHz, Chloroform-*d*) of **17a**

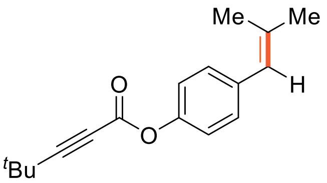

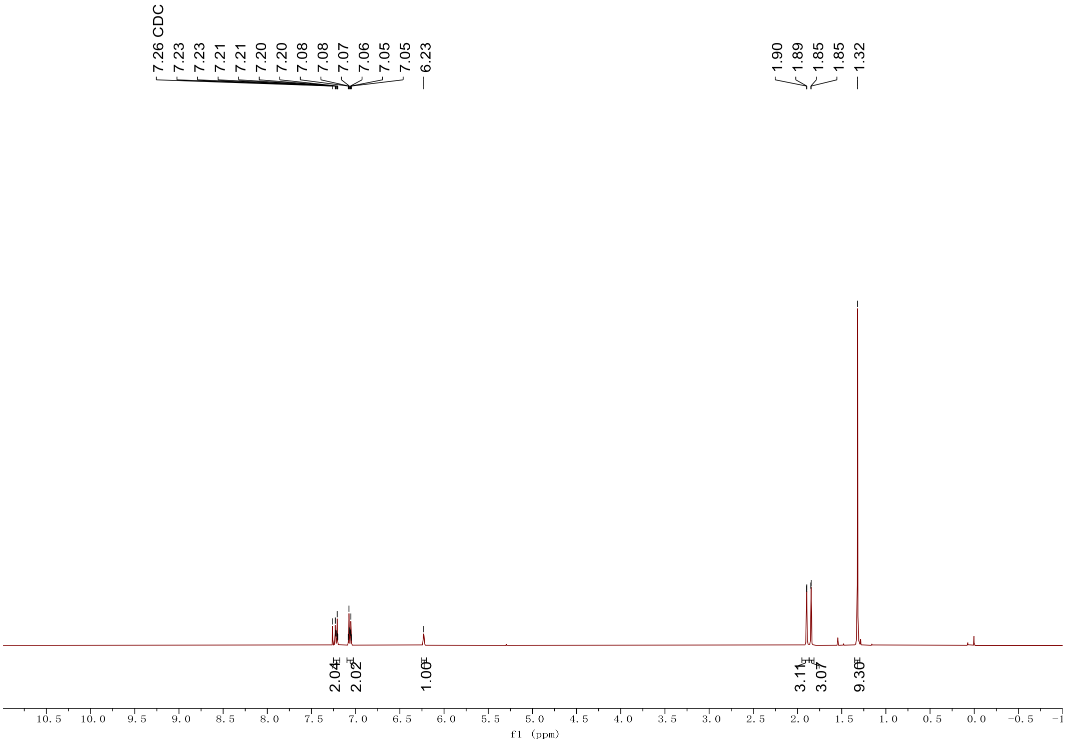

Supplementary Figure 36. ^{1}**H NMR** (400 MHz, Chloroform-*d*) of **18a**

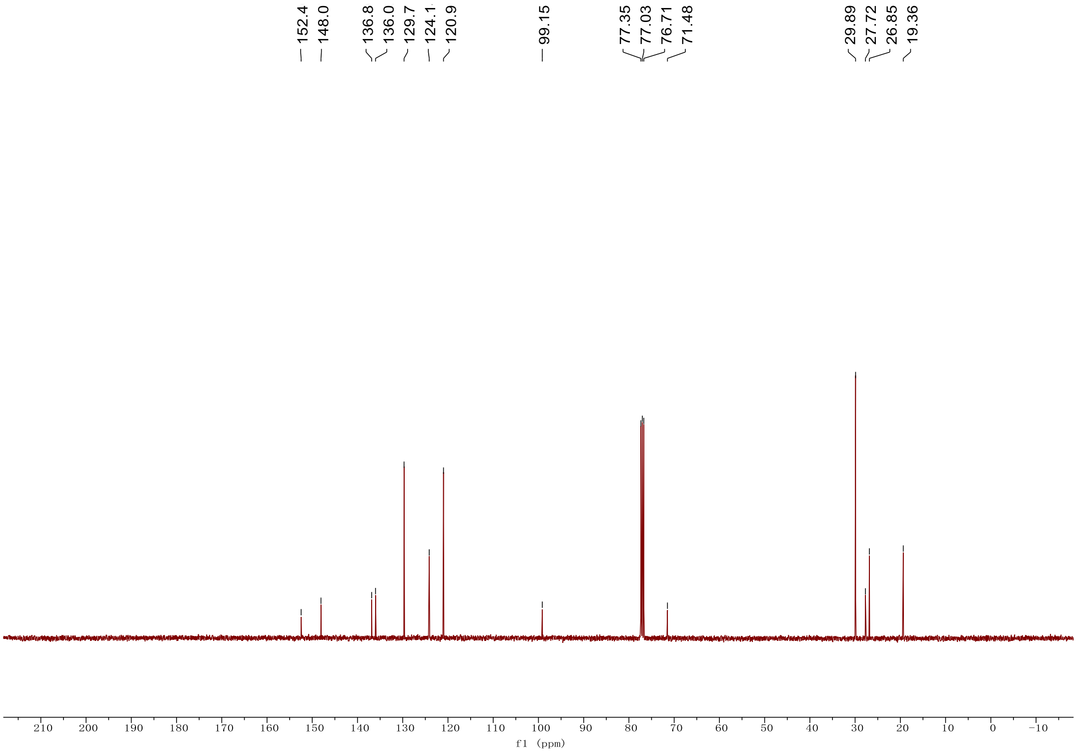

Supplementary Figure 37. ^{13}**C NMR (**101 MHz, Chloroform-*d*) of **18a**

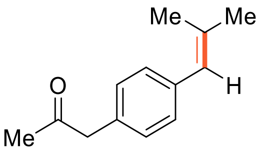

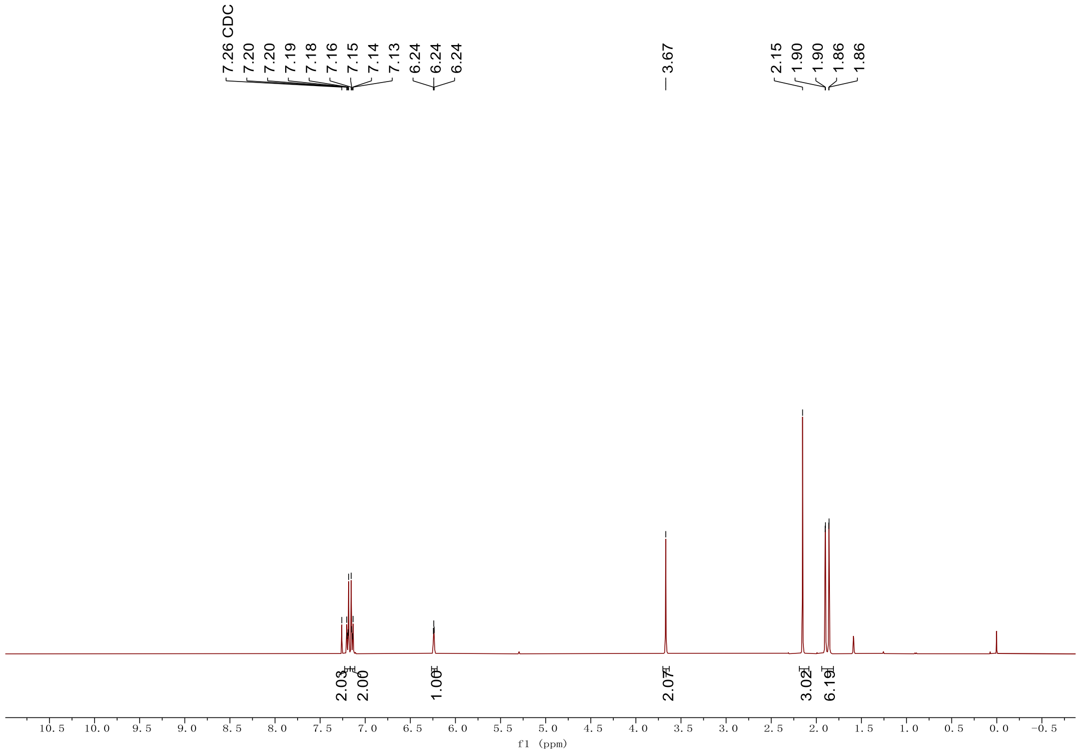

Supplementary Figure 38. ^{1}H NMR (400 MHz, Chloroform-*d*) of **19a**

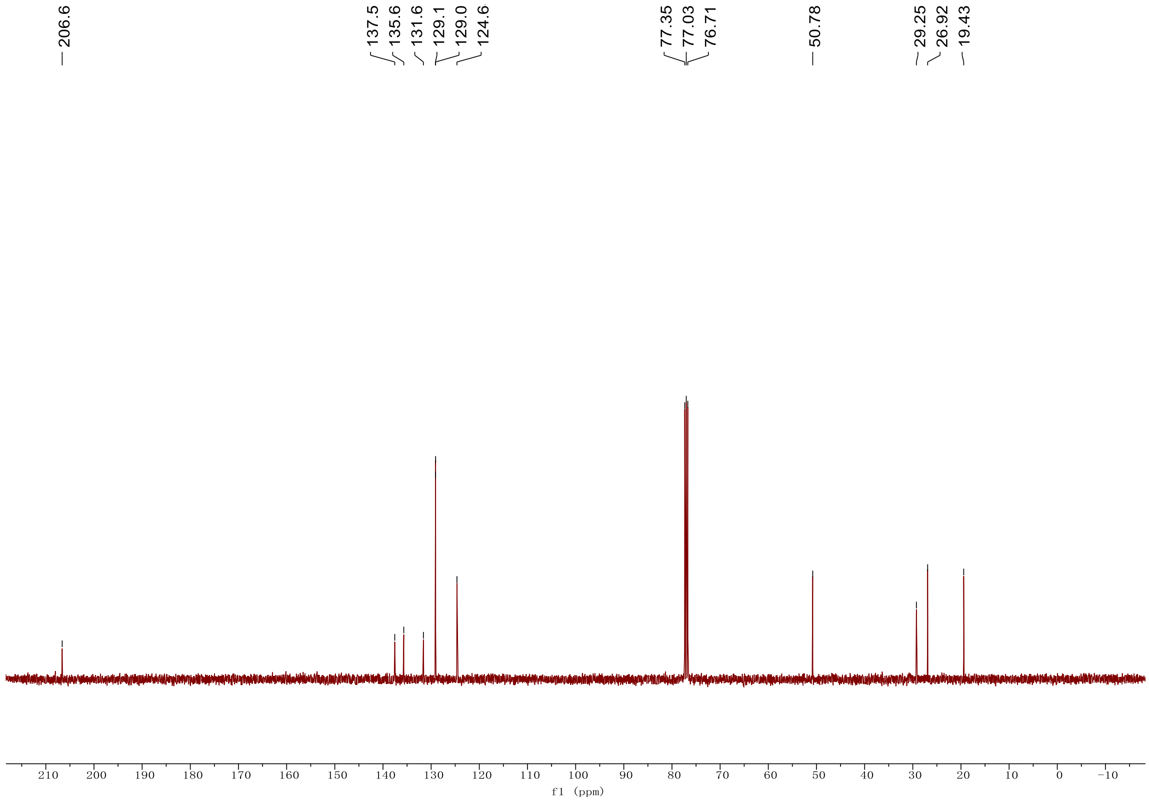

Supplementary Figure 39. ^{13}C NMR (101 MHz, Chloroform-*d*) of **19a**

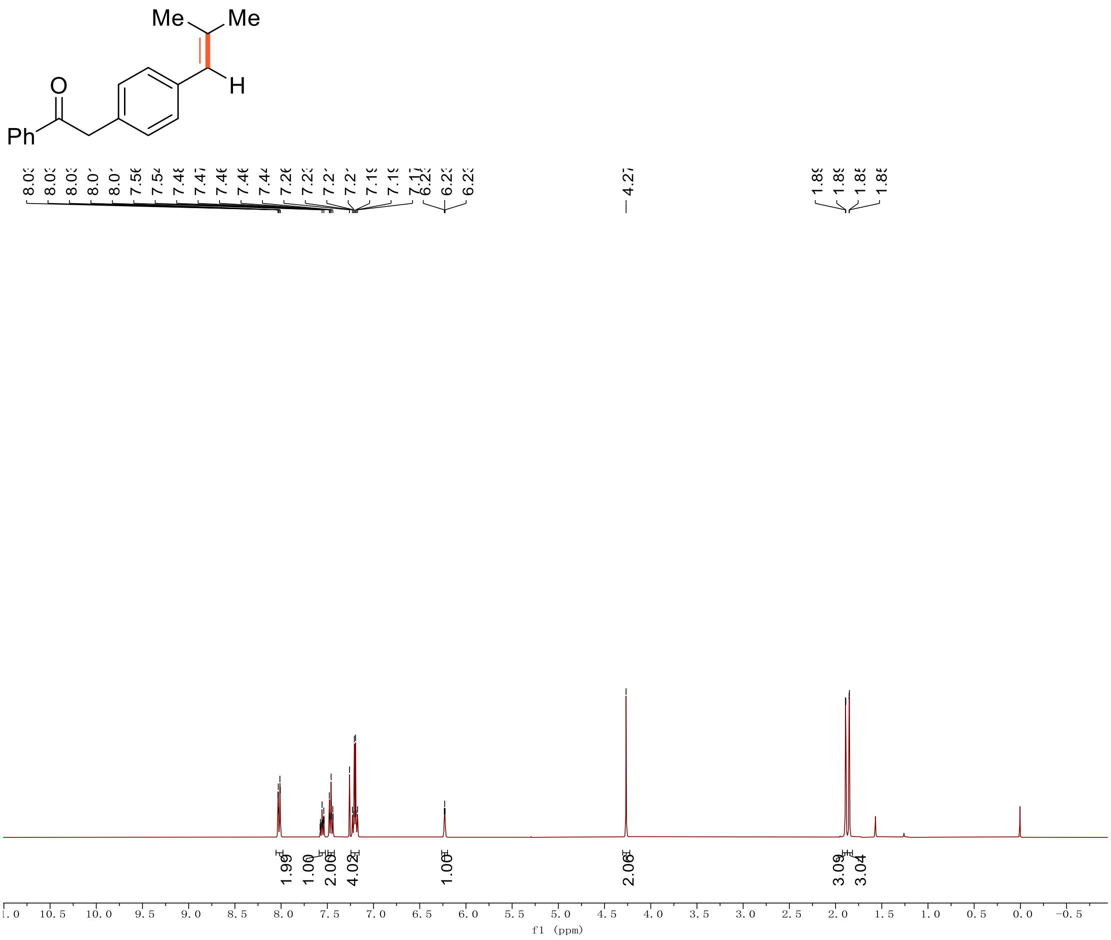

Supplementary Figure 40. **^{1}H NMR** (400 MHz, Chloroform-*d*) of **20a**

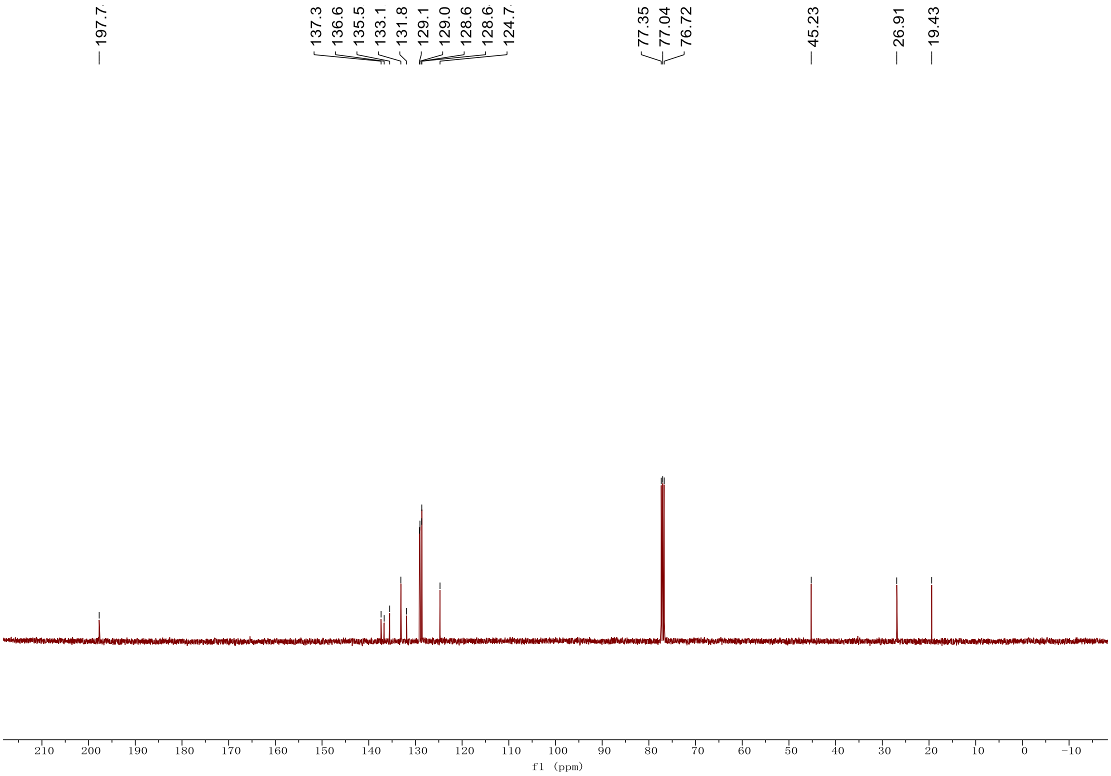

Supplementary Figure 41. **^{13}C NMR (**101 MHz, Chloroform-*d*) of **20a**

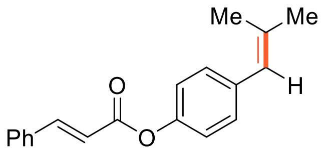

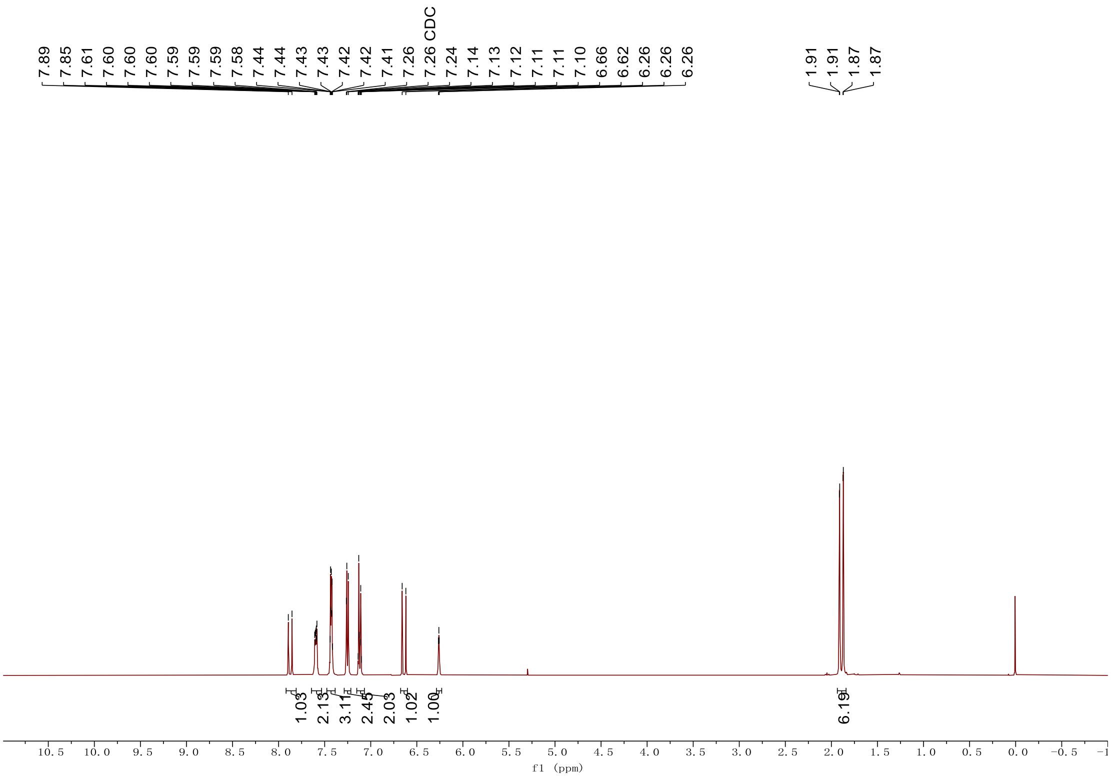

Supplementary Figure 42. **^{1}H NMR** (400 MHz, Chloroform-*d*) of **21a**

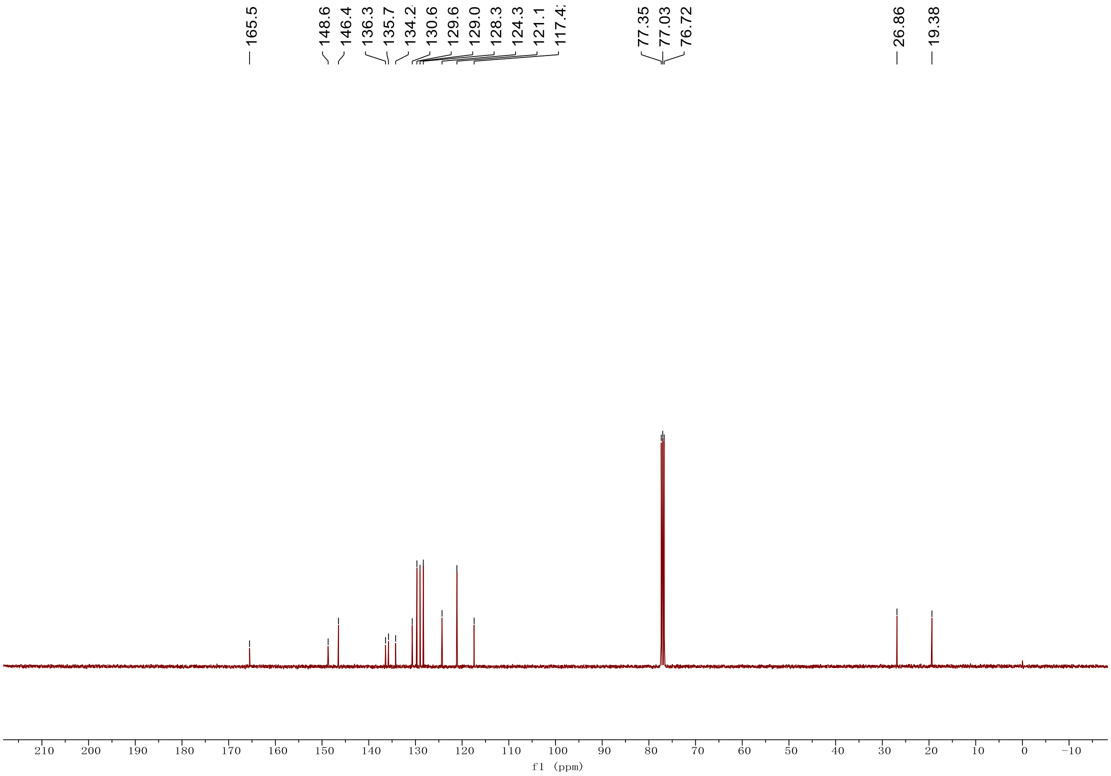

Supplementary Figure 43. **^{13}C NMR (**101 MHz, Chloroform-*d*) of **21a**

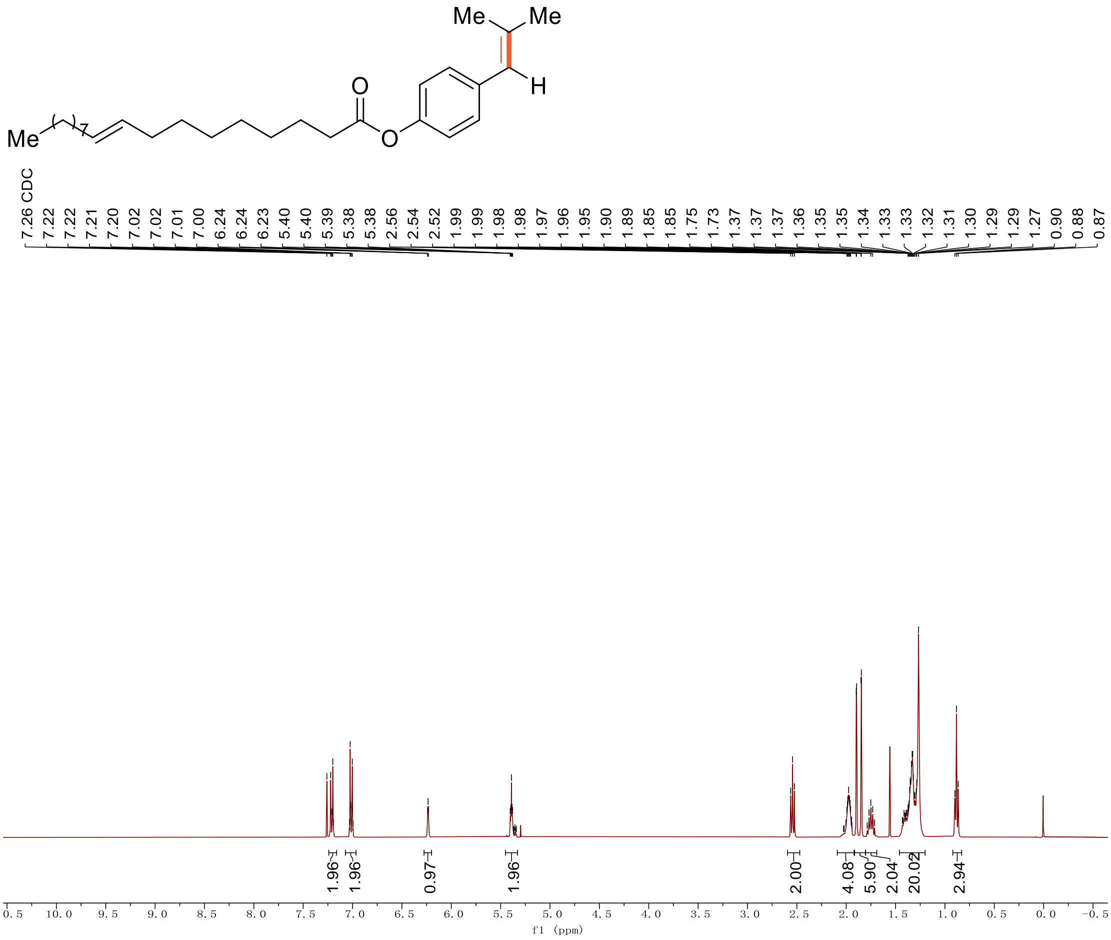

Supplementary Figure 44. ^{1}**H NMR** (400 MHz, Chloroform-*d*) of **22a**

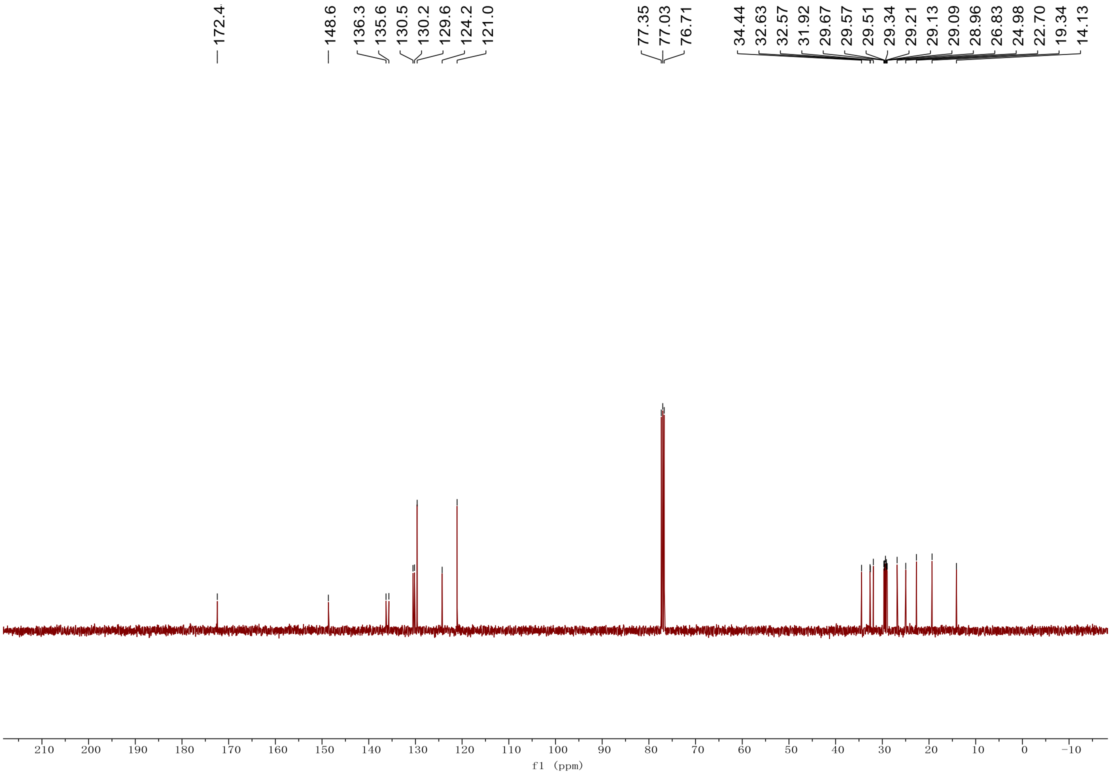

Supplementary Figure 45. ^{13}**C NMR (**101 MHz, Chloroform-*d*) of **22a**

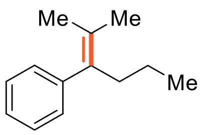

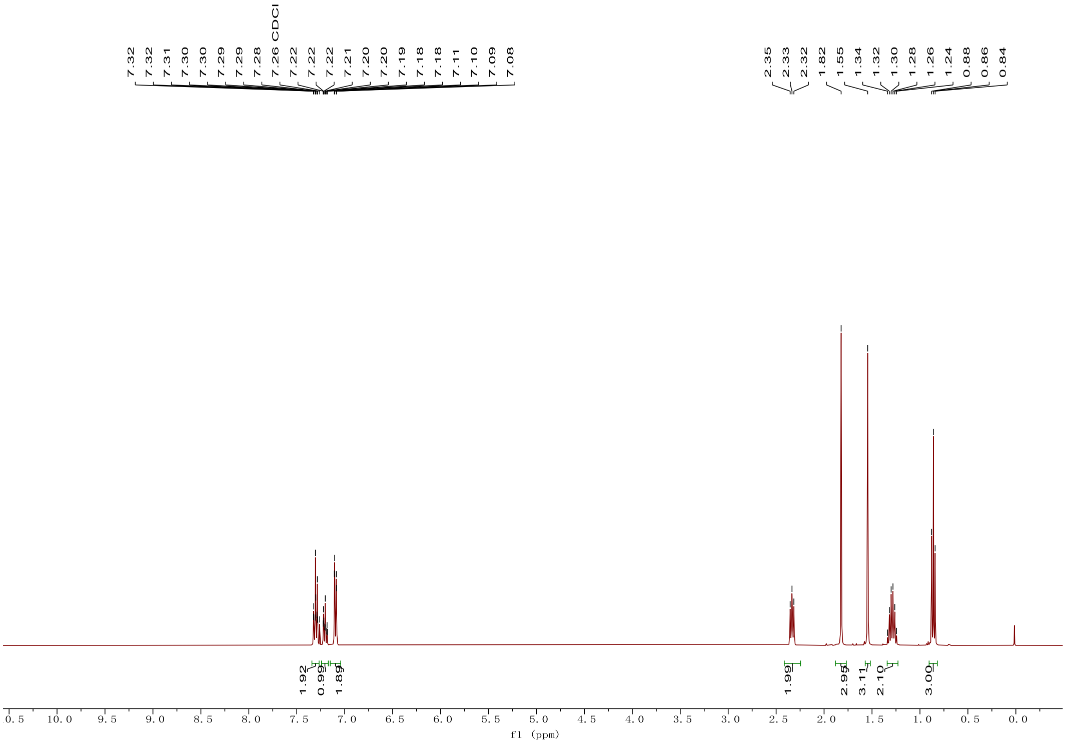

Supplementary Figure 46. ^{1}H NMR (400 MHz, Chloroform-*d*) of **23a**

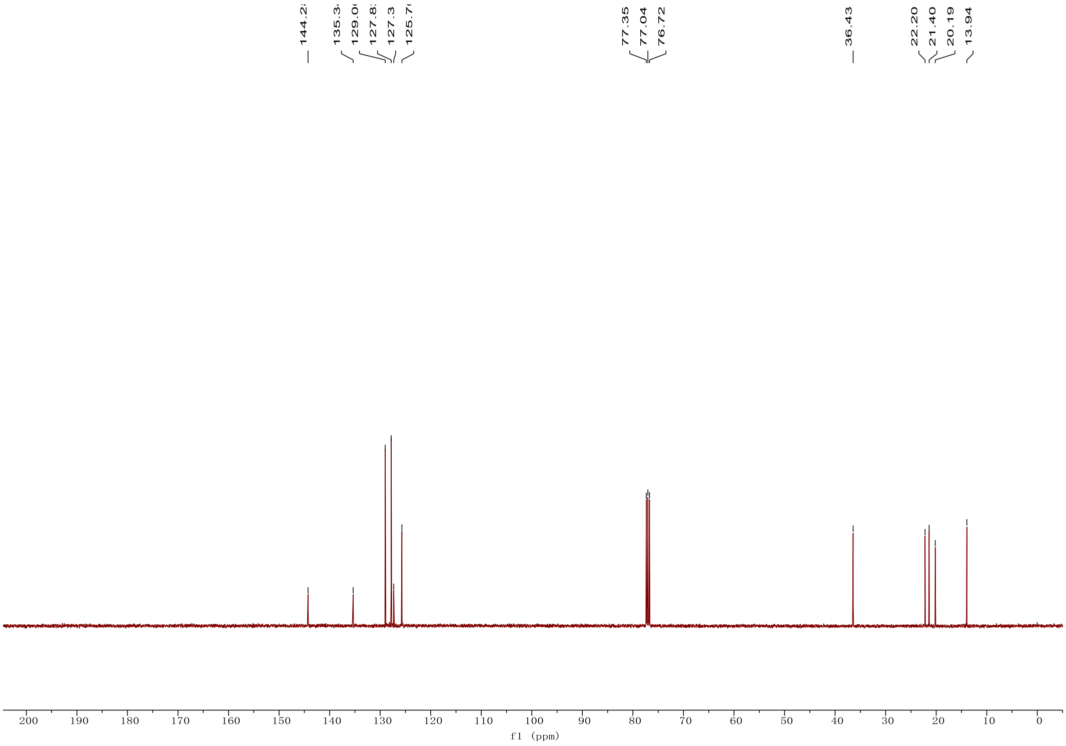

Supplementary Figure 47. ^{13}C NMR (101 MHz, Chloroform-*d*) of **23a**

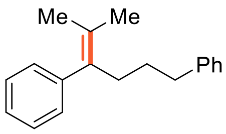

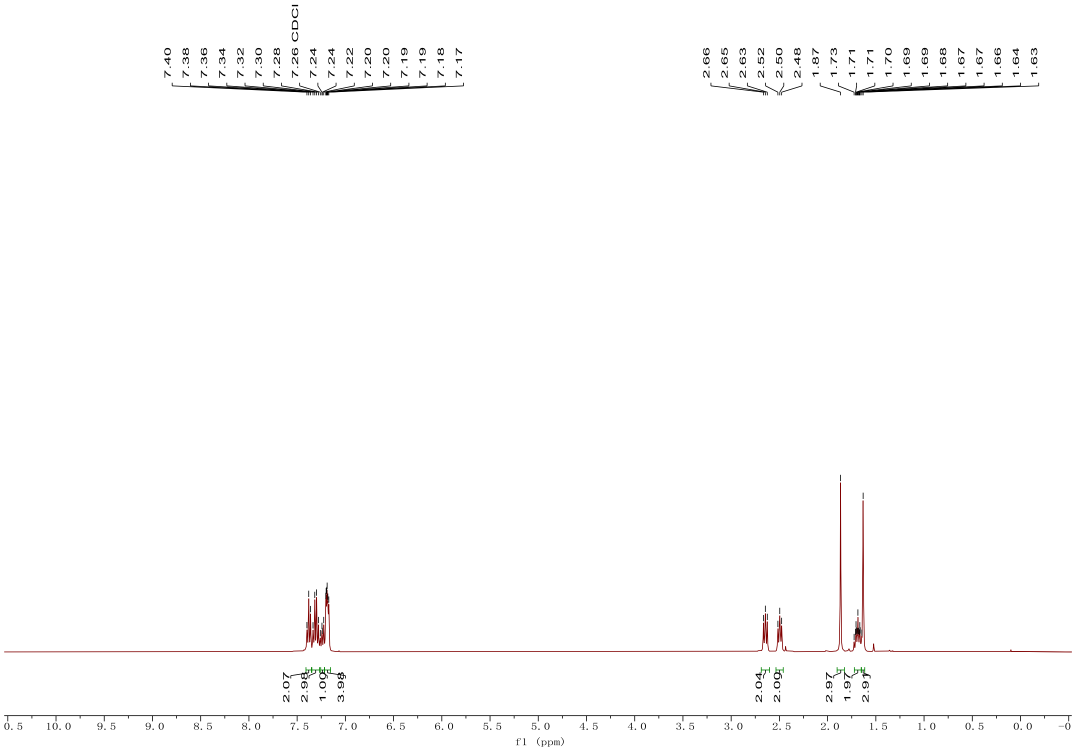

Supplementary Figure 48. ^{1}**H NMR** (400 MHz, Chloroform-*d*) of **24a**

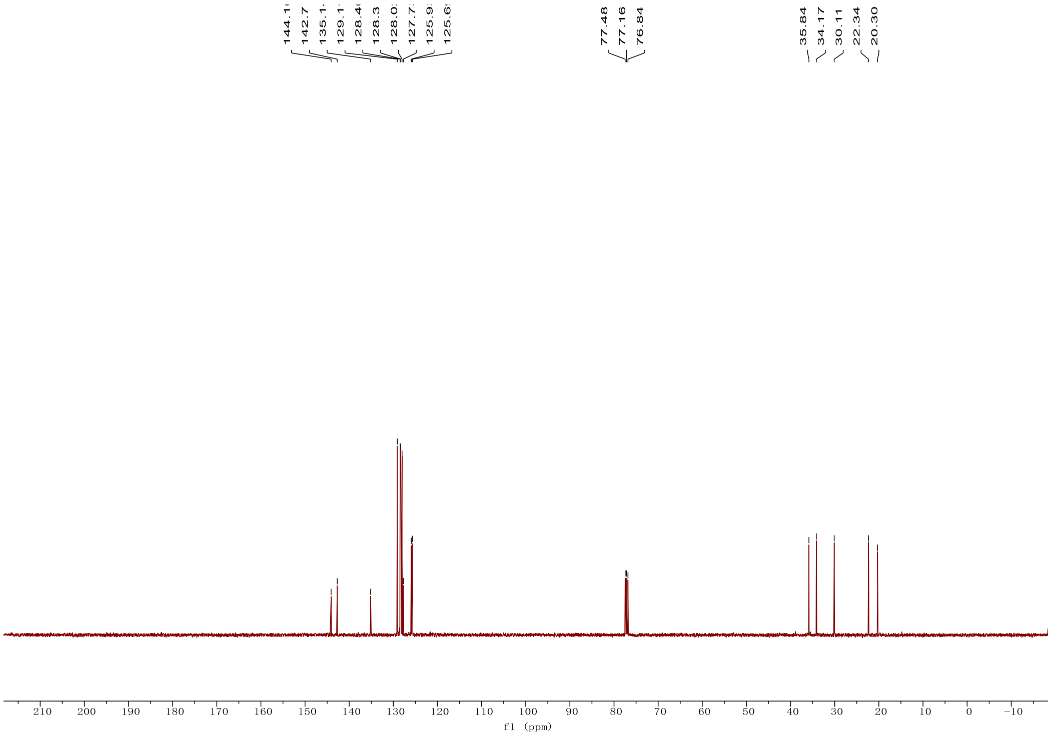

Supplementary Figure 49. ^{13}**C NMR (**101 MHz, Chloroform-*d*) of **24a**

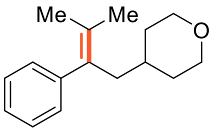

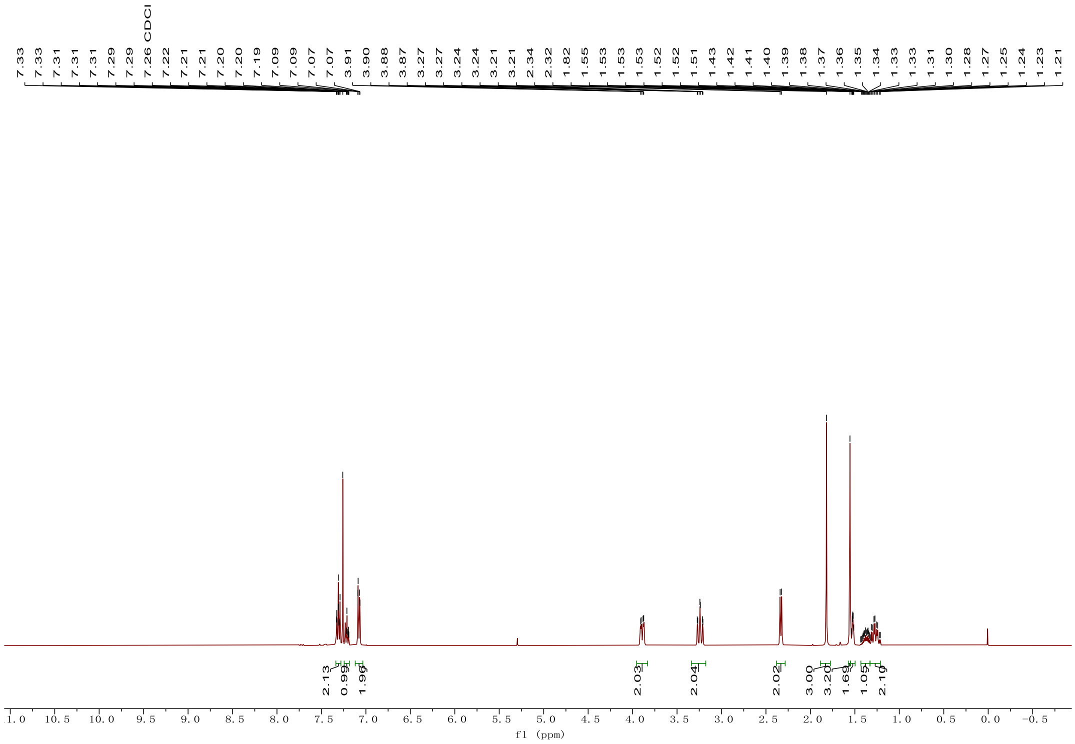

Supplementary Figure 50. ^{1}**H NMR** (400 MHz, Chloroform-*d*) of **25a**

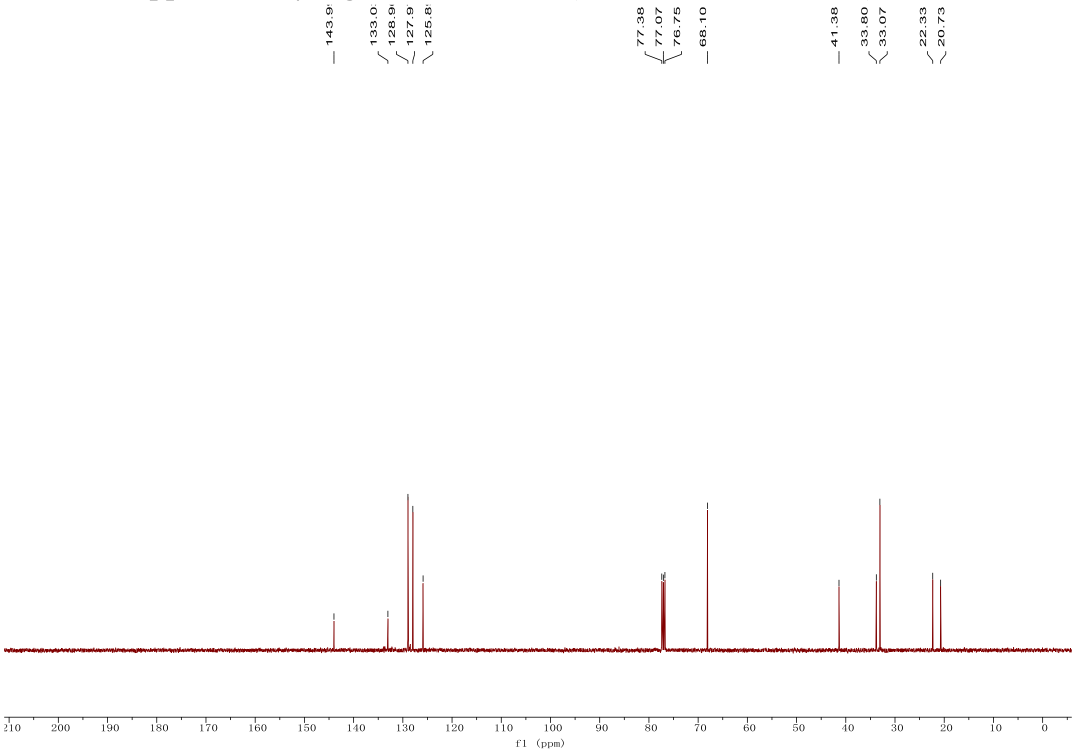

Supplementary Figure 51. ^{13}**C NMR (**101 MHz, Chloroform-*d*) of **25a**

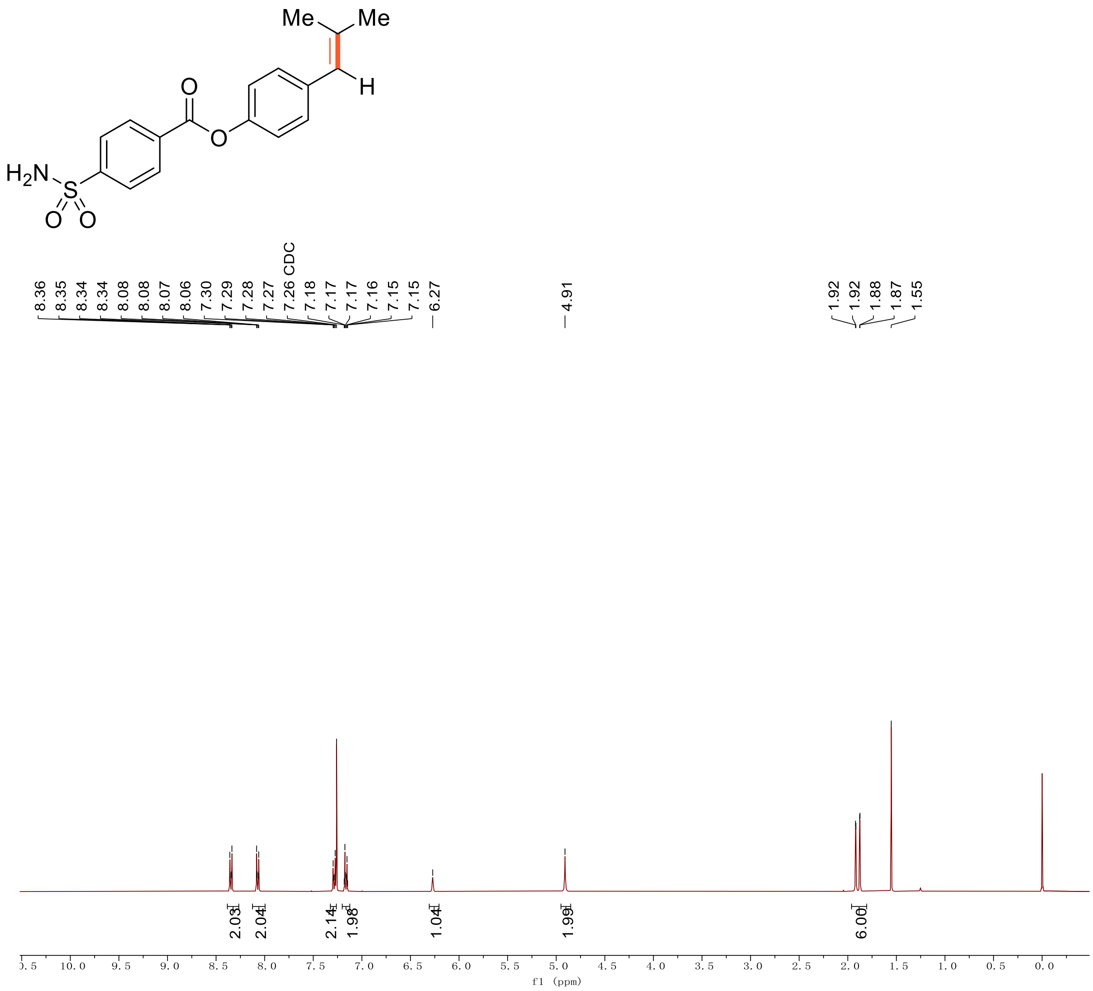

Supplementary Figure 52. ^{1}**H NMR** (400 MHz, Chloroform-*d*) of **29a**

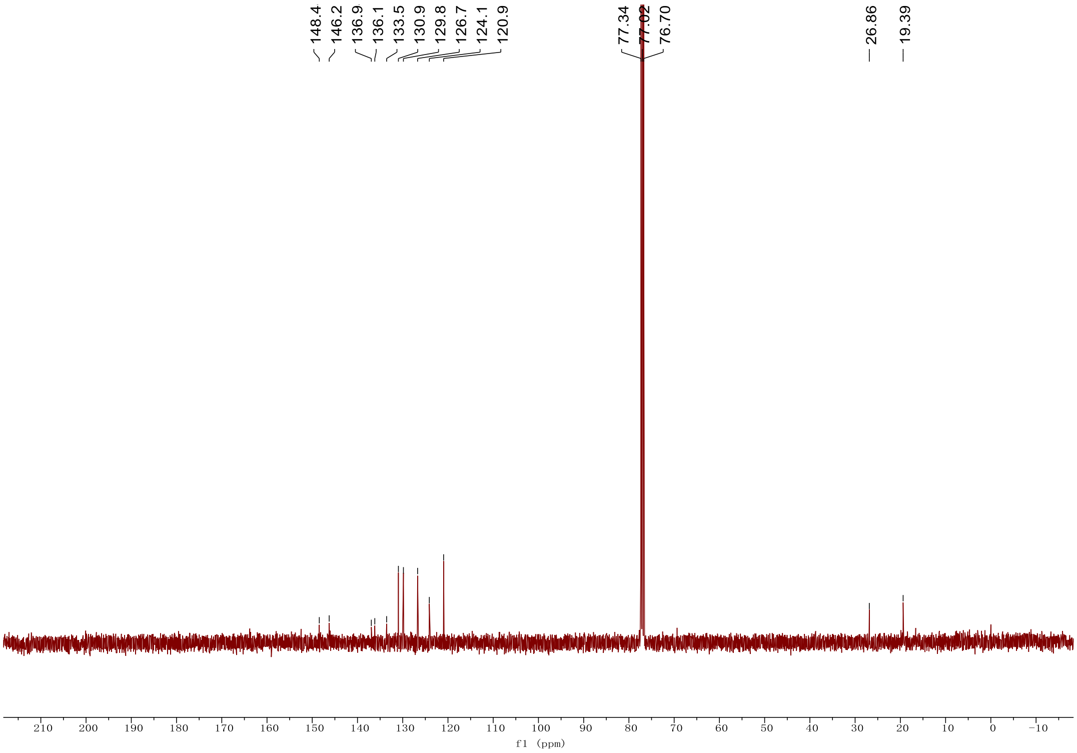

Supplementary Figure 53. ^{13}**C NMR (**101 MHz, Chloroform-*d*) of **29a**

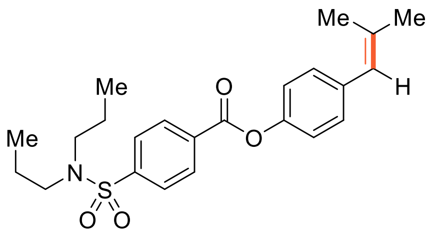

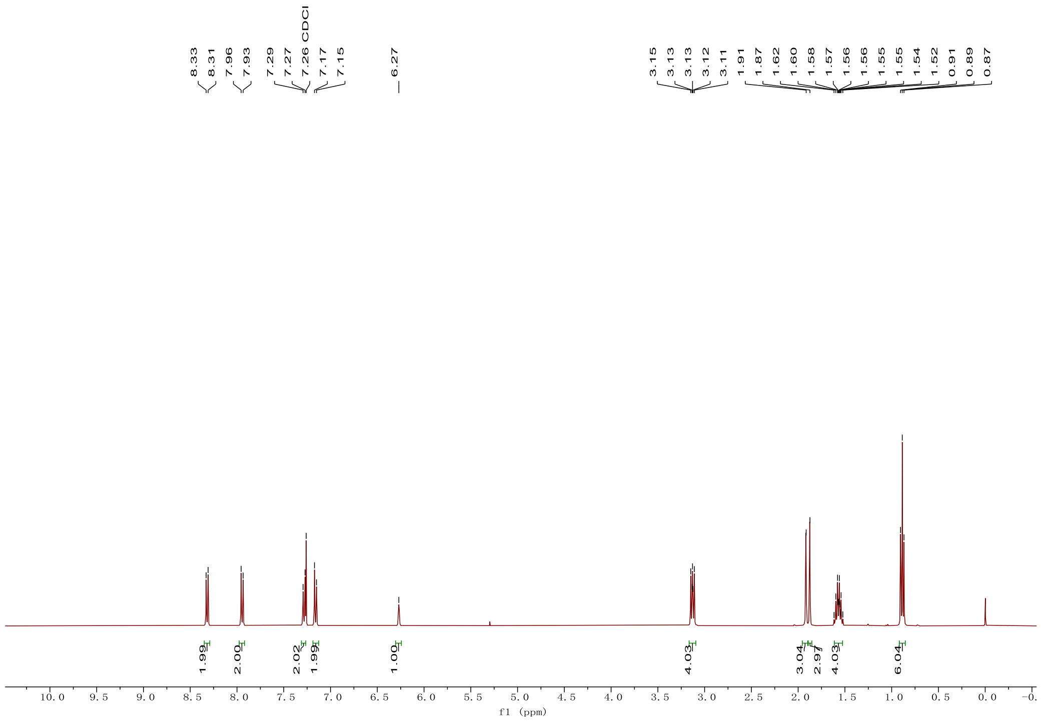

Supplementary Figure 54. ^{1}**H NMR** (400 MHz, Chloroform-*d*) of **30a**

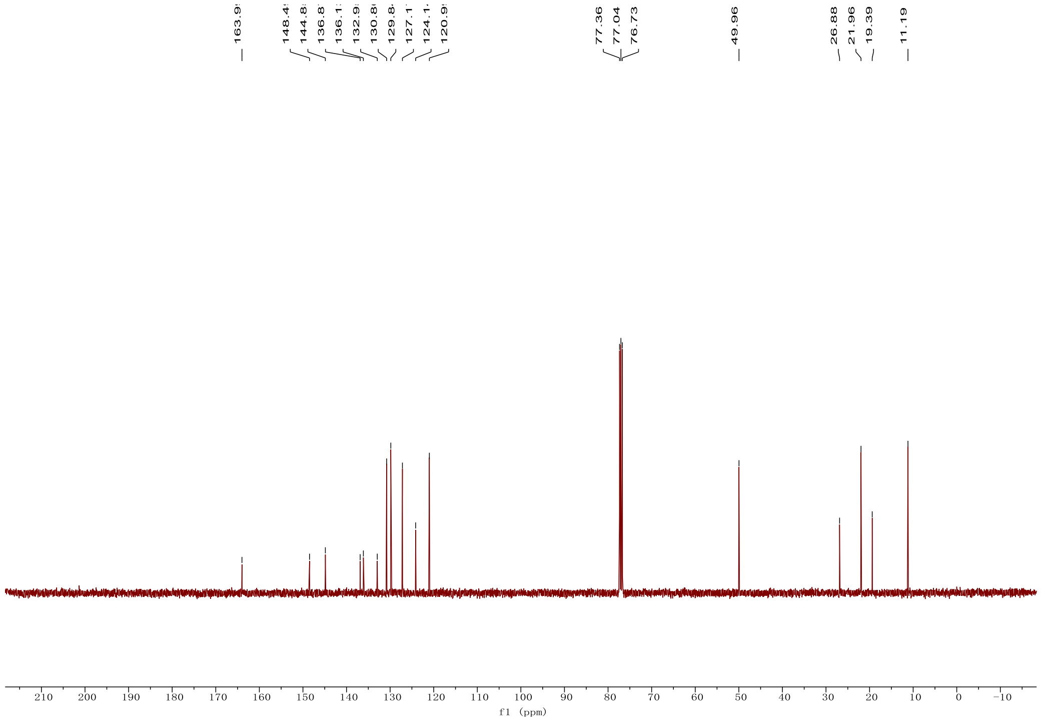

Supplementary Figure 55. ^{13}**C NMR (**101 MHz, Chloroform-*d*) of **30a**

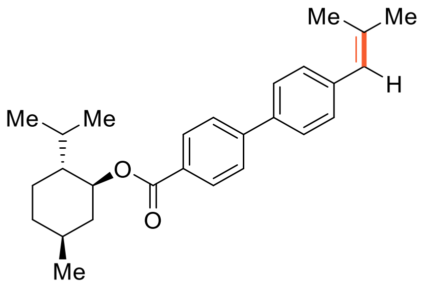

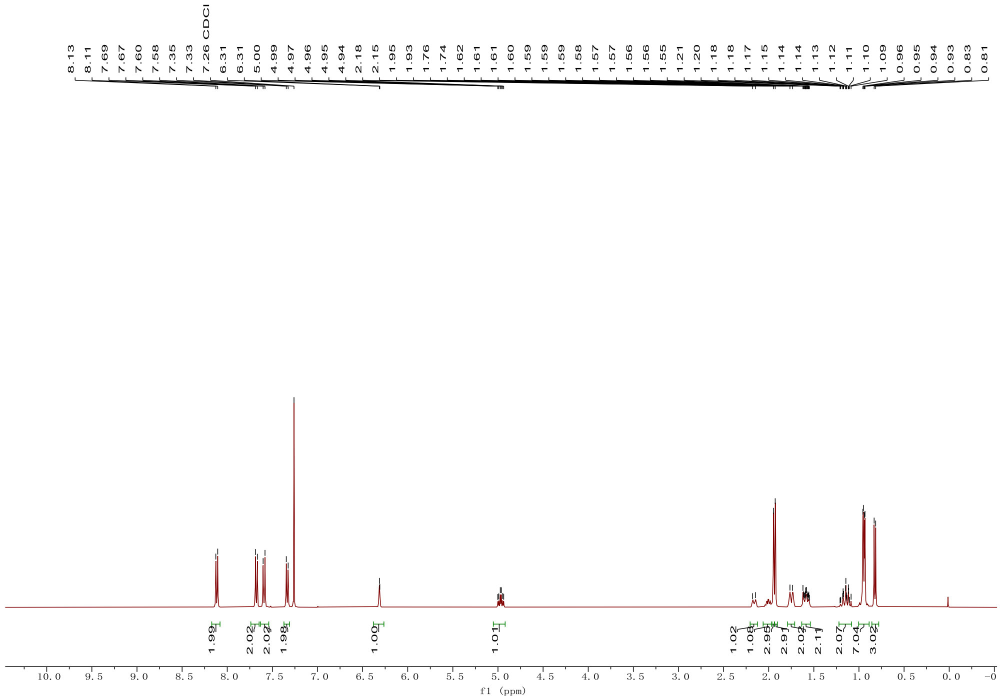

Supplementary Figure 56. ¹H NMR (400 MHz, Chloroform-*d*) of **31a**

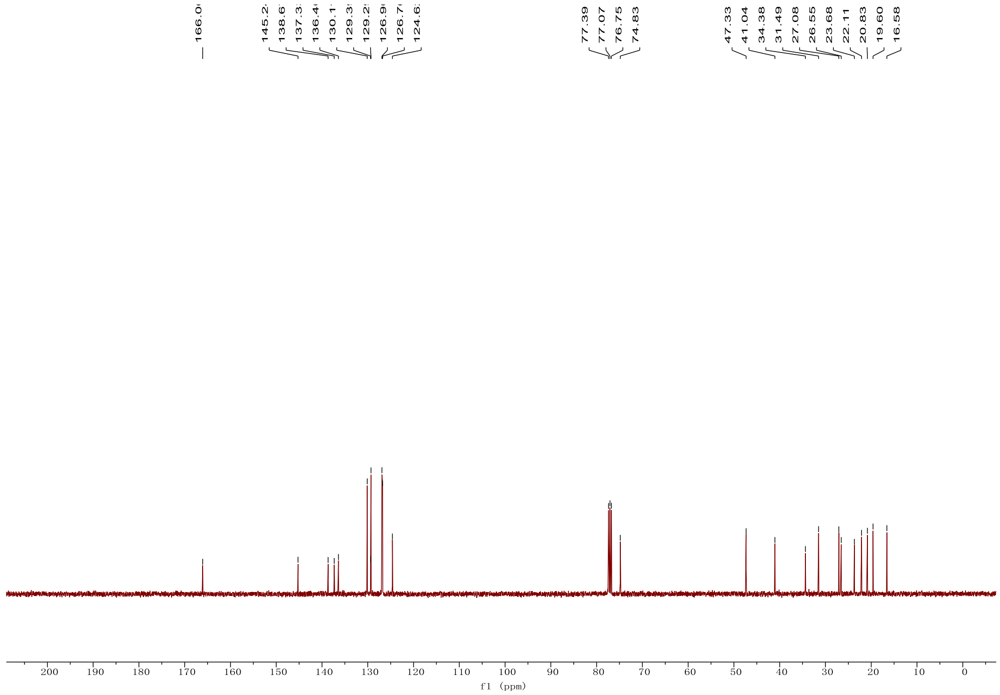

Supplementary Figure 57. ¹³C NMR (101 MHz, Chloroform-*d*) of **31a**

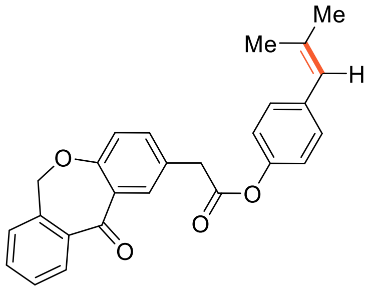

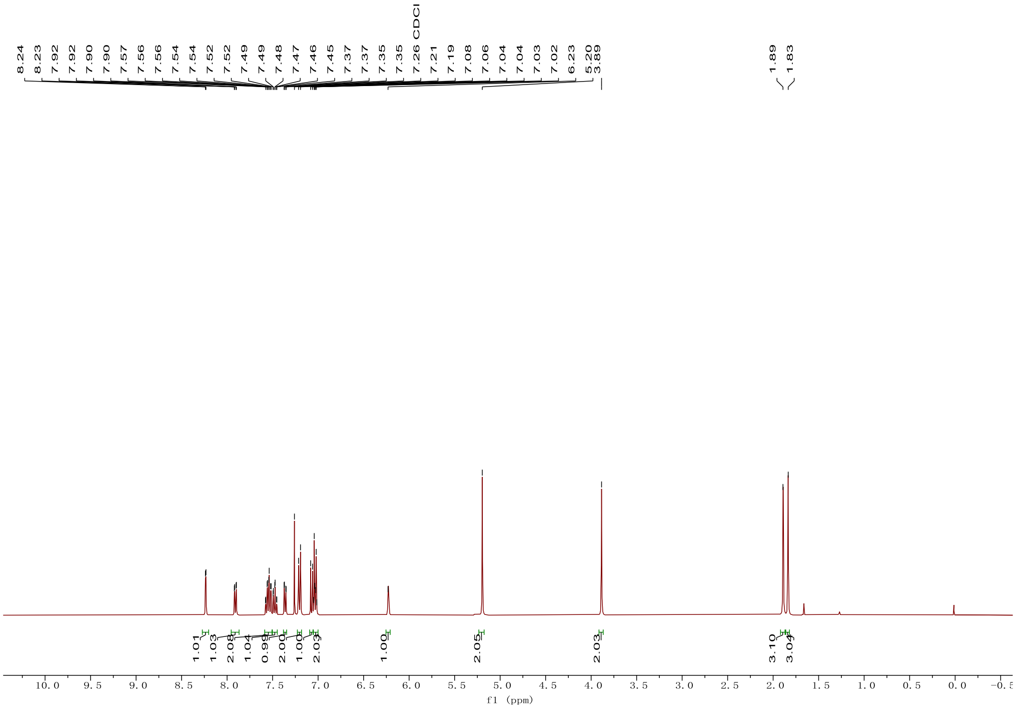

Supplementary Figure 58. **^{1}H NMR** (400 MHz, Chloroform-*d*) of **32a**

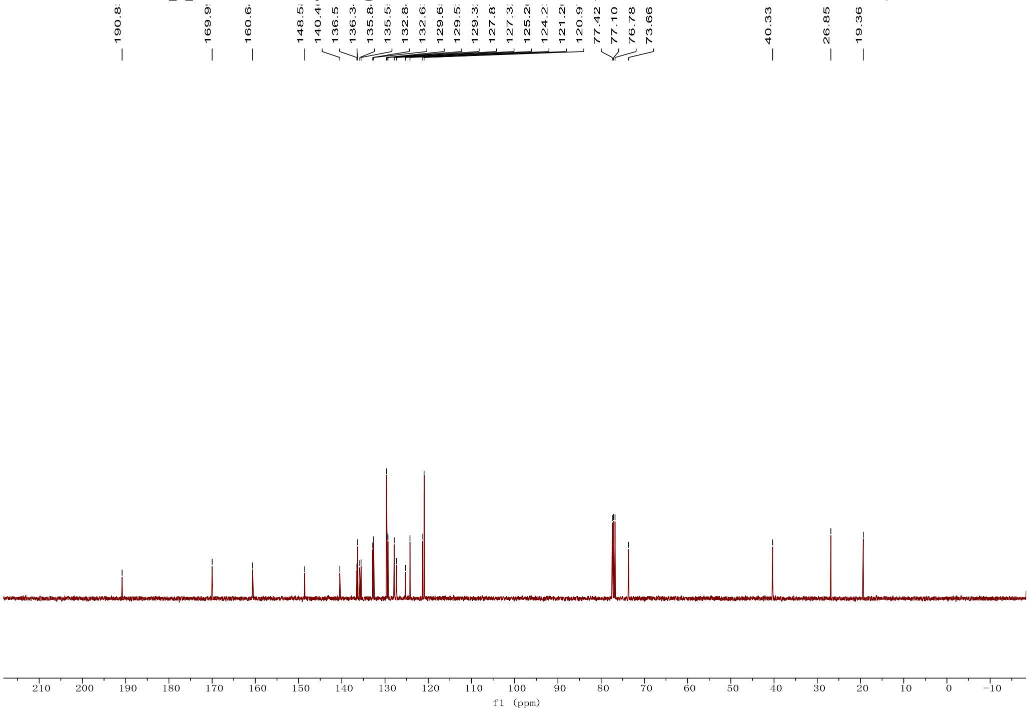

Supplementary Figure 59. **^{13}C NMR (**101 MHz, Chloroform-*d*) of **32a**

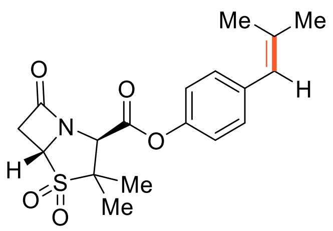

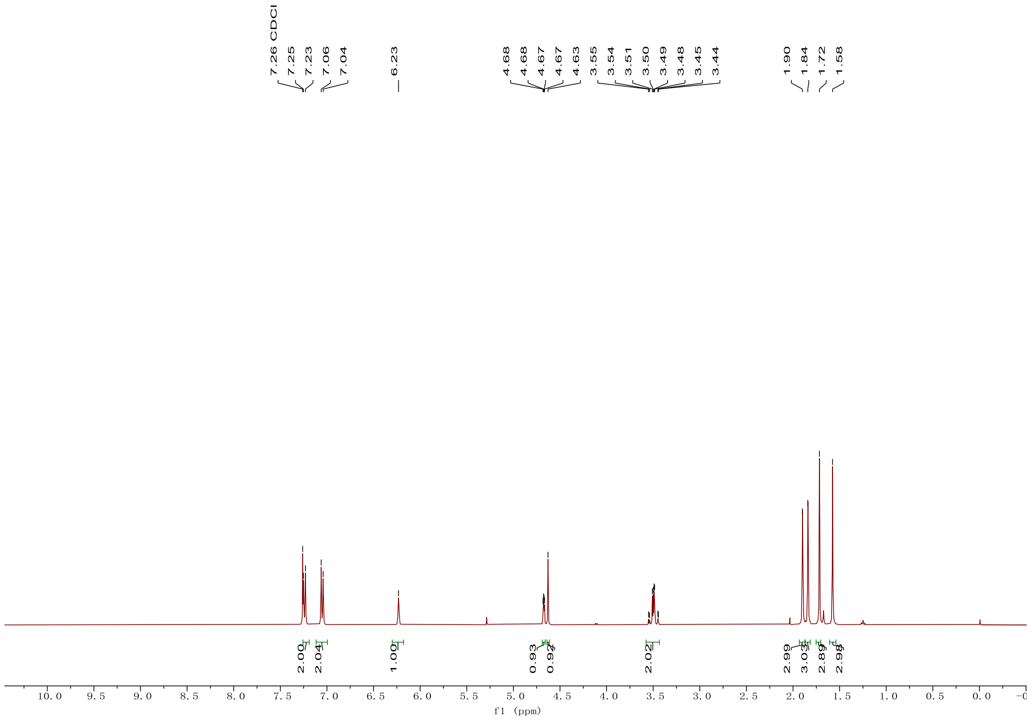

Supplementary Figure 60. ^{1}H NMR (400 MHz, Chloroform-*d*) of **33a**

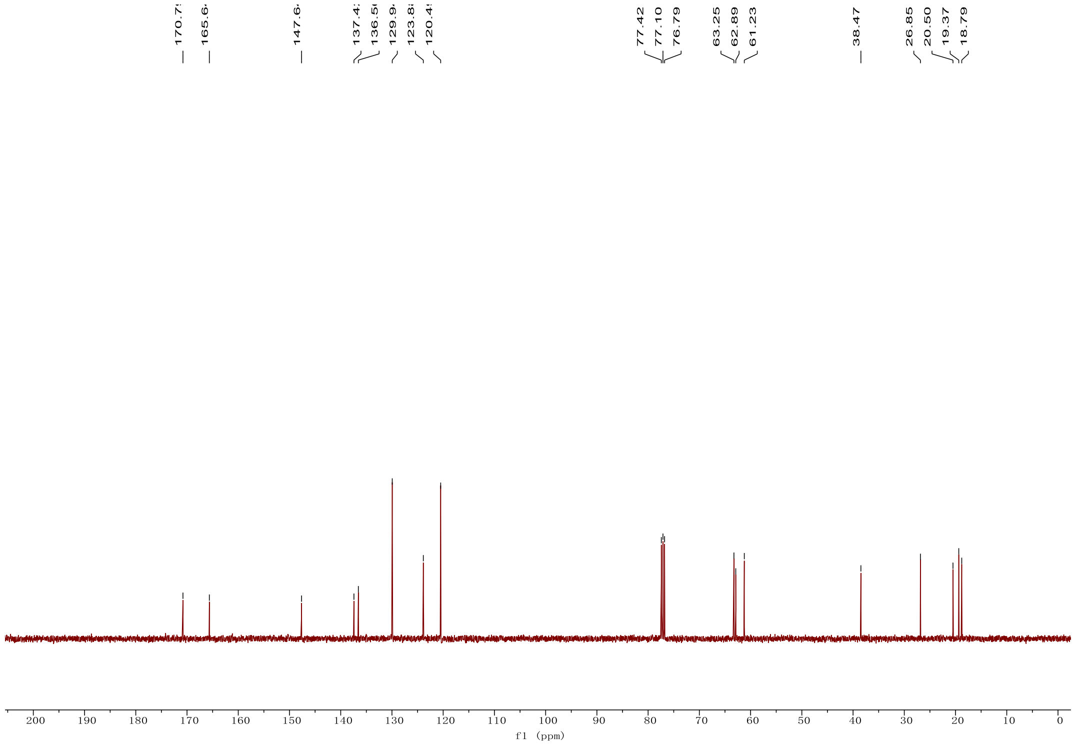

Supplementary Figure 61. ^{13}C NMR (101 MHz, Chloroform-*d*) of **33a**

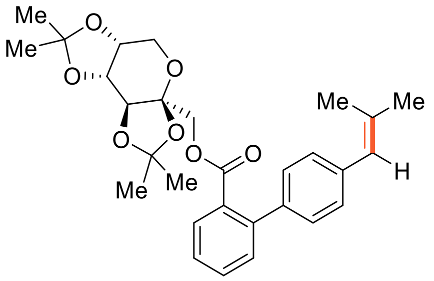

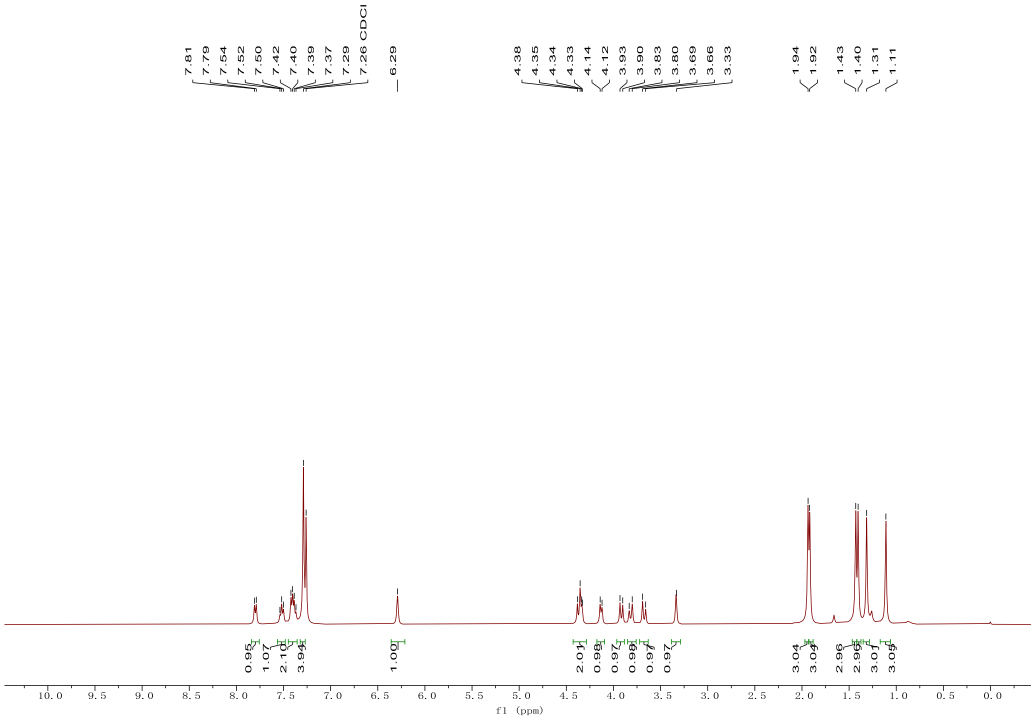

Supplementary Figure 62. ^{1}**H NMR** (400 MHz, Chloroform-*d*) of **34a**

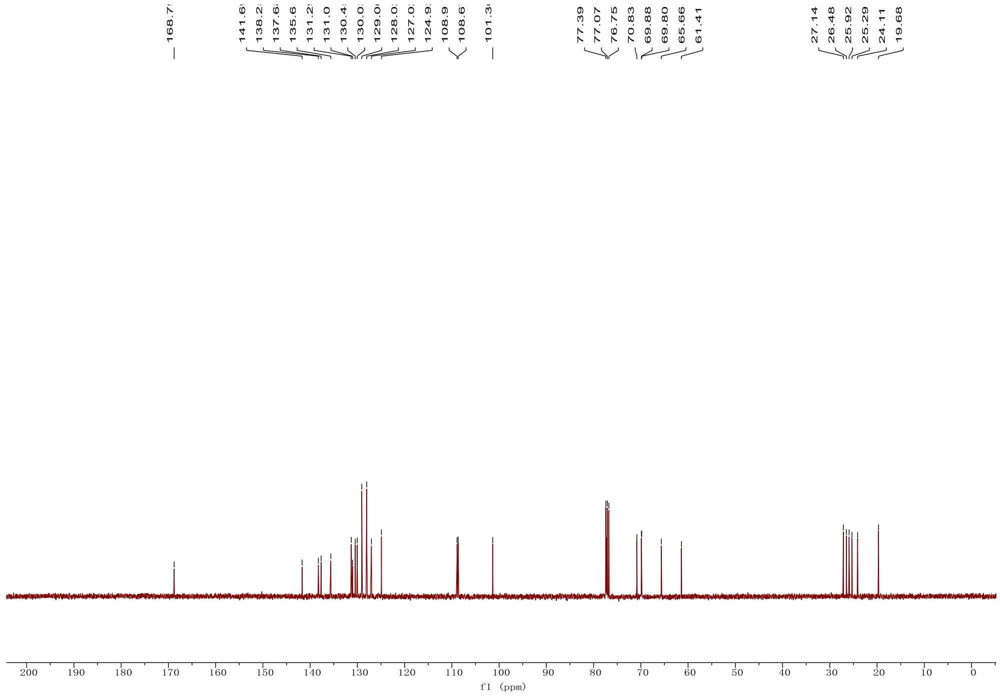

Supplementary Figure 63. ^{13}**C NMR (**101 MHz, Chloroform-*d*) of **34a**

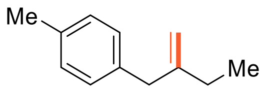

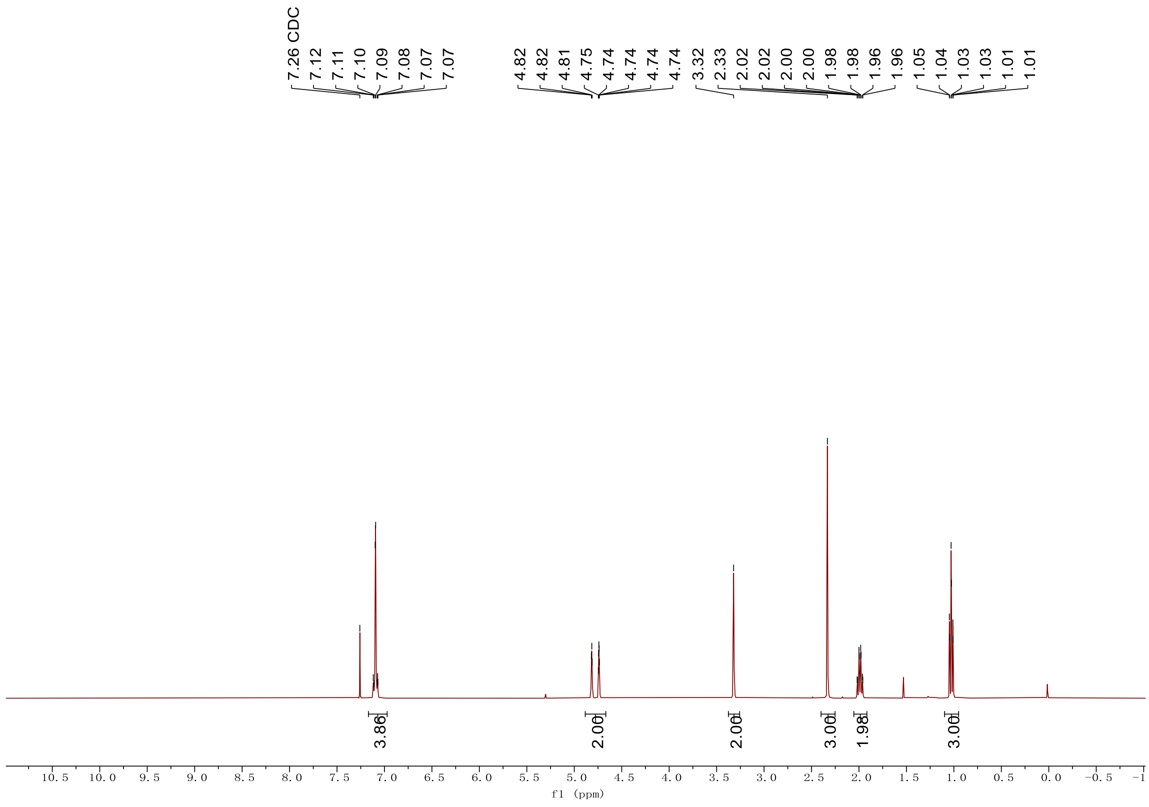

Supplementary Figure 64. ^{1}**H NMR** (400 MHz, Chloroform-*d*) of **36a**

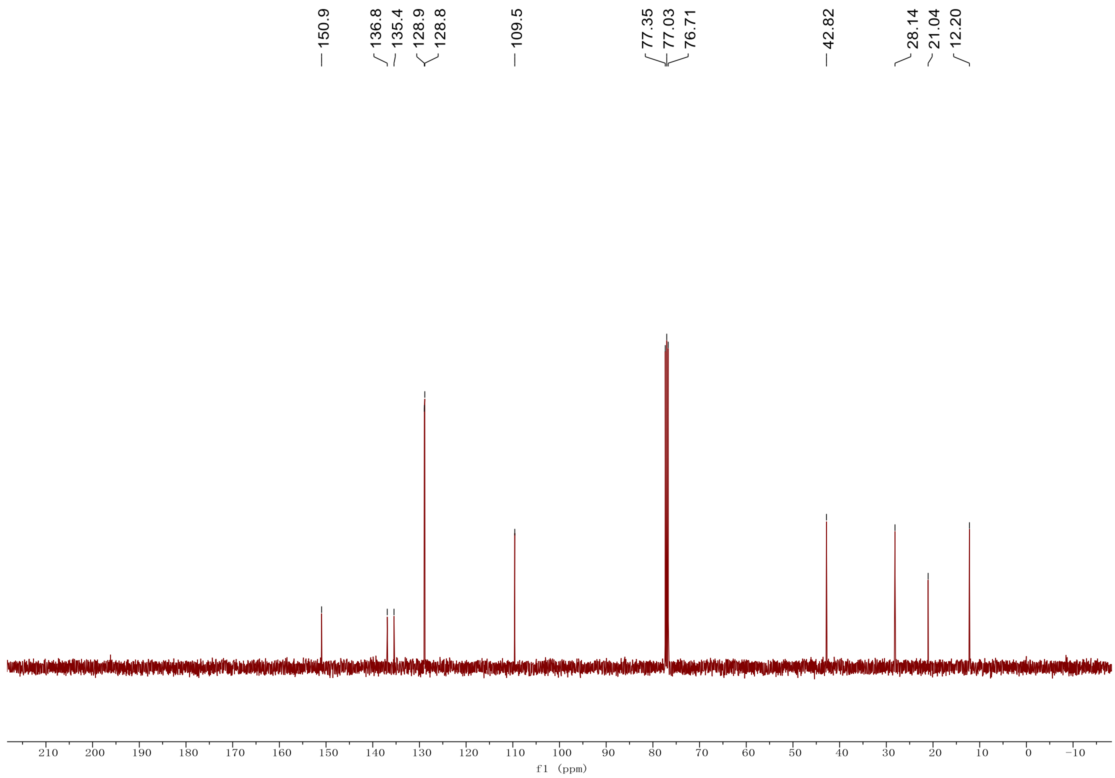

Supplementary Figure 65. ^{13}**C NMR (**101 MHz, Chloroform-*d*) of **36a**

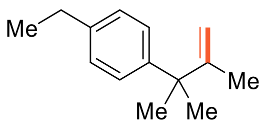

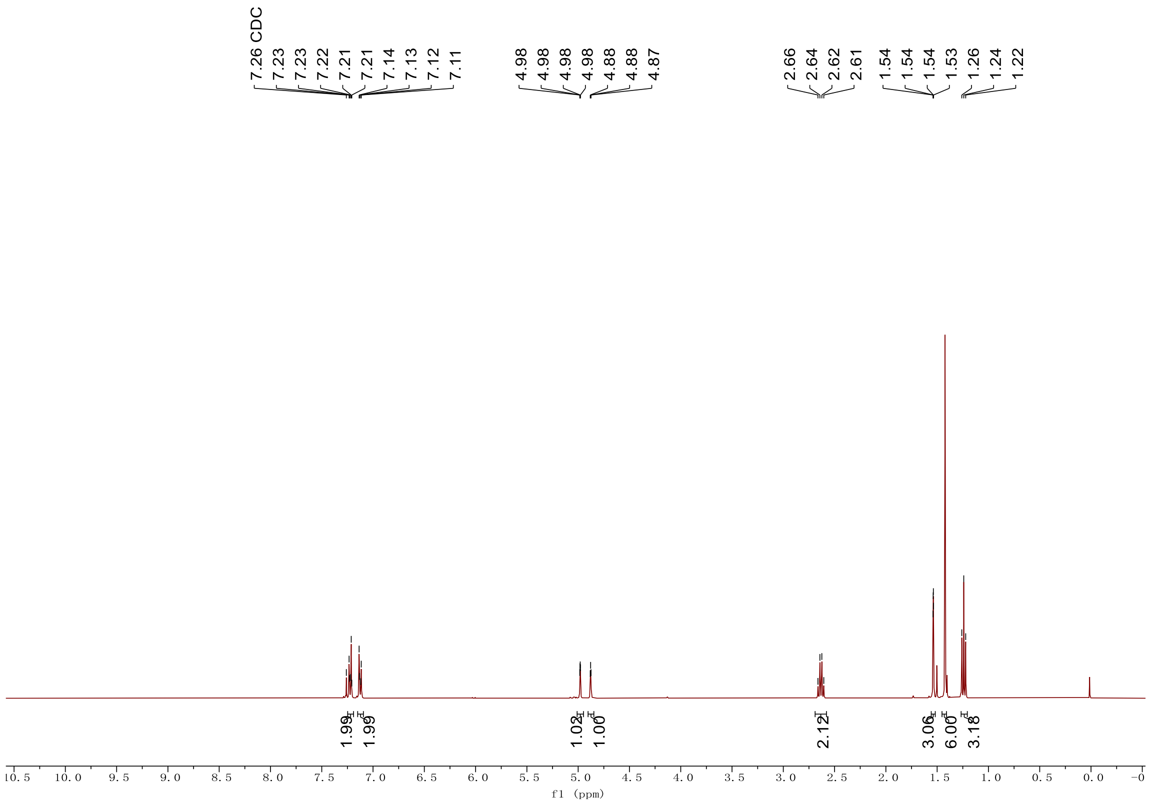

Supplementary Figure 66. **^{1}H NMR** (400 MHz, Chloroform-*d*) of **37a**

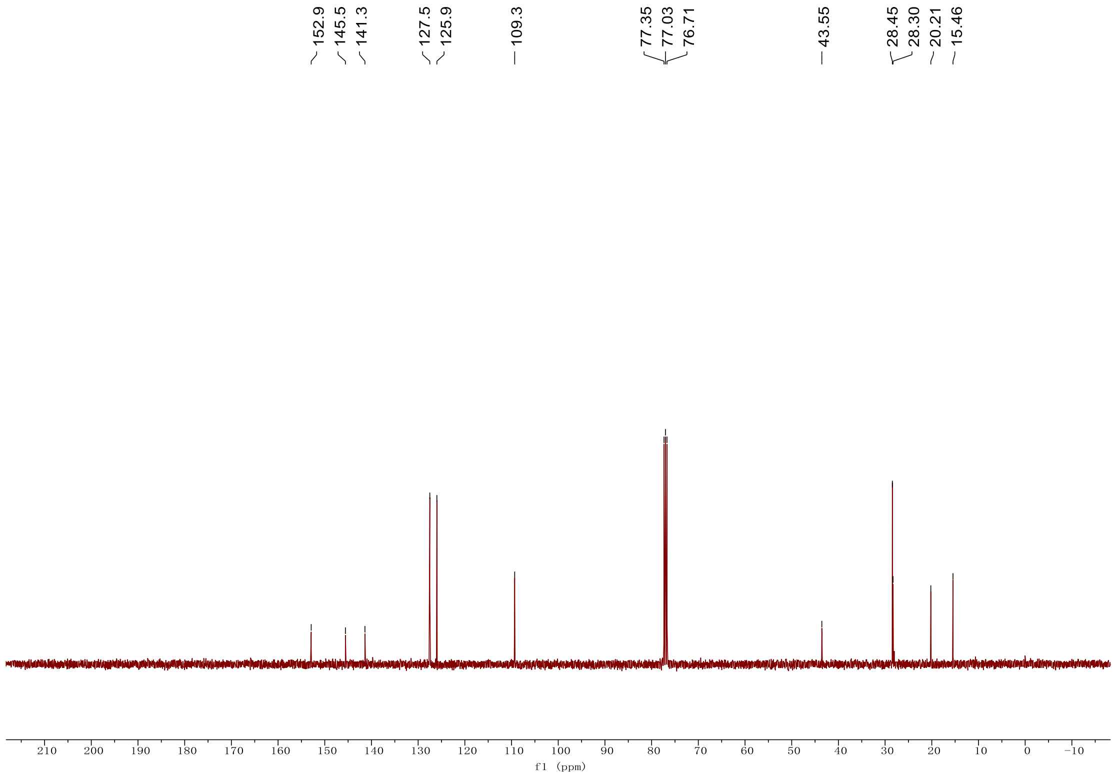

Supplementary Figure 67. **^{13}C NMR (**101 MHz, Chloroform-*d*) of **37a**

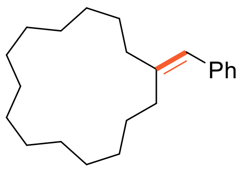

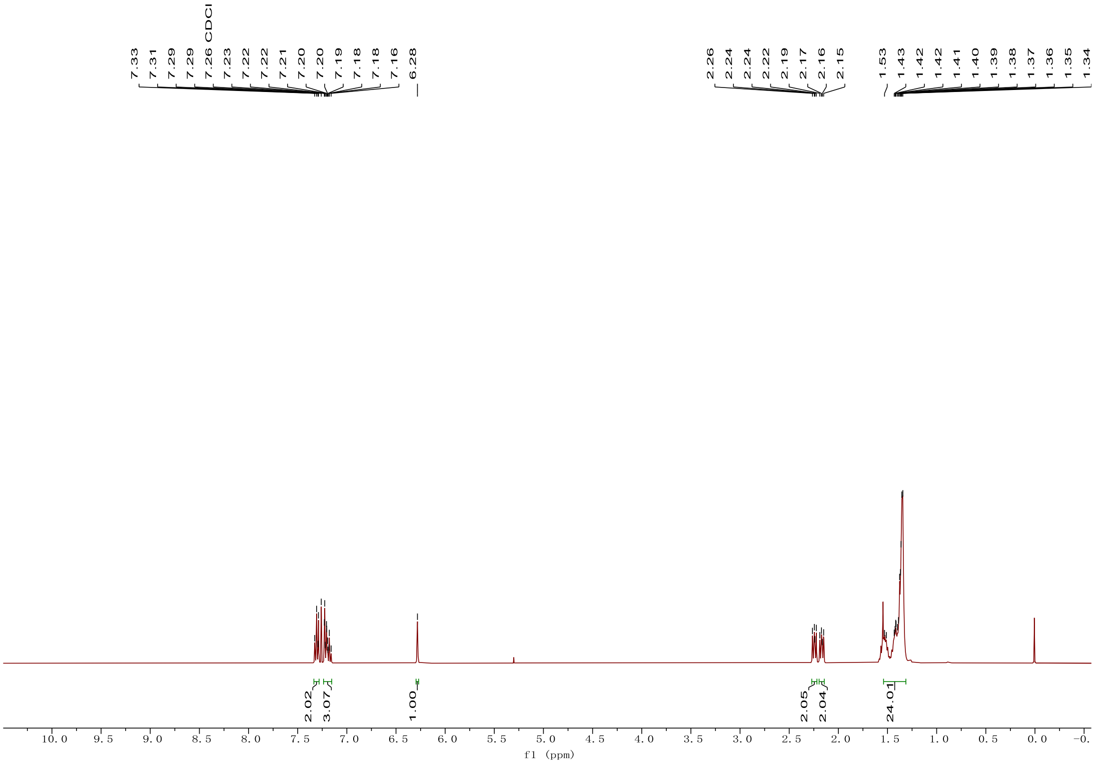

Supplementary Figure 68. ^{1}**H NMR** (400 MHz, Chloroform-*d*) of **40a**

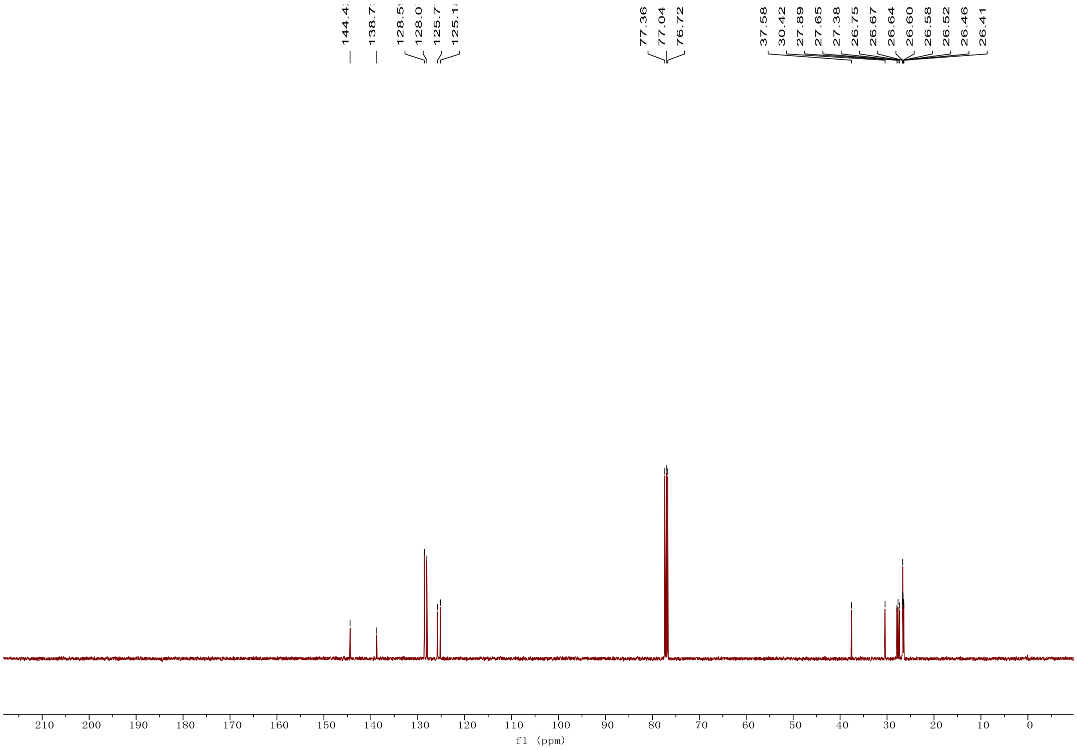

Supplementary Figure 69. ^{13}**C NMR (**101 MHz, Chloroform-*d*) of **40a**

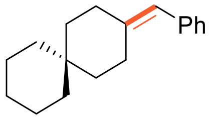

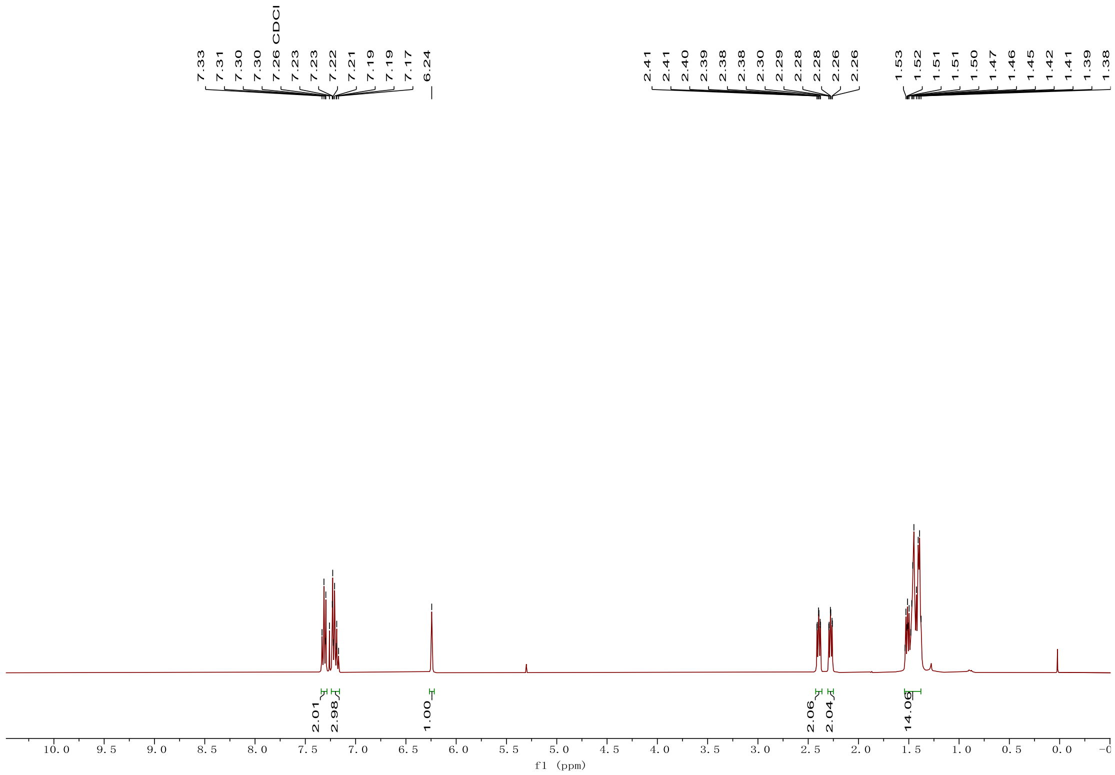

Supplementary Figure 70. ^{1}**H NMR** (400 MHz, Chloroform-*d*) of **41a**

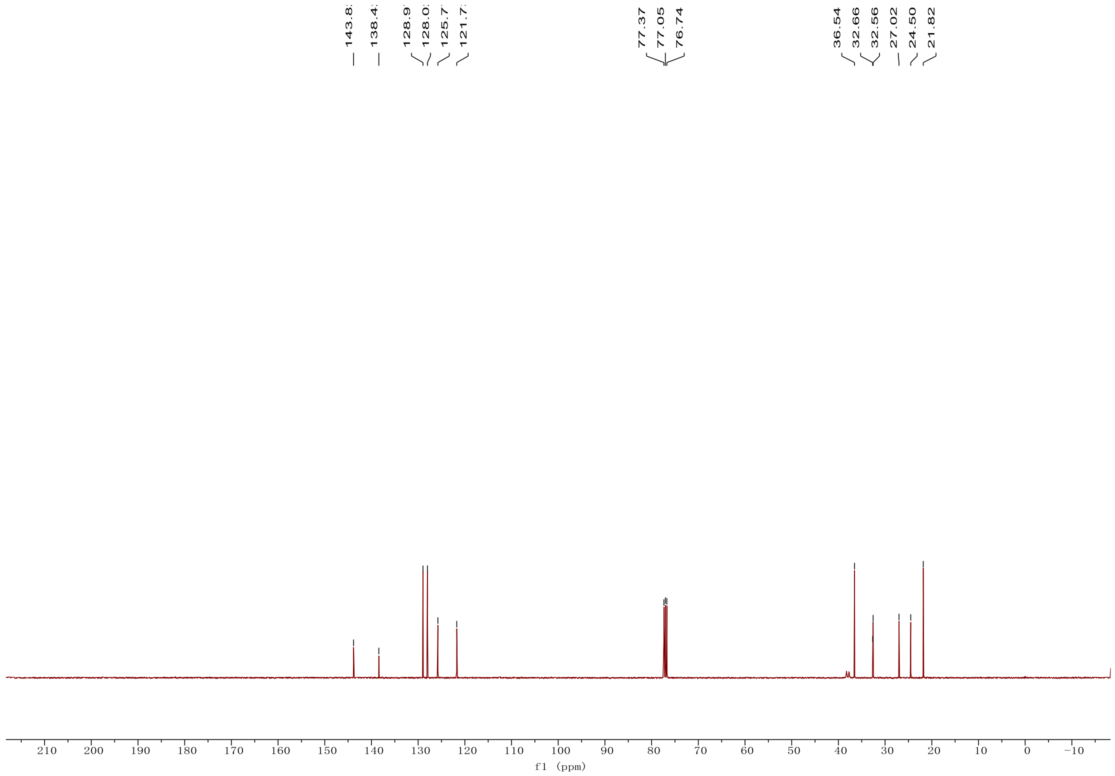

Supplementary Figure 71. ^{13}**C NMR (**101 MHz, Chloroform-*d*) of **41a**

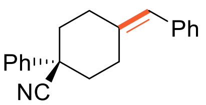

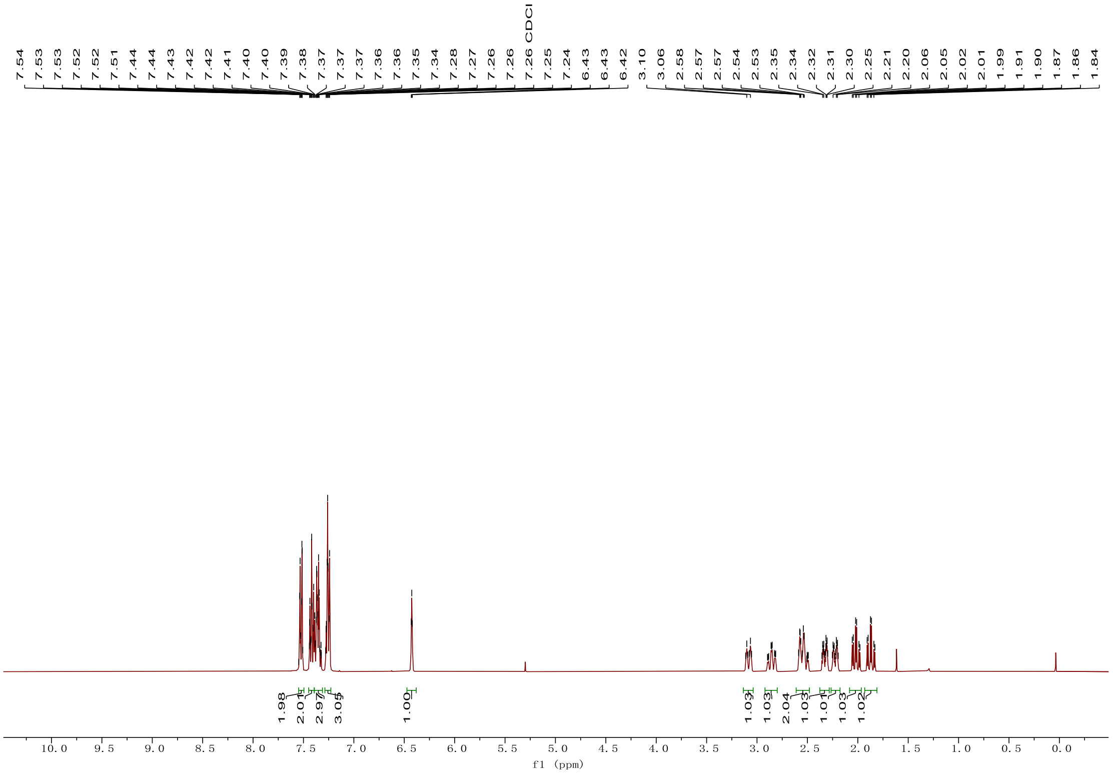

Supplementary Figure 72. ^{1}**H NMR** (400 MHz, Chloroform-*d*) of **43a**

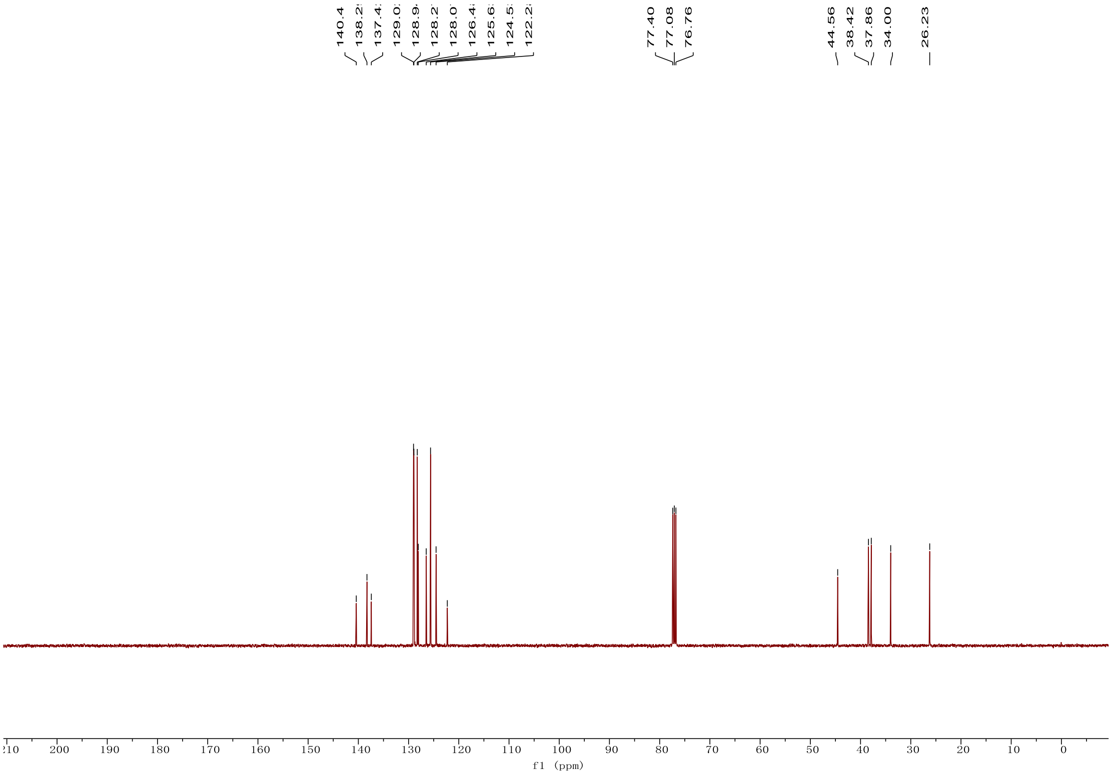

Supplementary Figure 73. ^{13}**C NMR (**101 MHz, Chloroform-*d*) of **43a**

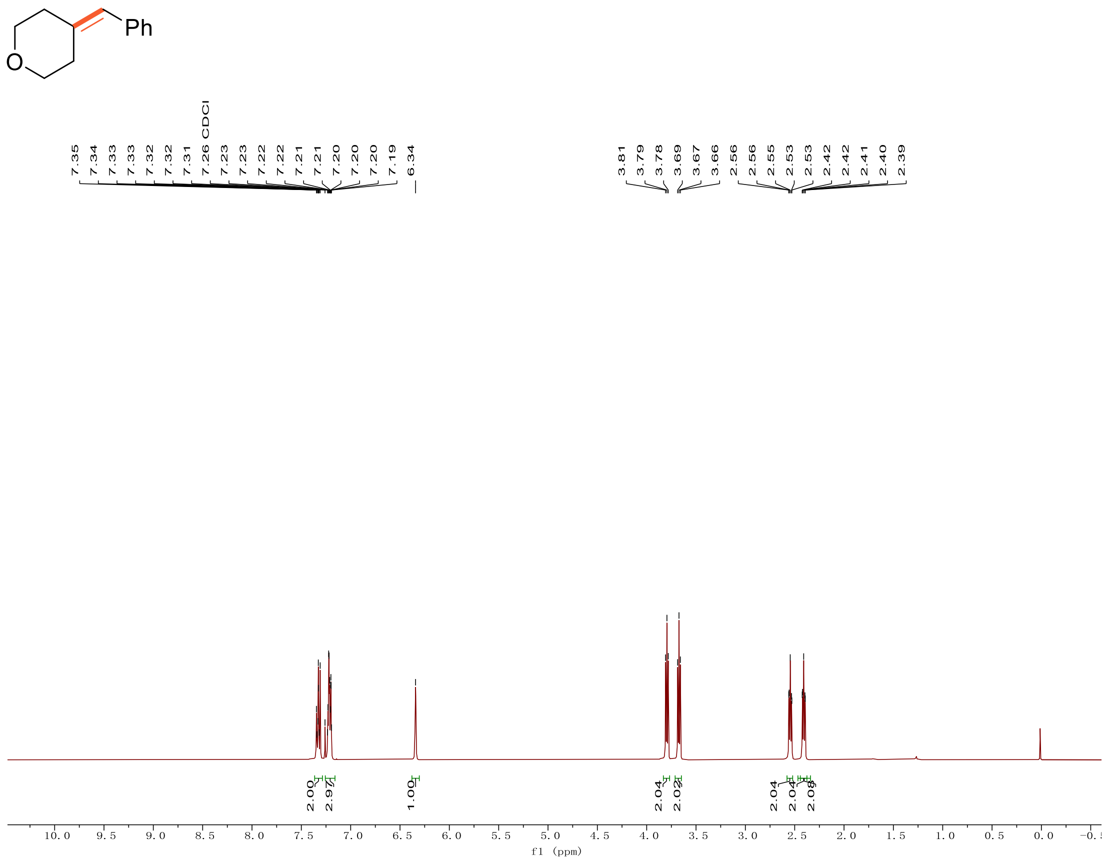

Supplementary Figure 74. ¹H NMR (400 MHz, Chloroform-*d*) of **45a**

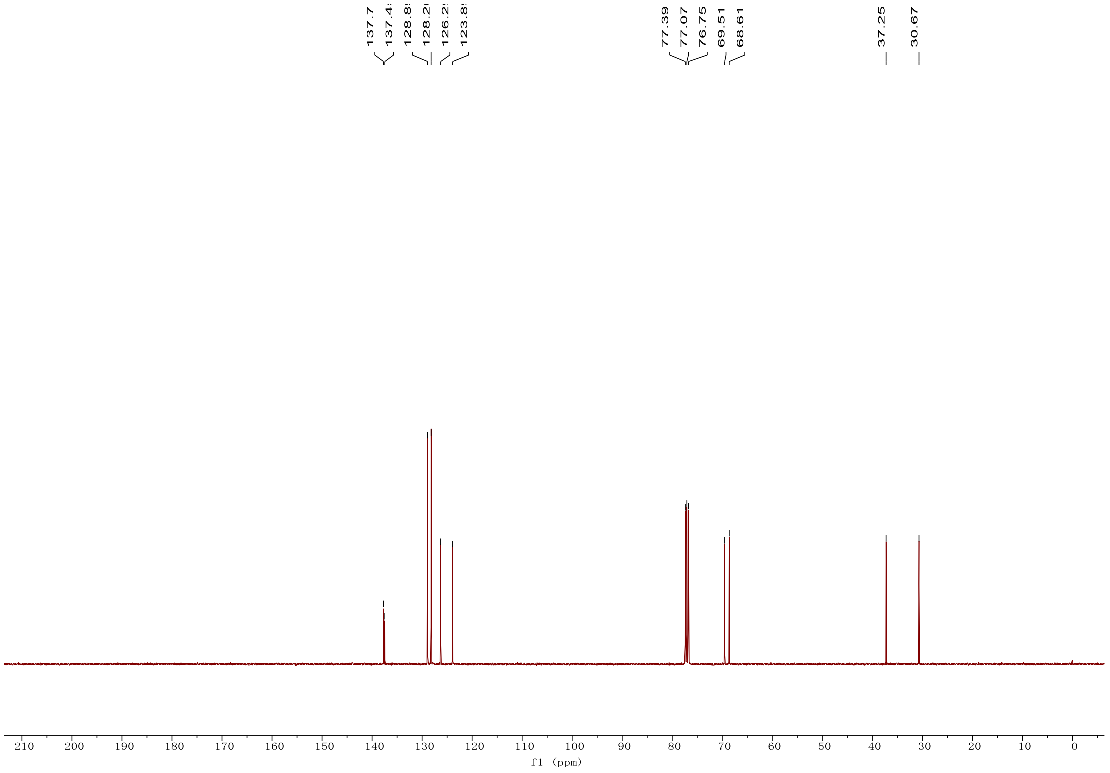

Supplementary Figure 75. ¹³C NMR (101 MHz, Chloroform-*d*) of **45a**

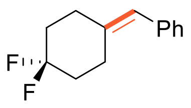

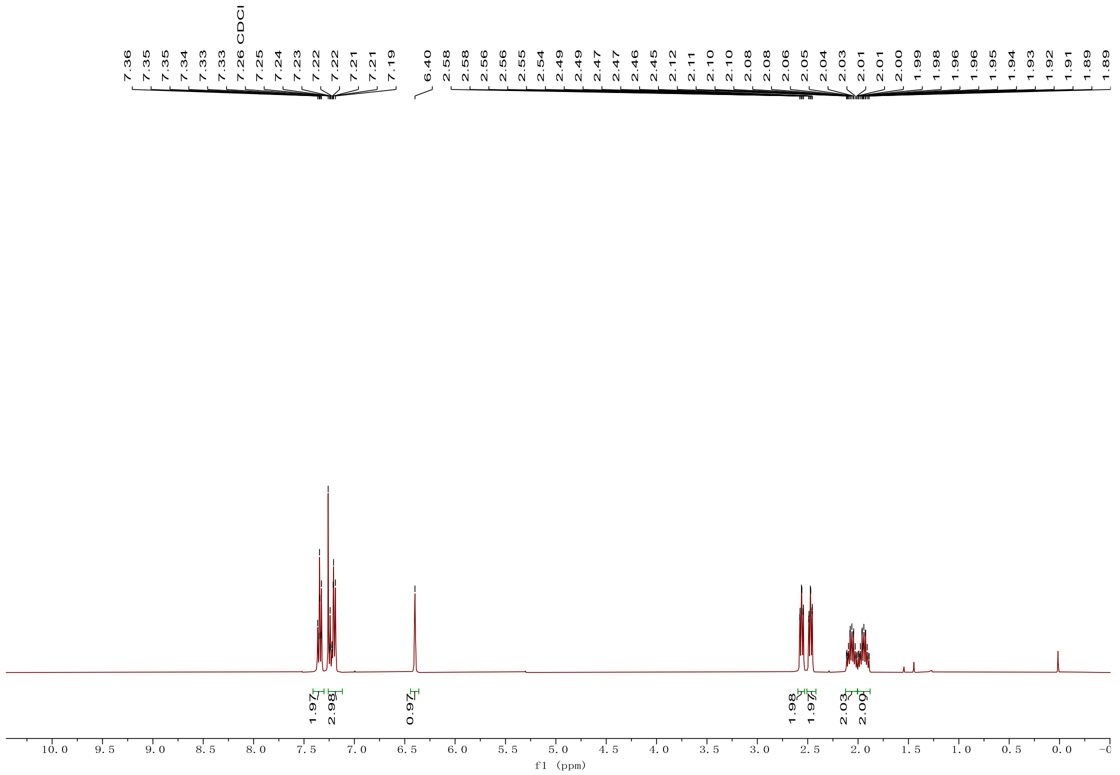

Supplementary Figure 76. ^{1}H NMR (400 MHz, Chloroform-*d*) of **46a**

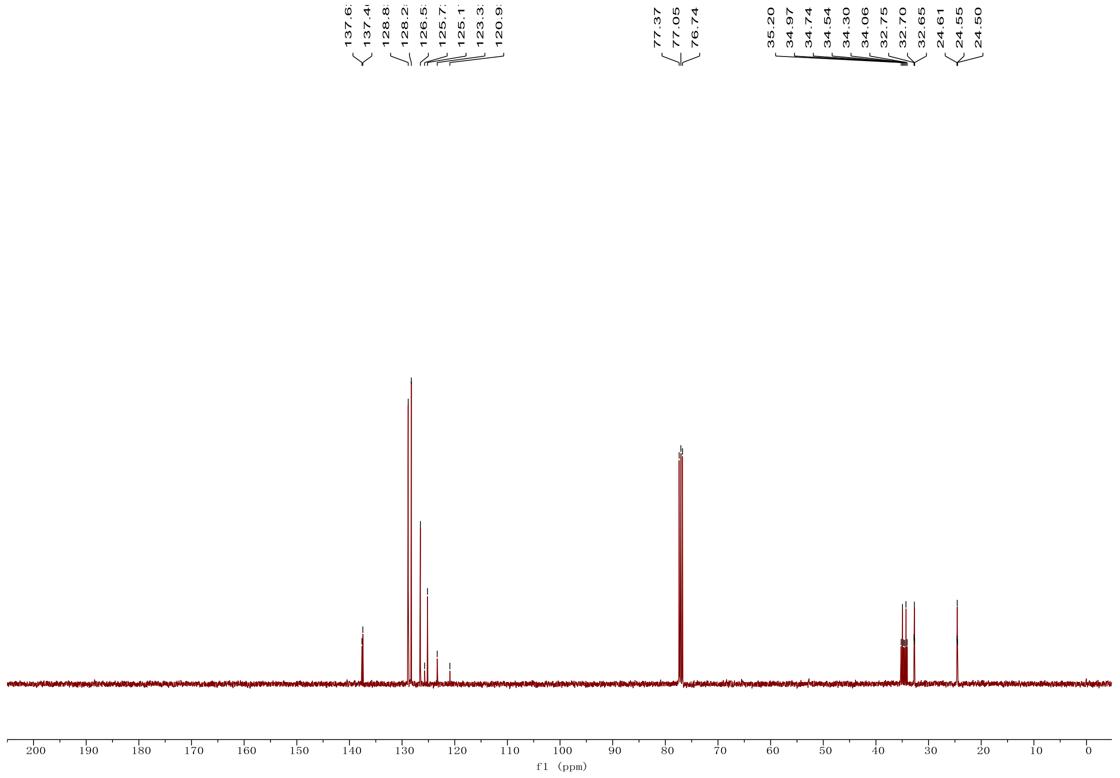

Supplementary Figure 77. ^{13}**C NMR (**101 MHz, Chloroform-*d*) of **46a**

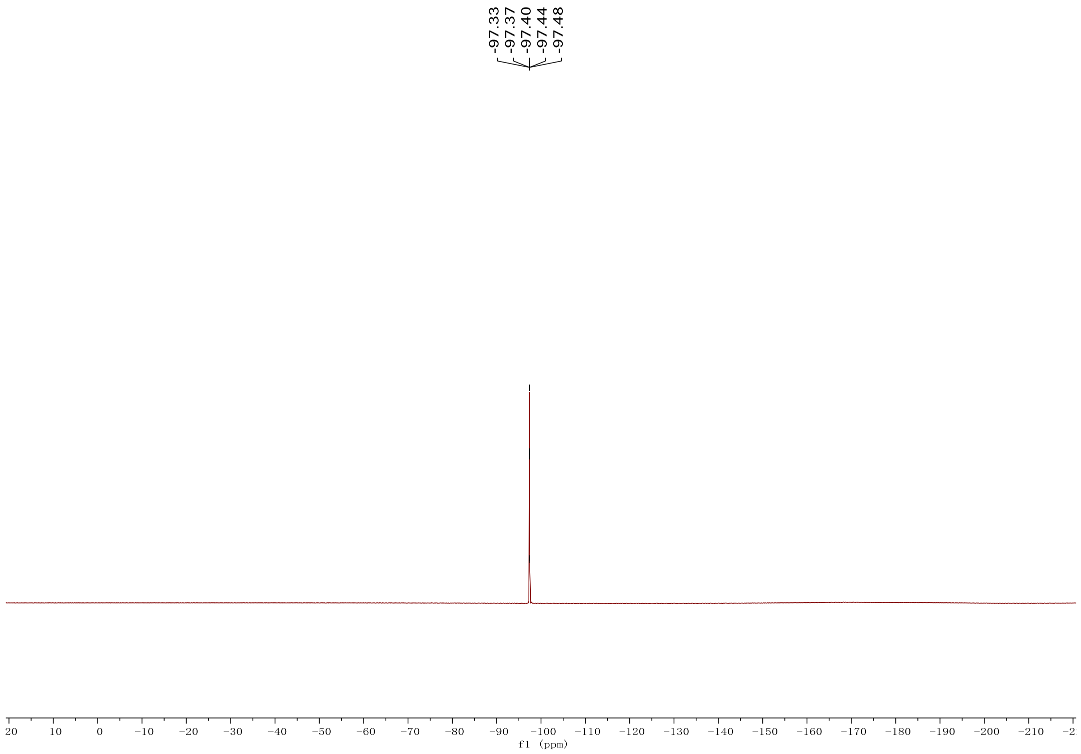

Supplementary Figure 78. ^{19}F NMR (376 MHz, Chloroform-*d*) of **46a**

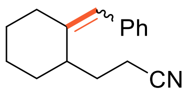

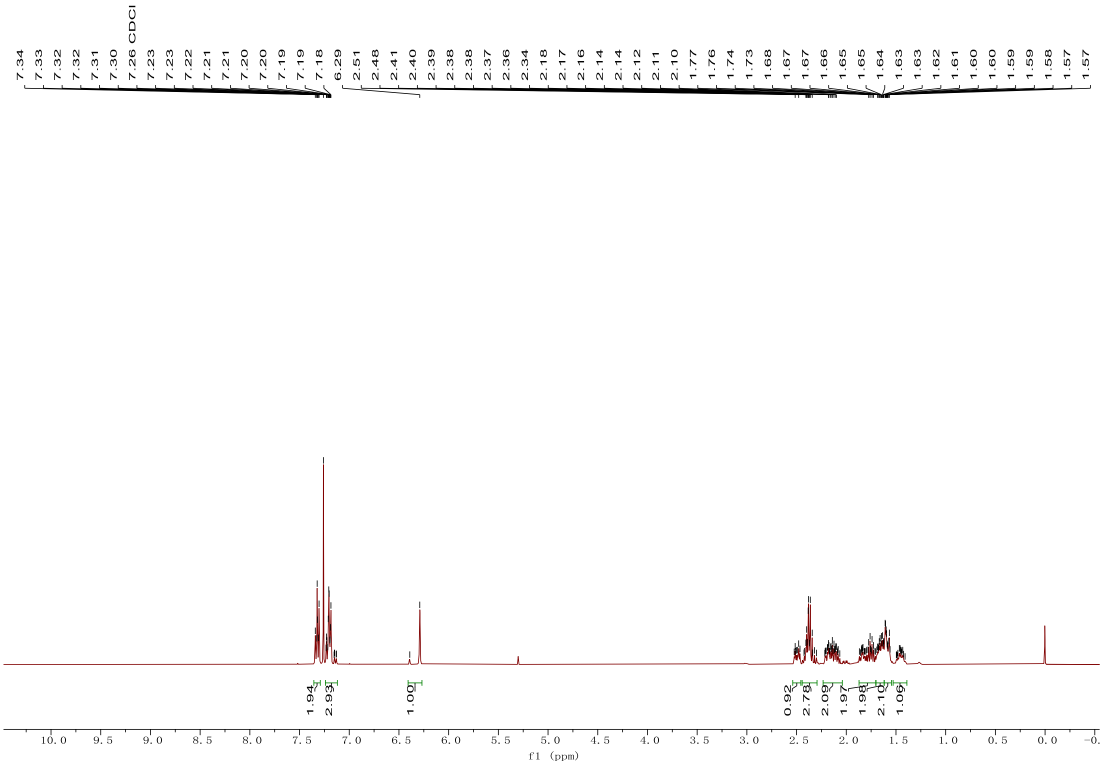

Supplementary Figure 79. ^{1}H NMR (400 MHz, Chloroform-*d*) of **47a**

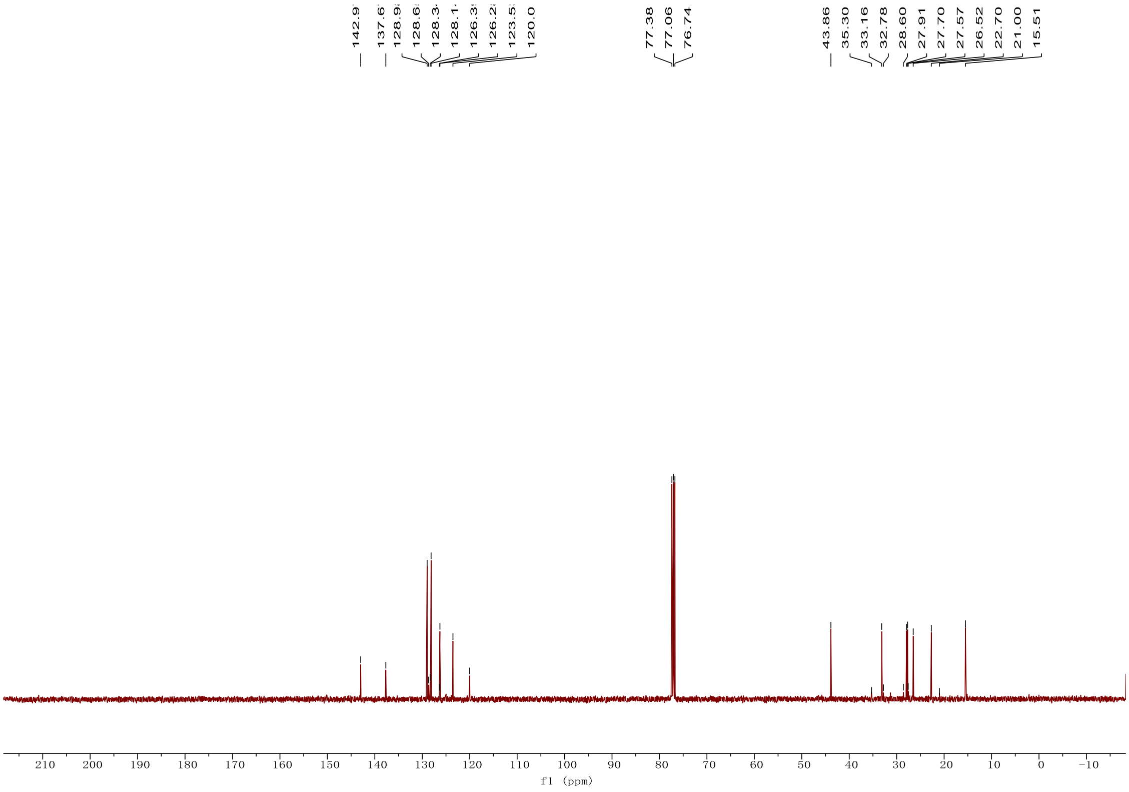

Supplementary Figure 80. ^{13}C NMR (101 MHz, Chloroform-*d*) of **47a**

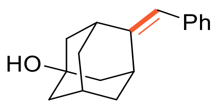

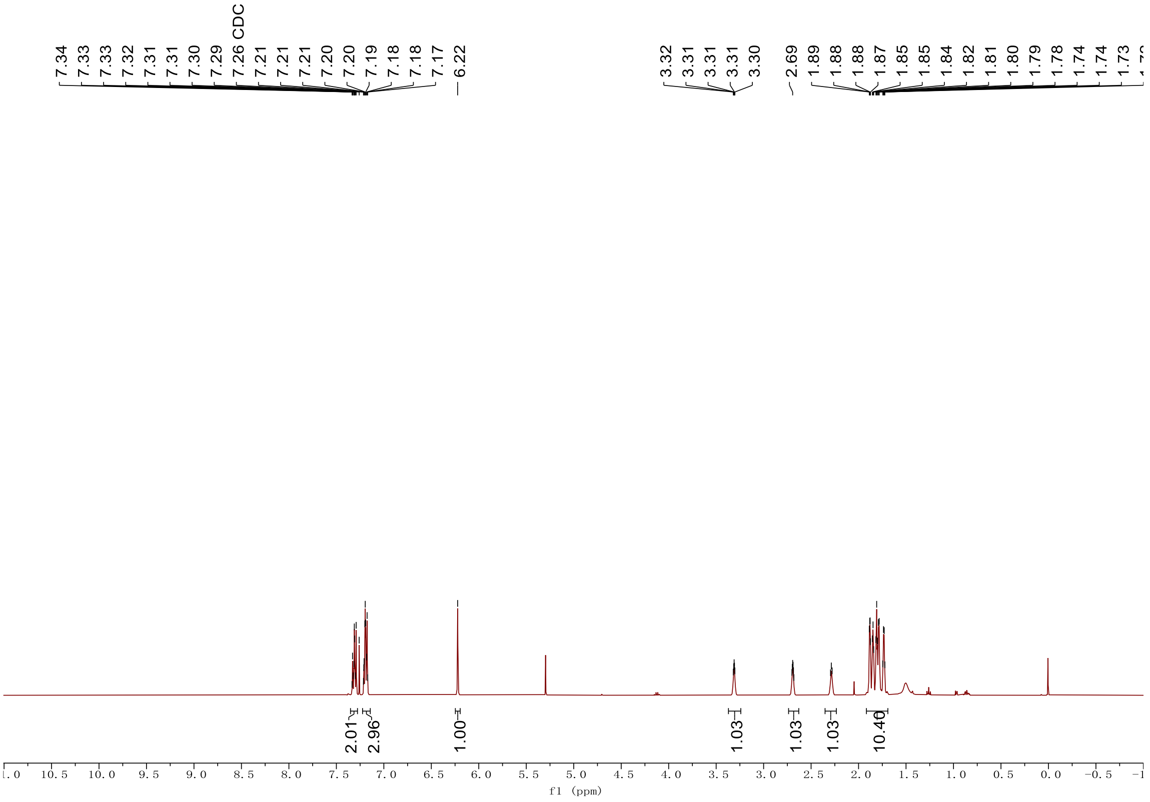

Supplementary Figure 81. ^{1}**H NMR** (400 MHz, Chloroform-*d*) of **48a**

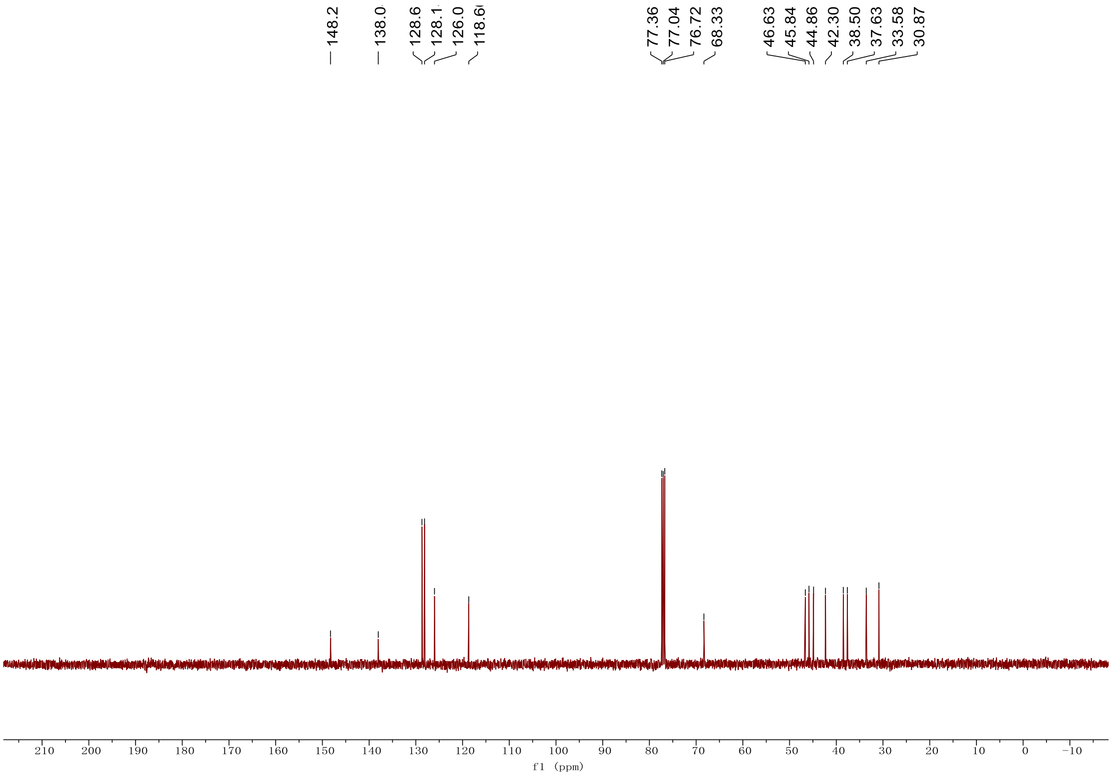

Supplementary Figure 82. ^{13}**C NMR** (101 MHz, Chloroform-*d*) of **48a**

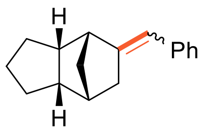

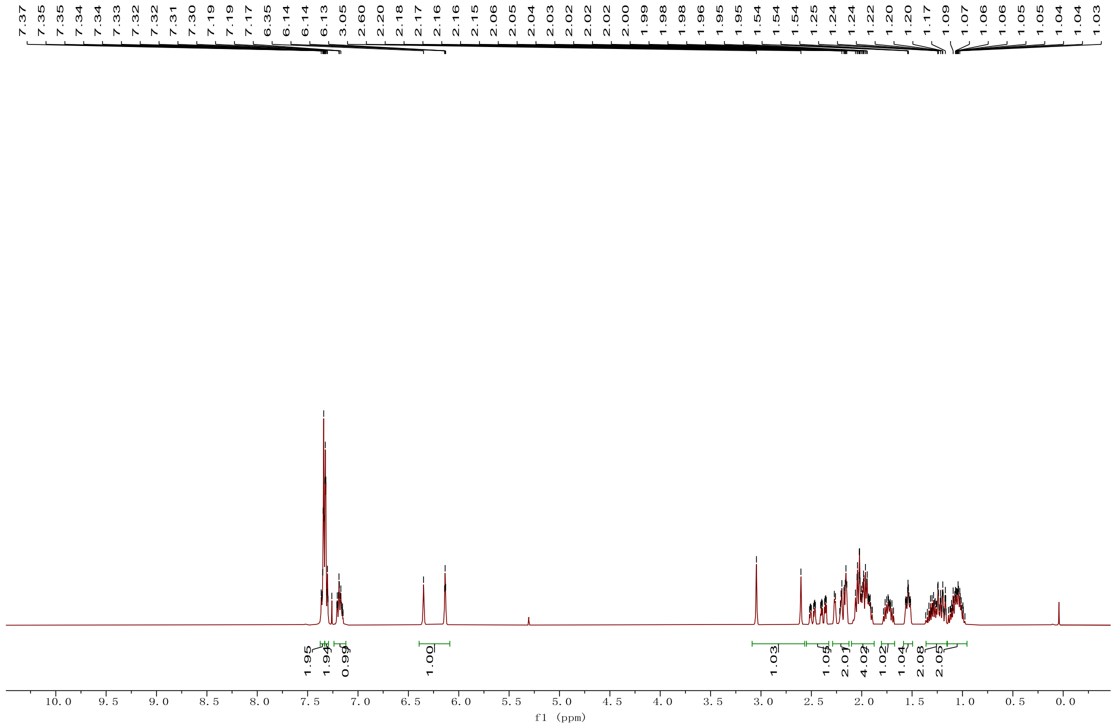

Supplementary Figure 83. ^{1}**H NMR** (400 MHz, Chloroform-*d*) of **49a**

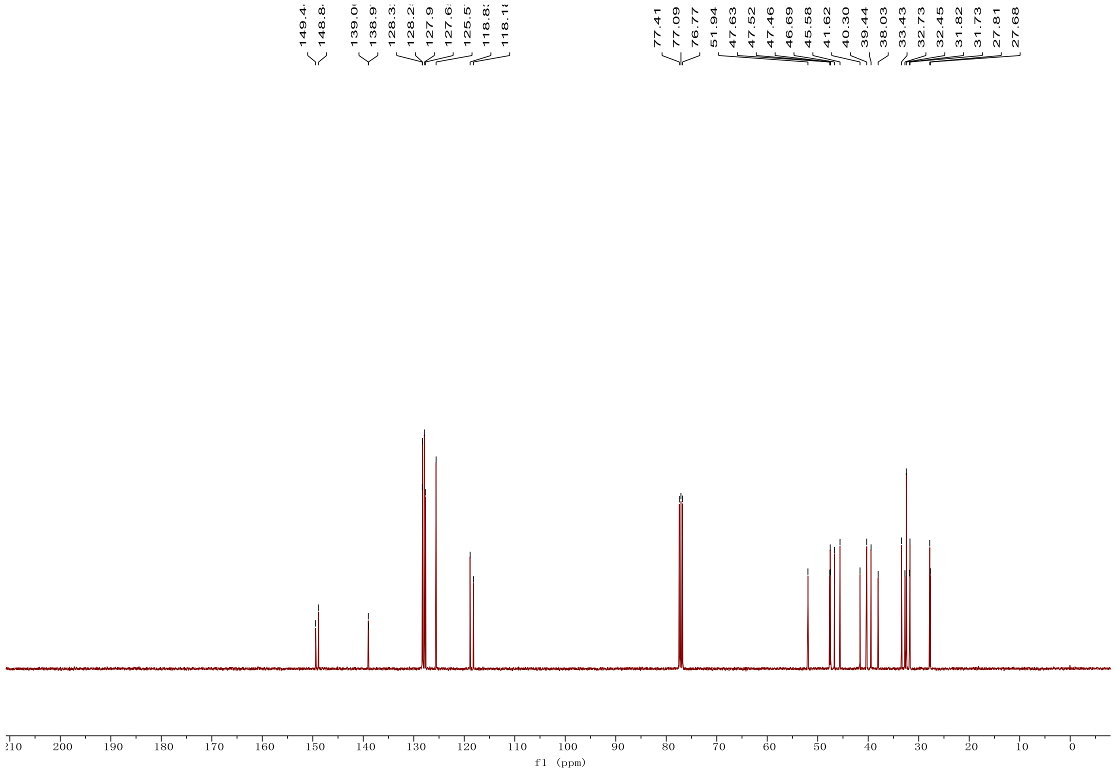

Supplementary Figure 84. ^{13}**C NMR (**101 MHz, Chloroform-*d*) of **49a**

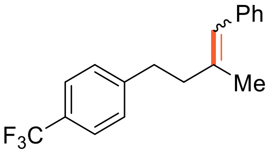

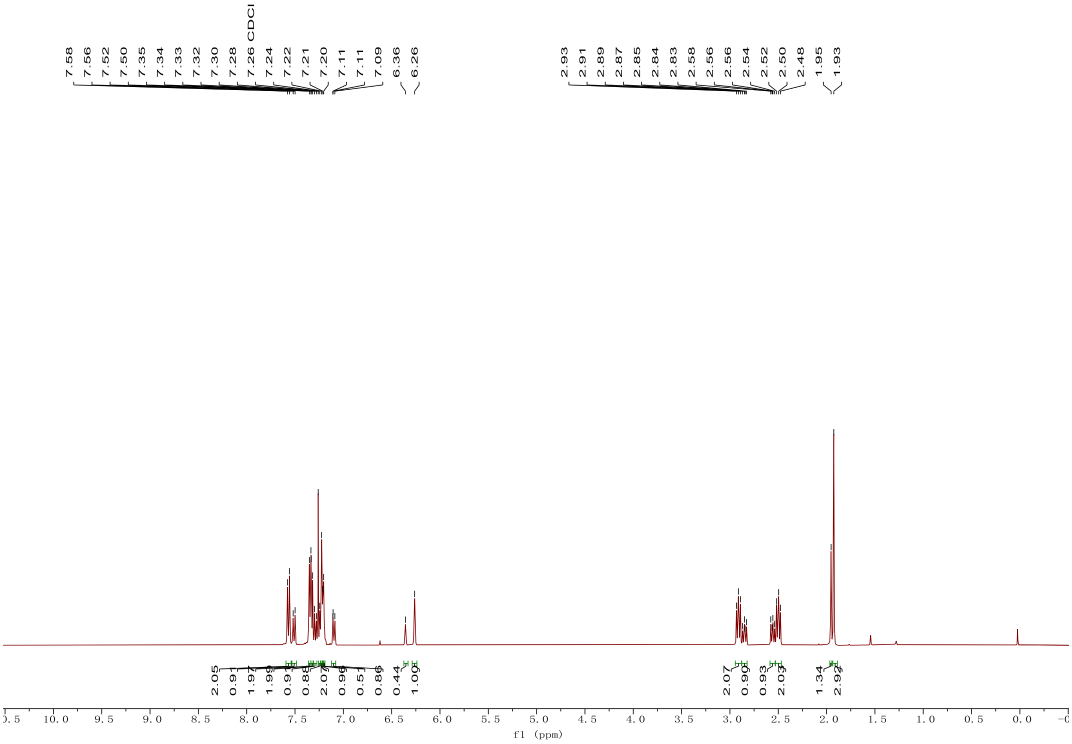

Supplementary Figure 85. ^{1}H NMR (400 MHz, Chloroform-*d*) of **51a**

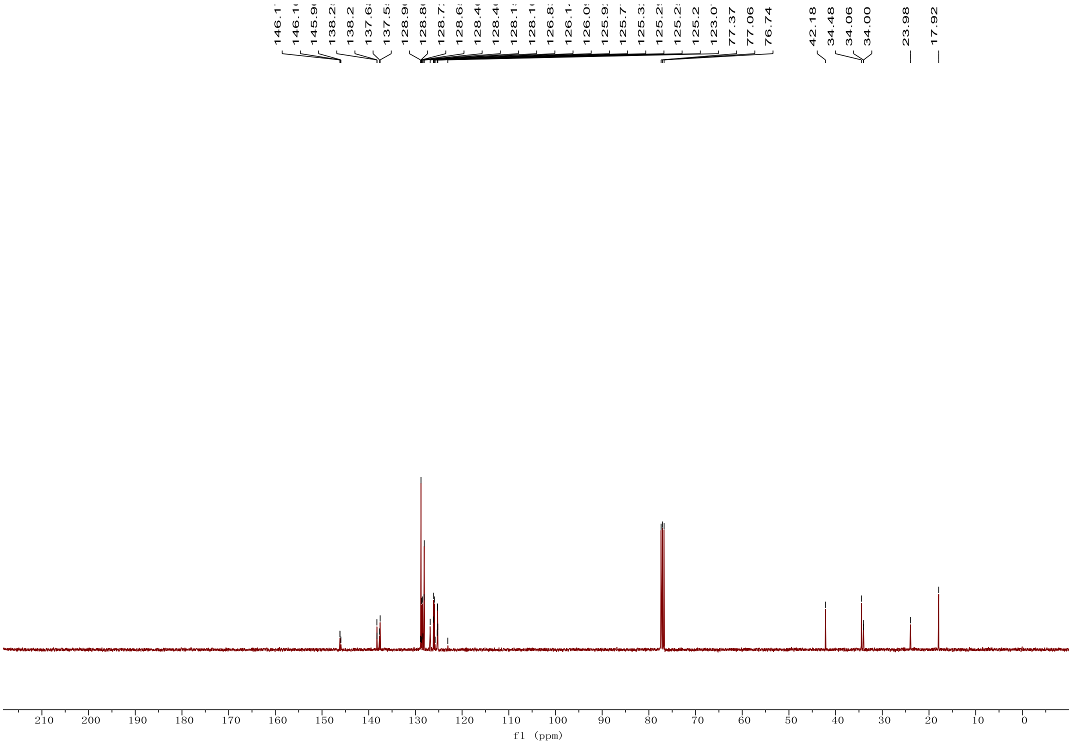

Supplementary Figure 86. ^{13}C NMR (101 MHz, Chloroform-*d*) of **51a**

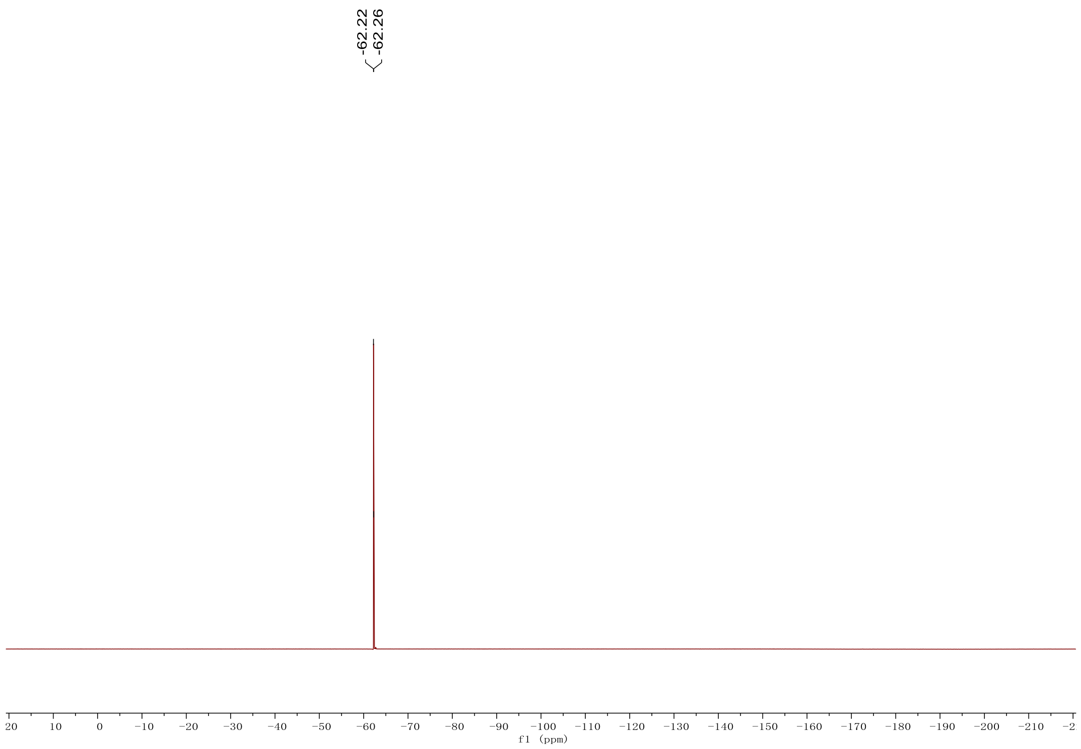

Supplementary Figure 87. ^{19}F NMR (376 MHz, Chloroform-*d*) of **51a**

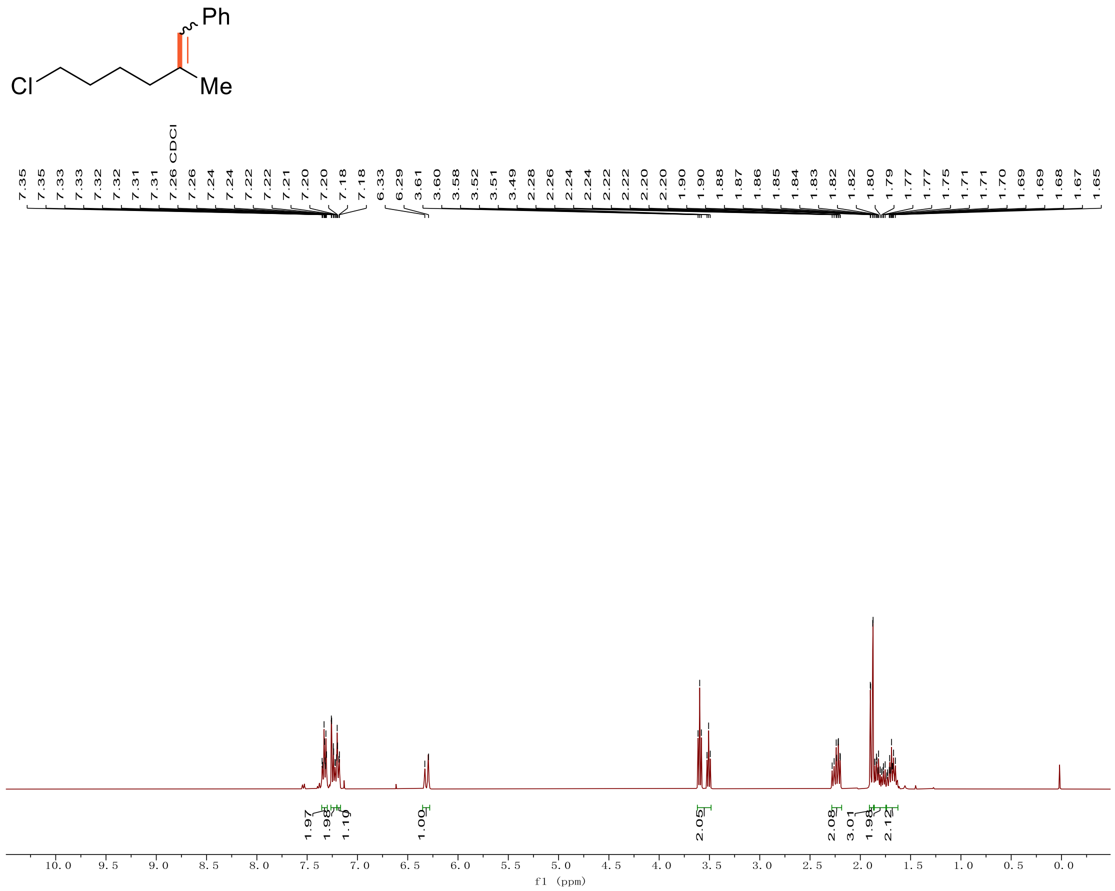

Supplementary Figure 88. ^{1}**H NMR** (400 MHz, Chloroform-*d*) of **52a**

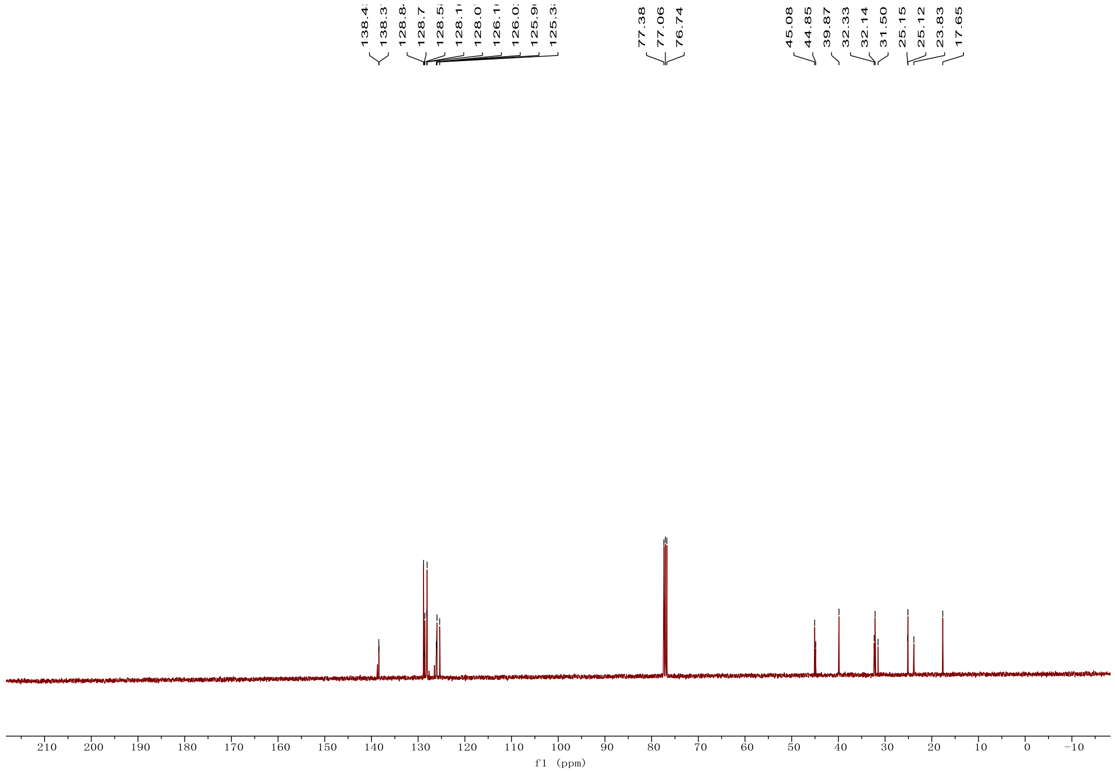

Supplementary Figure 89. ^{13}**C NMR (**101 MHz, Chloroform-*d*) of **52a**

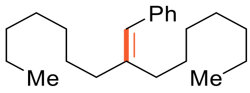

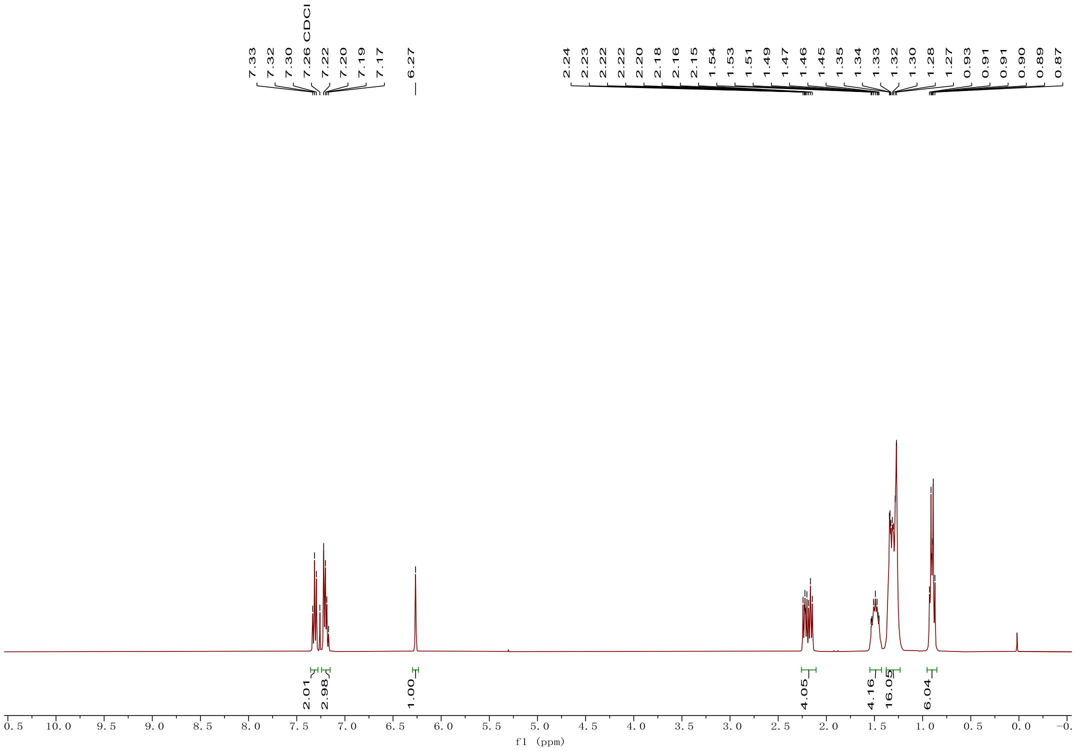

Supplementary Figure 90. ^{1}H NMR (400 MHz, Chloroform-*d*) of **54a**

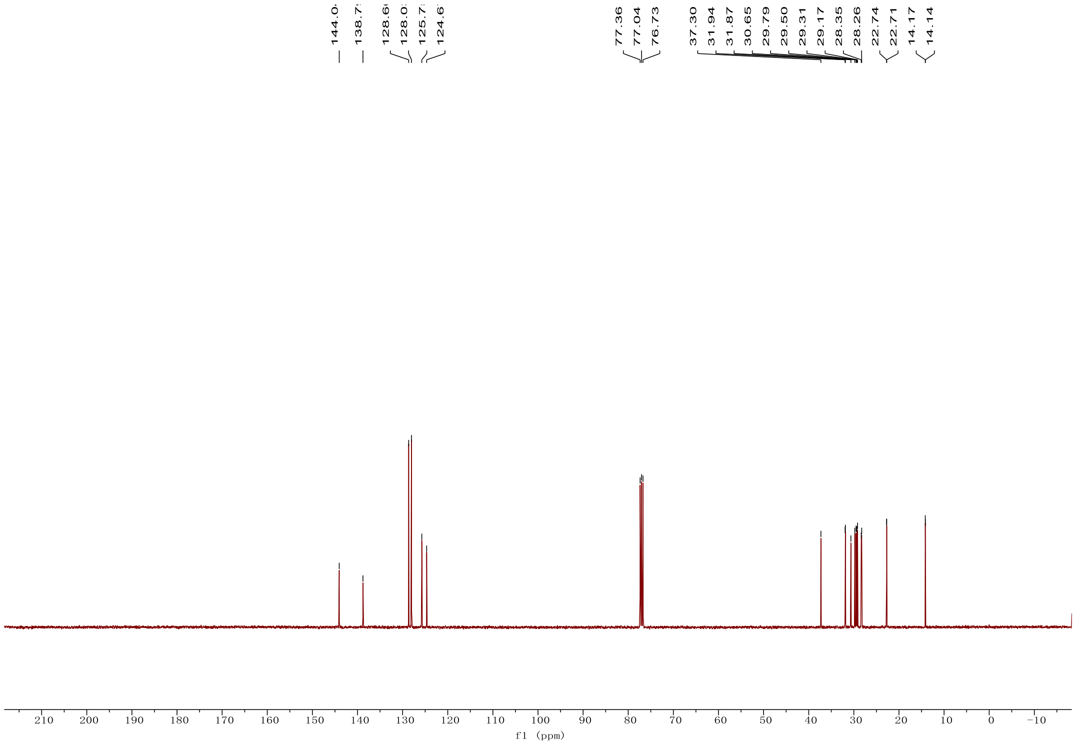

Supplementary Figure 91. ^{13}C NMR (101 MHz, Chloroform-*d*) of **54a**

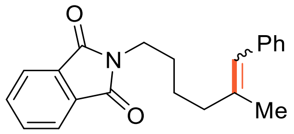

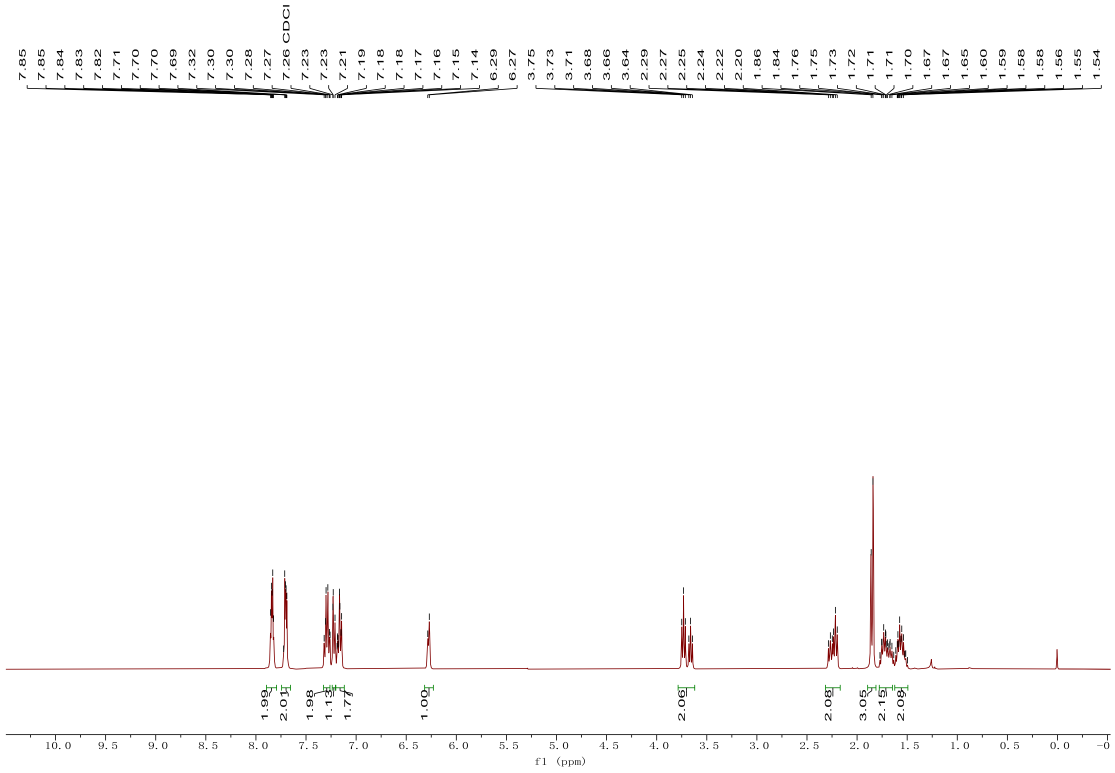

Supplementary Figure 92. ^{1}**H NMR** (400 MHz, Chloroform-*d*) of **57a**

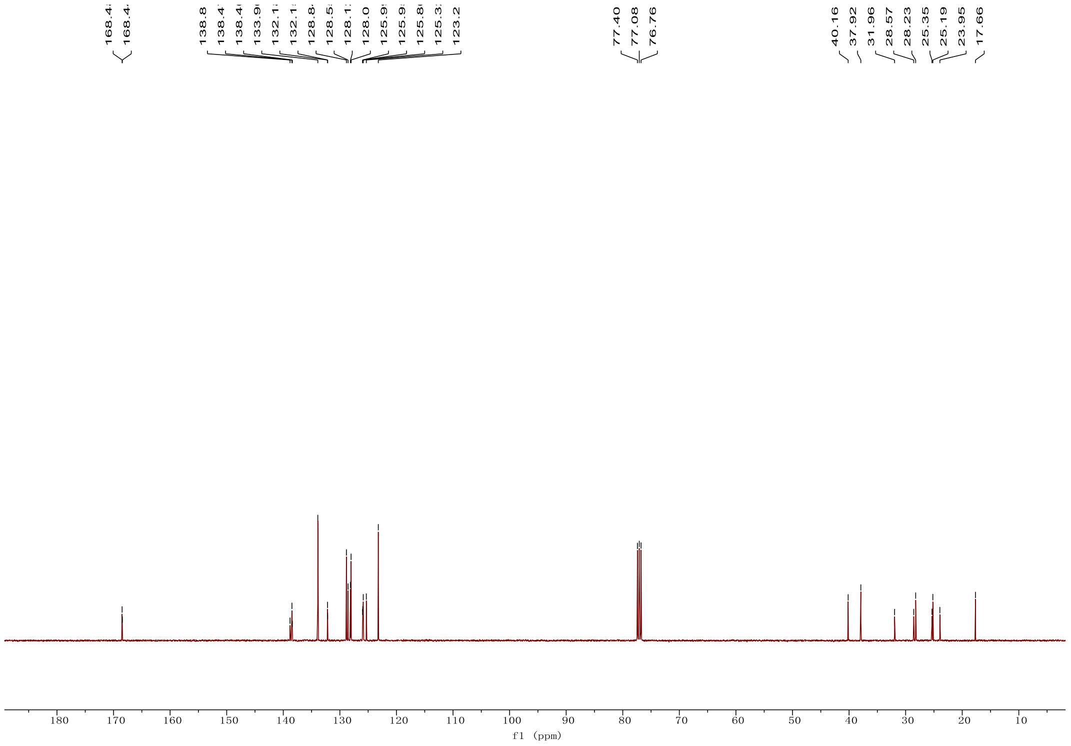

Supplementary Figure 93. ^{13}**C NMR (**101 MHz, Chloroform-*d*) of **57a**

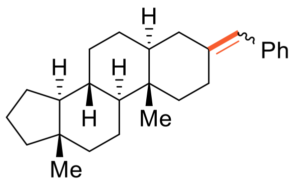

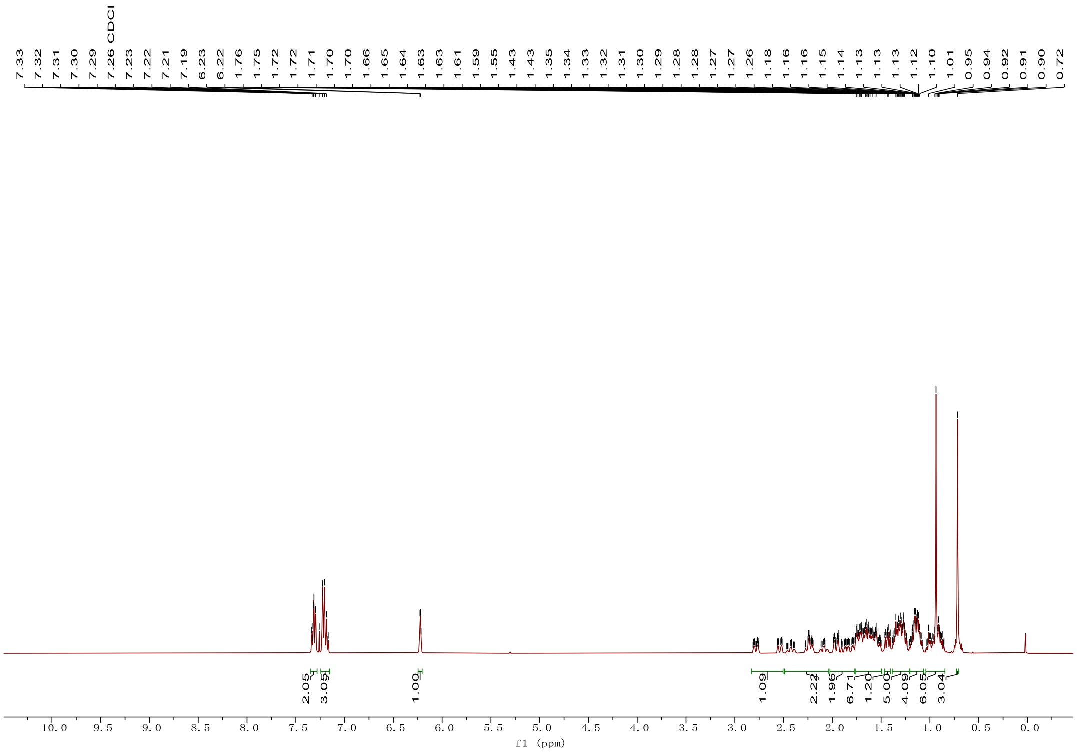

Supplementary Figure 94. **^{1}H NMR** (400 MHz, Chloroform-*d*) of **58a**

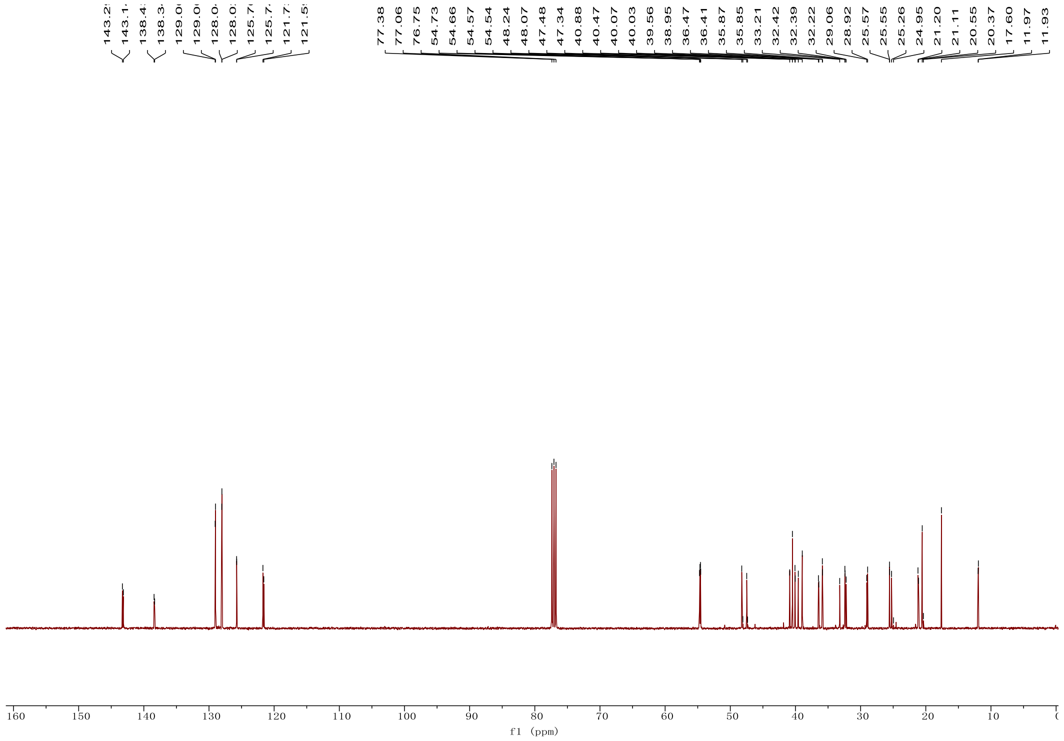

Supplementary Figure 95. **^{13}C NMR (**101 MHz, Chloroform-*d*) of **58a**

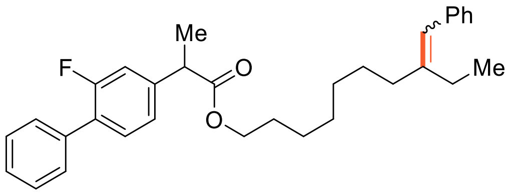

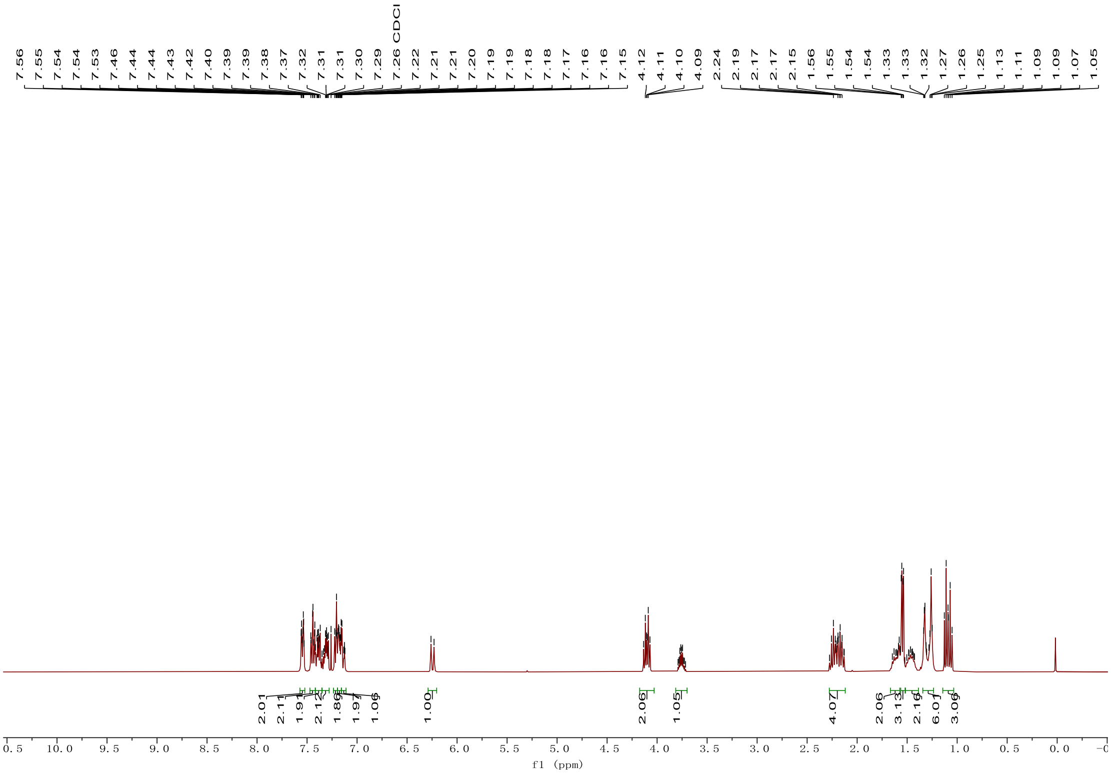

Supplementary Figure 96. ^{1}H NMR (400 MHz, Chloroform-*d*) of **59a**

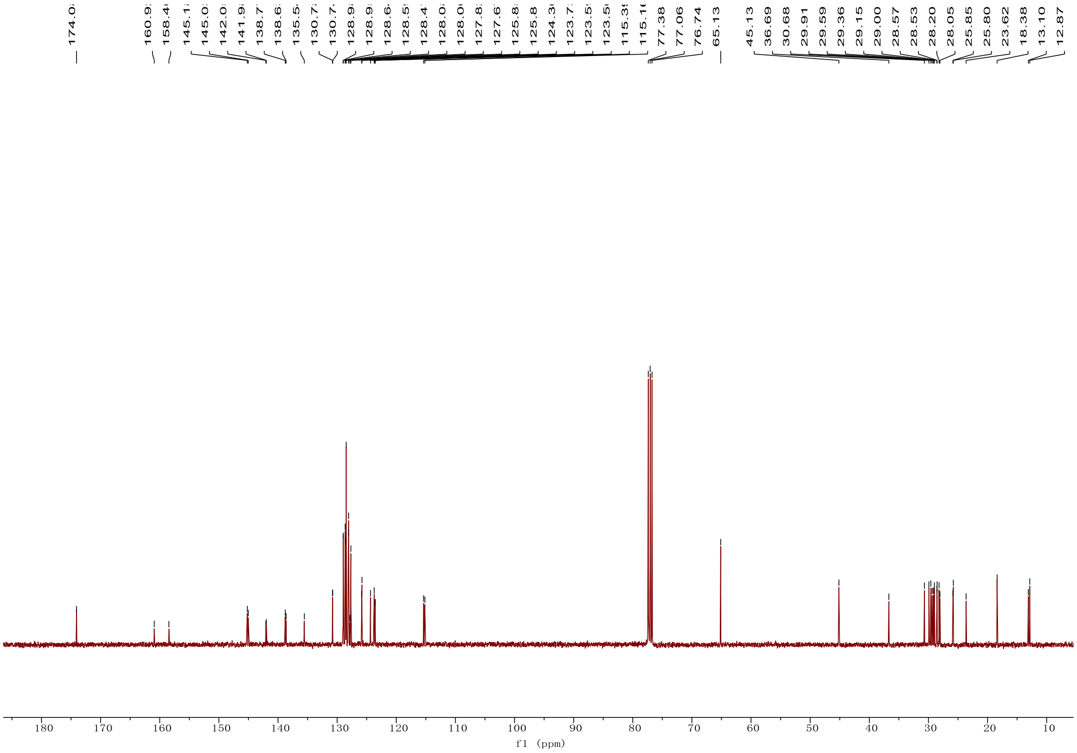

Supplementary Figure 97. ^{13}C NMR (101 MHz, Chloroform-*d*) of **59a**

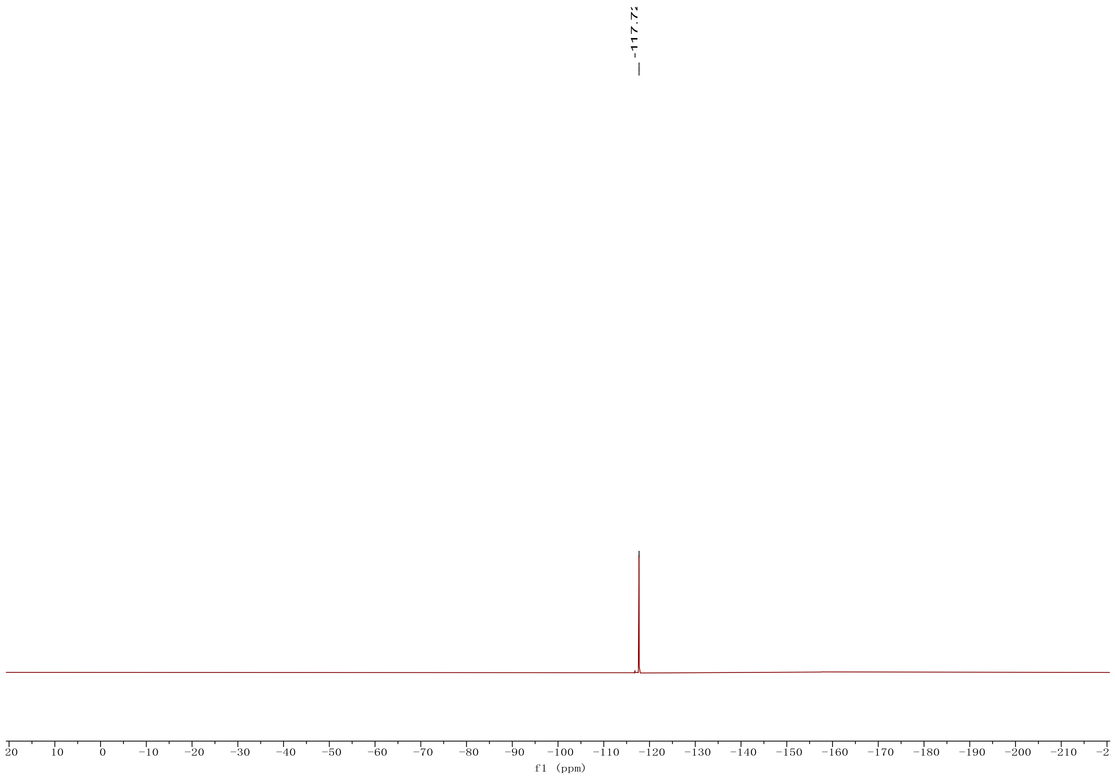

Supplementary Figure 98. ^{19}F NMR (376 MHz, Chloroform-*d*) of **59a**

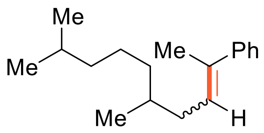

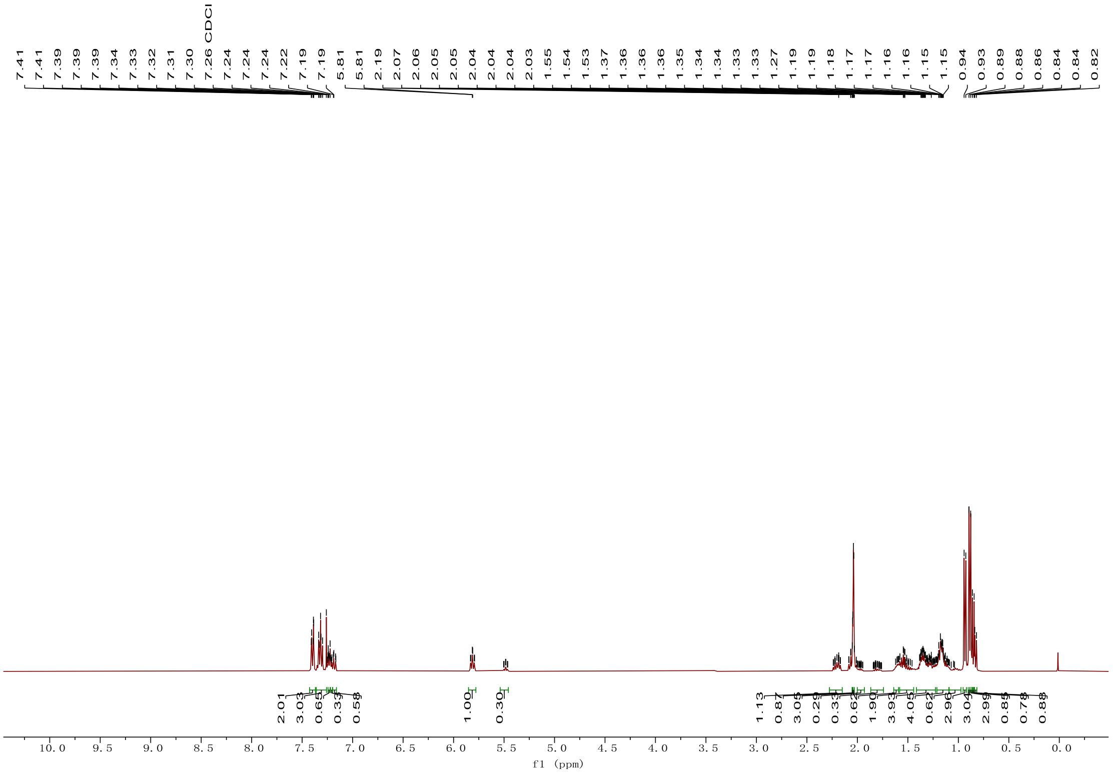

Supplementary Figure 99. ^{1}H NMR (400 MHz, Chloroform-*d*) of **61a**

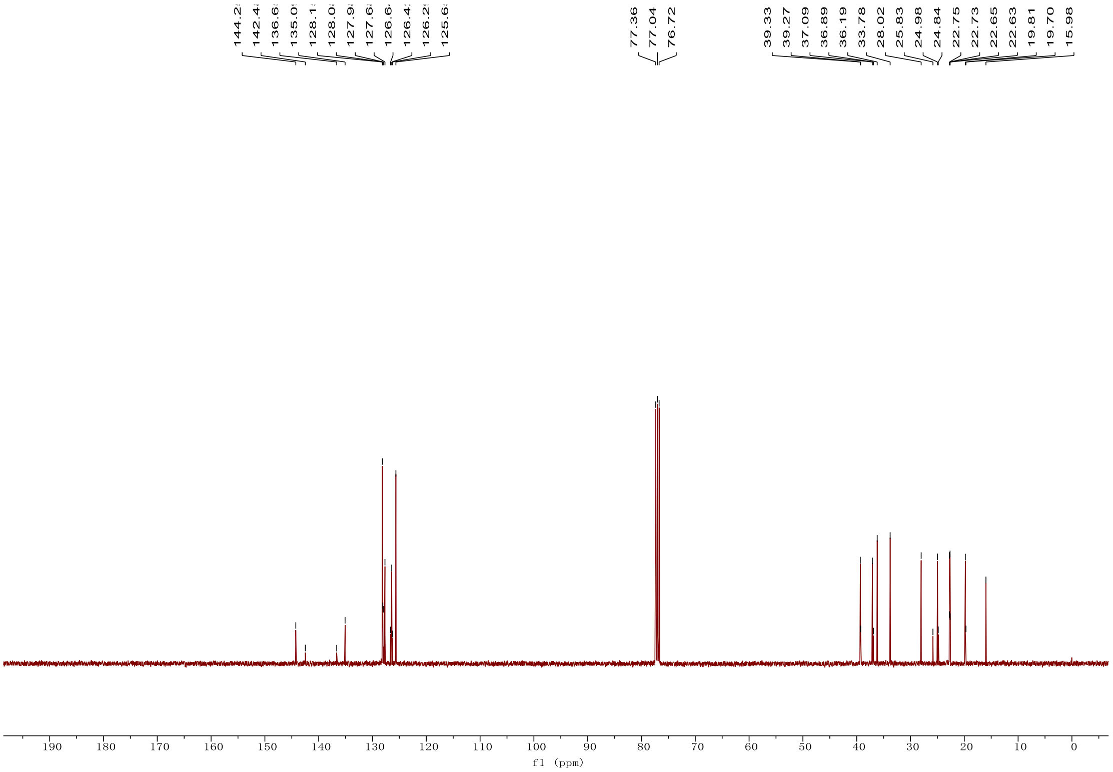

Supplementary Figure 100. ^{13}C NMR (101 MHz, Chloroform-*d*) of **61a**

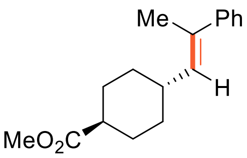

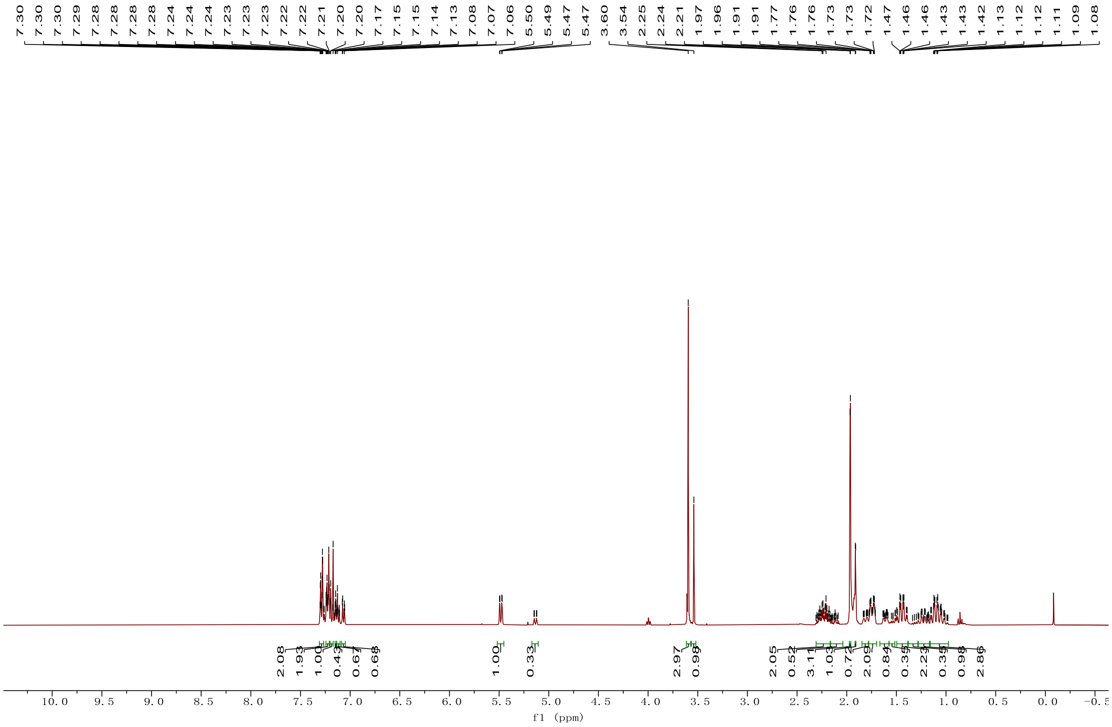

Supplementary Figure 101. ^{1}**H NMR** (400 MHz, Chloroform-*d*) of **62a**

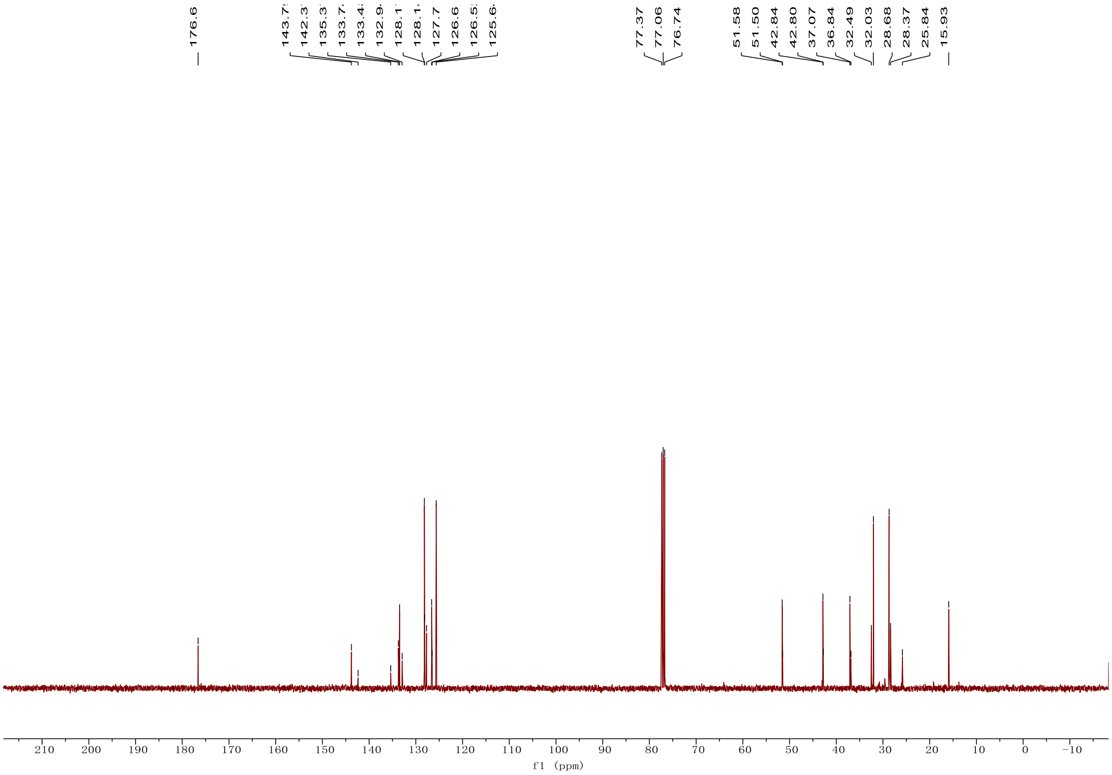

Supplementary Figure 102. ^{13}**C NMR (**101 MHz, Chloroform-*d*) of **62a**

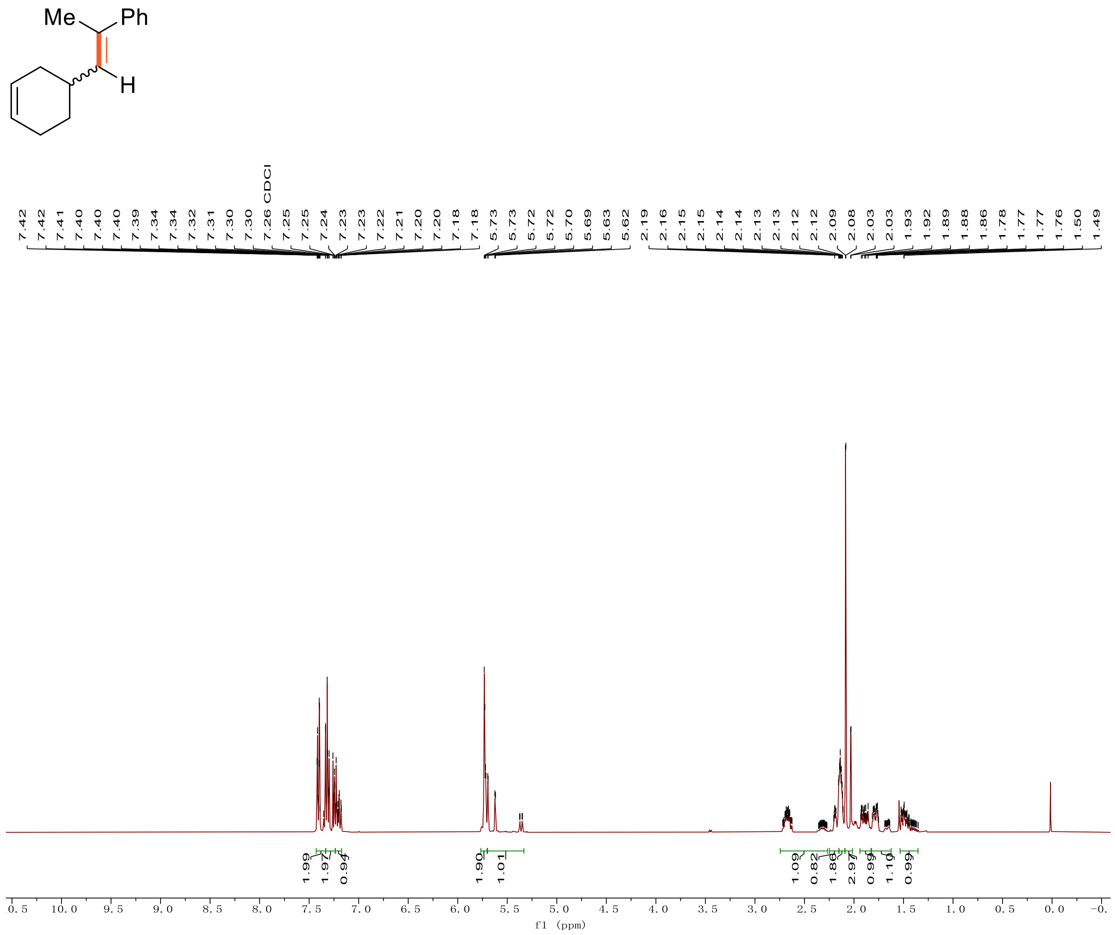

Supplementary Figure 103. ^{1}H NMR (400 MHz, Chloroform-*d*) of **64a**

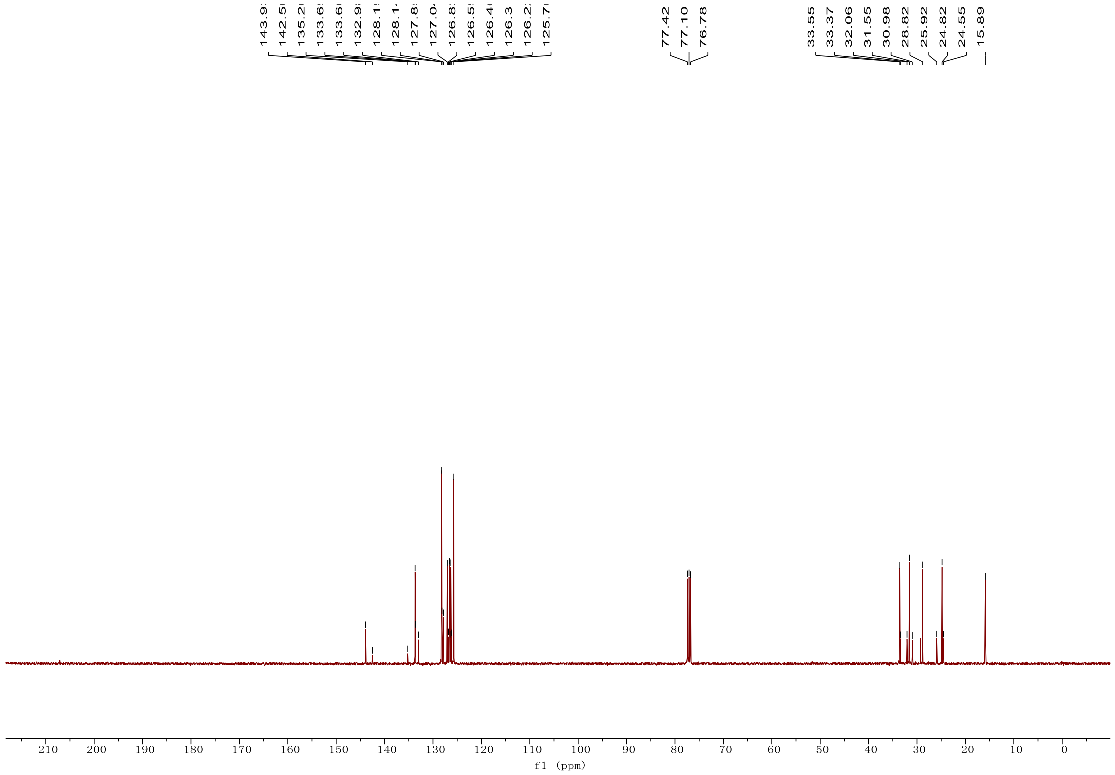

Supplementary Figure 104. ^{13}C NMR (101 MHz, Chloroform-*d*) of **64a**

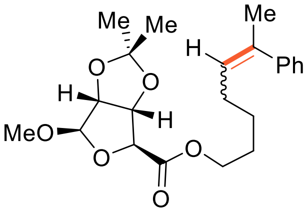

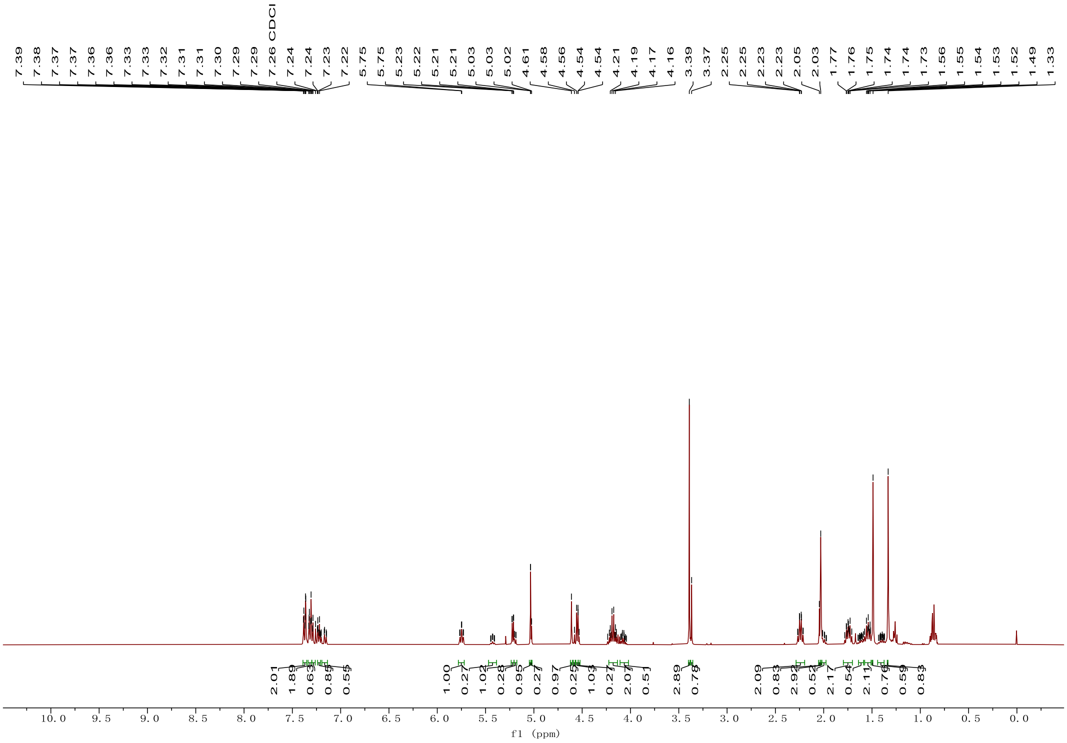

Supplementary Figure 105. ^{1}H NMR (400 MHz, Chloroform-*d*) of **65a**

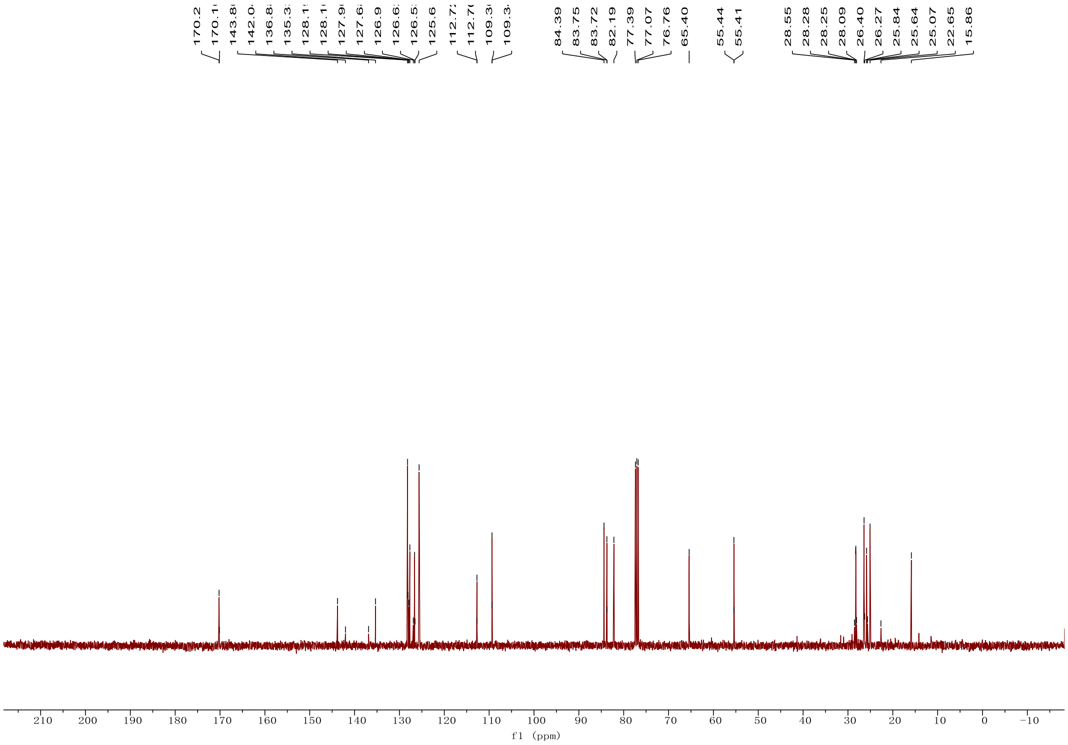

Supplementary Figure 106. ^{13}C NMR (101 MHz, Chloroform-*d*) of **65a**

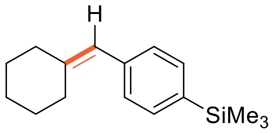

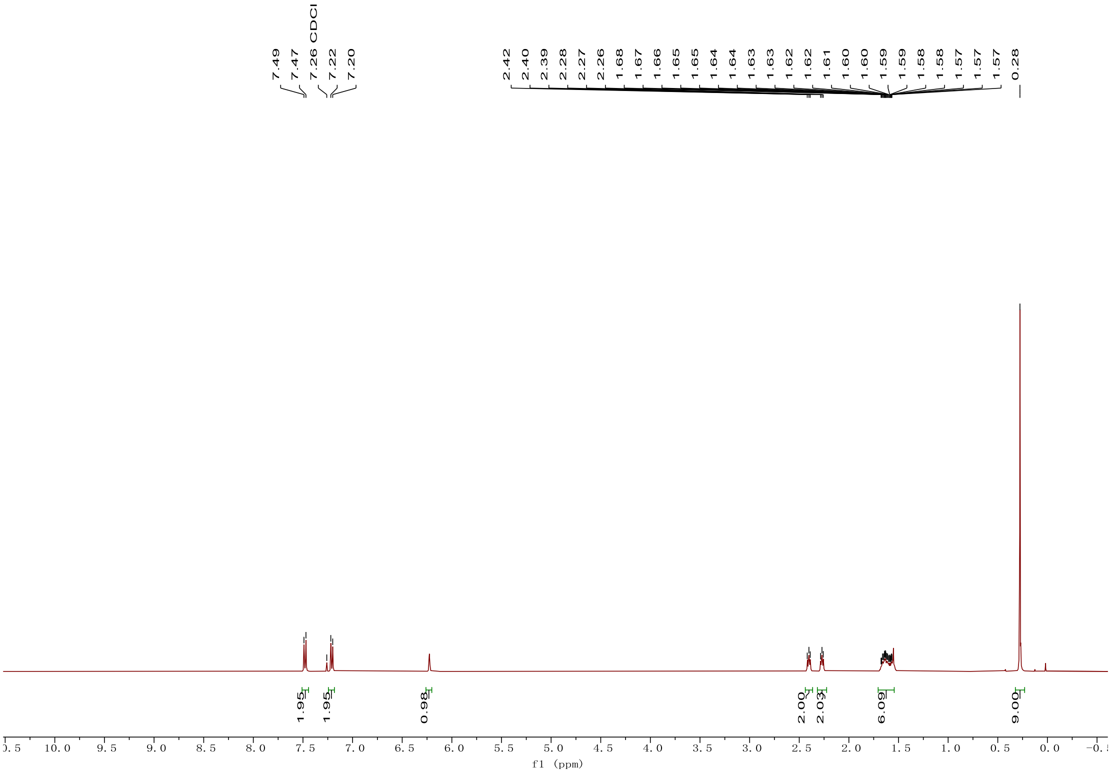

Supplementary Figure 107. ^{1}H NMR (400 MHz, Chloroform-*d*) of **66a**

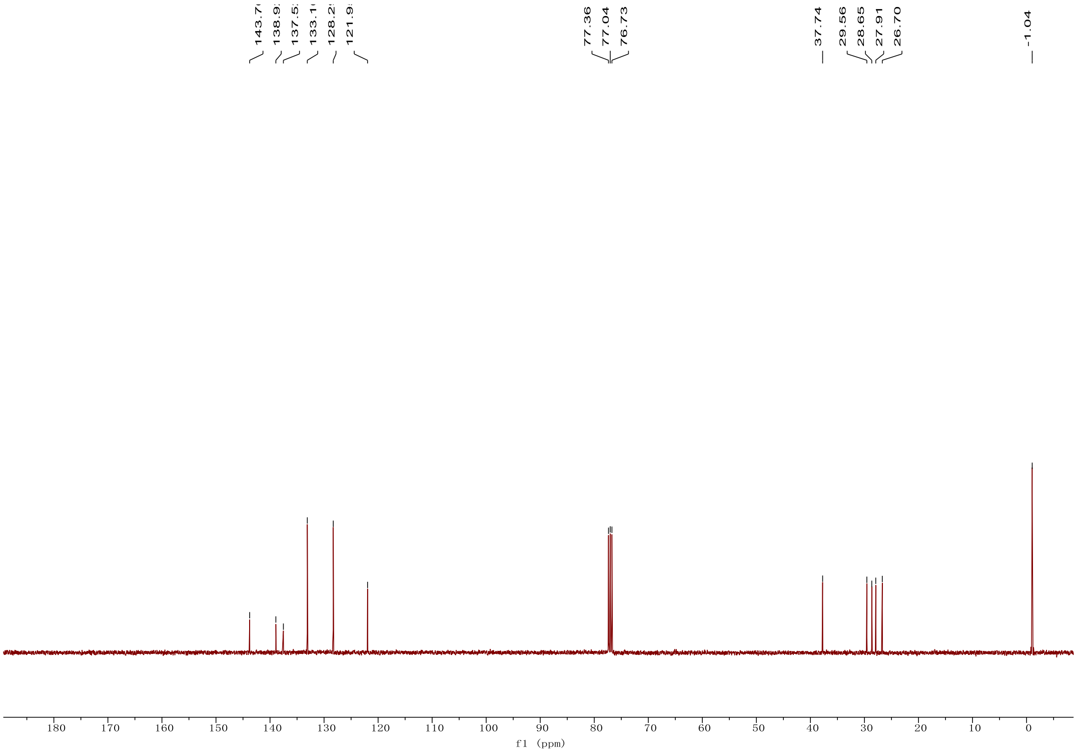

Supplementary Figure 108. ^{13}C NMR (101 MHz, Chloroform-*d*) of **66a**

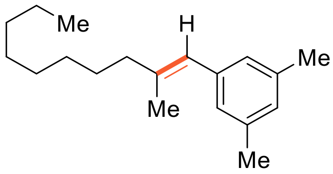

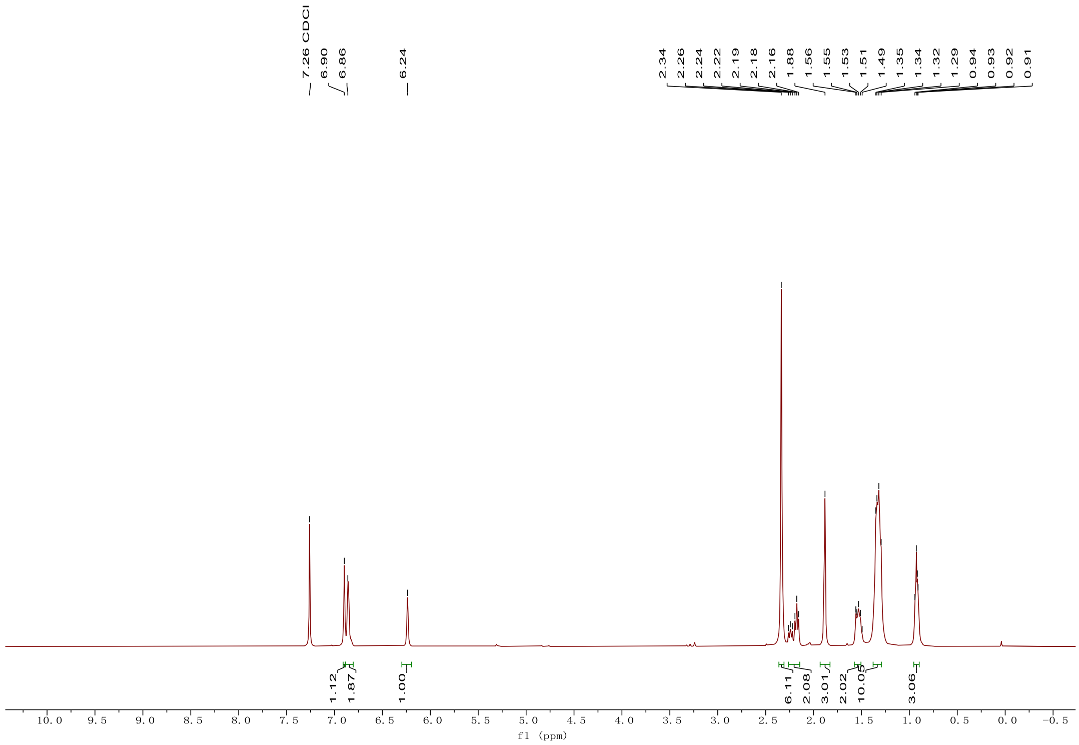

Supplementary Figure 109. ^{1}H NMR (400 MHz, Chloroform-*d*) of **68a**

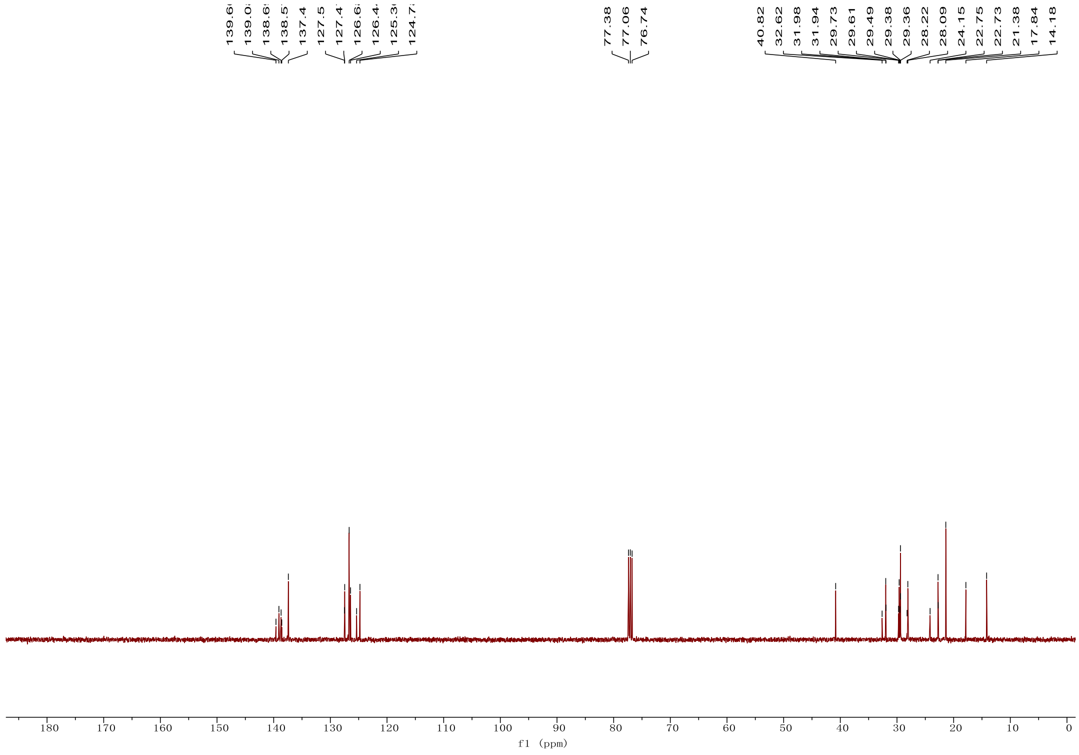

Supplementary Figure 110. ^{13}C NMR (101 MHz, Chloroform-*d*) of **68a**

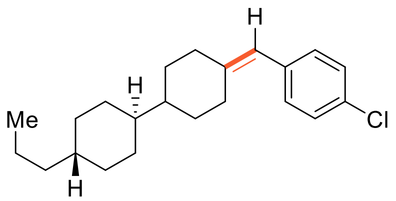

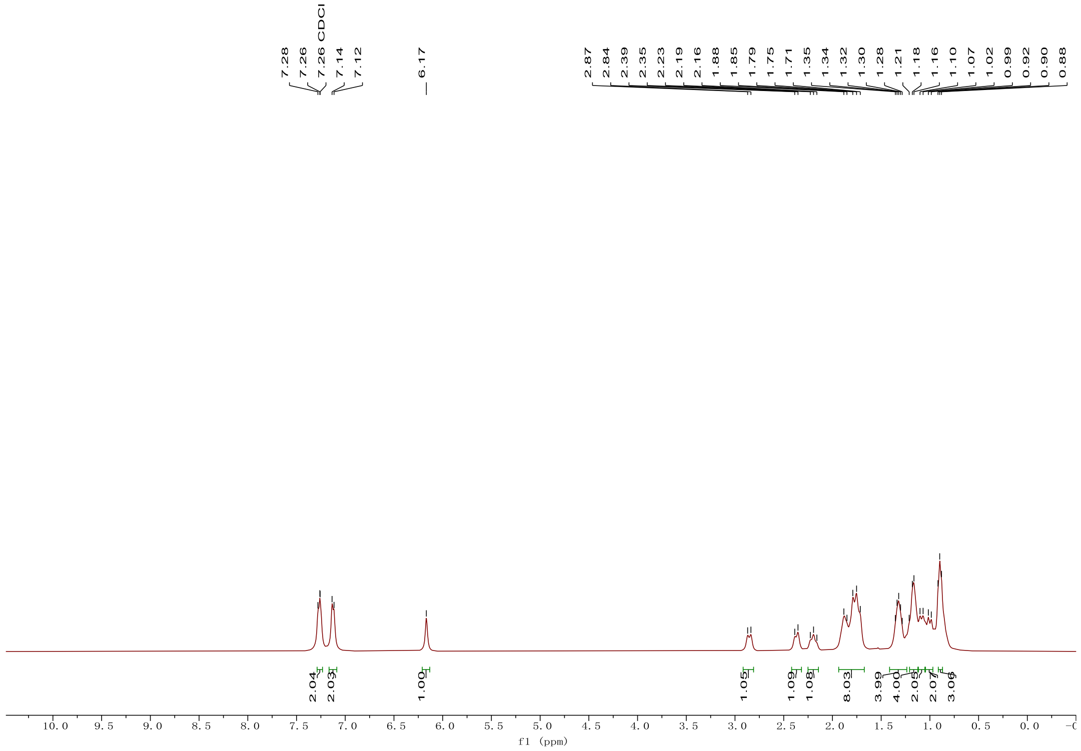

Supplementary Figure 111. **^{1}H NMR** (400 MHz, Chloroform-*d*) of **70a**

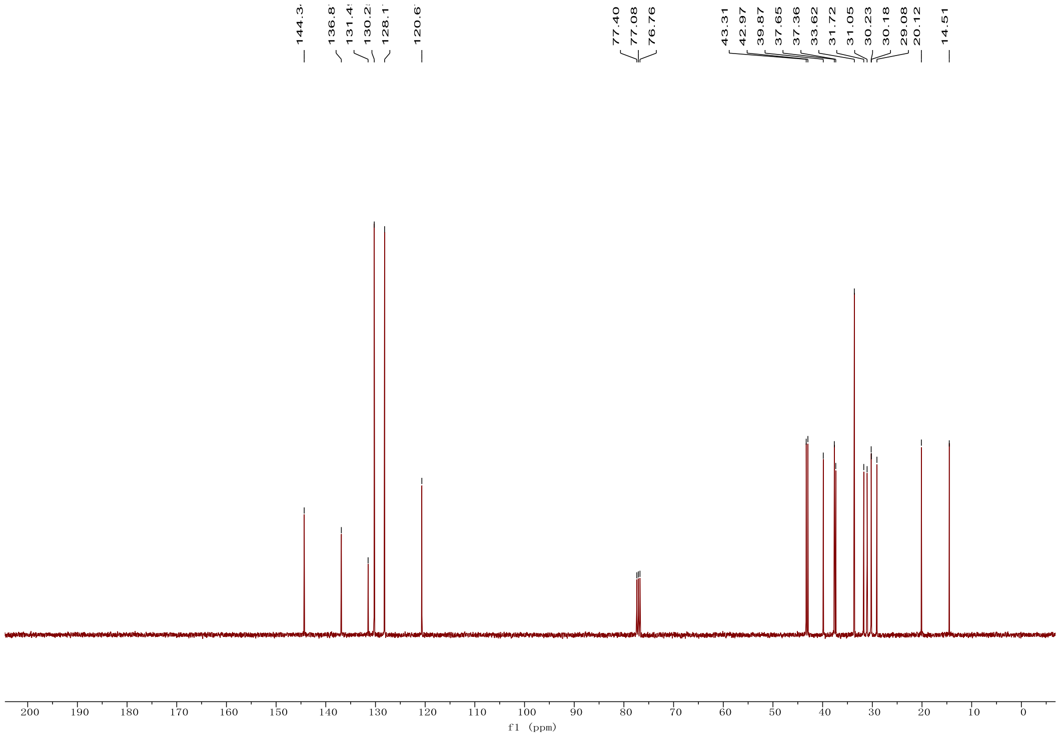

Supplementary Figure 112. **^{13}C NMR (**101 MHz, Chloroform-*d*) of **70a**

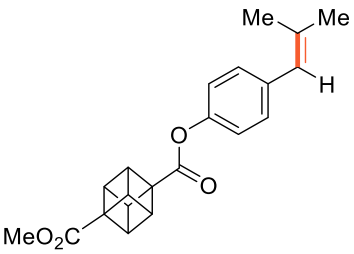

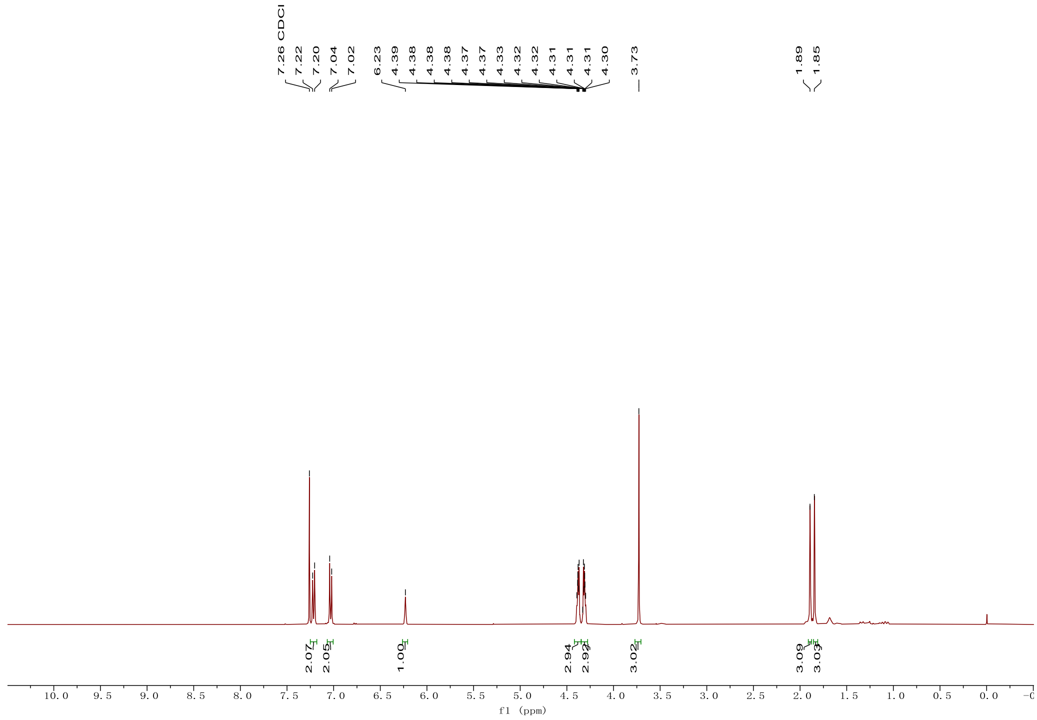

Supplementary Figure 113. ^{1}H NMR (400 MHz, Chloroform-*d*) of **77a**

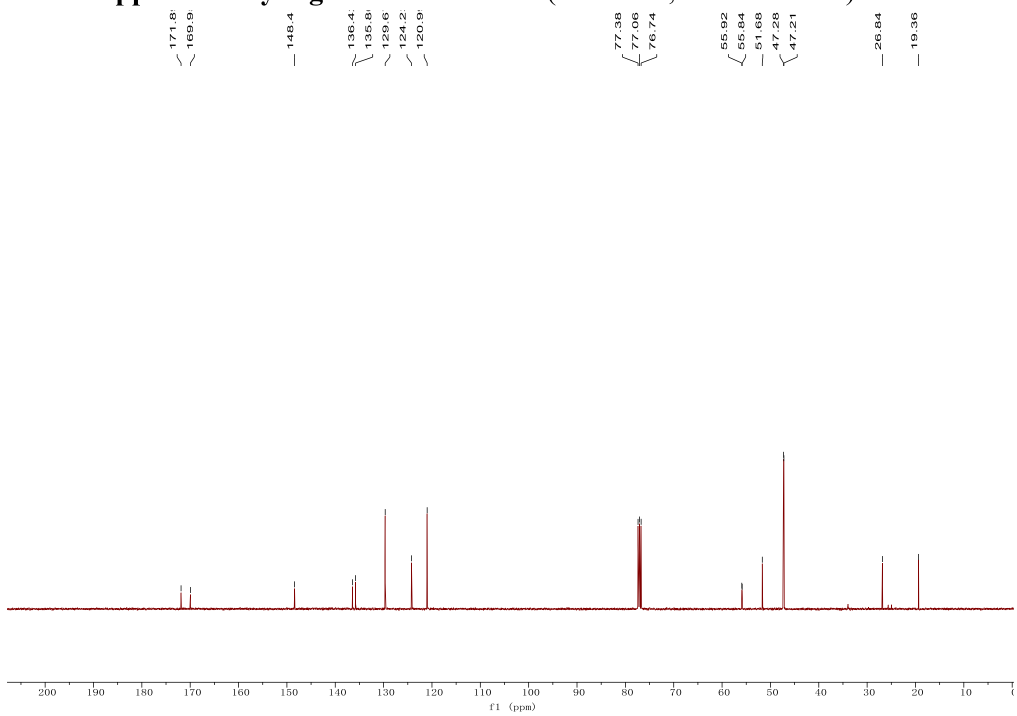

Supplementary Figure 114. ^{13}C NMR (101 MHz, Chloroform-*d*) of **77a**

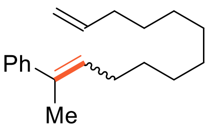

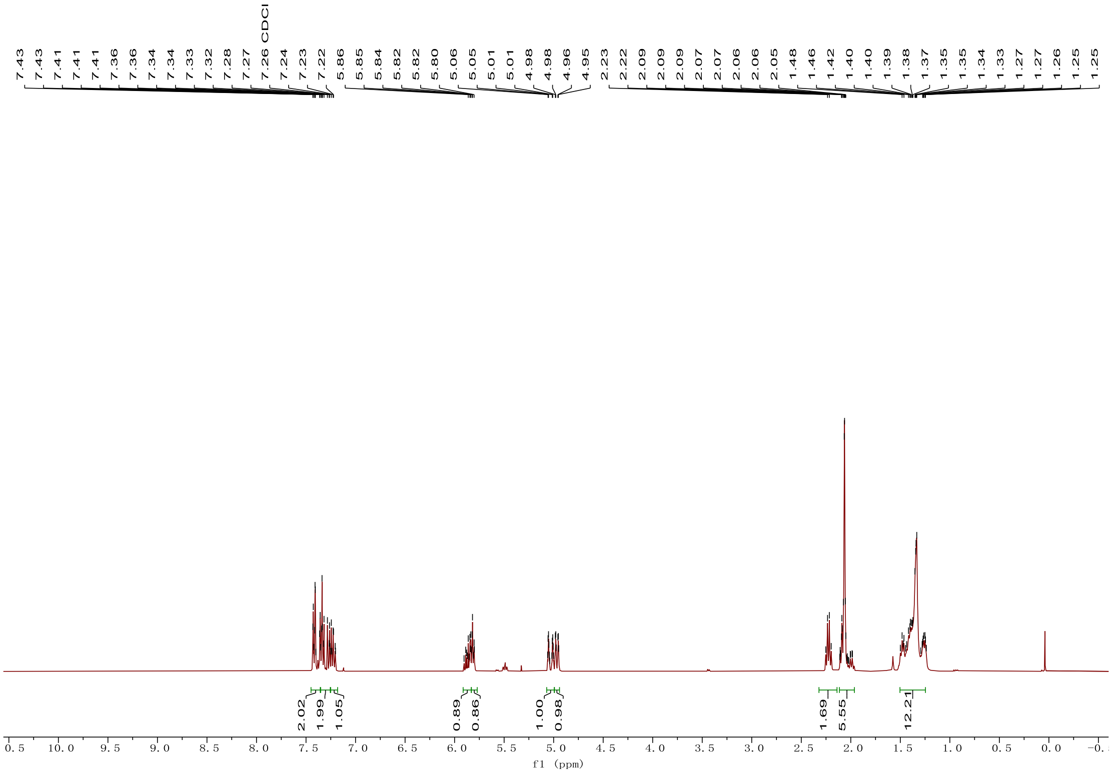

Supplementary Figure 115. **^{1}H NMR** (400 MHz, Chloroform-*d*) of **79a**

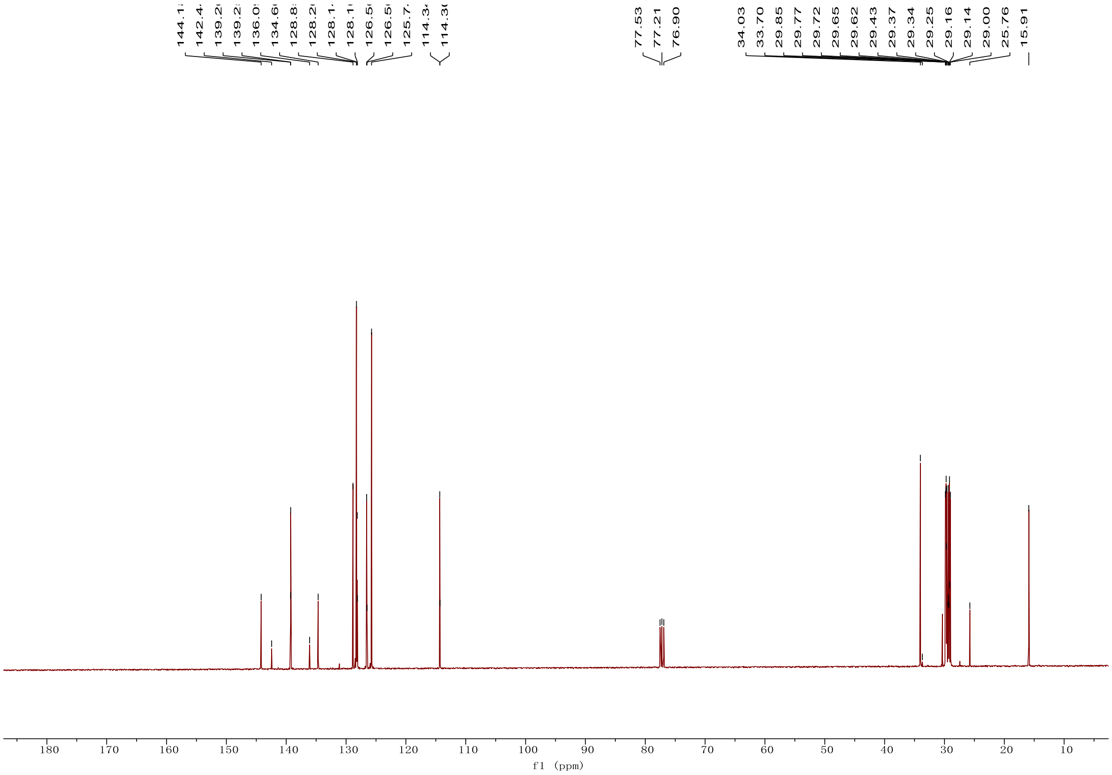

Supplementary Figure 116. **^{13}C NMR (**101 MHz, Chloroform-*d*) of **79a**

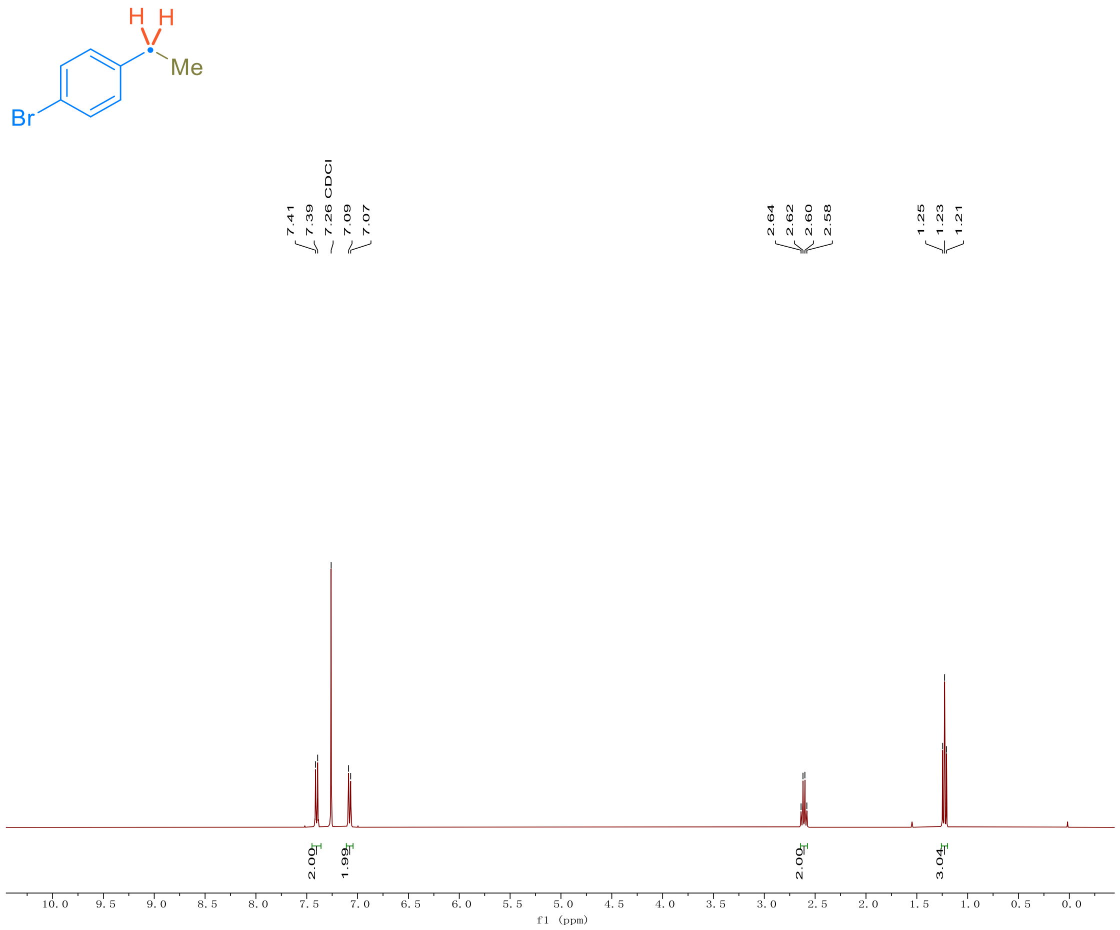

Supplementary Figure 117. ^{1}**H NMR** (400 MHz, Chloroform-*d*) of **1**

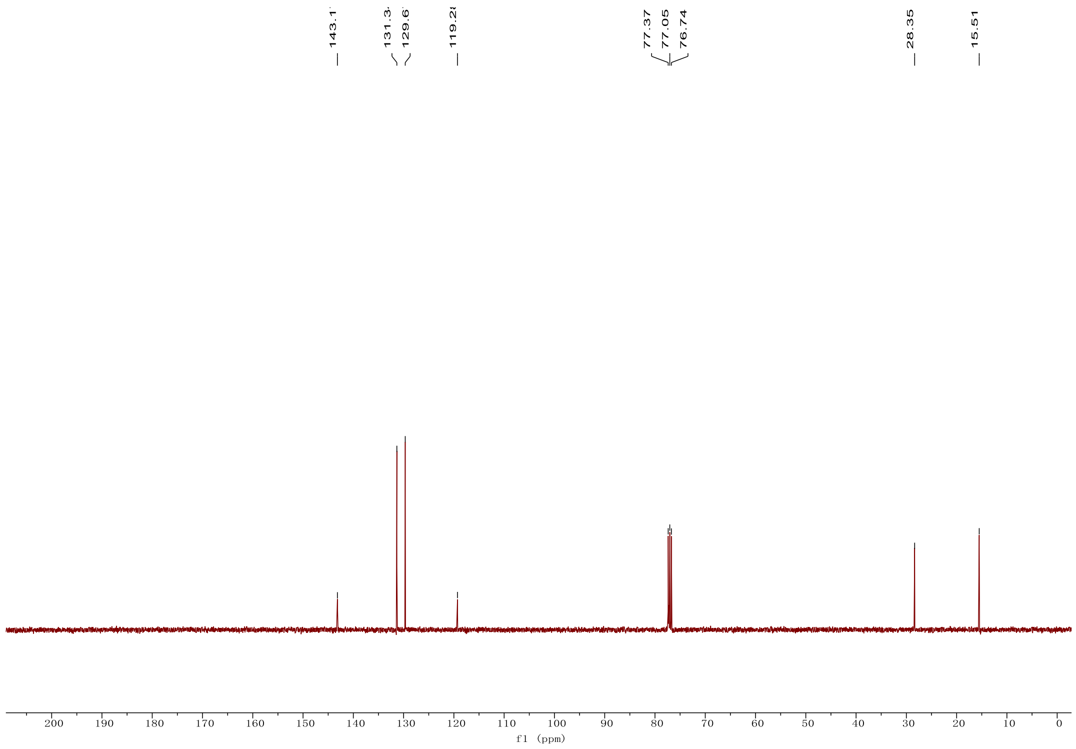

Supplementary Figure 118. ^{13}**C NMR (**101 MHz, Chloroform-*d*) of **1**

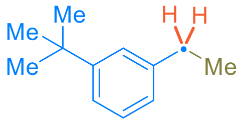

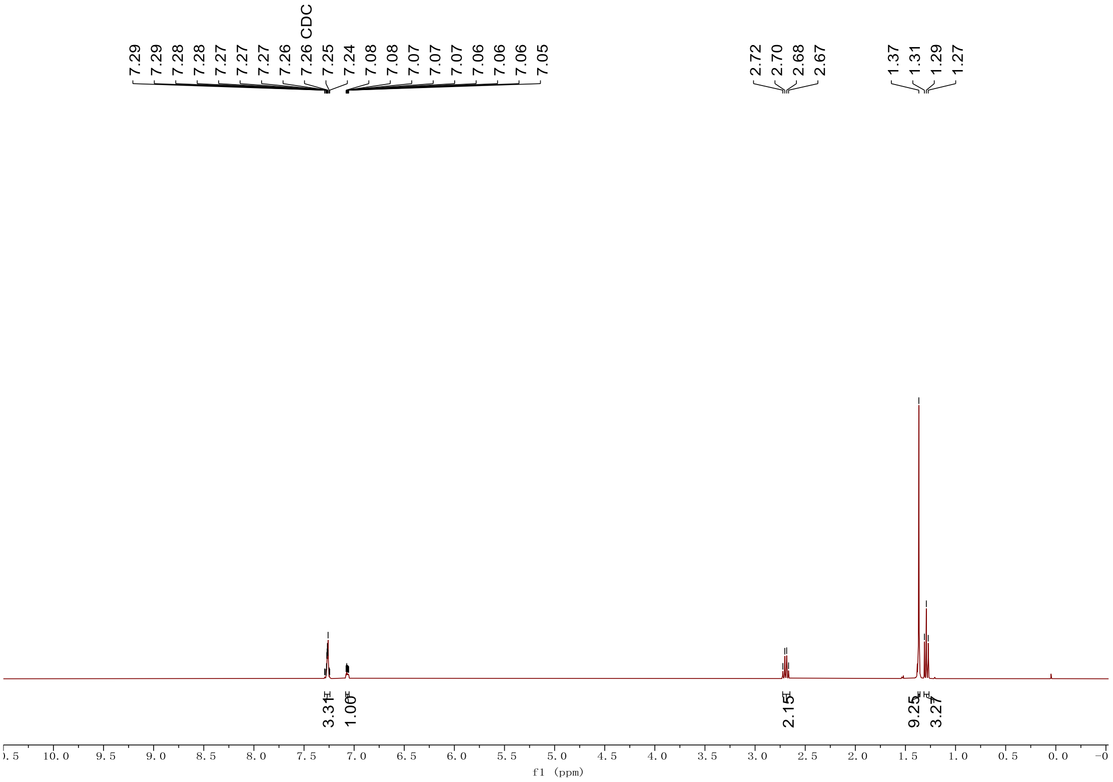

Supplementary Figure 119. ^{1}**H NMR** (400 MHz, Chloroform-*d*) of **3**

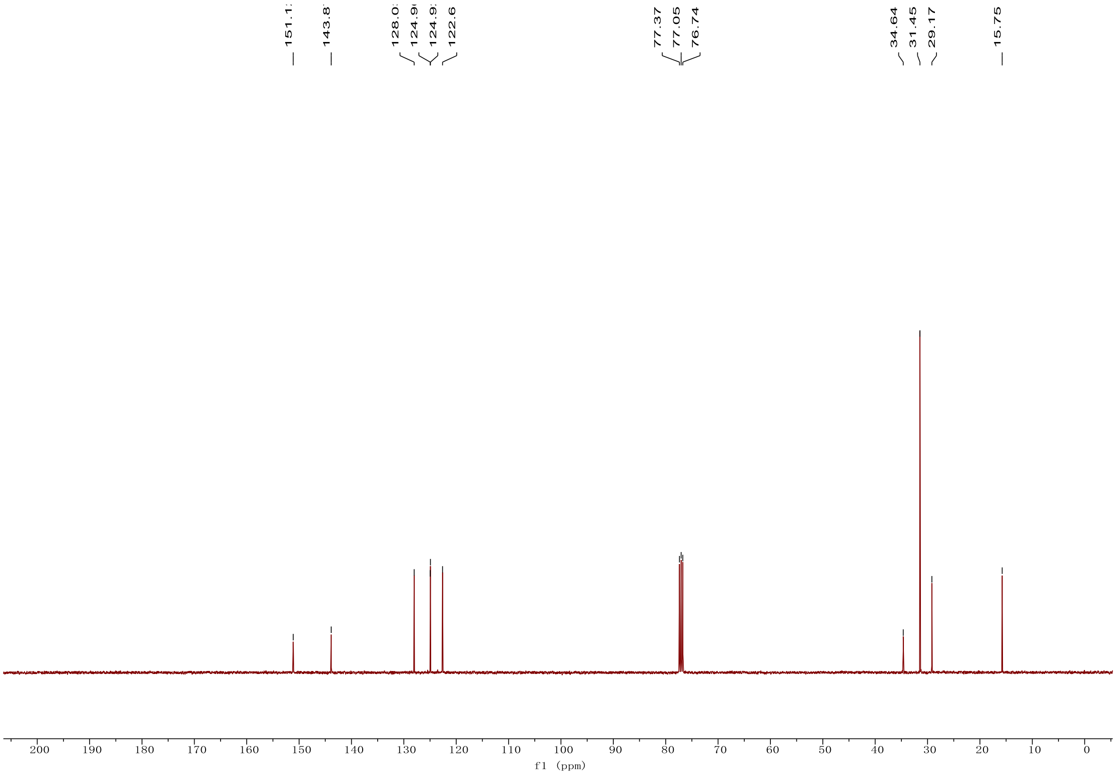

Supplementary Figure 120. ^{13}**C NMR (**101 MHz, Chloroform-*d*) of **3**

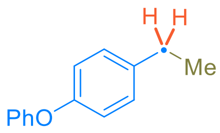

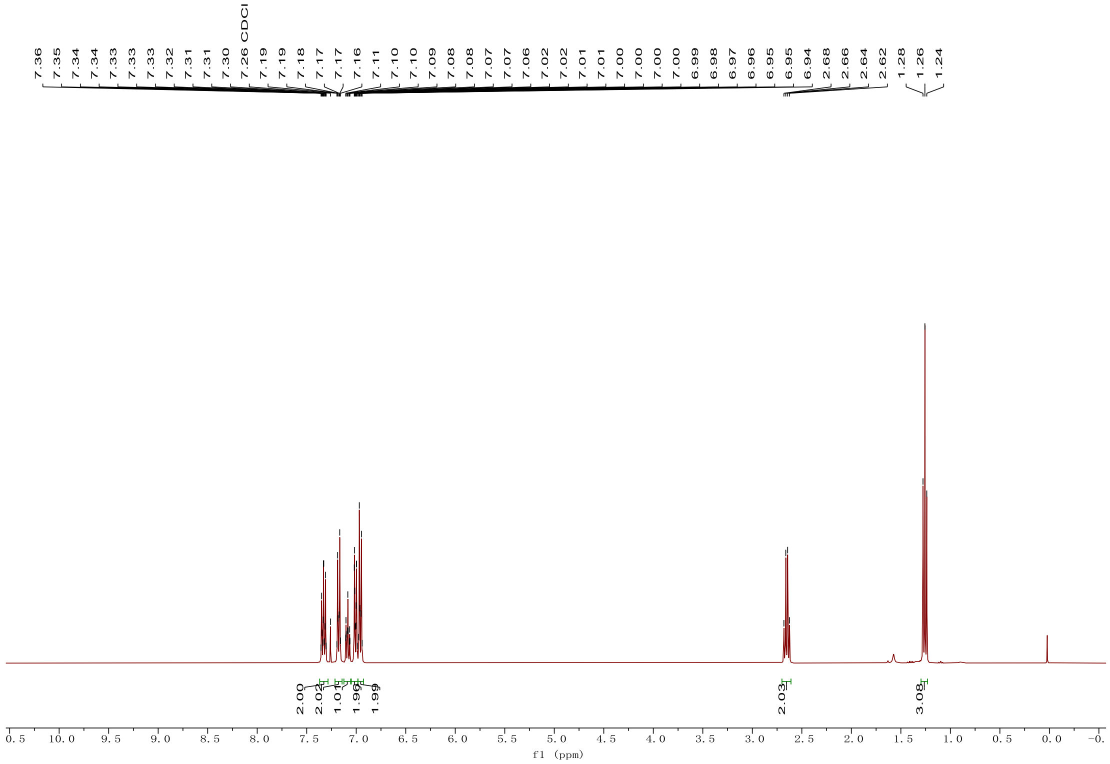

Supplementary Figure 121. ^{1}**H NMR** (400 MHz, Chloroform-*d*) of **4**

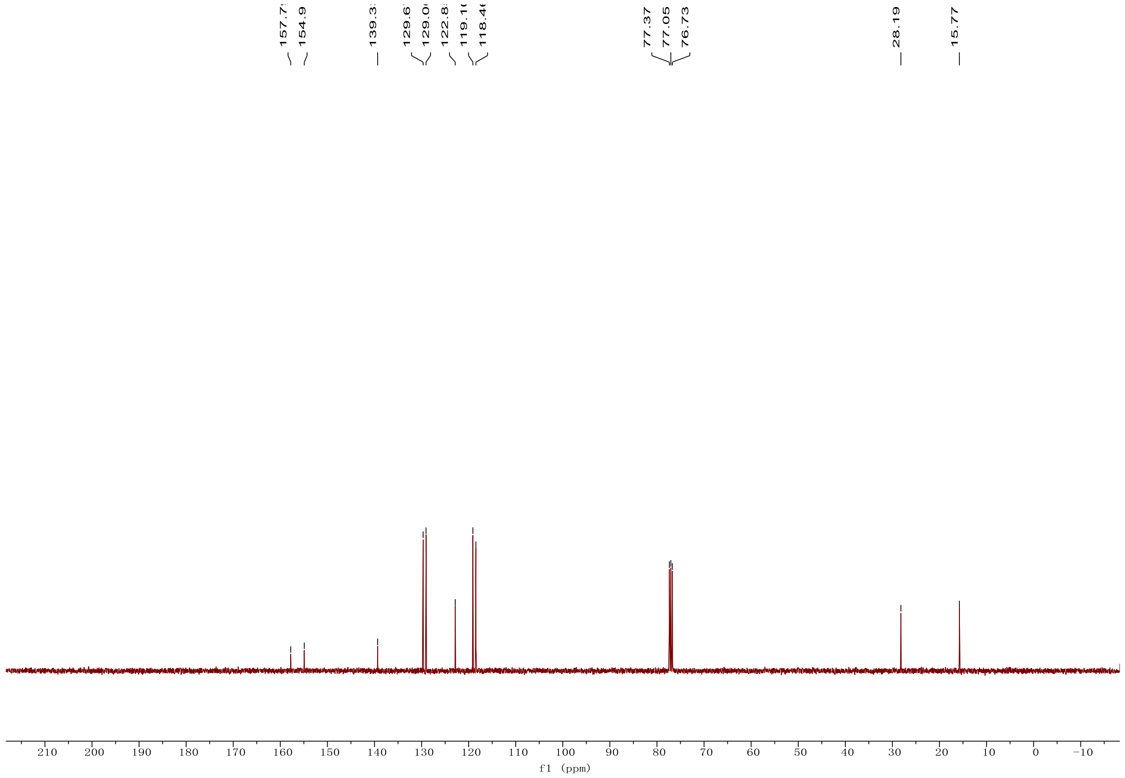

Supplementary Figure 122. ^{13}**C NMR (**101 MHz, Chloroform-*d*) of **4**

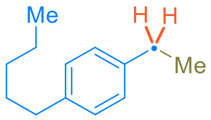

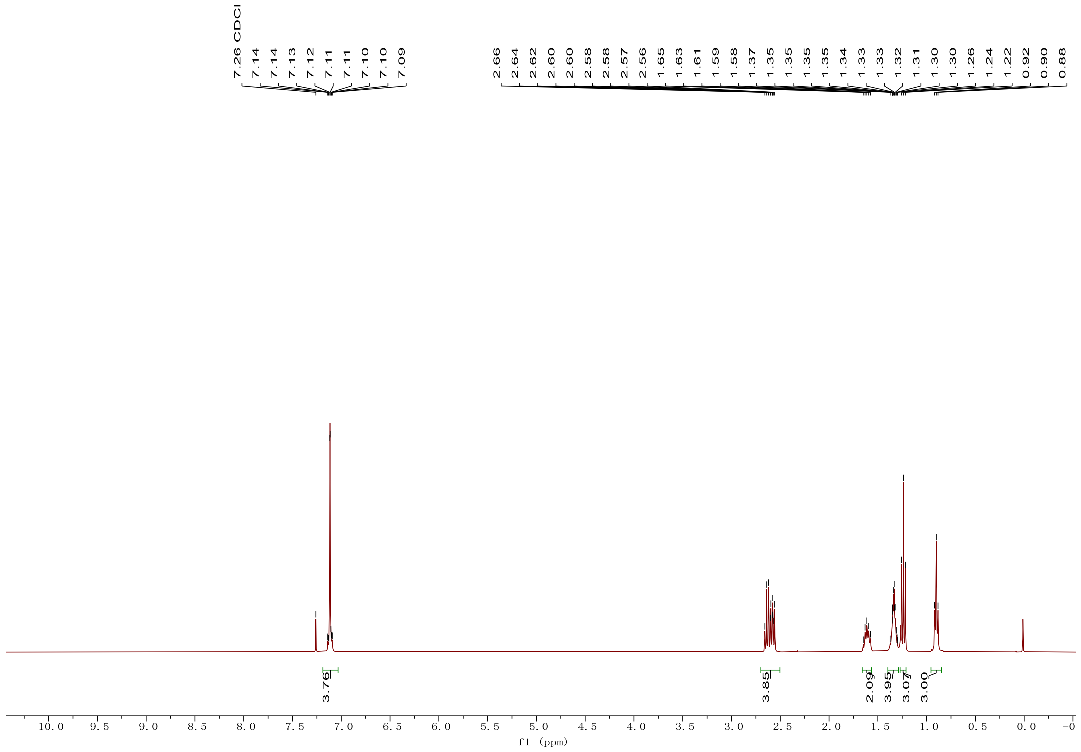

Supplementary Figure 123. ^{1}**H NMR** (400 MHz, Chloroform-*d*) of **5**

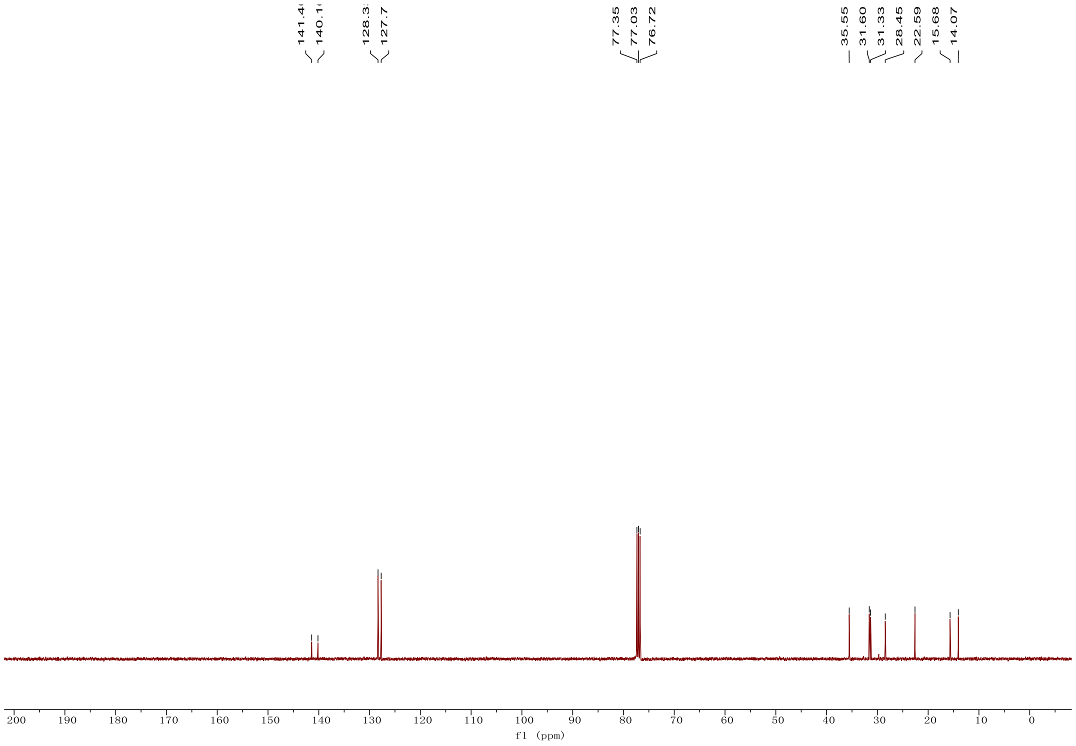

Supplementary Figure 124. ^{13}**C NMR** (101 MHz, Chloroform-*d*) of **5**

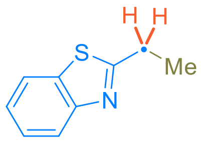

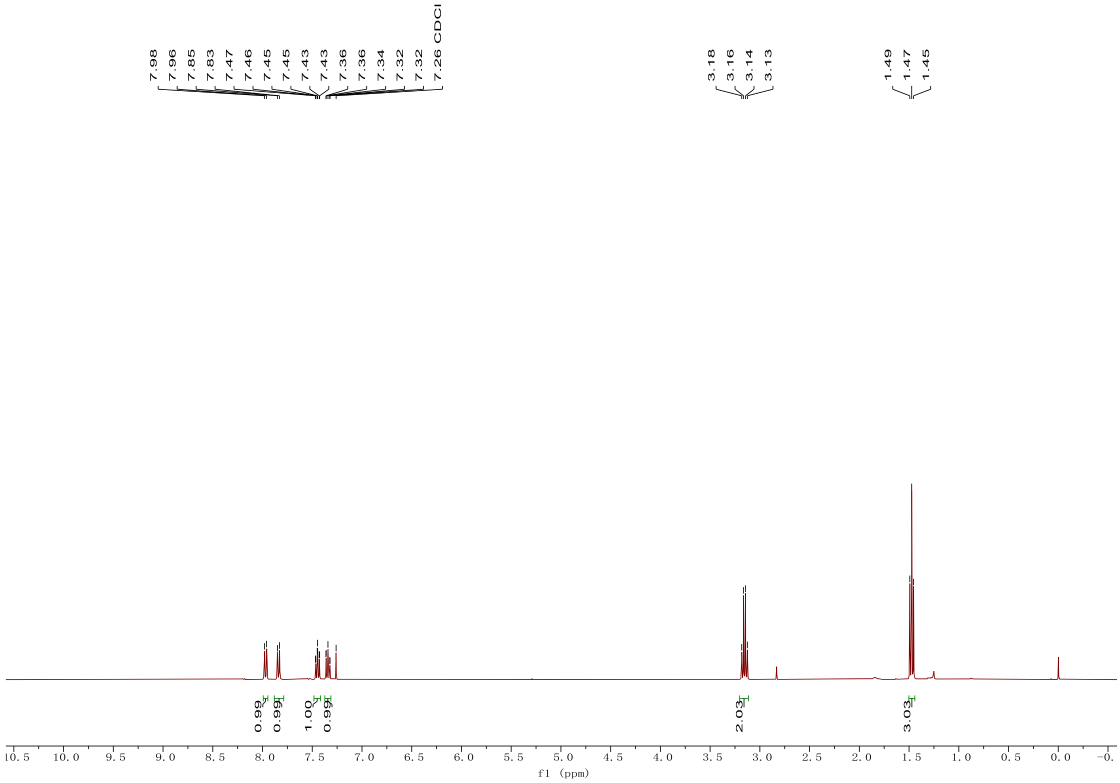

Supplementary Figure 125. ^{1}H NMR (400 MHz, Chloroform-*d*) of **6**

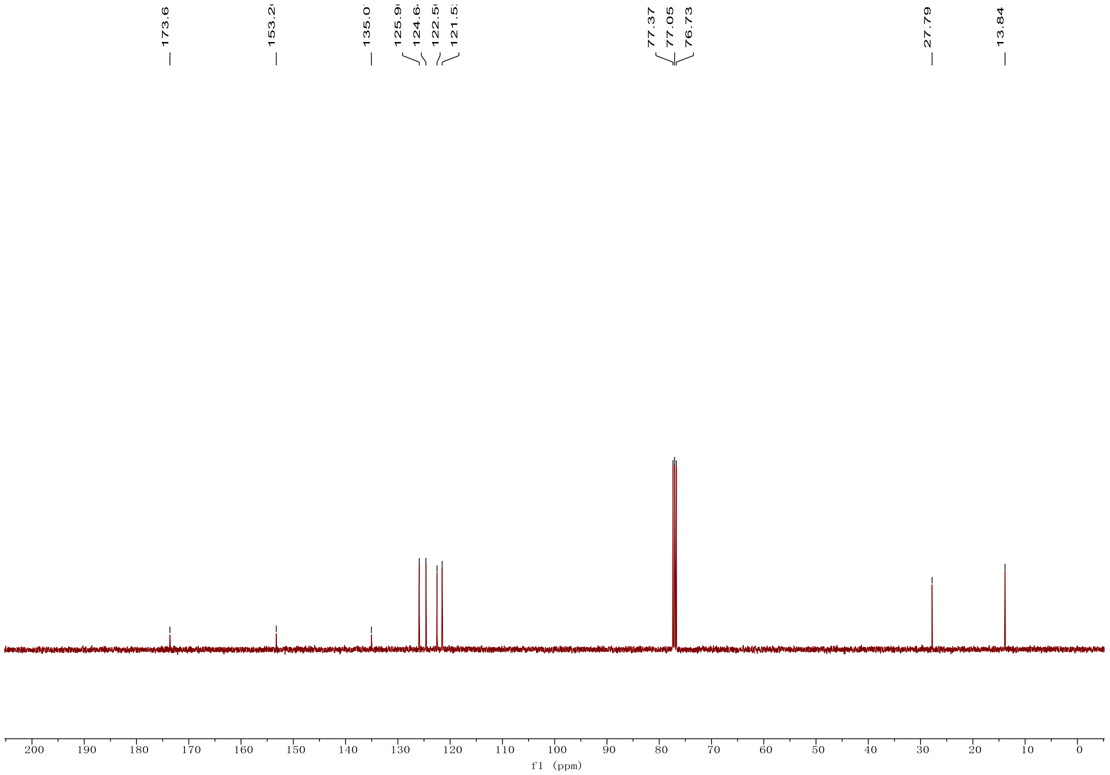

Supplementary Figure 126. ^{13}C NMR (101 MHz, Chloroform-*d*) of **6**

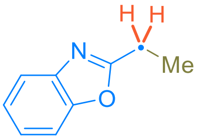

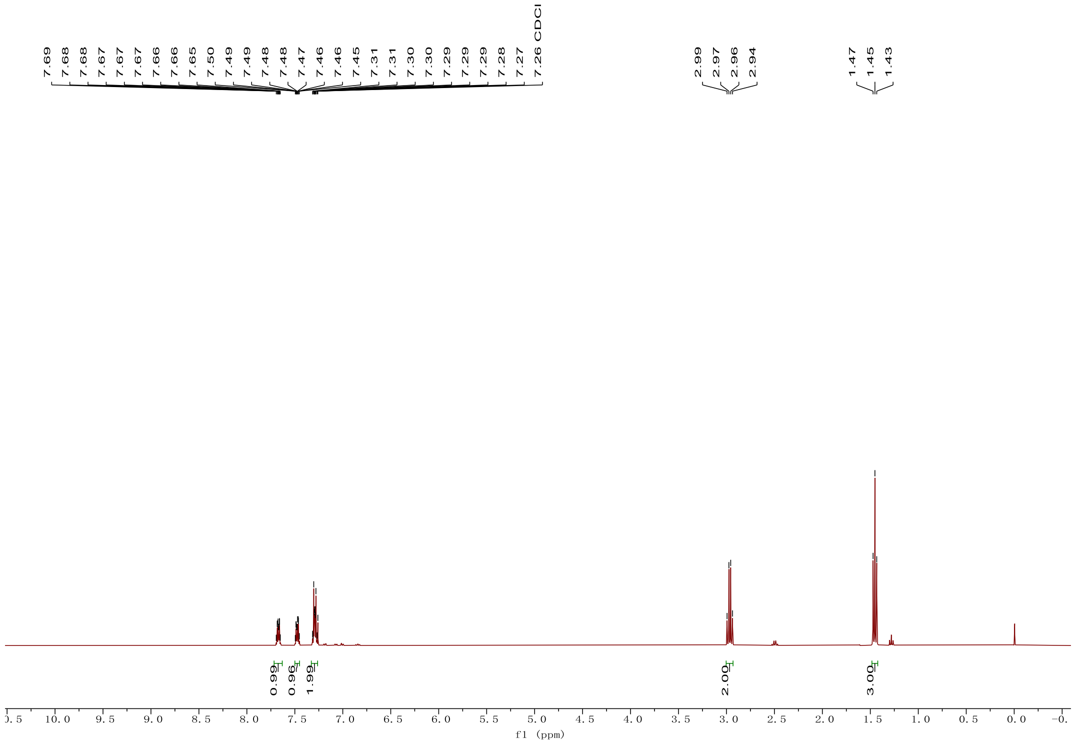

Supplementary Figure 127. ^{1}H NMR (400 MHz, Chloroform-*d*) of **7**

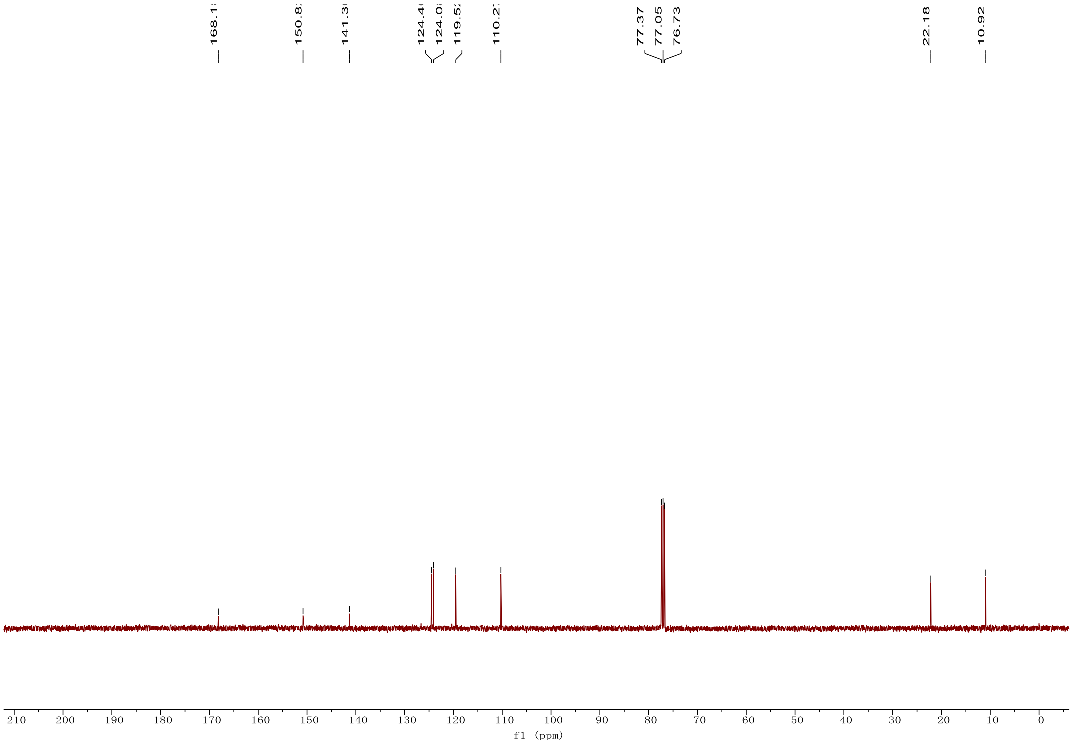

Supplementary Figure 128. ^{13}C NMR (101 MHz, Chloroform-*d*) of **7**

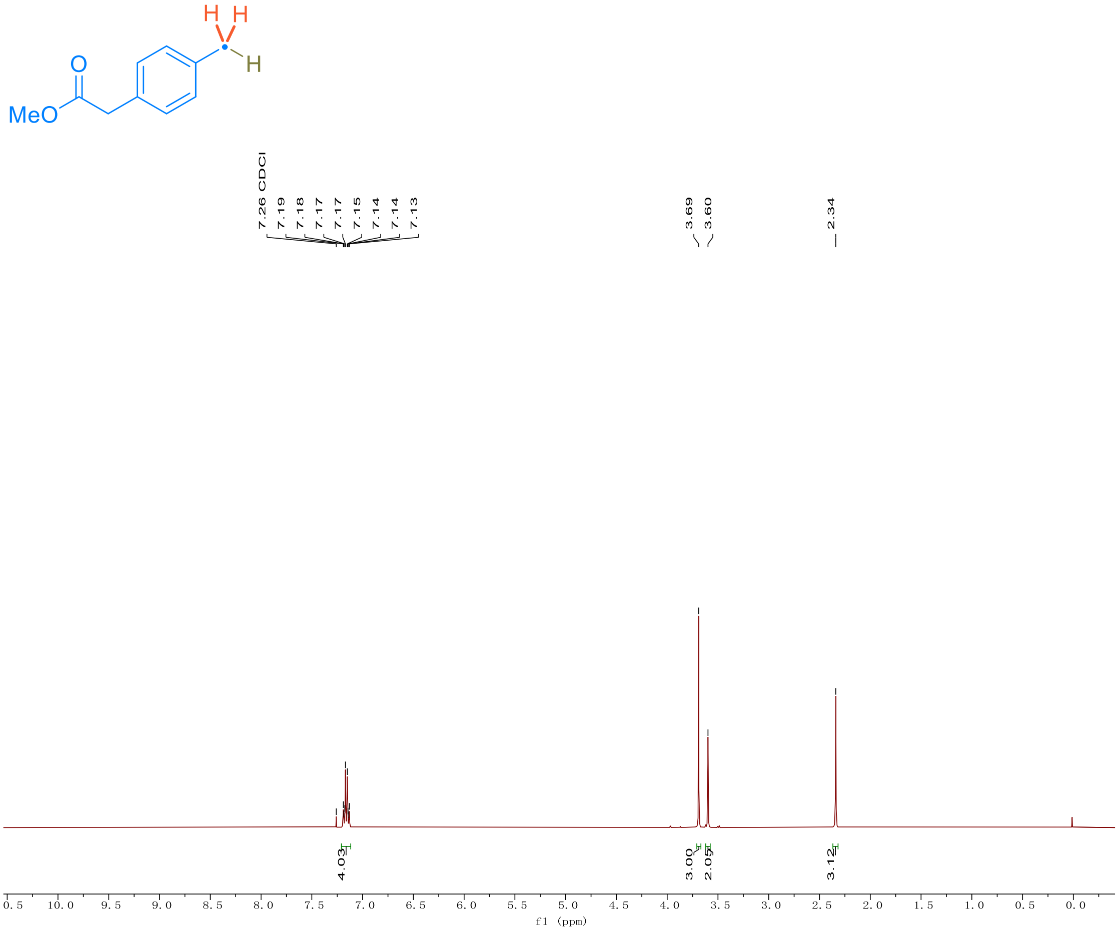

Supplementary Figure 129. **^{1}H NMR** (400 MHz, Chloroform-*d*) of **8**

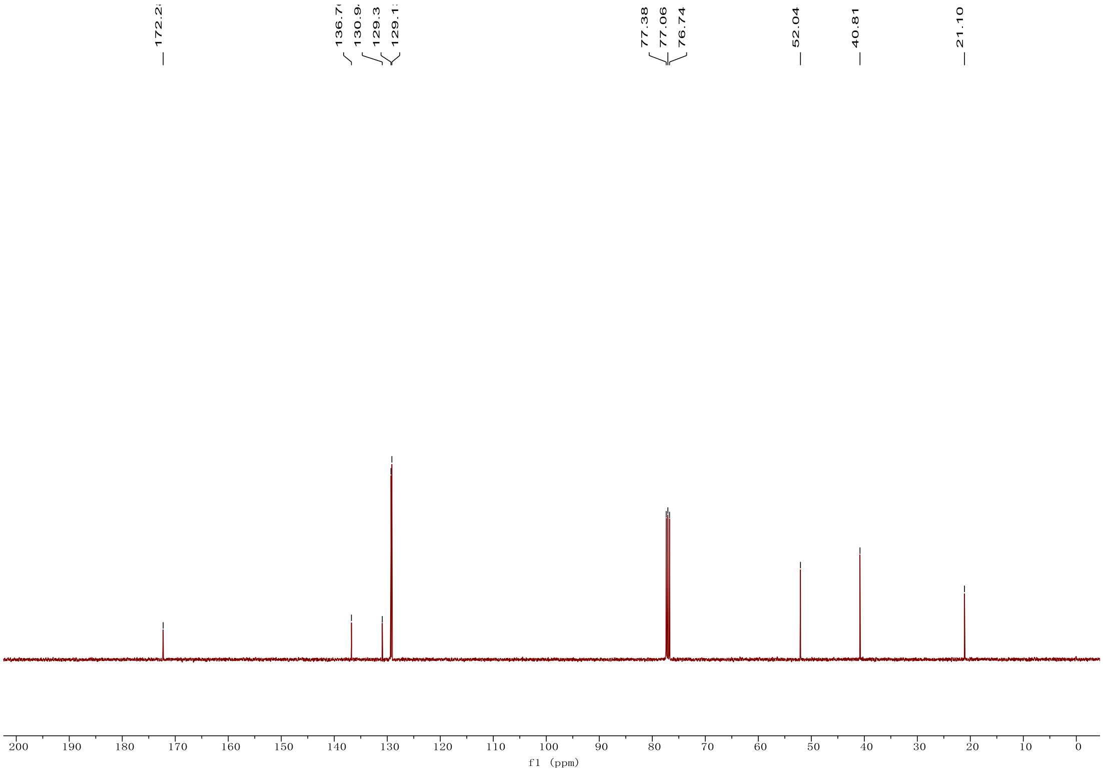

Supplementary Figure 130. **^{13}C NMR (**101 MHz, Chloroform-*d*) of **8**

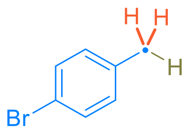

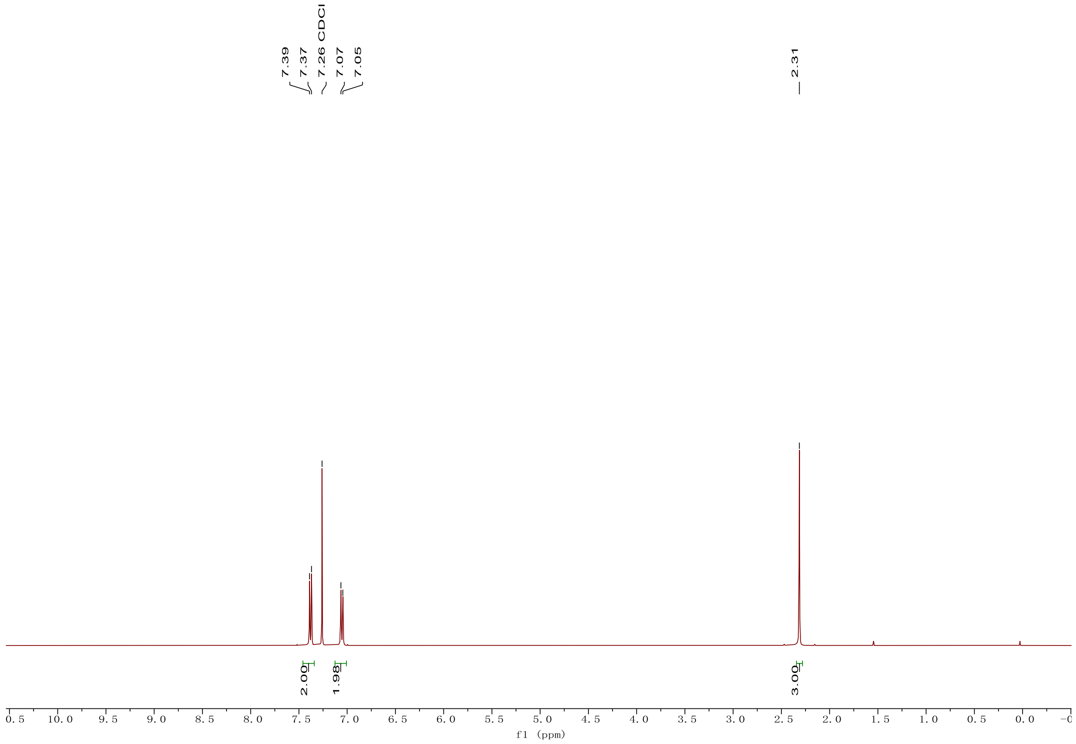

Supplementary Figure 131. ^{1}**H NMR** (400 MHz, Chloroform-*d*) of **9**

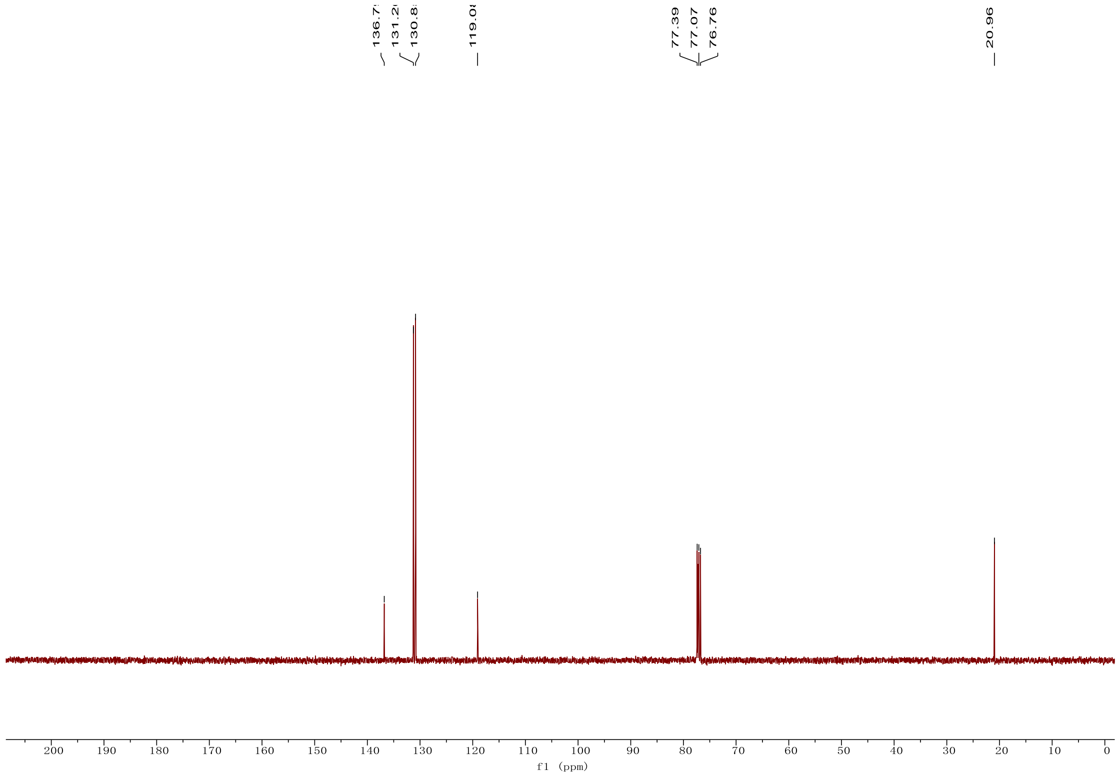

Supplementary Figure 132. ^{13}**C NMR** (101 MHz, Chloroform-*d*) of **9**

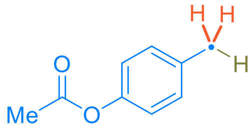

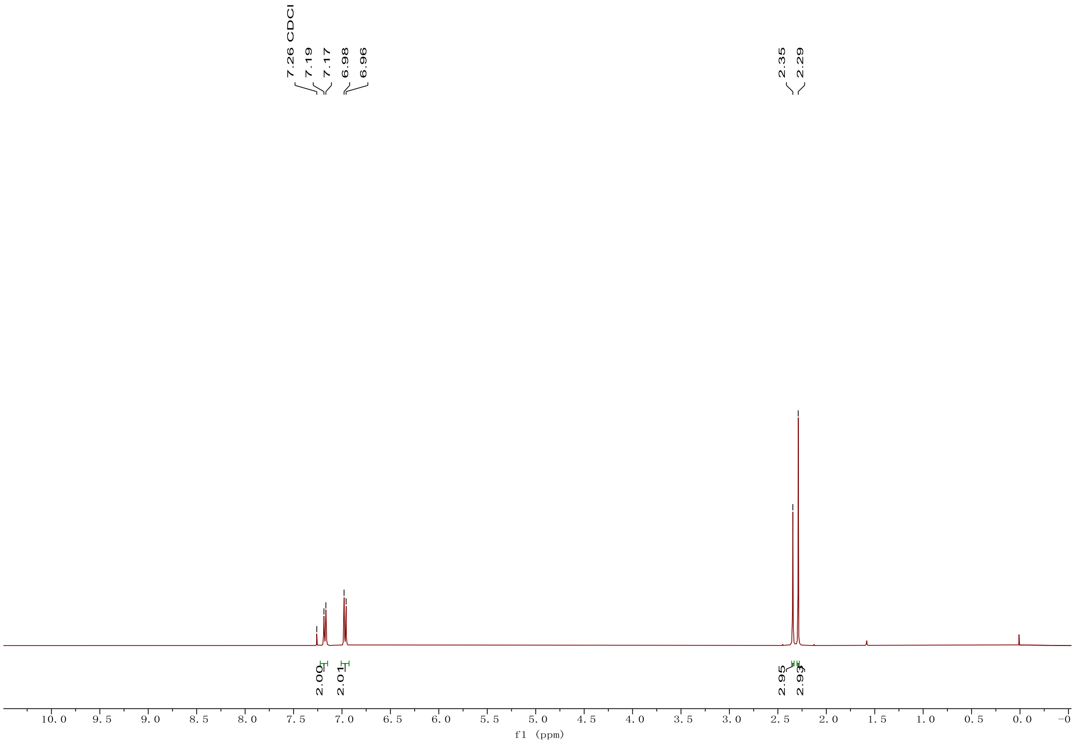

Supplementary Figure 133. **^{1}H NMR** (400 MHz, Chloroform-*d*) of **10**

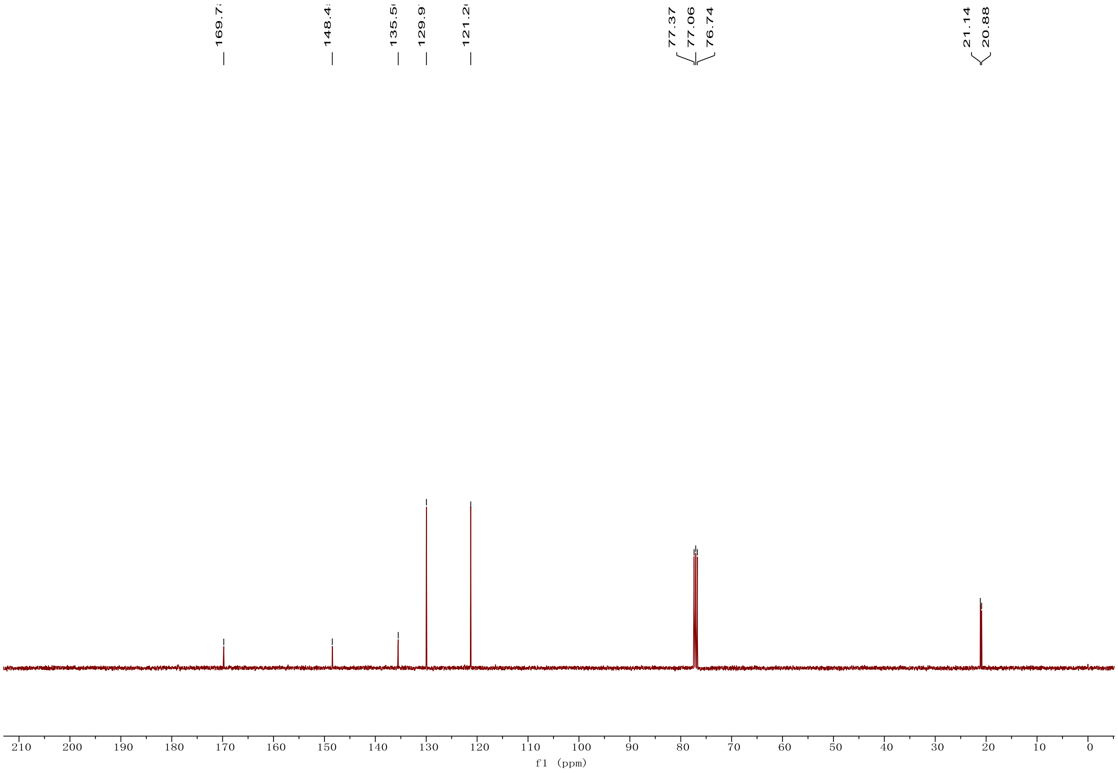

Supplementary Figure 134. **^{13}C NMR (**101 MHz, Chloroform-*d*) of **10**

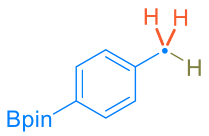

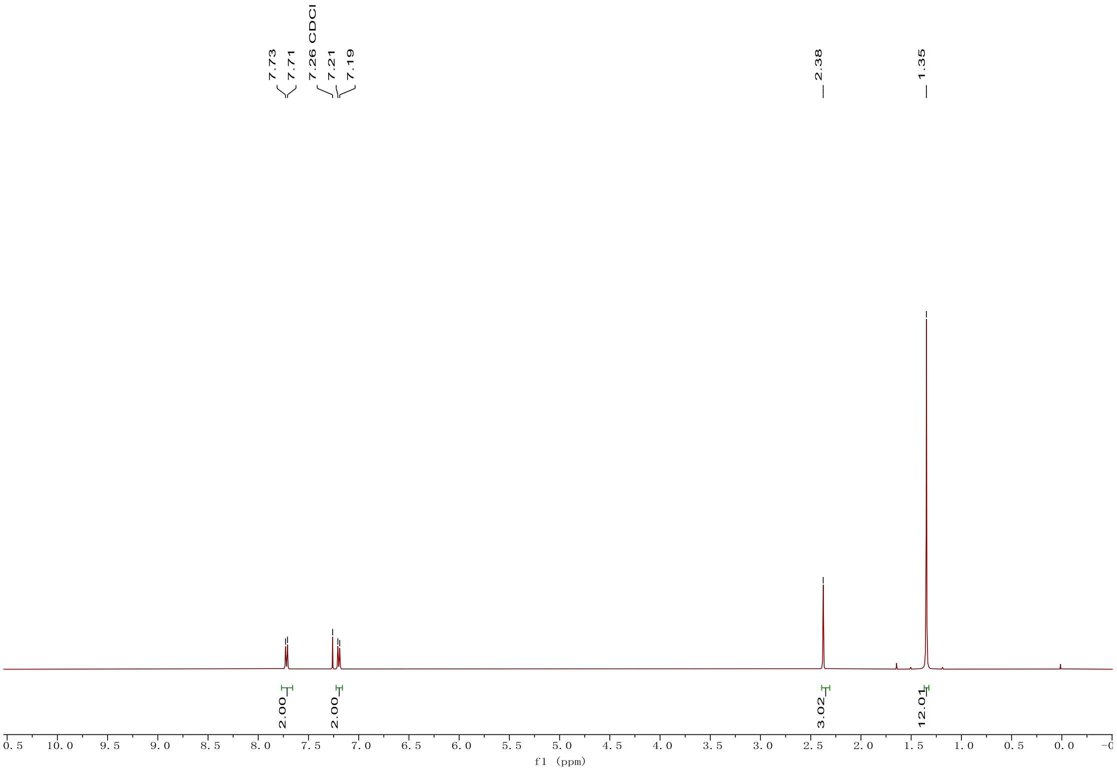

Supplementary Figure 135. ^{1}**H NMR** (400 MHz, Chloroform-*d*) of **11**

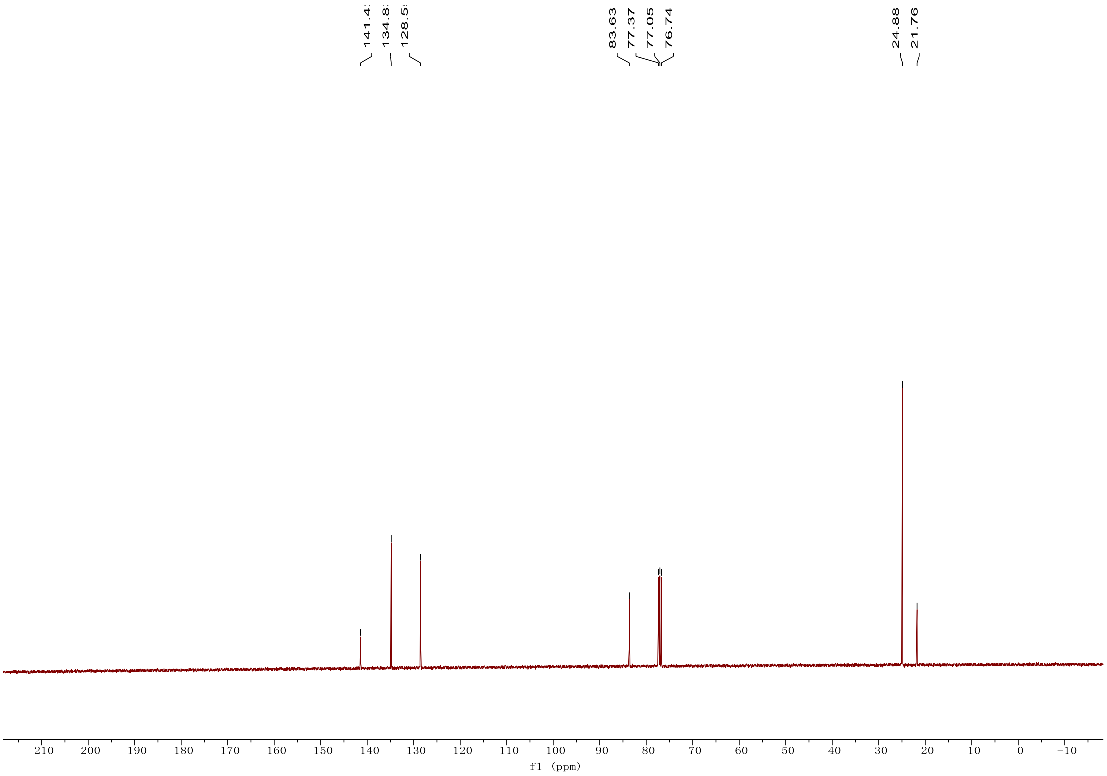

Supplementary Figure 136. ^{13}**C NMR** (101 MHz, Chloroform-*d*) of **11**

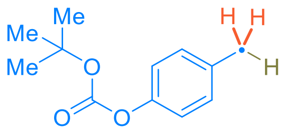

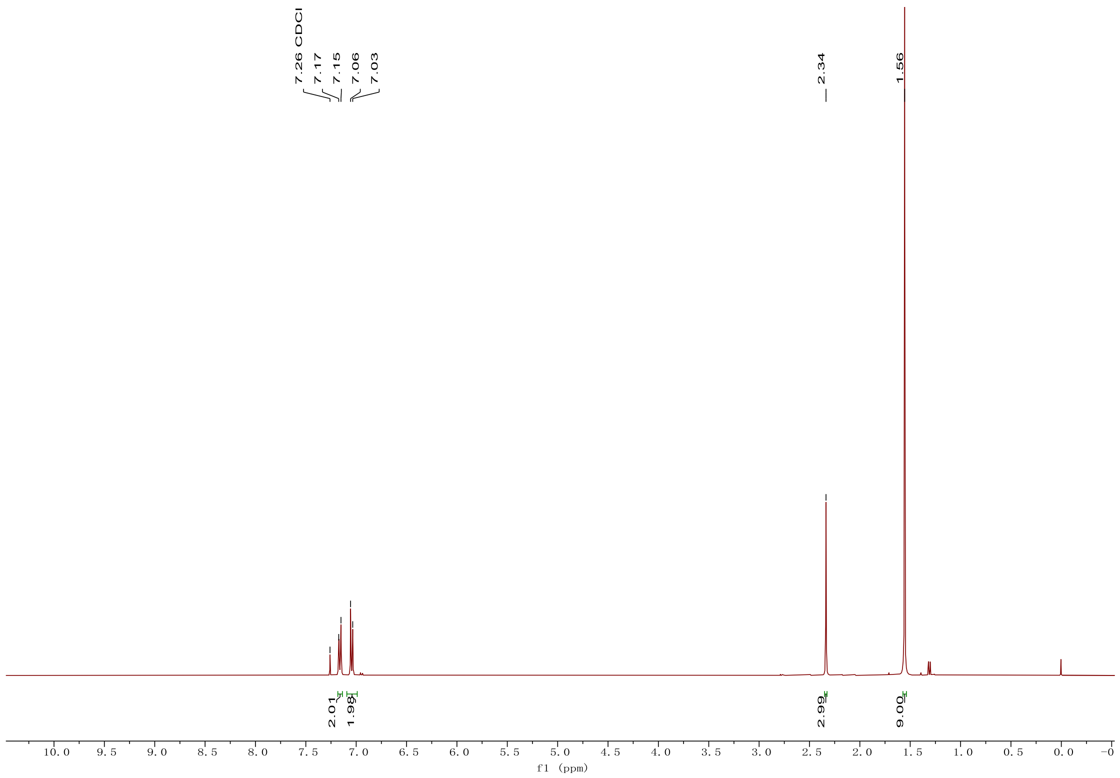

Supplementary Figure 137. ^{1}**H NMR** (400 MHz, Chloroform-*d*) of **13**

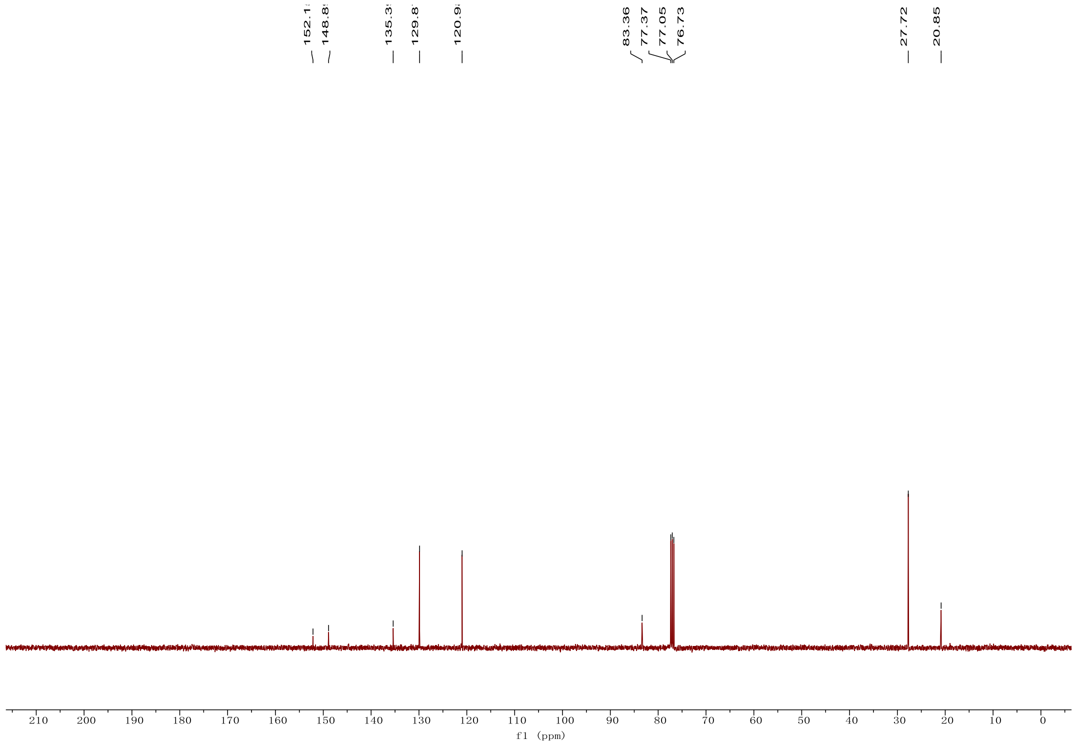

Supplementary Figure 138. ^{13}**C NMR (**101 MHz, Chloroform-*d*) of **13**

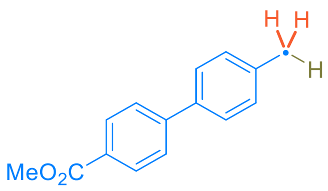

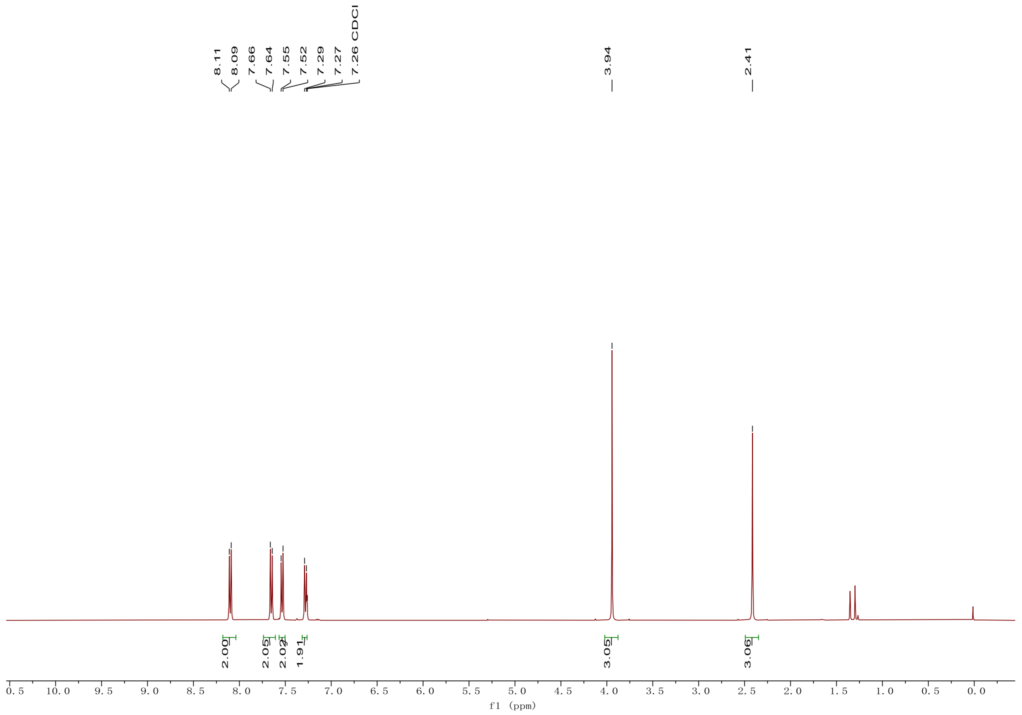

Supplementary Figure 139. ^{1}**H NMR** (400 MHz, Chloroform-*d*) of **14**

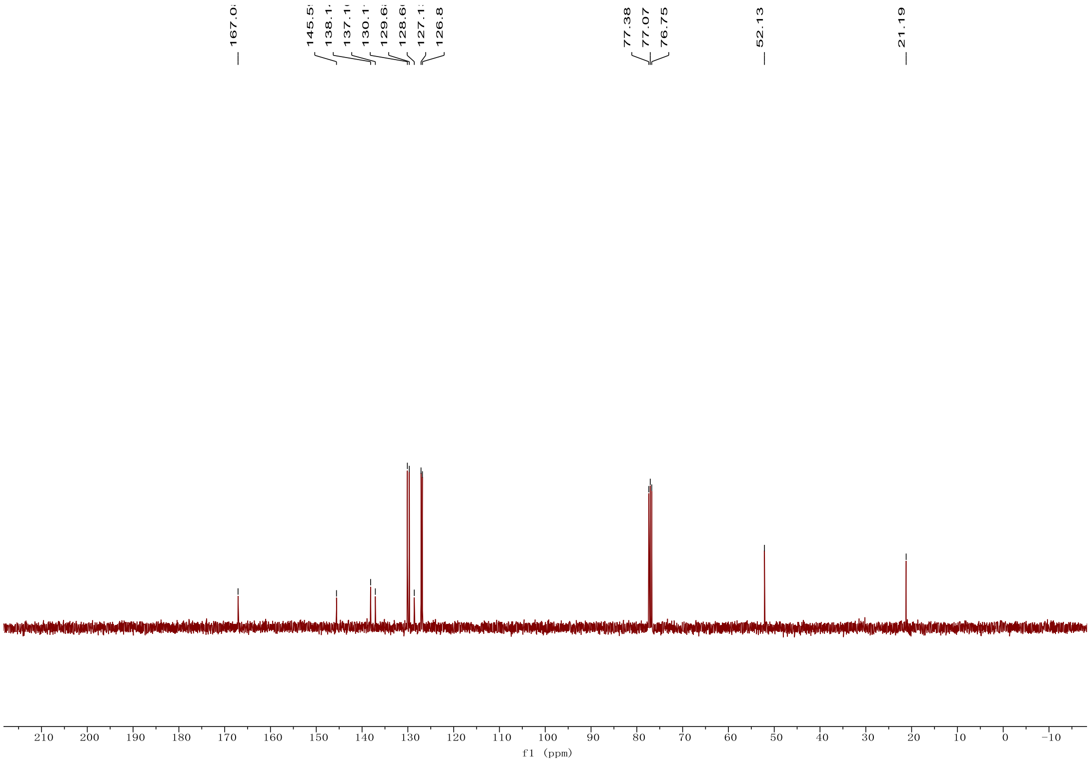

Supplementary Figure 140. ^{13}**C NMR (**101 MHz, Chloroform-*d*) of **14**

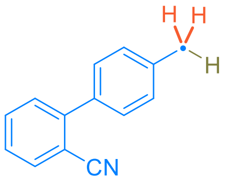

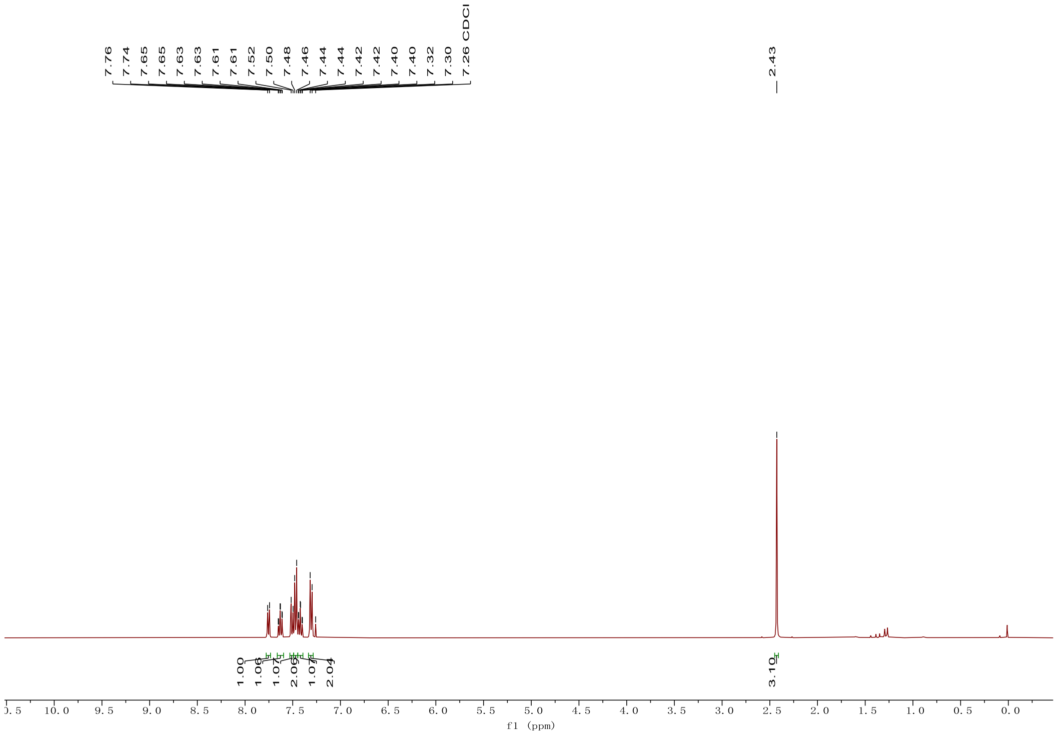

Supplementary Figure 141. ¹H NMR (400 MHz, Chloroform-*d*) of **15**

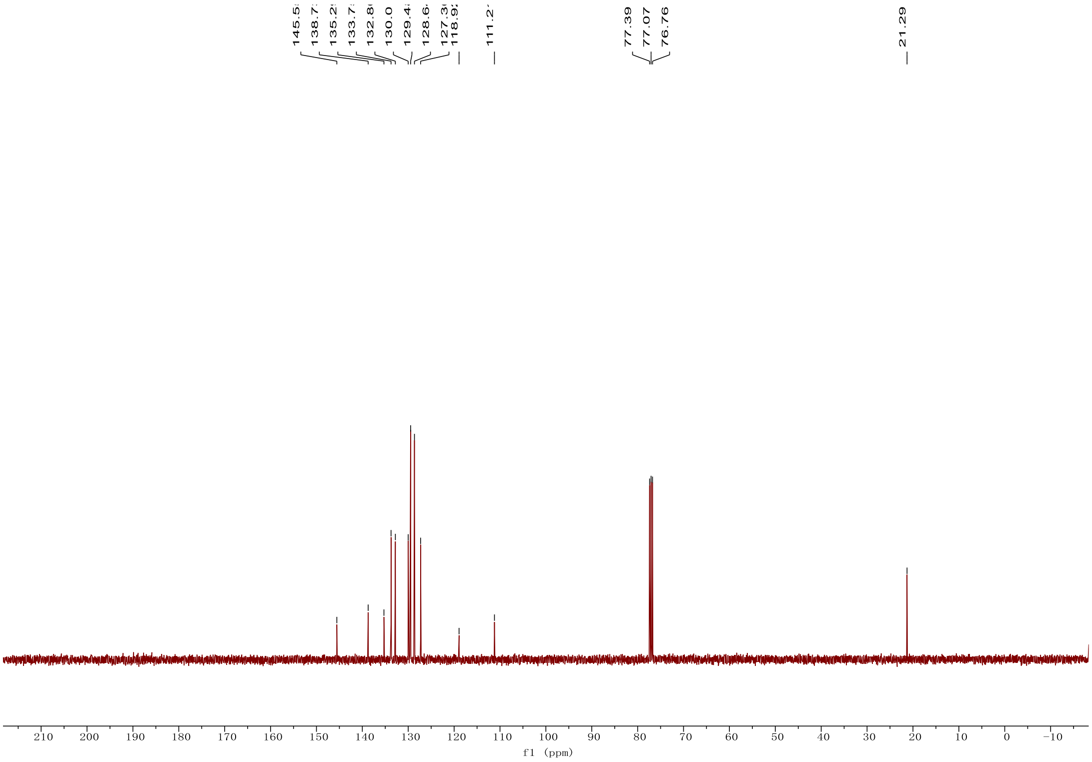

Supplementary Figure 142. ¹³C NMR (101 MHz, Chloroform-*d*) of **15**

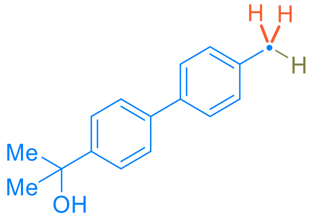

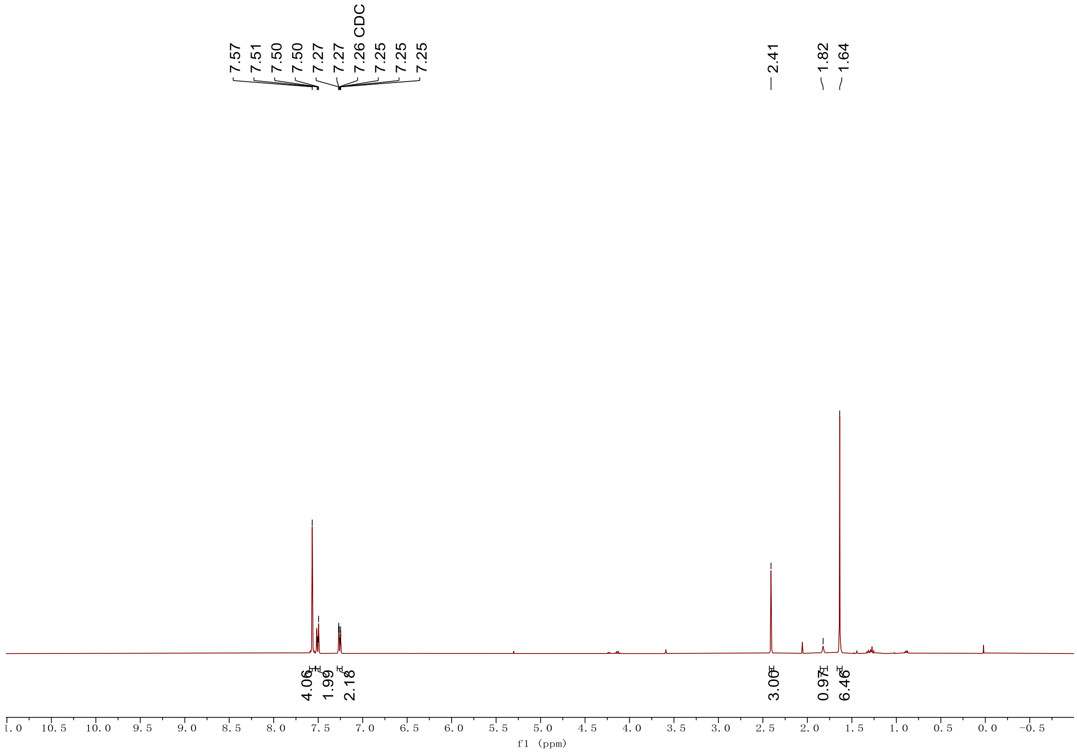

Supplementary Figure 143. ^{1}**H NMR** (400 MHz, Chloroform-*d*) of **16**

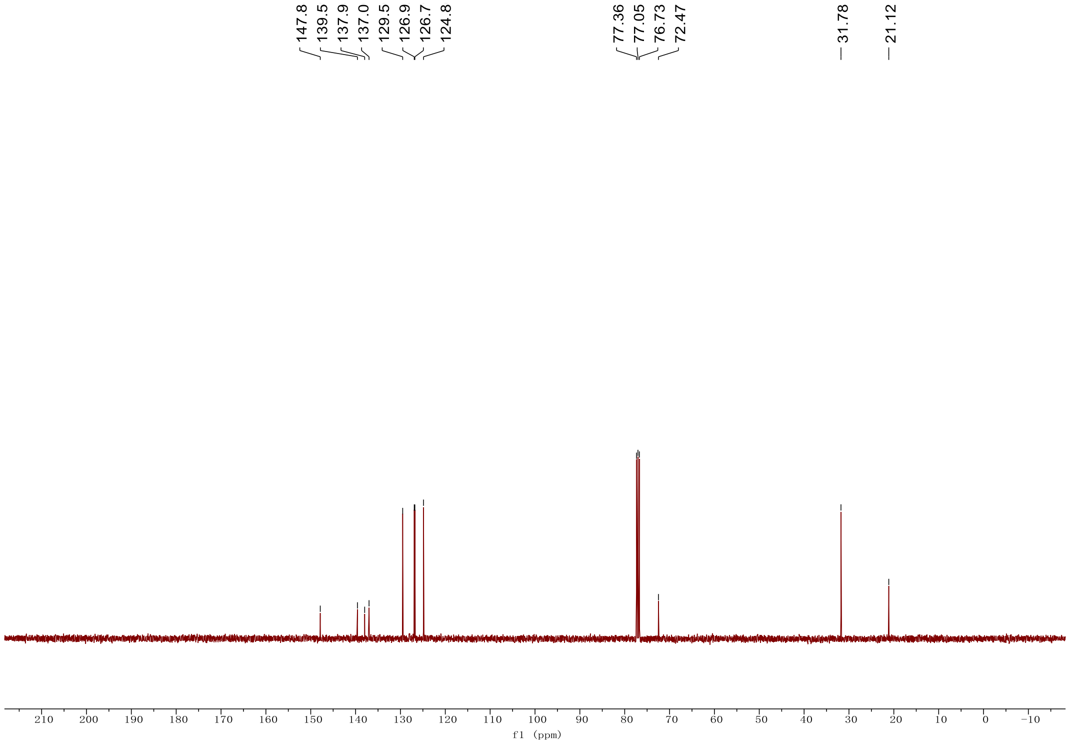

Supplementary Figure 144. ^{13}**C NMR (**101 MHz, Chloroform-*d*) of **16**

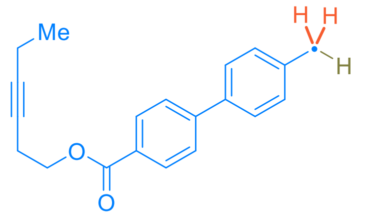

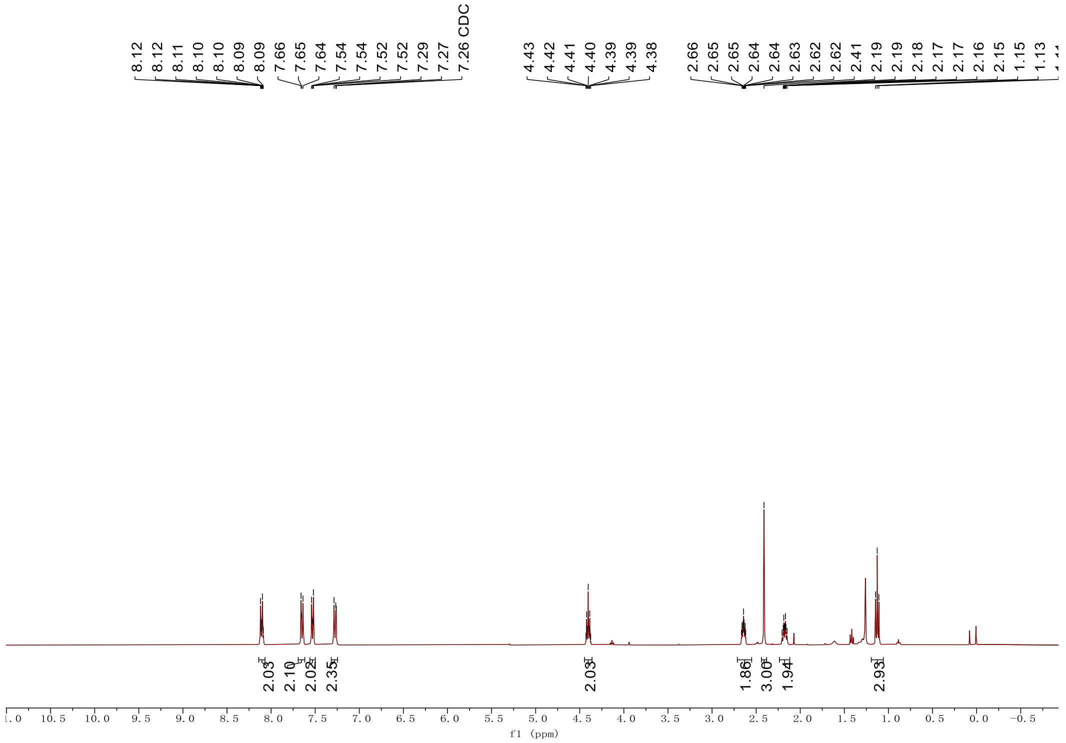

Supplementary Figure 145. ^{1}**H NMR** (400 MHz, Chloroform-*d*) of **17**

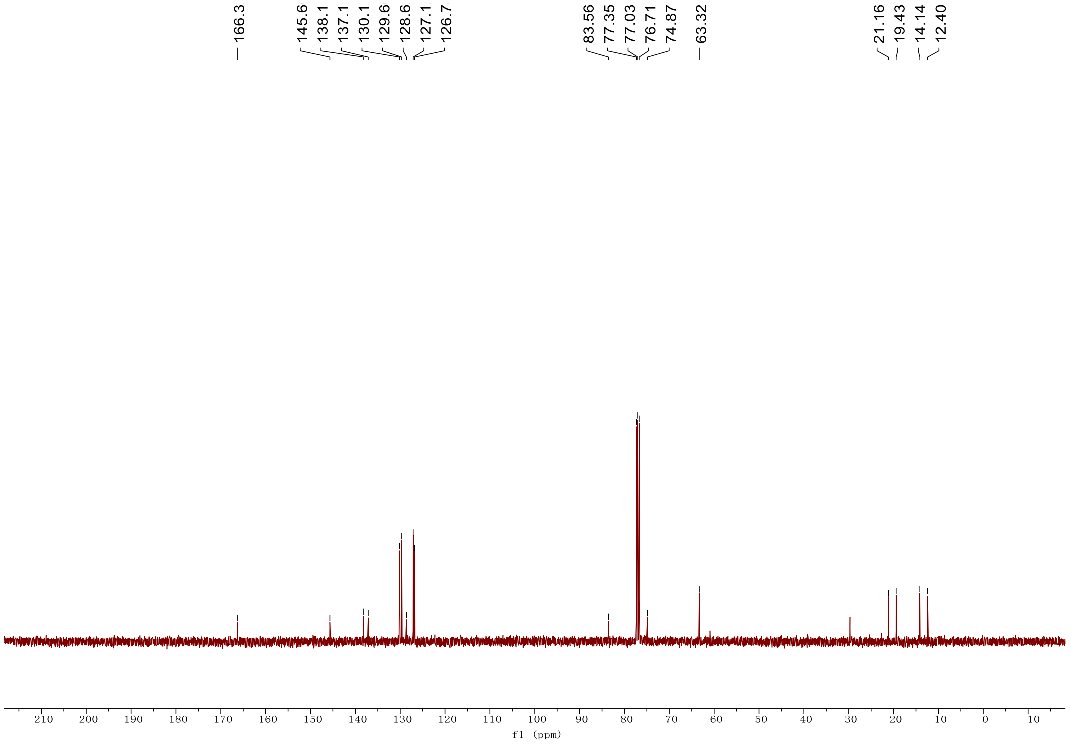

Supplementary Figure 146. ^{13}**C NMR (**101 MHz, Chloroform-*d*) of **17**

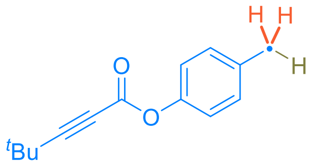

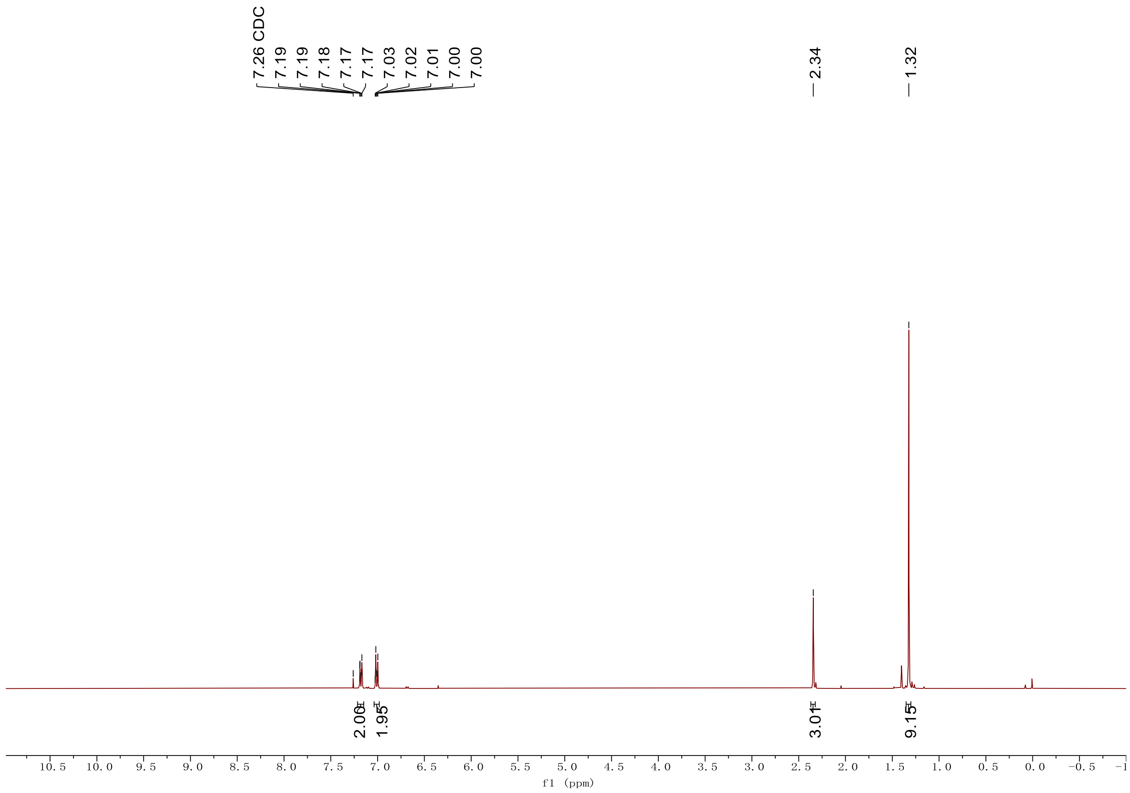

Supplementary Figure 147. ^{1}H NMR (400 MHz, Chloroform-*d*) of **18**

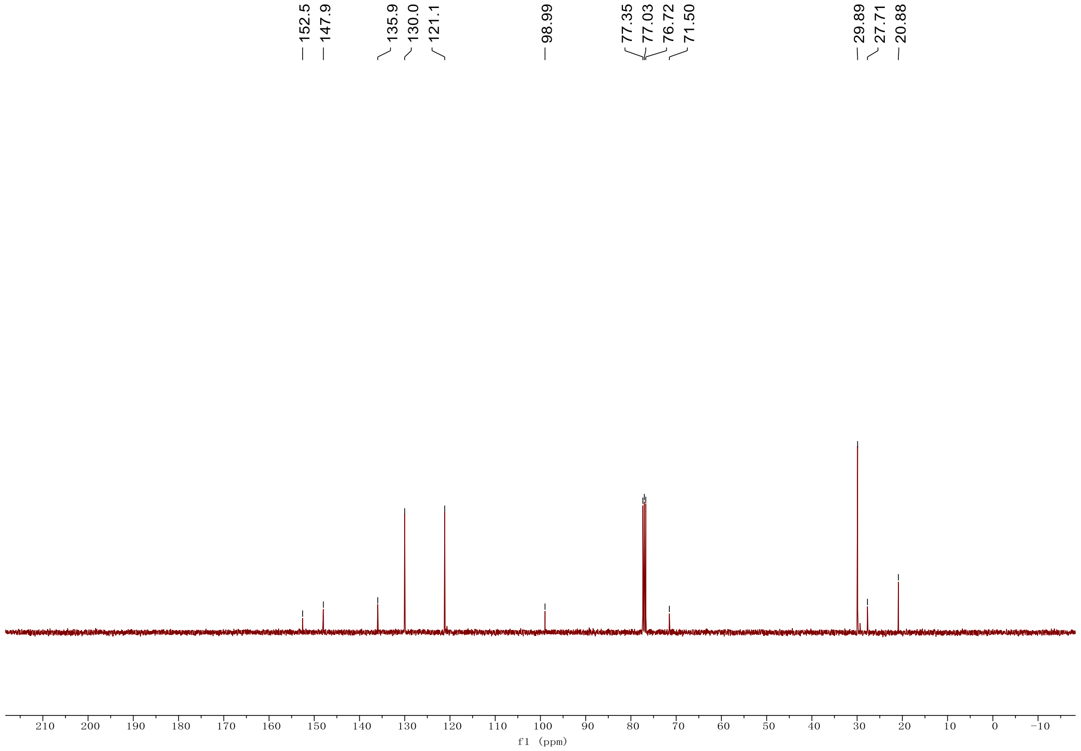

Supplementary Figure 148. ^{13}C NMR (101 MHz, Chloroform-*d*) of **18**

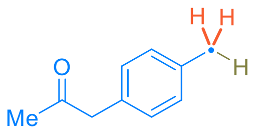

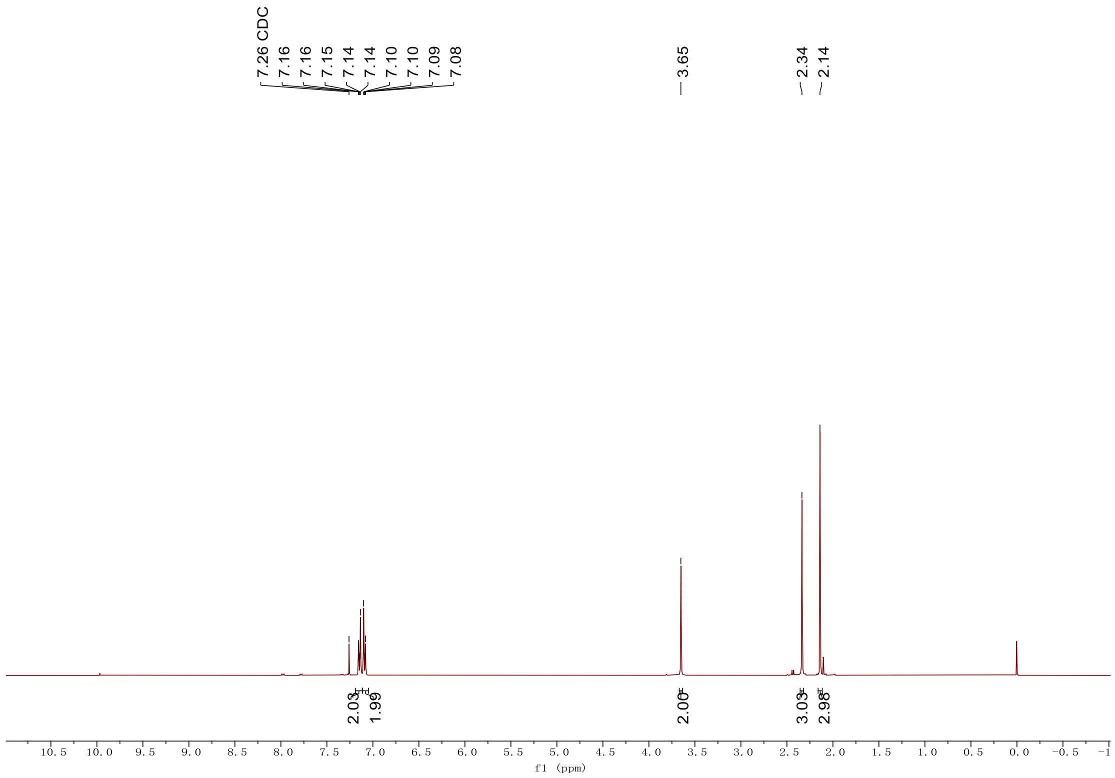

Supplementary Figure 149. ^{1}H NMR (400 MHz, Chloroform-*d*) of **19**

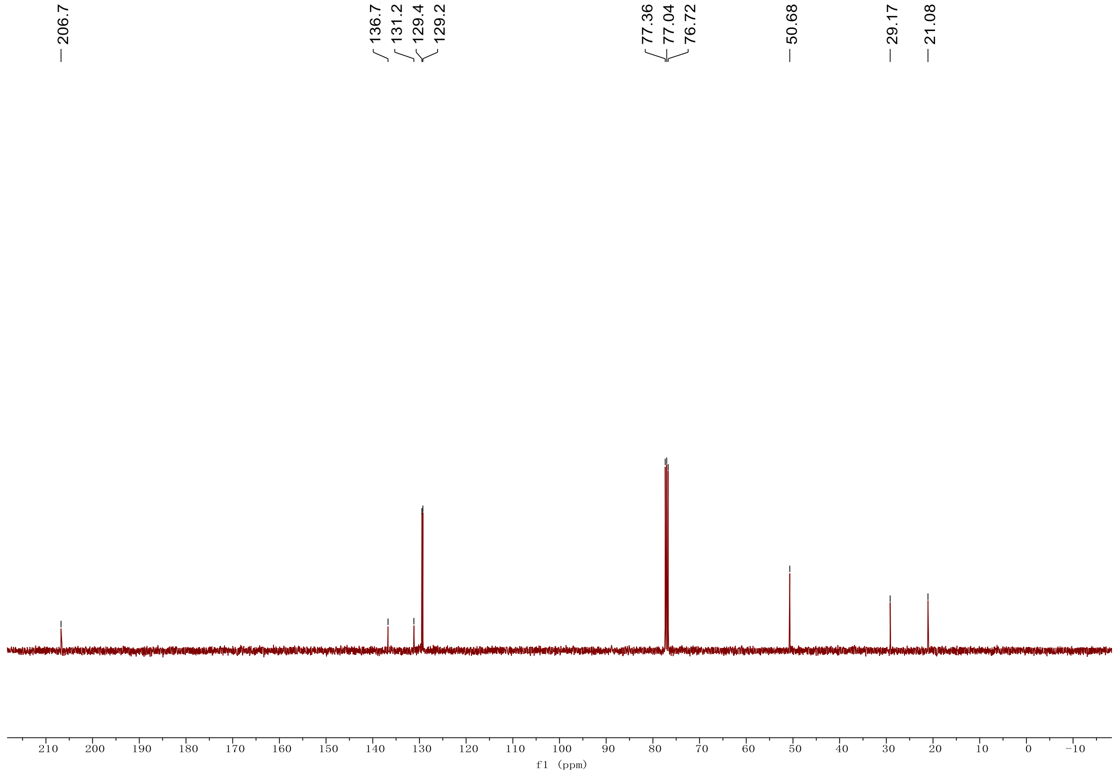

Supplementary Figure 150. ^{13}C NMR (101 MHz, Chloroform-*d*) of **19**

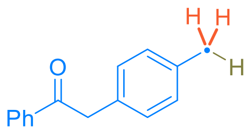

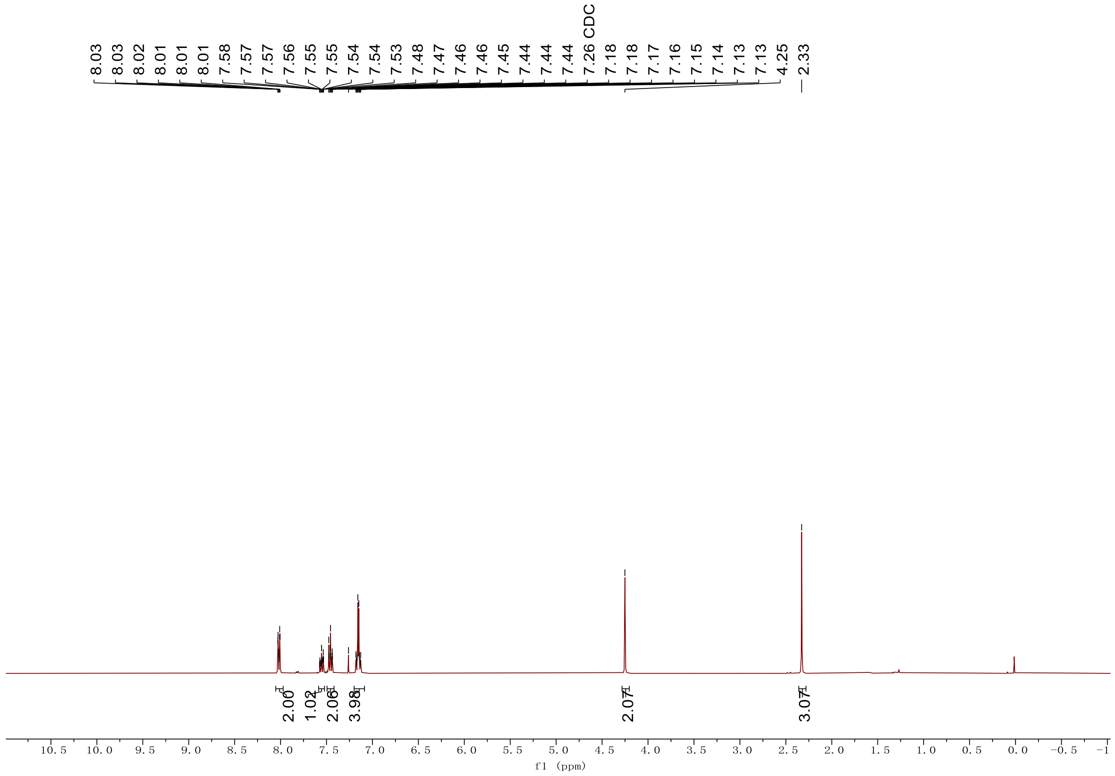

Supplementary Figure 151. ^{1}**H NMR** (400 MHz, Chloroform-*d*) of **20**

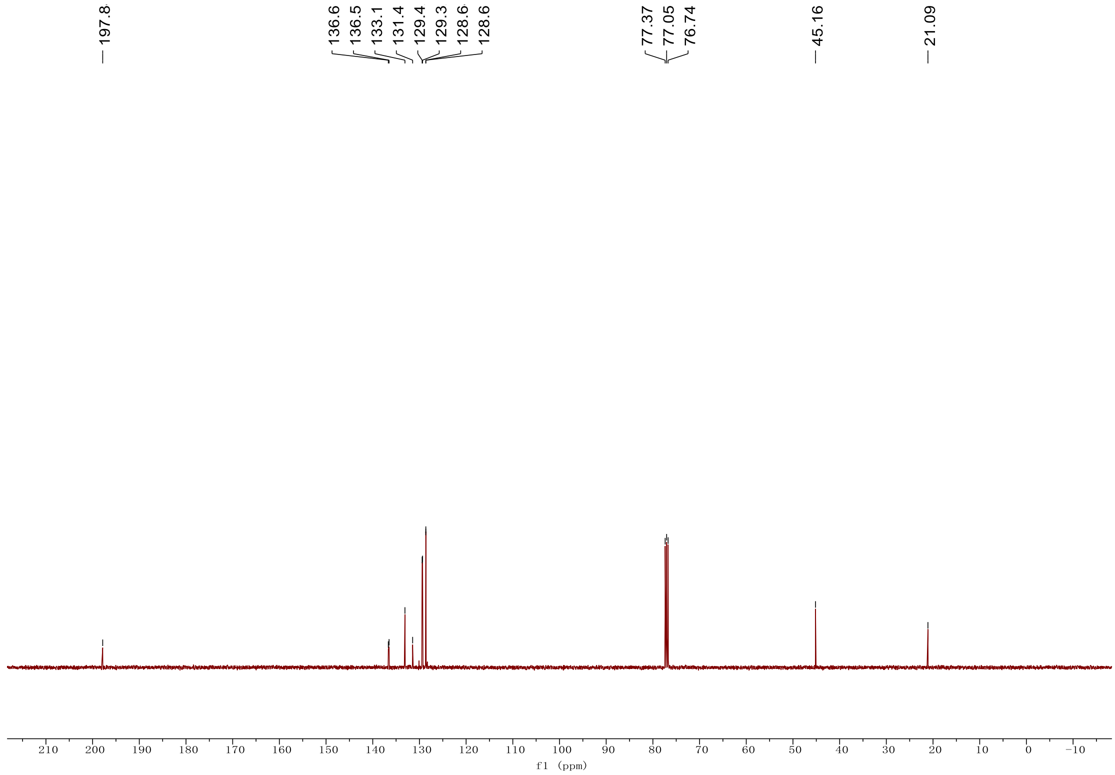

Supplementary Figure 152. ^{13}**C NMR** (101 MHz, Chloroform-*d*) of **20**

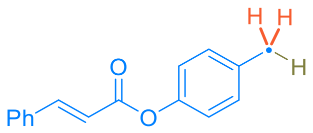

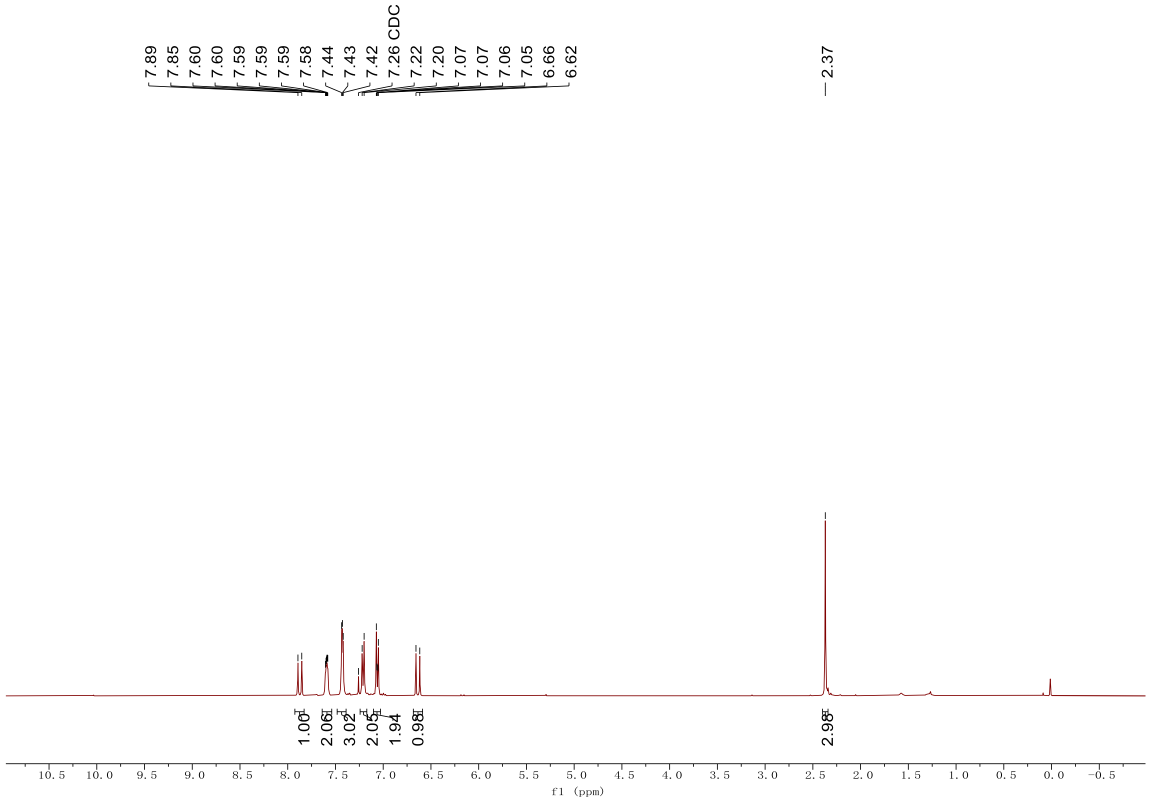

Supplementary Figure 153. **^{1}H NMR** (400 MHz, Chloroform-*d*) of **21**

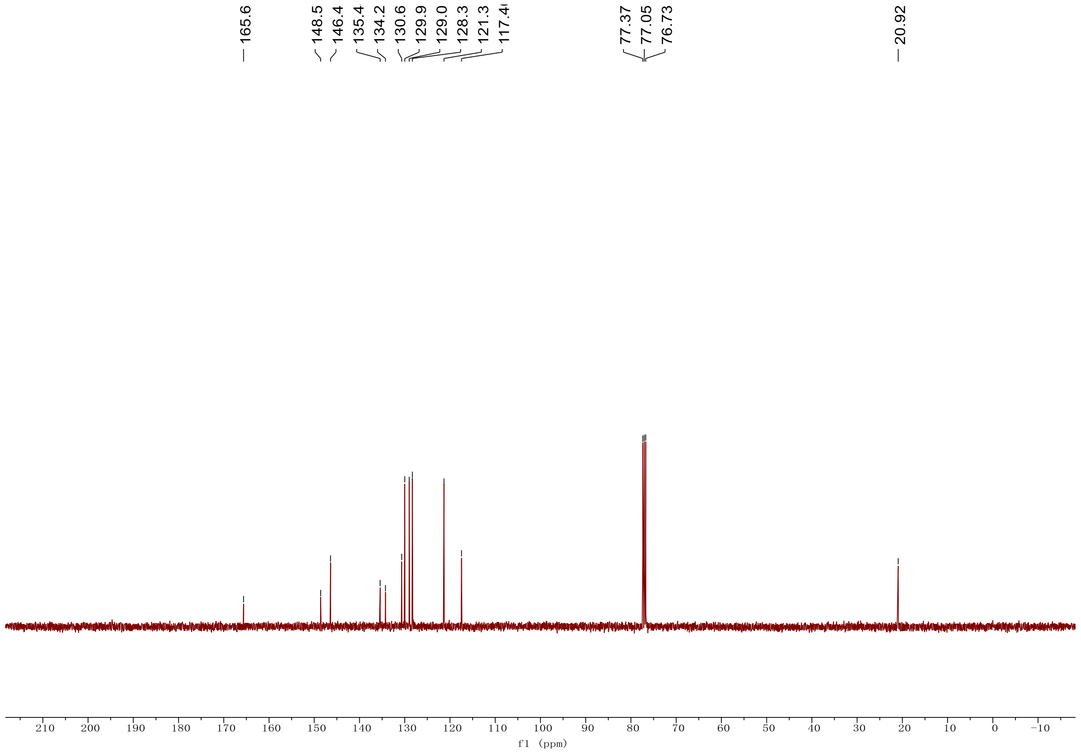

Supplementary Figure 154. **^{13}C NMR (**101 MHz, Chloroform-*d*) of **21**

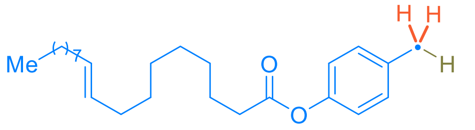

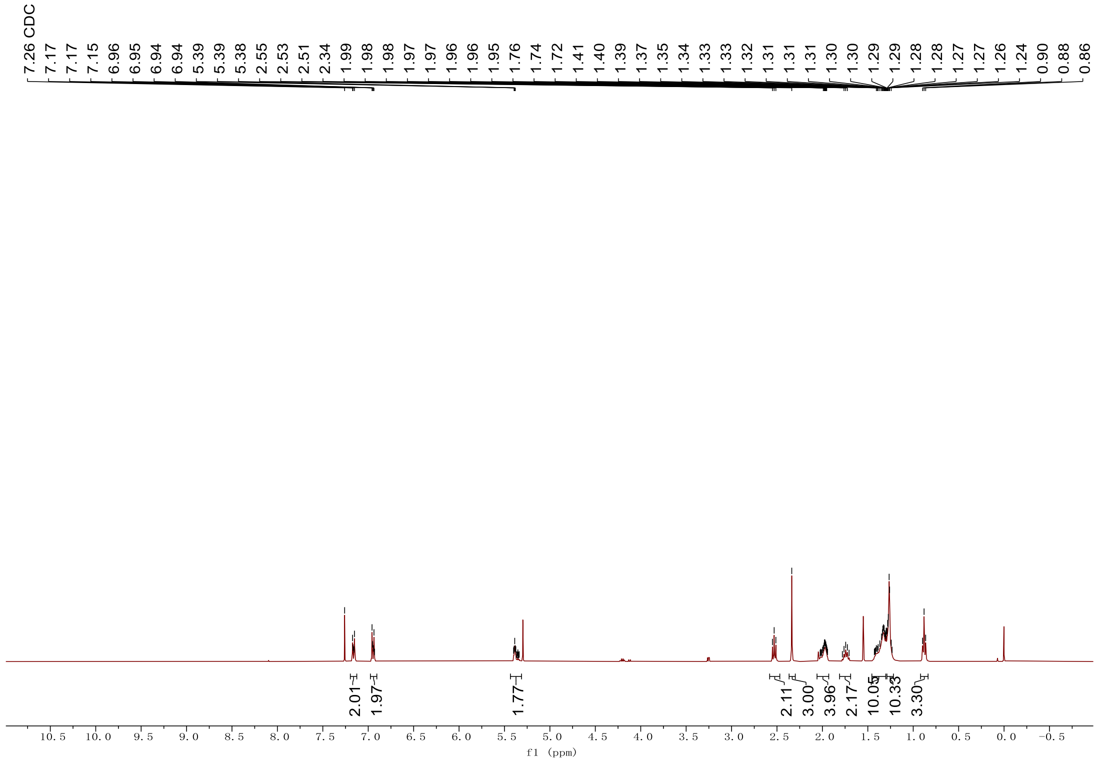

Supplementary Figure 155. ^{1}**H NMR** (400 MHz, Chloroform-*d*) of **22**

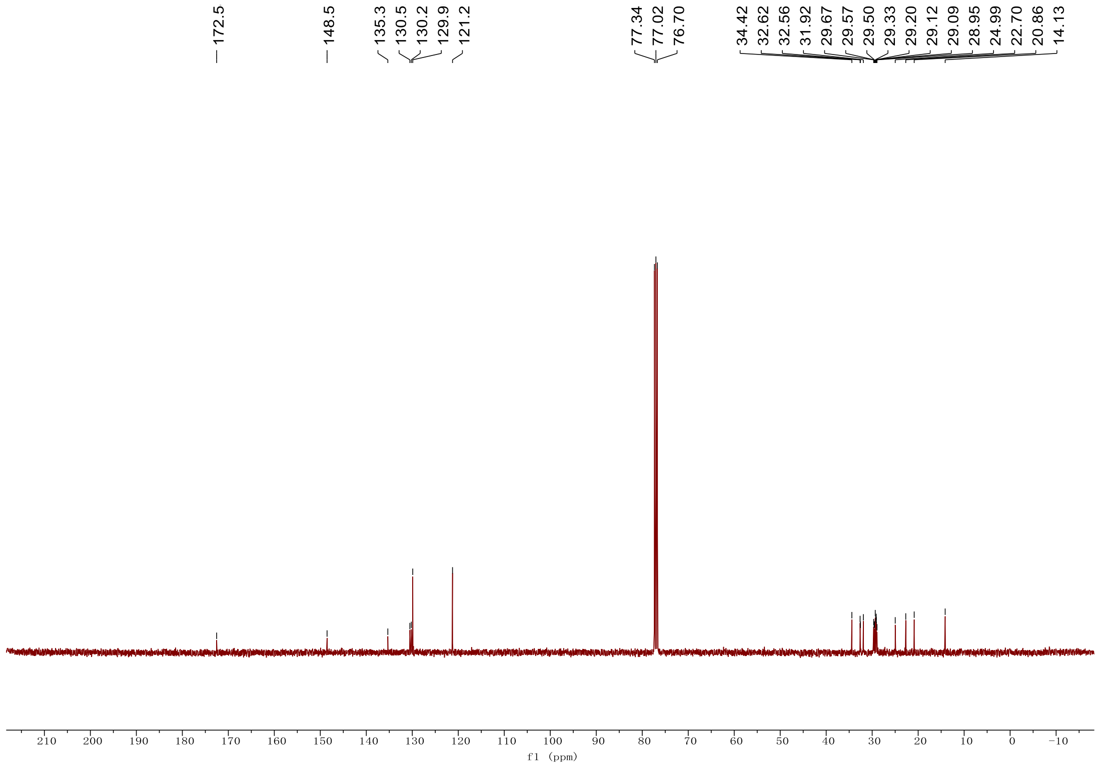

Supplementary Figure 156. ^{13}**C NMR** (101 MHz, Chloroform-*d*) of **22**

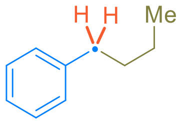

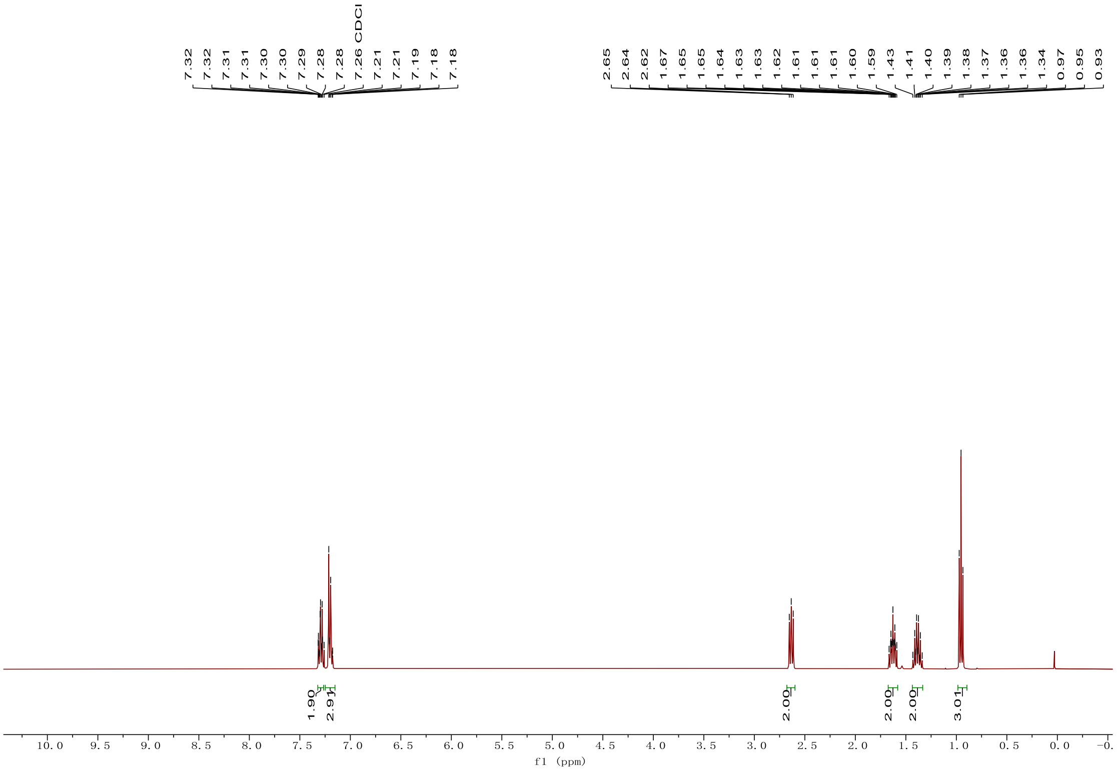

Supplementary Figure 157. ^{1}**H NMR** (400 MHz, Chloroform-*d*) of **23**

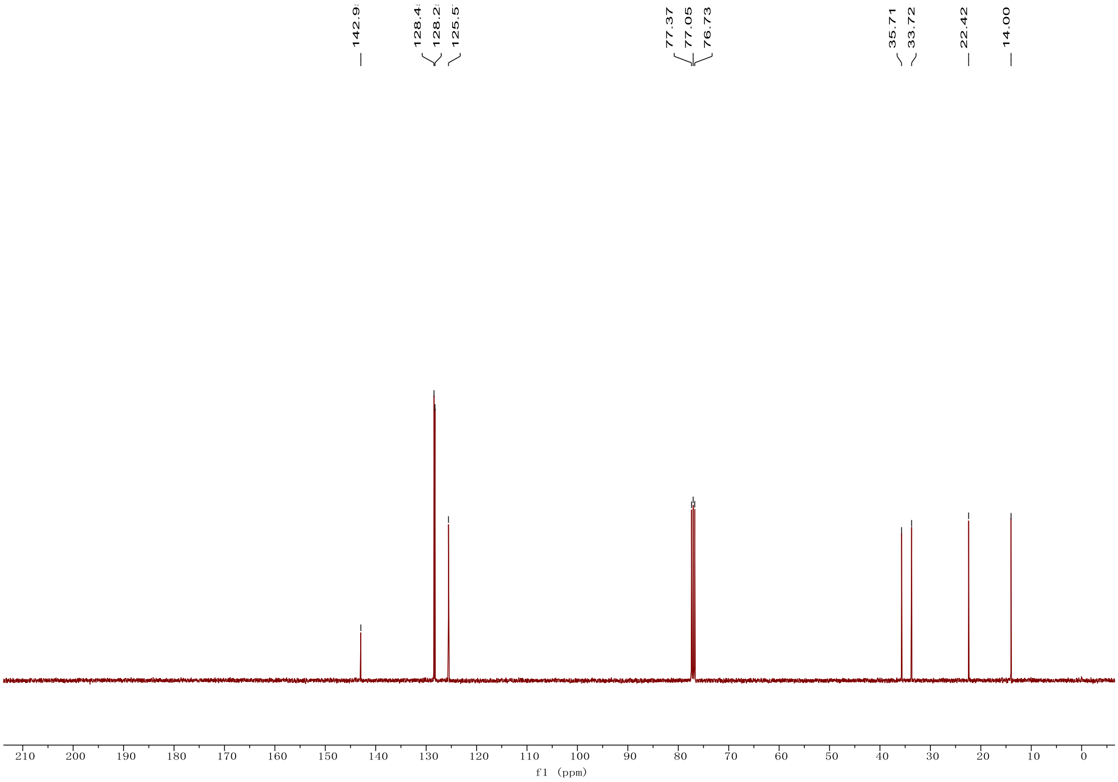

Supplementary Figure 158. ^{13}**C NMR (**101 MHz, Chloroform-*d*) of **23**

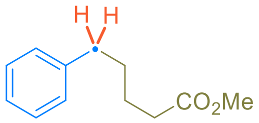

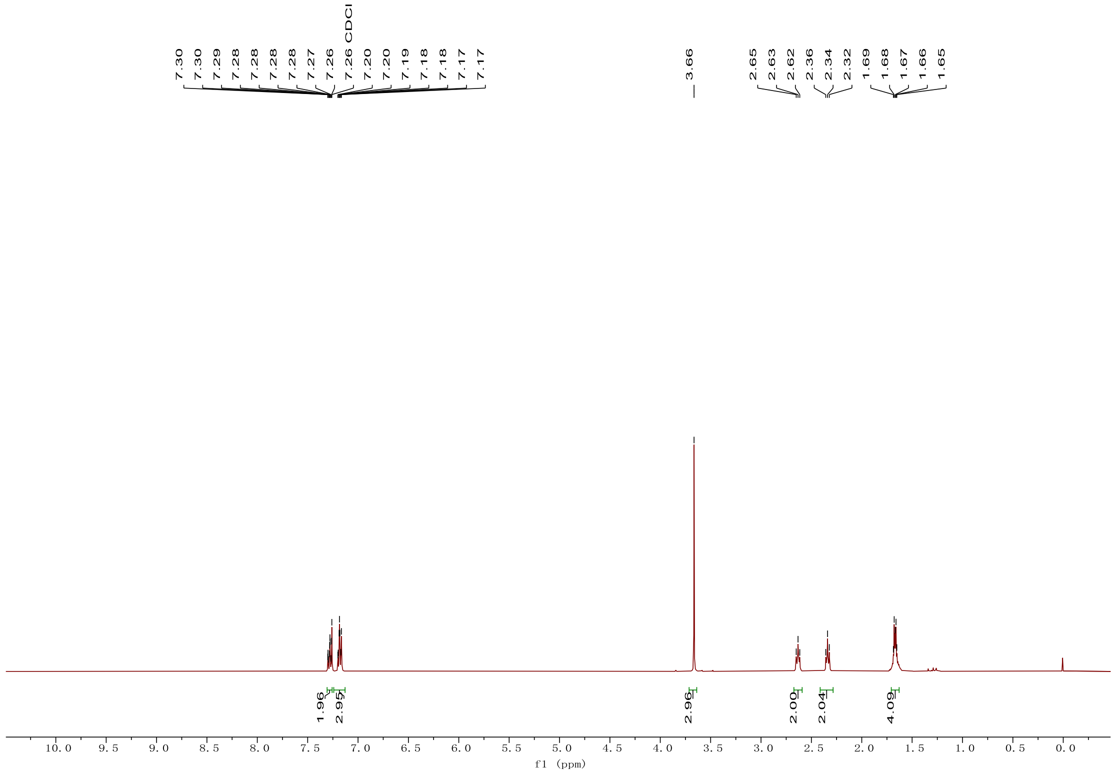

Supplementary Figure 159. ^{1}H NMR (400 MHz, Chloroform-*d*) of **24**

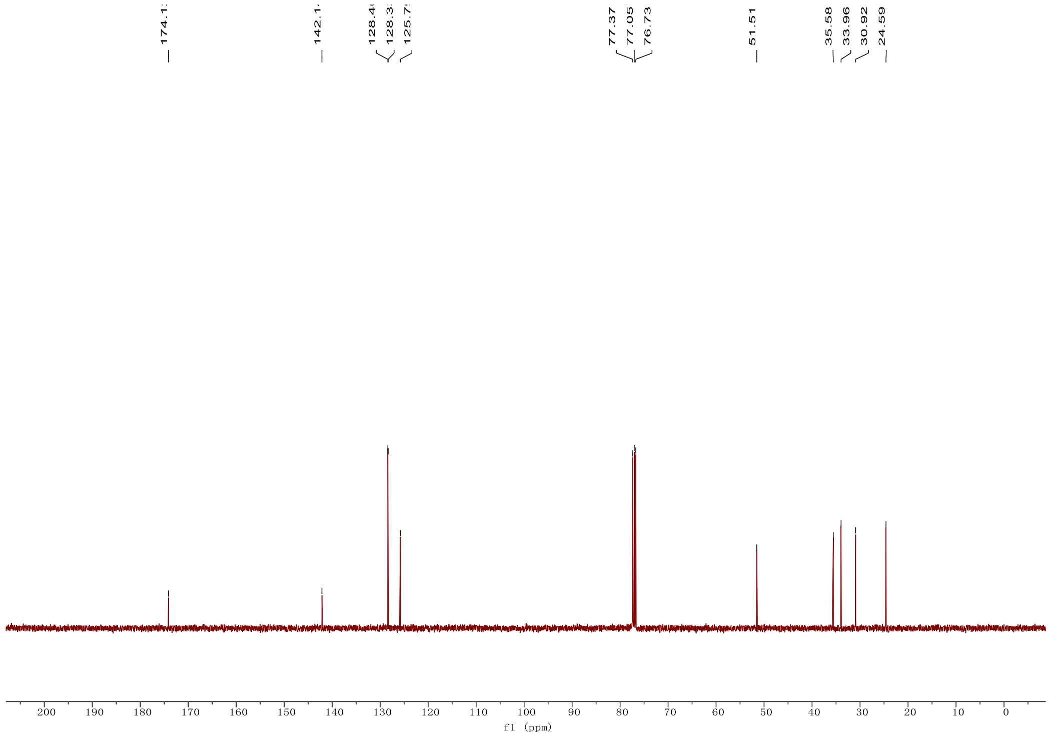

Supplementary Figure 160. ^{13}C NMR (101 MHz, Chloroform-*d*) of **24**

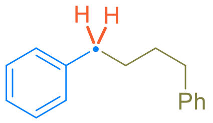

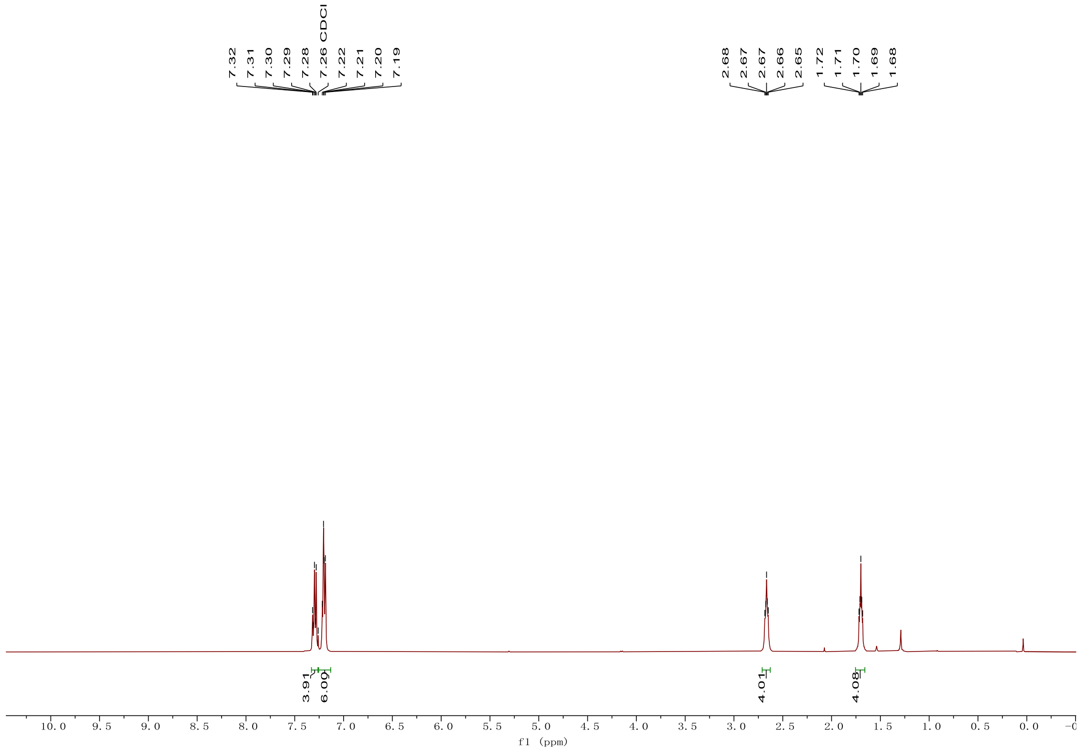

Supplementary Figure 161. ^{1}H NMR (400 MHz, Chloroform-*d*) of **25**

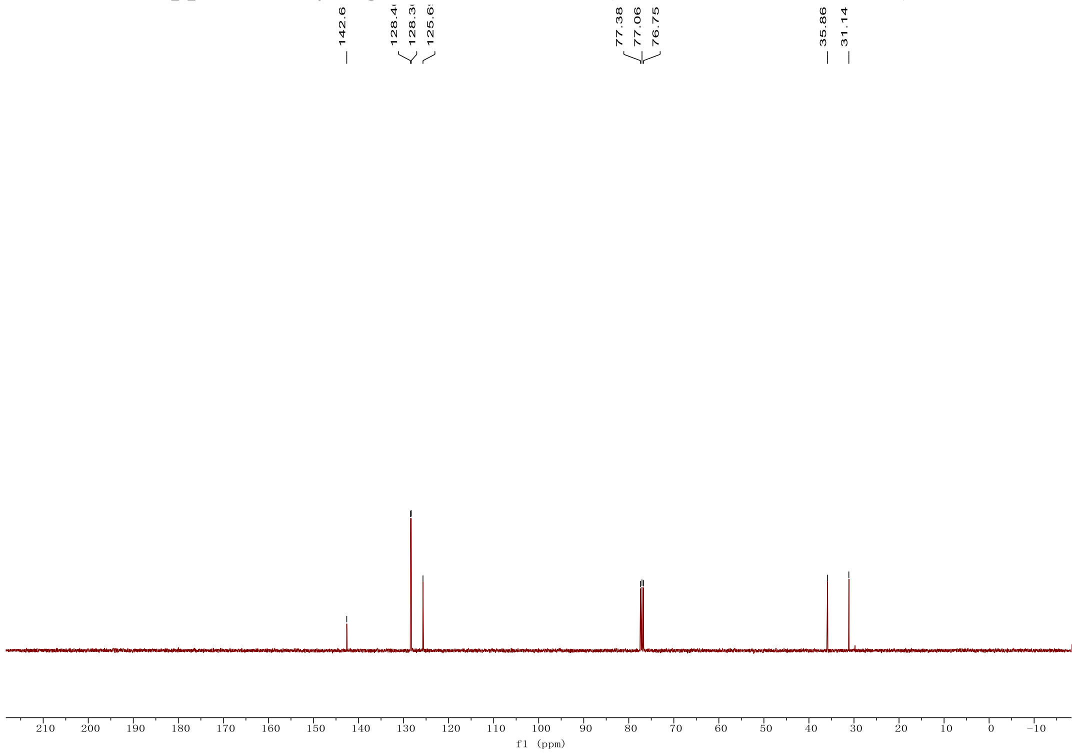

Supplementary Figure 162. ^{13}C NMR (101 MHz, Chloroform-*d*) of **25**

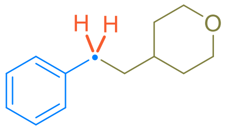

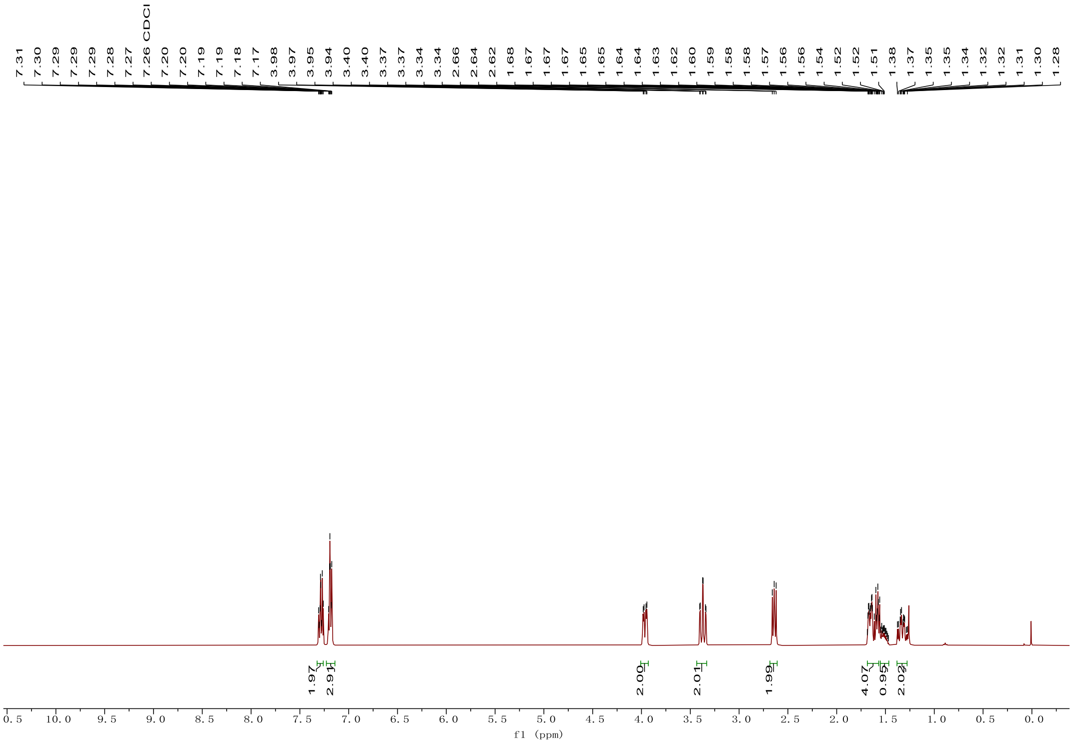

Supplementary Figure 163. ^{1}H NMR (400 MHz, Chloroform-*d*) of **26**

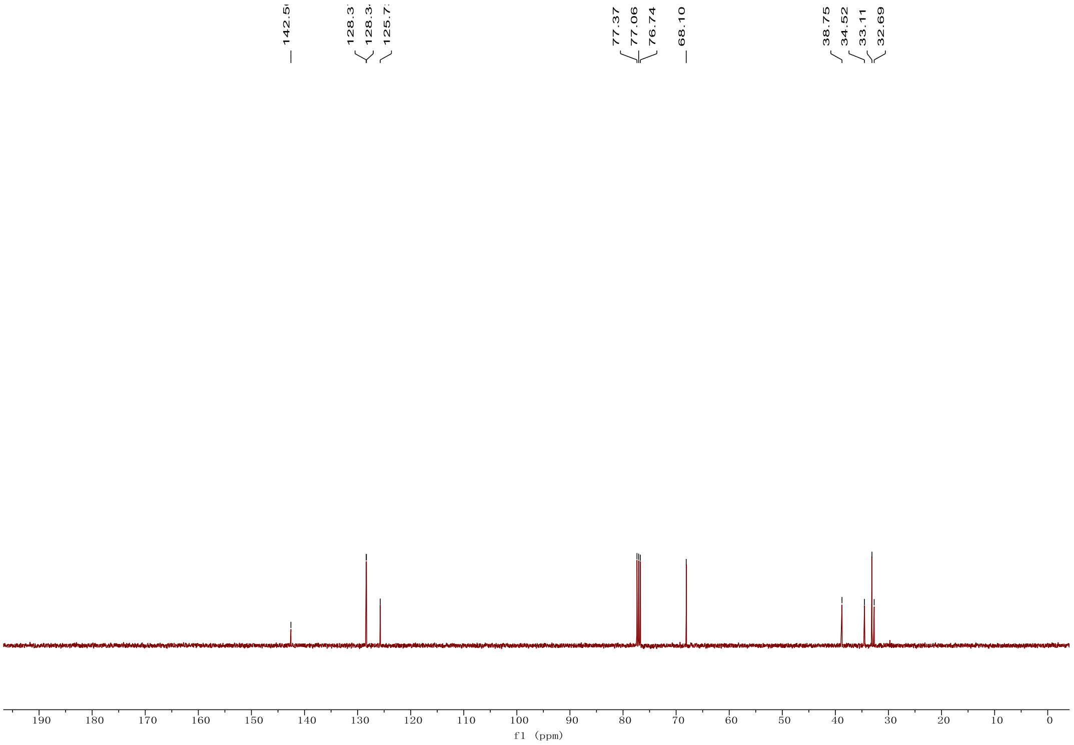

Supplementary Figure 164. ^{13}C NMR (101 MHz, Chloroform-*d*) of **26**

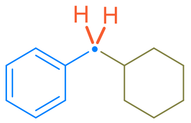

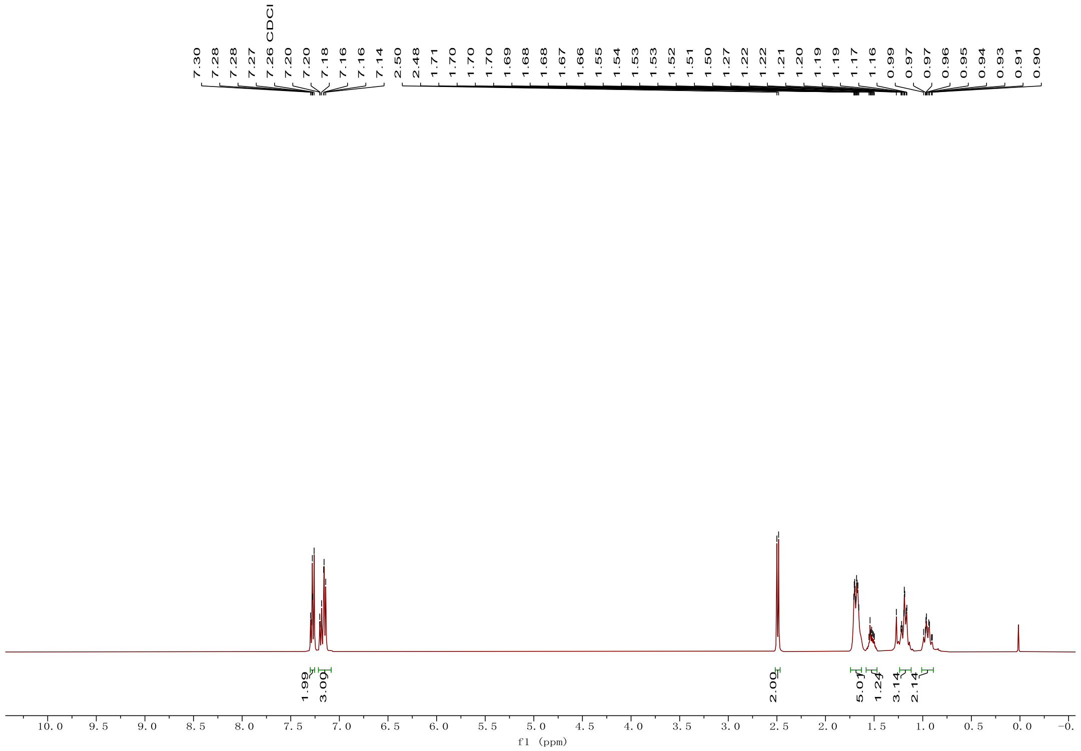

Supplementary Figure 165. ^{1}**H NMR** (400 MHz, Chloroform-*d*) of **27**

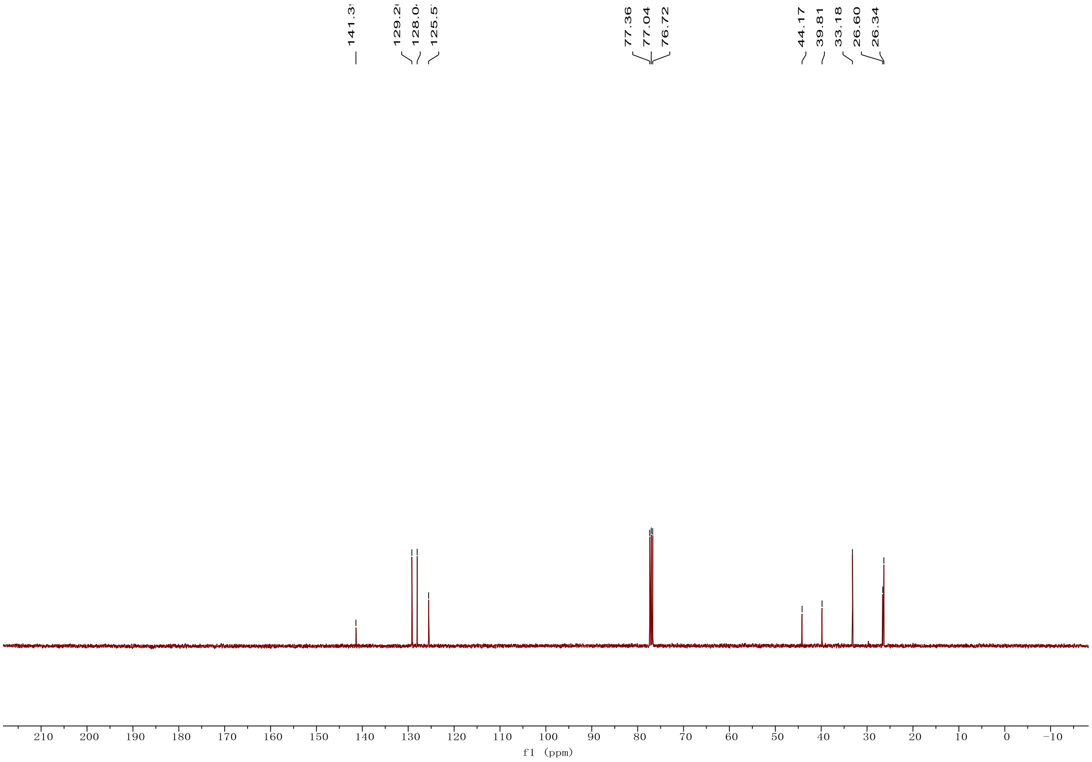

Supplementary Figure 166. ^{13}**C NMR (**101 MHz, Chloroform-*d*) of **27**

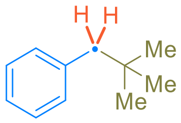

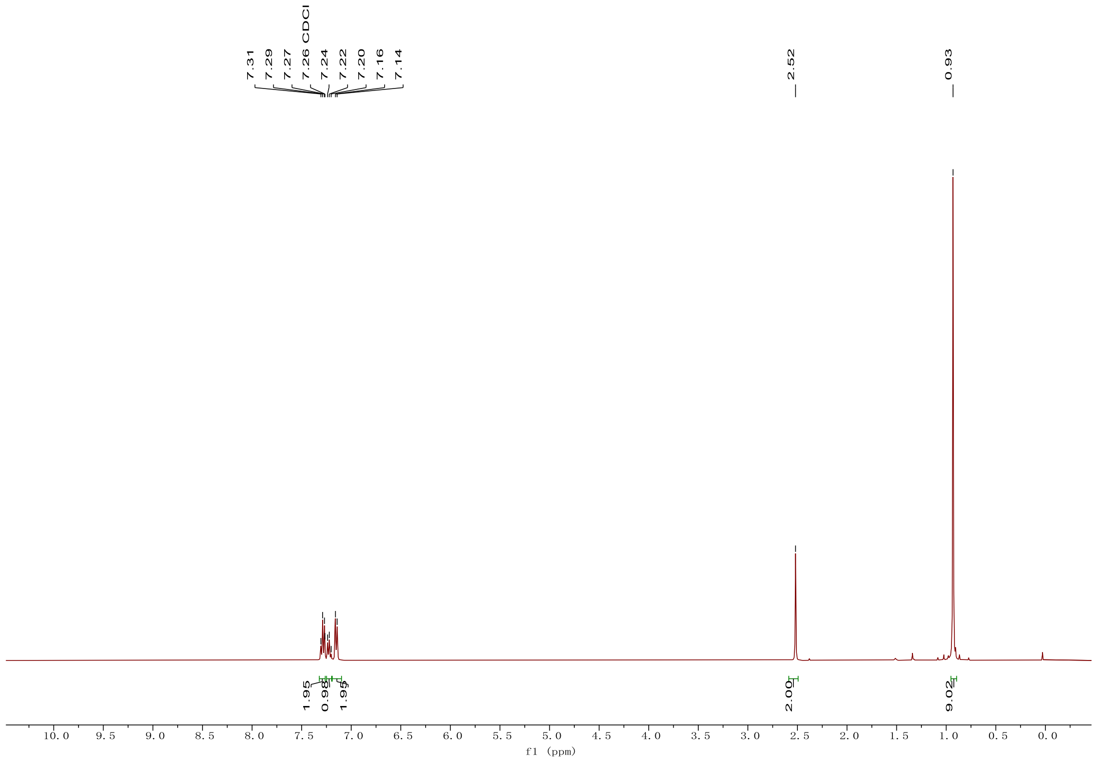

Supplementary Figure 167. ^{1}H NMR (400 MHz, Chloroform-*d*) of **28**

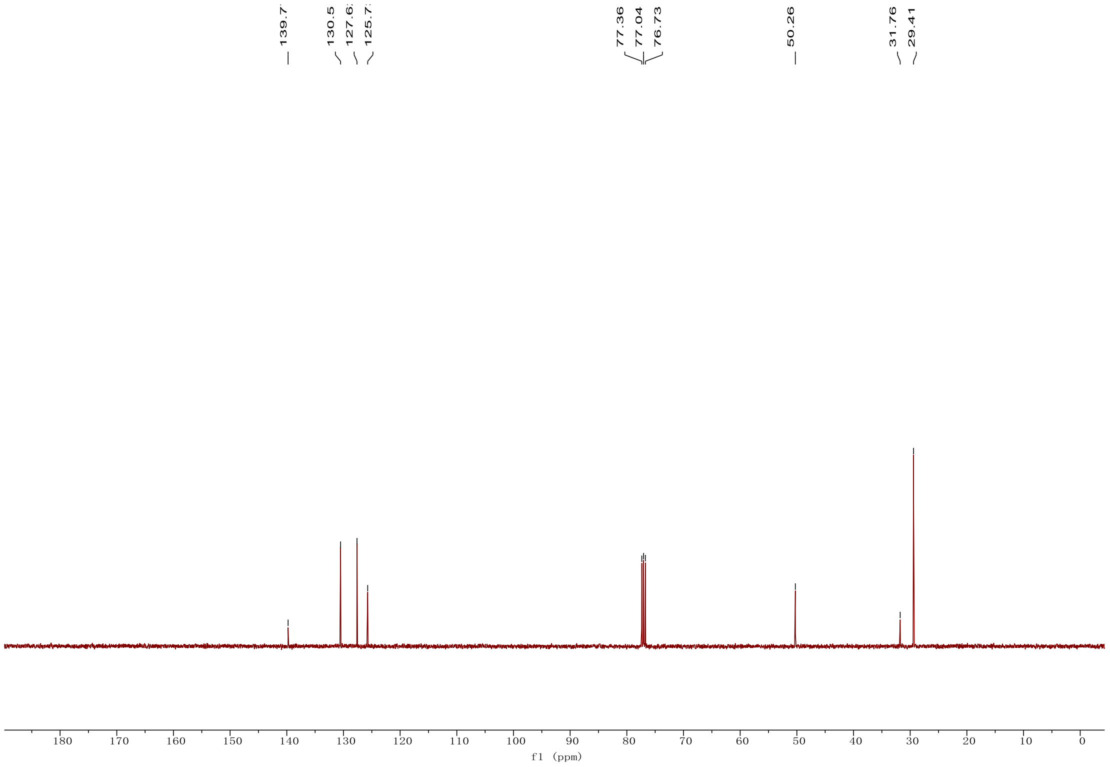

Supplementary Figure 168. ^{13}C NMR (101 MHz, Chloroform-*d*) of **28**

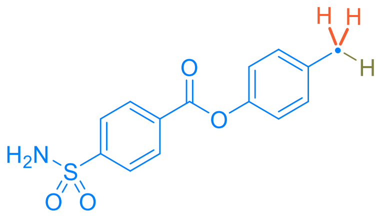

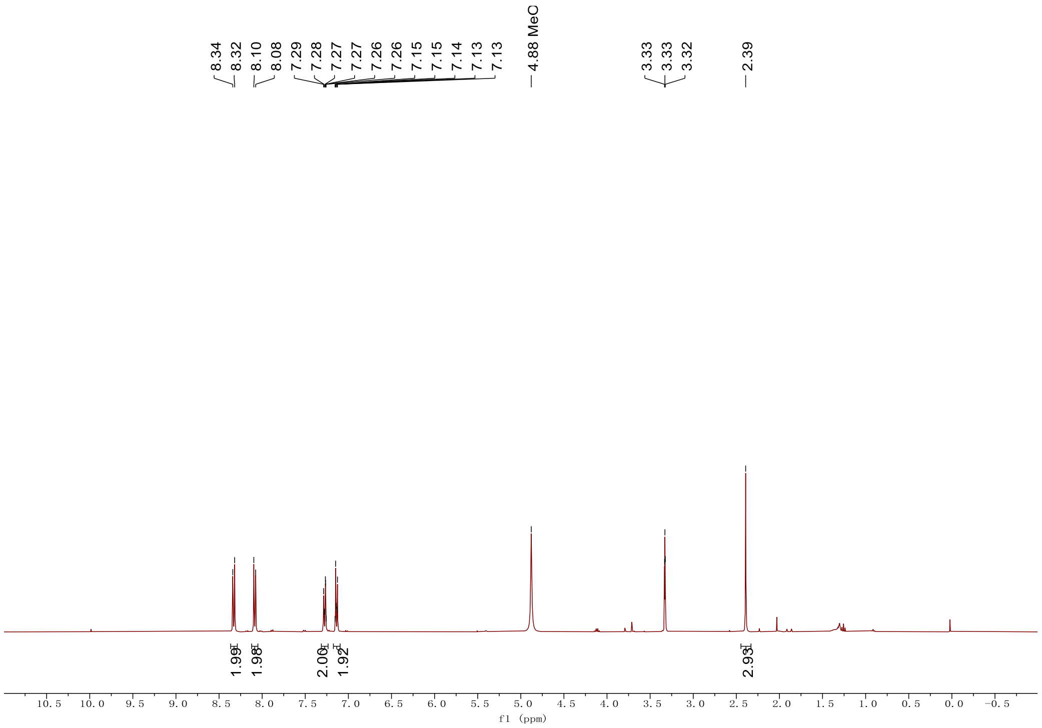

Supplementary Figure 169. ^{1}H NMR (400 MHz, Methanol-d_4) of **29**

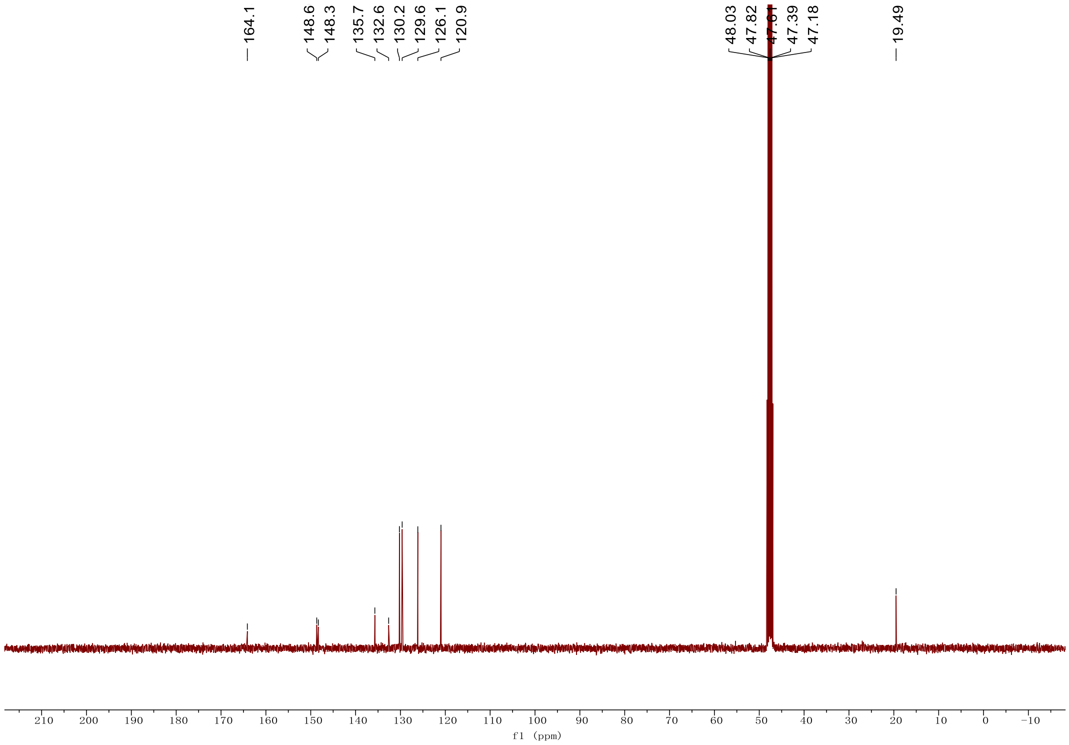

Supplementary Figure 170. ^{13}C NMR (101 MHz, Methanol-d_4) of **29**

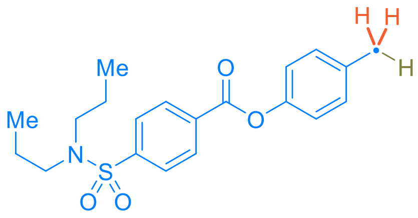

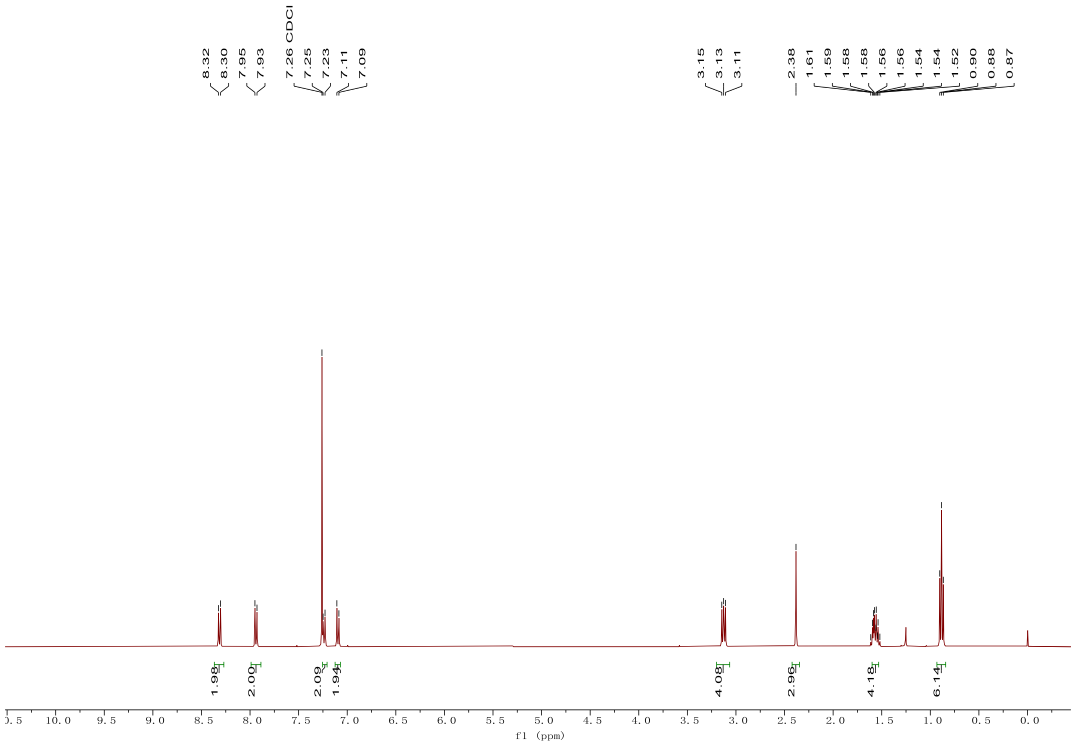

Supplementary Figure 171. ^{1}**H NMR** (400 MHz, Chloroform-*d*) of **30**

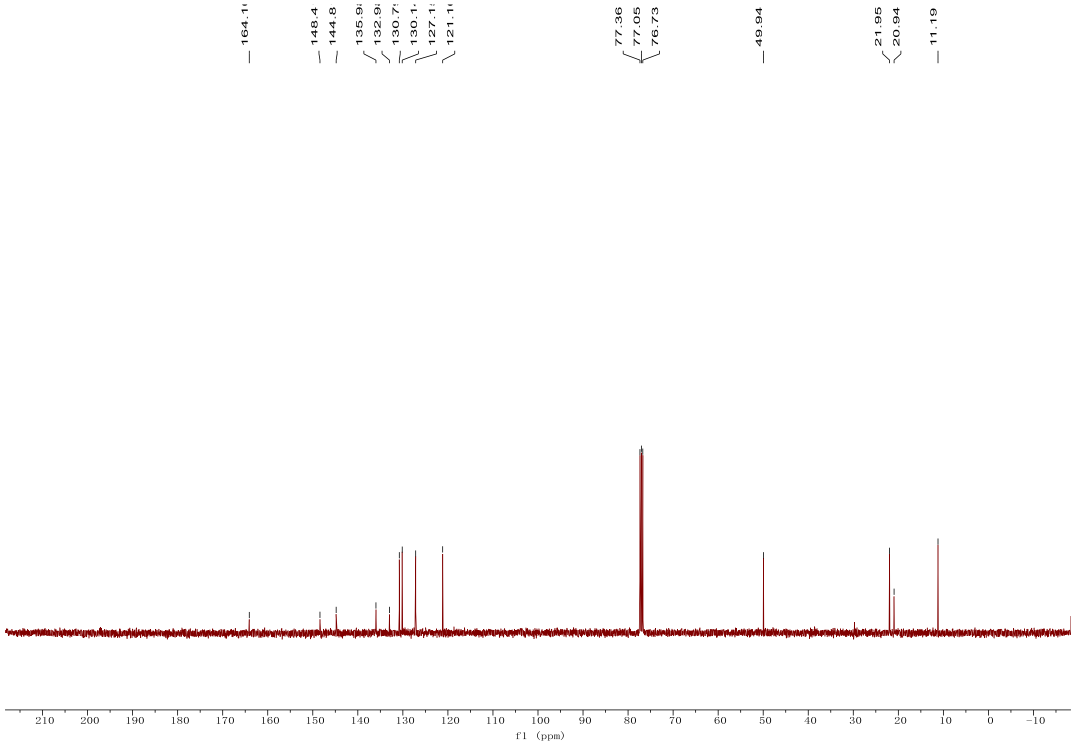

Supplementary Figure 172. ^{13}**C NMR (**101 MHz, Chloroform-*d*) of **30**

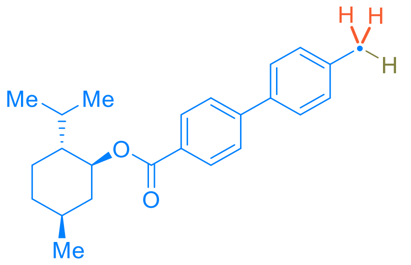

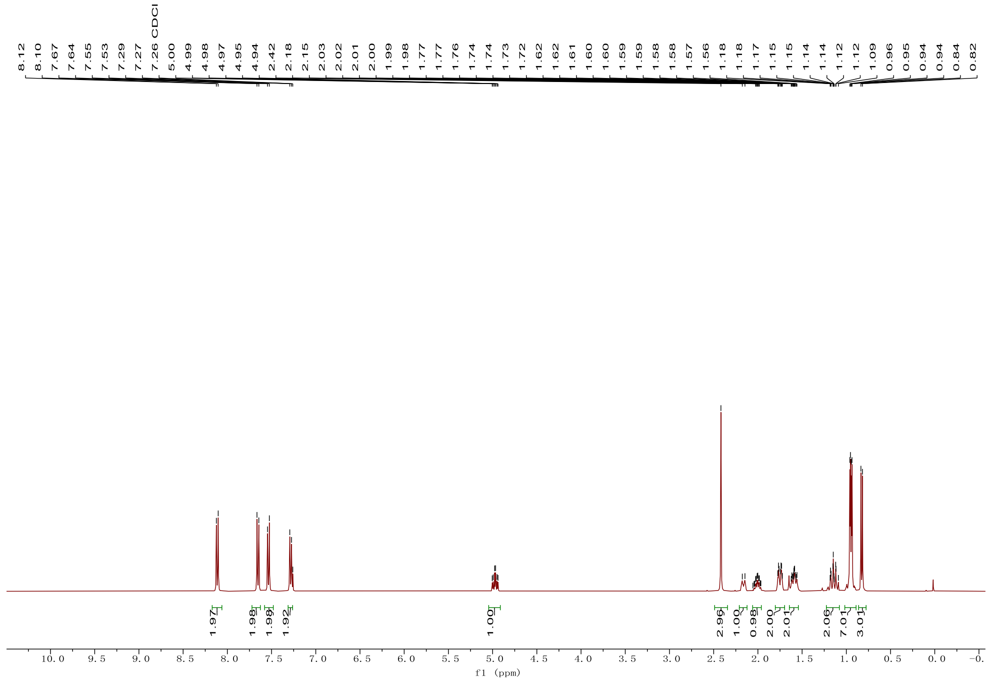

Supplementary Figure 173. ^{1}**H NMR** (400 MHz, Chloroform-*d*) of **31**

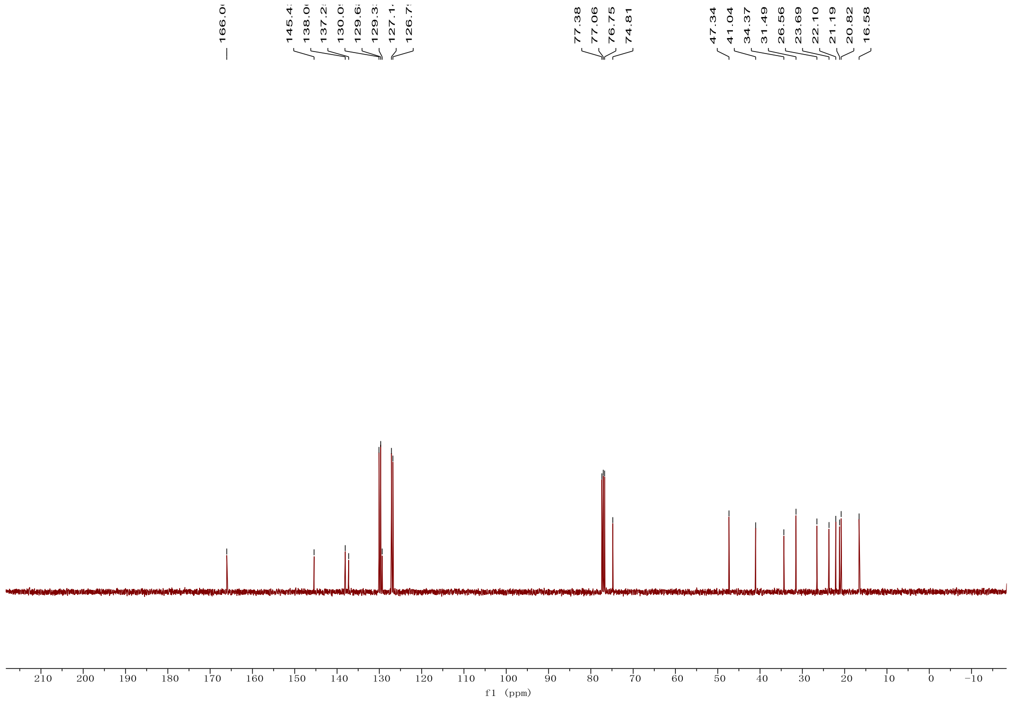

Supplementary Figure 174. ^{13}**C NMR (**101 MHz, Chloroform-*d*) of **31**

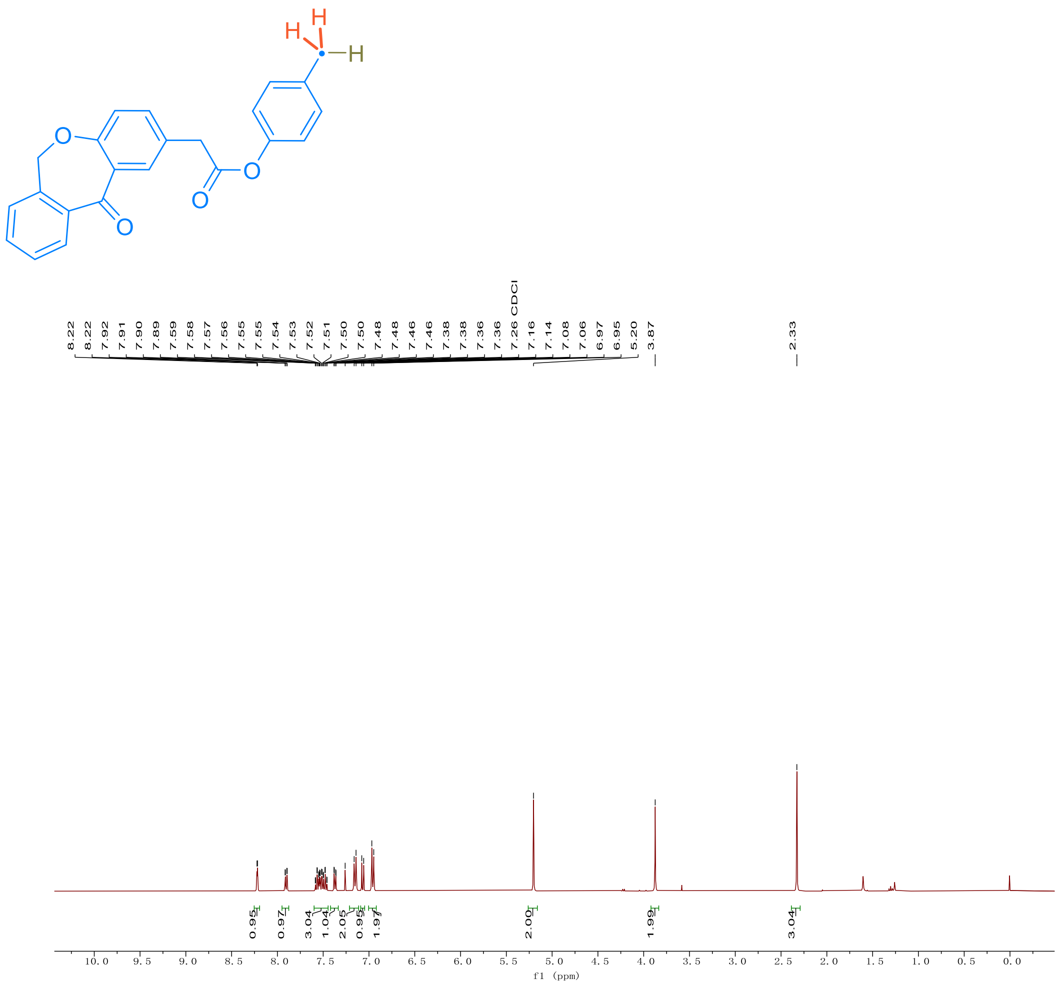

Supplementary Figure 175. ¹H NMR (400 MHz, Chloroform-*d*) of **32**

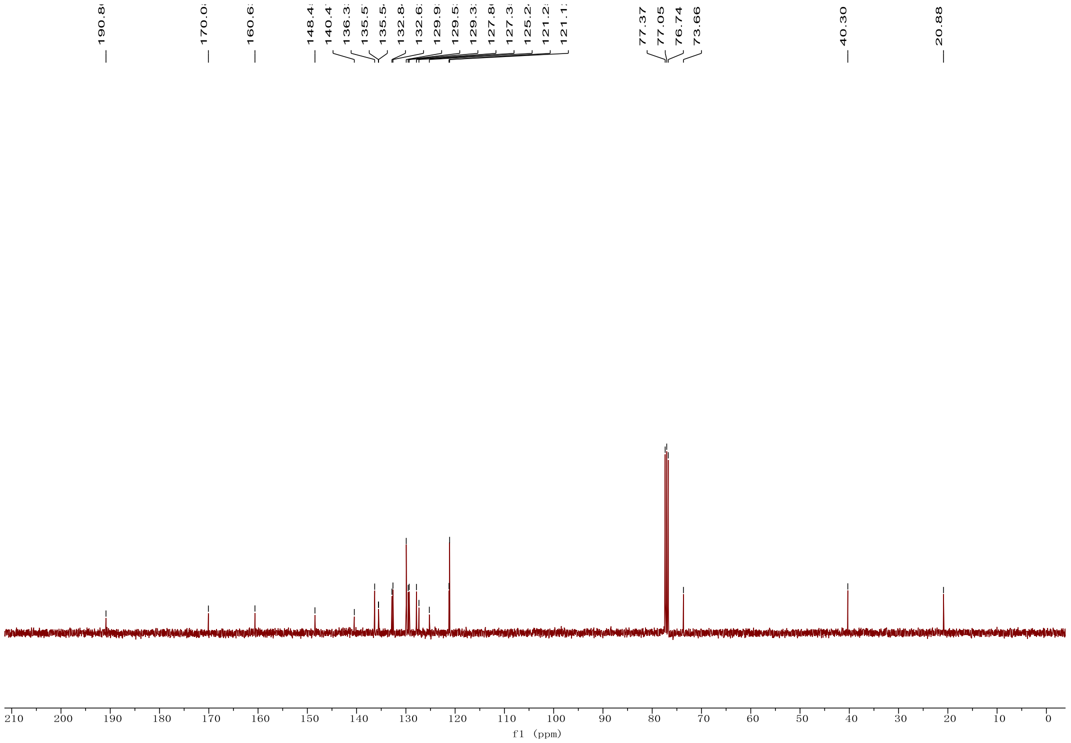

Supplementary Figure 176. ¹³C NMR (101 MHz, Chloroform-*d*) of **32**

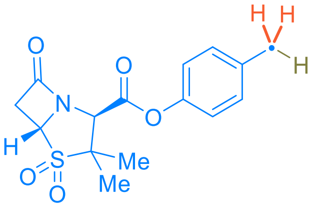

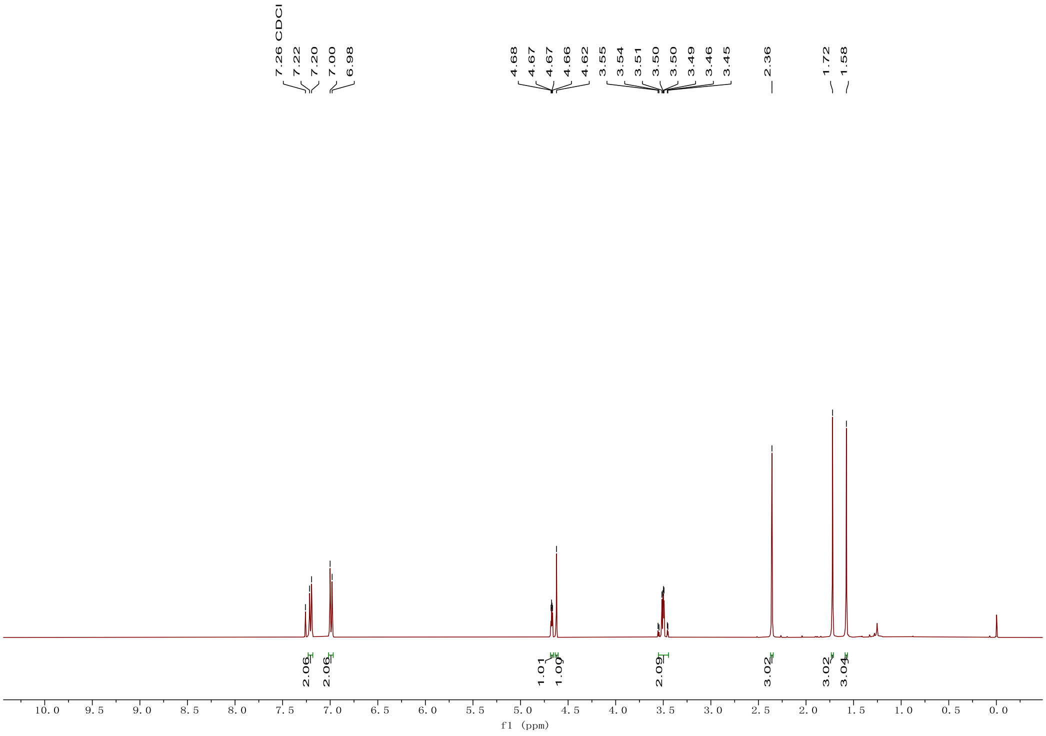

Supplementary Figure 177. ^{1}**H NMR** (400 MHz, Chloroform-*d*) of **33**

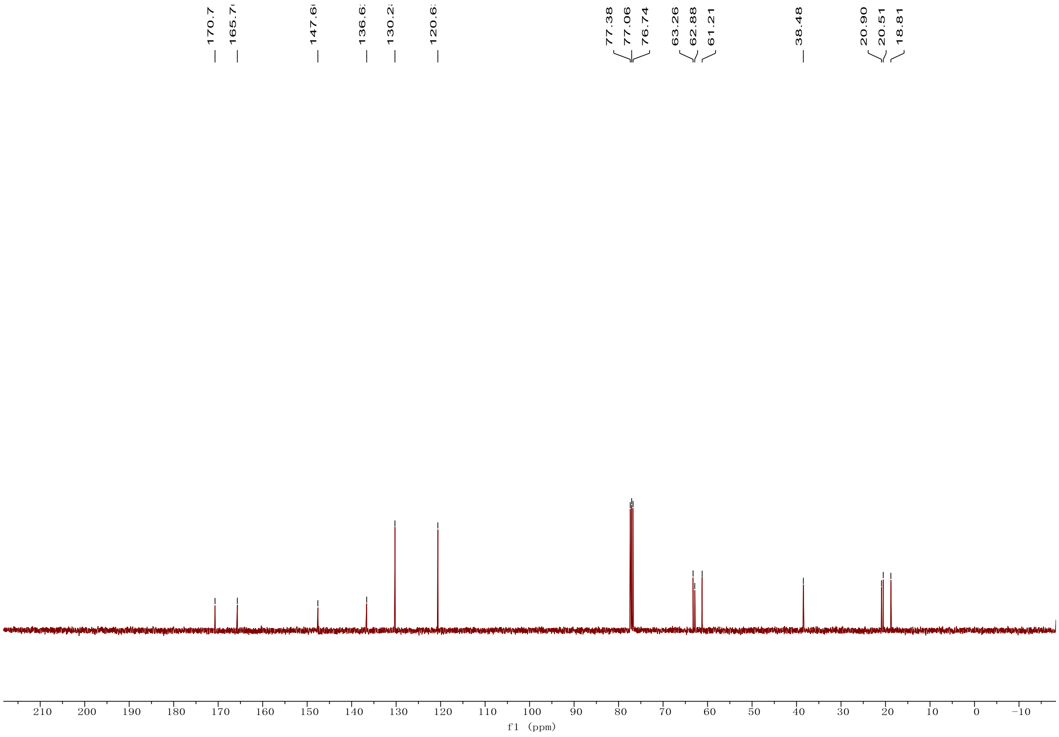

Supplementary Figure 178. ^{13}**C NMR (**101 MHz, Chloroform-*d*) of **33**

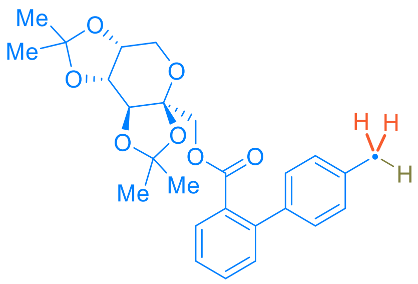

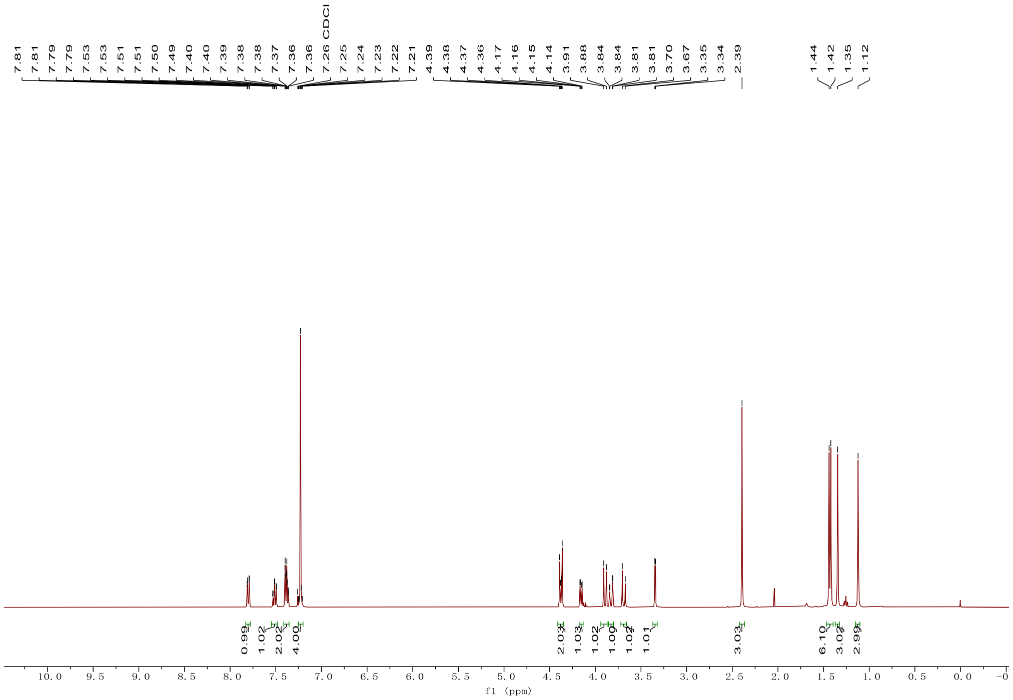

Supplementary Figure 179. ^{1}**H NMR** (400 MHz, Chloroform-*d*) of **34**

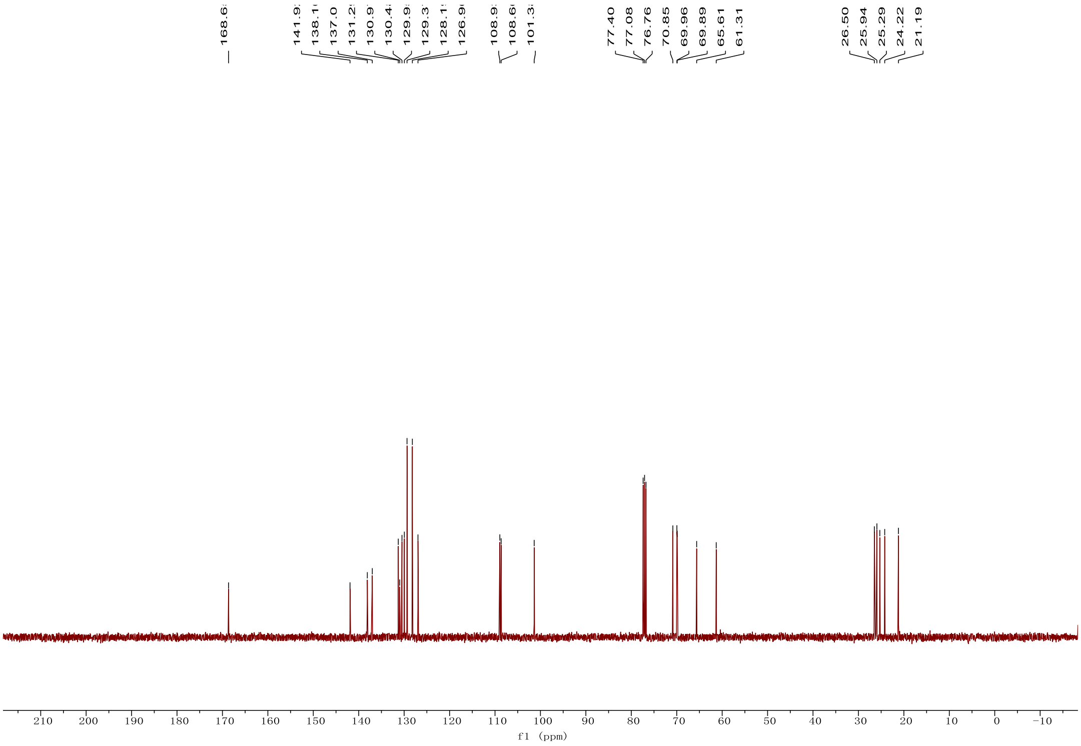

Supplementary Figure 180. ^{13}**C NMR** (101 MHz, Chloroform-*d*) of **34**

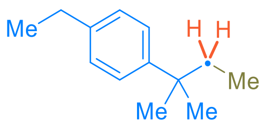

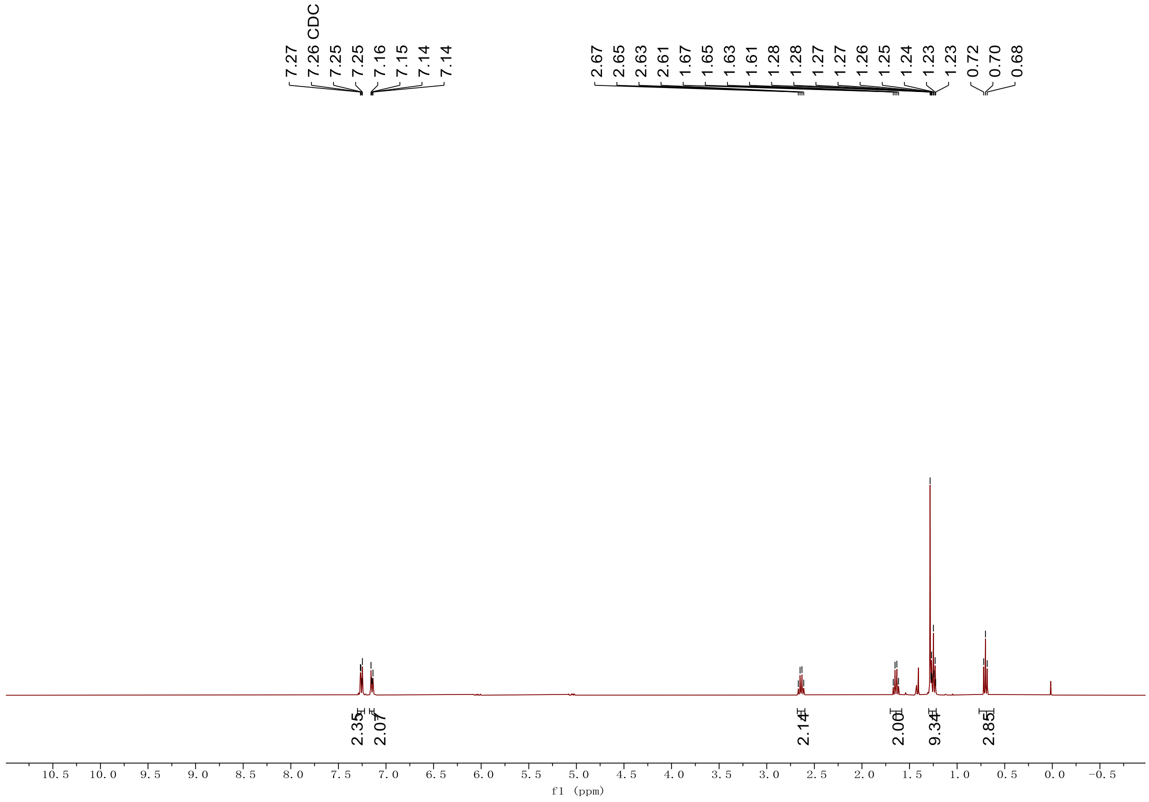

Supplementary Figure 181. ^{1}**H NMR** (400 MHz, Chloroform-*d*) of **37**

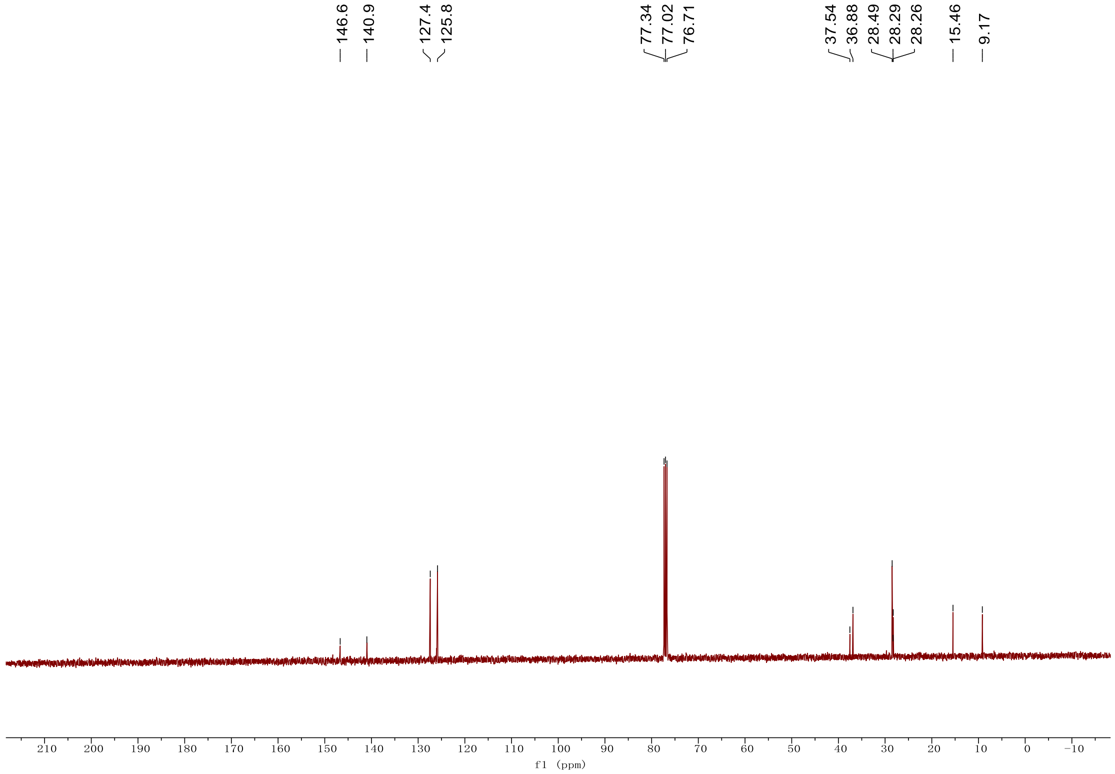

Supplementary Figure 182. ^{13}**C NMR (**101 MHz, Chloroform-*d*) of **37**

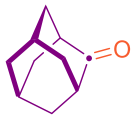

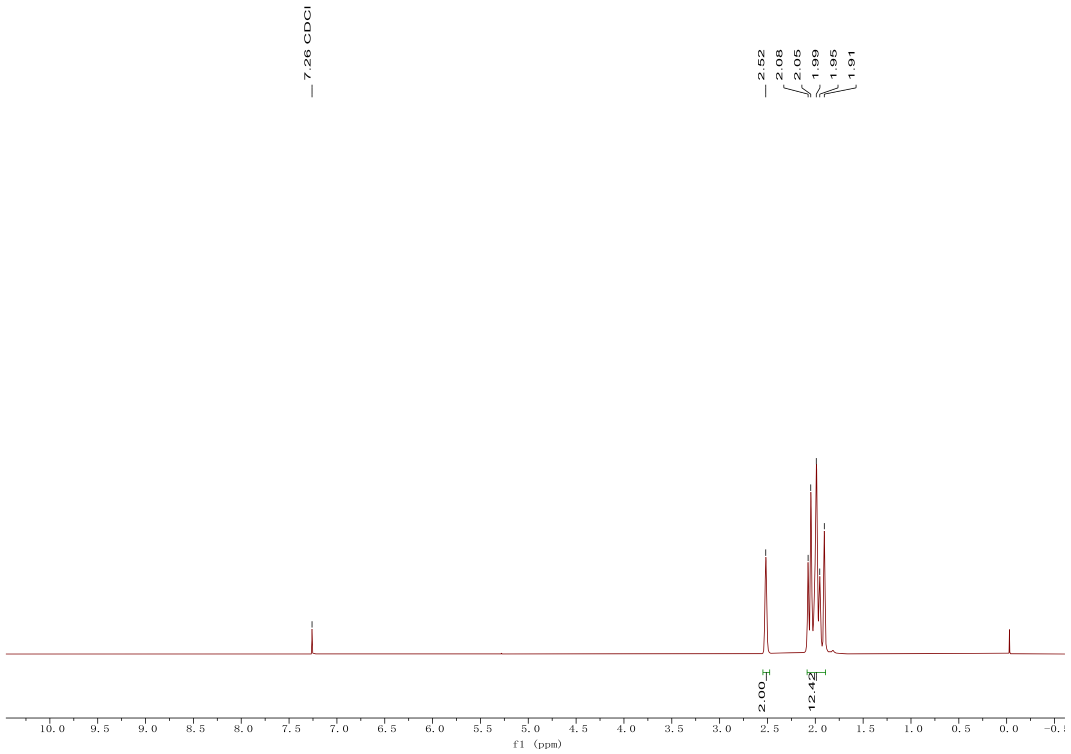

Supplementary Figure 183. ^{1}H NMR (400 MHz, Chloroform-*d*) of **38**

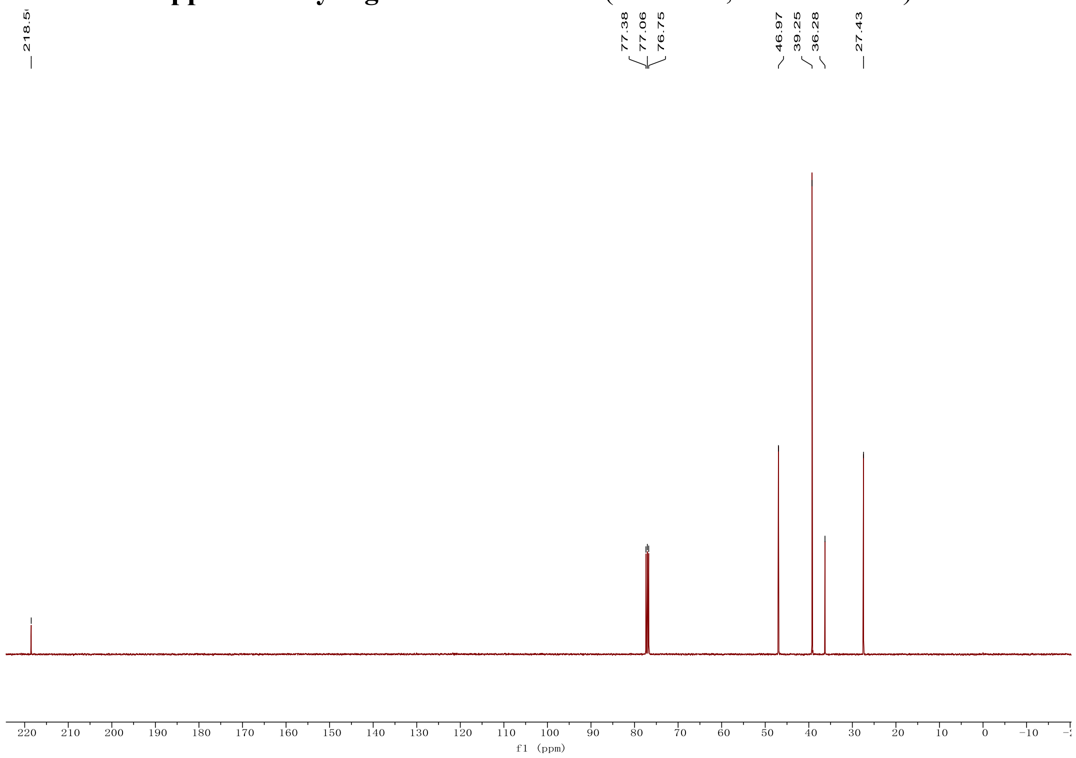

Supplementary Figure 184. ^{13}C NMR (101 MHz, Chloroform-*d*) of **38**

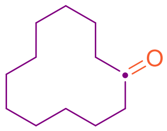

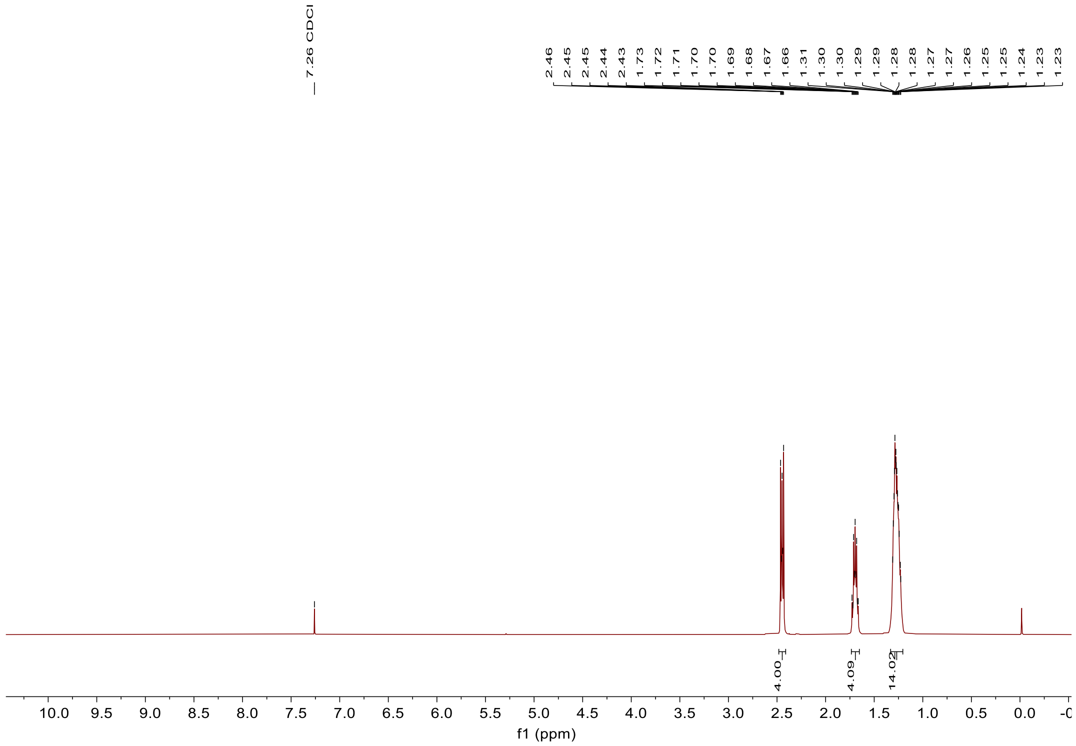

Supplementary Figure 185. ^{1}H NMR (400 MHz, Chloroform-*d*) of **39**

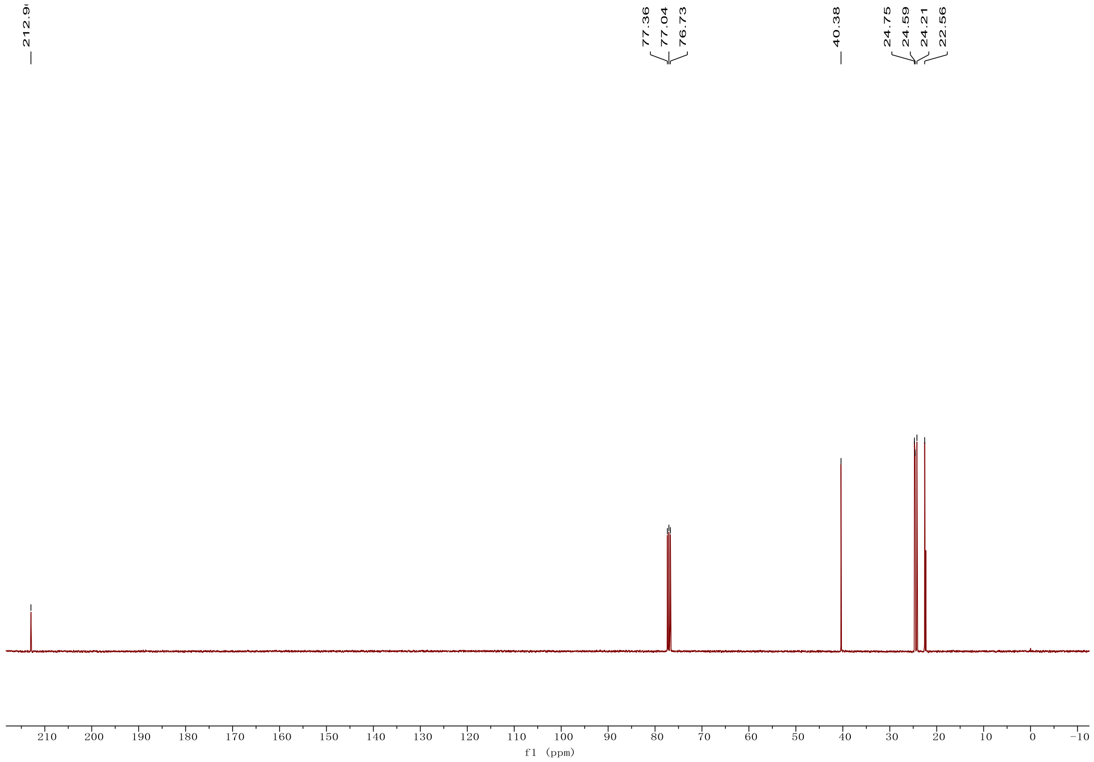

Supplementary Figure 186. ^{13}C NMR (101 MHz, Chloroform-*d*) of **39**

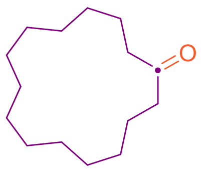

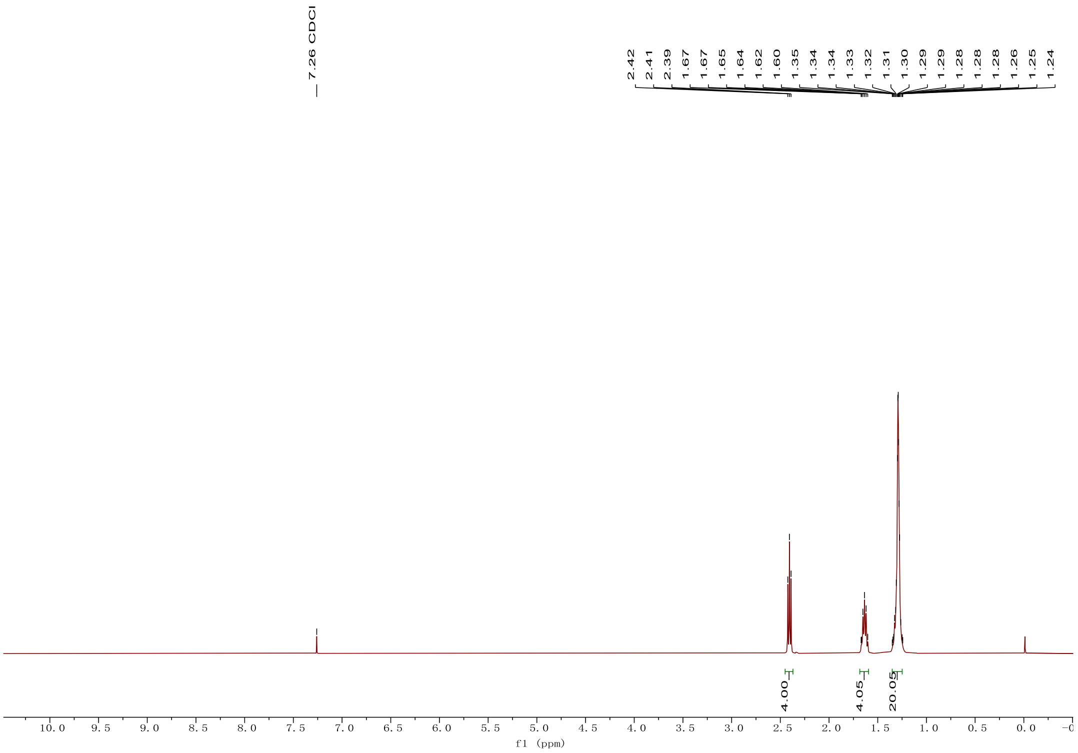

Supplementary Figure 187. ^{1}**H NMR** (400 MHz, Chloroform-*d*) of **40**

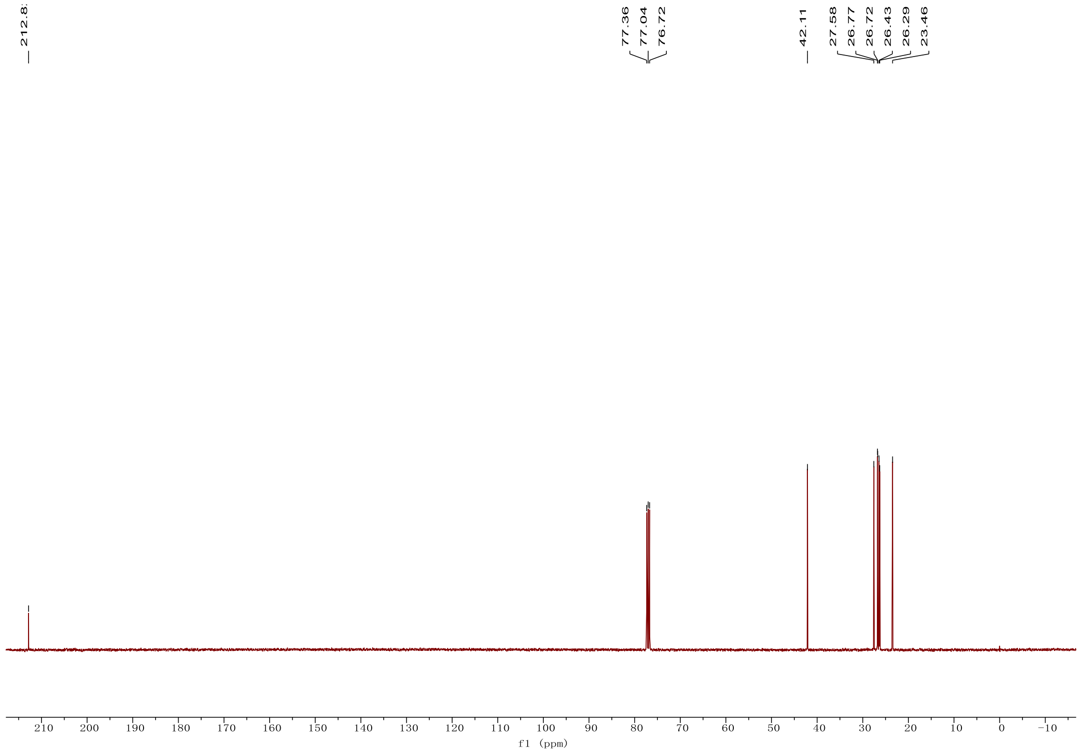

Supplementary Figure 188. ^{13}**C NMR (**101 MHz, Chloroform-*d*) of **40**

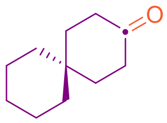

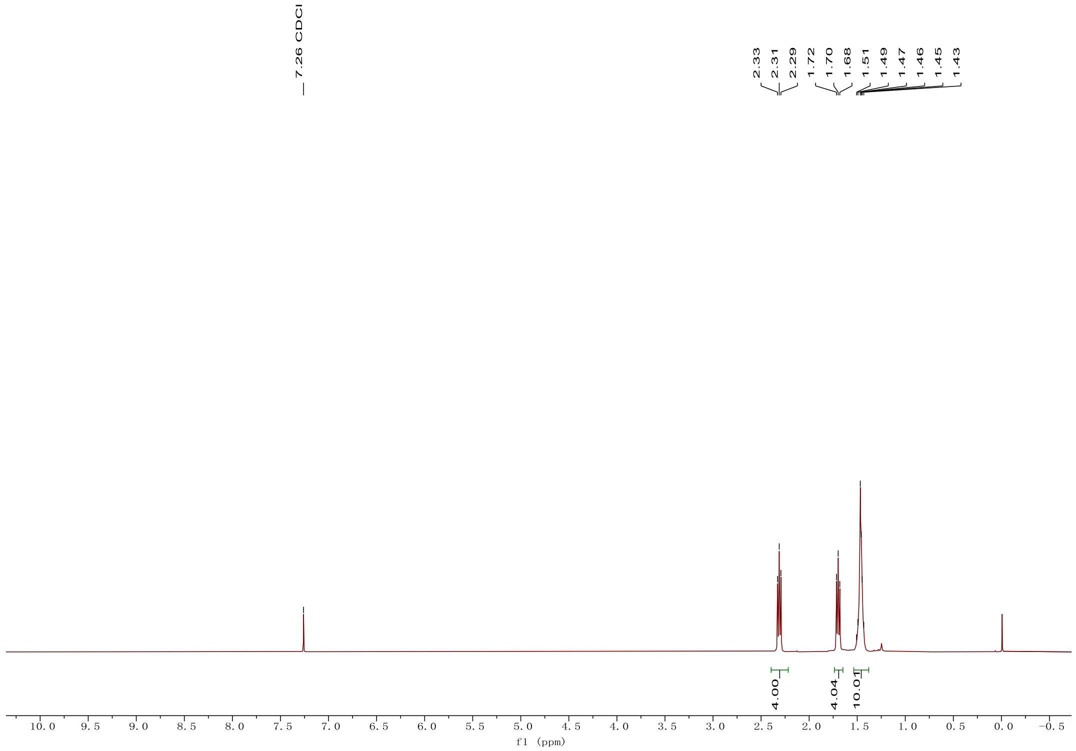

Supplementary Figure 189. ^{1}**H NMR** (400 MHz, Chloroform-*d*) of **41**

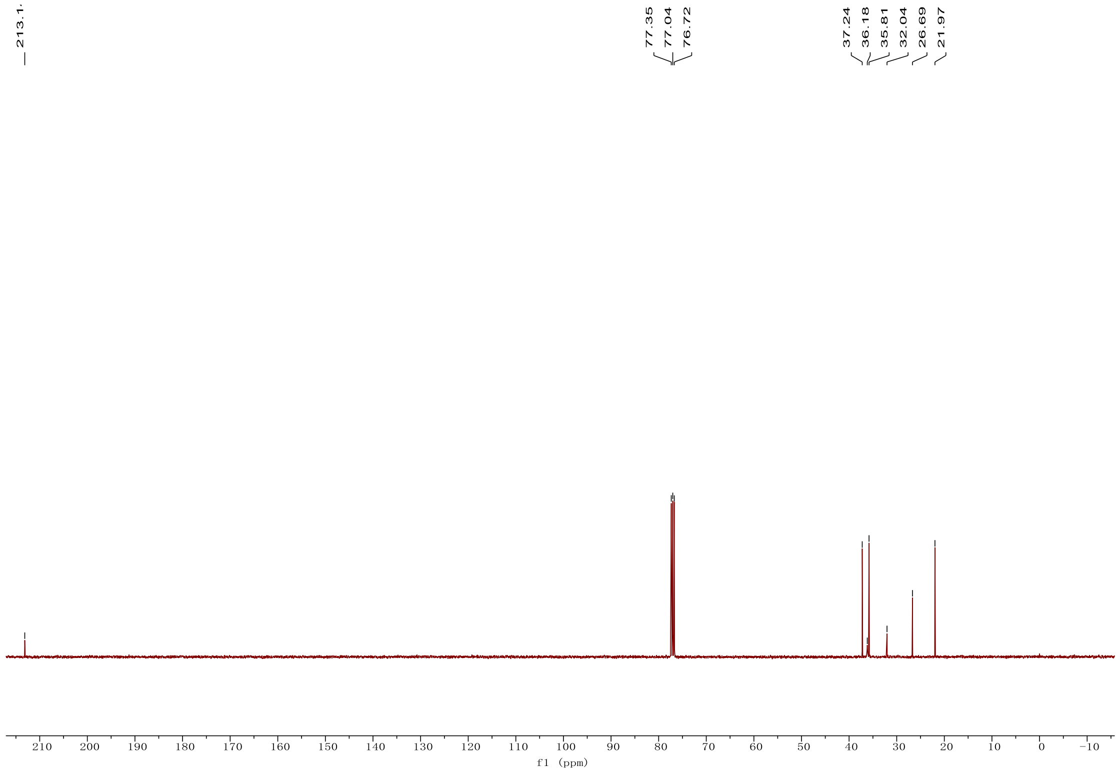

Supplementary Figure 190. ^{13}**C NMR (**101 MHz, Chloroform-*d*) of **41**

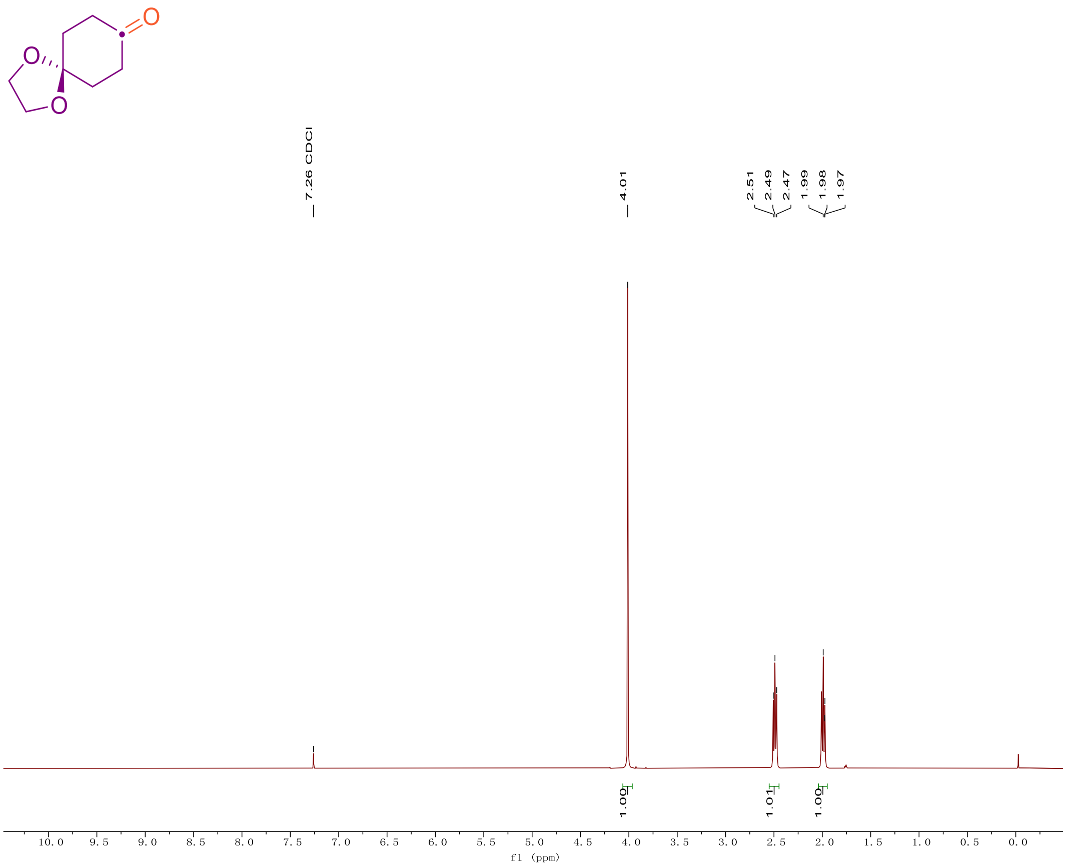

Supplementary Figure 191. ^{1}H NMR (400 MHz, Chloroform-*d*) of **42**

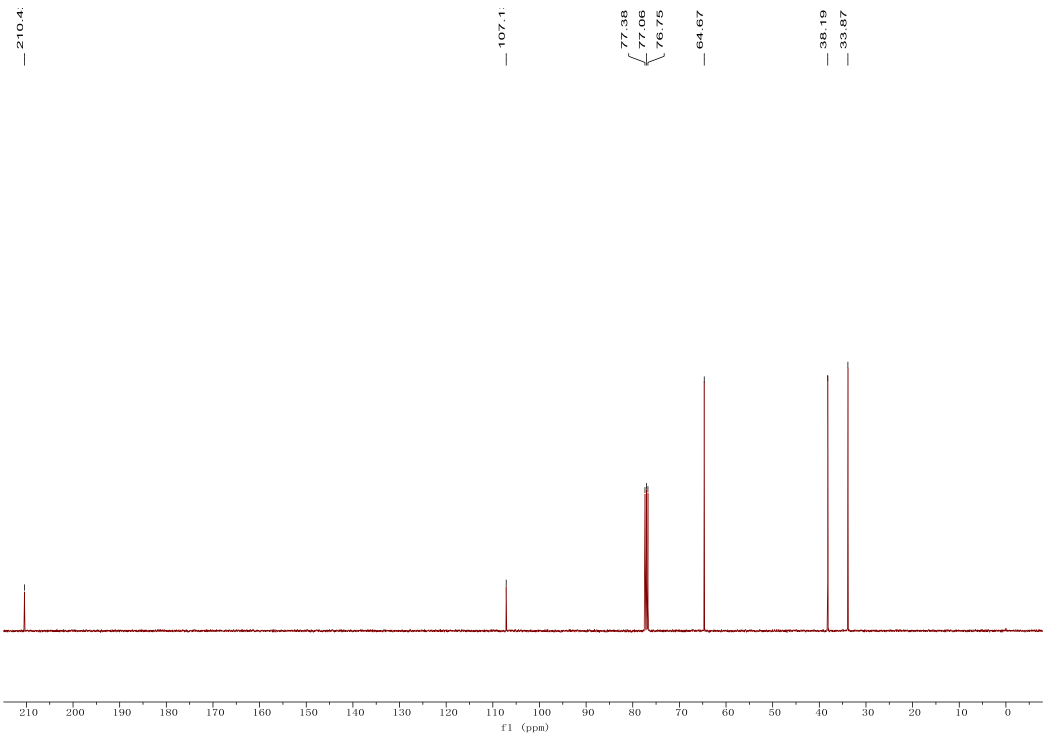

Supplementary Figure 192. ^{13}C NMR (101 MHz, Chloroform-*d*) of **42**

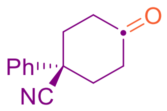

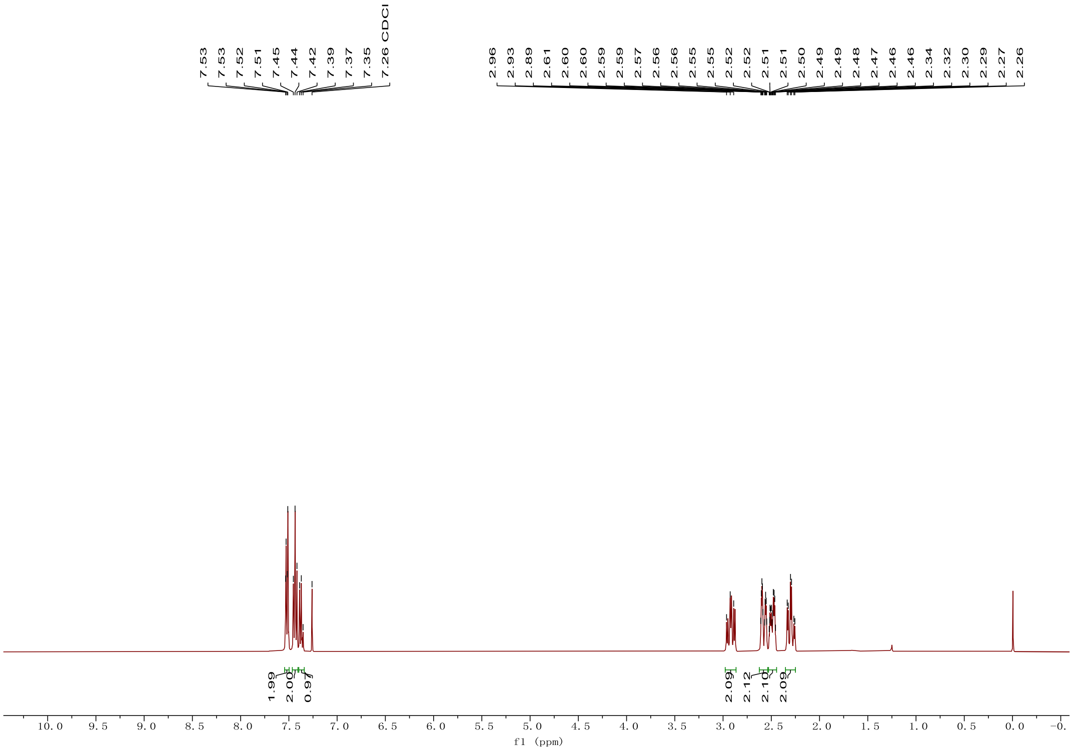

Supplementary Figure 193. ^{1}**H NMR** (400 MHz, Chloroform-*d*) of **43**

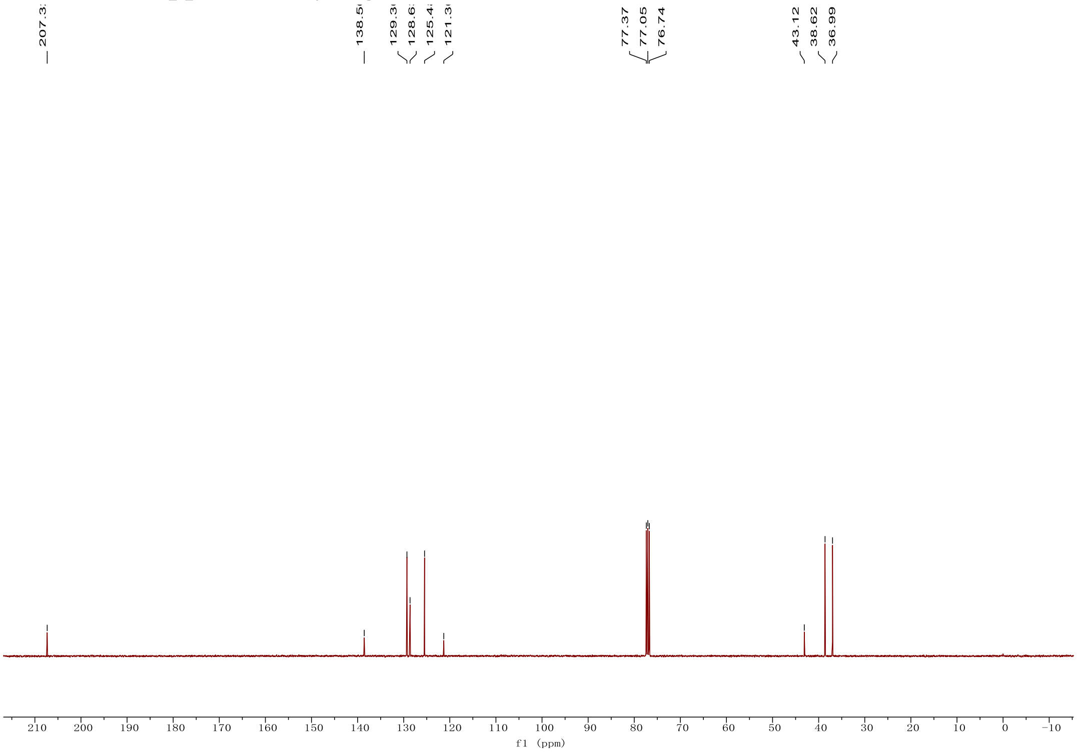

Supplementary Figure 194. ^{13}**C NMR (**101 MHz, Chloroform-*d*) of **43**

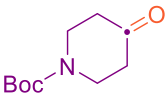

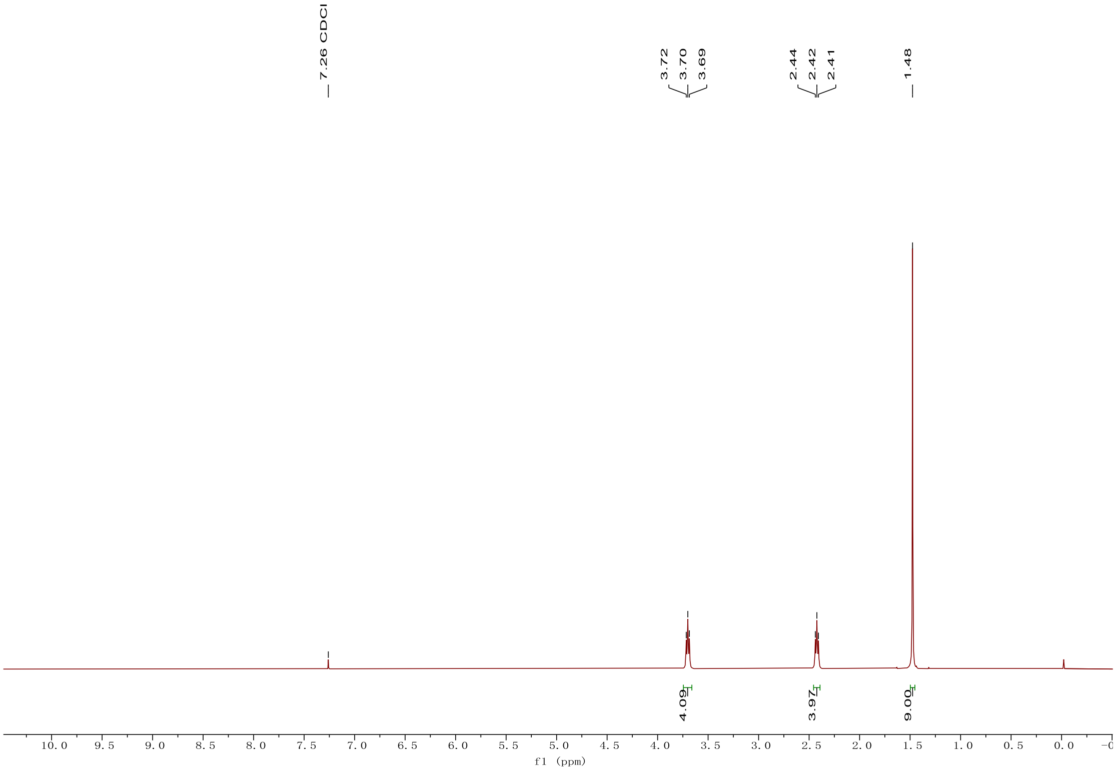

Supplementary Figure 195. ^{1}H NMR (400 MHz, Chloroform-*d*) of **44**

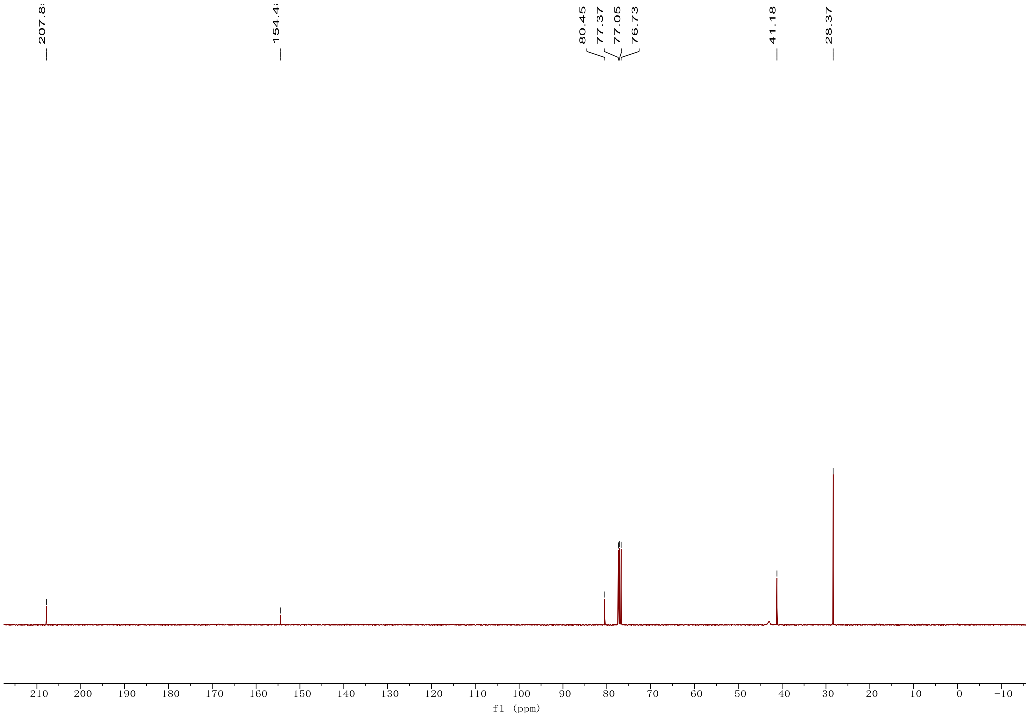

Supplementary Figure 196. ^{13}C NMR (101 MHz, Chloroform-*d*) of **44**

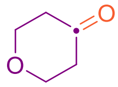

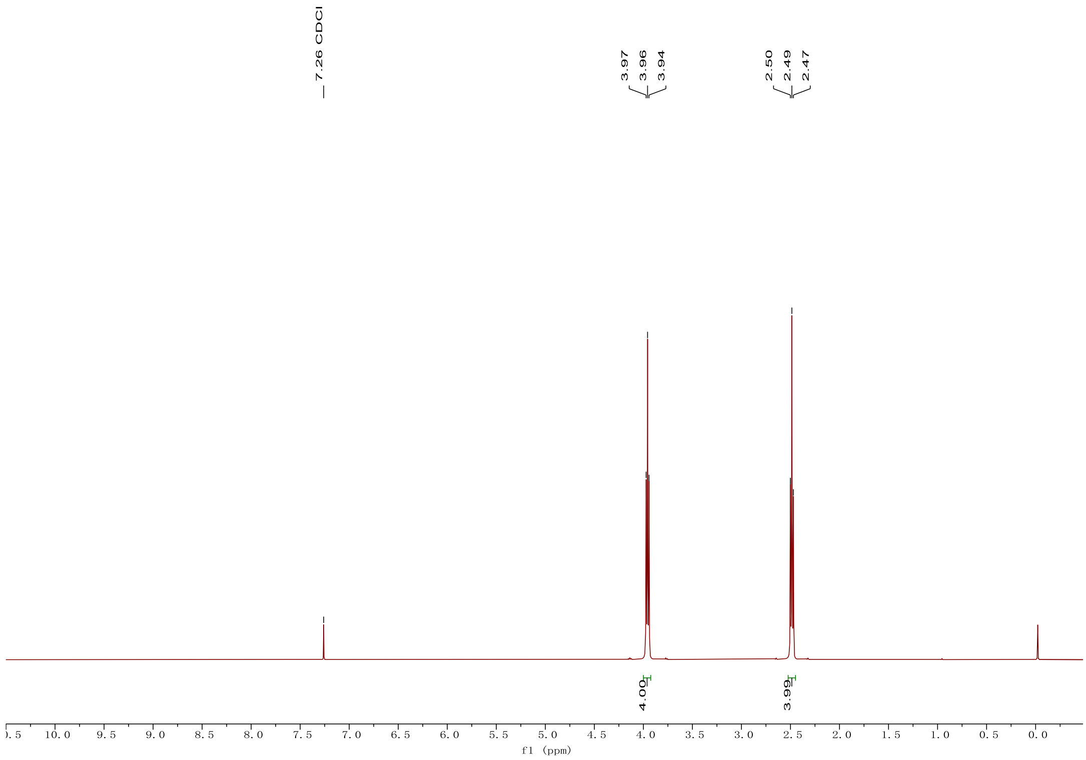

Supplementary Figure 197. ^{1}**H NMR** (400 MHz, Chloroform-*d*) of **45**

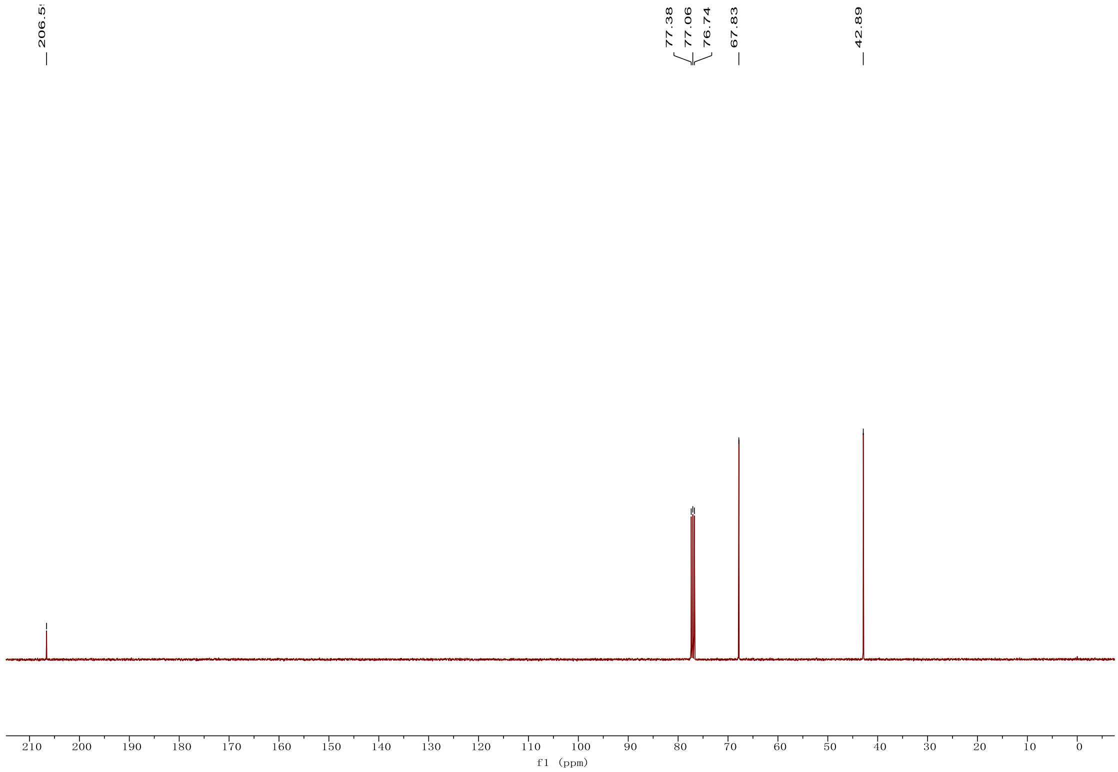

Supplementary Figure 198. ^{13}**C NMR (**101 MHz, Chloroform-*d*) of **45**

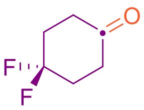

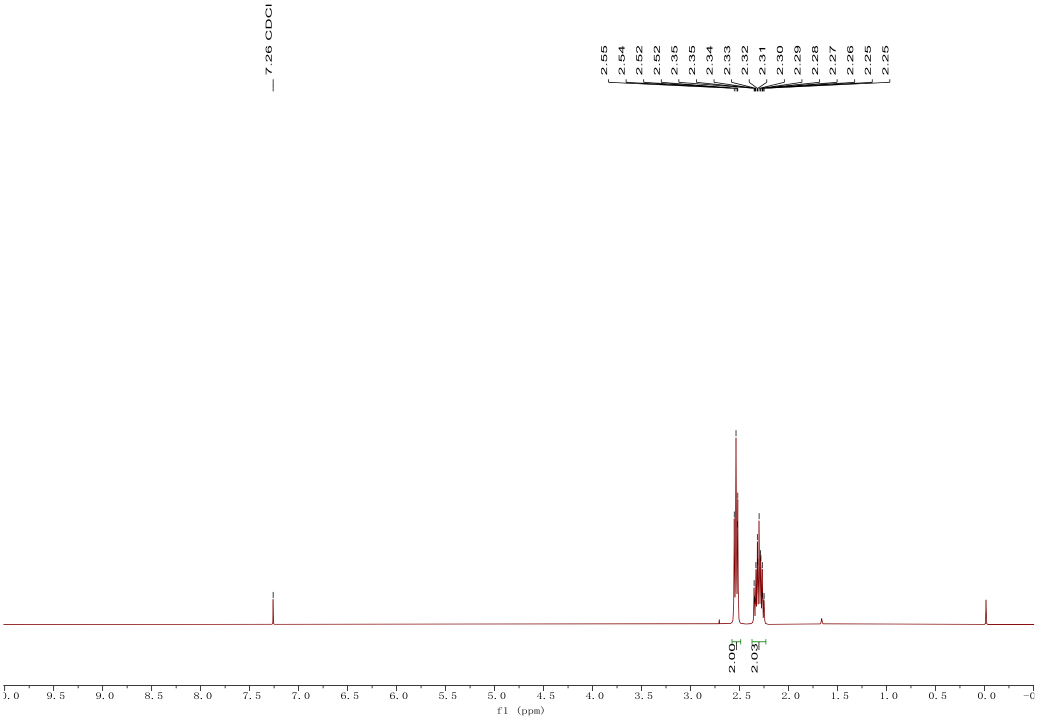

Supplementary Figure 199. ^{1}H NMR (400 MHz, Chloroform-*d*) of **46**

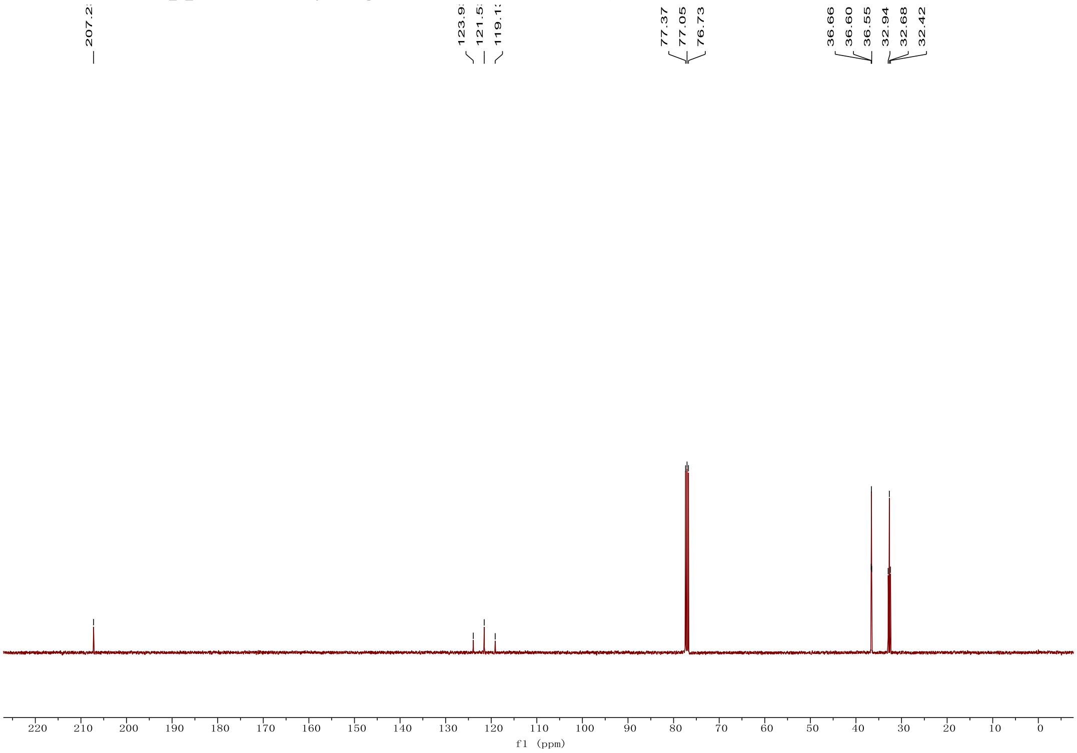

Supplementary Figure 200. ^{13}C NMR (101 MHz, Chloroform-*d*) of **46**

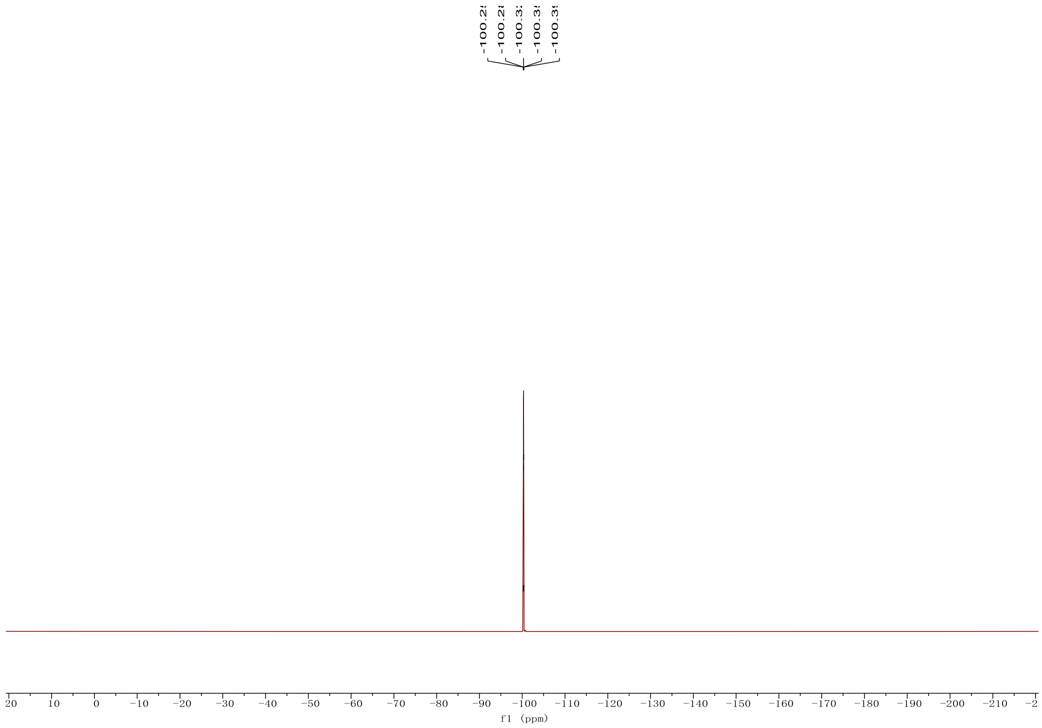

Supplementary Figure 201. ^{19}F NMR (376 MHz, Chloroform-*d*) of **46**

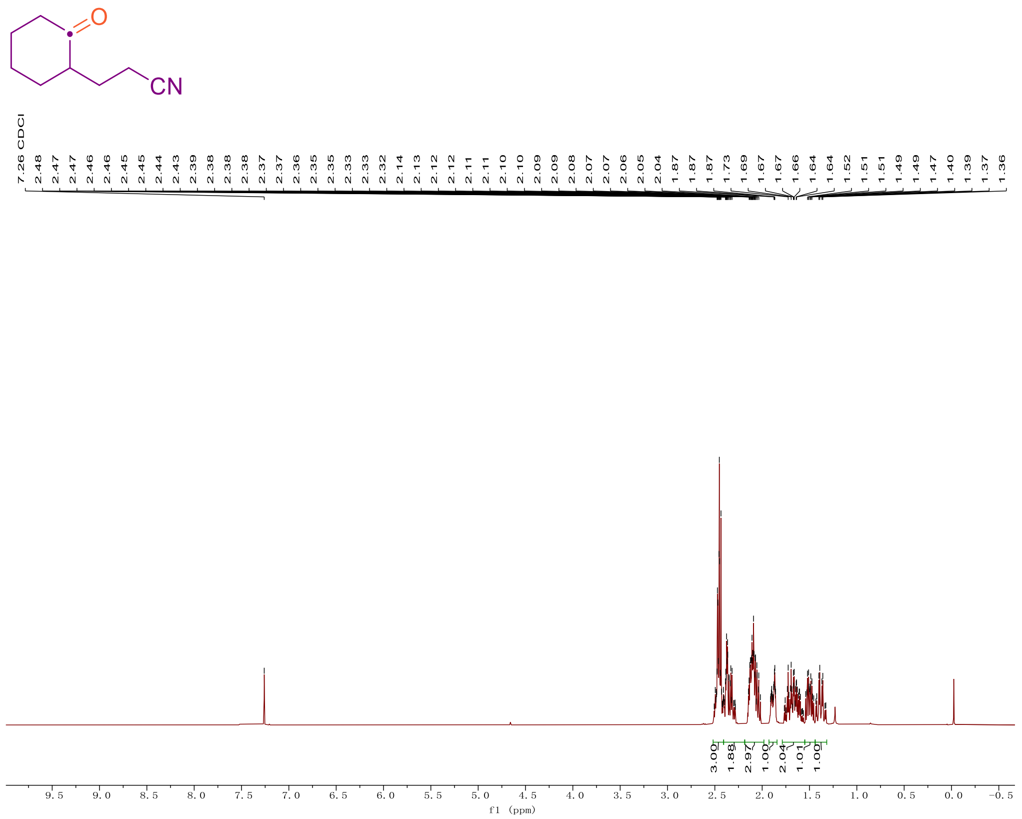

Supplementary Figure 202. ^{1}**H NMR** (400 MHz, Chloroform-*d*) of **47**

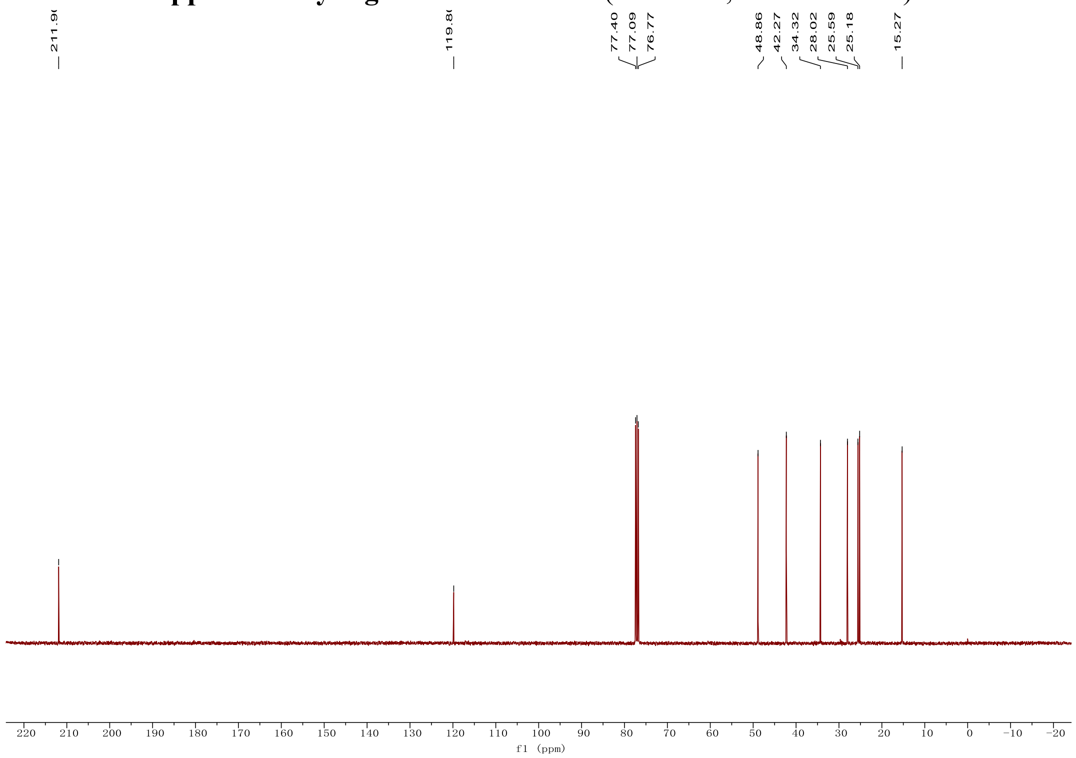

Supplementary Figure 203. ^{13}**C NMR (**101 MHz, Chloroform-*d*) of **47**

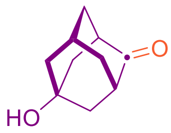

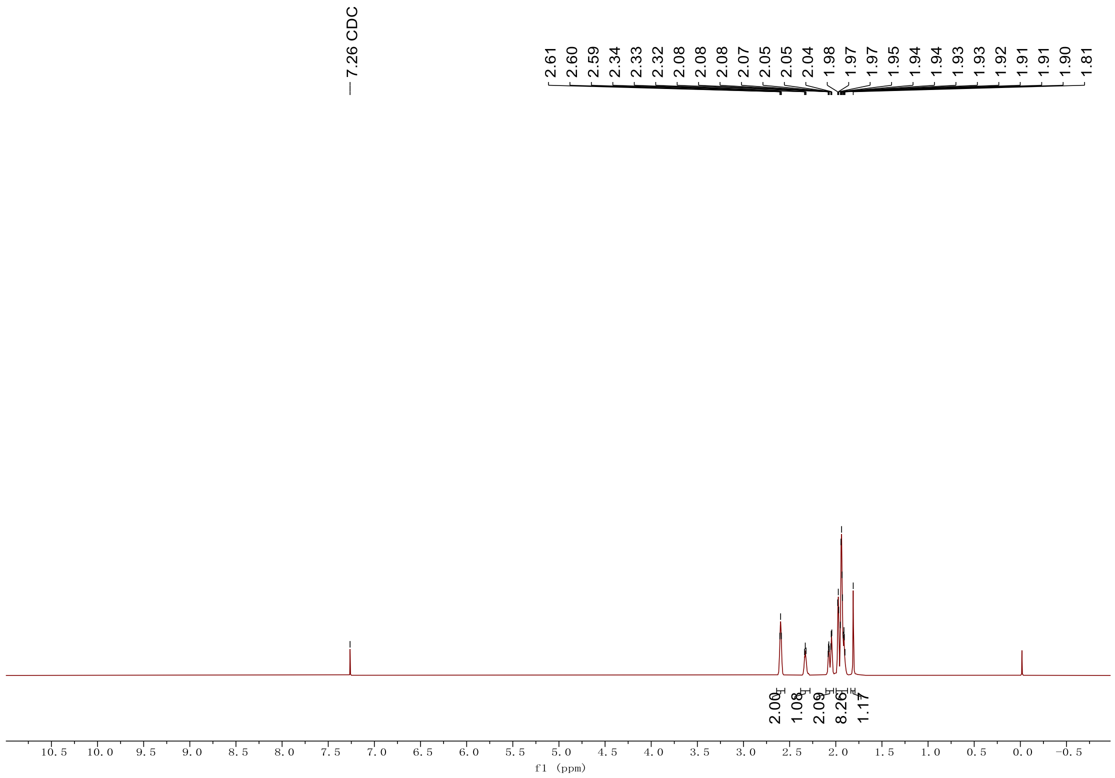

Supplementary Figure 204. ^{1}H NMR (400 MHz, Chloroform-*d*) of **48**

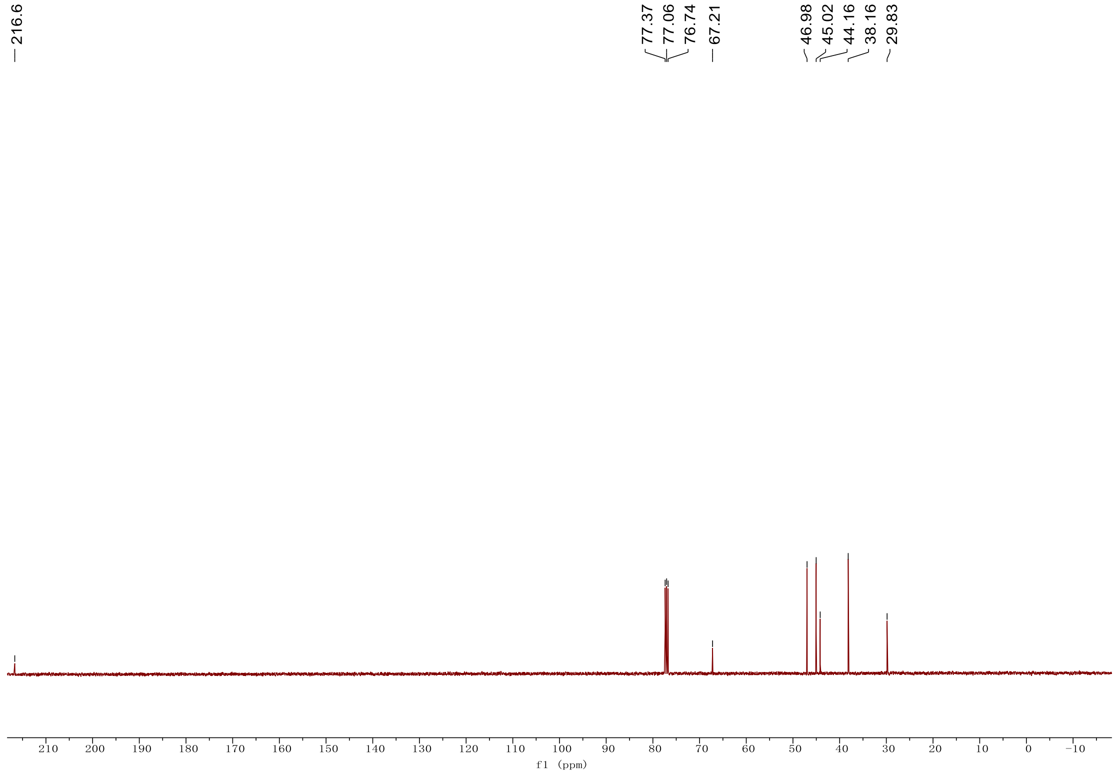

Supplementary Figure 205. ^{13}C NMR (101 MHz, Chloroform-*d*) of **48**

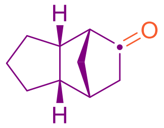

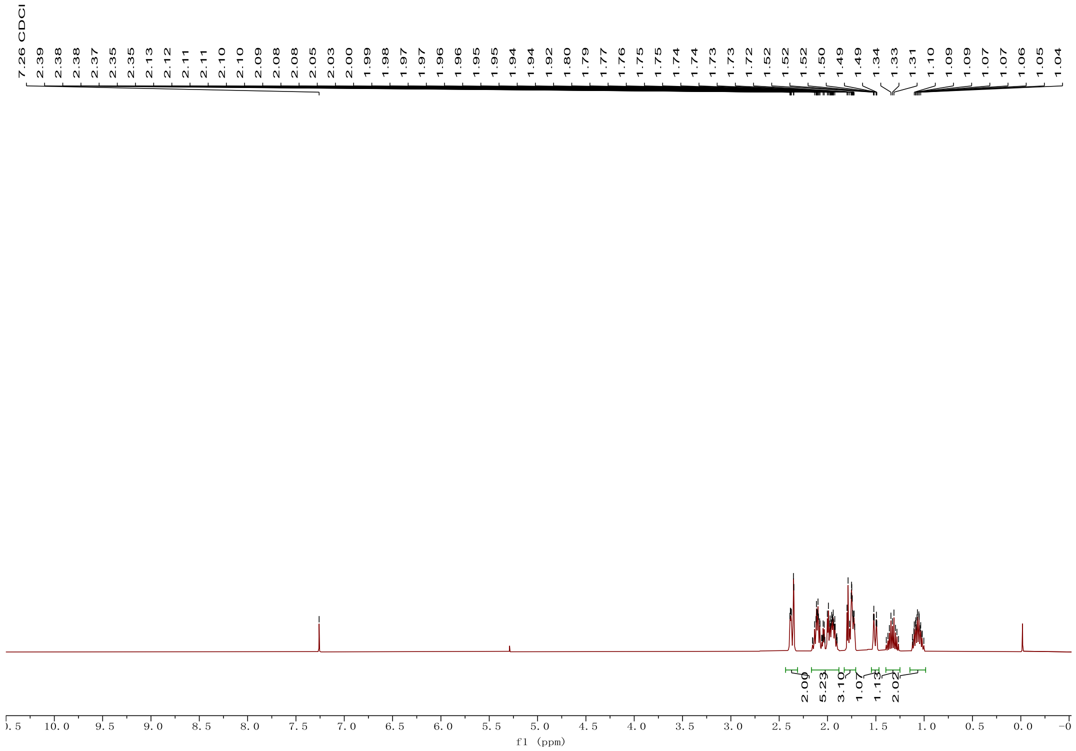

Supplementary Figure 206. ^{1}H NMR (400 MHz, Chloroform-*d*) of **49**

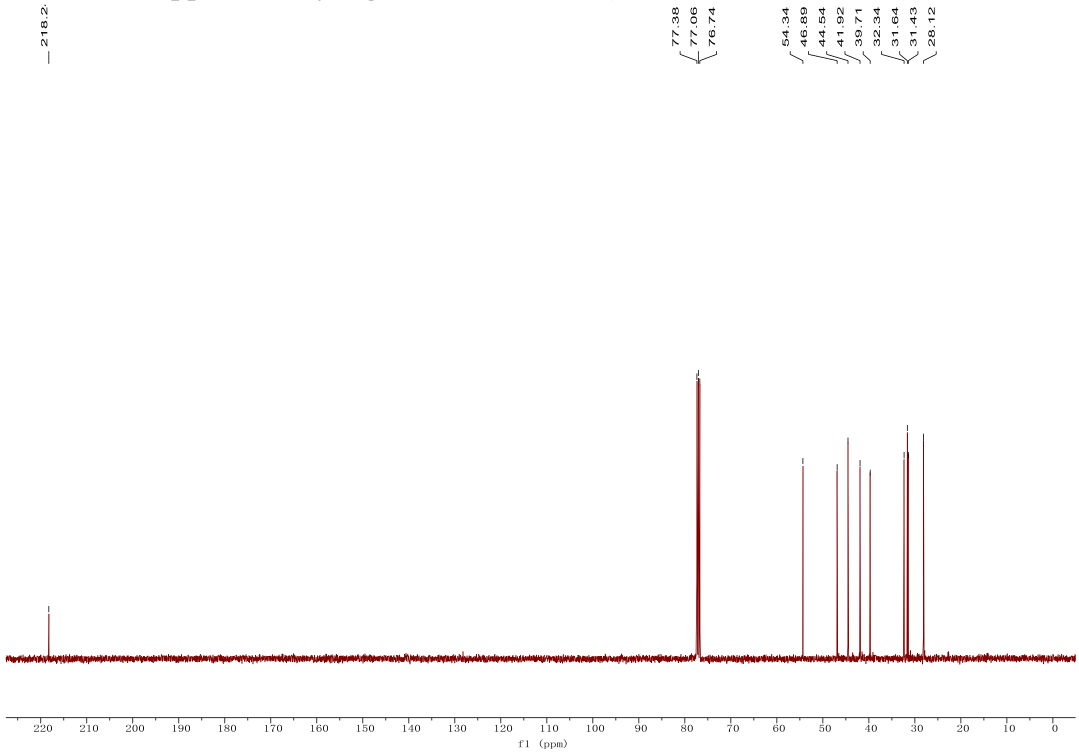

Supplementary Figure 207. ^{13}C NMR (101 MHz, Chloroform-*d*) of **37**

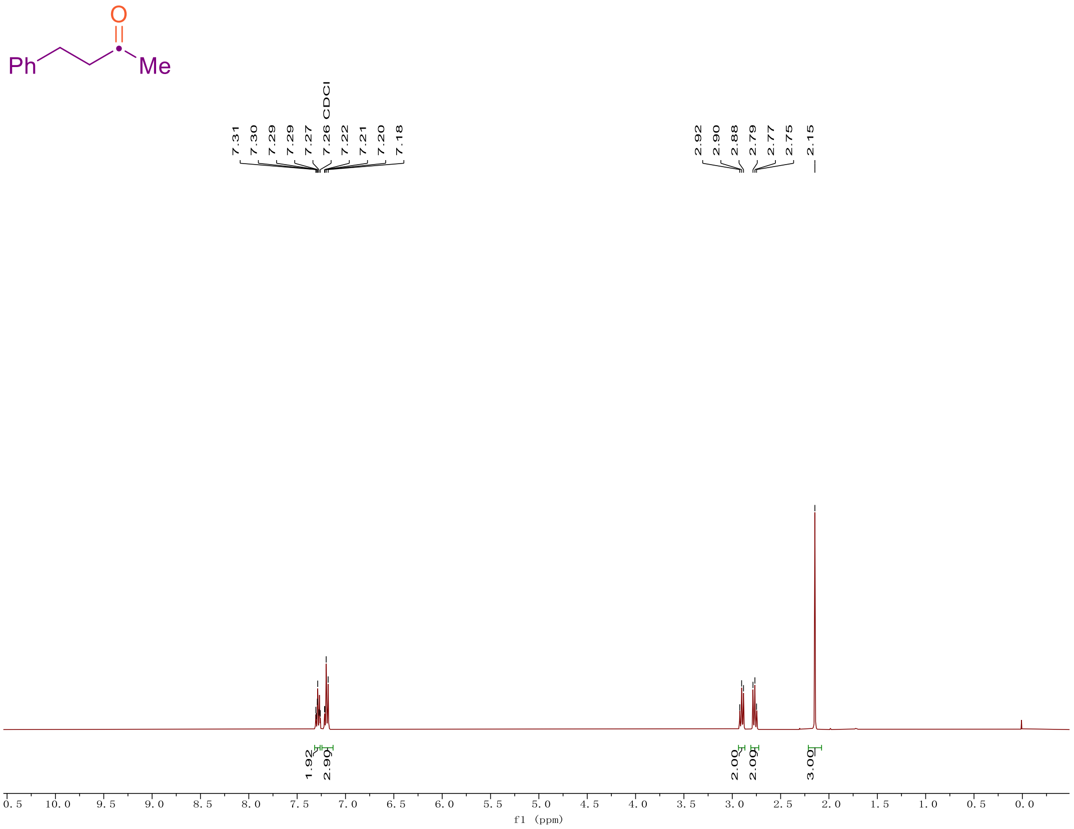

Supplementary Figure 208. ^{1}H NMR (400 MHz, Chloroform-*d*) of **50**

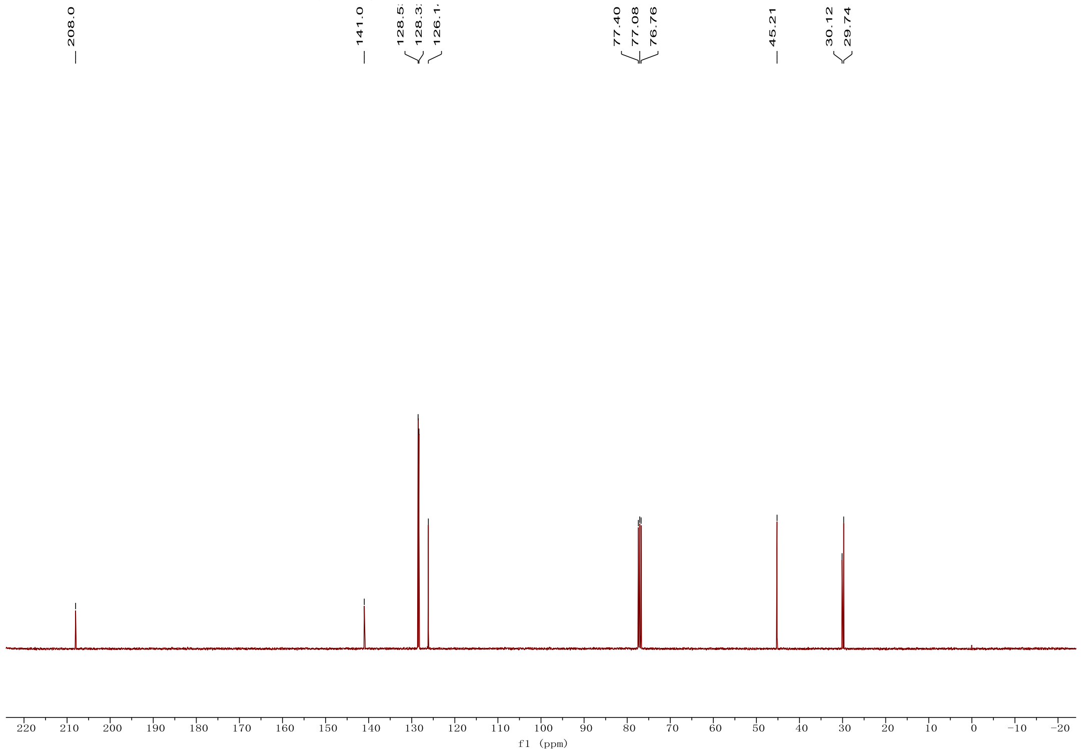

Supplementary Figure 209. ^{13}C NMR (101 MHz, Chloroform-*d*) of **50**

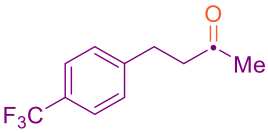

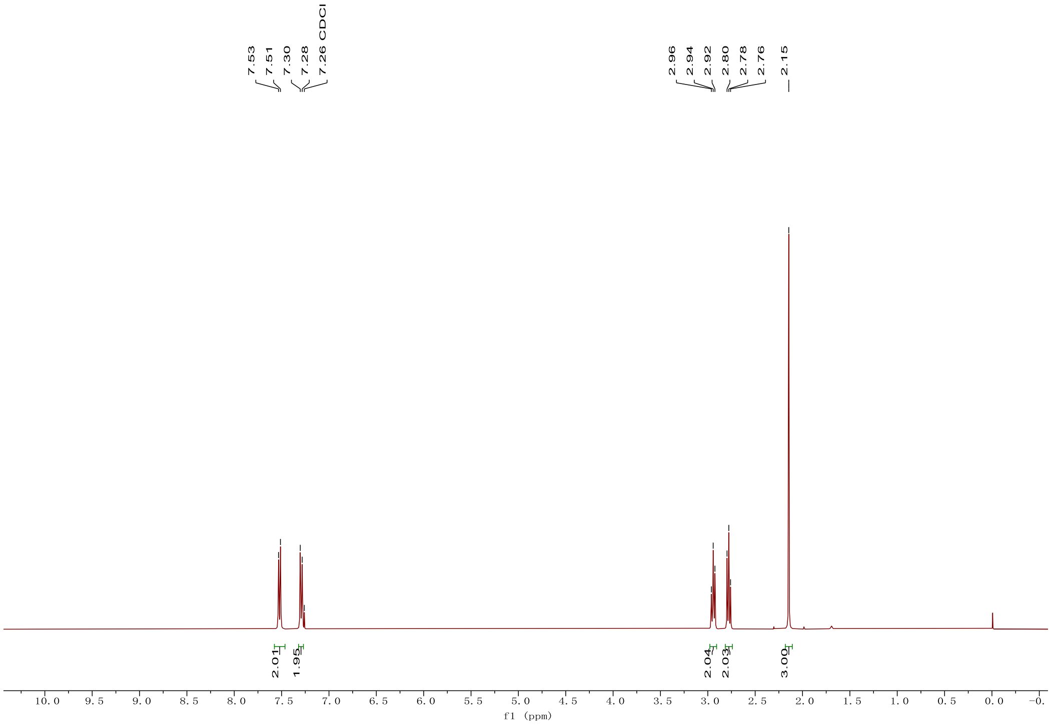

Supplementary Figure 210. **^{1}H NMR** (400 MHz, Chloroform-*d*) of **51**

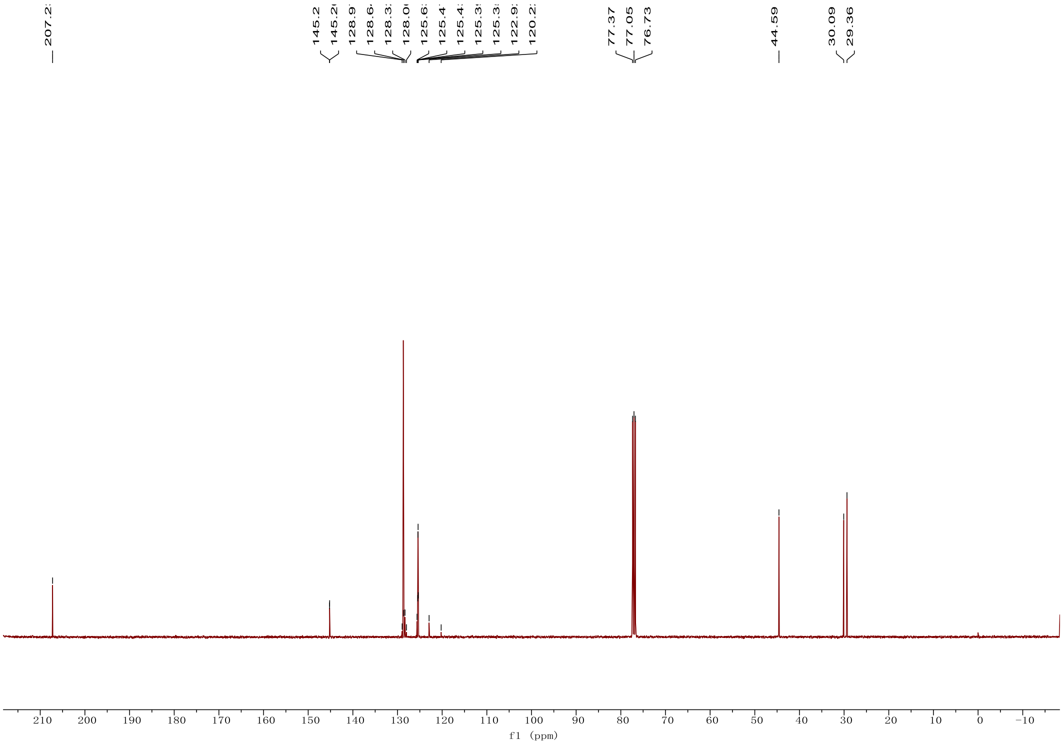

Supplementary Figure 211. ^{13}C NMR (101 MHz, Chloroform-*d*) of **51**

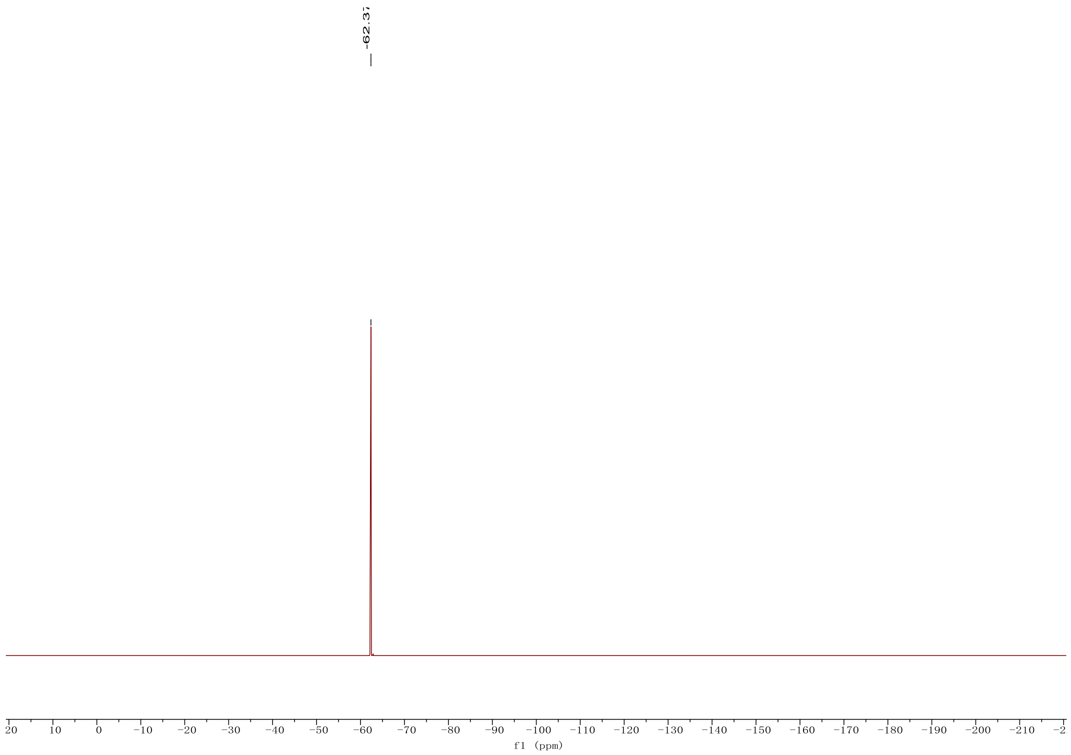

Supplementary Figure 212. ^{19}**F NMR (**376 MHz, Chloroform-*d*) of **51**

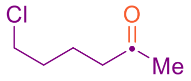

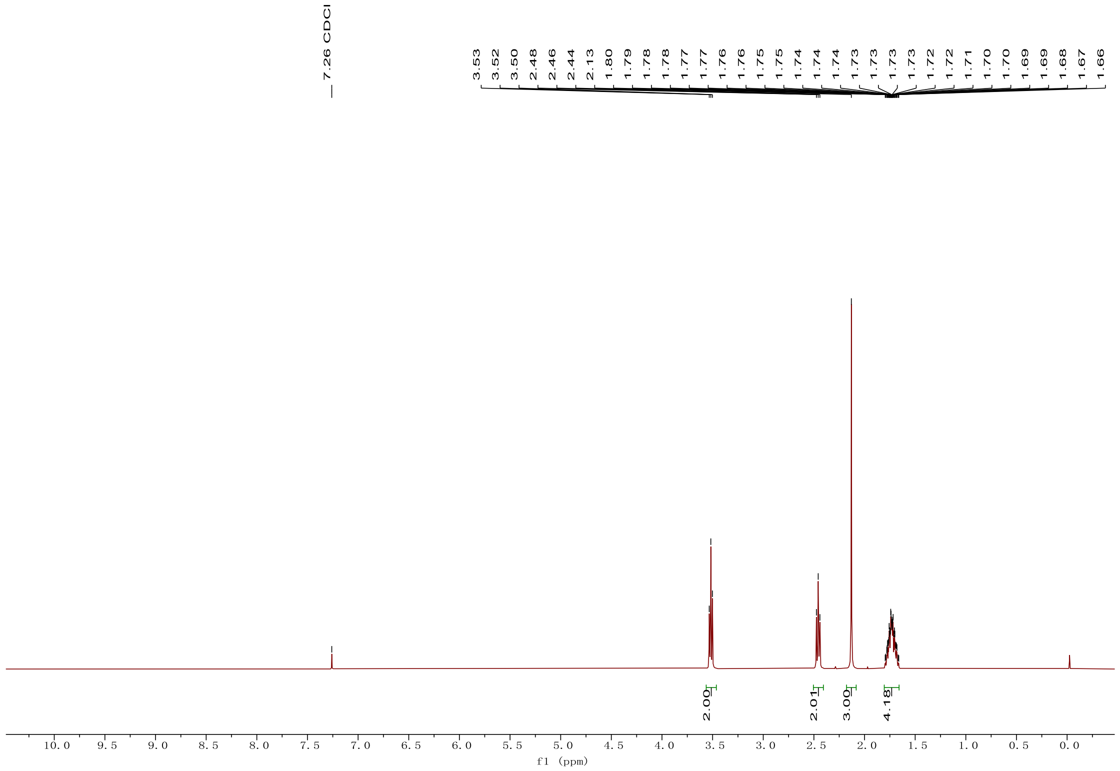

Supplementary Figure 213. ^{1}H NMR (400 MHz, Chloroform-*d*) of **52**

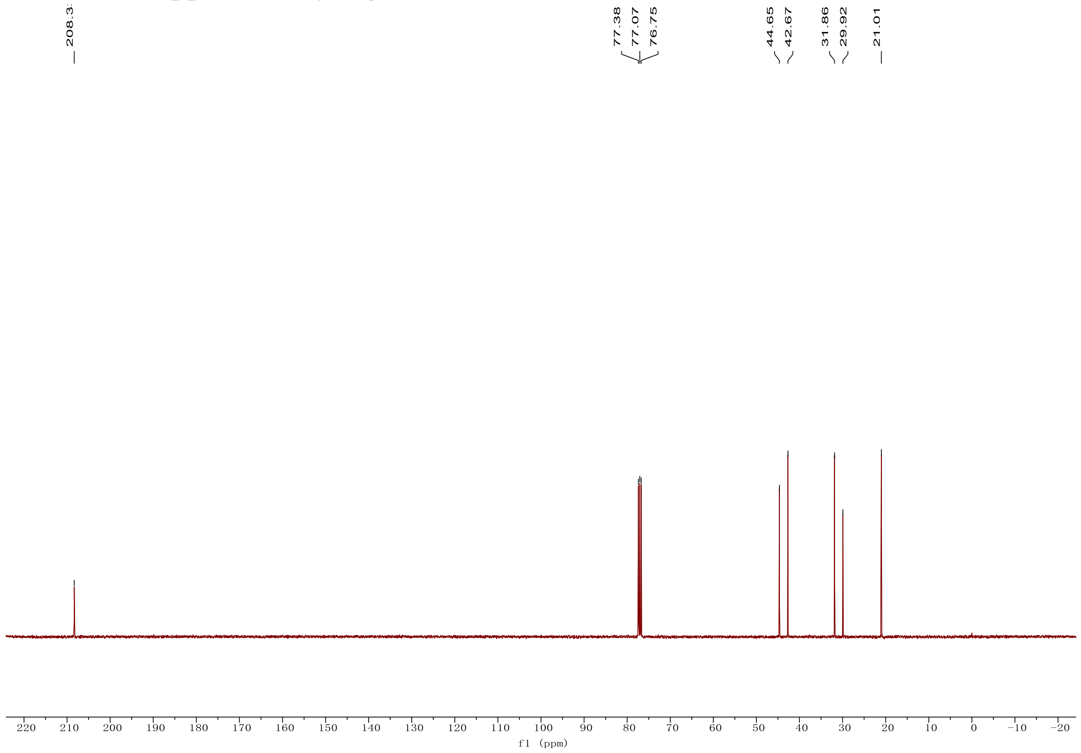

Supplementary Figure 214. ^{13}C NMR (101 MHz, Chloroform-*d*) of **52**

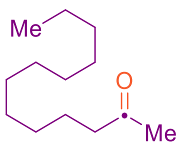

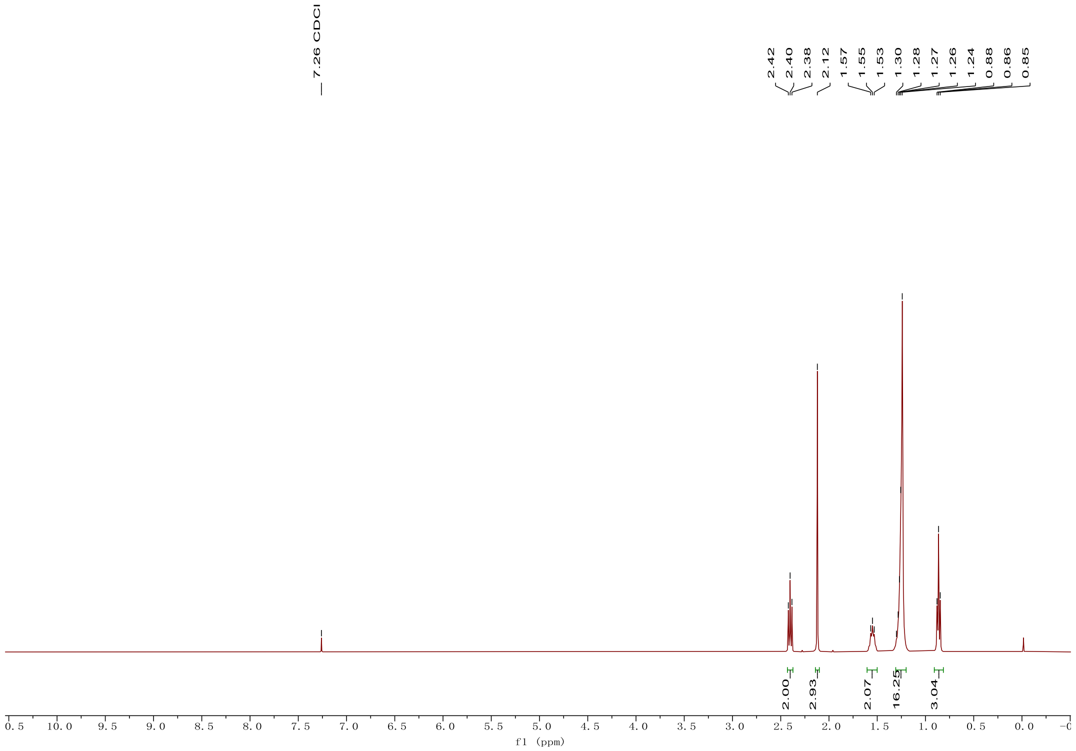

Supplementary Figure 215. ^{1}**H NMR** (400 MHz, Chloroform-*d*) of **53**

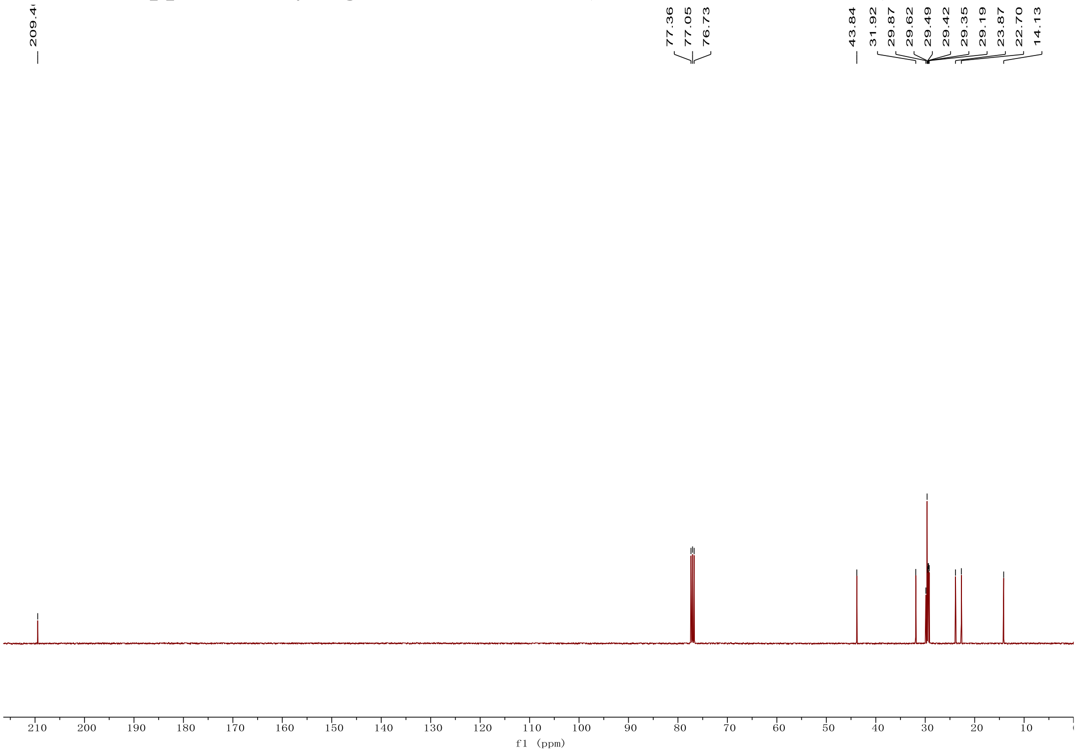

Supplementary Figure 216. ^{13}**C NMR (**101 MHz, Chloroform-*d*) of **53**

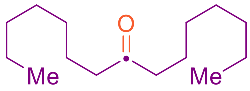

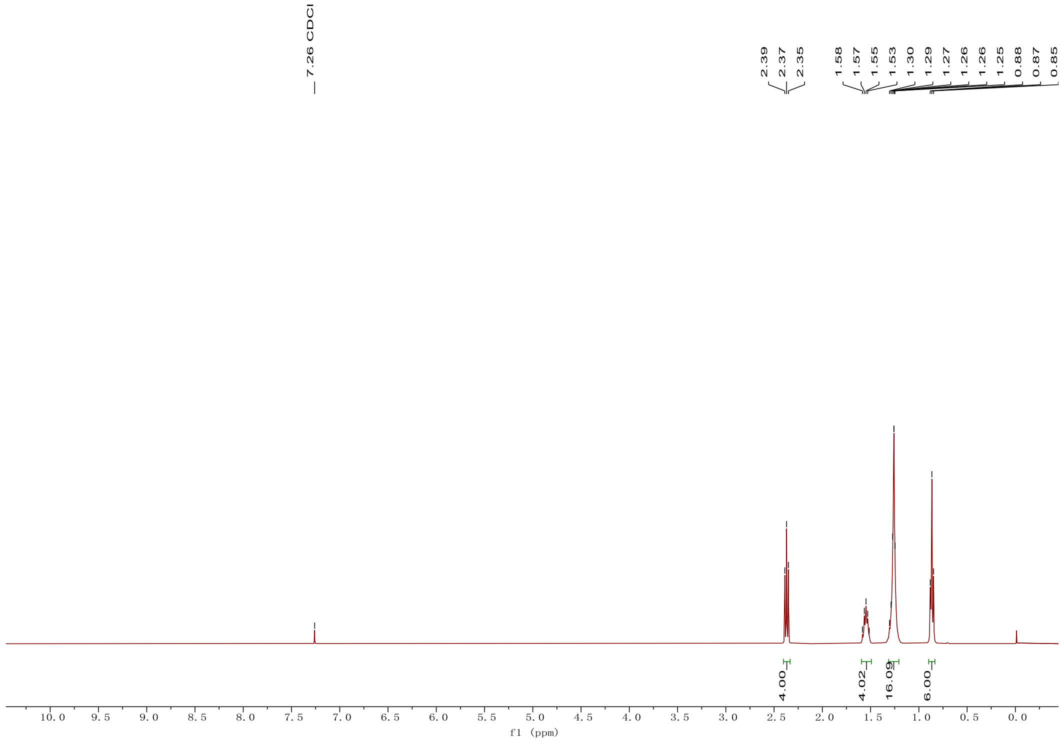

Supplementary Figure 217. **^{1}H NMR** (400 MHz, Chloroform-*d*) of **54**

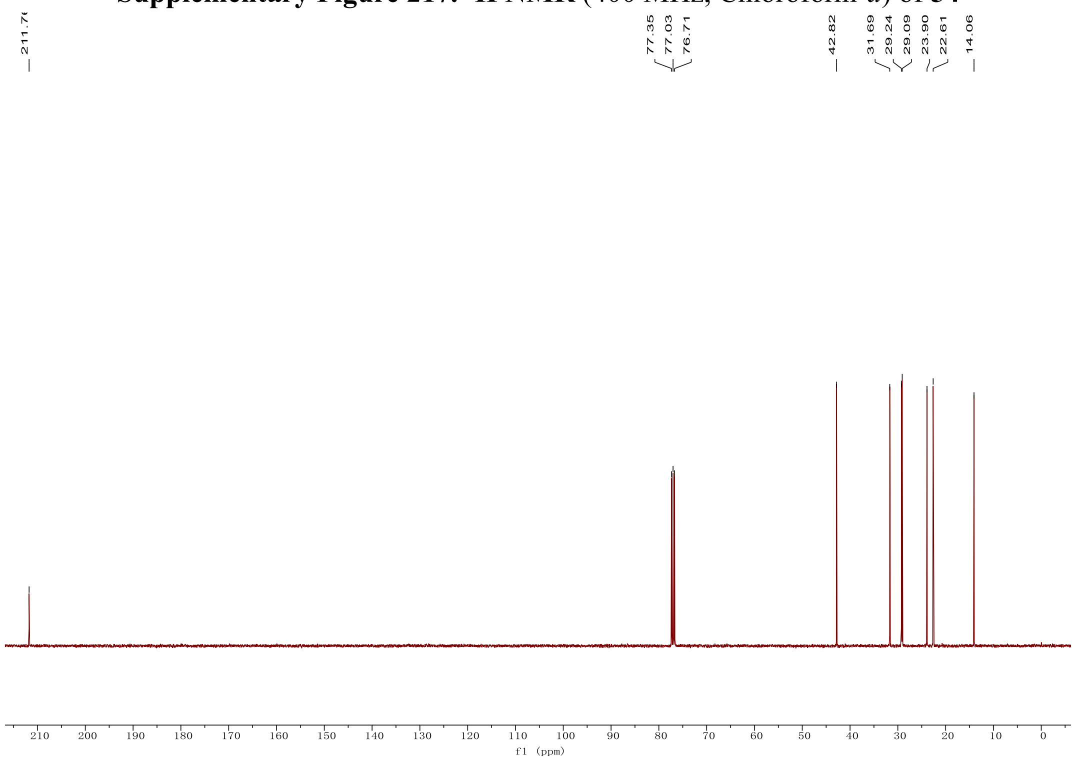

Supplementary Figure 218. **^{13}C NMR** (101 MHz, Chloroform-*d*) of **54**

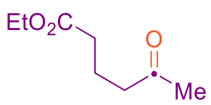

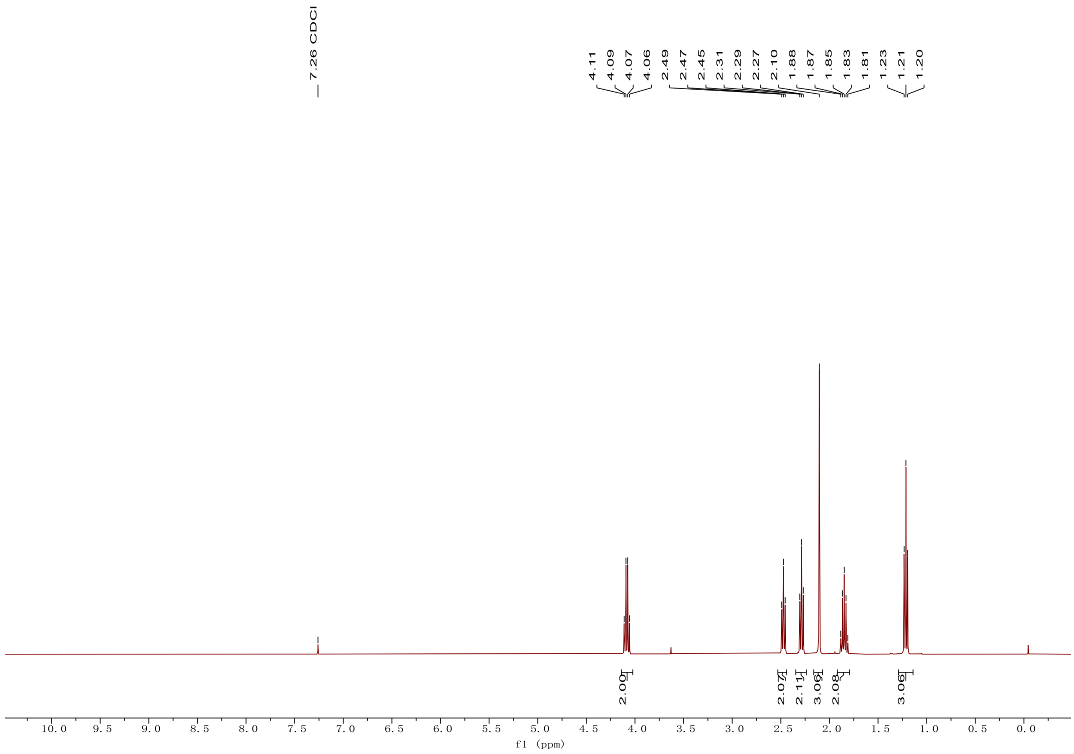

Supplementary Figure 219. ^{1}H NMR (400 MHz, Chloroform-*d*) of **55**

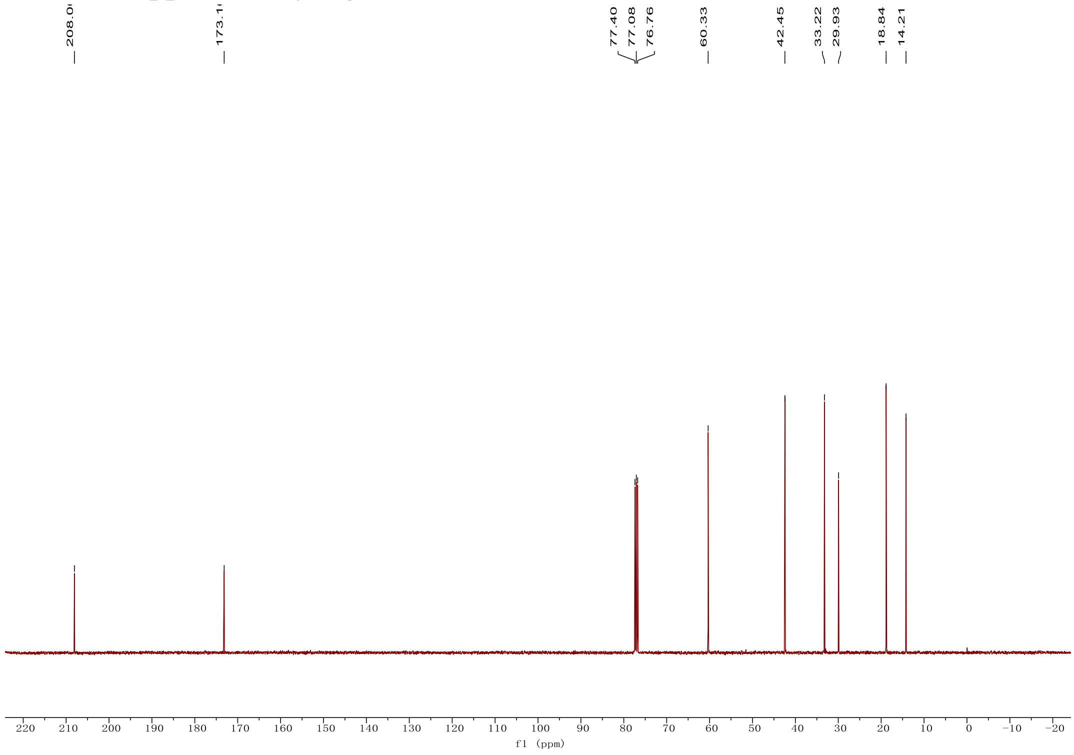

Supplementary Figure 220. ^{13}C NMR (101 MHz, Chloroform-*d*) of **55**

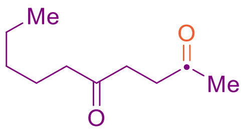

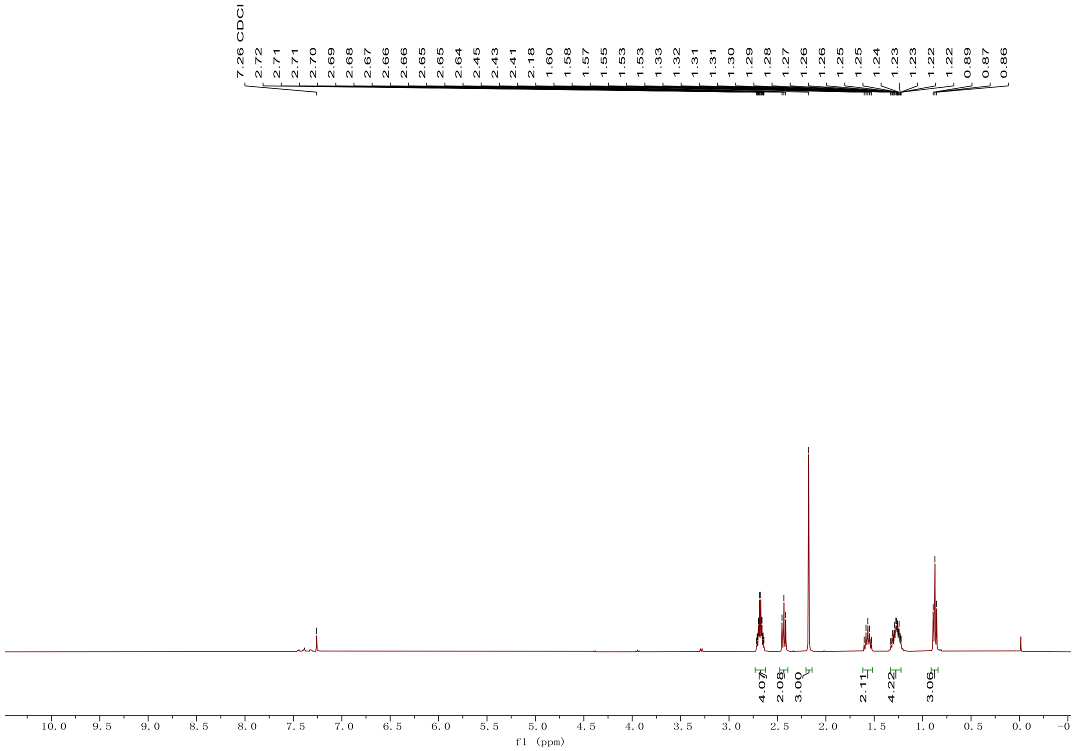

Supplementary Figure 221. ^{1}H NMR (400 MHz, Chloroform-*d*) of **56**

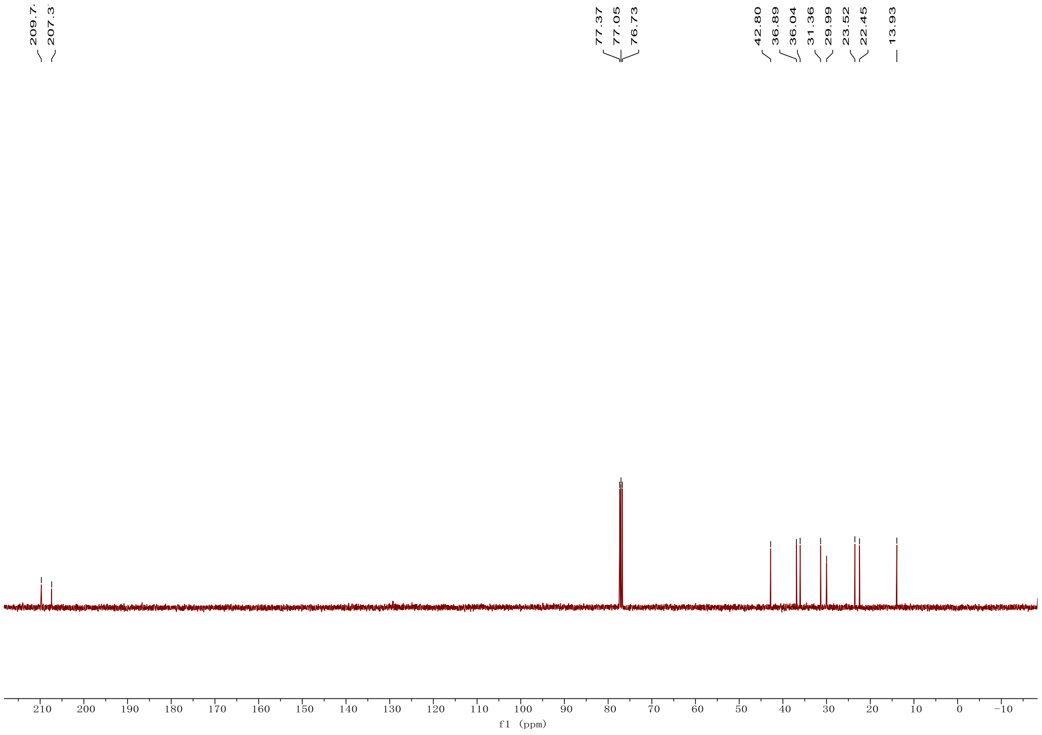

Supplementary Figure 222. ^{13}C NMR (101 MHz, Chloroform-*d*) of **56**

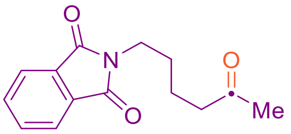

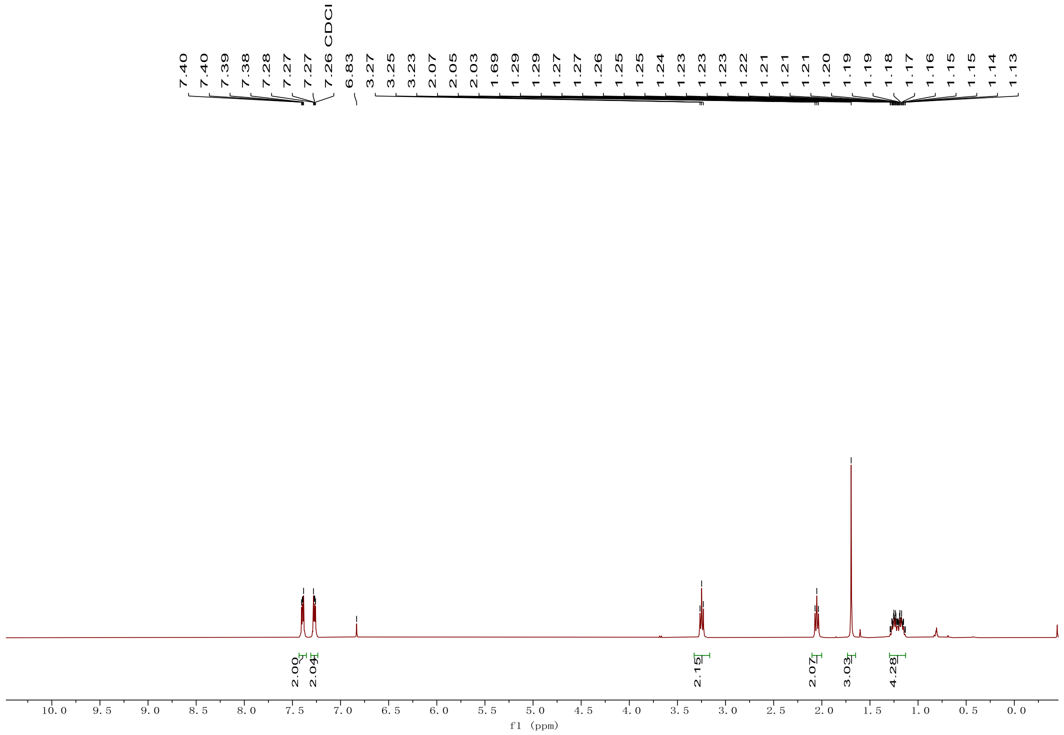

Supplementary Figure 223. ^{1}H NMR (400 MHz, Chloroform-*d*) of **57**

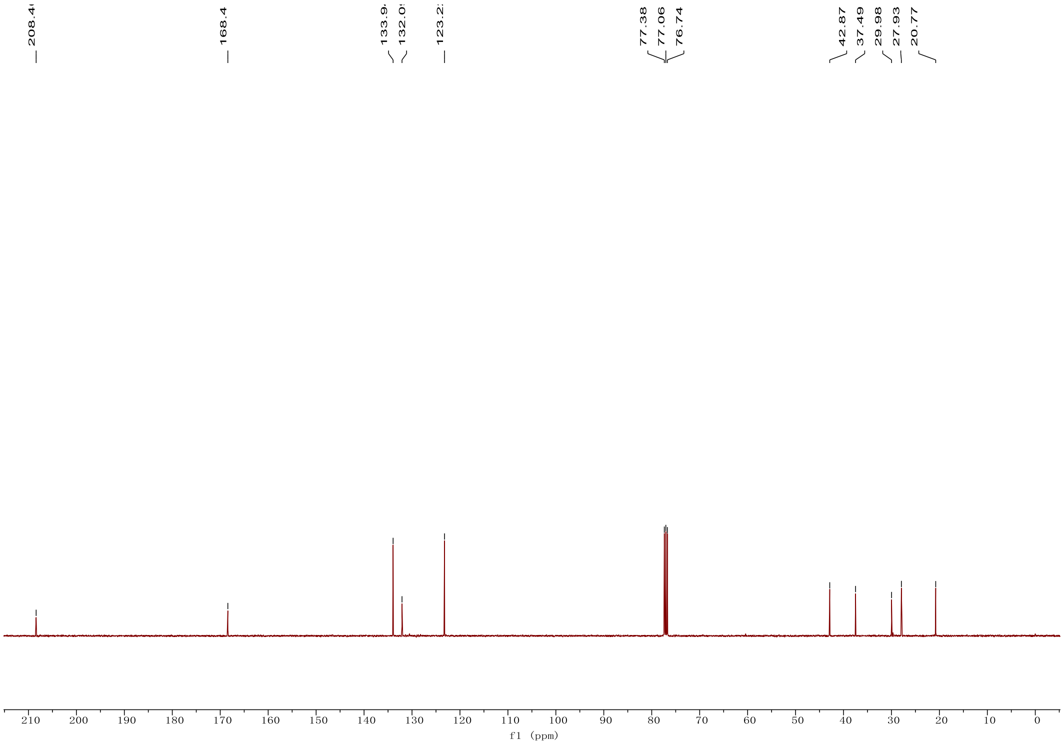

Supplementary Figure 224. ^{13}C NMR (101 MHz, Chloroform-*d*) of **57**

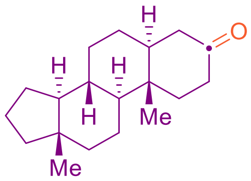

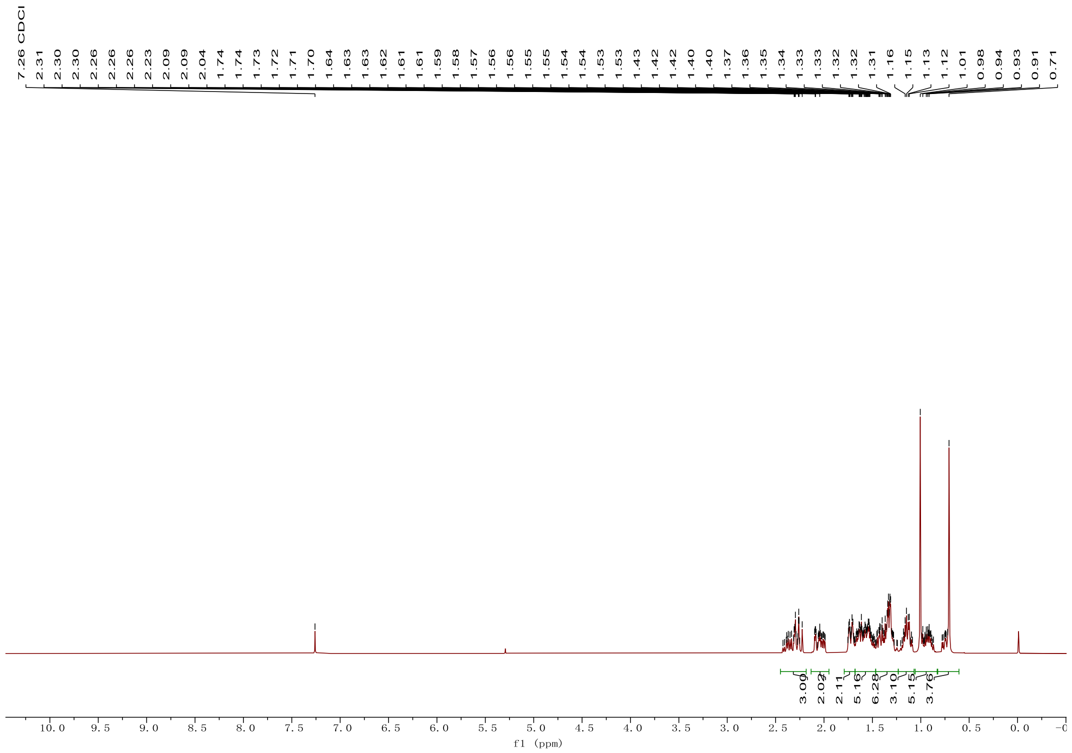

Supplementary Figure 225. **^{1}H NMR** (400 MHz, Chloroform-*d*) of **58**

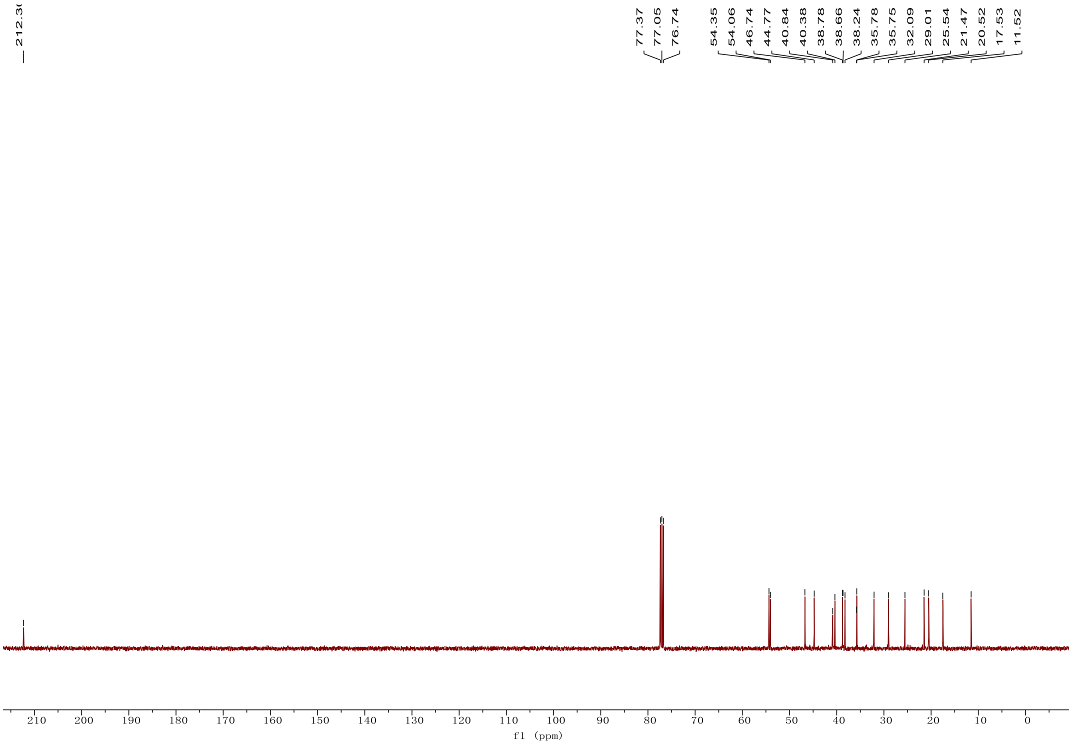

Supplementary Figure 226. **^{13}C NMR (**101 MHz, Chloroform-*d*) of **58**

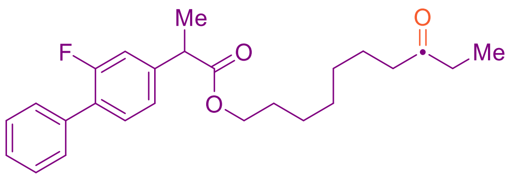

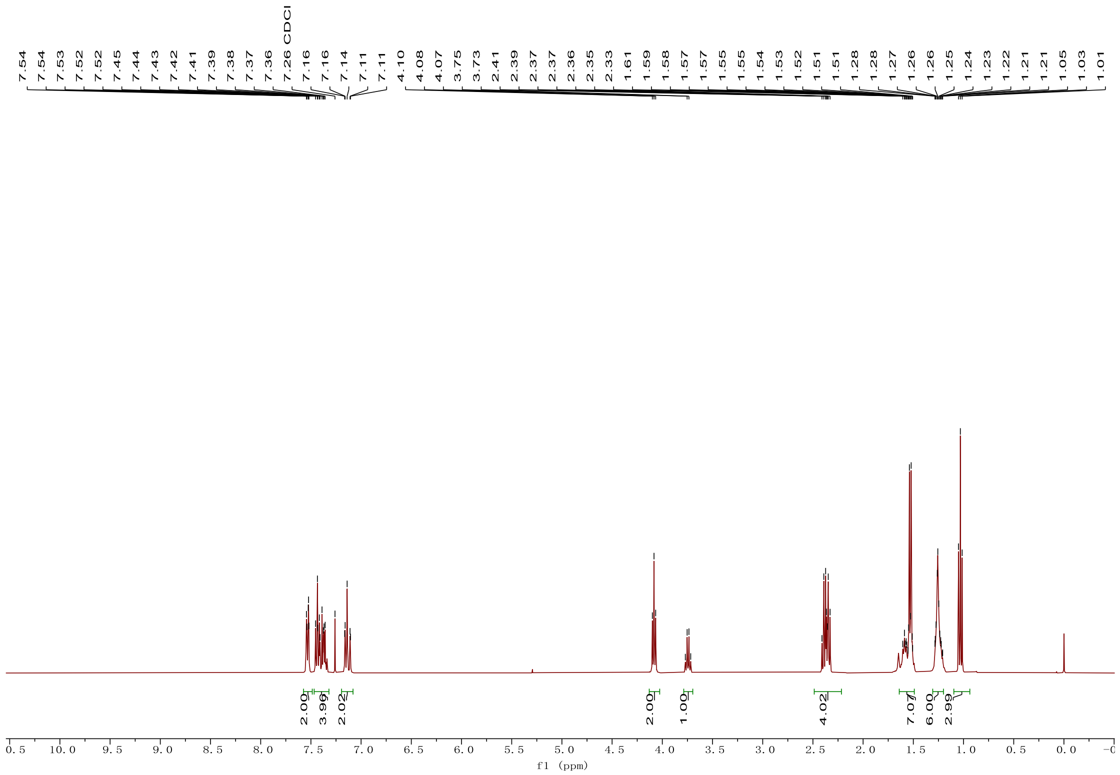

Supplementary Figure 227. **^{1}H NMR** (400 MHz, Chloroform-*d*) of **59**

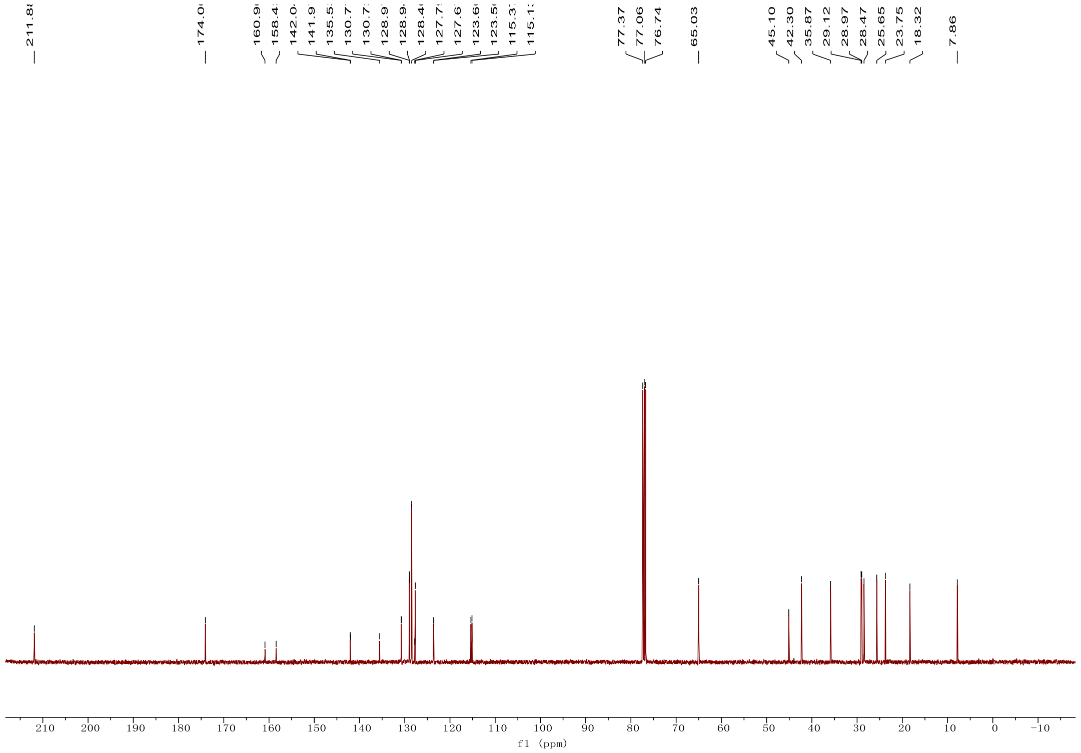

Supplementary Figure 228. **^{13}C NMR (**101 MHz, Chloroform-*d*) of **59**

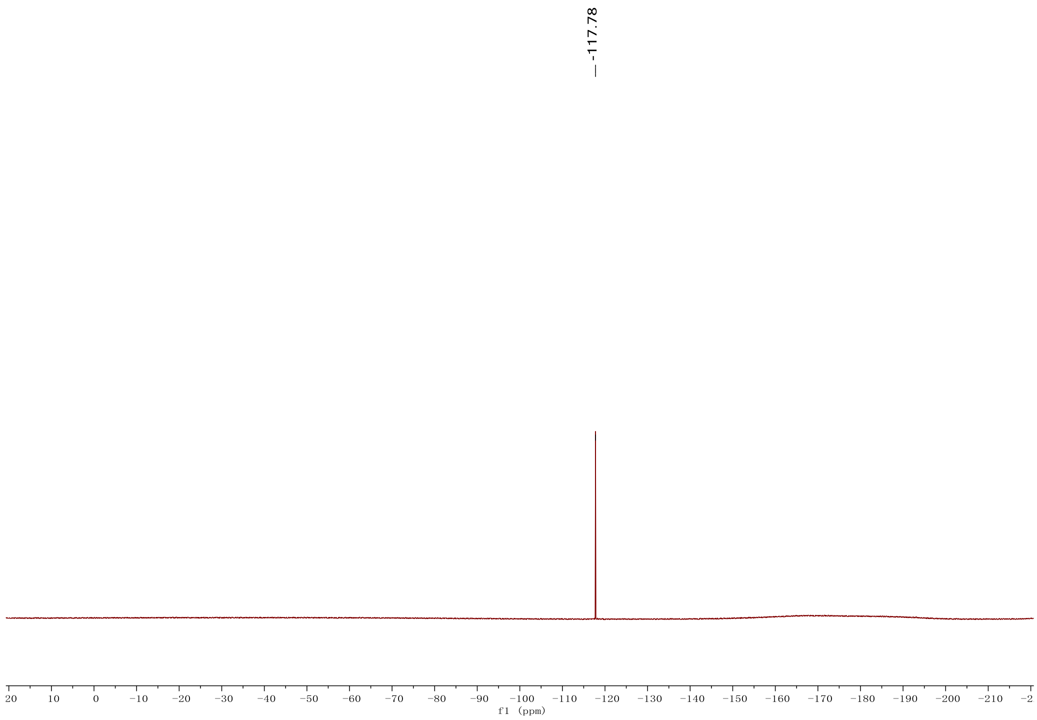

Supplementary Figure 229. ^{19}F NMR (376 MHz, Chloroform-*d*) of **59**

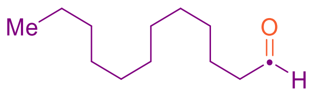

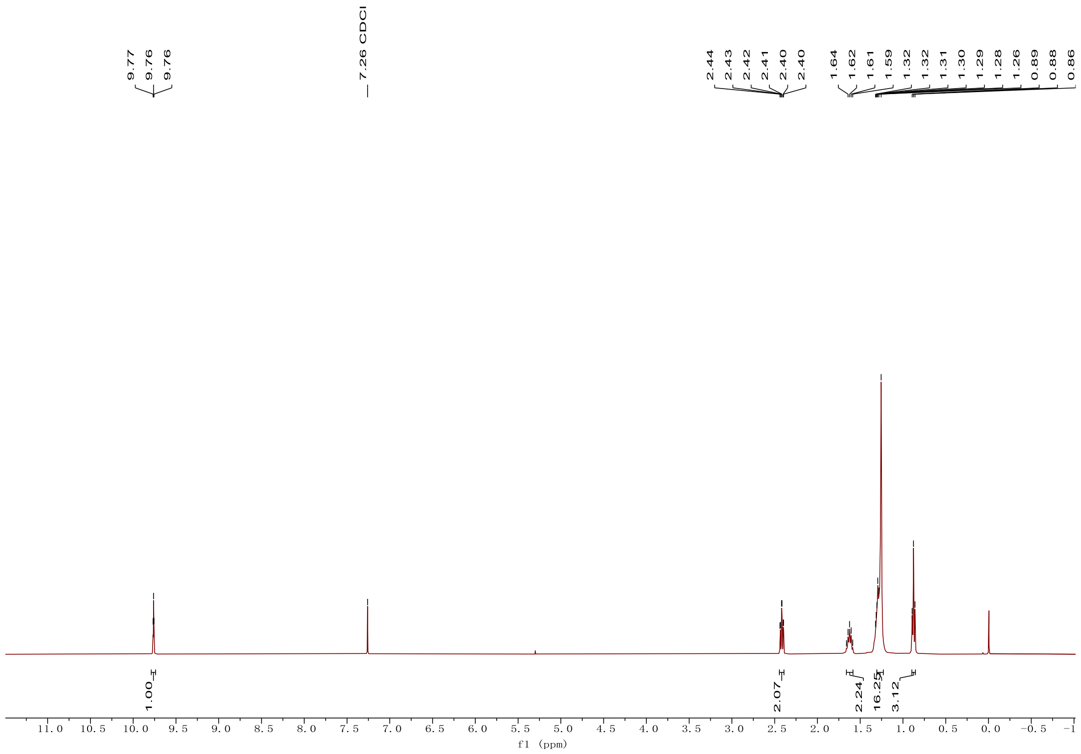

Supplementary Figure 230. ^{1}H NMR (400 MHz, Chloroform-*d*) of **60**

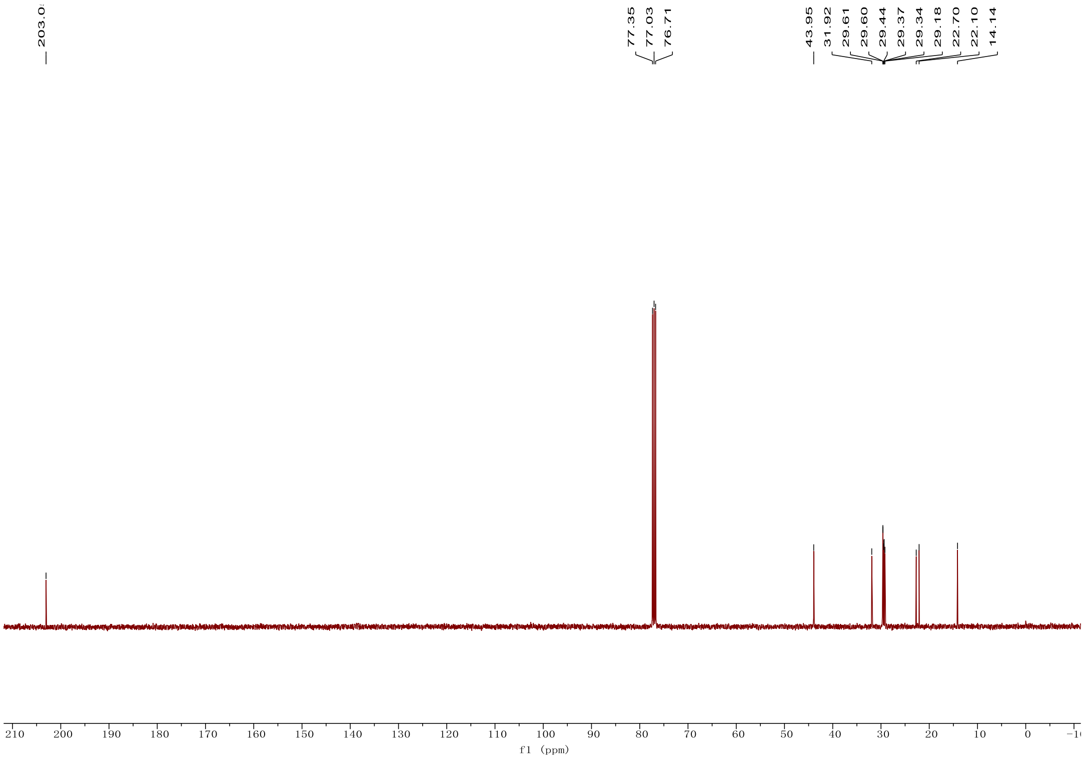

Supplementary Figure 231. ^{13}C NMR (101 MHz, Chloroform-*d*) of **60**

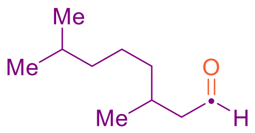

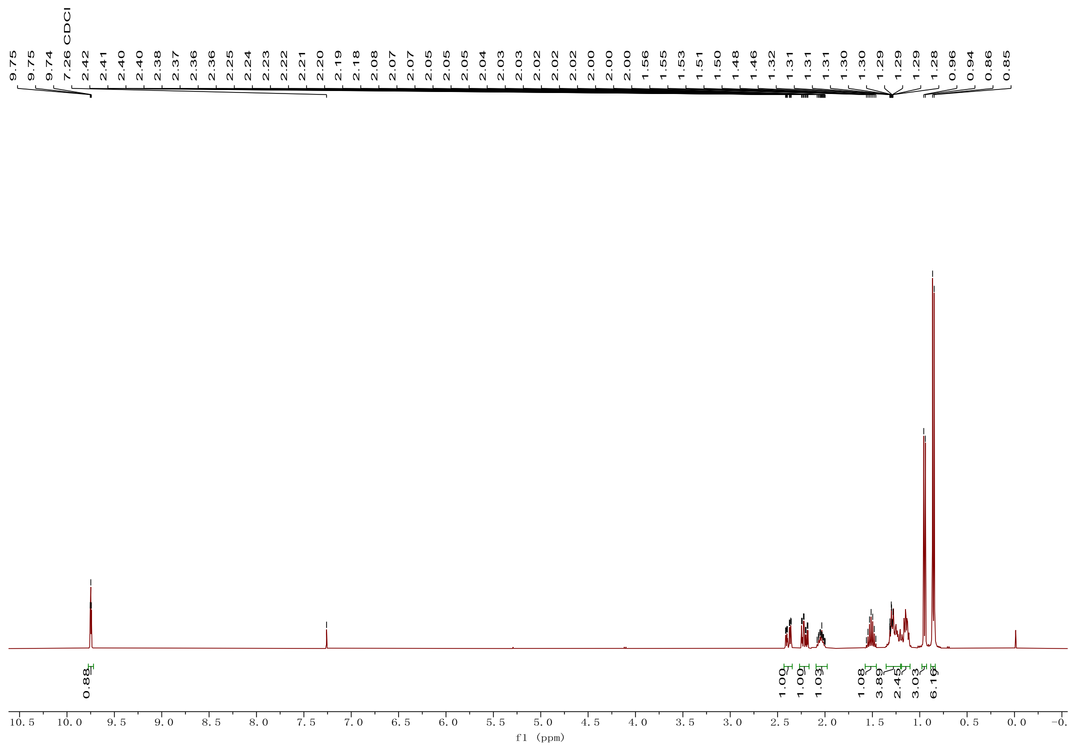

Supplementary Figure 232. ^{1}**H NMR** (400 MHz, Chloroform-*d*) of **61**

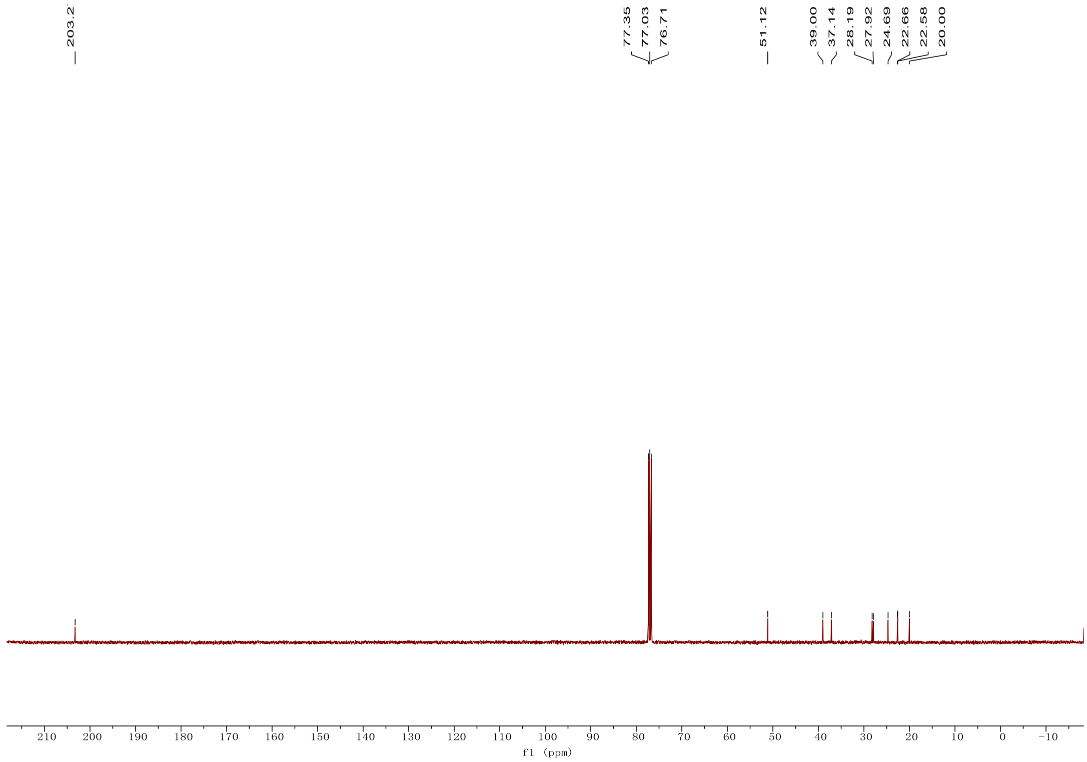

Supplementary Figure 233. ^{13}**C NMR (**101 MHz, Chloroform-*d*) of **61**

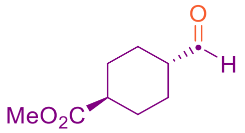

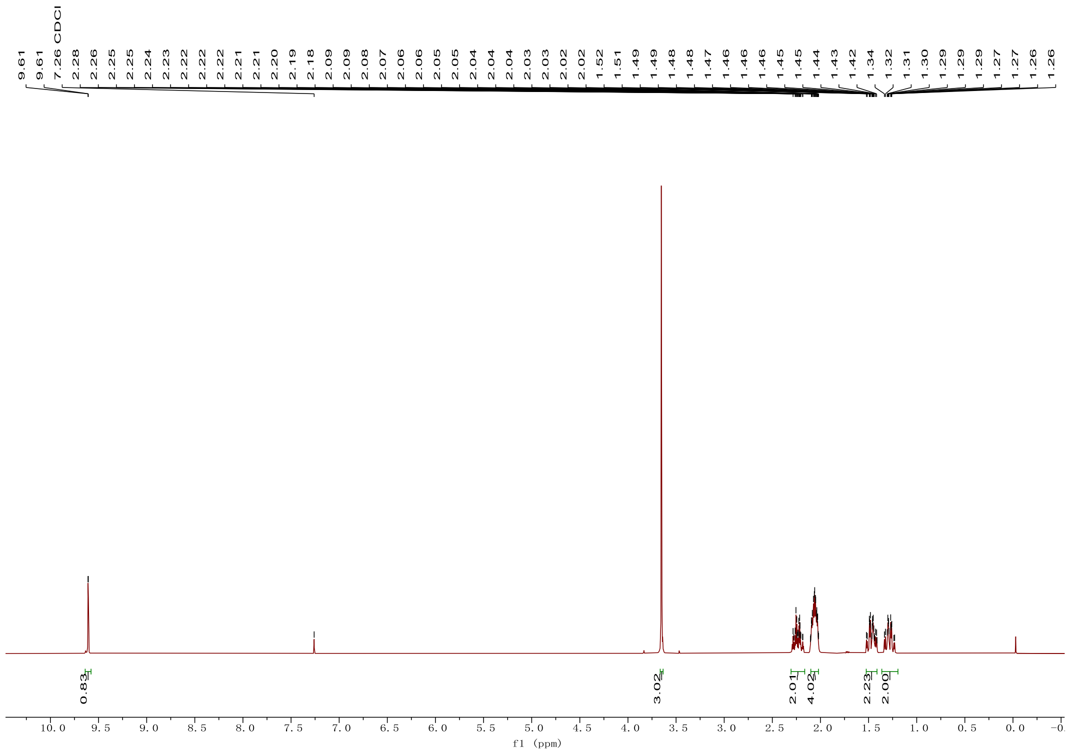

Supplementary Figure 234. ^{1}**H NMR** (400 MHz, Chloroform-*d*) of **62**

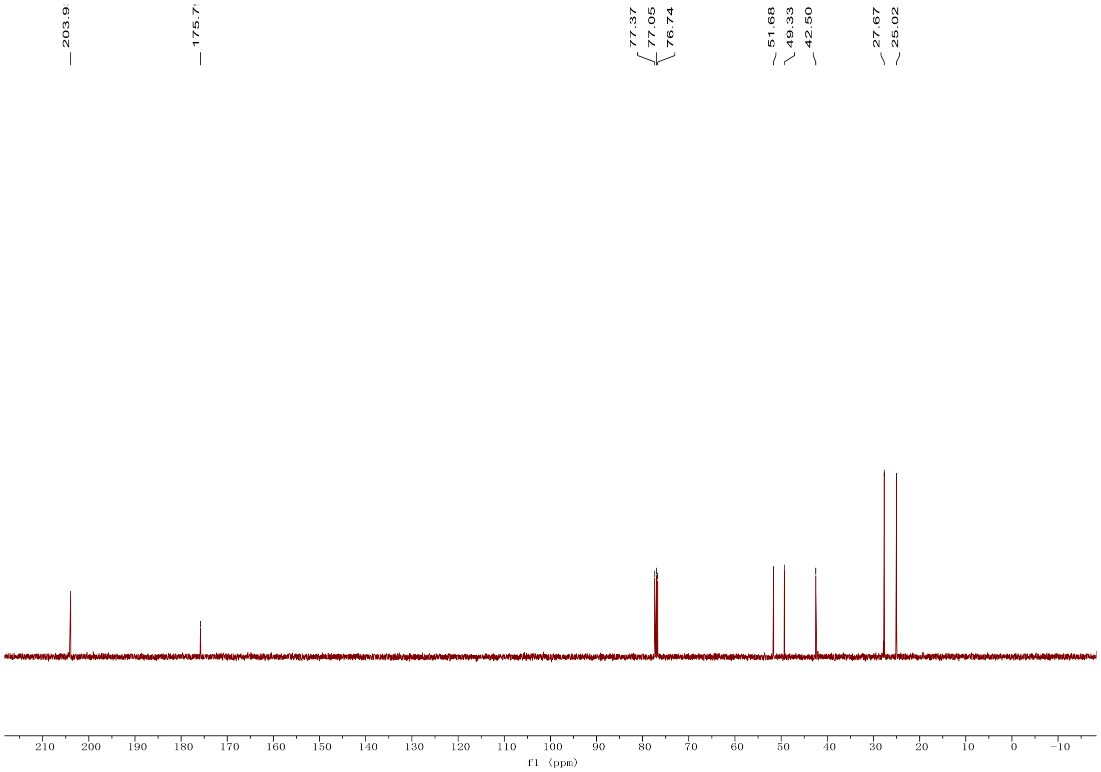

Supplementary Figure 235. ^{13}**C NMR (**101 MHz, Chloroform-*d*) of **62**

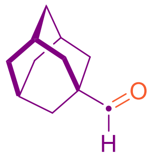

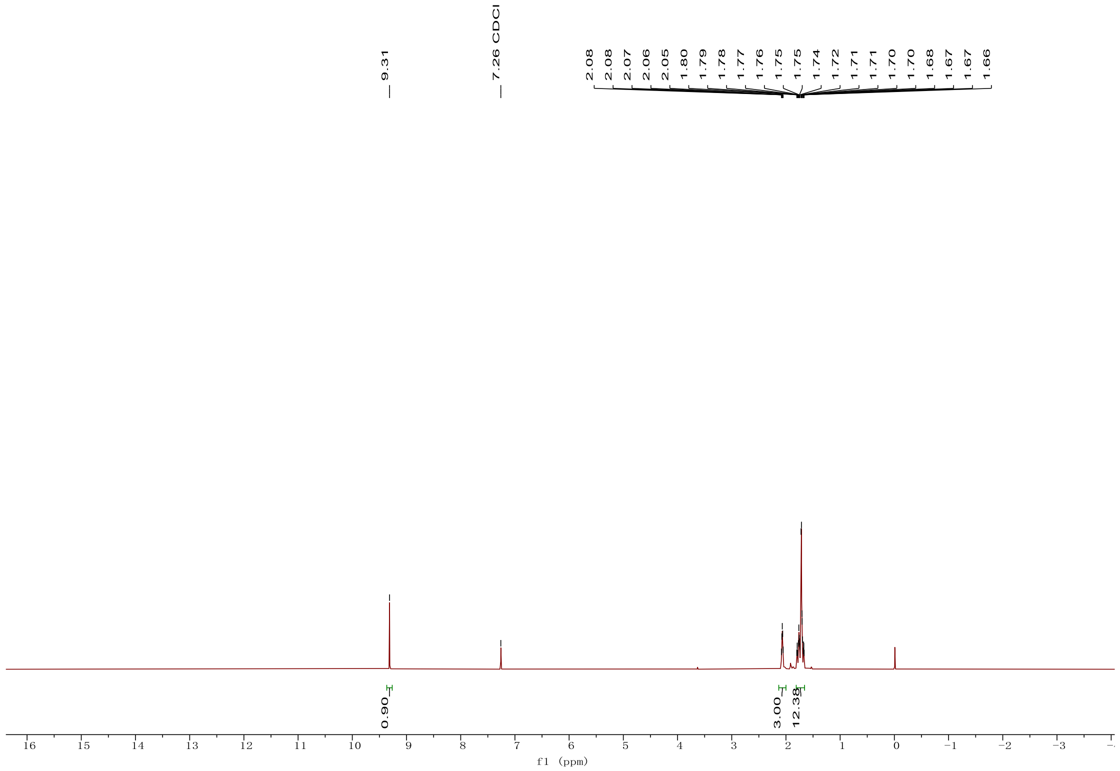

Supplementary Figure 236. ^{1}H NMR (400 MHz, Chloroform-*d*) of **63**

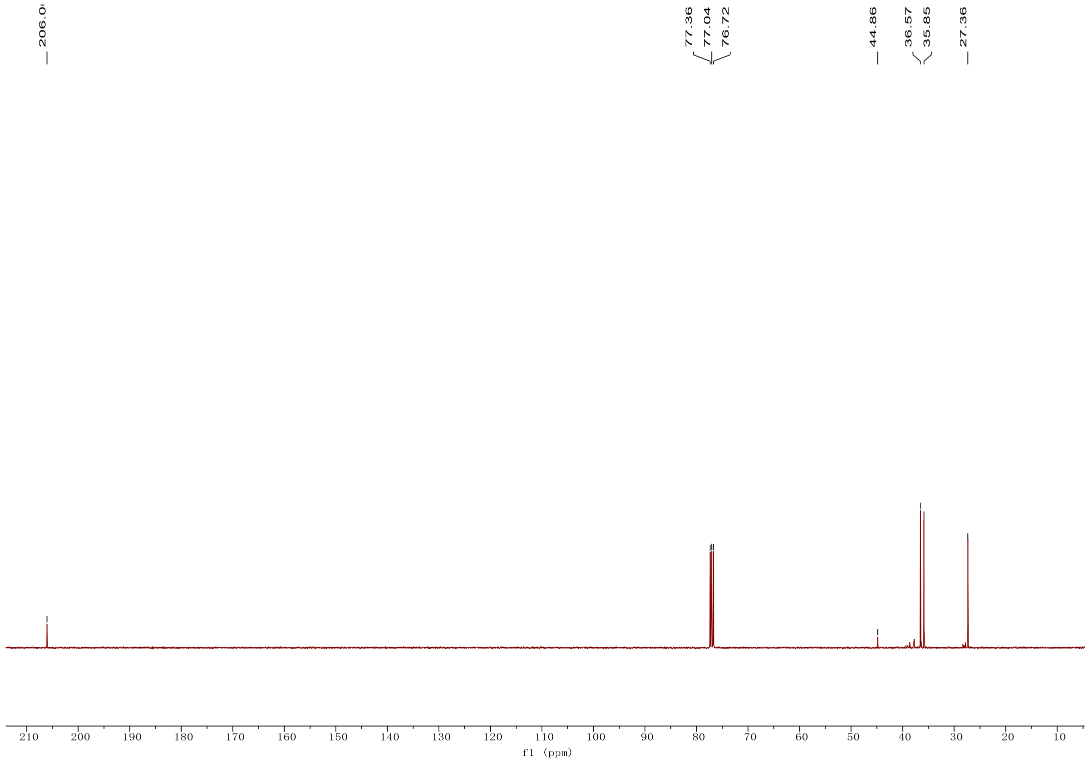

Supplementary Figure 237. ^{13}C NMR (101 MHz, Chloroform-*d*) of **63**

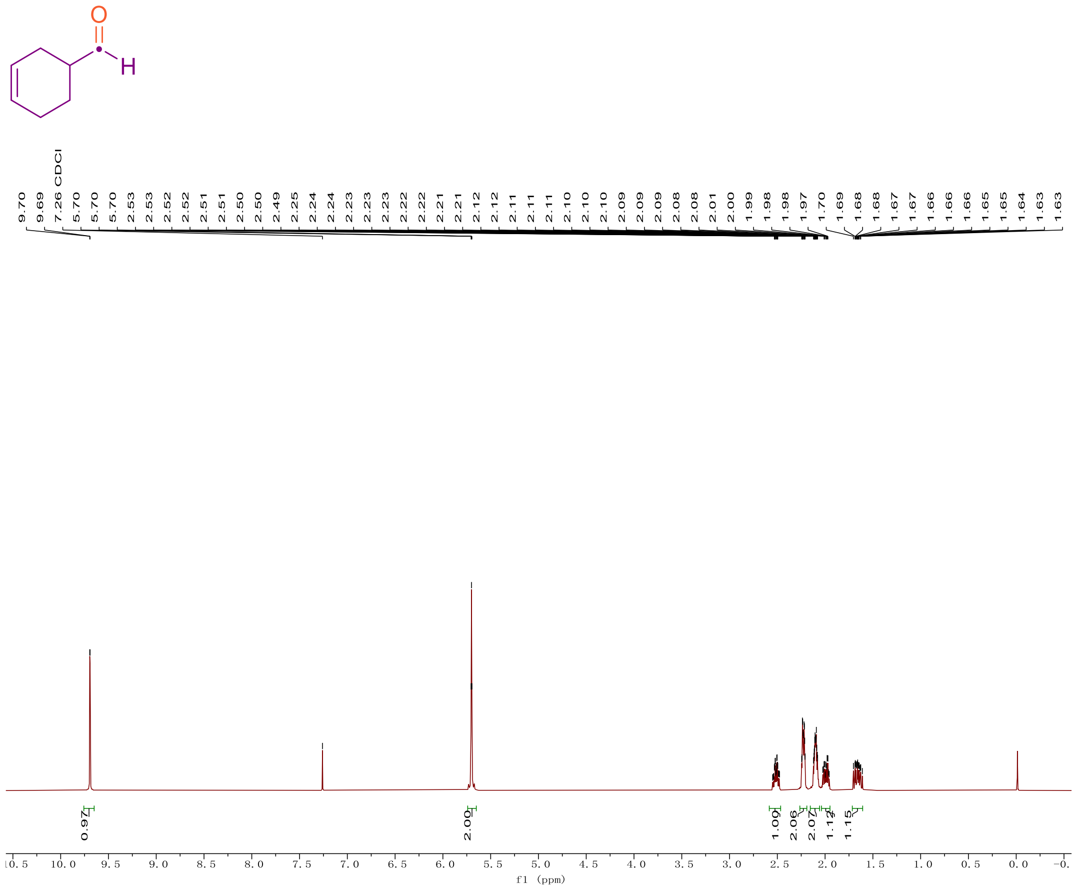

Supplementary Figure 238. ^{1}H NMR (400 MHz, Chloroform-*d*) of **64**

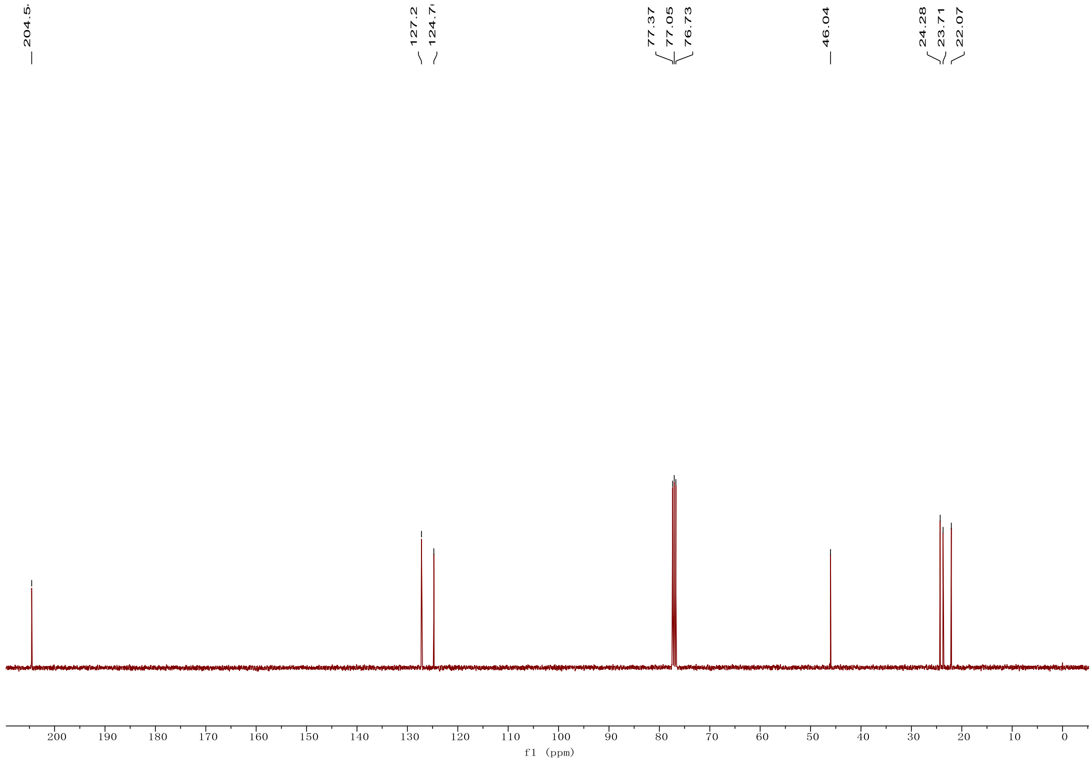

Supplementary Figure 239. ^{13}C NMR (101 MHz, Chloroform-*d*) of **64**

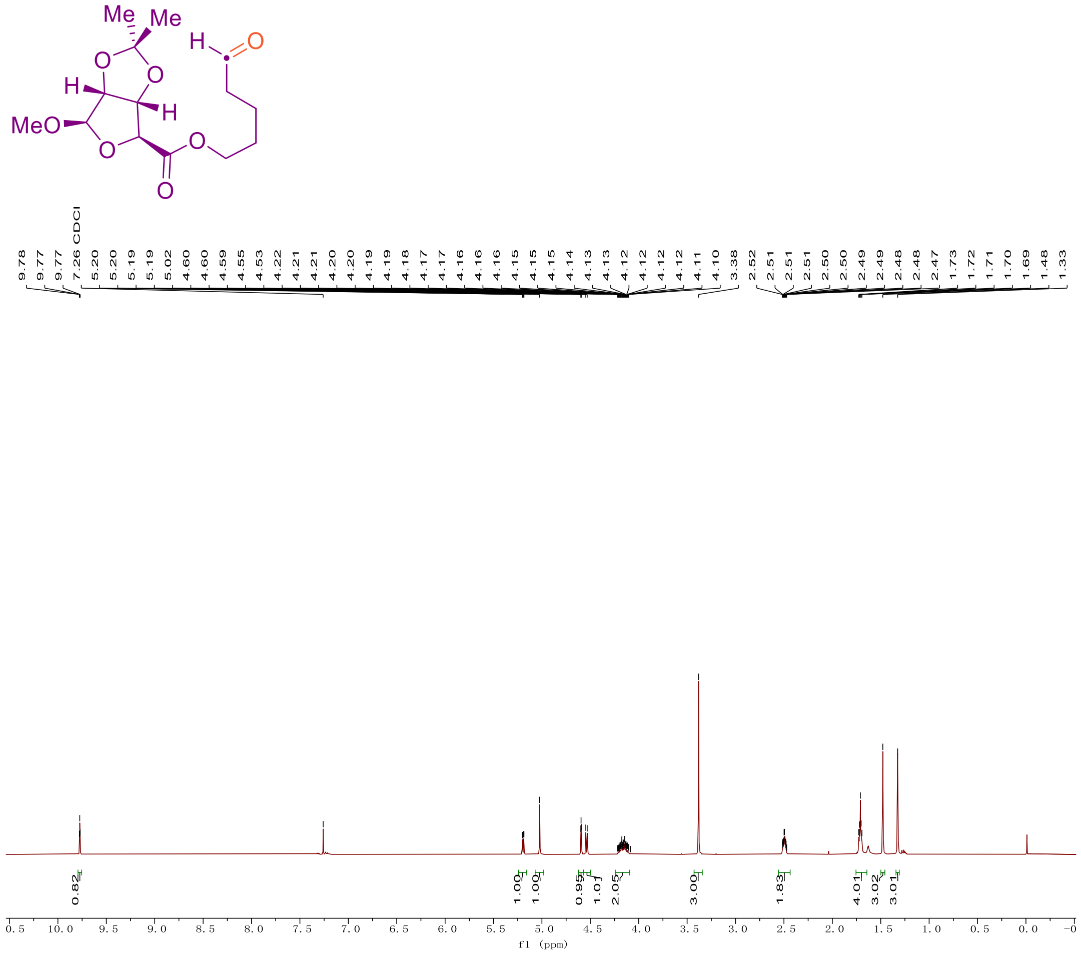

Supplementary Figure 240. ^{1}H NMR (400 MHz, Chloroform-*d*) of **65**

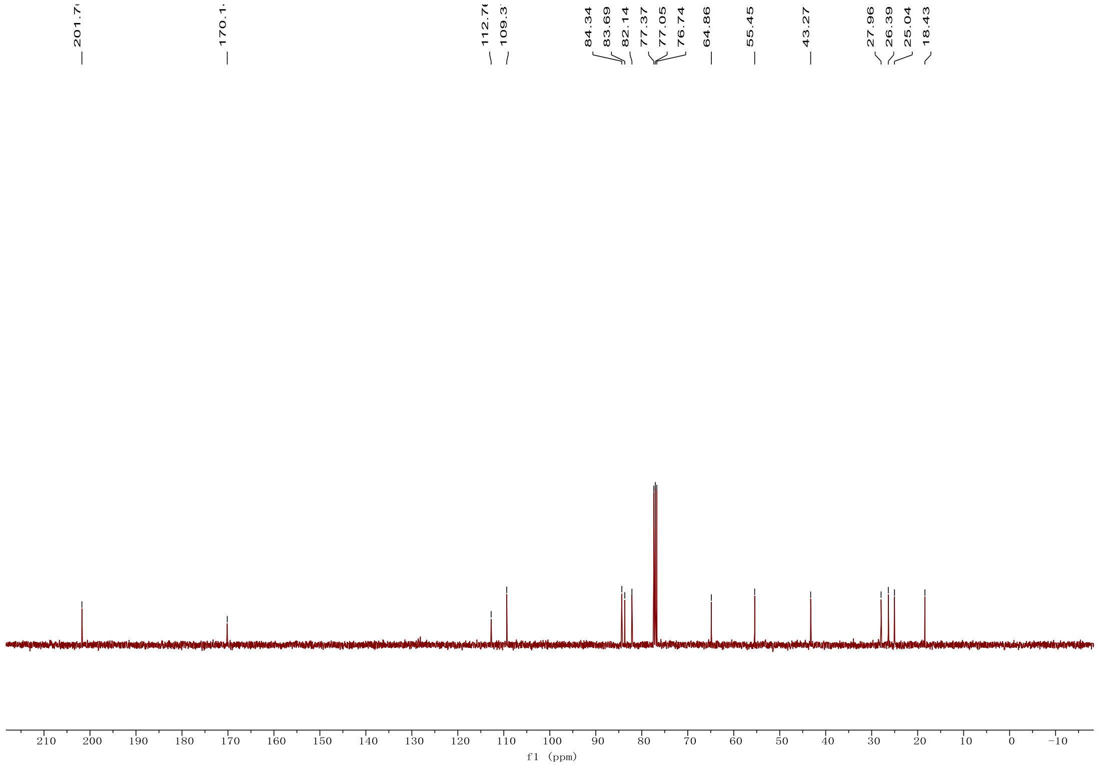

Supplementary Figure 241. ^{13}C NMR (101 MHz, Chloroform-*d*) of **65**

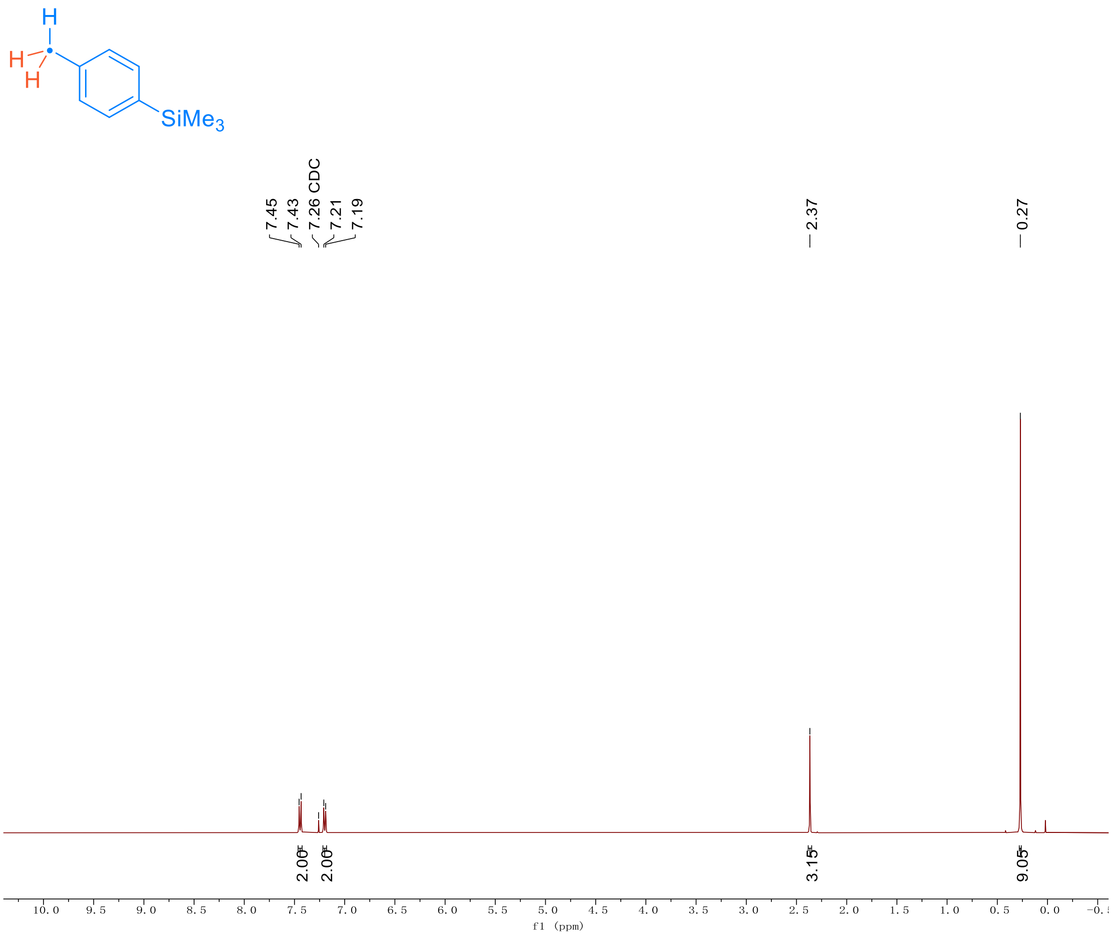

Supplementary Figure 242. ^{1}H NMR (400 MHz, Chloroform-*d*) of **66**

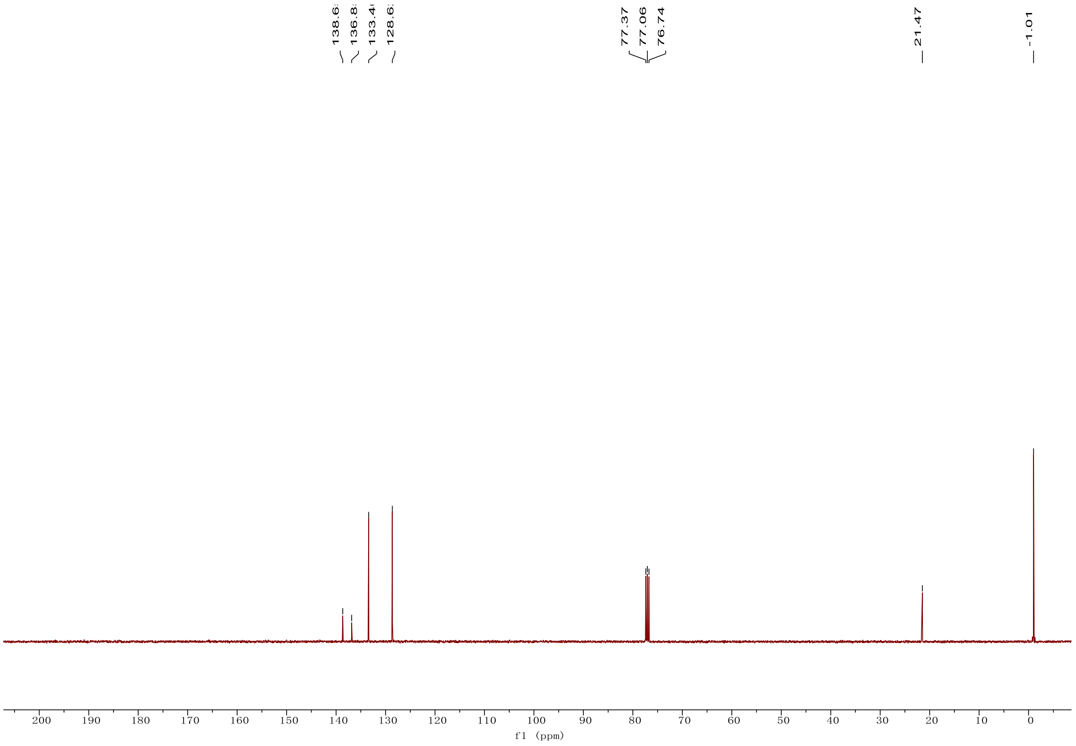

Supplementary Figure 243. ^{13}C NMR (101 MHz, Chloroform-*d*) of **66**

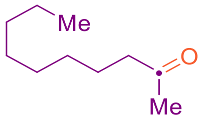

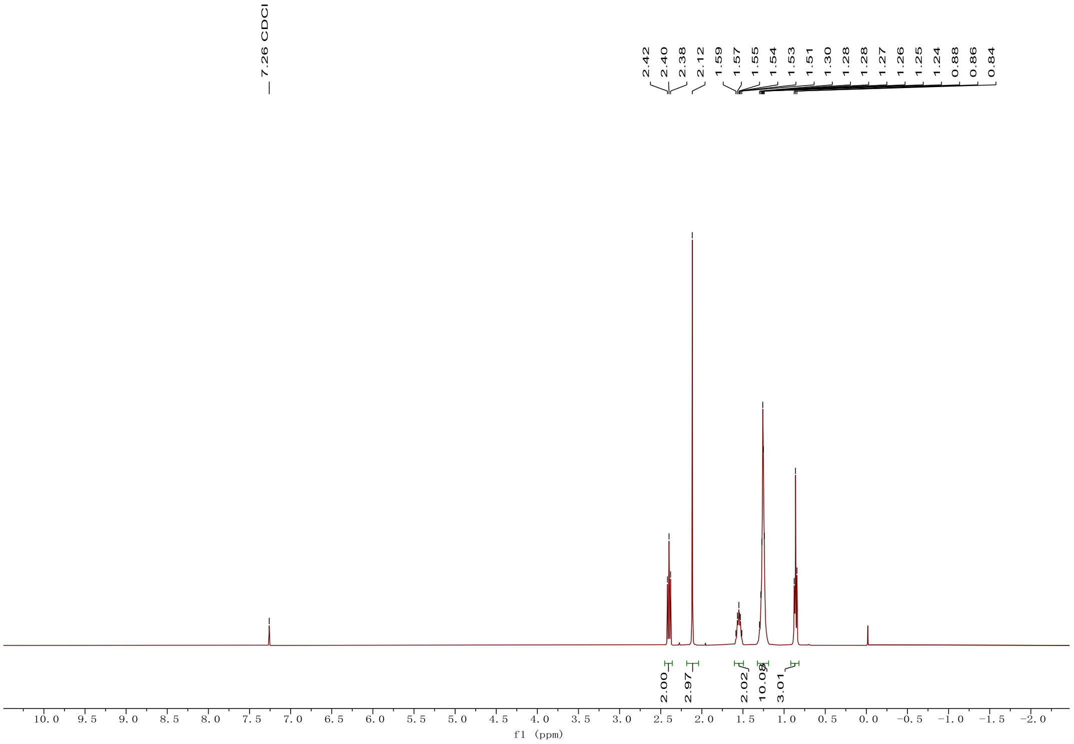

Supplementary Figure 244. ^{1}H NMR (400 MHz, Chloroform-*d*) of **69**

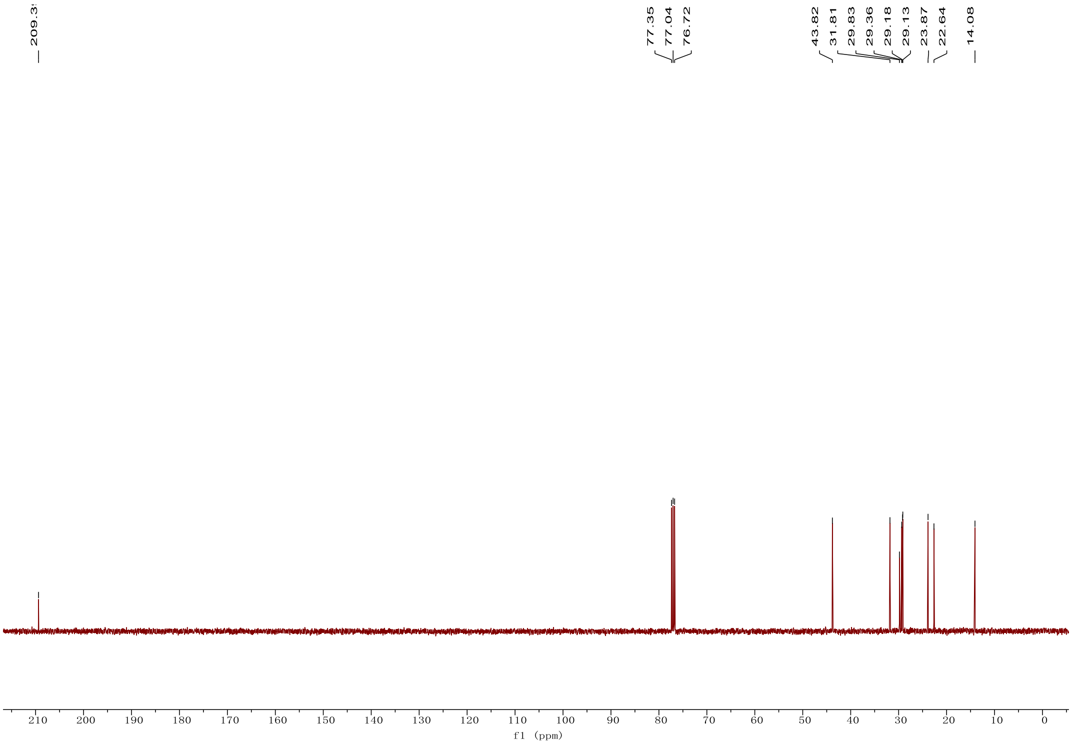

Supplementary Figure 245. ^{13}C NMR (101 MHz, Chloroform-*d*) of **69**

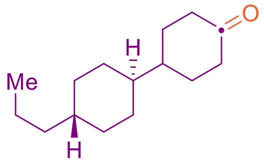

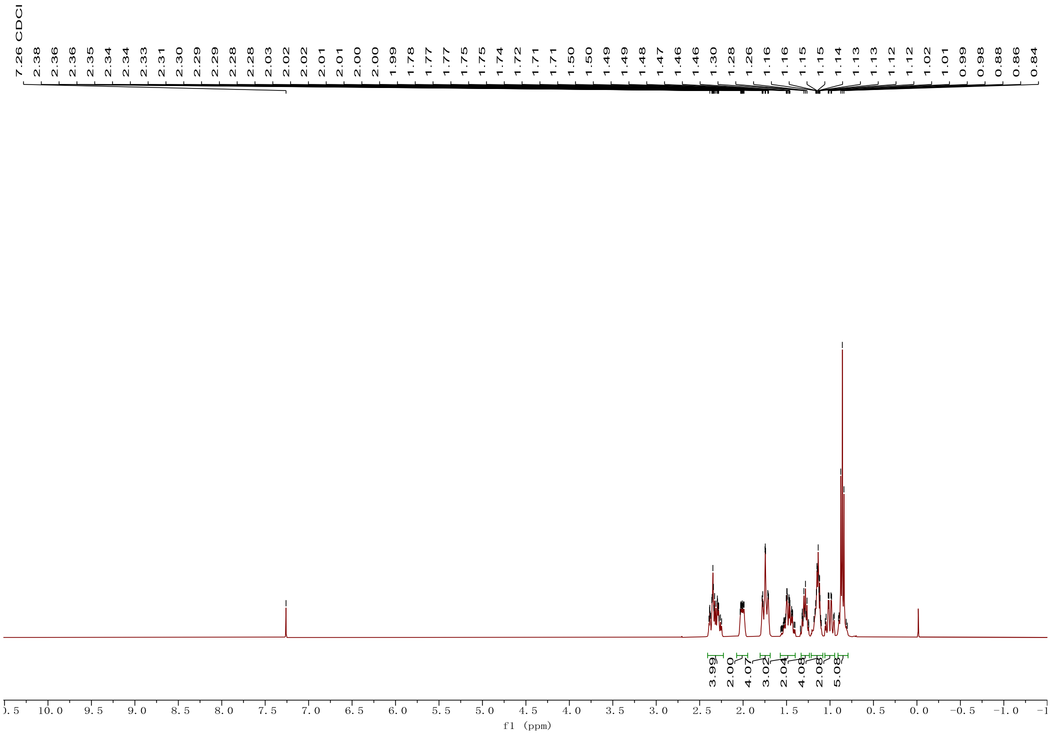

Supplementary Figure 246. ^{1}H NMR (400 MHz, Chloroform-*d*) of **71**

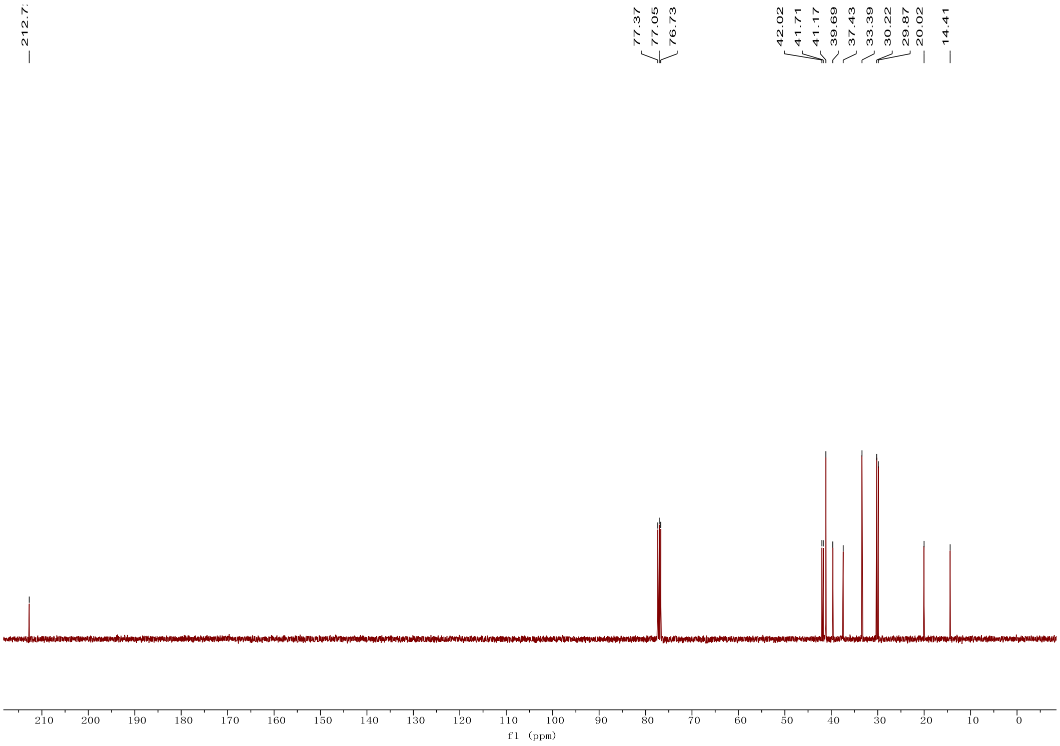

Supplementary Figure 247. ^{13}C NMR (101 MHz, Chloroform-*d*) of **71**

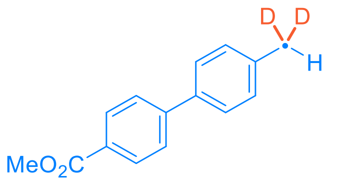

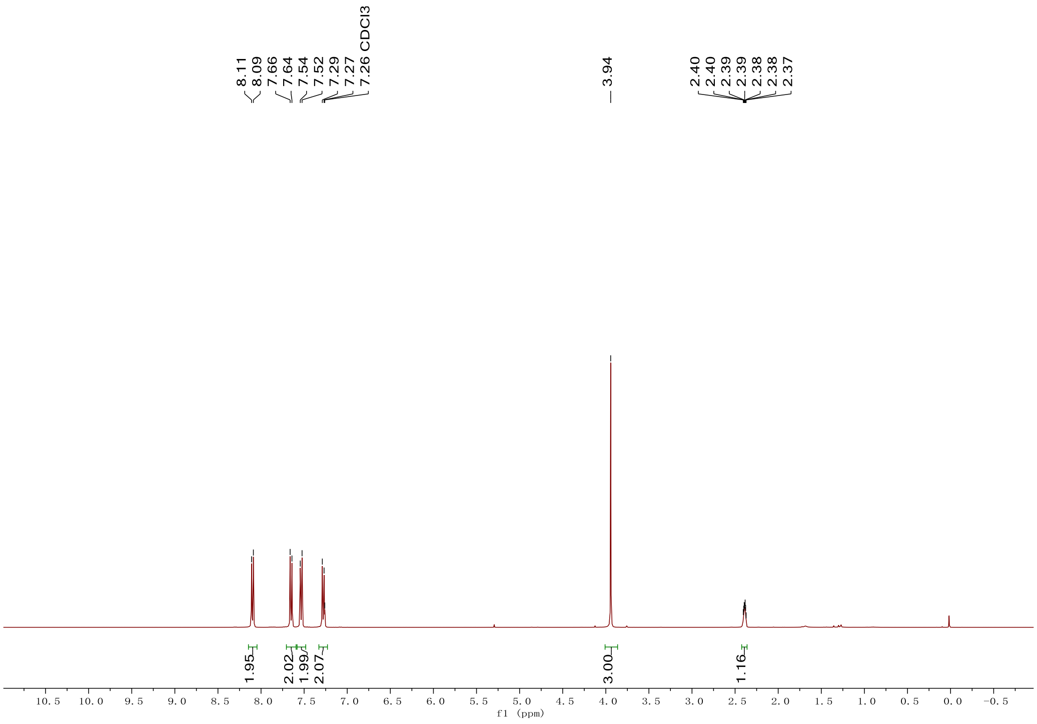

Supplementary Figure 248. ^{1}**H NMR** (400 MHz, Chloroform-*d*) of **72**

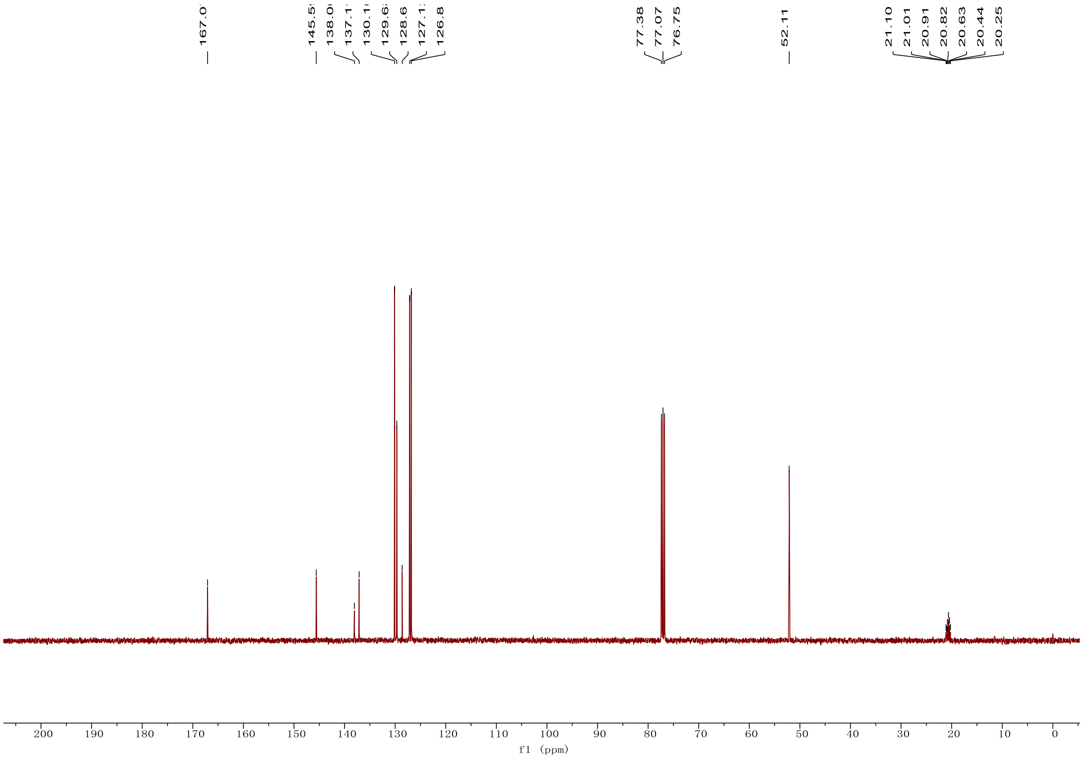

Supplementary Figure 249. ^{13}**C NMR (**101 MHz, Chloroform-*d*) of **72**

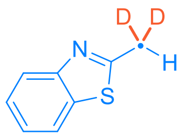

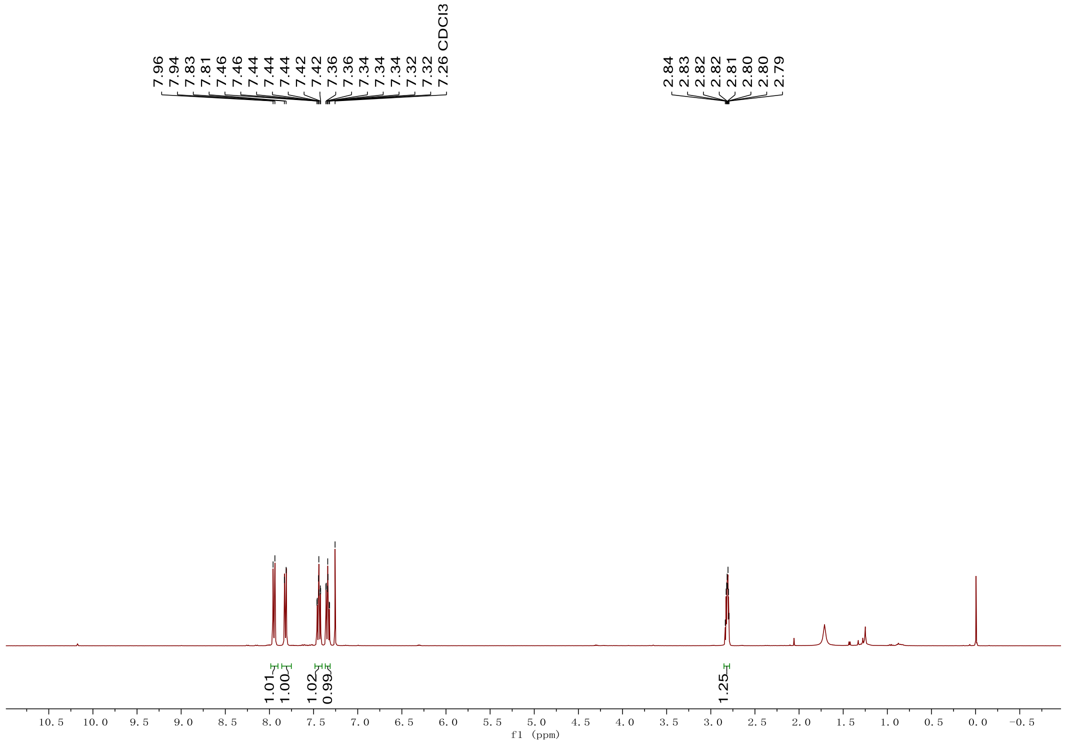

Supplementary Figure 250. ^{1}**H NMR** (400 MHz, Chloroform-*d*) of **73**

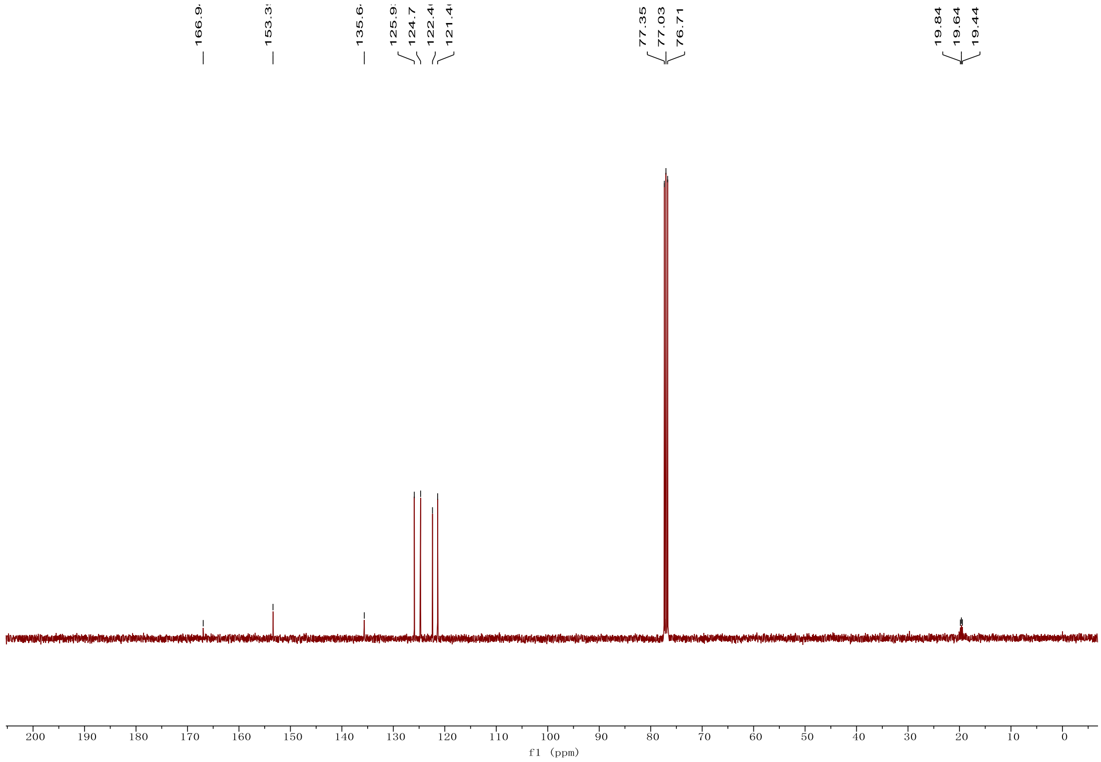

Supplementary Figure 251. ^{13}**C NMR (**101 MHz, Chloroform-*d*) of **73**

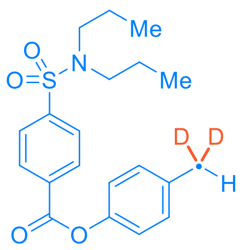

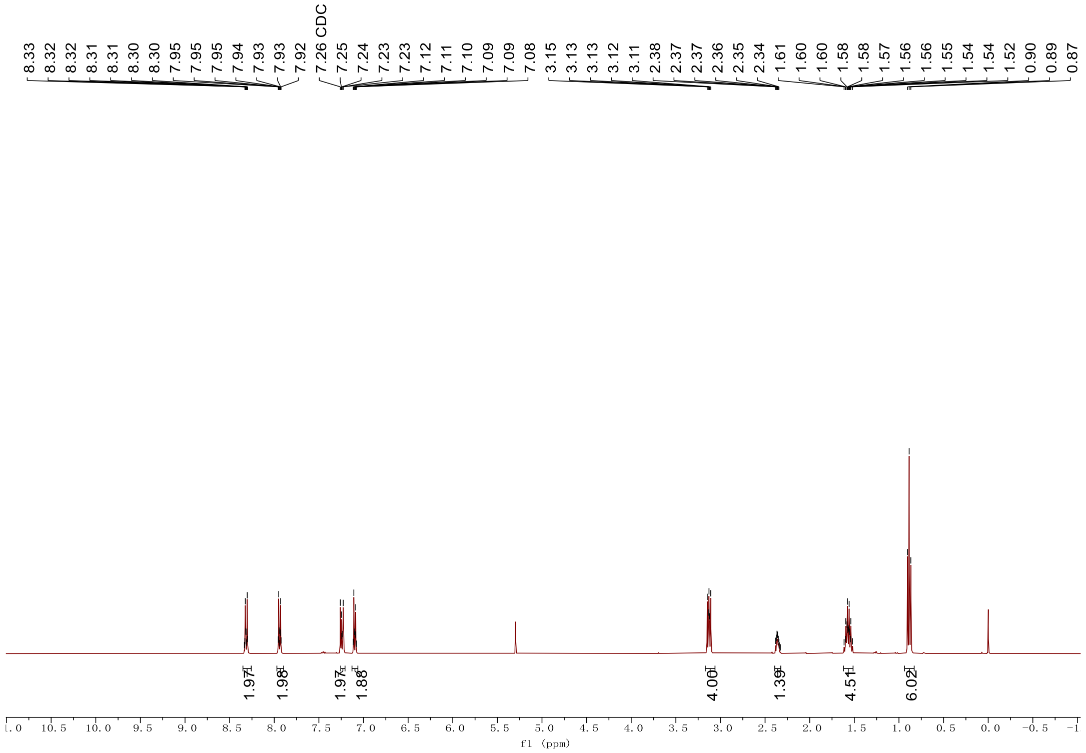

Supplementary Figure 252. **^{1}H NMR** (400 MHz, Chloroform-*d*) of **74**

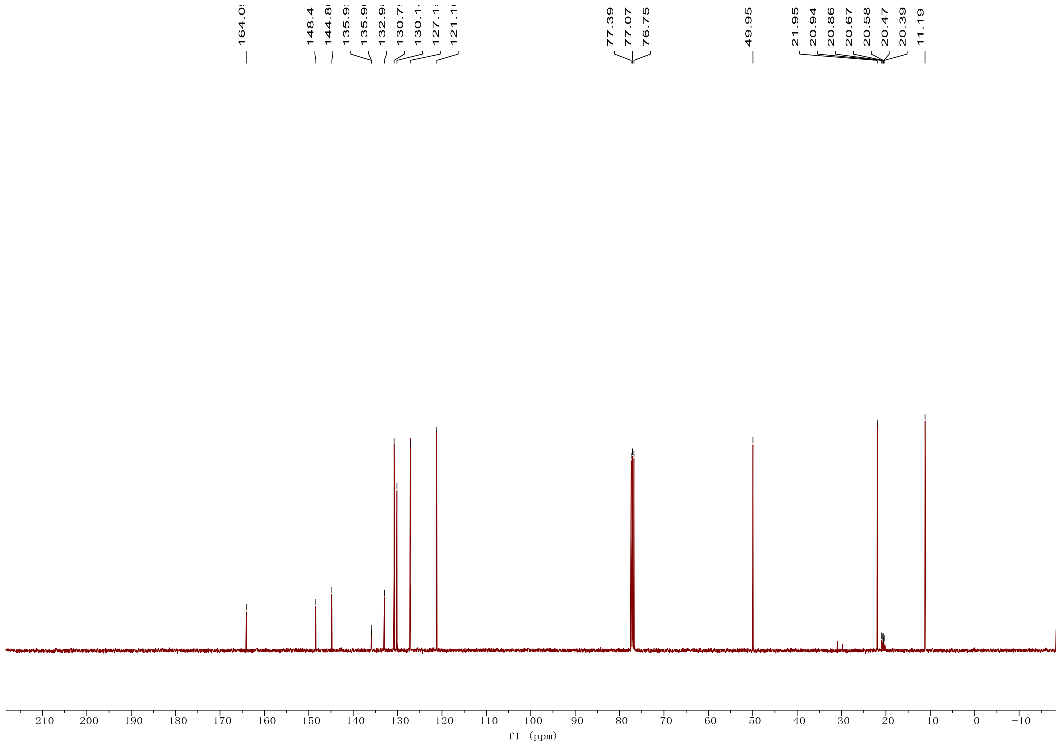

Supplementary Figure 253. ^{13}C NMR (101 MHz, Chloroform-*d*) of **74**

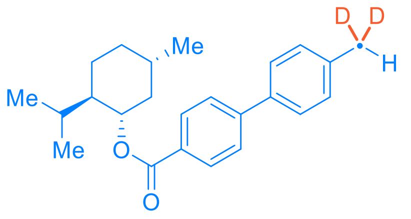

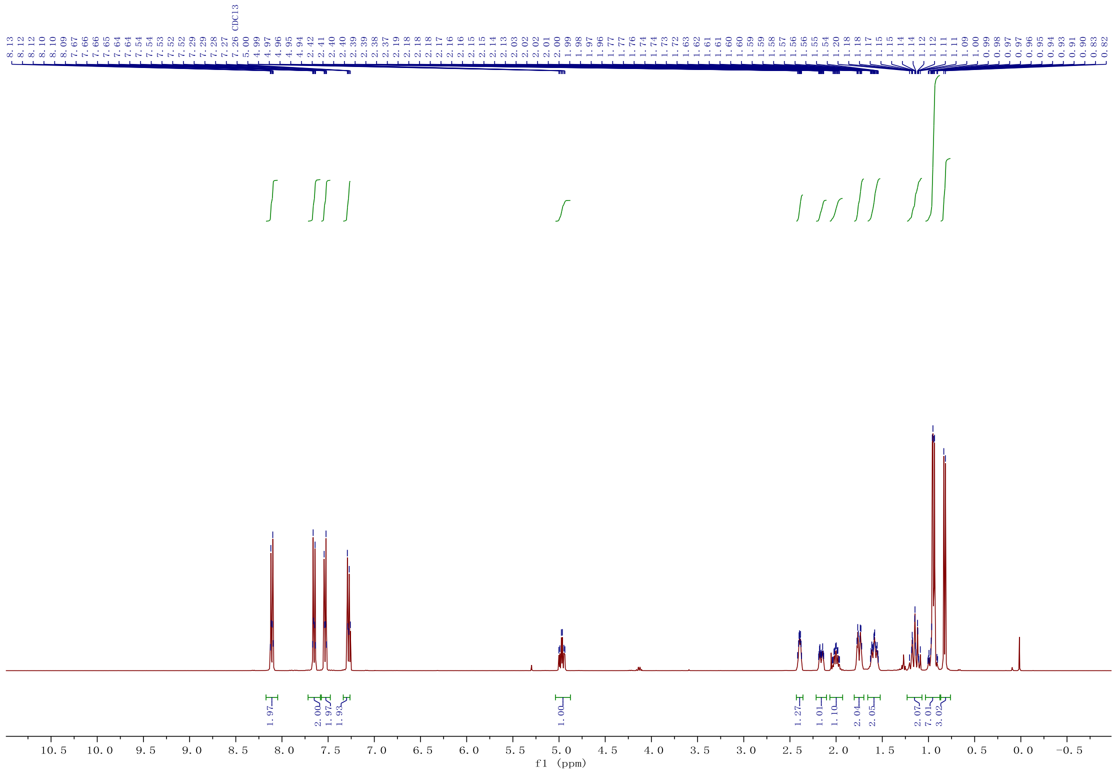

Supplementary Figure 254. **^{1}H NMR** (400 MHz, Chloroform-*d*) of **75**

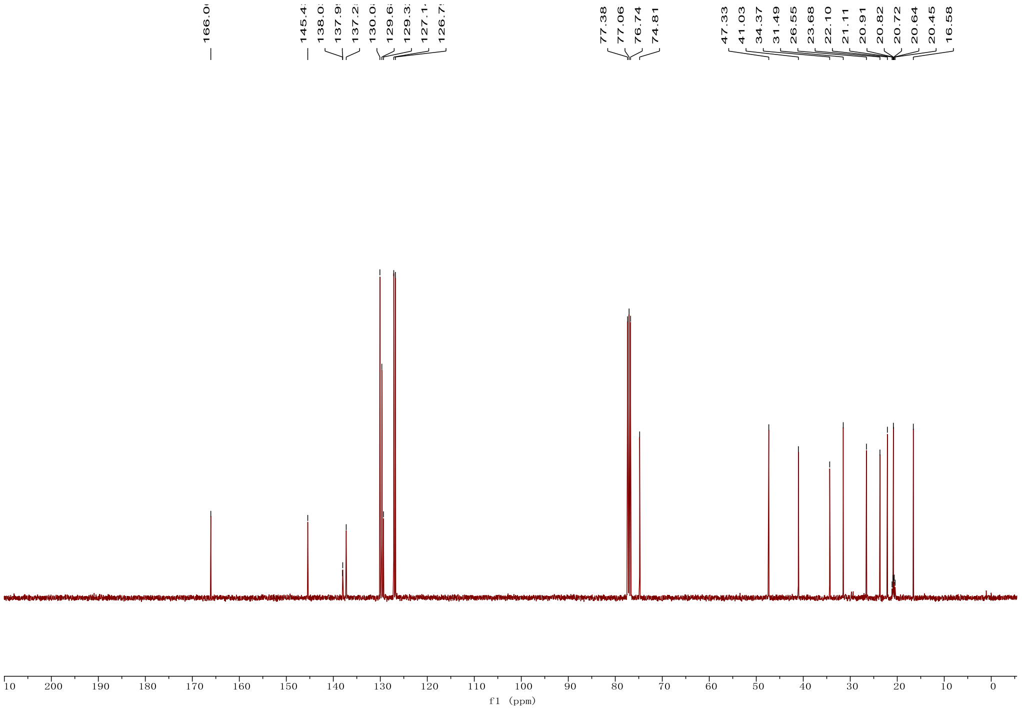

Supplementary Figure 255. **^{13}C NMR (**101 MHz, Chloroform-*d*) of **75**

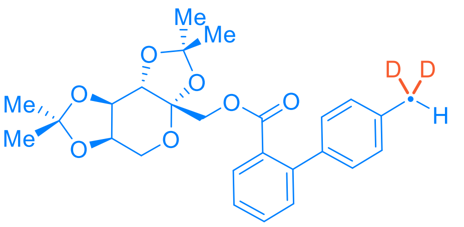

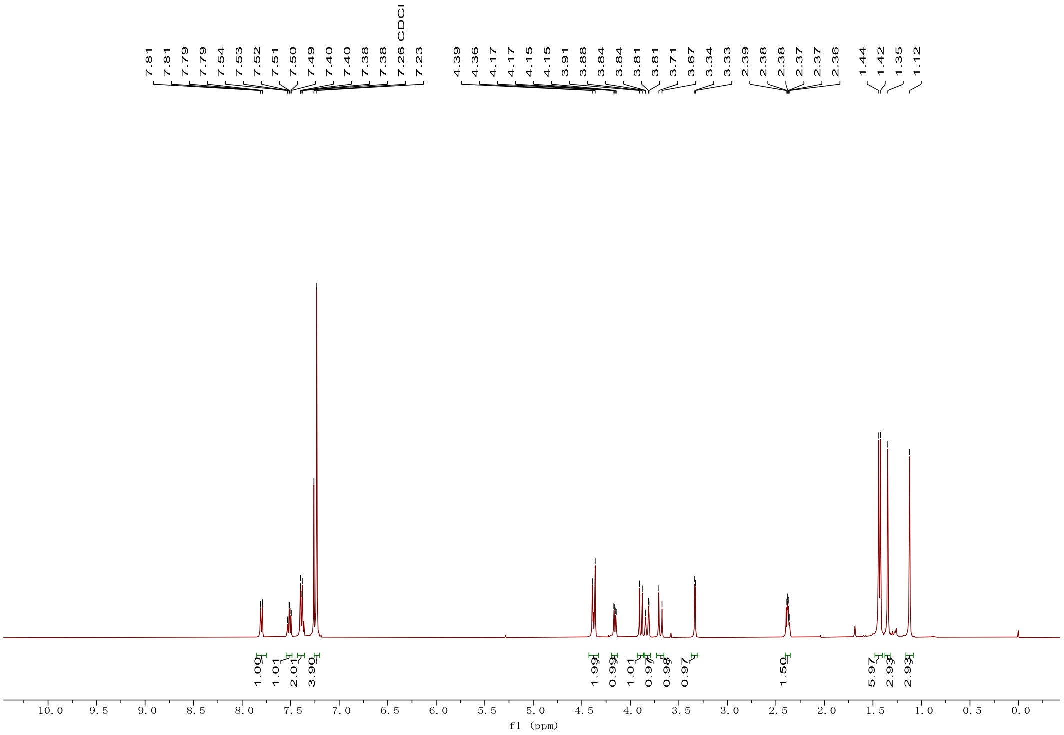

Supplementary Figure 256. **^{1}H NMR** (400 MHz, Chloroform-*d*) of **76**

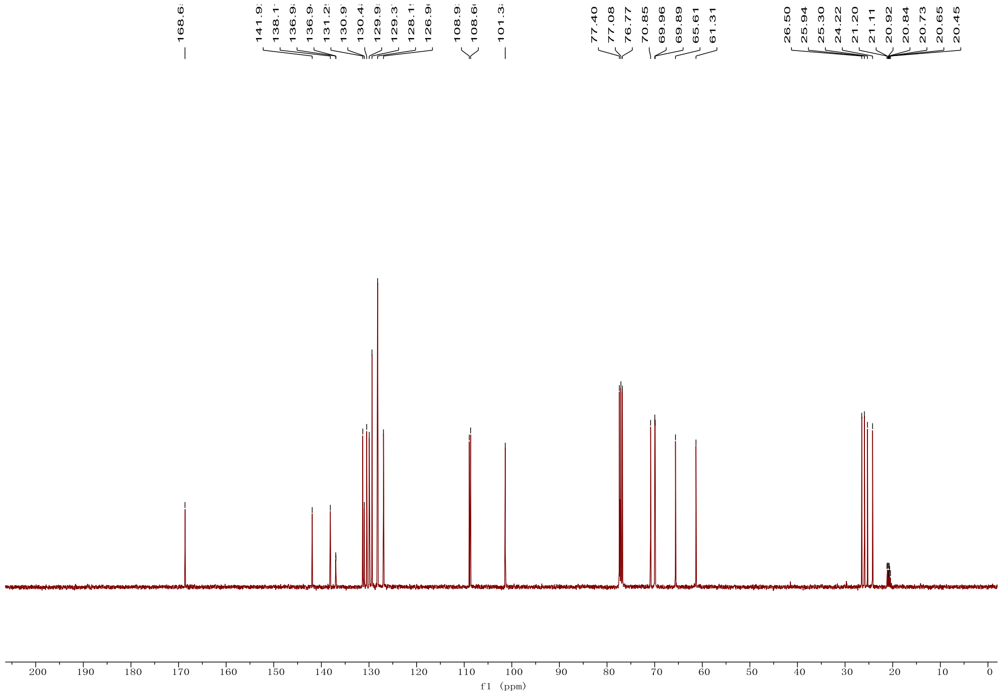

Supplementary Figure 257. **^{13}C NMR (**101 MHz, Chloroform-*d*) of **76**

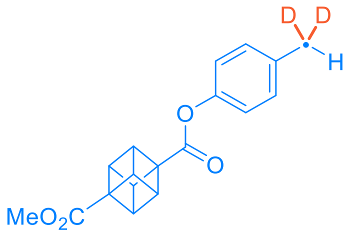

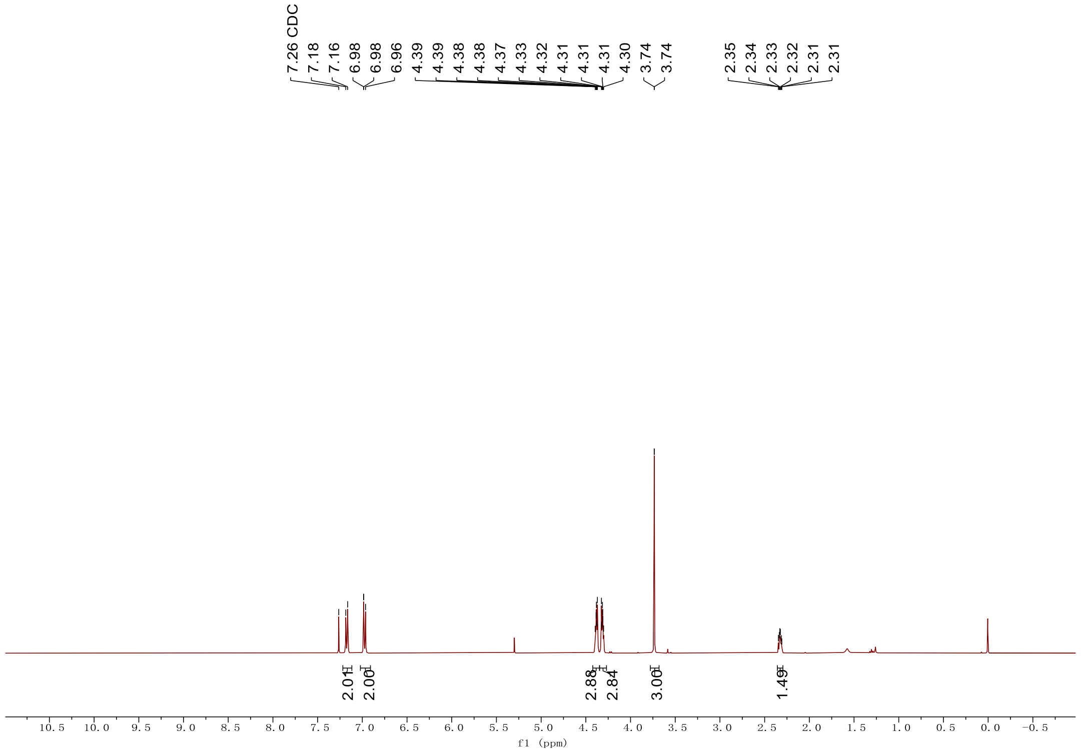

Supplementary Figure 258. ^{1}H NMR (400 MHz, Chloroform-*d*) of **77**

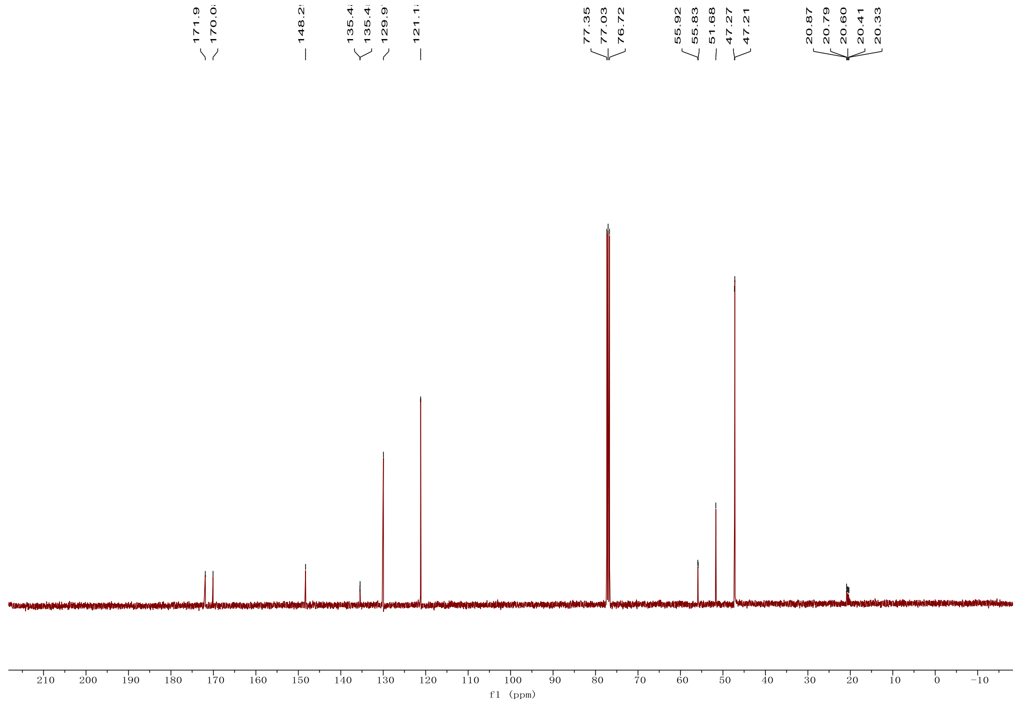

Supplementary Figure 259. ^{13}C NMR (101 MHz, Chloroform-*d*) of **77**

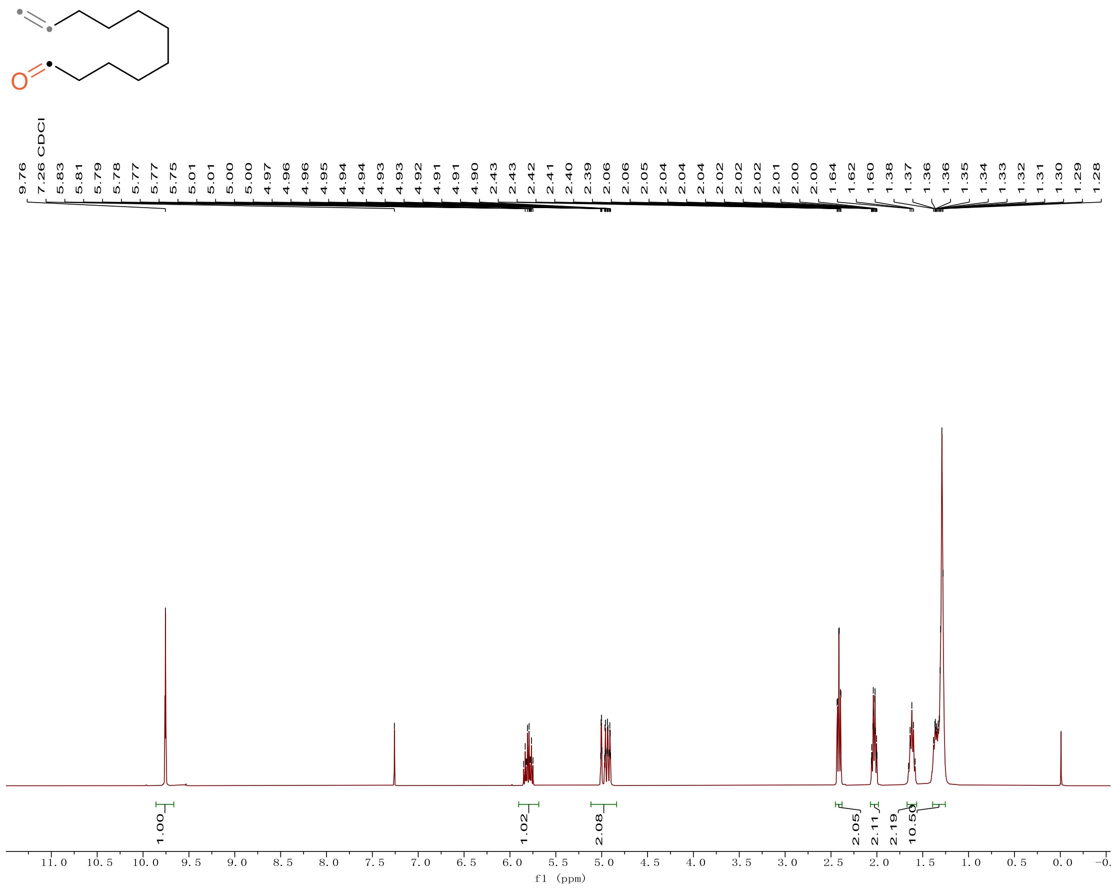

Supplementary Figure 260. ^{1}**H NMR** (400 MHz, Chloroform-*d*) of **79**

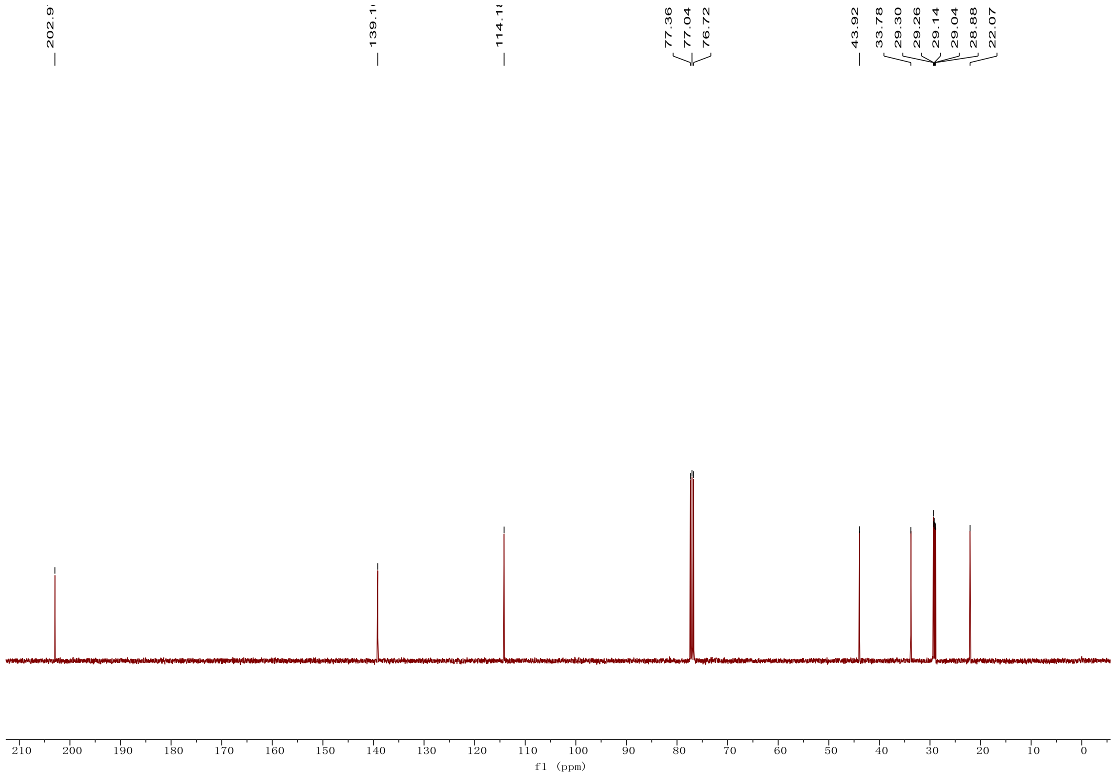

Supplementary Figure 261. ^{13}**C NMR (**101 MHz, Chloroform-*d*) of **79**

9. **References**

1. Chalikidi, P. N., Magkoev, T. T., Gutnov, A. V., Demidov, O. P., Uchuskin, M.G., Trushkov, I. V. & Abaev, V. T. One-step synthesis of triphenylphosphonium salts from (het)arylmethyl alcohols. *J. Org. Chem.* **86**, 9838-9846 (2021).

2. Biosca, M., Salomó, E., de La Cruz-Sánchez, P., Riera, A., Verdaguer, X., Pàmies, O. & Diéguez, M. Extending the substrate scope in the hydrogenation of unfunctionalized tetrasubstituted olefins with Ir-P stereogenic aminophosphine-oxazoline catalysts. *Org. Lett.* **21**, 807-811 (2019).

3. Izquierdo, F., Corpet, M. & Nolan, S. P. The Suzuki–Miyaura reaction performed using a palladium–N-heterocyclic carbene catalyst and a weak inorganic base. Eur. *J. Org. Chem.* **80**, 1920-1928 (2015).

4. Dalton, T., Greßies, S., Das, M., Niehues, M., Schrader, M. L., Gutheil, C., Ravoo, B. J. & Glorius, F. Silver-catalysed hydroarylation of highly substituted styrenes. *Angew. Chem. Int. Ed.* **60**, 8537-8541 (2021).

5. Gottumukkala, A. L., Derridj, F., Djebbar, S. & Doucet, H. Alkenyl bromides: useful coupling partners for the palladium-catalysed coupling with heteroaromatics via a C-H bond activation. *Tetrahedron Lett.* **49**, 2926-2930 (2008).

6. Occhialini, G., Palani, V. & Wendlandt, A. E. Catalytic, contra-thermodynamic positional alkene isomerization. *J. Am. Chem. Soc.* **144**, 145-152 (2022).

7. Kraszewski, K., Tomczyk, I., Drabinska, A., Bienkowski, K., Solarska, R. & Kalek, M. Mechanism of iodine(III)-promoted oxidative dearomatizing hydroxylation of phenols: evidence for a radical-chain pathway. *Chem. Eur. J.* **26**, 11584-11592 (2020).

8. Murai, K., Nakamura, A., Matsushita, T., Shimura, M. & Fujioka, H. C3-symmetric trisimidazoline-catalyzed enantioselective bromolactonization of internal alkenoic acids. *Chem. Eur. J.* **18**, 8448-8453 (2012).

9. Kolb, S. & Werz, D. B. Site-selective hydrogenation/deuteration of benzylic olefins enabled by electroreduction using water. *Chem. Eur. J.* **29**, e202300849 (2023).

10. Anderson, S. & Drakenberg, T. Barriers to internal rotation in neopentylbenzenes substituted on the benzyl group. A ^{13}C NMR band shape study. *Org. Magn. Reson*. **21**, 730-744 (1983).

11. Volla, C. M. R., Marković, D., Dubbaka, S. R. & Vogel, P. Ligandless iron-catalyzed desulfinylative C–C allylation reactions using grignard reagents and alk-2-enesulfonyl chlorides. *Eur. J. Org. Chem.* **2009,** 6281-6288 (2009).

12. Yasushi, Y., Hitoshi, N., Man, S. S., Yayoi, Y. & Kensaku, T. Synthesis of novel thermally reversible photochromic spiro[adamantane-2,7′(6′H)-benzothiophene]. *Bull. Chem. Soc. Jpn*. **76**, 355-361 (2003).

13. Ponzano, S., Berozzi, F., Mengatto, L., Dionisi, M., Armirotti, A., Romeo, E., Berteotti, A., Fiorelli, C., Tarozzo, G., Reggiani, A., Duranti, A., Tarzia, G., Mor, M., Cavalli, A., Piomelli, D. & Bandiera, T. Synthesis and structure–activity relationship (SAR) of 2-methyl-4-oxo-3-oxetanylcarbamic acid esters, a class of potent N-acylethanolamine acid amidase (NAAA) inhibitors. *J. Med. Chem.* **56**, 6917-6934 (2013).

14. Hill, J. & Crich, D. Synthesis of o-tert-butyl-N,N-disubstituted hydroxylamines by N–O bond formation. *Org. Lett.* **23**, 6396-6400 (2021).

15. Pan, X. Q., Wang, L., Zou, J. P. & Zhang, W. Manganese(III)-mediated phosphinoyl radical reactions for stereoselective synthesis of phosphinoylated tetrahydronaphthalenes. *Chem. Commun.*

47*,* 7875-7877 (2011).
16. Johnson, K. A., Biswas, S. & Weix, D. J. Cross-electrophile coupling of vinyl halides with alkyl halides. *Chem. Eur. J.* **22***,* 7399-7402 (2016).
17. Herath, A., Thompson, B. B. & Montgomery, J. Catalytic intermolecular reductive coupling of enones and alkynes. *J. Am. Chem. Soc.* 129*,* 8712-8713 (2007).
18. Huang, Z., Guan, R., Shanmugam, M., Bennett, E. J., Robertson, C. M., Brookfield, A., McInnes, E. J. L. & Xiao, J. Oxidative cleavage of alkenes by O_2 with a non-heme manganese catalyst. *J. Am. Chem. Soc.* **143***,* 10005-10013 (2021).
19. Affo, W., Ohmiya, H., Fujioka, T., Ikeda, Y., Nakamura, T., Yorimitsu, H., Oshima, K., Imamura, Y., Mizuta, T. & Miyoshi, K. Cobalt-catalyzed trimethylsilylmethylmagnesium-promoted radical alkenylation of alkyl halides: a complement to the heck Reaction. *J. Am. Chem. Soc.* **128***,* 8068-8077 (2006).
